# Chinese Medicine for Psoriasis Vulgaris Based on Syndrome Pattern: A Network Pharmacological Study

**DOI:** 10.1155/2020/5239854

**Published:** 2020-04-28

**Authors:** Dongmei Wang, Chuanjian Lu, Jingjie Yu, Miaomiao Zhang, Wei Zhu, Jiangyong Gu

**Affiliations:** ^1^Dermatology Hospital of Southern Medical University, Guangzhou 510091, China; ^2^Guangdong Provincial Academy of Chinese Medical Sciences, Guangzhou 510006, China; ^3^The Second Clinical College, Guangzhou University of Chinese Medicine, Guangzhou 510006, China; ^4^Department of Biochemistry, School of Basic Medical Science, Guangzhou University of Chinese Medicine, Guangzhou 510006, China

## Abstract

**Background:**

The long-term use of conventional therapy for psoriasis vulgaris remains a challenge due to limited or no patient response and severe side effects. Complementary and alternative treatments such as traditional Chinese medicine (TCM) are widely used in East Asia. TCM treatment is based on individual syndrome types. Three TCM formulae, Compound Qingdai Pills (F1), Yujin Yinxie Tablets (F2), and Xiaoyin Tablets (F3), are used for blood heat, blood stasis, and blood dryness type of psoriasis vulgaris, respectively.

**Objectives:**

To explore the mechanism of three TCM formulae for three syndrome types of psoriasis vulgaris.

**Methods:**

The compounds of the three TCM formulae were retrieved from the Psoriasis Database of Traditional Chinese Medicine (PDTCM). Their molecular properties of absorption, distribution, metabolism, excretion and toxicity (ADME/T), and drug-likeness were compared by analyzing the distribution of compounds in the chemical space. The cellular targets of the compounds were predicted by molecular docking. By constructing the compound-target network and analyzing network centrality, key targets and compounds for each formula were screened. Three syndrome types of psoriasis vulgaris related pathways and biological processes (BPs) were enriched by the Database for Annotation, Visualization, and Integrated Discovery (DAVID) v6.8.

**Results:**

The compounds of the three formulae exhibited structural diversity, good drug-like properties, and ADME/T properties. A total of 72, 97 and 85 targets were found to have interactions with compounds of F1, F2, and F3, respectively. The three formulae were all related to 53 targets, 8 pathways, 9 biological processes, and 10 molecular functions (MFs). In addition, each formula had unique targets and regulated different pathways and BPs.

**Conclusion:**

The three TCM formulae exhibited common mechanisms to some extent. The differences at molecular and systems levels may contribute to their unique applications in individualized treatment.

## 1. Introduction

Psoriasis vulgaris is the most common type of psoriasis and is characterized as sharply marginated, erythematous patches or plaques with silvery-white micaceous scales [1, 2]. The prevalence of psoriasis ranges from 0.91% to 8.5% across the world [3]. It causes a significant burden to patients including physical and psychological symptoms. Due to complex and unclear pathogenic factors, the treatment options for psoriasis remain unsatisfactory. Over the last decade, expanded understanding of psoriasis pathogenesis has led to the development of new systemic agents such as biological drugs that have revolutionized the treatment of psoriasis [4, 5]. Moreover, the use of complementary and alternative medicine including herbal medicine for subjects with psoriasis is increasing [6, 7]. Various traditional Chinese medicines (TCMs) are widely used to treat psoriasis on the basis of the unique clinical therapeutic theory and successful clinical applications [8–12]. The clinical effectiveness of TCMs for psoriasis has been validated by controlled clinical trials [13] and meta-analysis [14]. Controlled trials also demonstrate that the combination of TCM with traditional therapies for psoriasis is more efficacious [15].

In the view of the therapeutic potential of Chinese medicine, the diagnosis and treatment of psoriasis vulgaris are based on a group of individual symptoms, which is called as syndrome type or patterns [16, 17]. Psoriasis vulgaris is classified as three main blood-related phenotypes in Chinese medicine: blood heat, blood stasis, and blood dryness [8]. Blood heat is characterized by bright red papules with pruritus. Auspitz's sign appears following removal of the scale. The feature of blood dryness type manifests as light red and patchy particles, covered with plenty of dry silvery-white scales. With regard to blood stasis type, dull red, hard, and thick plaques are covered by thick, dry, silvery-white scales, with itchiness [18]. The syndrome type classification of patients with psoriasis vulgaris may vary according to the changes of the symptoms. The distribution of the three syndromes is closely related to the stage of psoriasis vulgaris. Blood heat, blood dryness, and blood stasis syndromes are commonly noted in progressive stage, extinction stage, and stationary stage of this disease, respectively [8]. Therefore, different syndrome types of psoriasis require different therapeutic regimens.

TCM formulae are mainly oral systematic drugs and the selection of appropriate treatment is based on the different syndrome type of psoriasis. These formulae can be used in the form of Chinese herbal medicine decoction or commercial products, such as pills and tablets.

Three commercially available herbal products are frequently used in the oral treatment of psoriasis vulgaris. According to the Chinese Pharmacopoeia (2015 Edition), *Compound Qingdai Pills* (F1), *Yujin Yinxie Tablets* (F2), and *Xiaoyin Tablets* (F3) are commonly used for blood heat, blood stasis, and blood dryness type of psoriasis vulgaris, respectively (Table 1). These formulae have been investigated in several clinical and experimental studies. Evidences from experimental studies suggest that F1 decreases the expression level of c-myc in keratinocytes, which can inhibit the hyperproliferation of these cells [19, 20]. Moreover, it has been reported that F1 modulates immunological function [21]. A randomized controlled trial indicates that F1 could enhance the clinical efficacy and increase the number of patients who achieve the psoriasis area and severity index 60 (PASI 60) as an add-on therapy to acitretin and mometasone furoate [22]. An *in vivo* study demonstrates that F2 could decrease the expression levels of keratinocyte growth factor and amphiregulin in Balb/c nude mice and downregulate the expression levels of *KGF* and *AREG* mRNAs [23]. In addition, F2 could inhibit the phagocytosis process of macrophages and thus modulates immunological response [24]. In a noncontrolled study, F2 has been proven to improve the clinical outcome of 313 patients with psoriasis [25]. The therapeutic efficacy of F3 for 60 patients with psoriasis is validated by comparison with another TCM formula (Yin Xie Ping Granule) [26, 27].

Each TCM formula consists of several herbs (Table 1). Therefore, the presence of multiple components and their interactions with multiple targets make it difficult to elucidate the mechanism of action (MoA) of TCM. In recent years, network pharmacology is rapidly becoming a promising tool to reveal the pharmacodynamic material basis and systematic features of TCM [28–30]. In this work, a network pharmacology study was performed to explore the MoA of three TCM formulae for different syndrome types of psoriasis by integrating ADME/T (absorption, distribution, metabolism, excretion, and toxicity) analysis, target prediction, drug-target network construction, and gene set enrichment analysis. The results indicated that multiple compounds of each formula could interact with multiple cellular targets and therefore regulate multiple pathways and BPs. In addition, three TCM formulae were associated with the same group of targets, pathways, BPs, and MFs, while each TCM formula also exhibited unique profile. Our findings will provide a preliminary basis for syndrome differentiation and treatment of psoriasis by TCM.

## 2. Materials and Methods

### 2.1. Data Collection

The herbal compositions of the three formulae were collected from the Chinese Pharmacopoeia (2015 Edition). Subsequently, the chemical identifications, physicochemical properties, and 3D structures of compounds (Table S1) which existed in each herb were retrieved from the Psoriasis Database of Traditional Chinese Medicine (PDTCM) [31]. The structures of the FDA-approved drugs for psoriasis were downloaded from DrugBank [32]. The psoriasis-associated proteins (Table S2) were collected from three sources: the targets of psoriasis drugs from DrugBank, the psoriasis-related targets from the Therapeutic Target Database (TTD) [33] and PDTCM, and the targets summarized from the literature which have been reported in a previous work [34].

### 2.2. Analysis of ADME/T Properties, Drug-Likeness, and Distribution in the Chemical Space

**Figure 1:**
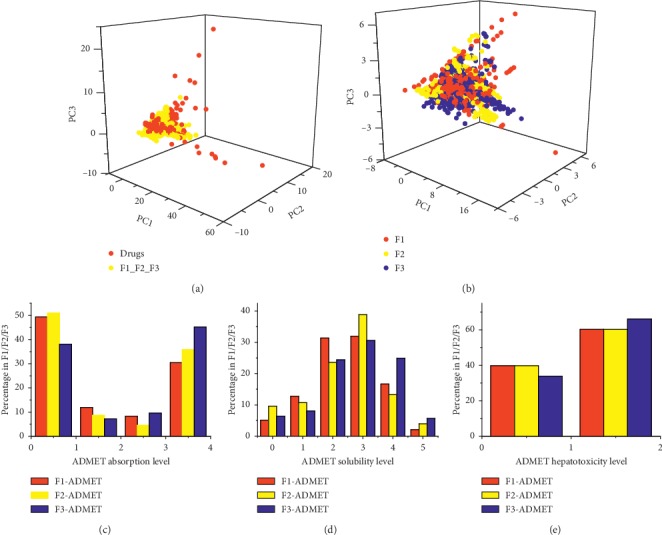
Drug-likeness and ADME/T properties of compounds in three TCM formulae for psoriasis. (a) Distributions in chemical space of FDA-approved drugs for psoriasis and compounds in three TCM formulae. (b) Distributions in chemical space of compounds in each TCM formula. Level of ADME/T absorption (c), solubility (d), and hepatotoxicity (e).

**Figure 2:**
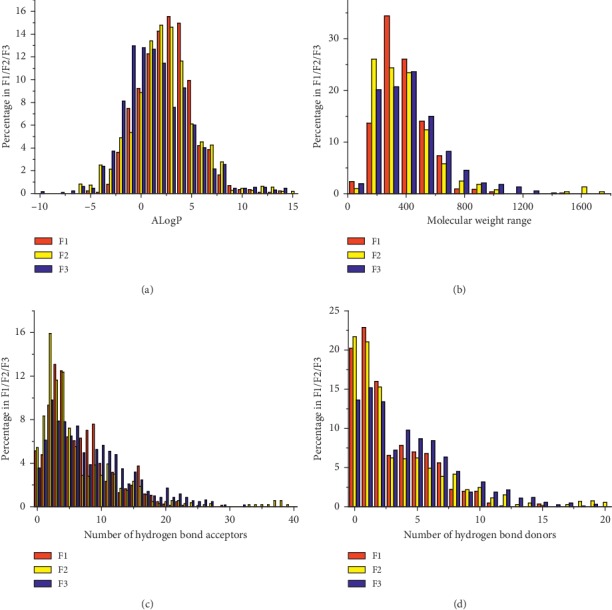
Distributions of four drug-like properties. AlogP (a), molecular weight (b), number of hydrogen bond acceptors (c), and that of donors (d).

The ADME/T descriptors (Aqueous Solubility, Blood Brain Barrier Penetration, CYP2D6 Binding, Hepatotoxicity, Intestinal Absorption, and Plasma Protein Binding) of the compounds in the three TCM formulae were calculated by ADME/T Descriptors module of Discovery Studio (DS) v2.5 (Figure S1). A total of eight molecular descriptors (molecular hydrophobicity (AlogP), molecular weight, number of hydrogen bond donors, number of hydrogen bond acceptors, number of rotatable bonds, number of rings, number of aromatic rings, and molecular fractional polar surface area) of the compounds and drugs were calculated by the General Purpose module of DS. Principal component analysis (PCA) was conducted in the Library Analysis module of DS by using the aforementioned molecular descriptors. The resulting three principal components (PC1, PC2, and PC3) were used to reduce the dimension of molecular descriptors, which can explain 89.5% of the total variance of these molecular descriptors. Subsequently, the distributions of compounds and drugs in the chemical space were illustrated by using these three principal components as coordinates (Figure 1). The distributions of four drug-like properties (AlogP, molecular weight, number of hydrogen bond acceptors, and that of and donors; see Table 2) were illustrated in Figure 2.

### 2.3. Molecular Docking

The compound-target interaction was simulated by molecular docking. The X-ray or NMR structures were downloaded from the RCSB Protein Data Bank and treated to be suitable for molecular docking by AutoDock v4.2.6 [35] according to the protocols described in a previous work [34]. The energy grid was a 30 × 30 × 30 Å cube centered on the occupied space of the original ligand with a spacing of 0.375 Å between the grid points. The Lamarckian genetic algorithm (LGA) was used to optimize the conformation of each compound in the binding pocket. The parameters for LGA were listed as follows: the number of individuals in population, the maximum number of energy evaluations, the maximum number of generations, and the rate of gene mutation set as 150, 2.5 × 10^7^, 2.7 × 10^4^, and 0.02, respectively. Other parameters were set to default. The binding energy calculated by AutoDock was used to evaluate the affinity between each compound and target protein.

### 2.4. Construction of Compound-Target Network

The binding energy was used to evaluate the affinity between the compound and the target. In general, the binding energy can be transferred to the inhibition constant *K*_*i*_. In this work, the threshold of binding energy was set to −12.28 kcal/mol (the estimated inhibition constant *K*_*i*_ was approximately 1 nM) for each compound and target (Table S3).

**Figure 3:**
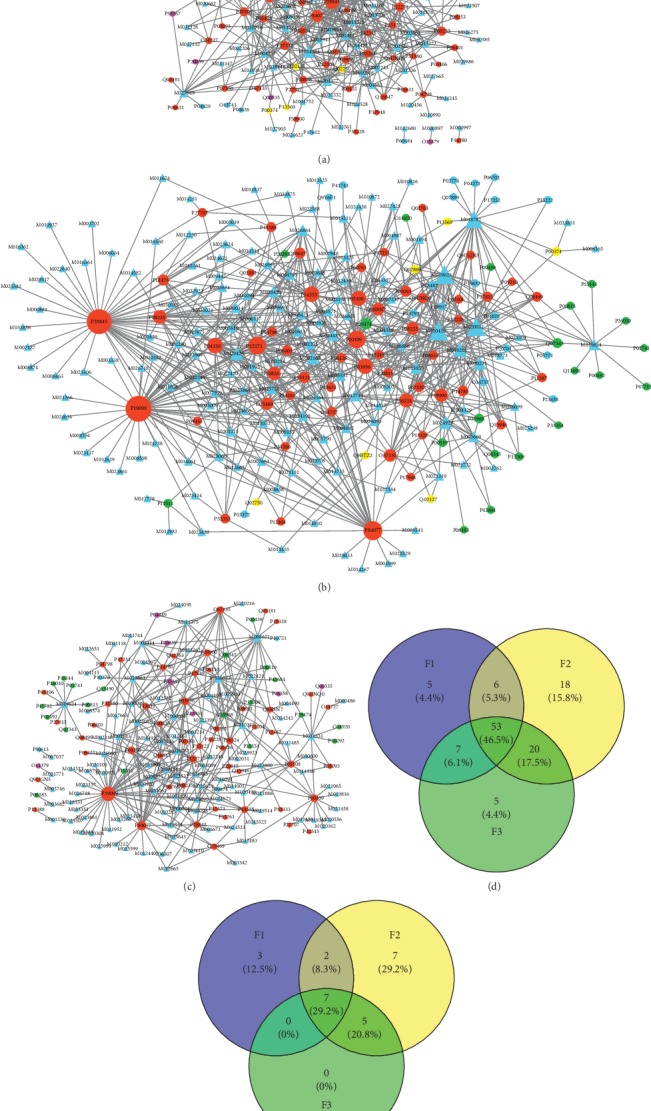
Compound-target networks. CTN for F1 (a), F2 (b), and F3 (c). The ellipse and triangle represent target and compounds in three TCM formulae, respectively. The color of target node represents the correlation between the target and three TCM formulae. If a target is related to only one TCM formula, the color is blue. If a target is related to F1 and F2, or F2 and F3, or F1 and F3, the color is yellow, green, and purple, respectively. And if a target is related to all three TCM formulae, the color is red. (d) Venn diagram of common targets in each formula. (e) Venn diagram of common key targets in each TCM formula.

Subsequently, the data of the compound-target interactions was imported into Cytoscape v3.6.0 [36] to construct the compound-target network (CTN) for each TCM formula (Figure 3). The network centralities of nodes (compounds and targets) were calculated by the Network Analyzer module of Cytoscape [37]. The degree centrality was used to evaluate the importance of the targets and compounds in CTNs (Tables 3 and 4).

### 2.5. Gene Set Enrichment Analysis by DAVID

**Figure 4:**
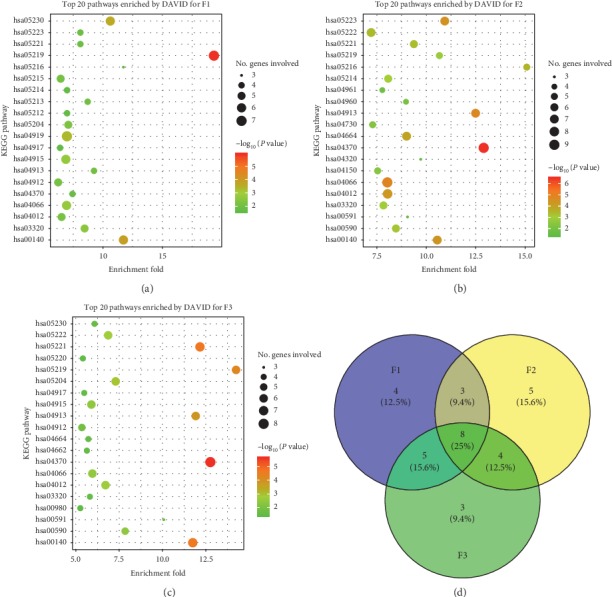
Top twenty KEGG pathways enriched by DAVID. (a), (b), and (c) for F1, F2, and F3, respectively. Venn diagram of common pathways (d) for three TCM formulae.

**Figure 5:**
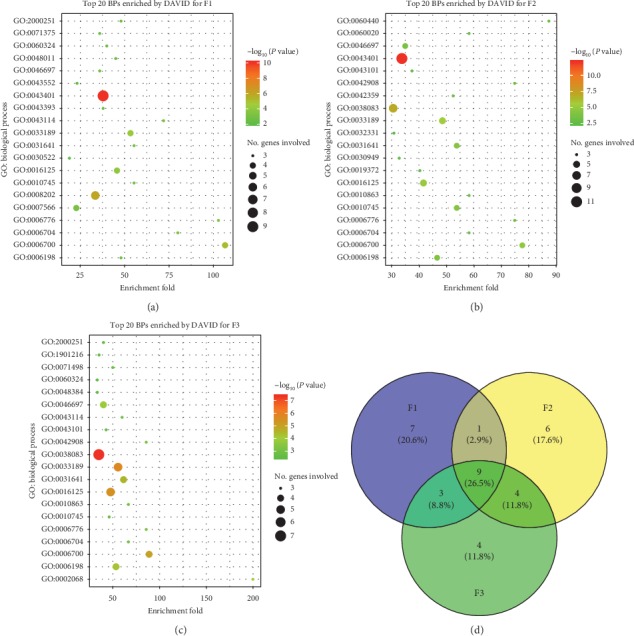
Top twenty GO biological processes enriched by DAVID. (a), (b), and (c) for F1, F2, and F3, respectively. Venn diagram of common biological processes (d) for three TCM formulae.

**Figure 6:**
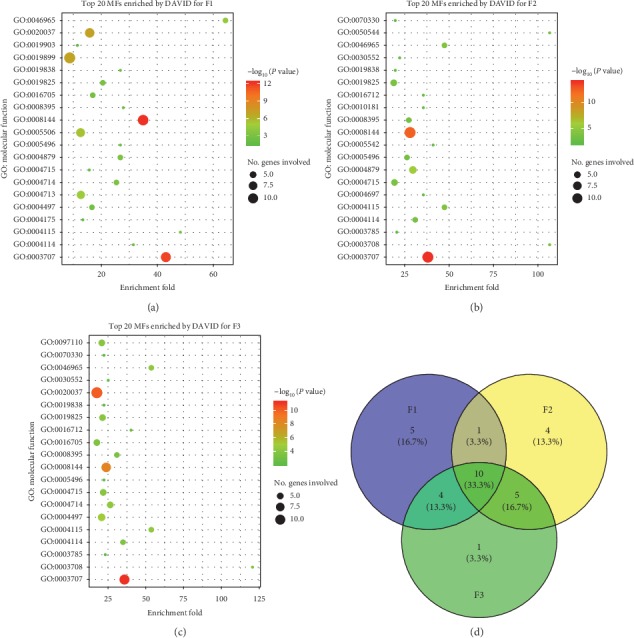
Top twenty GO molecular functions enriched by DAVID. (a), (b), and (c) for F1, F2, and F3, respectively. Venn diagram of common molecular functions (d) for three TCM formulae.

The gene set enrichment analysis was carried out on the web service of the Database for Annotation, Visualization, and Integrated Discovery (DAVID) v6.8 [38]. The gene list for each TCM formula was transferred from the CTN. The KEGG pathways and the Gene Ontology (GO) terms (biological processes and molecular functions) were enriched for each gene list by using the following parameters: the threshold of enriched genes and *P* value were 3 and 0.05, respectively (Table S4). The top twenty GO terms and KEGG pathways which were significantly associated with the targets were further illustrated by bubble diagrams (Figures 4–6) drawn by the ggplot2 package [39] in R 3.5.1.

## 3. Results

### 3.1. Compounds in Three TCM Formulae Have Good ADME/T and Drug-Like Properties

The three TCM formulae (F1, F2, and F3) contained 857, 1084, and 1295 compounds, respectively. The statistics of the molecular properties demonstrated that the majority of compounds obeyed Lipinski's rule of five (Ro5) [40], which was frequently used to evaluate the drug-likeness of the compounds (Table 2). The PCA results of the FDA-approved drugs and compounds in the three TCM formulae indicated a large overlap between their distributions in the chemical space (Figure 1(a)), which also suggested that these compounds exhibited good drug-like properties.

These compounds also had good ADME/T properties, which can promote their oral bioavailability (Figure S1). The ADME/T and physicochemical properties of the compounds in the three TCM formulae were similar. First, the compounds of each TCM formula exhibited approximately even distribution in the chemical space (Figure 1(b)). The scattered distributions indicated that these compounds appeared to have some degree of structural diversity. Second, the ADME/T levels of absorption, solubility, and hepatotoxicity in each TCM formula did not have significant differences (Chi-squared test, *P* value >0.05) (Figures 1(c)–1(e)). Third, four molecular properties (with the exception of the number of hydrogen bond acceptors) of Ro5 for each TCM formula exhibited broadly similar distributions (Figure 2). *P* values of the Chi-squared tests for the distributions of molecular hydrophobicity (AlogP), molecular weight, number of hydrogen bond acceptors, and number of hydrogen bond donors of the compounds between F1 and F2, F1 and F3, and F2 and F3 were 0.0056, 0.049, 0.13; 0.024, 0.12, 0.14; 4.8*E* − 12, 8.7*E* − 15, 7.4*E* − 11; and 0.20, 0.31, 0.27, respectively (Figure 2). In general, F2 had better similarity with F3. Meanwhile, the compounds in three TCM formulae exhibited significant differences in the distributions of hydrogen bond acceptors. The compounds of F1 had larger average value of AlogP than the compounds of F2 and F3. Therefore, similar ADME/T and physicochemical properties enabled the targeting of these compounds in the same group of proteins and the regulation of psoriasis-associated biological processes. Due to different properties and structural diversity, several compounds could also bind to different targets, which would lead to different molecular mechanisms for the treatment of different syndrome types of psoriasis by each TCM formula.

### 3.2. Compounds in Three TCM Formulae Have Multiple Targets

The TCM formula had multiple components and can interact with multiple cellular targets. Therefore, multiple biological processes (BPs) would be regulated in a holistic manner. The characteristics of “multicomponents and multitargets” of TCM can be illustrated by a bipartite CTN, which is composed of compounds and proteins linked by compound-target interactions [41, 42]. The CTN of F1 (Figure 3(a)) comprised 170 nodes (98 compounds and 72 targets) and 420 edges. The CTN of F2 (Figure 3(b)) consisted of 241 nodes (144 compounds and 97 targets) and 689 edges. A total of 189 nodes (104 compounds and 85 targets) and 354 edges were found in the CTN of F3 (Figure 3(c)). A total of 53 common targets were associated with all three TCM formulae. In addition, a total of 6, 20, and 7 common targets were associated with F1 and F2, F2 and F3 and, and F1 and F3, respectively (Figure 3(d)).

Typically, a compound would interact with several proteins and the polypharmacological effects are highly enriched in those compounds with large degree centrality in CTN [41, 43, 44]. Therefore, key targets (Table 3) and compounds (Table 4) can be identified by degree centrality in CTNs for F1, F2, and F3, respectively (Figures 3(a)–3(c)). These key targets may participate in the same BP or a group of related BPs. In addition, multiple compounds can bind to a common protein and exert a synergistic or antagonistic effect [45].

The ingredients of F1, F2, and F3 have affinities with 12, 21, and 12 key targets (degree of target node ≥10), respectively (Table 3). Seven common key targets (Figure 3(e)) were associated with all three TCM formulae as follows: Glucocorticoid receptor (UniProt: P04150), Adrenodoxin (UniProt: P10109), Retinaldehyde-binding protein 1 (UniProt: P12271), Phospholipase A2 (UniProt: P14555), Cytochrome P450 11B2 (UniProt: P19099), Corticosteroid 11-beta-dehydrogenase isozyme 1 (UniProt: P28845), and ADP-ribosylation factor 1 (UniProt: P84077). These proteins would play critical roles in the treatment of psoriasis. With the exception of common key proteins, F1 and F2 had 3 and 7 unique key targets, respectively. However, F3 was an exceptional case. The target set of F3 was a part of that of F2 (Figure 3(e)), which was in accordance with their clinical applications.

These key proteins would play critical roles in the treatment of psoriasis. First, three proteins (P04150, P10109, and P28845) were involved in metabolism and signaling of sterol and steroid. For example, Corticosteroid 11-beta-dehydrogenase reversibly converts cortisol to the inactive metabolite cortisone and is related to glucocorticoid biosynthetic process (Gene Ontology (GO): 0006704) [46]. Adrenodoxin is essential for the synthesis of various steroid hormones and participates in the reduction of mitochondrial cytochrome P450 for steroidogenesis [47, 48]. Glucocorticoid receptor affects inflammatory responses, cellular proliferation, and differentiation [49–51]. Evidence indicates that decreased expression of Glucocorticoid receptor may play an important role in the degeneration of keratinocytes in patients with psoriasis vulgaris [49]. Second, Phospholipase A2 is implicated in lipid signaling and inflammatory diseases [52], and the activity of phospholipase A2 in human psoriatic skin is regulated by systematic treatment with a retinoic acid derivative [53]. Third, Retinaldehyde-binding protein 1 is a soluble retinoid carrier and participates in the regeneration of active 11-cis-retinol and 11-cis-retinaldehyde, which presumably leads to disruption of retinal vitamin-A metabolism (GO: 0006776) [54]. Fourth, ADP-ribosylation factor 1 is a GTP-binding protein that functions as an allosteric activator of ADP-ribosyltransferase. This multifunctional protein can regulate cell proliferation, migration, and fusion [55]. Finally, Cytochrome P450 (CYP) is the sole enzyme responsible for the production of aldosterone in humans [56] and participates in the metabolism of therapeutic drugs, fatty acids, eicosanoids, sterols, steroids, vitamin A, and vitamin D [57–60]. It has been reported that, in some skin diseases, for example, in psoriasis, the expressions of many types of CYP are elevated, and CYP is regarded as a target in the development of drugs for skin diseases [59].

Key compounds (Table 4) for each TCM formula were screened by applying a somewhat arbitrary threshold (the degree in CTN was greater than or equal to 10). F1 and F2 had a common key compound ((24R)-24-ethylcholest-4-en-3, 6-dione). F2 and F3 contained three common key compounds (*β*-Carotene, 5-Phenylpentan-1,3, 4-triamine, and Clerosterol). A total of 14 key compounds were noted for F1. A total of eight key compounds were separated from *Schisandra sphenanthera*, which is worthy of attention. Other four herbs (*Arnebia euchroma*, *Dryopteris crassirhizoma*, *Portulaca oleracea*, and *Angelica dahurica*) contained three, two, and one key compounds, respectively. F2 had 17 key compounds from seven herbs. F3 had 8 key compounds from five herbs.

There were 14 key compounds of F1. The biological activities related to the pathogenesis of psoriasis of several key compounds are known. For example, Schinalactone A has significant cytotoxicity against SK-BR-3, PANC-1, and A-549 cell lines with IC50 values of 5.2, 5.9, and 17.7 *μ*M, respectively [61]. Ergone exhibits inhibitory activity of nitric oxide production in RAW264.7 cells stimulated by lipopolysaccharide with the IC50 value of 28.96 *μ*M [62].

F2 had 17 key compounds from seven herbs. Luteoxanthin has antiproliferative effect in combination with the anticancer drug epirubicin [63]. 5*α*-Stigmastan-3, 6-dione has anti-inflammatory effect [64]. *β*-Carotene has close relationship with psoriasis [65] and is an effective and safe treatment for patients with mild, chronic, plaque-type psoriasis [66]. Patients with psoriasis have an increased risk of other health problems, and observational studies suggest that *β*-Carotene might help prevent these conditions, such as type 2 diabetes [67], rheumatoid arthritis [68], asthma [69], and metabolic syndrome [70]. Ergosterol peroxide suppresses inflammatory responses in RAW264.7 macrophages [71] and has *in vitro* antiproliferative activity [72]. It also shows anticancer activity by downregulation of the *β*-catenin pathway in colorectal cancer [73, 74]. Campesterol can inhibit proliferation and has anti-inflammatory, antibacterial, and anticancer effects [75]. Sitosterols may be related to cholesterol metabolism or anti-inflammatory effects via interference with prostaglandin metabolism [76]. Clerosterol inhibited the growth of A2058 cells with an IC50 of 150 *μ*M [77]. Lutein has antitumor and antiproliferative activities [78, 79].

F3 had 8 key compounds from five herbs. 4′-O-methylochnaflavone is a bioflavonoid which shows suppressive activity against lymphocyte proliferation [80]. Auroxanthin [81] and Pyropheophorbide [82] have antioxidant activity.

These key proteins were involved in the pathogenesis of psoriasis vulgaris and would be promising targets for the discovery of novel drugs. The key compounds for each TCM formula could play important roles in the treatment of this disease and contribute to the pharmacodynamic material basis. Appropriate optimization of these compounds may provide new leads.

### 3.3. TCM Formula Systematically Regulates Human Body

The compounds of each TCM formula can interact with different sets of cellular targets and subsequently regulate different biological processes. The latter were presented by gene set enrichment analysis (GSEA). In general, the targets of F1 were significantly enriched in 37 pathways in Kyoto Encyclopedia of Genes and Genomes (KEGG), 79 GO biological processes, and 38 GO molecular functions (Table S4 and Figures 4(a), 5(a), and 6(a)). The corresponding numbers of enriched KEGG/GO terms for F2 and F3 were 68, 99, and 54 (Table S4 and Figures 4(b), 5(b), and 6(b)) and 35, 89, and 46 (Table S4 and Figures 4(c), 5(c), and 6(c)), respectively.

The top twenty KEGG/GO terms for each TCM formula were further studied. Eight common KEGG pathways were associated with all three TCM formulae (Figure 4) as follows: four signaling pathways (hsa03320: PPAR signaling pathway, hsa04370: VEGF signaling pathway, hsa04066: HIF-1 signaling pathway, and hsa04012: ErbB signaling pathway), two steroid-related pathways (hsa00140: steroid hormone biosynthesis and hsa04913: ovarian steroidogenesis), and two cancer pathways (hsa05219: bladder cancer and hsa05221: acute myeloid leukemia). In addition to these eight common KEGG pathways, F1 and F2, F2 and F3, and F1 and F3 shared 3, 4, and 5 pathways, respectively. F1, F2, and F3 were associated with 4, 5, and 3 other unique pathways, respectively; see Figure 4(d).

A total of 9 GO biological processes were associated with all three TCM formulae (Figure 5) as follows: sterol metabolic process (GO:0016125), glucocorticoid biosynthetic process (GO:0006704), C21-steroid hormone biosynthetic process (GO:0006700), response to vitamin A (GO:0033189), vitamin A metabolic process (GO:0006776), cAMP catabolic process (GO:0006198), negative regulation of macrophage derived foam cell differentiation (GO:0010745), regulation of myelination (GO:0031641), and decidualization (GO:0046697). These findings were in accordance with the result of KEGG pathway enrichment analysis. The associations between the KEGG pathways or the GO biological processes and psoriasis were summarized in Table S4.

Other than nine common GO biological processes, F1 and F2, F2 and F3, and F1 and F3 shared 1, 4, and 3 BPs, respectively. F1, F2, and F3 were associated with 7, 6, and 4 other unique BPs, respectively (Figure 5(d)).

With regard to molecular functions, 10 GO terms were associated with all three TCM formulae (Figure 6) as follows: retinoid X receptor binding (GO:0046965), 3′,5′-cyclic-AMP phosphodiesterase activity (GO:0004115), steroid hormone receptor activity (GO:0003707), drug binding (GO:0008144), 3′,5′-cyclic-nucleotide phosphodiesterase activity (GO:0004114), steroid hydroxylase activity (GO:0008395), steroid binding (GO:0005496), growth factor binding (GO:0019838), oxygen binding (GO:0019825), and nonmembrane spanning protein tyrosine kinase activity (GO:0004715). These molecular functions had close relationships with the aforementioned KEGG pathways and GO biological processes.

Three TCM formulae were associated with 8 common pathways, 9 common biological processes, and 10 common molecular functions (Figures 4(d), 5(d), and 6(d)). These findings indicated that the treatment of different syndrome types of psoriasis by three TCM formulae was associated with common mechanisms to some extent. Moreover, several unique factors were evident. For example, the thyroid hormone signaling pathway (hsa04919), the mTOR signaling pathway (hsa04150), and the B cell receptor signaling pathway (hsa04662) were only associated with F1, F2, and F3, respectively (Figure 4). With regard to the biological processes regulated by the three TCM formulae, great differences were found (Figure 5). Seven BPs, namely, cellular response to peptide hormone stimulus (GO: 0071375), embryo implantation (GO: 0007566), intracellular receptor signaling pathway (GO: 0030522), neurotrophin TRK receptor signaling pathway (GO: 0048011), positive regulation of phosphatidylinositol 3-kinase activity (GO: 0043552), regulation of protein binding (GO: 0043393), and steroid metabolic process (GO: 0008202), were only associated with F1. Six BPs, including Bergmann glial cell differentiation (GO: 0060020), lipoxygenase pathway (GO: 0019372), negative regulation of chondrocyte differentiation (GO: 0032331), positive regulation of vascular endothelial growth factor receptor signaling pathway (GO: 0030949), trachea formation (GO: 0060440), and vitamin D metabolic process (GO: 0042359), were only associated with F2. Four BPs, namely, cellular response to fluid shear stress (GO: 0071498), glandular epithelial cell development (GO: 0002068), positive regulation of neuron death (GO: 1901216), and retinoic acid receptor signaling pathway (GO: 0048384), were only associated with F3. It is noteworthy that the vitamin D metabolic process (GO: 0042359) was only associated with F2. Vitamin D plays an essential role in cell proliferation, differentiation, apoptosis, and angiogenesis. Reduced vitamin D levels in psoriatic patients correlated with psoriasis duration. Vitamin D is highly effective in the treatment of psoriasis. Calcipotriol and other vitamin D analogues are frequently used for the treatment of psoriasis due to their immunoregulating and antiproliferation activity [83]. This evidence indicated that vitamin D was notably required for the treatment of the blood stasis type of psoriasis vulgaris.

## 4. Discussion

The TCM formula is a complicated drug system; thus complex interactions between multicomponents and multitargets can lead to the regulation of multiple pathways and biological processes. In clinical practice of TCM, the selection of an appropriate formula is particularly important. Most commonly, the remedy will be modified along with the change of the syndrome pattern of the disease. In this work, network pharmacology was adopted to explore the therapeutic mechanisms of three TCM formulae which were indicated for different syndrome patterns of psoriasis vulgaris. Similarities and differences among all three TCM formulae were both identified at different scales.

The majority of the ingredients exhibited good ADME/T and drug-like properties, which provided the diversity and effectiveness of pharmacodynamic material basis for each TCM formula. A number of compounds have demonstrated affinities for multiple cellular targets. A total of 98 compounds of F1 were associated with 72 targets. The targets of F1 were significantly enriched in 37 KEGG pathways, 79 GO BPs, and 38 GO MFs. A total of 144 compounds of F2 were associated with 97 targets. The targets of F2 were significantly enriched in 68 KEGG pathways, 99 GO BPs, and 54 GO MFs. A total of 104 compounds of F3 were associated with 85 targets. The targets of F3 were significantly enriched in 35 KEGG pathways, 89 GO BPs, and 46 GO MFs. Although the presence of common compounds in three TCM formulae was rare, considerable intersections were noted in their targets, including 53 common proteins (red nodes in Figures 3(a)–3(c)) and 7 key targets. Meanwhile, F1 and F2, F2 and F3, and F1 and F3 shared 3, 4, and 5 KEGG pathways, 1, 4, and 3 GO BPs, and 1, 5, and 4 GO MFs, respectively. In addition, F1, F2, and F3 were associated with 5, 18, and 5 unique targets, 4, 5, and 3 KEGG pathways, 7, 6, and 4 GO BPs, and 5, 4, and 1 GO MFs, respectively.

All three TCM formulae can regulate steroid hormone biosynthesis, PPAR signaling pathway, VEGF signaling pathway, HIF-1 signaling pathway, and ErbB signaling pathway. Only F1 can regulate thyroid hormone signaling pathway. Compared with F1, F2 can regulate mTOR signaling pathway and Aldosterone-regulated sodium reabsorption alone. F3 can regulate B cell receptor signaling pathway uniquely. Seven common key targets (Glucocorticoid receptor, Adrenodoxin, Retinaldehyde-binding protein 1, Phospholipase A2, Cytochrome P450 11B2, Corticosteroid 11-beta-dehydrogenase isozyme 1, and ADP-ribosylation factor 1) were associated with all three TCM formulae. F1 and F2 and F2 and F3 had 2 and 5 common targets, respectively. However, the targets of F1 did not overlap except the above mentioned 7 key proteins.

In clinical practice, blood heat, blood dryness, and blood stasis syndromes are commonly seen in progressive stage, extinction stage, and stationary stage, respectively. The results indicated the underlying mechanisms with regard to three different aspects. First, a high percentage (46.5%, Figure 3(d)) of the targets noted for the three TCM formulae was the same, which was in accordance with the common pathogenesis of psoriasis vulgaris. Second, F1, F2, and F3 exhibited independent MoA by interacting with different sets of targets and by regulating different biological processes. These findings were further supported by the GSEA results (Figures 4(d), 5(d), and 6(d)). Finally, three TCM formulae exhibited common targets between each other, which reflected their associations with different stages of psoriasis vulgaris.

## 5. Conclusions

The clinical practice of TCM involves treatment of blood heat, blood stasis, and blood dryness type of psoriasis vulgaris by *Compound Qingdai Pills*, *Yujin Yinxie Tablets*, and *Xiaoyin Tablets*, respectively. Based on the evidence presented in the current study, the compounds of the three TCM formulae exhibited good ADME/T and drug-like properties. Multiple compounds of each formula can interact with multiple cellular targets and thus regulate multiple pathways and BPs. Three TCM formulae were associated with the same group of targets, pathways, BPs, and MFs, while each TCM formula exhibited unique profile. These findings reveal that different syndrome types of psoriasis vulgaris not only have common pathogenesis to some extent, but also exert inherent differences that require elaborate therapies.

## Figures and Tables

**Table 1:** Herbal compositions of three TCM formulae indicated for psoriasis vulgaris.

Formula	Composition	Syndrome type
Compound Qingdai Pills (F1)	*Baphicacanthus cusia*, *Prunus mume*, *Taraxacum mongolicum*, *Arnebia euchroma*, *Angelica dahurica*, *Salvia miltiorrhiza*, *Dictamnus dasycarpus*, *Dryopteris crassirhizoma*, *Smilax glabra*, *Portulaca oleracea*, *Dioscorea hypoglauca*, *Crataegus pinnatifida*, and *Schisandra sphenanthera*	Blood heat

Yujin Yinxie Tablets (F2)	*Gentiana macrophylla*, *Angelica sinensis*, *Acorus tatarinowii*, *Phellodendron amurense*, *Cyperus rotundus*, *Curcuma wenyujin*, *Curcuma kwangsiensis*, *Strychnos nux-vomica*, *Gleditsia sinensis*, *Prunus persica*, *Carthamus tinctorius*, *Boswellia carterii*, *Rheum palmatum*, *Eupolyphaga sinensis*, *Baphicacanthus cusia*, and *Momordica cochinchinensis*	Blood stasis

Xiaoyin Tablets (F3)	*Rehmannia glutinosa*, *Isatis indigotica*, *Paeonia suffruticosa*, *Paeonia lactiflora*, *Angelica sinensis*, *Sophora flavescens*, *Lonicera japonica*, *Scrophularia ningpoensis*, *Arctium lappa*, *Cryptotympana pustulata*, *Dictamnus dasycarpus*, *Saposhnikovia divaricata*, and *Carthamus tinctorius*	Blood dryness

**Table 2:** Statistics of molecular descriptors of compounds of three TCM formulae.

Formula	ALogP	Molecular weight (Da)	No. of hydrogen bond acceptors	No. of hydrogen bond donors	No. of compounds
F1	2.65 ± 2.69	384.23 ± 163.12	6.54 ± 4.79	2.91 ± 2.91	857
F2	2.37 ± 3.32	428.06 ± 314.44	7.16 ± 8.09	3.86 ± 4.76	1084
F3	1.56 ± 3.37	431.31 ± 230.42	8.19 ± 6.19	4.33 ± 3.87	1295

**Table 3:** Key targets according to CTNs.

Formula	UniProt ID	Degree	Protein name
F1	P19099	41	Cytochrome P450 11B2, mitochondrial
F1	P28845	32	Corticosteroid 11-beta-dehydrogenase isozyme 1
F1	P84077	24	ADP-ribosylation factor 1
F1	P04150	19	Glucocorticoid receptor
F1	P14555	16	Phospholipase A2, membrane associated
F1	P12271	15	Retinaldehyde-binding protein 1
F1	P10109	14	Adrenodoxin, mitochondrial
F1	P11388	14	DNA topoisomerase 2-alpha
F1	P08235	14	Mineralocorticoid receptor
F1	P37231	14	Peroxisome proliferator-activated receptor gamma
F1	P11473	12	Vitamin D3 receptor
F1	P33261	11	Cytochrome P450 2C19
F2	P19099	51	Cytochrome P450 11B2, mitochondrial
F2	P28845	48	Corticosteroid 11-beta-dehydrogenase isozyme 1
F2	P84077	32	ADP-ribosylation factor 1
F2	P10109	28	Adrenodoxin, mitochondrial
F2	P12271	28	Retinaldehyde-binding protein 1
F2	P03956	22	Interstitial collagenase
F2	P14555	22	Phospholipase A2, membrane associated
F2	P04150	22	Glucocorticoid receptor
F2	P05108	21	Cholesterol side-chain cleavage enzyme, mitochondrial
F2	P56524	18	Histone deacetylase 4
F2	P08235	17	Mineralocorticoid receptor
F2	P39900	16	Macrophage metalloelastase
F2	P10826	16	Retinoic acid receptor beta
F2	O67135	14	Acetoin utilization protein
F2	Q8N8N7	14	Prostaglandin reductase 2
F2	P08253	14	72 kDa type IV collagenase
F2	O75469	13	Nuclear receptor subfamily 1 group I member 2
F2	P29474	11	Nitric oxide synthase, endothelial
F2	P15121	11	Aldose reductase
F2	P05093	10	Steroid 17-alpha-hydroxylase/17,20 lyase
F2	P11473	10	Vitamin D3 receptor
F3	P19099	41	Cytochrome P450 11B2, mitochondrial
F3	P84077	20	ADP-ribosylation factor 1
F3	P10109	13	Adrenodoxin, mitochondrial
F3	O67135	13	Acetoin utilization protein
F3	P03956	13	Interstitial collagenase
F3	P12271	12	Retinaldehyde-binding protein 1
F3	P04150	12	Glucocorticoid receptor
F3	P05108	10	Cholesterol side-chain cleavage enzyme, mitochondrial
F3	P56524	10	Histone deacetylase 4
F3	P39900	10	Macrophage metalloelastase
F3	P28845	10	Corticosteroid 11-beta-dehydrogenase isozyme 1
F3	P14555	10	Phospholipase A2, membrane associated

**Table 4:** Key compounds according to CTNs.

Formula	Compound ID	Degree	Chemical name	Herb source
F1	M014384	25	Schinalactone A	*Schisandra sphenanthera*
F1	M018528	19	Schisphendilactone B	*Schisandra sphenanthera*
F1	M004977	16	Dryocrassyl acetate	*Dryopteris crassirhizoma*
F1	M002520	14	*β*-Sitostenone	*Portulaca oleracea*
F1	M002163	13	(24R)-24-ethylcholest-4-en-3,6-dione	*Arnebia euchroma* (F1) and *Gleditsia sinensis* (F2)
F1	M031368	13	Schisanol^a^	*Schisandra sphenanthera*
F1	M014132	12	Schisanol^a^	*Schisandra sphenanthera*
F1	M027649	12	Dahuribirin D	*Angelica dahurica*
F1	M004287	11	Anwuweizic acid^b^	*Schisandra sphenanthera*
F1	M018092	11	Schisphendilactone A	*Schisandra sphenanthera*
F1	M011284	11	Schisandronic acid	*Schisandra sphenanthera*
F1	M020716	11	Anwuweizic acid^b^	*Schisandra sphenanthera*
F1	M013272	10	Ergone	*Arnebia euchroma*
F1	M010568	10	Dammara-18(28),21-diene	*Dryopteris crassirhizoma*
F2	M020429	32	Strychnoflavine	*Strychnos nux-vomica*
F2	M019628	32	Demethoxyguiaflavine	*Strychnos nux-vomica*
F2	M020032	32	N/A	*Strychnos nux-vomica*
F2	M018782	28	Nb-methyl-longicaudata	*Strychnos nux-vomica*
F2	M019201	18	Strychnochrysine^c^	*Strychnos nux-vomica*
F2	M024929	16	Luteoxanthin	*Prunus persica*
F2	M029426	16	5*α*-Stigmastan-3,6-dione	*Gleditsia sinensis*
F2	M010231	14	*β*-Carotene	*Lonicera japonica* (F3) and *Momordica cochinchinensis* (F2)
F2	M015955	13	Strychnochrysine^c^	*Strychnos nux-vomica*
F2	M002163	13	(24R)-24-ethylcholest-4-en-3,6-dione	*Arnebia euchroma* (F1) and *Gleditsia sinensis* (F2)
F2	M012713	13	*α*-Carotene	*Momordica cochinchinensis*
F2	M019624	11	5-Phenylpentan-1,3,4-triamine	*Angelica sinensis*
F2	M026864	11	Ergosterol peroxide	*Cyperus rotundus*
F2	M024435	11	Campesterol	*Prunus persica*
F2	M028420	10	Sitosterol	*Prunus persica*
F2	M002205	10	Clerosterol	*Carthamus tinctorius*
F2	M003669	10	Lutein	*Momordica cochinchinensis*
F3	M008651	18	4′-O-Methylochnaflavone	*Lonicera japonica*
F3	M030533	16	(8R,8′R)-Auroxanthin	*Lonicera japonica*
F3	M010231	14	*β*-Carotene	*Lonicera japonica* (F3) and *Momordica cochinchinensis* (F2)
F3	M019624	11	5-Phenylpentan-1,3,4-triamine	*Angelica sinensis*
F3	M023863	11	Lappaphen-b	*Arctium lappa*
F3	M011831	10	Pyropheophorbide	*Isatis indigotica*
F3	M000604	10	Lappaphen-a	*Arctium lappa*
F3	M002205	10	Clerosterol	*Carthamus tinctorius*

Note that ^a, b, c^ these two compounds were enantiomers.

## Data Availability

Additional data to the manuscript will be available upon request.

## Abbreviations

ADME/T: Absorption, distribution, metabolism, excretion, and toxicity
BPs: Biological processes
CTN: Compound-target network
DAVID: Visualization and Integrated Discovery
F1: Compound Qingdai Pills
F2: Yujin Yinxie Tablets
F3: Xiaoyin Tablets
GO: Gene Ontology
GSEA: Gene set enrichment analysis
KEGG: Kyoto Encyclopedia of Genes and Genomes
LGA: Lamarckian genetic algorithm
PCA: Principal component analysis
PDTCM: Psoriasis Database of Traditional Chinese Medicine
TCM: Traditional Chinese medicine
TTD: Therapeutic Target Database.

## Supplementary Materials

Supplementary Materials Figure S1: ADME/T properties of compounds of three TCM formulae. Table S1: herb composition and compounds of each formula retrieved from PDTCM. Table S2: psoriasis-related proteins. Table S3: compound-target binding energy lower than −12.28 kcal/mol. Table S4: GSEA results for three TCM formulae.
