# Supplementary material for: Chinese Medicine for Psoriasis Vulgaris Based on Syndrome Pattern: A Network Pharmacological Study
Source: Evid Based Complement Alternat Med. 2020 Apr 28;2020:5239854. doi: 10.1155/2020/5239854 (PMC7204377; doi:10.1155/2020/5239854)
Supplement: Supplementary Materials — Figure S1: ADME/T properties of compounds of three TCM formulae. Table S1: herb composition and compounds of each formula retrieved from PDTCM. Table S2: psoriasis-related proteins. Table S3: compound-target binding energy lower than −12.28 kcal/mol. Table S4: GSEA results for three TCM formulae. [file 5239854.f1.zip › 5239854.f1/Supplementary Figures.docx]

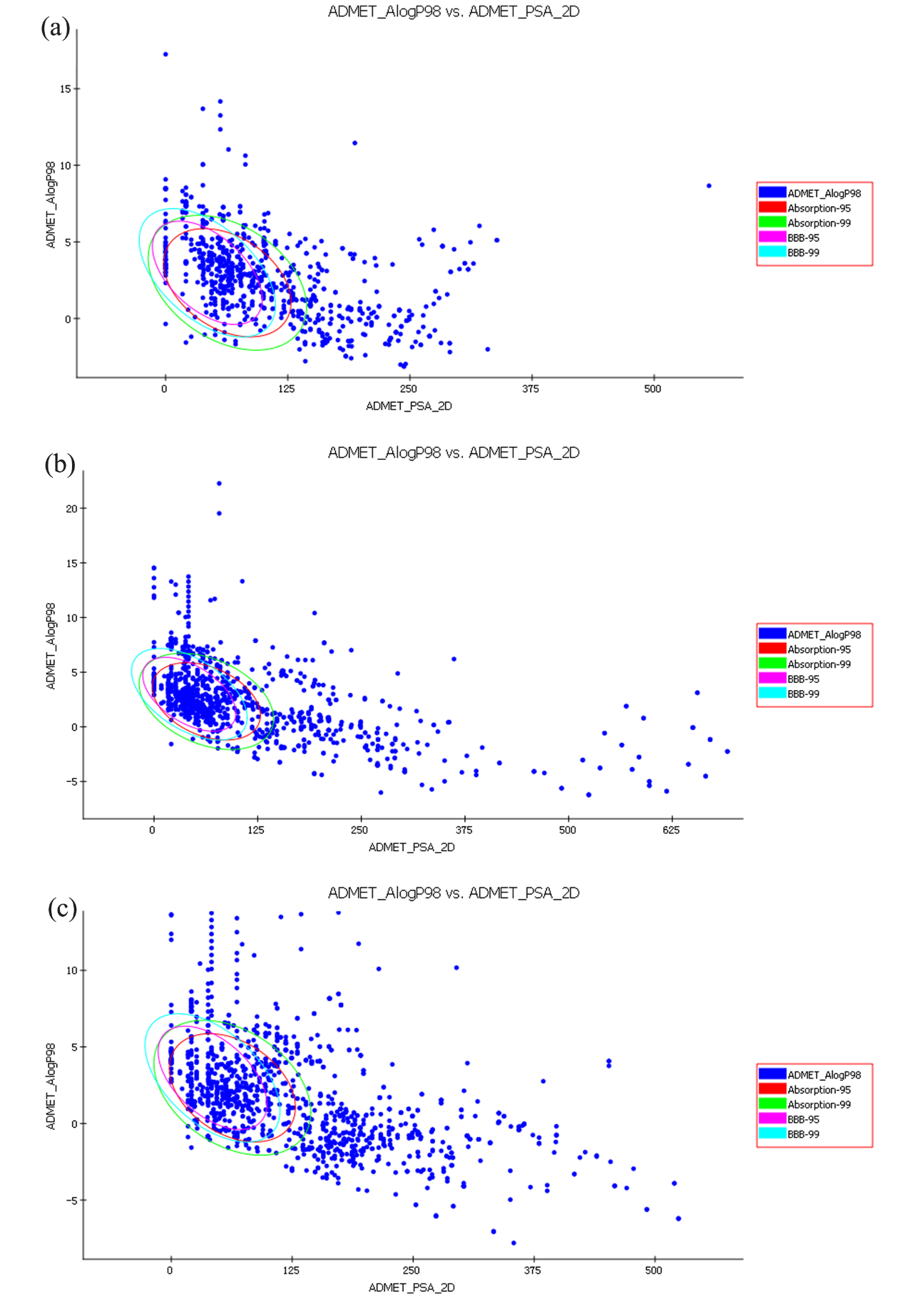


F_IGURE_ S1: ADME/T properties of compounds of three TCM formulae. The human intestinal absorption and blood-brain penetration (blood brain barrier, BBB) properties at the 95% or 99% confidence level against descriptors including AlogP98 and 2D polar surface area (PSA_2D) for compounds in F1 (a), F2 (b) and F3 (c), respectively.
