# Supplementary material for: Chinese Medicine for Psoriasis Vulgaris Based on Syndrome Pattern: A Network Pharmacological Study
Source: Evid Based Complement Alternat Med. 2020 Apr 28;2020:5239854. doi: 10.1155/2020/5239854 (PMC7204377; doi:10.1155/2020/5239854)
Supplement: Supplementary Materials — Figure S1: ADME/T properties of compounds of three TCM formulae. Table S1: herb composition and compounds of each formula retrieved from PDTCM. Table S2: psoriasis-related proteins. Table S3: compound-target binding energy lower than −12.28 kcal/mol. Table S4: GSEA results for three TCM formulae. [file 5239854.f1.zip › 5239854.f1/Table S1.docx]

**Table S1: Herb composition and compounds of each formula retrieved from PDTCM.**

| **Formula** | **Herb** | **PDTCM ID** | **Ref.** |
| --- | --- | --- | --- |
| F1 | *Angelica dahurica* | M000457 | (1) 阴健等. 中药现代研究与临床应用(1). 北京: 学苑出版社, 1993. (2) 江苏新医学院. 中药大辞典. 上海: 上海科学技术出版社, 1977. |
| F1 | *Angelica dahurica* | M000596 | Liu, Dongping; Kong, Guiping; Chen, Quan Cheng; Wang, Guanghui; Li, Jie; Xu, Yang; Lin, Ting; Tian, Yingpu; Zhang, Xiaokun; Yao, Xinsheng; Feng, Gensheng; Lu, Zhongxian; Chen, Haifeng; Bioorganic and Medicinal Chemistry Letters; vol. 21; 22; (2011); p. 6833 - 6837. |
| F1 | *Angelica dahurica* | M001018 | (1) 阴健等. 中药现代研究与临床应用(1). 北京: 学苑出版社, 1993. (2) 孙文基等. 天然活性成分简明手册. 北京: 中国医药科技出版社, 1998. (3) 国家中医药管理局《中华本草》编委会. 中华本草. Vol. 1-30 上海:上海科学技术出版社, 1999. (4) Hua AN, et al. 南京中医药大学学报, 1990, 6 (3): 179. |
| F1 | *Angelica dahurica* | M001442 | (1) 阴健等. 中药现代研究与临床应用(1). 北京: 学苑出版社, 1993. (2) 江苏新医学院. 中药大辞典. 上海: 上海科学技术出版社, 1977. (3) 饶高雄等. 中国中药杂志, 1993, 18 (12): 736. (4) 吉力等. 中国中药杂志, 1995, 20 (2): 120. (5) 孙文基等. 天然活性成分简明手册. 北京: 中国医药科技出版社, 1998. (6) 欧明等. 简明中药成分手册. 北京: 中国医药科技出版社, 2003. |
| F1 | *Angelica dahurica* | M002144 | 阴健等. 中药现代研究与临床应用(1). 北京: 学苑出版社, 1993 |
| F1 | *Angelica dahurica* | M002246 | Liu, Dongping; Kong, Guiping; Chen, Quan Cheng; Wang, Guanghui; Li, Jie; Xu, Yang; Lin, Ting; Tian, Yingpu; Zhang, Xiaokun; Yao, Xinsheng; Feng, Gensheng; Lu, Zhongxian; Chen, Haifeng; Bioorganic and Medicinal Chemistry Letters; vol. 21; 22; (2011); p. 6833 - 6837. |
| F1 | *Angelica dahurica* | M002282 | 阴健等. 中药现代研究与临床应用(1). 北京: 学苑出版社, 1993 |
| F1 | *Angelica dahurica* | M002557 | Liu, Dongping; Kong, Guiping; Chen, Quan Cheng; Wang, Guanghui; Li, Jie; Xu, Yang; Lin, Ting; Tian, Yingpu; Zhang, Xiaokun; Yao, Xinsheng; Feng, Gensheng; Lu, Zhongxian; Chen, Haifeng; Bioorganic and Medicinal Chemistry Letters; vol. 21; 22; (2011); p. 6833 - 6837. |
| F1 | *Angelica dahurica* | M003359 | Kwon, Yong-Soo; Kobayashi, Akio; Kajiyama, Shin-Ichiro; Kawazu, Kazuyoshi; Kanzaki, Hiroshi; Kim, Chang-Min; Phytochemistry; vol. 44; 5; (1997); p. 887 - 889. |
| F1 | *Angelica dahurica* | M003419 | Oh, Hyuncheol; Lee, Ho-Sub; Kim, Taewan; Chai, Kyu-Yun; Chung, Hun-Taeg; Kwon, Tae-Oh; Jun, Jung-Yang; Jeong, Ok-Sam; Kim, Youn-Chul; Yun, Young-Gab; Planta Medica; vol. 68; 5; (2002); p. 463 - 464. |
| F1 | *Angelica dahurica* | M003601 | 阴健等. 中药现代研究与临床应用(1). 北京: 学苑出版社, 1993 |
| F1 | *Angelica dahurica* | M003889 | (1) 阴健等. 中药现代研究与临床应用(1). 北京: 学苑出版社, 1993. (2) 季宇彬等. 中药抗肿瘤有效成分药理与应用. 哈尔滨: 黑龙江科学技术出版社, 1995. (3) 宋振玉等. 中草药现代研究. 第2卷. 第28章 九里香. 333-361 北京: 北京医科大学中国协和医科大学联合出版社, 1996. (4) 张印俊等. 药学学报, 1998, 33 (11): 836. (5) 饶高雄等. 中国中药杂志, 1996, 21 (8): 482. (6) 宋蔚等. 中国中药杂志, 1997, 22 (6): 359. (7) 王栋等. 中国中药杂志, 1997, 22 (8): 486. (8) 孙文基等. 天然�钚猿煞旨蛎魇植� 北京: 中国医药科技出版社, 1998. (9) 国家中医药管理局《中华本草》编委会. 中华本草. Vol. 1-30 上海:上海科学技术出版社, 1999. (10) El-Khrisy EAM, et al.Chem. Abstr., 1992, 117, 86802g. (11) Uno T, et al.Chem. Abstr., 1972, 76, 56567b. (12) Chaurasia N, et al.Chem. Abstr., 1986, 104, 85467w. (13) Khvorost PP, et al.Chem. Abstr., 1981, 94, 136170d. (14) Wu PL, et al. Chem Pharm Bull, 2005, 53 (1): 56. (15) Syu WJ, et al. JNP, 2001, 64 (9): 1232. (16) Yun BS, et al. JNP, 2001, 64 (9): 1238. (17) YUAN Z, et al. Chem Pharm Bull, 2002, 50 (1): 73. (18) MORIKAWA T, et al. Chem Pharm Bull, 2003, 51 (1): 62. (19) WANG N-H, et al. Chem Pharm Bull, 2003, 51 (1): 68. (20) WU T-S, et al. Chem [...truncated...] |
| F1 | *Angelica dahurica* | M003918 | Note; Qiao; Yao; Wang; Planta Medica; vol. 62; 6; (1996); p. 584 - 584. |
| F1 | *Angelica dahurica* | M004014 | 阴健等. 中药现代研究与临床应用(1). 北京: 学苑出版社, 1993 |
| F1 | *Angelica dahurica* | M004376 | 阴健等. 中药现代研究与临床应用(1). 北京: 学苑出版社, 1993 |
| F1 | *Angelica dahurica* | M004552 | Piao, Xiang Lan; Baek, Seung Hoon; Park, Man Ki; Park, Jeong Hill; Biological and Pharmaceutical Bulletin; vol. 27; 7; (2004); p. 1144 - 1146.; Chaya, Norihito; Terauchi, Kazuko; Yamagata, Yuriko; Kinjo, Junei; Okabe, Hikaru; Biological and Pharmaceutical Bulletin; vol. 27; 8; (2004); p. 1312 - 1316. |
| F1 | *Angelica dahurica* | M005661 | Liu, Dong-Ping; Luo, Qiang; Wang, Guang-Hui; Xu, Yang; Zhang, Xiao-Kun; Chen, Quan-Cheng; Chen, Hai-Feng; Molecules; vol. 16; 8; (2011); p. 6339 - 6348. |
| F1 | *Angelica dahurica* | M006192 | Kwon, Yong-Soo; Kobayashi, Akio; Kajiyama, Shin-Ichiro; Kawazu, Kazuyoshi; Kanzaki, Hiroshi; Kim, Chang-Min; Phytochemistry; vol. 44; 5; (1997); p. 887 - 889. |
| F1 | *Angelica dahurica* | M006455 | Liu, Dong-Ping; Luo, Qiang; Wang, Guang-Hui; Xu, Yang; Zhang, Xiao-Kun; Chen, Quan-Cheng; Chen, Hai-Feng; Molecules; vol. 16; 8; (2011); p. 6339 - 6348. |
| F1 | *Angelica dahurica* | M006850 | (1) 阴健等. 中药现代研究与临床应用(1). 北京: 学苑出版社, 1993. (2) 李其生等. 中国中药杂志, 1993, 18 (8): 486. (3) 肖永庆等. 中国中药杂志, 1995, 20 (5): 294. (4) 肖永庆等. 中国中药杂志, 1995, 20 (7): 423. (5) 饶高雄等. 中国中药杂志, 1995, 20 (12): 740. (6) 顾志平等. 中国中药杂志, 1997, 22 (1): 40. (7) 韦松等. 中国中药杂志, 1997, 22 (5): 293. (8) 宋蔚等. 中国中药杂志, 1997, 22 (6): 359. (9) 梁培瑜等. 中国中药杂志, 1998, 23 (1): 39. (10) 王海燕等. 中国中药杂志, 1998, 23 (3): 167. (11) 国家中医药管理局《中华本草》编委会. 中华本草. Vol. 1-30 上海:上海科学技术出版社, 1999. (12) Buckingham J(Executive Editor): et al. Dictionary of Natural Products, Vol 1-7, Chapman & Hall, London, 1994; 1995, Vol 8; 1996, Vol 9; 1997, Vol 10; 1998, Vol 11.. (13) Sautour M, et al. Chem Pharm Bull, 2004, 52 (10): 1235. (14) BEGUM S, et al. Chem Pharm Bull, 2003, 51 (2): 134. (15) Calixto JB, et al. Planta Med, 2003, 69 (11): 973. (16) Lin WY, et al. Planta Med, 2003, 69, 757. |
| F1 | *Angelica dahurica* | M006866 | Okuyama; Takata; Nishino; Takayasu; Iwashima; Chemical and Pharmaceutical Bulletin; vol. 38; 4; (1990); p. 1084 - 1086. |
| F1 | *Angelica dahurica* | M007369 | (1) 陈泽乃等. 中国中药杂志, 1996, 21 (7): 420. (2) 国家中医药管理局《中华本草》编委会. 中华本草. Vol. 1-30 上海:上海科学技术出版社, 1999. (3) 陈蕙芳等. 植物活性成分辞典. 第1册. 北京: 中国医药科技出版�� 2001. (4) Buckingham J(Executive Editor): et al. Dictionary of Natural Products, Vol 1-7, Chapman & Hall, London, 1994; 1995, Vol 8; 1996, Vol 9; 1997, Vol 10; 1998, Vol 11.. (5) Sautour M, et al. Chem Pharm Bull, 2004, 52 (10): 1235. (6) Fukuda T, et al. Phytochemistry, 2003, 63, 795. (7) ISHIKAWA T, et al. Chem Pharm Bull, 2001, 49 (5): 584. (8) YUAN Z, et al. Chem Pharm Bull, 2002, 50 (1): 73. (9) LI C-Y, et al. Chem Pharm Bull, 2002, 50 (10): 1305. (10) KITAJIMA J, et al. Chem Pharm Bull, 2003, 51 (6): 673. (11) Li CY, et al. JNP, 2002, 65 (10): 1452. (12) 欧明等. 简明中药成分手册. 北京: 中国医药科技出版社, 2003. |
| F1 | *Angelica dahurica* | M008239 | Liu, Dong-Ping; Luo, Qiang; Wang, Guang-Hui; Xu, Yang; Zhang, Xiao-Kun; Chen, Quan-Cheng; Chen, Hai-Feng; Molecules; vol. 16; 8; (2011); p. 6339 - 6348. |
| F1 | *Angelica dahurica* | M008578 | Retrieved from CNPD |
| F1 | *Angelica dahurica* | M008730 | Jia, Xiaodong; Zhao, Xingzeng; Wang, Ming; Chen, Yu; Dong, Yunfa; Feng, Xu; Chemistry of Natural Compounds; vol. 44; 6; (2008); p. 692 - 695. |
| F1 | *Angelica dahurica* | M008998 | 阴健等. 中药现代研究与临床应用(1). 北京: 学苑出版社, 1993 |
| F1 | *Angelica dahurica* | M009312 | Liu, Dong-Ping; Luo, Qiang; Wang, Guang-Hui; Xu, Yang; Zhang, Xiao-Kun; Chen, Quan-Cheng; Chen, Hai-Feng; Molecules; vol. 16; 8; (2011); p. 6339 - 6348. |
| F1 | *Angelica dahurica* | M009887 | 阴健等. 中药现代研究与临床应用(1). 北京: 学苑出版社, 1993 |
| F1 | *Angelica dahurica* | M009888 | 阴健等. 中药现代研究与临床应用(1). 北京: 学苑出版社, 1993 |
| F1 | *Angelica dahurica* | M009922 | (1) 季宇彬等. 中药抗肿瘤有效成分药理与应用. 哈尔滨: 黑龙江科学技术出版社, 1995. (2) 刘锁兰等. 药学学报, 1991, 26 (11): 836. (3) 张晓峰等. 药学学报, 1995, 30 (3): 211. (4) 刘桂芳等. 中国中药杂志, 1995, 20 (12): 738. (5) 饶高雄等. 中国中药杂志, 1996, 21 (8): 482. (6) 孙文基等. 天然活性成分简明手册. 北京: 中国医药科技出版社, 1998. (7) 国家中医药管理局《中华本草》编委会. 中华本草. Vol. 1-30 上海:上海�蒲Ъ际醭霭嫔� 1999. (8) Kang SY, et al. JNP, 2001, 64 (5): 683. (9) Patnam R, et al. JNP, 2001, 64 (7): 948. (10) MATSUDA H, et al. Chem Pharm Bull, 2005, 53 (4): 387. (11) CHIU C-Y, et al. Chem Pharm Bull, 2005, 53 (9): 1118. (12) Wu TS, et al. JNP, 2003, 66 (9): 1207. (13) Ito C, et al. Planta Med, 2005, 71 (1): 84. (14) 欧明等. 简明中药成分手册. 北京: 中国医药科技出版社, 2003. |
| F1 | *Angelica dahurica* | M010708 | Liu, Dongping; Kong, Guiping; Chen, Quan Cheng; Wang, Guanghui; Li, Jie; Xu, Yang; Lin, Ting; Tian, Yingpu; Zhang, Xiaokun; Yao, Xinsheng; Feng, Gensheng; Lu, Zhongxian; Chen, Haifeng; Bioorganic and Medicinal Chemistry Letters; vol. 21; 22; (2011); p. 6833 - 6837. |
| F1 | *Angelica dahurica* | M010952 | Liu, Dongping; Kong, Guiping; Chen, Quan Cheng; Wang, Guanghui; Li, Jie; Xu, Yang; Lin, Ting; Tian, Yingpu; Zhang, Xiaokun; Yao, Xinsheng; Feng, Gensheng; Lu, Zhongxian; Chen, Haifeng; Bioorganic and Medicinal Chemistry Letters; vol. 21; 22; (2011); p. 6833 - 6837. |
| F1 | *Angelica dahurica* | M011464 | (1) 阴健等. 中药现代研究与临床应用(1). 北京: 学苑出版社, 1993. (2) 国家中医药管理局《中华本草》编委会. 中华本草. Vol. 1-30 上海:上海科学技术出版社, 1999. |
| F1 | *Angelica dahurica* | M011753 | 阴健等. 中药现代研究与临床应用(1). 北京: 学苑出版社, 1993 |
| F1 | *Angelica dahurica* | M011949 | 阴健等. 中药现代研究与临床应用(1). 北京: 学苑出版社, 1993 |
| F1 | *Angelica dahurica* | M012570 | Liu, Dong-Ping; Luo, Qiang; Wang, Guang-Hui; Xu, Yang; Zhang, Xiao-Kun; Chen, Quan-Cheng; Chen, Hai-Feng; Molecules; vol. 16; 8; (2011); p. 6339 - 6348. |
| F1 | *Angelica dahurica* | M012580 | (1) 阴健等. 中药现代研究与临床应用(1). 北京: 学苑出版社, 1993. (2) 饶高雄等. 药学学报, 1991, 26 (1): 30. (3) 肖永庆等. 中国中药杂志, 1995, 20 (7): 423. (4) 汪纪武等. 植物药有效成分手册. 北京: 人民卫生出版社, 1986. (5) Buckingham J(Executive Editor): et al. Dictionary of Natural Products, Vol 1-7, Chapman & Hall, London, 1994; 1995, Vol 8; 1996, Vol 9; 1997, Vol 10; 1998, Vol 11.. (6) Kang SY, et al. JNP, 2001, 64 (5): 683. (7) Lee D, et al. JNP, 2001, 64 (10): 1286. (8) Ahua KM, et al. Phytochemistry, 2004, 65, 963. (9) Jiménez B, et al. Phytochemistry, 2000, 53, 1025. (10) Jang DS, et al. JNP, 2003, 66 (9): 1166. |
| F1 | *Angelica dahurica* | M013912 | 阴健等. 中药现代研究与临床应用(1). 北京: 学苑出版社, 1993 |
| F1 | *Angelica dahurica* | M014063 | WANG N-H, et al. Chem Pharm Bull, 2001,49 (9): 1085 |
| F1 | *Angelica dahurica* | M014583 | Note; Qiao; Yao; Wang; Planta Medica; vol. 62; 6; (1996); p. 584 - 584. |
| F1 | *Angelica dahurica* | M014609 | Kwon, Yong-Soo; Kobayashi, Akio; Kajiyama, Shin-Ichiro; Kawazu, Kazuyoshi; Kanzaki, Hiroshi; Kim, Chang-Min; Phytochemistry; vol. 44; 5; (1997); p. 887 - 889. |
| F1 | *Angelica dahurica* | M014815 | Hata et al.; Yakugaku Zasshi; vol. 83; (1963); p. 611,613; Chem.Abstr.; vol. 59; 7318; (1963). |
| F1 | *Angelica dahurica* | M015310 | 阴健等. 中药现代研究与临床应用(1). 北京: 学苑出版社, 1993 |
| F1 | *Angelica dahurica* | M015611 | 阴健等. 中药现代研究与临床应用(1). 北京: 学苑出版社, 1993 |
| F1 | *Angelica dahurica* | M015647 | Oh, Hyuncheol; Lee, Ho-Sub; Kim, Taewan; Chai, Kyu-Yun; Chung, Hun-Taeg; Kwon, Tae-Oh; Jun, Jung-Yang; Jeong, Ok-Sam; Kim, Youn-Chul; Yun, Young-Gab; Planta Medica; vol. 68; 5; (2002); p. 463 - 464. |
| F1 | *Angelica dahurica* | M016196 | Liu, Dongping; Kong, Guiping; Chen, Quan Cheng; Wang, Guanghui; Li, Jie; Xu, Yang; Lin, Ting; Tian, Yingpu; Zhang, Xiaokun; Yao, Xinsheng; Feng, Gensheng; Lu, Zhongxian; Chen, Haifeng; Bioorganic and Medicinal Chemistry Letters; vol. 21; 22; (2011); p. 6833 - 6837. |
| F1 | *Angelica dahurica* | M016205 | Liu, Dongping; Kong, Guiping; Chen, Quan Cheng; Wang, Guanghui; Li, Jie; Xu, Yang; Lin, Ting; Tian, Yingpu; Zhang, Xiaokun; Yao, Xinsheng; Feng, Gensheng; Lu, Zhongxian; Chen, Haifeng; Bioorganic and Medicinal Chemistry Letters; vol. 21; 22; (2011); p. 6833 - 6837. |
| F1 | *Angelica dahurica* | M016853 | Liu, Dong-Ping; Luo, Qiang; Wang, Guang-Hui; Xu, Yang; Zhang, Xiao-Kun; Chen, Quan-Cheng; Chen, Hai-Feng; Molecules; vol. 16; 8; (2011); p. 6339 - 6348. |
| F1 | *Angelica dahurica* | M016854 | Liu, Dong-Ping; Luo, Qiang; Wang, Guang-Hui; Xu, Yang; Zhang, Xiao-Kun; Chen, Quan-Cheng; Chen, Hai-Feng; Molecules; vol. 16; 8; (2011); p. 6339 - 6348. |
| F1 | *Angelica dahurica* | M016861 | Xie, Ying; Zhao, Weiquan; Zhou, Tingting; Fan, Guorong; Wu, Yutian; Phytochemical Analysis; vol. 21; 5; (2010); p. 473 - 482. |
| F1 | *Angelica dahurica* | M016999 | Liu, Dongping; Kong, Guiping; Chen, Quan Cheng; Wang, Guanghui; Li, Jie; Xu, Yang; Lin, Ting; Tian, Yingpu; Zhang, Xiaokun; Yao, Xinsheng; Feng, Gensheng; Lu, Zhongxian; Chen, Haifeng; Bioorganic and Medicinal Chemistry Letters; vol. 21; 22; (2011); p. 6833 - 6837. |
| F1 | *Angelica dahurica* | M017000 | Liu, Dongping; Kong, Guiping; Chen, Quan Cheng; Wang, Guanghui; Li, Jie; Xu, Yang; Lin, Ting; Tian, Yingpu; Zhang, Xiaokun; Yao, Xinsheng; Feng, Gensheng; Lu, Zhongxian; Chen, Haifeng; Bioorganic and Medicinal Chemistry Letters; vol. 21; 22; (2011); p. 6833 - 6837. |
| F1 | *Angelica dahurica* | M017600 | Liu, Dong-Ping; Luo, Qiang; Wang, Guang-Hui; Xu, Yang; Zhang, Xiao-Kun; Chen, Quan-Cheng; Chen, Hai-Feng; Molecules; vol. 16; 8; (2011); p. 6339 - 6348. |
| F1 | *Angelica dahurica* | M017625 | Liu, Dongping; Kong, Guiping; Chen, Quan Cheng; Wang, Guanghui; Li, Jie; Xu, Yang; Lin, Ting; Tian, Yingpu; Zhang, Xiaokun; Yao, Xinsheng; Feng, Gensheng; Lu, Zhongxian; Chen, Haifeng; Bioorganic and Medicinal Chemistry Letters; vol. 21; 22; (2011); p. 6833 - 6837. |
| F1 | *Angelica dahurica* | M019082 | Liu, Dongping; Kong, Guiping; Chen, Quan Cheng; Wang, Guanghui; Li, Jie; Xu, Yang; Lin, Ting; Tian, Yingpu; Zhang, Xiaokun; Yao, Xinsheng; Feng, Gensheng; Lu, Zhongxian; Chen, Haifeng; Bioorganic and Medicinal Chemistry Letters; vol. 21; 22; (2011); p. 6833 - 6837. |
| F1 | *Angelica dahurica* | M019273 | Kwon, Yong-Soo; Kobayashi, Akio; Kajiyama, Shin-Ichiro; Kawazu, Kazuyoshi; Kanzaki, Hiroshi; Kim, Chang-Min; Phytochemistry; vol. 44; 5; (1997); p. 887 - 889. |
| F1 | *Angelica dahurica* | M020637 | Li, Bin; Zhang, Xu; Wang, Juan; Zhang, Le; Gao, Bowen; Shi, Shepo; Wang, Xiaohui; Li, Jun; Tu, Pengfei; Phytochemical Analysis; vol. 25; 3; (2014); p. 229 - 240. |
| F1 | *Angelica dahurica* | M022162 | 阴健等. 中药现代研究与临床应用(1). 北京: 学苑出版社, 1993 |
| F1 | *Angelica dahurica* | M022266 | Kimura, Yoshiyuki; Okuda, Hiromichi; Baba, Kimiye; Journal of Natural Products; vol. 60; 3; (1997); p. 249 - 251. |
| F1 | *Angelica dahurica* | M023372 | 阴健等. 中药现代研究与临床应用(1). 北京: 学苑出版社, 1993 |
| F1 | *Angelica dahurica* | M023614 | (1) 季宇彬等. 中药抗肿瘤有效成分药理与应用. 哈尔滨: 黑龙江科学技术出版社, 1995. (2) 孙文基等. 天然活性成分简明手册. 北京: 中国医药科技出版社, 1998. |
| F1 | *Angelica dahurica* | M023623 | Li, Bin; Zhang, Xu; Wang, Juan; Zhang, Le; Gao, Bowen; Shi, Shepo; Wang, Xiaohui; Li, Jun; Tu, Pengfei; Phytochemical Analysis; vol. 25; 3; (2014); p. 229 - 240. |
| F1 | *Angelica dahurica* | M023624 | Oh, Hyuncheol; Lee, Ho-Sub; Kim, Taewan; Chai, Kyu-Yun; Chung, Hun-Taeg; Kwon, Tae-Oh; Jun, Jung-Yang; Jeong, Ok-Sam; Kim, Youn-Chul; Yun, Young-Gab; Planta Medica; vol. 68; 5; (2002); p. 463 - 464. |
| F1 | *Angelica dahurica* | M024904 | (1) 阴健等. 中药现代研究与临床应用(1). 北京: 学苑出版社, 1993. (2) 王曙等. 中国中药杂志, 1996, 21 (5): 295. (3) 国家中医药管理局《中华本草》编委会. 中华本草. Vol. 1-30 上海:上海科学技术出版社, 1999. (4) 陈蕙芳等. 植物活性成分辞典. 第1册. 北京: 中国医药科技出版社, 2001. (5) Kang SY, et al. JNP, 2001, 64 (5): 683. (6) 欧明等. 简明中药成分手册. 北京: 中国医药科技出版社, 2003. (7) 刈米达夫著, 杨本文译. 植物化学, 科学出版社, 北京, 1985. |
| F1 | *Angelica dahurica* | M025149 | Hata et al.; Yakugaku Zasshi; vol. 83; (1963); p. 611,613; Chem.Abstr.; vol. 59; 7318; (1963). |
| F1 | *Angelica dahurica* | M025409 | Jia, Xiaodong; Feng, Xu; Zhao, Xingzeng; Dong, Yunfa; Zhao, Youyi; Sun, Hao; Chemistry of Natural Compounds; vol. 44; 2; (2008); p. 166 - 168. |
| F1 | *Angelica dahurica* | M025468 | Jia, Xiaodong; Zhao, Xingzeng; Wang, Ming; Chen, Yu; Dong, Yunfa; Feng, Xu; Chemistry of Natural Compounds; vol. 44; 6; (2008); p. 692 - 695. |
| F1 | *Angelica dahurica* | M025924 | Chaya, Norihito; Terauchi, Kazuko; Yamagata, Yuriko; Kinjo, Junei; Okabe, Hikaru; Biological and Pharmaceutical Bulletin; vol. 27; 8; (2004); p. 1312 - 1316. |
| F1 | *Angelica dahurica* | M025995 | Hata et al.; Yakugaku Zasshi; vol. 83; (1963); p. 611,613; Chem.Abstr.; vol. 59; 7318; (1963). |
| F1 | *Angelica dahurica* | M026446 | Liu, Dong-Ping; Luo, Qiang; Wang, Guang-Hui; Xu, Yang; Zhang, Xiao-Kun; Chen, Quan-Cheng; Chen, Hai-Feng; Molecules; vol. 16; 8; (2011); p. 6339 - 6348. |
| F1 | *Angelica dahurica* | M027639 | Ban, Hyun Seung; Lim, Soon Sung; Suzuki, Katsuya; Jung, Sang Hoon; Lee, Sanghyun; Lee, Yeon Sil; Shin, Kuk Hyun; Ohuchi, Kazuo; Planta Medica; vol. 69; 5; (2003); p. 408 - 412. |
| F1 | *Angelica dahurica* | M027649 | WANG N-H, et al. Chem Pharm Bull, 2001,49 (9): 1085 |
| F1 | *Angelica dahurica* | M027754 | Hata et al.; Yakugaku Zasshi; vol. 83; (1963); p. 611,613; Chem.Abstr.; vol. 59; 7318; (1963). |
| F1 | *Angelica dahurica* | M027770 | 阴健等. 中药现代研究与临床应用(1). 北京: 学苑出版社, 1993 |
| F1 | *Angelica dahurica* | M027793 | Note; Qiao; Yao; Wang; Planta Medica; vol. 62; 6; (1996); p. 584 - 584. |
| F1 | *Angelica dahurica* | M027822 | Xie, Ying; Zhao, Weiquan; Zhou, Tingting; Fan, Guorong; Wu, Yutian; Phytochemical Analysis; vol. 21; 5; (2010); p. 473 - 482. |
| F1 | *Angelica dahurica* | M028027 | Lechner, Doris; Stavri, Michael; Oluwatuyi, Moyosoluwa; Pereda-Miranda, Rogelio; Gibbons, Simon; Phytochemistry; vol. 65; 3; (2004); p. 331 - 335. |
| F1 | *Angelica dahurica* | M028396 | (1) 阴健等. 中药现代研究与临床应用(1). 北京: 学苑出版社, 1993. (2) Carcache-Blanco EJ, et al. JNP, 2003, 67 (1): 126. |
| F1 | *Angelica dahurica* | M028401 | Jia, Xiaodong; Feng, Xu; Zhao, Xingzeng; Dong, Yunfa; Zhao, Youyi; Sun, Hao; Chemistry of Natural Compounds; vol. 44; 2; (2008); p. 166 - 168. |
| F1 | *Angelica dahurica* | M028776 | Liu, Dong-Ping; Luo, Qiang; Wang, Guang-Hui; Xu, Yang; Zhang, Xiao-Kun; Chen, Quan-Cheng; Chen, Hai-Feng; Molecules; vol. 16; 8; (2011); p. 6339 - 6348. |
| F1 | *Angelica dahurica* | M028779 | (1) 阴健等. 中药现代研究与临床应用(1). 北京: 学苑出版社, 1993. (2) Kang SY, et al. JNP, 2001, 64 (5): 683. (3) Ito C, et al. Planta Med, 2005, 71 (1): 84. |
| F1 | *Angelica dahurica* | M028841 | Hata et al.; Yakugaku Zasshi; vol. 83; (1963); p. 611,613; Chem.Abstr.; vol. 59; 7318; (1963). |
| F1 | *Angelica dahurica* | M030466 | WANG N-H, et al. Chem Pharm Bull, 2001,49 (9): 1085 |
| F1 | *Angelica dahurica* | M030835 | Liu, Dongping; Kong, Guiping; Chen, Quan Cheng; Wang, Guanghui; Li, Jie; Xu, Yang; Lin, Ting; Tian, Yingpu; Zhang, Xiaokun; Yao, Xinsheng; Feng, Gensheng; Lu, Zhongxian; Chen, Haifeng; Bioorganic and Medicinal Chemistry Letters; vol. 21; 22; (2011); p. 6833 - 6837. |
| F1 | *Angelica dahurica* | M031240 | Li, Bin; Zhang, Xu; Wang, Juan; Zhang, Le; Gao, Bowen; Shi, Shepo; Wang, Xiaohui; Li, Jun; Tu, Pengfei; Phytochemical Analysis; vol. 25; 3; (2014); p. 229 - 240. |
| F1 | *Angelica dahurica* | M031564 | Kuk hyun S, et al.Chem. Abstr., 1988, 109, 122447r |
| F1 | *Angelica dahurica* | M033133 | Liu, Dongping; Kong, Guiping; Chen, Quan Cheng; Wang, Guanghui; Li, Jie; Xu, Yang; Lin, Ting; Tian, Yingpu; Zhang, Xiaokun; Yao, Xinsheng; Feng, Gensheng; Lu, Zhongxian; Chen, Haifeng; Bioorganic and Medicinal Chemistry Letters; vol. 21; 22; (2011); p. 6833 - 6837. |
| F1 | *Angelica dahurica* | M033147 | WANG N-H, et al. Chem Pharm Bull, 2001,49 (9): 1085 |
| F1 | *Angelica dahurica* | M033650 | Zhao, Xingzeng; Feng, Xu; Jia, Xiaodong; Wang, Ming; Shan, Yu; Dong, Yunfa; Chemistry of Natural Compounds; vol. 43; 4; (2007); p. 399 - 401. |
| F1 | *Angelica dahurica* | M033744 | WANG N-H, et al. Chem Pharm Bull, 2001,49 (9): 1085 |
| F1 | *Angelica dahurica* | M033771 | 阴健等. 中药现代研究与临床应用(1). 北京: 学苑出版社, 1993 |
| F1 | *Angelica dahurica* | M034127 | Bergendorff, Ola; Dekermendjian, Kim; Nielsen, Mogens; Shan, Rudong; Witt, Robin; Ai, Jinglu; Sterner, Olov; Phytochemistry; vol. 44; 6; (1997); p. 1121 - 1124. |
| F1 | *Angelica dahurica* | M034213 | (1) 阴健等. 中药现代研究与临床应用(1). 北京: 学苑出版社, 1993. (2) 国家中医药管理局《中华本草》编委会. 中华本草. Vol. 1-30 上海:上海科学技术出版社, 1999. |
| F1 | *Arnebia euchroma* | M000127 | Yao; Ebizuka; Noguchi; Kiuchi; Shibuya; Iitaka; Seto; Sankawa; Chemical and Pharmaceutical Bulletin; vol. 39; 11; (1991); p. 2956 - 2961. |
| F1 | *Arnebia euchroma* | M000874 | (1) 江苏新医学院. 中药大辞典. 上海: 上海科学技术出版社, 1977. (2) 佟晓杰等. 中国药学杂志, 1993, 28 (3): 133. (3) 鲁学照等. 中国中药杂志, 1997, 22 (11): 680. (4) 黄浩等. 中国中药杂志, 1998, 23 (1): 37. (5) 甘露等. 中国中药杂志, 1998, 23 (6): 361. (6) 李强等. 中国中药杂志, 1998, 23 (9): 570. (7) 陈蕙芳等. 植物活性成分辞典. 第1册. 北京: 中国医药科技出版社, 2001. |
| F1 | *Arnebia euchroma* | M001391 | Shen, Chien-Chang; Syu, Wan-Jr; Li, Shyh-Yuan; Lin, Chia-Hung; Lee, Gum-Hee; Sun, Chang-Ming; Journal of Natural Products; vol. 65; 12; (2002); p. 1857 - 1862. |
| F1 | *Arnebia euchroma* | M001504 | 张慧祯等. 天然产物研究与开发, 2002, 14 (1): 74 |
| F1 | *Arnebia euchroma* | M002163 | Shen, Chien-Chang; Syu, Wan-Jr; Li, Shyh-Yuan; Lin, Chia-Hung; Lee, Gum-Hee; Sun, Chang-Ming; Journal of Natural Products; vol. 65; 12; (2002); p. 1857 - 1862. |
| F1 | *Arnebia euchroma* | M003921 | Wang, Feixin; US2005/222258; A1; (2005). |
| F1 | *Arnebia euchroma* | M004268 | Xin-Sheng, Yao; Ebizuka, Yutaka; Noguchi, Hiroshi; Kiuchi, Fumiyuki; Seto, Haruo; Sankawa, Ushio; Tetrahedron Letters; vol. 25; 48; (1984); p. 5541 - 5542. |
| F1 | *Arnebia euchroma* | M006767 | 张慧祯等. 天然产物研究与开发, 2002, 14 (1): 74 |
| F1 | *Arnebia euchroma* | M007580 | Shen, Chien-Chang; Syu, Wan-Jr; Li, Shyh-Yuan; Lin, Chia-Hung; Lee, Gum-Hee; Sun, Chang-Ming; Journal of Natural Products; vol. 65; 12; (2002); p. 1857 - 1862. |
| F1 | *Arnebia euchroma* | M008484 | 张慧祯等. 天然产物研究与开发, 2002, 14 (1): 1 |
| F1 | *Arnebia euchroma* | M008616 | Yao; Ebizuka; Noguchi; Kiuchi; Shibuya; Iitaka; Seto; Sankawa; Chemical and Pharmaceutical Bulletin; vol. 39; 11; (1991); p. 2956 - 2961. |
| F1 | *Arnebia euchroma* | M008803 | Shen, Chien-Chang; Syu, Wan-Jr; Li, Shyh-Yuan; Lin, Chia-Hung; Lee, Gum-Hee; Sun, Chang-Ming; Journal of Natural Products; vol. 65; 12; (2002); p. 1857 - 1862. |
| F1 | *Arnebia euchroma* | M008895 | Chang; Kuo; Weng; Jan; Ko; Teng; Planta Medica; vol. 59; 5; (1993); p. 401 - 404. |
| F1 | *Arnebia euchroma* | M009182 | Yao; Ebizuka; Noguchi; Kiuchi; Shibuya; Iitaka; Seto; Sankawa; Chemical and Pharmaceutical Bulletin; vol. 39; 11; (1991); p. 2962 - 2964. |
| F1 | *Arnebia euchroma* | M009523 | Shen, Chien-Chang; Syu, Wan-Jr; Li, Shyh-Yuan; Lin, Chia-Hung; Lee, Gum-Hee; Sun, Chang-Ming; Journal of Natural Products; vol. 65; 12; (2002); p. 1857 - 1862. |
| F1 | *Arnebia euchroma* | M009576 | Shen, Chien-Chang; Syu, Wan-Jr; Li, Shyh-Yuan; Lin, Chia-Hung; Lee, Gum-Hee; Sun, Chang-Ming; Journal of Natural Products; vol. 65; 12; (2002); p. 1857 - 1862. |
| F1 | *Arnebia euchroma* | M010079 | 张慧祯等. 天然产物研究与开发, 2002, 14 (1): 74 |
| F1 | *Arnebia euchroma* | M010620 | 张慧祯等. 天然产物研究与开发, 2002, 14 (1): 74 |
| F1 | *Arnebia euchroma* | M011323 | Chang; Kuo; Weng; Jan; Ko; Teng; Planta Medica; vol. 59; 5; (1993); p. 401 - 404. |
| F1 | *Arnebia euchroma* | M012218 | Shen, Chien-Chang; Syu, Wan-Jr; Li, Shyh-Yuan; Lin, Chia-Hung; Lee, Gum-Hee; Sun, Chang-Ming; Journal of Natural Products; vol. 65; 12; (2002); p. 1857 - 1862. |
| F1 | *Arnebia euchroma* | M012338 | Liu, Hong; Jin, Yong-Sheng; Song, Yan; Yang, Xiang-Nan; Yang, Xiao-Wei; Geng, Dong-Sheng; Chen, Hai-Sheng; Journal of Asian Natural Products Research; vol. 12; 4; (2010); p. 286 - 292. |
| F1 | *Arnebia euchroma* | M012848 | Wang, Feixin; US2005/222258; A1; (2005). |
| F1 | *Arnebia euchroma* | M013272 | Shen, Chien-Chang; Syu, Wan-Jr; Li, Shyh-Yuan; Lin, Chia-Hung; Lee, Gum-Hee; Sun, Chang-Ming; Journal of Natural Products; vol. 65; 12; (2002); p. 1857 - 1862. |
| F1 | *Arnebia euchroma* | M014393 | Chang; Kuo; Weng; Jan; Ko; Teng; Planta Medica; vol. 59; 5; (1993); p. 401 - 404. |
| F1 | *Arnebia euchroma* | M015278 | Wang, Feixin; US2005/222258; A1; (2005). |
| F1 | *Arnebia euchroma* | M015348 | Yao; Ebizuka; Noguchi; Kiuchi; Shibuya; Iitaka; Seto; Sankawa; Chemical and Pharmaceutical Bulletin; vol. 39; 11; (1991); p. 2956 - 2961. |
| F1 | *Arnebia euchroma* | M015524 | Xin-Sheng, Yao; Ebizuka, Yutaka; Noguchi, Hiroshi; Kiuchi, Fumiyuki; Sankawa, Ushio; Seto, Harou; Tetrahedron Letters; vol. 24; 31; (1983); p. 3247 - 3250. |
| F1 | *Arnebia euchroma* | M015738 | Liu, Hong; Jin, Yong-Sheng; Song, Yan; Yang, Xiang-Nan; Yang, Xiao-Wei; Geng, Dong-Sheng; Chen, Hai-Sheng; Journal of Asian Natural Products Research; vol. 12; 4; (2010); p. 286 - 292. |
| F1 | *Arnebia euchroma* | M021996 | 张慧祯等. 天然产物研究与开发, 2002, 14 (1): 1 |
| F1 | *Arnebia euchroma* | M022243 | 张慧祯等. 天然产物研究与开发, 2002, 14 (1): 74 |
| F1 | *Arnebia euchroma* | M022630 | 张慧祯等. 天然产物研究与开发, 2002, 14 (1): 74 |
| F1 | *Arnebia euchroma* | M023881 | Liu, Hong; Jin, Yong-Sheng; Song, Yan; Yang, Xiang-Nan; Yang, Xiao-Wei; Geng, Dong-Sheng; Chen, Hai-Sheng; Journal of Asian Natural Products Research; vol. 12; 4; (2010); p. 286 - 292. |
| F1 | *Arnebia euchroma* | M024215 | Shen, Chien-Chang; Syu, Wan-Jr; Li, Shyh-Yuan; Lin, Chia-Hung; Lee, Gum-Hee; Sun, Chang-Ming; Journal of Natural Products; vol. 65; 12; (2002); p. 1857 - 1862. |
| F1 | *Arnebia euchroma* | M024720 | 张慧祯等. 天然产物研究与开发, 2002, 14 (1): 74 |
| F1 | *Arnebia euchroma* | M026504 | 张慧祯等. 天然产物研究与开发, 2002, 14 (1): 74 |
| F1 | *Arnebia euchroma* | M030694 | 张慧祯等. 天然产物研究与开发, 2002, 14 (1): 74 |
| F1 | *Arnebia euchroma* | M031225 | Shen, Chien-Chang; Syu, Wan-Jr; Li, Shyh-Yuan; Lin, Chia-Hung; Lee, Gum-Hee; Sun, Chang-Ming; Journal of Natural Products; vol. 65; 12; (2002); p. 1857 - 1862. |
| F1 | *Arnebia euchroma* | M031979 | 张慧祯等. 天然产物研究与开发, 2002, 14 (1): 74 |
| F1 | *Arnebia euchroma* | M032120 | Wang, Feixin; US2005/222258; A1; (2005). |
| F1 | *Arnebia euchroma* | M032787 | Shen, Chien-Chang; Syu, Wan-Jr; Li, Shyh-Yuan; Lin, Chia-Hung; Lee, Gum-Hee; Sun, Chang-Ming; Journal of Natural Products; vol. 65; 12; (2002); p. 1857 - 1862. |
| F1 | *Arnebia euchroma* | M033769 | Shen, Chien-Chang; Syu, Wan-Jr; Li, Shyh-Yuan; Lin, Chia-Hung; Lee, Gum-Hee; Sun, Chang-Ming; Journal of Natural Products; vol. 65; 12; (2002); p. 1857 - 1862. |
| F1 | *Arnebia euchroma* | M034287 | Xin-Sheng, Yao; Ebizuka, Yutaka; Noguchi, Hiroshi; Kiuchi, Fumiyuki; Iitaka, Yoichi; Sankawa, Ushio; Tetrahedron Letters; vol. 24; 23; (1983); p. 2407 - 2410. |
| F1 | *Arnebia euchroma* | M034352 | Shen, Chien-Chang; Syu, Wan-Jr; Li, Shyh-Yuan; Lin, Chia-Hung; Lee, Gum-Hee; Sun, Chang-Ming; Journal of Natural Products; vol. 65; 12; (2002); p. 1857 - 1862. |
| F1 | *Baphicacanthus cusia* | M001362 | (1) 邹继纯等. 药学学报, 1985, 20 (1): 45. (2) 国家中医药管理局《中华本草》编委会. 中华本草. Vol. 1-30 上海:上海科学技术出版社, 1999. |
| F1 | *Baphicacanthus cusia* | M001859 | (1) 江苏新医学院. 中药大辞典. 上海: 上海科学技术出版社, 1977. (2) 王建华等. 中国药学杂志, 1994, 29 (5): 268. (3) 孙文基等. 天然活性成分简明手册. 北京: 中国医药科技出版社, 1998. (4) 国家中医药管理局《�谢静荨繁辔� 中华本草. Vol. 1-30 上海:上海科学技术出版社, 1999. (5) Wu TS, et al. Phytochemistry, 1999, 52, 901. (6) Chumkaew P, et al. Chem Pharm Bull, 2005, 53 (1): 95. (7) Tanaka T, et al. Chem Pharm Bull, 2004, 52 (10): 1242. (8) Mutai C, et al. Phytochemistry, 2004, 65, 1159. (9) Tanaka R, et al. Planta Med, 2004, 70, 1234. (10) TORIUMI Y, et al. Chem Pharm Bull, 2003, 51 (1): 89. (11) Calixto JB, et al. Planta Med, 2003, 69 (11): 973. (12) NAKANISHI T, et al. Chem Pharm Bull, 2005, 53 (2): 229. (13) KIEM PV, et al. Chem Pharm Bull, 2005, 53 (4): 428. (14) Gutierrez-Lugo M-T, et al. Planta Med, 2004, 70 (3): 263. (15) Madureira AM, et al. Planta Med, 2004, 70 (9): 828. (16) Puapairoj P, et al. Planta Med, 2005, 71 (3): 208. (17) Li XQ, et al. Planta Med, 2003, 69, 356. (18) Chaaib F, et al. Planta Med, 2003, 69, 316. |
| F1 | *Baphicacanthus cusia* | M006509 | (1) 阴健等. 中药现代研究与临床应用(1). 北京: 学苑出版社, 1993. (2) 孙文基等. 天然活性成分简明手册. 北京: 中国医药科技出版社, 1998. (3) 国家中医药管理局《中华本草》编委会. 中华本草. Vol. 1-30 上海:上海科学技术出版社, 1999. (4) Heinemann C, et al. Planta Med, 2004, 70 (5): 385. (5) Oberthür C, et al. Planta Med, 2004, 70 (7): 642. |
| F1 | *Baphicacanthus cusia* | M007555 | (1) 阴健等. 中药现代研究与临床应用(1). 北京: 学苑出版社, 1993. (2) 陈妙华等. 中国中药杂志, 1993, 18 (7): 424. (3) 金秀莲等. 中国中药杂志, 1994, 19 (11): 695. (4) 倪慕云等. 中国中药杂志, 1989, 14 (7): 41. (5) 孙文基等. 天然活性成分简明手册. 北京: 中国医药科技出版社, 1998. (6) 国家中医药管理局《中华本草》编委会. 中华本草. Vol. 1-30 上海:上海科学技术出版社, 1999. (7) Tanaka T, et al. Chem Pharm Bull, 2004, 52 (10): 1242. (8) Kanchanapoom T, et al. Chem Pharm Bull, 2004, 52 (8): 980. (9) Pinar ?ahin F, et al. Phytochemistry, 2004, 65, 2095. (10) HARPUT US, et al. Chem Pharm Bull, 2002, 50 (6): 869. (11) ONO M, et al. Chem Pharm Bull, 2005, 53 (9): 1175. (12) He ZD, et al. JNP, 2003, 66 (6): 851. (13) Lin L-C, et al. Planta Med, 2004, 70 (1): 50. (14) K?rm?z?bekmez H, et al. Planta Med, 2004, 70 (8): 711. (15) Pu XP, et al. Planta Med, 2003, 69, 65. (16) Martin-Nizard F, et al. Planta Med, 2003, 69, 207. (17) Budzianowska A, et al. Planta Med, 2004, 70 (9): 834. (18) Abougazar H, et al. Planta Med, 2003, 69, 814. (19) Boje K, et al. Planta Med, 2003, 69, 820. |
| F1 | *Baphicacanthus cusia* | M008101 | (1) 季宇彬等. 中药抗肿瘤有效成分药理与应用. 哈尔滨: 黑龙江科学技术出版社, 1995. (2) 孙文基等. 天然活性成分简明手册. 北京: 中国医药科技出版社, 1998. (3) 国家中医药管理局《中华本草》编委会. 中华本草. Vol. 1-30 上海:上海科学技术出版社, 1999. (4) 欧明等. 简明中药成分手册. 北京: 中国医药科技出版社, 2003. |
| F1 | *Baphicacanthus cusia* | M010268 | Tanaka T, et al. Chem Pharm Bull, 2004, 52 (10): 1242 |
| F1 | *Baphicacanthus cusia* | M013248 | (1) 国家中医药管理局《中华本草》编委会. 中华本草(精选本上下册). 上海: 上海科学技术出版社, 1998. (2) Leclerc S, et al. J. Biol. Chem., 2001, 276 (1): 251. |
| F1 | *Baphicacanthus cusia* | M013402 | Tanaka T, et al. Chem Pharm Bull, 2004, 52 (10): 1242 |
| F1 | *Baphicacanthus cusia* | M015729 | Tanaka T, et al. Chem Pharm Bull, 2004, 52 (10): 1242 |
| F1 | *Baphicacanthus cusia* | M026404 | Tanaka T, et al. Chem Pharm Bull, 2004, 52 (10): 1242 |
| F1 | *Baphicacanthus cusia* | M028269 | Tanaka T, et al. Chem Pharm Bull, 2004, 52 (10): 1242 |
| F1 | *Baphicacanthus cusia* | M031379 | (1) 李典鹏等. 药学学报, 1999, 34 (1): 43. (2) Tanaka T, et al. Chem Pharm Bull, 2004, 52 (10): 1242. (3) MIN B-S, et al. Chem Pharm Bull, 2003, 51 (11): 1322. |
| F1 | *Crataegus pinnatifida* | M001050 | (1) 阴健等. 中药现代研究与临床应用(1). 北京: 学苑出版社, 1993. (2) 季宇彬等. 中药抗肿瘤有效成分药理与应用. 哈尔滨: 黑龙江科学技术出版社, 1995. (3) 宋蔚等. 中国中药杂志, 1997, 22 (6): 359. (4) 凌云等. 中国中药杂志, 1998, 23 (4): 232. (5) 黄西峰. 中国中药杂志, 1997, 22 (4): 247. (6) 孙文基等. 天然活性成分简明手册. 北京: 中国医药科技出版社, 1998. (7) 国家中医药管理局《中华本草》编委会. 中华本草. Vol. 1-30 上海:上海科学技术出版社, 1999. (8) Kimura T, et al. Phytochemistry, 2004, 65, 423. (9) YUAN Z, et al. Chem Pharm Bull, 2002, 50 (1): 73. (10) KITAJIMA M, et al. Chem Pharm Bull, 2005, 53 (10): 1355. (11) Yoshikawa M, et al. JNP, 2002, 65 (8): 1151. (12) Hou CC, et al. JNP, 2003, 66 (5): 625. (13) Itoh A, et al. JNP, 2003, 66 (9): 1212. (14) 毛水春等. 中国药物化学杂志, 2004, 14 (6): 326. (15) K?rm?z?bekmez H, et al. Planta Med, 2004, 70 (8): 711. (16) Zidorn C, et al. Phytochemistry, 2005, 66, 1691. (17) Chaubal R, et al. Planta Med, 2003, 69, 287. (18) 欧明等. 简明中药成分手册. 北京: 中国医药科技出版社, 2003. |
| F1 | *Crataegus pinnatifida* | M001968 | Min, Byung Sun; Jung, Hyun Ju; Lee, Jun Sung; Kim, Young Ho; Bok, Song Hae; Ma, Chao Mei; Nakamura, Norio; Hattori, Masao; Bae, KiHwan; Planta Medica; vol. 65; 4; (1999); p. 374 - 375. |
| F1 | *Crataegus pinnatifida* | M002050 | Bykov et al.; Chemistry of Natural Compounds; vol. 8; (1972); p. 694; Khimiya Prirodnykh Soedinenii; vol. 8; (1972); p. 709,715. |
| F1 | *Crataegus pinnatifida* | M002504 | Min, Byung Sun; Jung, Hyun Ju; Lee, Jun Sung; Kim, Young Ho; Bok, Song Hae; Ma, Chao Mei; Nakamura, Norio; Hattori, Masao; Bae, KiHwan; Planta Medica; vol. 65; 4; (1999); p. 374 - 375. |
| F1 | *Crataegus pinnatifida* | M003009 | (1) 江苏新医学院. 中药大辞典. 上海: 上海科学技术出版社, 1977. (2) 王菊英等. 中国药学杂志, 1996, 31 (5): 266. (3) 白银娟等. 中国药学杂志, 1997, 32 (8): 462. (4) 胡幼华等. 中国中药杂志, 1994, 19 (3): 164. (5) 杜海燕等. 中国中药杂志, 1998, 23 (11): 682. (6) 国家中医药管理局《中华本草》编委会. 中华本草. Vol. 1-30 上海:上海科学技术出版社, 1999. (7) Kundakovic T, et al. Chem Pharm Bull, 2004, 52 (12): 1462. (8) Block S, et al. Phytochemistry, 2004, 65, 1165. (9) TORIUMI Y, et al. Chem Pharm Bull, 2003, 51 (1): 89. (10) Zheng Y, et al. JNP, 2004, 67 (9): 1617. (11) Gutierrez-Lugo M-T, et al. Planta Med, 2004, 70 (3): 263. |
| F1 | *Crataegus pinnatifida* | M003449 | (1) 汪纪武等. 植物药有效成分手册. 北京: 人民卫生出版社, 1986. (2) 阴健等. 中药现代研究与临床应用(1). 北京: 学苑出版社, 1993. (3) 季宇彬等. 中药抗肿瘤有效成分药理与应用. 哈尔滨: 黑龙江科学技术出版社, 1995. (4) 赵浩如等. 中国中药杂志, 1993, 18 (4): 226. (5) 李更生等. 中国中药杂志, 1997, 22 (9): 548. (6) 凌云等. 中国中药杂志, 1998, 23 (4): 232. (7) 徐丽萍等. 中国中药杂志, 1998, 23 (5): 293. (8) 国家中医药管理局《中华本草》编委会. 中华本草. Vol. 1-30 上海:上海科学技术出版社, 1999. (9) Wu PL, et al. Chem Pharm Bull, 2005, 53 (1): 56. (10) Kim SR, et al. Phytochemistry, 2000, 54, 503. (11) YUAN Z, et al. Chem Pharm Bull, 2002, 50 (1): 73. (12) EL-SEEDI H-R, et al. Chem Pharm Bull, 2003, 51 (12): 1439. (13) CHIU C-Y, et al. Chem Pharm Bull, 2005, 53 (9): 1118. (14) Mo S, et al. JNP, 2004, 67 (5): 823. (15) Heitzman ME, et al. Phytochemistry, 2005, 66, 5. (16) 欧明等. 简明中药成分手册. 北京: 中国医药科技出版社, 2003. (17) Kimura T, et al. Phytochemistry, 2004, 65, 423. (18) Zidorn C, et al. Phytochemistry, 2005, 66, 1691. (19) Banskota AH, et al. Planta Med, 2003, 69, 500. (20) Boje K, et al. Planta Med, 2003, 69, 820. |
| F1 | *Crataegus pinnatifida* | M003470 | Ye, Xiao-L.I.; Huang, Wen-Wen; Chen, Zhu; Li, Xue-Gang; Li, Ping; Lan, Ping; Wang, Liang; Gao, Ying; Zhao, Zhong-Q.I.; Chen, Xin; Journal of Agricultural and Food Chemistry; vol. 58; 5; (2010); p. 3132 - 3138. |
| F1 | *Crataegus pinnatifida* | M003835 | (1) 阴健等. 中药现代研究与临床应用(1). 北京: 学苑出版社, 1993. (2) 江苏新医学院. 中药大辞典. 上海: 上海科学技术出版社, 1977. (3) 孙文基等. 天然活性成分简明手册. 北京: 中国医药科技出版社, 1998. (4) 国�抑幸揭┕芾砭帧吨谢静荨繁辔� 中华本草. Vol. 1-30 上海:上海科学技术出版社, 1999. |
| F1 | *Crataegus pinnatifida* | M004428 | Ye, Xiao-L.I.; Huang, Wen-Wen; Chen, Zhu; Li, Xue-Gang; Li, Ping; Lan, Ping; Wang, Liang; Gao, Ying; Zhao, Zhong-Q.I.; Chen, Xin; Journal of Agricultural and Food Chemistry; vol. 58; 5; (2010); p. 3132 - 3138. |
| F1 | *Crataegus pinnatifida* | M004870 | Zhou, Chen-Chen; Huang, Xiao-Xiao; Gao, Pin-Yi; Li, Fei-Fei; Li, Dian-Ming; Li, Ling-Zhi; Song, Shao-Jiang; Journal of Asian Natural Products Research; vol. 16; 2; (2014); p. 169 - 174. |
| F1 | *Crataegus pinnatifida* | M005023 | 阴健等. 中药现代研究与临床应用(1). 北京: 学苑出版社, 1993 |
| F1 | *Crataegus pinnatifida* | M005246 | Bykov; Glyzin; Chemistry of Natural Compounds; vol. 8; (1972); p. 657; Khimiya Prirodnykh Soedinenii; vol. 8; (1972); p. 672. |
| F1 | *Crataegus pinnatifida* | M005991 | Zhang, Pei-Cheng; Xu, Sui-Xu; Phytochemistry; vol. 57; 8; (2001); p. 1249 - 1253. |
| F1 | *Crataegus pinnatifida* | M006380 | 阴健等. 中药现代研究与临床应用(1). 北京: 学苑出版社, 1993 |
| F1 | *Crataegus pinnatifida* | M006584 | Zhang, Pei-Cheng; Xu, Sui-Xu; Phytochemistry; vol. 57; 8; (2001); p. 1249 - 1253. |
| F1 | *Crataegus pinnatifida* | M006919 | (1) 阴健等. 中药现代研究与临床应用(1). 北京: 学苑出版社, 1993. (2) 季宇彬等. 中药抗肿瘤有效成分药理与应用. 哈尔滨: 黑龙江科学技术出版社, 1995. (3) 李彤梅等. 药学学报, 1998, 33 (8): 591. (4) 傅宏征等. 中国药学杂志, 1998, 33 (3): 140. (5) 周燕生等. 中国中药杂志, 1994, 19 (3): 162. (6) 徐丽珍等. 中国中药杂志, 1994, 19 (11): 675. (7) 陈妙华等. 中国中药杂志, 1993, 18 (7): 424. (8) 石磊等. 中国中药杂志, 1997, 22 (12): 743. (9) 徐丽萍等. 中国中药杂志, 1998, 23 (5): 293. (10) 孙文基等. 天然活性成分简明手册. 北京: 中国医药科技出版社, 1998. (11) 国家中医药管理局《中华本草》编委会. 中华本草. Vol. 1-30 上海:上海科学技术出版社, 1999. (12) Zheng Y, et al. JNP, 2004, 67 (9): 1617. (13) 欧明等. 简明中药成分手册. 北京: 中国医药科技出版社, 2003. |
| F1 | *Crataegus pinnatifida* | M006965 | 阴健等. 中药现代研究与临床应用(1). 北京: 学苑出版社, 1993 |
| F1 | *Crataegus pinnatifida* | M007009 | 阴健等. 中药现代研究与临床应用(1). 北京: 学苑出版社, 1993 |
| F1 | *Crataegus pinnatifida* | M007437 | (1) 阴健等. 中药现代研究与临床应用(1). 北京: 学苑出版社, 1993. (2) 孙文基等. 天然活性成分简明手册. 北京: 中国医药科技出版社, 1998. (3) 国家中医药管理局《中华本草》编委会. 中华本草. Vol. 1-30 上海:上海科学技术出版社, 1999. |
| F1 | *Crataegus pinnatifida* | M007984 | Song, Shao-Jiang; Li, Ling-Zhi; Gao, Pin-Yi; Peng, Ying; Yang, Jing-Yu; Wu, Chun-Fu; Food Chemistry; vol. 129; 3; (2011); p. 933 - 939. |
| F1 | *Crataegus pinnatifida* | M008061 | Song, Shao-Jiang; Li, Ling-Zhi; Gao, Pin-Yi; Peng, Ying; Yang, Jing-Yu; Wu, Chun-Fu; Food Chemistry; vol. 129; 3; (2011); p. 933 - 939. |
| F1 | *Crataegus pinnatifida* | M008157 | 阴健等. 中药现代研究与临床应用(1). 北京: 学苑出版社, 1993 |
| F1 | *Crataegus pinnatifida* | M008269 | Ye, Xiao-L.I.; Huang, Wen-Wen; Chen, Zhu; Li, Xue-Gang; Li, Ping; Lan, Ping; Wang, Liang; Gao, Ying; Zhao, Zhong-Q.I.; Chen, Xin; Journal of Agricultural and Food Chemistry; vol. 58; 5; (2010); p. 3132 - 3138. |
| F1 | *Crataegus pinnatifida* | M008986 | (1) 阴健等. 中药现代研究与临床应用(1). 北京: 学苑出版社, 1993. (2) 饶高雄等. 药学学报, 1991, 26 (1): 30. (3) 杨峻山等. 药学学报, 1993, 28 (3): 197. (4) 赵余庆等. 中国中药杂志, 1993, 18 (7): 428. (5) �母咝鄣� 中国中药杂志, 1993, 18 (12): 736. (6) 饶高雄等. 中国中药杂志, 1995, 20 (12): 740. (7) 国家中医药管理局《中华本草》编委会. 中华本草. Vol. 1-30 上海:上海科学技术出版社, 1999. (8) TORIUMI Y, et al. Chem Pharm Bull, 2003, 51 (1): 89. (9) Carcache-Blanco EJ, et al. JNP, 2003, 67 (1): 126. |
| F1 | *Crataegus pinnatifida* | M009845 | (1) 阴健等. 中药现代研究与临床应用(1). 北京: 学苑出版社, 1993. (2) Buckingham J(Executive Editor): et al. Dictionary of Natural Products, Vol 1-7, Chapman & Hall, London, 1994; 1995, Vol 8; 1996, Vol 9; 1997, Vol 10; 1998, Vol 11.. |
| F1 | *Crataegus pinnatifida* | M010532 | Huang, Xiao-Xiao; Zhou, Chen-Chen; Li, Ling-Zhi; Li, Fei-Fei; Lou, Li-Li; Li, Dian-Ming; Ikejima, Takshi; Peng, Ying; Song, Shao-Jiang; Bioorganic and Medicinal Chemistry Letters; vol. 23; 20; (2013); p. 5599 - 5604. |
| F1 | *Crataegus pinnatifida* | M011679 | 阴健等. 中药现代研究与临床应用(1). 北京: 学苑出版社, 1993 |
| F1 | *Crataegus pinnatifida* | M012861 | (1) 江苏新医学院. 中药大辞典. 上海: 上海科学技术出版社, 1977. (2) 国家中医药管理局《中华本草》编委会. 中华本草. Vol. 1-30 上海:上海科学技术出版社, 1999. |
| F1 | *Crataegus pinnatifida* | M012949 | Song, Shao-Jiang; Li, Ling-Zhi; Gao, Pin-Yi; Peng, Ying; Yang, Jing-Yu; Wu, Chun-Fu; Food Chemistry; vol. 129; 3; (2011); p. 933 - 939. |
| F1 | *Crataegus pinnatifida* | M013188 | 阴健等. 中药现代研究与临床应用(1). 北京: 学苑出版社, 1993 |
| F1 | *Crataegus pinnatifida* | M013303 | 阴健等. 中药现代研究与临床应用(1). 北京: 学苑出版社, 1993 |
| F1 | *Crataegus pinnatifida* | M013324 | Retrieved from CNPD |
| F1 | *Crataegus pinnatifida* | M013971 | 汪纪武等. 植物药有效成分手册. 北京: 人民卫生出版社, 1986 |
| F1 | *Crataegus pinnatifida* | M014226 | 阴健等. 中药现代研究与临床应用(1). 北京: 学苑出版社, 1993 |
| F1 | *Crataegus pinnatifida* | M014516 | Moon, Hyung-In; Kim, Tae-im; Cho, Hyun-Soo; Kim, Eung Kweon; Bioorganic and Medicinal Chemistry Letters; vol. 20; 3; (2010); p. 991 - 993. |
| F1 | *Crataegus pinnatifida* | M015739 | Zhang, Pei-Cheng; Xu, Sui-Xu; Phytochemistry; vol. 57; 8; (2001); p. 1249 - 1253. |
| F1 | *Crataegus pinnatifida* | M015801 | 阴健等. 中药现代研究与临床应用(1). 北京: 学苑出版社, 1993 |
| F1 | *Crataegus pinnatifida* | M015911 | 阴健等. 中药现代研究与临床应用(1). 北京: 学苑出版社, 1993 |
| F1 | *Crataegus pinnatifida* | M016191 | Song, Shao-Jiang; Li, Ling-Zhi; Gao, Pin-Yi; Peng, Ying; Yang, Jing-Yu; Wu, Chun-Fu; Food Chemistry; vol. 129; 3; (2011); p. 933 - 939. |
| F1 | *Crataegus pinnatifida* | M017555 | Song, Shao-Jiang; Li, Ling-Zhi; Gao, Pin-Yi; Peng, Ying; Yang, Jing-Yu; Wu, Chun-Fu; Food Chemistry; vol. 129; 3; (2011); p. 933 - 939. |
| F1 | *Crataegus pinnatifida* | M017556 | Song, Shao-Jiang; Li, Ling-Zhi; Gao, Pin-Yi; Peng, Ying; Yang, Jing-Yu; Wu, Chun-Fu; Food Chemistry; vol. 129; 3; (2011); p. 933 - 939. |
| F1 | *Crataegus pinnatifida* | M017557 | Song, Shao-Jiang; Li, Ling-Zhi; Gao, Pin-Yi; Peng, Ying; Yang, Jing-Yu; Wu, Chun-Fu; Food Chemistry; vol. 129; 3; (2011); p. 933 - 939. |
| F1 | *Crataegus pinnatifida* | M017558 | Song, Shao-Jiang; Li, Ling-Zhi; Gao, Pin-Yi; Peng, Ying; Yang, Jing-Yu; Wu, Chun-Fu; Food Chemistry; vol. 129; 3; (2011); p. 933 - 939. |
| F1 | *Crataegus pinnatifida* | M017559 | Song, Shao-Jiang; Li, Ling-Zhi; Gao, Pin-Yi; Peng, Ying; Yang, Jing-Yu; Wu, Chun-Fu; Food Chemistry; vol. 129; 3; (2011); p. 933 - 939. |
| F1 | *Crataegus pinnatifida* | M017560 | Song, Shao-Jiang; Li, Ling-Zhi; Gao, Pin-Yi; Peng, Ying; Yang, Jing-Yu; Wu, Chun-Fu; Food Chemistry; vol. 129; 3; (2011); p. 933 - 939. |
| F1 | *Crataegus pinnatifida* | M017561 | Song, Shao-Jiang; Li, Ling-Zhi; Gao, Pin-Yi; Peng, Ying; Yang, Jing-Yu; Wu, Chun-Fu; Food Chemistry; vol. 129; 3; (2011); p. 933 - 939. |
| F1 | *Crataegus pinnatifida* | M017634 | Huang, Xiao-Xiao; Zhou, Chen-Chen; Li, Ling-Zhi; Li, Fei-Fei; Lou, Li-Li; Li, Dian-Ming; Ikejima, Takshi; Peng, Ying; Song, Shao-Jiang; Bioorganic and Medicinal Chemistry Letters; vol. 23; 20; (2013); p. 5599 - 5604. |
| F1 | *Crataegus pinnatifida* | M017985 | Huang, Xiao-Xiao; Zhou, Chen-Chen; Li, Ling-Zhi; Li, Fei-Fei; Lou, Li-Li; Li, Dian-Ming; Ikejima, Takshi; Peng, Ying; Song, Shao-Jiang; Bioorganic and Medicinal Chemistry Letters; vol. 23; 20; (2013); p. 5599 - 5604. |
| F1 | *Crataegus pinnatifida* | M017986 | Huang, Xiao-Xiao; Zhou, Chen-Chen; Li, Ling-Zhi; Li, Fei-Fei; Lou, Li-Li; Li, Dian-Ming; Ikejima, Takshi; Peng, Ying; Song, Shao-Jiang; Bioorganic and Medicinal Chemistry Letters; vol. 23; 20; (2013); p. 5599 - 5604. |
| F1 | *Crataegus pinnatifida* | M018876 | Huang, Xiao-Xiao; Zhou, Chen-Chen; Li, Ling-Zhi; Li, Fei-Fei; Lou, Li-Li; Li, Dian-Ming; Ikejima, Takshi; Peng, Ying; Song, Shao-Jiang; Bioorganic and Medicinal Chemistry Letters; vol. 23; 20; (2013); p. 5599 - 5604. |
| F1 | *Crataegus pinnatifida* | M018877 | Huang, Xiao-Xiao; Zhou, Chen-Chen; Li, Ling-Zhi; Li, Fei-Fei; Lou, Li-Li; Li, Dian-Ming; Ikejima, Takshi; Peng, Ying; Song, Shao-Jiang; Bioorganic and Medicinal Chemistry Letters; vol. 23; 20; (2013); p. 5599 - 5604. |
| F1 | *Crataegus pinnatifida* | M019285 | Huang, Xiao-Xiao; Zhou, Chen-Chen; Li, Ling-Zhi; Li, Fei-Fei; Lou, Li-Li; Li, Dian-Ming; Ikejima, Takshi; Peng, Ying; Song, Shao-Jiang; Bioorganic and Medicinal Chemistry Letters; vol. 23; 20; (2013); p. 5599 - 5604. |
| F1 | *Crataegus pinnatifida* | M019793 | Zhou, Chen-Chen; Huang, Xiao-Xiao; Gao, Pin-Yi; Li, Fei-Fei; Li, Dian-Ming; Li, Ling-Zhi; Song, Shao-Jiang; Journal of Asian Natural Products Research; vol. 16; 2; (2014); p. 169 - 174. |
| F1 | *Crataegus pinnatifida* | M019848 | Huang, Xiao-Xiao; Zhou, Chen-Chen; Li, Ling-Zhi; Li, Fei-Fei; Lou, Li-Li; Li, Dian-Ming; Ikejima, Takshi; Peng, Ying; Song, Shao-Jiang; Bioorganic and Medicinal Chemistry Letters; vol. 23; 20; (2013); p. 5599 - 5604. |
| F1 | *Crataegus pinnatifida* | M020119 | Huang, Xiao-Xiao; Zhou, Chen-Chen; Li, Ling-Zhi; Li, Fei-Fei; Lou, Li-Li; Li, Dian-Ming; Ikejima, Takshi; Peng, Ying; Song, Shao-Jiang; Bioorganic and Medicinal Chemistry Letters; vol. 23; 20; (2013); p. 5599 - 5604. |
| F1 | *Crataegus pinnatifida* | M020196 | Zhou, Chen-Chen; Huang, Xiao-Xiao; Gao, Pin-Yi; Li, Fei-Fei; Li, Dian-Ming; Li, Ling-Zhi; Song, Shao-Jiang; Journal of Asian Natural Products Research; vol. 16; 2; (2014); p. 169 - 174. |
| F1 | *Crataegus pinnatifida* | M020692 | Ye, Xiao-L.I.; Huang, Wen-Wen; Chen, Zhu; Li, Xue-Gang; Li, Ping; Lan, Ping; Wang, Liang; Gao, Ying; Zhao, Zhong-Q.I.; Chen, Xin; Journal of Agricultural and Food Chemistry; vol. 58; 5; (2010); p. 3132 - 3138. |
| F1 | *Crataegus pinnatifida* | M020937 | Huang, Xiao-Xiao; Zhou, Chen-Chen; Li, Ling-Zhi; Li, Fei-Fei; Lou, Li-Li; Li, Dian-Ming; Ikejima, Takshi; Peng, Ying; Song, Shao-Jiang; Bioorganic and Medicinal Chemistry Letters; vol. 23; 20; (2013); p. 5599 - 5604. |
| F1 | *Crataegus pinnatifida* | M020938 | Huang, Xiao-Xiao; Zhou, Chen-Chen; Li, Ling-Zhi; Li, Fei-Fei; Lou, Li-Li; Li, Dian-Ming; Ikejima, Takshi; Peng, Ying; Song, Shao-Jiang; Bioorganic and Medicinal Chemistry Letters; vol. 23; 20; (2013); p. 5599 - 5604. |
| F1 | *Crataegus pinnatifida* | M021216 | Liu, Xun; Wang, Dong; Zhang, Wenjie; Wang, Nan; Wang, Siyuan; Li, Haibo; Ying, Xixiang; Kang, Tingguo; Natural Product Research; vol. 26; 10; (2012); p. 962 - 967. |
| F1 | *Crataegus pinnatifida* | M021387 | Huang, Xiao-Xiao; Zhou, Chen-Chen; Li, Ling-Zhi; Li, Fei-Fei; Lou, Li-Li; Li, Dian-Ming; Ikejima, Takshi; Peng, Ying; Song, Shao-Jiang; Bioorganic and Medicinal Chemistry Letters; vol. 23; 20; (2013); p. 5599 - 5604. |
| F1 | *Crataegus pinnatifida* | M021388 | Huang, Xiao-Xiao; Zhou, Chen-Chen; Li, Ling-Zhi; Li, Fei-Fei; Lou, Li-Li; Li, Dian-Ming; Ikejima, Takshi; Peng, Ying; Song, Shao-Jiang; Bioorganic and Medicinal Chemistry Letters; vol. 23; 20; (2013); p. 5599 - 5604. |
| F1 | *Crataegus pinnatifida* | M021791 | Jeong, Tae-Sook; Hwang, Eui-Ll; Lee, Hyang-Bok; Lee, Eun-Sook; Kim, Young-Kook; Min, Byung-Sun; Bae, Ki-Hwan; Bok, Song-Hae; Kim, Sung-Uk; Planta Medica; vol. 65; 3; (1999); p. 261 - 263. |
| F1 | *Crataegus pinnatifida* | M021824 | Huang, Xiao-Xiao; Zhou, Chen-Chen; Li, Ling-Zhi; Li, Fei-Fei; Lou, Li-Li; Li, Dian-Ming; Ikejima, Takshi; Peng, Ying; Song, Shao-Jiang; Bioorganic and Medicinal Chemistry Letters; vol. 23; 20; (2013); p. 5599 - 5604. |
| F1 | *Crataegus pinnatifida* | M021825 | Huang, Xiao-Xiao; Zhou, Chen-Chen; Li, Ling-Zhi; Li, Fei-Fei; Lou, Li-Li; Li, Dian-Ming; Ikejima, Takshi; Peng, Ying; Song, Shao-Jiang; Bioorganic and Medicinal Chemistry Letters; vol. 23; 20; (2013); p. 5599 - 5604. |
| F1 | *Crataegus pinnatifida* | M022638 | 阴健等. 中药现代研究与临床应用(1). 北京: 学苑出版社, 1993 |
| F1 | *Crataegus pinnatifida* | M022772 | 阴健等. 中药现代研究与临床应用(1). 北京: 学苑出版社, 1993 |
| F1 | *Crataegus pinnatifida* | M023138 | Bykov; Glyzin; Chemistry of Natural Compounds; vol. 8; (1972); p. 657; Khimiya Prirodnykh Soedinenii; vol. 8; (1972); p. 672. |
| F1 | *Crataegus pinnatifida* | M023429 | 阴健等. 中药现代研究与临床应用(1). 北京: 学苑出版社, 1993 |
| F1 | *Crataegus pinnatifida* | M023851 | Ye, Xiao-L.I.; Huang, Wen-Wen; Chen, Zhu; Li, Xue-Gang; Li, Ping; Lan, Ping; Wang, Liang; Gao, Ying; Zhao, Zhong-Q.I.; Chen, Xin; Journal of Agricultural and Food Chemistry; vol. 58; 5; (2010); p. 3132 - 3138. |
| F1 | *Crataegus pinnatifida* | M023868 | Huang, Xiao-Xiao; Zhou, Chen-Chen; Li, Ling-Zhi; Li, Fei-Fei; Lou, Li-Li; Li, Dian-Ming; Ikejima, Takshi; Peng, Ying; Song, Shao-Jiang; Bioorganic and Medicinal Chemistry Letters; vol. 23; 20; (2013); p. 5599 - 5604. |
| F1 | *Crataegus pinnatifida* | M024200 | 阴健等. 中药现代研究与临床应用(1). 北京: 学苑出版社, 1993 |
| F1 | *Crataegus pinnatifida* | M024220 | 阴健等. 中药现代研究与临床应用(1). 北京: 学苑出版社, 1993 |
| F1 | *Crataegus pinnatifida* | M024294 | 阴健等. 中药现代研究与临床应用(1). 北京: 学苑出版社, 1993 |
| F1 | *Crataegus pinnatifida* | M024344 | Zhang, Pei-Cheng; Xu, Sui-Xu; Phytochemistry; vol. 57; 8; (2001); p. 1249 - 1253. |
| F1 | *Crataegus pinnatifida* | M025163 | 阴健等. 中药现代研究与临床应用(1). 北京: 学苑出版社, 1993 |
| F1 | *Crataegus pinnatifida* | M025188 | 阴健等. 中药现代研究与临床应用(1). 北京: 学苑出版社, 1993 |
| F1 | *Crataegus pinnatifida* | M025710 | 阴健等. 中药现代研究与临床应用(1). 北京: 学苑出版社, 1993 |
| F1 | *Crataegus pinnatifida* | M025760 | 张培成等. 药学学报, 2001, 35 (10): 754 |
| F1 | *Crataegus pinnatifida* | M025849 | (1) 阴健等. 中药现代研究与临床应用(1). 北京: 学苑出版社, 1993. (2) 刘丽娟等. 药学学报, 1992, 27 (11): 837. (3) 尚明英等. 中国中药杂志, 1998, 23 (10): 614. (4) 孙文基等. 天然活性成分简明手册. 北京: 中�揭┛萍汲霭嫔� 1998. (5) 国家中医药管理局《中华本草》编委会. 中华本草. Vol. 1-30 上海:上海科学技术出版社, 1999. (6) Buckingham J(Executive Editor): et al. Dictionary of Natural Products, Vol 1-7, Chapman & Hall, London, 1994; 1995, Vol 8; 1996, Vol 9; 1997, Vol 10; 1998, Vol 11.. (7) Xiang W, et al. Phytochemistry, 2004, 65, 1173. (8) McNally DJ, et al. JNP, 2003, 66 (9): 1280. (9) Deachathai S, et al. Phytochemistry, 2005, 66, 2368. (10) 欧明等. 简明中药成分手册. 北京: 中国医药科技出版社, 2003. (11) 刈米达夫著, 杨本文译. 植物化学, 科学出版社, 北京, 1985. |
| F1 | *Crataegus pinnatifida* | M026033 | 阴健等. 中药现代研究与临床应用(1). 北京: 学苑出版社, 1993 |
| F1 | *Crataegus pinnatifida* | M026236 | Bykov; Glyzin; Chemistry of Natural Compounds; vol. 8; (1972); p. 657; Khimiya Prirodnykh Soedinenii; vol. 8; (1972); p. 672. |
| F1 | *Crataegus pinnatifida* | M026253 | 阴健等. 中药现代研究与临床应用(1). 北京: 学苑出版社, 1993 |
| F1 | *Crataegus pinnatifida* | M026959 | (1) 阴健等. 中药现代研究与临床应用(1). 北京: 学苑出版社, 1993. (2) 季宇彬等. 中药抗肿瘤有效成分药理与应用. 哈尔滨: 黑龙江科学技术出版社, 1995. (3) 赵余庆等. 中国中药杂志, 1993, 18 (7): 428. (4) 孙文基等. 天然活性成分简明手册. 北京: 中国医药科技出版社, 1998. (5) 国家中医药管理局《中华本草》编委会. 中华本草. Vol. 1-30 上海:上海科学技术出版社, 1999. (6) 欧明等. 简明中药成分手册. 北京: 中国医药科技出版社, 2003. |
| F1 | *Crataegus pinnatifida* | M027455 | Li, Hai-Bo; Ying, Xi-Xiang; Lu, Jia; Natural Product Research; vol. 24; 18; (2010); p. 1695 - 1703. |
| F1 | *Crataegus pinnatifida* | M027501 | Moon, Hyung-In; Kim, Tae-im; Cho, Hyun-Soo; Kim, Eung Kweon; Bioorganic and Medicinal Chemistry Letters; vol. 20; 3; (2010); p. 991 - 993. |
| F1 | *Crataegus pinnatifida* | M028188 | (1) 阴健等. 中药现代研究与临床应用(1). 北京: 学苑出版社, 1993. (2) 孙文基等. 天然活性成分简明手册. 北京: 中国医药科技出版社, 1998. (3) 国家中医药管理局《中华本草》编委会. 中华本草. Vol. 1-30 上海:上海科学技术出版社, 1999. (4) OHASHI K, et al. Chem Pharm Bull, 2003, 51 (3): 343. (5) Morikawa T, et al. JNP, 2002, 65 (10): 1468. (6) Park WS, et al. Planta Med, 2003, 69, 459. |
| F1 | *Crataegus pinnatifida* | M028499 | (1) 江苏新医学院. 中药大辞典. 上海: 上海科学技术出版社, 1977. (2) 国家中医药管理局《中华本草》编委会. 中华本草. Vol. 1-30 上海:上海科学技术出版社, 1999. |
| F1 | *Crataegus pinnatifida* | M028648 | (1) 阴健等. 中药现代研究与临床应用(1). 北京: 学苑出版社, 1993. (2) 江苏新医学院. 中药大辞典. 上海: 上海科学技术出版社, 1977. (3) 钟永利等. 天然产物研究与开发, 1998, 10 (2): 15. (4) 国家中医药管理局《�谢静荨繁辔� 中华本草. Vol. 1-30 上海:上海科学技术出版社, 1999. (5) Krenn L, et al. JNP, 2003, 66 (8): 1107. (6) Chaubal R, et al. Planta Med, 2003, 69, 287. |
| F1 | *Crataegus pinnatifida* | M029505 | Min, Byung-Sun; Huong, Ha-Thi-Thanh; Kim, Jung-Hee; Jun, Hyun-Ju; Na, Min-Kyun; Nam, Nguyen-Hai; Lee, Hyeong-Kyu; Bae, KiHwan; Kang, Sam-Sik; Planta Medica; vol. 70; 12; (2004); p. 1166 - 1169. |
| F1 | *Crataegus pinnatifida* | M029645 | 阴健等. 中药现代研究与临床应用(1). 北京: 学苑出版社, 1993 |
| F1 | *Crataegus pinnatifida* | M030019 | 阴健等. 中药现代研究与临床应用(1). 北京: 学苑出版社, 1993 |
| F1 | *Crataegus pinnatifida* | M030107 | Bykov; Glyzin; Chemistry of Natural Compounds; vol. 8; (1972); p. 657; Khimiya Prirodnykh Soedinenii; vol. 8; (1972); p. 672. |
| F1 | *Crataegus pinnatifida* | M030226 | 阴健等. 中药现代研究与临床应用(1). 北京: 学苑出版社, 1993 |
| F1 | *Crataegus pinnatifida* | M030585 | Jeong, Tae-Sook; Hwang, Eui-Ll; Lee, Hyang-Bok; Lee, Eun-Sook; Kim, Young-Kook; Min, Byung-Sun; Bae, Ki-Hwan; Bok, Song-Hae; Kim, Sung-Uk; Planta Medica; vol. 65; 3; (1999); p. 261 - 263. |
| F1 | *Crataegus pinnatifida* | M030762 | Zhou, Chen-Chen; Huang, Xiao-Xiao; Gao, Pin-Yi; Li, Fei-Fei; Li, Dian-Ming; Li, Ling-Zhi; Song, Shao-Jiang; Journal of Asian Natural Products Research; vol. 16; 2; (2014); p. 169 - 174. |
| F1 | *Crataegus pinnatifida* | M030957 | Moon, Hyung-In; Kim, Tae-im; Cho, Hyun-Soo; Kim, Eung Kweon; Bioorganic and Medicinal Chemistry Letters; vol. 20; 3; (2010); p. 991 - 993. |
| F1 | *Crataegus pinnatifida* | M031056 | Huang, Xiao-Xiao; Zhou, Chen-Chen; Li, Ling-Zhi; Li, Fei-Fei; Lou, Li-Li; Li, Dian-Ming; Ikejima, Takshi; Peng, Ying; Song, Shao-Jiang; Bioorganic and Medicinal Chemistry Letters; vol. 23; 20; (2013); p. 5599 - 5604. |
| F1 | *Crataegus pinnatifida* | M031290 | Huang, Xiao-Xiao; Zhou, Chen-Chen; Li, Ling-Zhi; Li, Fei-Fei; Lou, Li-Li; Li, Dian-Ming; Ikejima, Takshi; Peng, Ying; Song, Shao-Jiang; Bioorganic and Medicinal Chemistry Letters; vol. 23; 20; (2013); p. 5599 - 5604. |
| F1 | *Crataegus pinnatifida* | M031668 | Min BS, et al. Planta Med, 2004, 70, 1166 |
| F1 | *Crataegus pinnatifida* | M032959 | 阴健等. 中药现代研究与临床应用(1). 北京: 学苑出版社, 1993 |
| F1 | *Crataegus pinnatifida* | M033429 | 阴健等. 中药现代研究与临床应用(1). 北京: 学苑出版社, 1993 |
| F1 | *Crataegus pinnatifida* | M033766 | 国家中医药管理局《中华本草》编委会. 中华本草. Vol. 1-30 上海:上海科学技术出版社, 1999 |
| F1 | *Crataegus pinnatifida* | M033832 | 阴健等. 中药现代研究与临床应用(1). 北京: 学苑出版社, 1993 |
| F1 | *Crataegus pinnatifida* | M034095 | Moon, Hyung-In; Kim, Tae-im; Cho, Hyun-Soo; Kim, Eung Kweon; Bioorganic and Medicinal Chemistry Letters; vol. 20; 3; (2010); p. 991 - 993. |
| F1 | *Dictamnus dasycarpus* | M000208 | Zhao, Weimin; Wolfender, Jean-Luc; Hostettmann, Kurt; Xu, Rensheng; Qin, Guowei; Phytochemistry; vol. 47; 1; (1998); p. 7 - 11. |
| F1 | *Dictamnus dasycarpus* | M000939 | Zhao, Weimin; Wolfender, Jean-Luc; Hostettmann, Kurt; Xu, Rensheng; Qin, Guowei; Phytochemistry; vol. 47; 1; (1998); p. 7 - 11. |
| F1 | *Dictamnus dasycarpus* | M001683 | Zhao, Weimin; Wolfender, Jean-Luc; Hostettmann, Kurt; Xu, Rensheng; Qin, Guowei; Phytochemistry; vol. 47; 1; (1998); p. 7 - 11. |
| F1 | *Dictamnus dasycarpus* | M002329 | Yang, Jun-Li; Liu, Lei-Lei; Shi, Yan-Ping; Planta Medica; vol. 77; 3; (2011); p. 271 - 276. |
| F1 | *Dictamnus dasycarpus* | M002357 | Zhao, Weimin; Wolfender, Jean-Luc; Hostettmann, Kurt; Xu, Rensheng; Qin, Guowei; Phytochemistry; vol. 47; 1; (1998); p. 7 - 11. |
| F1 | *Dictamnus dasycarpus* | M002677 | Chang; Xuan; Xu; Zhang; Journal of Natural Products; vol. 64; 7; (2001); p. 935 - 938. |
| F1 | *Dictamnus dasycarpus* | M004676 | Yoon, Jeong Seon; Sung, Sang Hyun; Kim, Young Choon; Journal of Natural Products; vol. 71; 2; (2008); p. 208 - 211. |
| F1 | *Dictamnus dasycarpus* | M004997 | Takeuchi; Fujita; Goto; Morisaki; Osone; Tobinaga; Chemical and Pharmaceutical Bulletin; vol. 41; 5; (1993); p. 923 - 925. |
| F1 | *Dictamnus dasycarpus* | M005948 | Zhao, Weimin; Wolfender, Jean-Luc; Hostettmann, Kurt; Xu, Rensheng; Qin, Guowei; Phytochemistry; vol. 47; 1; (1998); p. 7 - 11. |
| F1 | *Dictamnus dasycarpus* | M006253 | (1) 阴健等. 中药现代研究与临床应用(1). 北京: 学苑出版社, 1993. (2) 国家中医药管理局《中华本草》编委会. 中华本草. Vol. 1-30 上海:上海科学技术出版社, 1999. |
| F1 | *Dictamnus dasycarpus* | M006654 | Yoon, Jeong Seon; Sung, Sang Hyun; Kim, Young Choon; Journal of Natural Products; vol. 71; 2; (2008); p. 208 - 211. |
| F1 | *Dictamnus dasycarpus* | M006672 | Zhao, Weimin; Wolfender, Jean-Luc; Hostettmann, Kurt; Xu, Rensheng; Qin, Guowei; Phytochemistry; vol. 47; 1; (1998); p. 7 - 11. |
| F1 | *Dictamnus dasycarpus* | M006686 | Chang J, et al. JNP, 2001, 64 (7): 935 |
| F1 | *Dictamnus dasycarpus* | M006964 | Zhao, Weimin; Wolfender, Jean-Luc; Hostettmann, Kurt; Li, Hong-Yu; Stoeckli-Evans, Helen; Xu, Rensheng; Qin, Guowei; Phytochemistry; vol. 47; 1; (1998); p. 63 - 68. |
| F1 | *Dictamnus dasycarpus* | M007243 | (1) 阴健等. 中药现代研究与临床应用(1). 北京: 学苑出版社, 1993. (2) 孙文基等. 天然活性成分简明手册. 北京: 中国医药科技出版社, 1998. (3) 国家中医药管理局《中华本草》编委会. 中华本草. Vol. 1-30 上海:上海科学技术出版社, 1999. (4) Akihisa T, et al. Phytochemistry, 1991, 30 (7): 2369. (5) Behari M, et al.Chem. Abstr., 1987, 107, 151196x. |
| F1 | *Dictamnus dasycarpus* | M007471 | Yoon, Jeong Seon; Sung, Sang Hyun; Kim, Young Choon; Journal of Natural Products; vol. 71; 2; (2008); p. 208 - 211. |
| F1 | *Dictamnus dasycarpus* | M007601 | Chang, Jun; Xuan, Li-Jiang; Xu, Ya-Ming; Zhang, Jin-Sheng; Planta Medica; vol. 68; 5; (2002); p. 425 - 429. |
| F1 | *Dictamnus dasycarpus* | M008032 | Zhao, Weimin; Wolfender, Jean-Luc; Hostettmann, Kurt; Li, Hong-Yu; Stoeckli-Evans, Helen; Xu, Rensheng; Qin, Guowei; Phytochemistry; vol. 47; 1; (1998); p. 63 - 68. |
| F1 | *Dictamnus dasycarpus* | M008229 | 国家中医药管理局《中华本草》编委会. 中华本草. Vol. 1-30 上海:上海科学技术出版社, 1999 |
| F1 | *Dictamnus dasycarpus* | M008238 | Zhao, Weimin; Wolfender, Jean-Luc; Hostettmann, Kurt; Li, Hong-Yu; Stoeckli-Evans, Helen; Xu, Rensheng; Qin, Guowei; Phytochemistry; vol. 47; 1; (1998); p. 63 - 68. |
| F1 | *Dictamnus dasycarpus* | M008412 | Chang J, et al. JNP, 2001, 64 (7): 935 |
| F1 | *Dictamnus dasycarpus* | M009060 | Chang, Jun; Xuan, Li-Jiang; Xu, Ya-Ming; Zhang, Jin-Sheng; Planta Medica; vol. 68; 5; (2002); p. 425 - 429. |
| F1 | *Dictamnus dasycarpus* | M009473 | Yoon, Jeong Seon; Sung, Sang Hyun; Kim, Young Choon; Journal of Natural Products; vol. 71; 2; (2008); p. 208 - 211. |
| F1 | *Dictamnus dasycarpus* | M009673 | Zhao, Weimin; Wolfender, Jean-Luc; Hostettmann, Kurt; Xu, Rensheng; Qin, Guowei; Phytochemistry; vol. 47; 1; (1998); p. 7 - 11. |
| F1 | *Dictamnus dasycarpus* | M010008 | Zhao, Weimin; Wolfender, Jean-Luc; Hostettmann, Kurt; Xu, Rensheng; Qin, Guowei; Phytochemistry; vol. 47; 1; (1998); p. 7 - 11. |
| F1 | *Dictamnus dasycarpus* | M011344 | Chang J, et al. JNP, 2001, 64 (7): 935 |
| F1 | *Dictamnus dasycarpus* | M011933 | Chang J, et al. JNP, 2001, 64 (7): 935 |
| F1 | *Dictamnus dasycarpus* | M012020 | Zhao, Weimin; Wolfender, Jean-Luc; Hostettmann, Kurt; Xu, Rensheng; Qin, Guowei; Phytochemistry; vol. 47; 1; (1998); p. 7 - 11. |
| F1 | *Dictamnus dasycarpus* | M012178 | Yoon, Jeong Seon; Sung, Sang Hyun; Kim, Young Choon; Journal of Natural Products; vol. 71; 2; (2008); p. 208 - 211. |
| F1 | *Dictamnus dasycarpus* | M013001 | Takeuchi; Fujita; Goto; Morisaki; Osone; Tobinaga; Chemical and Pharmaceutical Bulletin; vol. 41; 5; (1993); p. 923 - 925. |
| F1 | *Dictamnus dasycarpus* | M013131 | Chang, Jun; Xuan, Li-Jiang; Xu, Ya-Ming; Zhang, Jin-Sheng; Planta Medica; vol. 68; 5; (2002); p. 425 - 429. |
| F1 | *Dictamnus dasycarpus* | M013826 | Yang, Jun-Li; Liu, Lei-Lei; Shi, Yan-Ping; Planta Medica; vol. 77; 3; (2011); p. 271 - 276. |
| F1 | *Dictamnus dasycarpus* | M014932 | Zhao, Weimin; Wolfender, Jean-Luc; Hostettmann, Kurt; Xu, Rensheng; Qin, Guowei; Phytochemistry; vol. 47; 1; (1998); p. 7 - 11. |
| F1 | *Dictamnus dasycarpus* | M015144 | Yang, Jun-Li; Liu, Lei-Lei; Shi, Yan-Ping; Planta Medica; vol. 77; 3; (2011); p. 271 - 276. |
| F1 | *Dictamnus dasycarpus* | M015149 | (1) 江苏新医学院. 中药大辞典. 上海: 上海科学技术出版社, 1977. (2) 宋振玉等. 中草药现代研究. 第2卷. 第28章 九里香. 333-361 北京: 北京医科大学中国协和医科大学联合出版社, 1996. (3) 孙文基等. 天然活性成分简明手册. 北京: 中国医药科技出版社, 1998. (4) Ito C, et al. JNP, 2004, 67 (11): 1800. (5) Hanawa F, et al. Planta Med, 2004, 70 (6): 531. (6) Chen J-J, et al. Planta Med, 2003, 69, 542. (7) 欧明等. 简明中药成分手册. 北京: 中国医药科技出版社, 2003. |
| F1 | *Dictamnus dasycarpus* | M015336 | Zhao; Wang; Qin; Xu; Hostettmann; Indian Journal of Chemistry - Section B Organic and Medicinal Chemistry; vol. 40; 8; (2001); p. 748 - 750. |
| F1 | *Dictamnus dasycarpus* | M015411 | Liu, Zhi Long; Xu, Yuan Jian; Wu, Jien; Goh, Swee Hock; Ho, Shuit Hung; Journal of agricultural and food chemistry; vol. 50; 6; (2002); p. 1447 - 1450. |
| F1 | *Dictamnus dasycarpus* | M015525 | (1) 阴健等. 中药现代研究与临床应用(1). 北京: 学苑出版社, 1993. (2) 江苏新医学院. 中药大辞典. 上海: 上海科学技术出版社, 1977. (3) 孙文基等. 天然活性成分简明手册. 北京: 中国医药科技出版社, 1998. |
| F1 | *Dictamnus dasycarpus* | M016464 | Chang; Xuan; Xu; Zhang; Journal of Natural Products; vol. 64; 7; (2001); p. 935 - 938. |
| F1 | *Dictamnus dasycarpus* | M017310 | Guo, Li-Na; Pei, Yue-Hu; Chen, Gang; Cong, Huan; Liu, Ji-Cheng; Journal of Asian Natural Products Research; vol. 14; 2; (2012); p. 105 - 110. |
| F1 | *Dictamnus dasycarpus* | M017311 | Guo, Li-Na; Pei, Yue-Hu; Chen, Gang; Cong, Huan; Liu, Ji-Cheng; Journal of Asian Natural Products Research; vol. 14; 2; (2012); p. 105 - 110. |
| F1 | *Dictamnus dasycarpus* | M017820 | Guo, Li-Na; Pei, Yue-Hu; Chen, Gang; Lu, Xuan; Xu, Hao; Liu, Ji-Cheng; Journal of Asian Natural Products Research; vol. 14; 3; (2012); p. 210 - 215. |
| F1 | *Dictamnus dasycarpus* | M018238 | Yang, Jun-Li; Liu, Lei-Lei; Shi, Yan-Ping; Planta Medica; vol. 77; 3; (2011); p. 271 - 276. |
| F1 | *Dictamnus dasycarpus* | M018662 | Yang, Jun-Li; Liu, Lei-Lei; Shi, Yan-Ping; Planta Medica; vol. 77; 3; (2011); p. 271 - 276. |
| F1 | *Dictamnus dasycarpus* | M018783 | 江苏新医学院. 中药大辞典. 上海: 上海科学技术出版社, 1977 |
| F1 | *Dictamnus dasycarpus* | M019514 | Yang, Jun-Li; Liu, Lei-Lei; Shi, Yan-Ping; Planta Medica; vol. 77; 3; (2011); p. 271 - 276. |
| F1 | *Dictamnus dasycarpus* | M020101 | Chen, Hong-Yu; Hu, Zhen-Yi; Tang, Chun-Ping; Quinn, Ronald J.; Feng, Yunjiang; Yao, Sheng; Ye, Yang; Tetrahedron Letters; vol. 54; 32; (2013); p. 4150 - 4153. |
| F1 | *Dictamnus dasycarpus* | M020498 | Chen, Hong-Yu; Hu, Zhen-Yi; Tang, Chun-Ping; Quinn, Ronald J.; Feng, Yunjiang; Yao, Sheng; Ye, Yang; Tetrahedron Letters; vol. 54; 32; (2013); p. 4150 - 4153. |
| F1 | *Dictamnus dasycarpus* | M020688 | Yang, Jun-Li; Liu, Lei-Lei; Shi, Yan-Ping; Planta Medica; vol. 77; 3; (2011); p. 271 - 276. |
| F1 | *Dictamnus dasycarpus* | M020920 | Chen, Hong-Yu; Hu, Zhen-Yi; Tang, Chun-Ping; Quinn, Ronald J.; Feng, Yunjiang; Yao, Sheng; Ye, Yang; Tetrahedron Letters; vol. 54; 32; (2013); p. 4150 - 4153. |
| F1 | *Dictamnus dasycarpus* | M021176 | Yang, Jun-Li; Liu, Lei-Lei; Shi, Yan-Ping; Planta Medica; vol. 77; 3; (2011); p. 271 - 276. |
| F1 | *Dictamnus dasycarpus* | M021210 | Guo, Li-Na; Pei, Yue-Hu; Chen, Gang; Lu, Xuan; Xu, Hao; Liu, Ji-Cheng; Journal of Asian Natural Products Research; vol. 14; 3; (2012); p. 210 - 215. |
| F1 | *Dictamnus dasycarpus* | M021649 | Guo, Li-Na; Pei, Yue-Hu; Chen, Gang; Lu, Xuan; Xu, Hao; Liu, Ji-Cheng; Journal of Asian Natural Products Research; vol. 14; 3; (2012); p. 210 - 215. |
| F1 | *Dictamnus dasycarpus* | M022194 | Yoon, Jeong Seon; Sung, Sang Hyun; Kim, Young Choon; Journal of Natural Products; vol. 71; 2; (2008); p. 208 - 211. |
| F1 | *Dictamnus dasycarpus* | M023414 | Yoon, Jeong Seon; Sung, Sang Hyun; Kim, Young Choon; Journal of Natural Products; vol. 71; 2; (2008); p. 208 - 211. |
| F1 | *Dictamnus dasycarpus* | M023861 | Zhao, Weimin; Wolfender, Jean-Luc; Hostettmann, Kurt; Xu, Rensheng; Qin, Guowei; Phytochemistry; vol. 47; 1; (1998); p. 7 - 11. |
| F1 | *Dictamnus dasycarpus* | M024671 | (1) Buckingham J(Executive Editor): et al. Dictionary of Natural Products, Vol 1-7, Chapman & Hall, London, 1994; 1995, Vol 8; 1996, Vol 9; 1997, Vol 10; 1998, Vol 11.. (2) 杜程芳等. 中国中药杂志, 2005, 30 (21): 1663. |
| F1 | *Dictamnus dasycarpus* | M024989 | (1) 江苏新医学院. 中药大辞典. 上海: 上海科学技术出版社, 1977. (2) 孙文基等. 天然活性成分简明手册. 北京: 中国医药科技出版社, 1998. (3) Ito C, et al. JNP, 2004, 67 (11): 1800. |
| F1 | *Dictamnus dasycarpus* | M025160 | Chang J, et al. JNP, 2001, 64 (7): 935 |
| F1 | *Dictamnus dasycarpus* | M025618 | Zhao; Wang; Qin; Xu; Hostettmann; Indian Journal of Chemistry - Section B Organic and Medicinal Chemistry; vol. 40; 8; (2001); p. 748 - 750. |
| F1 | *Dictamnus dasycarpus* | M025747 | Yoon, Jeong Seon; Sung, Sang Hyun; Kim, Young Choon; Journal of Natural Products; vol. 71; 2; (2008); p. 208 - 211. |
| F1 | *Dictamnus dasycarpus* | M026241 | Chang, Jun; Xuan, Li-Jiang; Xu, Ya-Ming; Zhang, Jin-Sheng; Planta Medica; vol. 68; 5; (2002); p. 425 - 429. |
| F1 | *Dictamnus dasycarpus* | M027019 | (1) 江苏新医学院. 中药大辞典. 上海: 上海科学技术出版社, 1977. (2) 孙文基等. 天然活性成分简明手册. 北京: 中国医药科技出版社, 1998. (3) 欧明等. 简明中药成分手册. 北京: 中国医药科技出版社, 2003. |
| F1 | *Dictamnus dasycarpus* | M027535 | (1) 国家中医药管理局《中华本草》编委会. 中华本草. Vol. 1-30 上海:上海科学技术出版社, 1999. (2) Storer R, et al. Tetrahedron, 1973, 29, 1217. |
| F1 | *Dictamnus dasycarpus* | M027906 | Zhao, Weimin; Wolfender, Jean-Luc; Hostettmann, Kurt; Xu, Rensheng; Qin, Guowei; Phytochemistry; vol. 47; 1; (1998); p. 7 - 11. |
| F1 | *Dictamnus dasycarpus* | M028148 | Zhao, Weimin; Wolfender, Jean-Luc; Hostettmann, Kurt; Li, Hong-Yu; Stoeckli-Evans, Helen; Xu, Rensheng; Qin, Guowei; Phytochemistry; vol. 47; 1; (1998); p. 63 - 68. |
| F1 | *Dictamnus dasycarpus* | M028258 | Chang J, et al. JNP, 2001, 64 (7): 935 |
| F1 | *Dictamnus dasycarpus* | M028363 | Chang; Xuan; Xu; Zhang; Journal of Natural Products; vol. 64; 7; (2001); p. 935 - 938. |
| F1 | *Dictamnus dasycarpus* | M028523 | Chang J, et al. JNP, 2001, 64 (7): 935 |
| F1 | *Dictamnus dasycarpus* | M029231 | Komissarenko, N. F.; Levashova, I. G.; Nadeshina, T. P.; Chemistry of Natural Compounds; vol. 19; 4; (1983); p. 502; Khimiya Prirodnykh Soedinenii; vol. 19; 4; (1983); p. 529 - 530. |
| F1 | *Dictamnus dasycarpus* | M029291 | Yoon, Jeong Seon; Sung, Sang Hyun; Kim, Young Choon; Journal of Natural Products; vol. 71; 2; (2008); p. 208 - 211. |
| F1 | *Dictamnus dasycarpus* | M029517 | Zhao, Weimin; Wolfender, Jean-Luc; Hostettmann, Kurt; Li, Hong-Yu; Stoeckli-Evans, Helen; Xu, Rensheng; Qin, Guowei; Phytochemistry; vol. 47; 1; (1998); p. 63 - 68. |
| F1 | *Dictamnus dasycarpus* | M030095 | Chang; Xuan; Xu; Zhang; Journal of Natural Products; vol. 64; 7; (2001); p. 935 - 938. |
| F1 | *Dictamnus dasycarpus* | M030718 | Chang; Xuan; Xu; Zhang; Journal of Natural Products; vol. 64; 7; (2001); p. 935 - 938. |
| F1 | *Dictamnus dasycarpus* | M031017 | Zhao, Weimin; Wolfender, Jean-Luc; Hostettmann, Kurt; Xu, Rensheng; Qin, Guowei; Phytochemistry; vol. 47; 1; (1998); p. 7 - 11. |
| F1 | *Dictamnus dasycarpus* | M031293 | Jung, HyunJu; Sok, Dai-Eun; Kim, YoungHo; Min, ByungSun; Lee, JongPill; Bae, KiHwan; Planta Medica; vol. 66; 1; (2000); p. 74 - 76. |
| F1 | *Dictamnus dasycarpus* | M031614 | Yoon, Jeong Seon; Sung, Sang Hyun; Kim, Young Choon; Journal of Natural Products; vol. 71; 2; (2008); p. 208 - 211. |
| F1 | *Dictamnus dasycarpus* | M031664 | Chang; Xuan; Xu; Zhang; Journal of Natural Products; vol. 64; 7; (2001); p. 935 - 938. |
| F1 | *Dictamnus dasycarpus* | M032693 | Yang, Jun-Li; Liu, Lei-Lei; Shi, Yan-Ping; Planta Medica; vol. 77; 3; (2011); p. 271 - 276. |
| F1 | *Dictamnus dasycarpus* | M032848 | Miyazawa, Mitsuo; Shimamura, Hideo; Nakamura, Sei-ichi; Kameoka, Hiromu; Journal of Agricultural and Food Chemistry; vol. 43; 6; (1995); p. 1428 - 1431. |
| F1 | *Dictamnus dasycarpus* | M033089 | Chang, Jun; Xuan, Li-Jiang; Xu, Ya-Ming; Zhang, Jin-Sheng; Planta Medica; vol. 68; 5; (2002); p. 425 - 429. |
| F1 | *Dictamnus dasycarpus* | M033337 | Chang; Xuan; Xu; Zhang; Journal of Natural Products; vol. 64; 7; (2001); p. 935 - 938. |
| F1 | *Dictamnus dasycarpus* | M033643 | Zhao, Weimin; Wolfender, Jean-Luc; Hostettmann, Kurt; Xu, Rensheng; Qin, Guowei; Phytochemistry; vol. 47; 1; (1998); p. 7 - 11. |
| F1 | *Dryopteris crassirhizoma* | M000236 | Ageta et al.; Chemical and Pharmaceutical Bulletin; vol. 11; (1963); p. 408. |
| F1 | *Dryopteris crassirhizoma* | M000568 | Lee, Ji Suk; Miyashiro, Hirotsugu; Nakamura, Norio; Hattori, Masao; Chemical and Pharmaceutical Bulletin; vol. 56; 5; (2008); p. 711 - 714. |
| F1 | *Dryopteris crassirhizoma* | M003201 | MIN B-S, et al. Chem Pharm Bull, 2001, 49 (5): 546 |
| F1 | *Dryopteris crassirhizoma* | M003422 | Min; Tomiyama; Ma; Nakamura; Hattori; Chemical and Pharmaceutical Bulletin; vol. 49; 5; (2001); p. 546 - 550. |
| F1 | *Dryopteris crassirhizoma* | M004652 | MIN B-S, et al. Chem Pharm Bull, 2001, 49 (5): 546 |
| F1 | *Dryopteris crassirhizoma* | M004782 | 江苏新医学院. 中药大辞典. 上海: 上海科学技术出版社, 1977 |
| F1 | *Dryopteris crassirhizoma* | M004952 | Chang, Xiaolong; Li, Wei; Koike, Kazuo; Wu, Lijun; Nikaido, Tamotsu; Chemical and Pharmaceutical Bulletin; vol. 54; 5; (2006); p. 748 - 750. |
| F1 | *Dryopteris crassirhizoma* | M004977 | Ageta et al.; Tetrahedron Letters; (1975); p. 3297. |
| F1 | *Dryopteris crassirhizoma* | M005622 | Noro et al.; Phytochemistry (Elsevier); vol. 12; (1973); p. 1491. |
| F1 | *Dryopteris crassirhizoma* | M005874 | 江苏新医学院. 中药大辞典. 上海: 上海科学技术出版社, 1977 |
| F1 | *Dryopteris crassirhizoma* | M006297 | Lee, Ji Suk; Miyashiro, Hirotsugu; Nakamura, Norio; Hattori, Masao; Chemical and Pharmaceutical Bulletin; vol. 56; 5; (2008); p. 711 - 714. |
| F1 | *Dryopteris crassirhizoma* | M006763 | (1) 阴健等. 中药现代研究与临床应用(1). 北京: 学苑出版社, 1993. (2) 季宇彬等. 中药抗肿瘤有效成分药理与应用. 哈尔滨: 黑龙江科学技术出版社, 1995. (3) 孙文基等. 天然活性成分简明手册. 北京: 中国医药科技出�嫔� 1998. (4) 国家中医药管理局《中华本草》编委会. 中华本草. Vol. 1-30 上海:上海科学技术出版社, 1999. (5) Park S-H, et al. Planta Med, 2005, 71 (1): 24. (6) 欧明等. 简明中药成分手册. 北京: 中国医药科技出版社, 2003. |
| F1 | *Dryopteris crassirhizoma* | M006830 | Lee, Ji Suk; Miyashiro, Hirotsugu; Nakamura, Norio; Hattori, Masao; Chemical and Pharmaceutical Bulletin; vol. 56; 5; (2008); p. 711 - 714. |
| F1 | *Dryopteris crassirhizoma* | M006957 | Lee, Sang-Myung; Na, Min-Kyun; Na, Ren-Bo; Min, Byung-Sun; Lee, Hyeong-Kyu; Biological and Pharmaceutical Bulletin; vol. 26; 9; (2003); p. 1354 - 1356. |
| F1 | *Dryopteris crassirhizoma* | M006974 | Chang, Xiaolong; Li, Wei; Koike, Kazuo; Wu, Lijun; Nikaido, Tamotsu; Chemical and Pharmaceutical Bulletin; vol. 54; 5; (2006); p. 748 - 750. |
| F1 | *Dryopteris crassirhizoma* | M007272 | Lee, Ji Suk; Miyashiro, Hirotsugu; Nakamura, Norio; Hattori, Masao; Chemical and Pharmaceutical Bulletin; vol. 56; 5; (2008); p. 711 - 714. |
| F1 | *Dryopteris crassirhizoma* | M007751 | Na, MinKyun; Jang, JunPil; Min, Byung Sun; Lee, Sang Jun; Lee, Myung Sun; Kim, Bo Yeon; Oh, Won Keun; Ahn, Jong Seog; Bioorganic and Medicinal Chemistry Letters; vol. 16; 18; (2006); p. 4738 - 4742. |
| F1 | *Dryopteris crassirhizoma* | M008725 | Chang, Xiaolong; Li, Wei; Koike, Kazuo; Wu, Lijun; Nikaido, Tamotsu; Chemical and Pharmaceutical Bulletin; vol. 54; 5; (2006); p. 748 - 750. |
| F1 | *Dryopteris crassirhizoma* | M009436 | Lee, Ji Suk; Miyashiro, Hirotsugu; Nakamura, Norio; Hattori, Masao; Chemical and Pharmaceutical Bulletin; vol. 56; 5; (2008); p. 711 - 714. |
| F1 | *Dryopteris crassirhizoma* | M009743 | Lee, Ji Suk; Miyashiro, Hirotsugu; Nakamura, Norio; Hattori, Masao; Chemical and Pharmaceutical Bulletin; vol. 56; 5; (2008); p. 711 - 714. |
| F1 | *Dryopteris crassirhizoma* | M010568 | Shinozaki, Junichi; Shibuya, Masaaki; Masuda, Kazuo; Ebizuka, Yutaka; Phytochemistry; vol. 69; 14; (2008); p. 2559 - 2564. |
| F1 | *Dryopteris crassirhizoma* | M010653 | Lee, Ji Suk; Miyashiro, Hirotsugu; Nakamura, Norio; Hattori, Masao; Chemical and Pharmaceutical Bulletin; vol. 56; 5; (2008); p. 711 - 714. |
| F1 | *Dryopteris crassirhizoma* | M010969 | (1) 江苏新医学院. 中药大辞典. 上海: 上海科学技术出版社, 1977. (2) Buckingham J(Executive Editor): et al. Dictionary of Natural Products, Vol 1-7, Chapman & Hall, London, 1994; 1995, Vol 8; 1996, Vol 9; 1997, Vol 10; 1998, Vol 11.. |
| F1 | *Dryopteris crassirhizoma* | M011329 | Na, MinKyun; Jang, JunPil; Min, Byung Sun; Lee, Sang Jun; Lee, Myung Sun; Kim, Bo Yeon; Oh, Won Keun; Ahn, Jong Seog; Bioorganic and Medicinal Chemistry Letters; vol. 16; 18; (2006); p. 4738 - 4742. |
| F1 | *Dryopteris crassirhizoma* | M011465 | Na, MinKyun; Jang, JunPil; Min, Byung Sun; Lee, Sang Jun; Lee, Myung Sun; Kim, Bo Yeon; Oh, Won Keun; Ahn, Jong Seog; Bioorganic and Medicinal Chemistry Letters; vol. 16; 18; (2006); p. 4738 - 4742. |
| F1 | *Dryopteris crassirhizoma* | M011564 | Chang, Xiaolong; Li, Wei; Koike, Kazuo; Wu, Lijun; Nikaido, Tamotsu; Chemical and Pharmaceutical Bulletin; vol. 54; 5; (2006); p. 748 - 750. |
| F1 | *Dryopteris crassirhizoma* | M011647 | (1) 江苏新医学院. 中药大辞典. 上海: 上海科学技术出版社, 1977. (2) Buckingham J(Executive Editor): et al. Dictionary of Natural Products, Vol 1-7, Chapman & Hall, London, 1994; 1995, Vol 8; 1996, Vol 9; 1997, Vol 10; 1998, Vol 11.. |
| F1 | *Dryopteris crassirhizoma* | M012293 | Na, MinKyun; Jang, JunPil; Min, Byung Sun; Lee, Sang Jun; Lee, Myung Sun; Kim, Bo Yeon; Oh, Won Keun; Ahn, Jong Seog; Bioorganic and Medicinal Chemistry Letters; vol. 16; 18; (2006); p. 4738 - 4742. |
| F1 | *Dryopteris crassirhizoma* | M013157 | Na, MinKyun; Jang, JunPil; Min, Byung Sun; Lee, Sang Jun; Lee, Myung Sun; Kim, Bo Yeon; Oh, Won Keun; Ahn, Jong Seog; Bioorganic and Medicinal Chemistry Letters; vol. 16; 18; (2006); p. 4738 - 4742. |
| F1 | *Dryopteris crassirhizoma* | M013275 | Shiojima, Kenji; Arai, Yoko; Ageta, Hiroyuki; Phytochemistry (Elsevier); vol. 29; 4; (1990); p. 1079 - 1082. |
| F1 | *Dryopteris crassirhizoma* | M013708 | (1) 江苏新医学院. 中药大辞典. 上海: 上海科学技术出版社, 1977. (2) 国家中医药管理局《中华本草》编委会. 中华本草. Vol. 1-30 上海:上海科学技术出版社, 1999. (3) Buckingham J(Executive Editor): et al. Dictionary of Natural Products, Vol 1-7, Chapman & Hall, London, 1994; 1995, Vol 8; 1996, Vol 9; 1997, Vol 10; 1998, Vol 11.. |
| F1 | *Dryopteris crassirhizoma* | M013948 | (1) 江苏新医学院. 中药大辞典. 上海: 上海科学技术出版社, 1977. (2) 孙文基等. 天然活性成分简明手册. 北京: 中国医药科技出版社, 1998. |
| F1 | *Dryopteris crassirhizoma* | M014422 | Ageta et al.; Tetrahedron Letters; (1975); p. 3297. |
| F1 | *Dryopteris crassirhizoma* | M014578 | Lee, Ji Suk; Miyashiro, Hirotsugu; Nakamura, Norio; Hattori, Masao; Chemical and Pharmaceutical Bulletin; vol. 56; 5; (2008); p. 711 - 714. |
| F1 | *Dryopteris crassirhizoma* | M015288 | Lee, Ji Suk; Miyashiro, Hirotsugu; Nakamura, Norio; Hattori, Masao; Chemical and Pharmaceutical Bulletin; vol. 56; 5; (2008); p. 711 - 714. |
| F1 | *Dryopteris crassirhizoma* | M015323 | MIN B-S, et al. Chem Pharm Bull, 2001, 49 (5): 546 |
| F1 | *Dryopteris crassirhizoma* | M015487 | Lee, Ji Suk; Miyashiro, Hirotsugu; Nakamura, Norio; Hattori, Masao; Chemical and Pharmaceutical Bulletin; vol. 56; 5; (2008); p. 711 - 714. |
| F1 | *Dryopteris crassirhizoma* | M021081 | Lee, Ji Suk; Miyashiro, Hirotsugu; Nakamura, Norio; Hattori, Masao; Chemical and Pharmaceutical Bulletin; vol. 56; 5; (2008); p. 711 - 714. |
| F1 | *Dryopteris crassirhizoma* | M022289 | 江苏新医学院. 中药大辞典. 上海: 上海科学技术出版社, 1977 |
| F1 | *Dryopteris crassirhizoma* | M022943 | Chang, Xiaolong; Li, Wei; Koike, Kazuo; Wu, Lijun; Nikaido, Tamotsu; Chemical and Pharmaceutical Bulletin; vol. 54; 5; (2006); p. 748 - 750. |
| F1 | *Dryopteris crassirhizoma* | M023087 | Lee, Sang-Myung; Na, Min-Kyun; Na, Ren-Bo; Min, Byung-Sun; Lee, Hyeong-Kyu; Biological and Pharmaceutical Bulletin; vol. 26; 9; (2003); p. 1354 - 1356. |
| F1 | *Dryopteris crassirhizoma* | M023347 | Chang, Xiaolong; Li, Wei; Koike, Kazuo; Wu, Lijun; Nikaido, Tamotsu; Chemical and Pharmaceutical Bulletin; vol. 54; 5; (2006); p. 748 - 750. |
| F1 | *Dryopteris crassirhizoma* | M023536 | Min; Tomiyama; Ma; Nakamura; Hattori; Chemical and Pharmaceutical Bulletin; vol. 49; 5; (2001); p. 546 - 550. |
| F1 | *Dryopteris crassirhizoma* | M023769 | Na, MinKyun; Jang, JunPil; Min, Byung Sun; Lee, Sang Jun; Lee, Myung Sun; Kim, Bo Yeon; Oh, Won Keun; Ahn, Jong Seog; Bioorganic and Medicinal Chemistry Letters; vol. 16; 18; (2006); p. 4738 - 4742. |
| F1 | *Dryopteris crassirhizoma* | M024014 | 江苏新医学院. 中药大辞典. 上海: 上海科学技术出版社, 1977 |
| F1 | *Dryopteris crassirhizoma* | M024559 | Na, MinKyun; Jang, JunPil; Min, Byung Sun; Lee, Sang Jun; Lee, Myung Sun; Kim, Bo Yeon; Oh, Won Keun; Ahn, Jong Seog; Bioorganic and Medicinal Chemistry Letters; vol. 16; 18; (2006); p. 4738 - 4742. |
| F1 | *Dryopteris crassirhizoma* | M025062 | 江苏新医学院. 中药大辞典. 上海: 上海科学技术出版社, 1977 |
| F1 | *Dryopteris crassirhizoma* | M025914 | Min; Tomiyama; Ma; Nakamura; Hattori; Chemical and Pharmaceutical Bulletin; vol. 49; 5; (2001); p. 546 - 550. |
| F1 | *Dryopteris crassirhizoma* | M025978 | MIN B-S, et al. Chem Pharm Bull, 2001, 49 (5): 546 |
| F1 | *Dryopteris crassirhizoma* | M027323 | Shiojima; Suzuki; Matsumura; Ageta; Chemical and Pharmaceutical Bulletin; vol. 42; 2; (1994); p. 377 - 378. |
| F1 | *Dryopteris crassirhizoma* | M027461 | Retrieved from CNPD |
| F1 | *Dryopteris crassirhizoma* | M028068 | Lee, Ji Suk; Miyashiro, Hirotsugu; Nakamura, Norio; Hattori, Masao; Chemical and Pharmaceutical Bulletin; vol. 56; 5; (2008); p. 711 - 714. |
| F1 | *Dryopteris crassirhizoma* | M028141 | Ageta et al.; Chemical and Pharmaceutical Bulletin; vol. 11; (1963); p. 408. |
| F1 | *Dryopteris crassirhizoma* | M028158 | Retrieved from CNPD |
| F1 | *Dryopteris crassirhizoma* | M028941 | Retrieved from CNPD |
| F1 | *Dryopteris crassirhizoma* | M030960 | Lee, Ji Suk; Miyashiro, Hirotsugu; Nakamura, Norio; Hattori, Masao; Chemical and Pharmaceutical Bulletin; vol. 56; 5; (2008); p. 711 - 714. |
| F1 | *Dryopteris crassirhizoma* | M031236 | Lee, Ji Suk; Miyashiro, Hirotsugu; Nakamura, Norio; Hattori, Masao; Chemical and Pharmaceutical Bulletin; vol. 56; 5; (2008); p. 711 - 714. |
| F1 | *Dryopteris crassirhizoma* | M031332 | Lee, Ji Suk; Miyashiro, Hirotsugu; Nakamura, Norio; Hattori, Masao; Chemical and Pharmaceutical Bulletin; vol. 56; 5; (2008); p. 711 - 714. |
| F1 | *Dryopteris crassirhizoma* | M031701 | Na, MinKyun; Jang, JunPil; Min, Byung Sun; Lee, Sang Jun; Lee, Myung Sun; Kim, Bo Yeon; Oh, Won Keun; Ahn, Jong Seog; Bioorganic and Medicinal Chemistry Letters; vol. 16; 18; (2006); p. 4738 - 4742. |
| F1 | *Dryopteris crassirhizoma* | M031754 | Lee, Ji Suk; Miyashiro, Hirotsugu; Nakamura, Norio; Hattori, Masao; Chemical and Pharmaceutical Bulletin; vol. 56; 5; (2008); p. 711 - 714. |
| F1 | *Dryopteris crassirhizoma* | M033307 | 江苏新医学院. 中药大辞典. 上海: 上海科学技术出版社, 1977 |
| F1 | *Dryopteris crassirhizoma* | M033847 | Lee, Ji Suk; Miyashiro, Hirotsugu; Nakamura, Norio; Hattori, Masao; Chemical and Pharmaceutical Bulletin; vol. 56; 5; (2008); p. 711 - 714. |
| F1 | *Dryopteris crassirhizoma* | M033978 | Min; Tomiyama; Ma; Nakamura; Hattori; Chemical and Pharmaceutical Bulletin; vol. 49; 5; (2001); p. 546 - 550. |
| F1 | *Dryopteris crassirhizoma* | M034370 | Lee, Ji Suk; Miyashiro, Hirotsugu; Nakamura, Norio; Hattori, Masao; Chemical and Pharmaceutical Bulletin; vol. 56; 5; (2008); p. 711 - 714. |
| F1 | *Portulaca oleracea* | M000668 | Sakai, Naomi; Inada, Kyouko; Okamoto, Michi; Shizuri, Yoshikazu; Fukuyama, Yoshiyasu; Phytochemistry; vol. 42; 6; (1996); p. 1625 - 1628. |
| F1 | *Portulaca oleracea* | M001019 | Xiang, Lan; Xing, Dongming; Wang, Wei; Wang, Rufeng; Ding, Yi; Du, Lijun; Phytochemistry; vol. 66; 21; (2005); p. 2595 - 2601. |
| F1 | *Portulaca oleracea* | M001470 | Imperato; Phytochemistry (Elsevier); vol. 14; (1975); p. 2091. |
| F1 | *Portulaca oleracea* | M002156 | Tian, Jin-Long; Liang, Xiao; Gao, Pin-Yi; Li, Dan-Qi; Sun, Qian; Li, Ling-Zhi; Song, Shao-Jiang; Journal of Asian Natural Products Research; vol. 16; 3; (2014); p. 259 - 264. |
| F1 | *Portulaca oleracea* | M002497 | Xiang L, et al. Phytochemistry, 2005, 66, 2595 |
| F1 | *Portulaca oleracea* | M002520 | Tulloch; Lipids; vol. 9; (1974); p. 664,665. |
| F1 | *Portulaca oleracea* | M003914 | Tulloch; Lipids; vol. 9; (1974); p. 664,665. |
| F1 | *Portulaca oleracea* | M004480 | Xiang L, et al. Phytochemistry, 2005, 66, 2595 |
| F1 | *Portulaca oleracea* | M006981 | Yan, Jian; Sun, Li-Rong; Zhou, Zhong-Yu; Chen, Yu-Chan; Zhang, Wei-Min; Dai, Hao-Fu; Tan, Jian-Wen; Phytochemistry; vol. 80; (2012); p. 37 - 41. |
| F1 | *Portulaca oleracea* | M007192 | Yan, Jian; Sun, Li-Rong; Zhou, Zhong-Yu; Chen, Yu-Chan; Zhang, Wei-Min; Dai, Hao-Fu; Tan, Jian-Wen; Phytochemistry; vol. 80; (2012); p. 37 - 41. |
| F1 | *Portulaca oleracea* | M009554 | Yan, Jian; Sun, Li-Rong; Zhou, Zhong-Yu; Chen, Yu-Chan; Zhang, Wei-Min; Dai, Hao-Fu; Tan, Jian-Wen; Phytochemistry; vol. 80; (2012); p. 37 - 41. |
| F1 | *Portulaca oleracea* | M010694 | (1) 江苏新医学院. 中药大辞典. 上海: 上海科学技术出版社, 1977. (2) 孙文基等. 天然活性成分简明手册. 北京: 中国医药科技出版社, 1998. |
| F1 | *Portulaca oleracea* | M010766 | Xiang L, et al. Phytochemistry, 2005, 66, 2595 |
| F1 | *Portulaca oleracea* | M012512 | Liu, Dianyu; Shen, Tao; Xiang, Lan; Helvetica Chimica Acta; vol. 94; 3; (2011); p. 497 - 501. |
| F1 | *Portulaca oleracea* | M012680 | 国家中医药管理局《中华本草》编委会. 中华本草. Vol. 1-30 上海:上海科学技术出版社, 1999 |
| F1 | *Portulaca oleracea* | M015819 | Xiang, Lan; Xing, Dongming; Wang, Wei; Wang, Rufeng; Ding, Yi; Du, Lijun; Phytochemistry; vol. 66; 21; (2005); p. 2595 - 2601. |
| F1 | *Portulaca oleracea* | M017421 | Wu, Bin; Yu, Liyan; Wu, Xiaodan; Chen, Jianbo; Carbohydrate Research; vol. 351; (2012); p. 68 - 71. |
| F1 | *Portulaca oleracea* | M017422 | Wu, Bin; Yu, Liyan; Wu, Xiaodan; Chen, Jianbo; Carbohydrate Research; vol. 351; (2012); p. 68 - 71. |
| F1 | *Portulaca oleracea* | M017423 | Wu, Bin; Yu, Liyan; Wu, Xiaodan; Chen, Jianbo; Carbohydrate Research; vol. 351; (2012); p. 68 - 71. |
| F1 | *Portulaca oleracea* | M017822 | Yan, Jian; Sun, Li-Rong; Zhou, Zhong-Yu; Chen, Yu-Chan; Zhang, Wei-Min; Dai, Hao-Fu; Tan, Jian-Wen; Phytochemistry; vol. 80; (2012); p. 37 - 41. |
| F1 | *Portulaca oleracea* | M018101 | Tian, Jin-Long; Liang, Xiao; Gao, Pin-Yi; Li, Dan-Qi; Sun, Qian; Li, Ling-Zhi; Song, Shao-Jiang; Journal of Asian Natural Products Research; vol. 16; 3; (2014); p. 259 - 264. |
| F1 | *Portulaca oleracea* | M018275 | Yan, Jian; Sun, Li-Rong; Zhou, Zhong-Yu; Chen, Yu-Chan; Zhang, Wei-Min; Dai, Hao-Fu; Tan, Jian-Wen; Phytochemistry; vol. 80; (2012); p. 37 - 41. |
| F1 | *Portulaca oleracea* | M018536 | Tian, Jin-Long; Liang, Xiao; Gao, Pin-Yi; Li, Dan-Qi; Sun, Qian; Li, Ling-Zhi; Song, Shao-Jiang; Journal of Asian Natural Products Research; vol. 16; 3; (2014); p. 259 - 264. |
| F1 | *Portulaca oleracea* | M019033 | Xiang, Lan; Xing, Dongming; Wang, Wei; Wang, Rufeng; Ding, Yi; Du, Lijun; Phytochemistry; vol. 66; 21; (2005); p. 2595 - 2601. |
| F1 | *Portulaca oleracea* | M019824 | Yan, Jian; Sun, Li-Rong; Zhou, Zhong-Yu; Chen, Yu-Chan; Zhang, Wei-Min; Dai, Hao-Fu; Tan, Jian-Wen; Phytochemistry; vol. 80; (2012); p. 37 - 41. |
| F1 | *Portulaca oleracea* | M020632 | Yan, Jian; Sun, Li-Rong; Zhou, Zhong-Yu; Chen, Yu-Chan; Zhang, Wei-Min; Dai, Hao-Fu; Tan, Jian-Wen; Phytochemistry; vol. 80; (2012); p. 37 - 41. |
| F1 | *Portulaca oleracea* | M020662 | (1) 国家中医药管理局《中华本草》编委会. 中华本草. Vol. 1-30 上海:上海科学技术出版社, 1999. (2) 孙文基等. 天然活性成分简明手册. 北京: 中国医药科技出版社, 1998. |
| F1 | *Portulaca oleracea* | M021214 | Yan, Jian; Sun, Li-Rong; Zhou, Zhong-Yu; Chen, Yu-Chan; Zhang, Wei-Min; Dai, Hao-Fu; Tan, Jian-Wen; Phytochemistry; vol. 80; (2012); p. 37 - 41. |
| F1 | *Portulaca oleracea* | M021447 | Zheng, Guo-Yin; Qu, Li-Ping; Yue, Xiao-Qiang; Gu, Wei; Zhang, Hong; Xin, Hai-Liang; Phytochemistry Letters; vol. 7; 1; (2014); p. 77 - 84. |
| F1 | *Portulaca oleracea* | M021652 | Yan, Jian; Sun, Li-Rong; Zhou, Zhong-Yu; Chen, Yu-Chan; Zhang, Wei-Min; Dai, Hao-Fu; Tan, Jian-Wen; Phytochemistry; vol. 80; (2012); p. 37 - 41. |
| F1 | *Portulaca oleracea* | M021926 | Tian, Jin-Long; Liang, Xiao; Gao, Pin-Yi; Li, Dan-Qi; Sun, Qian; Li, Ling-Zhi; Song, Shao-Jiang; Journal of Asian Natural Products Research; vol. 16; 3; (2014); p. 259 - 264. |
| F1 | *Portulaca oleracea* | M022787 | Hofmann, Diana; Knop, Mona; Hao, Huang; Hennig, Lothar; Sicker, Dieter; Schulz, Margot; Journal of Natural Products; vol. 69; 1; (2006); p. 34 - 37. |
| F1 | *Portulaca oleracea* | M022994 | Xiang L, et al. Phytochemistry, 2005, 66, 2595 |
| F1 | *Portulaca oleracea* | M026176 | Liu, Dianyu; Shen, Tao; Xiang, Lan; Helvetica Chimica Acta; vol. 94; 3; (2011); p. 497 - 501. |
| F1 | *Portulaca oleracea* | M029182 | Xiang, Lan; Xing, Dongming; Wang, Wei; Wang, Rufeng; Ding, Yi; Du, Lijun; Phytochemistry; vol. 66; 21; (2005); p. 2595 - 2601. |
| F1 | *Portulaca oleracea* | M029482 | Hofmann, Diana; Knop, Mona; Hao, Huang; Hennig, Lothar; Sicker, Dieter; Schulz, Margot; Journal of Natural Products; vol. 69; 1; (2006); p. 34 - 37. |
| F1 | *Portulaca oleracea* | M031003 | Imperato; Phytochemistry (Elsevier); vol. 14; (1975); p. 2091. |
| F1 | *Portulaca oleracea* | M031226 | Xin, Hai-Liang; Xu, Yan-Feng; Hou, Yin-Huan; Zhang, Ya-Ni; Yue, Xiao-Qiang; Lu, Jin-Cai; Ling, Chang-Quan; Helvetica Chimica Acta; vol. 91; 11; (2008); p. 2075 - 2080. |
| F1 | *Portulaca oleracea* | M032062 | Wu, Bin; Yu, Liyan; Wu, Xiaodan; Chen, Jianbo; Carbohydrate Research; vol. 351; (2012); p. 68 - 71. |
| F1 | *Portulaca oleracea* | M034108 | Wu, Bin; Yu, Liyan; Wu, Xiaodan; Chen, Jianbo; Carbohydrate Research; vol. 351; (2012); p. 68 - 71. |
| F1 | *Portulaca oleracea* | M034278 | (1) 阴健等. 中药现代研究与临床应用(1). 北京: 学苑出版社, 1993. (2) 江苏新医学院. 中药大辞典. 上海: 上海科学技术出版社, 1977. (3) 孙文基等. 天然活性成分简明手册. 北京: 中国医药科技出版社, 1998. |
| F1 | *Prunus mume* | M001420 | Hasegawa; Journal of Organic Chemistry; vol. 24; (1959); p. 408. |
| F1 | *Prunus mume* | M001552 | Retrieved from CNPD |
| F1 | *Prunus mume* | M002773 | Nakamura, Seikou; Fujimoto, Katsuyoshi; Matsumoto, Takahiro; Nakashima, Souichi; Ohta, Tomoe; Ogawa, Keiko; Matsuda, Hisashi; Yoshikawa, Masayuki; Phytochemistry; vol. 92; (2013); p. 128 - 136. |
| F1 | *Prunus mume* | M004344 | Fujimoto, Katsuyoshi; Nakamura, Seikou; Matsumoto, Takahiro; Ohta, Tomoe; Ogawa, Keiko; Tamura, Haruka; Matsuda, Hisashi; Yoshikawa, Masayuki; Chemical and Pharmaceutical Bulletin; vol. 61; 4; (2013); p. 445 - 451. |
| F1 | *Prunus mume* | M004795 | (1) 江苏新医学院. 中药大辞典. 上海: 上海科学技术出版社, 1977. (2) 国家中医药管理局《中华本草》编委会. 中华本草. Vol. 1-30 上海:上海科学技术出版社, 1999. |
| F1 | *Prunus mume* | M007010 | Yoshikawa M, et al. JNP, 2002, 65 (8): 1151 |
| F1 | *Prunus mume* | M007650 | Retrieved from CNPD |
| F1 | *Prunus mume* | M007919 | Nakamura, Seikou; Fujimoto, Katsuyoshi; Matsumoto, Takahiro; Nakashima, Souichi; Ohta, Tomoe; Ogawa, Keiko; Matsuda, Hisashi; Yoshikawa, Masayuki; Phytochemistry; vol. 92; (2013); p. 128 - 136. |
| F1 | *Prunus mume* | M007945 | Nakamura, Seikou; Fujimoto, Katsuyoshi; Matsumoto, Takahiro; Nakashima, Souichi; Ohta, Tomoe; Ogawa, Keiko; Matsuda, Hisashi; Yoshikawa, Masayuki; Phytochemistry; vol. 92; (2013); p. 128 - 136. |
| F1 | *Prunus mume* | M008170 | Fujimoto, Katsuyoshi; Nakamura, Seikou; Matsumoto, Takahiro; Ohta, Tomoe; Ogawa, Keiko; Tamura, Haruka; Matsuda, Hisashi; Yoshikawa, Masayuki; Chemical and Pharmaceutical Bulletin; vol. 61; 4; (2013); p. 445 - 451. |
| F1 | *Prunus mume* | M008461 | Yoshikawa M, et al. JNP, 2002, 65 (8): 1151 |
| F1 | *Prunus mume* | M009171 | Nakamura, Seikou; Fujimoto, Katsuyoshi; Matsumoto, Takahiro; Nakashima, Souichi; Ohta, Tomoe; Ogawa, Keiko; Matsuda, Hisashi; Yoshikawa, Masayuki; Phytochemistry; vol. 92; (2013); p. 128 - 136. |
| F1 | *Prunus mume* | M009270 | Nakamura, Seikou; Fujimoto, Katsuyoshi; Matsumoto, Takahiro; Nakashima, Souichi; Ohta, Tomoe; Ogawa, Keiko; Matsuda, Hisashi; Yoshikawa, Masayuki; Phytochemistry; vol. 92; (2013); p. 128 - 136. |
| F1 | *Prunus mume* | M009585 | Fujimoto, Katsuyoshi; Nakamura, Seikou; Matsumoto, Takahiro; Ohta, Tomoe; Ogawa, Keiko; Tamura, Haruka; Matsuda, Hisashi; Yoshikawa, Masayuki; Chemical and Pharmaceutical Bulletin; vol. 61; 4; (2013); p. 445 - 451. |
| F1 | *Prunus mume* | M009790 | (1) Kanchanapoom T, et al. Chem Pharm Bull, 2004, 52 (8): 980. (2) Miyase T, et al. Chem Pharm Bull, 1987, 35, 1109. (3) DOI K, et al. Chem Pharm Bull, 2001, 49 (2): 151. (4) ISHIKAWA T, et al. Chem Pharm Bull, 2001, 49 (5): 584. (5) ISHIKAWA T, et al. Chem Pharm Bull, 2002, 50 (4): 501. (6) HUA H, et al. Chem Pharm Bull, 2002, 50 (10): 1393. (7) KITAJIMA J, et al. Chem Pharm Bull, 2003, 51 (7): 890. (8) Yoshikawa M, et al. JNP, 2002, 65 (8): 1151. (9) Braca A, et al. Planta Med, 2004, 70 (10): 960. |
| F1 | *Prunus mume* | M009802 | Chuda, Yoshihiro; Ono, Hiroshi; Ohnishi-Kameyama, Mayumi; Matsumoto, Kousai; Nagata, Tadahiro; Kikuchi, Yuji; Journal of Agricultural and Food Chemistry; vol. 47; 3; (1999); p. 828 - 831. |
| F1 | *Prunus mume* | M010258 | Nakamura, Seikou; Fujimoto, Katsuyoshi; Matsumoto, Takahiro; Nakashima, Souichi; Ohta, Tomoe; Ogawa, Keiko; Matsuda, Hisashi; Yoshikawa, Masayuki; Phytochemistry; vol. 92; (2013); p. 128 - 136. |
| F1 | *Prunus mume* | M011005 | Miyazawa, Mitsuo; Utsunomiya, Hirotoshi; Inada, Ken-Ichi; Yamada, Tomoki; Okuno, Yoshiharu; Tanaka, Harunari; Tatematsu, Masae; Biological and Pharmaceutical Bulletin; vol. 29; 1; (2006); p. 172 - 173. |
| F1 | *Prunus mume* | M011881 | Ichikawa; Kinoshita; Sankawa; Chemical and Pharmaceutical Bulletin; vol. 37; 2; (1989); p. 345 - 348. |
| F1 | *Prunus mume* | M012864 | 国家中医药管理局《中华本草》编委会. 中华本草. Vol. 1-30 上海:上海科学技术出版社, 1999 |
| F1 | *Prunus mume* | M013627 | Nakamura, Seikou; Fujimoto, Katsuyoshi; Matsumoto, Takahiro; Nakashima, Souichi; Ohta, Tomoe; Ogawa, Keiko; Matsuda, Hisashi; Yoshikawa, Masayuki; Phytochemistry; vol. 92; (2013); p. 128 - 136. |
| F1 | *Prunus mume* | M013675 | (1) 周志宏等. 中草药, 2001, 32 (6): 484. (2) Yoshikawa M, et al. JNP, 2002, 65 (8): 1151. |
| F1 | *Prunus mume* | M017931 | Nakamura, Seikou; Fujimoto, Katsuyoshi; Matsumoto, Takahiro; Nakashima, Souichi; Ohta, Tomoe; Ogawa, Keiko; Matsuda, Hisashi; Yoshikawa, Masayuki; Phytochemistry; vol. 92; (2013); p. 128 - 136. |
| F1 | *Prunus mume* | M017932 | Fujimoto, Katsuyoshi; Nakamura, Seikou; Matsumoto, Takahiro; Ohta, Tomoe; Ogawa, Keiko; Tamura, Haruka; Matsuda, Hisashi; Yoshikawa, Masayuki; Chemical and Pharmaceutical Bulletin; vol. 61; 4; (2013); p. 445 - 451. |
| F1 | *Prunus mume* | M018082 | Yan, Xi-Tao; Li, Wei; Sun, Ya-Nan; Yang, Seo-Young; Lee, Sang-Hyun; Chen, Jian-Bo; Jang, Hae-Dong; Kim, Young-Ho; Bioorganic and Medicinal Chemistry Letters; vol. 24; 5; (2014); p. 1397 - 1402. |
| F1 | *Prunus mume* | M018381 | Fujimoto, Katsuyoshi; Nakamura, Seikou; Matsumoto, Takahiro; Ohta, Tomoe; Ogawa, Keiko; Tamura, Haruka; Matsuda, Hisashi; Yoshikawa, Masayuki; Chemical and Pharmaceutical Bulletin; vol. 61; 4; (2013); p. 445 - 451. |
| F1 | *Prunus mume* | M018382 | Fujimoto, Katsuyoshi; Nakamura, Seikou; Matsumoto, Takahiro; Ohta, Tomoe; Ogawa, Keiko; Tamura, Haruka; Matsuda, Hisashi; Yoshikawa, Masayuki; Chemical and Pharmaceutical Bulletin; vol. 61; 4; (2013); p. 445 - 451. |
| F1 | *Prunus mume* | M018519 | Yan, Xi-Tao; Li, Wei; Sun, Ya-Nan; Yang, Seo-Young; Lee, Sang-Hyun; Chen, Jian-Bo; Jang, Hae-Dong; Kim, Young-Ho; Bioorganic and Medicinal Chemistry Letters; vol. 24; 5; (2014); p. 1397 - 1402. |
| F1 | *Prunus mume* | M018816 | Fujimoto, Katsuyoshi; Nakamura, Seikou; Matsumoto, Takahiro; Ohta, Tomoe; Ogawa, Keiko; Tamura, Haruka; Matsuda, Hisashi; Yoshikawa, Masayuki; Chemical and Pharmaceutical Bulletin; vol. 61; 4; (2013); p. 445 - 451. |
| F1 | *Prunus mume* | M018817 | Nakamura, Seikou; Fujimoto, Katsuyoshi; Matsumoto, Takahiro; Nakashima, Souichi; Ohta, Tomoe; Ogawa, Keiko; Matsuda, Hisashi; Yoshikawa, Masayuki; Phytochemistry; vol. 92; (2013); p. 128 - 136. |
| F1 | *Prunus mume* | M018883 | Nakamura, Seikou; Fujimoto, Katsuyoshi; Matsumoto, Takahiro; Ohta, Tomoe; Ogawa, Keiko; Tamura, Haruka; Matsuda, Hisashi; Yoshikawa, Masayuki; Journal of Natural Medicines; vol. 67; 4; (2013); p. 799 - 806. |
| F1 | *Prunus mume* | M019230 | Nakamura, Seikou; Fujimoto, Katsuyoshi; Matsumoto, Takahiro; Nakashima, Souichi; Ohta, Tomoe; Ogawa, Keiko; Matsuda, Hisashi; Yoshikawa, Masayuki; Phytochemistry; vol. 92; (2013); p. 128 - 136. |
| F1 | *Prunus mume* | M019288 | Nakamura, Seikou; Fujimoto, Katsuyoshi; Matsumoto, Takahiro; Nakashima, Souichi; Ohta, Tomoe; Ogawa, Keiko; Matsuda, Hisashi; Yoshikawa, Masayuki; Phytochemistry; vol. 92; (2013); p. 128 - 136. |
| F1 | *Prunus mume* | M019291 | Nakamura, Seikou; Fujimoto, Katsuyoshi; Matsumoto, Takahiro; Nakashima, Souichi; Ohta, Tomoe; Ogawa, Keiko; Matsuda, Hisashi; Yoshikawa, Masayuki; Phytochemistry; vol. 92; (2013); p. 128 - 136. |
| F1 | *Prunus mume* | M019657 | Fujimoto, Katsuyoshi; Nakamura, Seikou; Matsumoto, Takahiro; Ohta, Tomoe; Ogawa, Keiko; Tamura, Haruka; Matsuda, Hisashi; Yoshikawa, Masayuki; Chemical and Pharmaceutical Bulletin; vol. 61; 4; (2013); p. 445 - 451. |
| F1 | *Prunus mume* | M019707 | Nakamura, Seikou; Fujimoto, Katsuyoshi; Matsumoto, Takahiro; Ohta, Tomoe; Ogawa, Keiko; Tamura, Haruka; Matsuda, Hisashi; Yoshikawa, Masayuki; Journal of Natural Medicines; vol. 67; 4; (2013); p. 799 - 806. |
| F1 | *Prunus mume* | M019711 | Nakamura, Seikou; Fujimoto, Katsuyoshi; Matsumoto, Takahiro; Nakashima, Souichi; Ohta, Tomoe; Ogawa, Keiko; Matsuda, Hisashi; Yoshikawa, Masayuki; Phytochemistry; vol. 92; (2013); p. 128 - 136. |
| F1 | *Prunus mume* | M019851 | (1) 阴健等. 中药现代研究与临床应用(1). 北京: 学苑出版社, 1993. (2) 贾世山等. 药学学报, 1986, 21 (6): 441. (3) YOSHIKAWA M, et al. Chem Pharm Bull, 2001, 49 (7): 863. (4) 陈嬿等. 中草药, 1990, 21 (2): 2. (5) Seetharaman TR, et al. J Indian Chem Soc, 1996, 73 (9): 499. (6) Pietta P, et al. J Chromatogr, 1992, 593 (1-2): 165. (7) FURUSAWA M, et al. Chem Pharm Bull, 2005, 53 (5): 591. (8) Yoshikawa M, et al. JNP, 2002, 65 (8): 1151. |
| F1 | *Prunus mume* | M020056 | Nakamura, Seikou; Fujimoto, Katsuyoshi; Matsumoto, Takahiro; Nakashima, Souichi; Ohta, Tomoe; Ogawa, Keiko; Matsuda, Hisashi; Yoshikawa, Masayuki; Phytochemistry; vol. 92; (2013); p. 128 - 136. |
| F1 | *Prunus mume* | M020124 | Nakamura, Seikou; Fujimoto, Katsuyoshi; Matsumoto, Takahiro; Ohta, Tomoe; Ogawa, Keiko; Tamura, Haruka; Matsuda, Hisashi; Yoshikawa, Masayuki; Journal of Natural Medicines; vol. 67; 4; (2013); p. 799 - 806. |
| F1 | *Prunus mume* | M020129 | Nakamura, Seikou; Fujimoto, Katsuyoshi; Matsumoto, Takahiro; Nakashima, Souichi; Ohta, Tomoe; Ogawa, Keiko; Matsuda, Hisashi; Yoshikawa, Masayuki; Phytochemistry; vol. 92; (2013); p. 128 - 136. |
| F1 | *Prunus mume* | M020460 | Fujimoto, Katsuyoshi; Nakamura, Seikou; Matsumoto, Takahiro; Ohta, Tomoe; Ogawa, Keiko; Tamura, Haruka; Matsuda, Hisashi; Yoshikawa, Masayuki; Chemical and Pharmaceutical Bulletin; vol. 61; 4; (2013); p. 445 - 451. |
| F1 | *Prunus mume* | M020516 | Nakamura, Seikou; Fujimoto, Katsuyoshi; Matsumoto, Takahiro; Ohta, Tomoe; Ogawa, Keiko; Tamura, Haruka; Matsuda, Hisashi; Yoshikawa, Masayuki; Journal of Natural Medicines; vol. 67; 4; (2013); p. 799 - 806. |
| F1 | *Prunus mume* | M020519 | Nakamura, Seikou; Fujimoto, Katsuyoshi; Matsumoto, Takahiro; Nakashima, Souichi; Ohta, Tomoe; Ogawa, Keiko; Matsuda, Hisashi; Yoshikawa, Masayuki; Phytochemistry; vol. 92; (2013); p. 128 - 136. |
| F1 | *Prunus mume* | M020874 | Nakamura, Seikou; Fujimoto, Katsuyoshi; Matsumoto, Takahiro; Nakashima, Souichi; Ohta, Tomoe; Ogawa, Keiko; Matsuda, Hisashi; Yoshikawa, Masayuki; Phytochemistry; vol. 92; (2013); p. 128 - 136. |
| F1 | *Prunus mume* | M020875 | Fujimoto, Katsuyoshi; Nakamura, Seikou; Matsumoto, Takahiro; Ohta, Tomoe; Ogawa, Keiko; Tamura, Haruka; Matsuda, Hisashi; Yoshikawa, Masayuki; Chemical and Pharmaceutical Bulletin; vol. 61; 4; (2013); p. 445 - 451. |
| F1 | *Prunus mume* | M020943 | Nakamura, Seikou; Fujimoto, Katsuyoshi; Matsumoto, Takahiro; Ohta, Tomoe; Ogawa, Keiko; Tamura, Haruka; Matsuda, Hisashi; Yoshikawa, Masayuki; Journal of Natural Medicines; vol. 67; 4; (2013); p. 799 - 806. |
| F1 | *Prunus mume* | M021331 | Fujimoto, Katsuyoshi; Nakamura, Seikou; Matsumoto, Takahiro; Ohta, Tomoe; Ogawa, Keiko; Tamura, Haruka; Matsuda, Hisashi; Yoshikawa, Masayuki; Chemical and Pharmaceutical Bulletin; vol. 61; 4; (2013); p. 445 - 451. |
| F1 | *Prunus mume* | M021332 | Fujimoto, Katsuyoshi; Nakamura, Seikou; Matsumoto, Takahiro; Ohta, Tomoe; Ogawa, Keiko; Tamura, Haruka; Matsuda, Hisashi; Yoshikawa, Masayuki; Chemical and Pharmaceutical Bulletin; vol. 61; 4; (2013); p. 445 - 451. |
| F1 | *Prunus mume* | M021773 | Fujimoto, Katsuyoshi; Nakamura, Seikou; Matsumoto, Takahiro; Ohta, Tomoe; Ogawa, Keiko; Tamura, Haruka; Matsuda, Hisashi; Yoshikawa, Masayuki; Chemical and Pharmaceutical Bulletin; vol. 61; 4; (2013); p. 445 - 451. |
| F1 | *Prunus mume* | M021774 | Fujimoto, Katsuyoshi; Nakamura, Seikou; Matsumoto, Takahiro; Ohta, Tomoe; Ogawa, Keiko; Tamura, Haruka; Matsuda, Hisashi; Yoshikawa, Masayuki; Chemical and Pharmaceutical Bulletin; vol. 61; 4; (2013); p. 445 - 451. |
| F1 | *Prunus mume* | M021915 | Yan, Xi-Tao; Li, Wei; Sun, Ya-Nan; Yang, Seo-Young; Lee, Sang-Hyun; Chen, Jian-Bo; Jang, Hae-Dong; Kim, Young-Ho; Bioorganic and Medicinal Chemistry Letters; vol. 24; 5; (2014); p. 1397 - 1402. |
| F1 | *Prunus mume* | M022346 | Jong, Tae Jeong; Moon, Jae-Hak; Park, Keun-Hyung; Chul, Soo Shin; Journal of Agricultural and Food Chemistry; vol. 54; 6; (2006); p. 2123 - 2128. |
| F1 | *Prunus mume* | M022532 | (1) 江苏新医学院. 中药大辞典. 上海: 上海科学技术出版社, 1977. (2) 孙文基等. 天然活性成分简明手册. 北京: 中国医药科技出版社, 1998. (3) Konishi T, et al. Chem Pharm Bull, 2005, 53 (1): 121. (4) Block S, et al. Phytochemistry, 2004, 65, 1165. (5) Yoshikawa M, et al. JNP, 2002, 65 (8): 1151. |
| F1 | *Prunus mume* | M022709 | Nakamura, Seikou; Fujimoto, Katsuyoshi; Matsumoto, Takahiro; Nakashima, Souichi; Ohta, Tomoe; Ogawa, Keiko; Matsuda, Hisashi; Yoshikawa, Masayuki; Phytochemistry; vol. 92; (2013); p. 128 - 136. |
| F1 | *Prunus mume* | M022821 | Hasegawa; Journal of Organic Chemistry; vol. 24; (1959); p. 408. |
| F1 | *Prunus mume* | M023567 | Hasegawa; Journal of Organic Chemistry; vol. 24; (1959); p. 408. |
| F1 | *Prunus mume* | M025565 | (1) 江苏新医学院. 中药大辞典. 上海: 上海科学技术出版社, 1977. (2) 国家中医药管理局《中华本草》编委会. 中华本草. Vol. 1-30 上海:上海科学技术出版社, 1999. |
| F1 | *Prunus mume* | M026260 | (1) 季宇彬等. 中药抗肿瘤有效成分药理与应用. 哈尔滨: 黑龙江科学技术出版社, 1995. (2) 江苏新医学院. 中药大辞典. 上海: 上海科学技术出版社, 1977. (3) 魏均娴等. 中国中药杂志, 1997, 22 (4): 228. (4) 尚明英�� 中国中药杂志, 1998, 23 (10): 614. (5) 孙文基等. 天然活性成分简明手册. 北京: 中国医药科技出版社, 1998. (6) 国家中医药管理局《中华本草》编委会. 中华本草. Vol. 1-30 上海:上海科学技术出版社, 1999. (7) Hou AJ, et al. JNP, 2001, 64 (1): 65. (8) Lee D, et al. JNP, 2001, 64 (10): 1286. (9) Danelutte AP, et al. Phytochemistry, 2003, 64, 555. (10) ZHANG Y-J, et al. Chem Pharm Bull, 2002, 50 (6): 841. (11) Calixto JB, et al. Planta Med, 2003, 69 (11): 973. (12) Lin JH, et al. JNP, 2002, 65 (5): 638. (13) Chiang YM, et al. JNP, 2003, 66 (8): 1070. (14) Park S-H, et al. Planta Med, 2005, 71 (1): 24. |
| F1 | *Prunus mume* | M026376 | (1) HUA H, et al. Chem Pharm Bull, 2002, 50 (10): 1393. (2) Yoshikawa M, et al. JNP, 2002, 65 (8): 1151. |
| F1 | *Prunus mume* | M026813 | Yoshikawa M, et al. JNP, 2002, 65 (8): 1151 |
| F1 | *Prunus mume* | M027597 | Yan, Xi-Tao; Li, Wei; Sun, Ya-Nan; Yang, Seo-Young; Lee, Sang-Hyun; Chen, Jian-Bo; Jang, Hae-Dong; Kim, Young-Ho; Bioorganic and Medicinal Chemistry Letters; vol. 24; 5; (2014); p. 1397 - 1402. |
| F1 | *Prunus mume* | M028311 | (1) Itoh A, et al. JNP, 2004, 67 (3): 427. (2) Elliger CA, et al. Chem. Abstr., 1992, 117, 66647g. (3) 黄先荣等. 植物学报, 1981, 23 (3): 222. (4) Yoshikawa M, et al. JNP, 2002, 65 (8): 1151. |
| F1 | *Prunus mume* | M030683 | Fujimoto, Katsuyoshi; Nakamura, Seikou; Matsumoto, Takahiro; Ohta, Tomoe; Ogawa, Keiko; Tamura, Haruka; Matsuda, Hisashi; Yoshikawa, Masayuki; Chemical and Pharmaceutical Bulletin; vol. 61; 4; (2013); p. 445 - 451. |
| F1 | *Prunus mume* | M031197 | Yoshikawa M, et al. JNP, 2002, 65 (8): 1151 |
| F1 | *Prunus mume* | M031894 | Yoshikawa M, et al. JNP, 2002, 65 (8): 1151 |
| F1 | *Prunus mume* | M032088 | Hasegawa; Journal of Organic Chemistry; vol. 24; (1959); p. 408. |
| F1 | *Prunus mume* | M033080 | Yoshikawa M, et al. JNP, 2002, 65 (8): 1151 |
| F1 | *Prunus mume* | M034019 | Matsuda, Hisashi; Morikawa, Toshio; Ishiwada, Tomoko; Managi, Hiromi; Kagawa, Masatomo; Higashi, Yoshihiko; Yoshikawa, Masayuki; Chemical and pharmaceutical bulletin; vol. 51; 4; (2003); p. 440 - 443. |
| F1 | *Prunus mume* | M034058 | Nakamura, Seikou; Fujimoto, Katsuyoshi; Matsumoto, Takahiro; Nakashima, Souichi; Ohta, Tomoe; Ogawa, Keiko; Matsuda, Hisashi; Yoshikawa, Masayuki; Phytochemistry; vol. 92; (2013); p. 128 - 136. |
| F1 | *Salvia miltiorrhiza* | M000147 | Zhang, De-Wu; Liu, Xiao; Xie, Dan; Chen, Ridao; Tao, Xiao-Yu; Zou, Jian-Hua; Dai, Jungui; Chemical and Pharmaceutical Bulletin; vol. 61; 5; (2013); p. 576 - 580. |
| F1 | *Salvia miltiorrhiza* | M000201 | Nguyen, Tien Dat; Jin, Xuejun; Lee, Jeong-Hyung; Lee, Dongho; Hong, Young-Soo; Lee, Kyeong; Young, Ho Kim; Jung, Joon Lee; Journal of Natural Products; vol. 70; 7; (2007); p. 1093 - 1097. |
| F1 | *Salvia miltiorrhiza* | M000327 | 国家中医药管理局《中华本草》编委会. 中华本草. Vol. 1-30 上海:上海科学技术出版社, 1999 |
| F1 | *Salvia miltiorrhiza* | M000831 | v. Wessely; Lauterbach; Chemische Berichte; vol. 75; (1942); p. 958,967.; Nakao; Fukushima; Yakugaku Zasshi; vol. 54; (1934); p. engl. Ref. S. 154, 156. |
| F1 | *Salvia miltiorrhiza* | M000897 | Yan, Xijun; Wu, Naifeng; Guo, Zhixin; Ye, Zhengliang; Liu, Yan; US2005/37094; A1; (2005). |
| F1 | *Salvia miltiorrhiza* | M001169 | Chang, Hson Mou; Cheng, Kwok Ping; Choang, Tai Francis; Chow, Hak Fun; Chui, Kuk Ying; et al.; Journal of Organic Chemistry; vol. 55; 11; (1990); p. 3537 - 3543. |
| F1 | *Salvia miltiorrhiza* | M001191 | Yan, Xijun; Wu, Naifeng; Guo, Zhixin; Ye, Zhengliang; Liu, Yan; US2005/37094; A1; (2005). |
| F1 | *Salvia miltiorrhiza* | M001245 | Zhang, De-Wu; Liu, Xiao; Xie, Dan; Chen, Ridao; Tao, Xiao-Yu; Zou, Jian-Hua; Dai, Jungui; Chemical and Pharmaceutical Bulletin; vol. 61; 5; (2013); p. 576 - 580. |
| F1 | *Salvia miltiorrhiza* | M001344 | Ryu, Shi Yong; No, Zaesung; Kim, Sung Hoon; Ahn, Jong Woong; Planta Medica; vol. 63; 1; (1997); p. 44 - 46. |
| F1 | *Salvia miltiorrhiza* | M001572 | Chang, Hson Mou; Cheng, Kwok Ping; Choang, Tai Francis; Chow, Hak Fun; Chui, Kuk Ying; et al.; Journal of Organic Chemistry; vol. 55; 11; (1990); p. 3537 - 3543. |
| F1 | *Salvia miltiorrhiza* | M001856 | Ikeshiro, Yasumasa; Mase, Izumi; Tomita, Yutaka; Phytochemistry (Elsevier); vol. 28; 11; (1989); p. 3139 - 3142. |
| F1 | *Salvia miltiorrhiza* | M002139 | Lia, Xiao-Jun; Tang, Hao-Yu; Duan, Jia-Li; Gao, Jin-Ming; Xue, Quan-Hong; Natural Product Research; vol. 27; 4-5; (2013); p. 496 - 499. |
| F1 | *Salvia miltiorrhiza* | M002167 | Wang, Xihong; Bastow, Kenneth F.; Sun, Chang-Ming; Lin, Yun-Lian; Yu, Hsi-Jung; Don, Ming-Jaw; Wu, Tian-Shung; Nakamura, Seikou; Lee, Kuo-Hsiung; Journal of Medicinal Chemistry; vol. 47; 23; (2004); p. 5816 - 5819. |
| F1 | *Salvia miltiorrhiza* | M002638 | Ryu, Shi Yong; Lee, Chong Ock; Choi, Sang Un; Planta Medica; vol. 63; 4; (1997); p. 339 - 342. |
| F1 | *Salvia miltiorrhiza* | M003142 | Ginda, Haro; Kusumi, Takenori; Ishitsuka, Midori O.; Kakisawa, Hiroshi; Weijie, Zhao; et al.; Tetrahedron Letters; vol. 29; 36; (1988); p. 4603 - 4606. |
| F1 | *Salvia miltiorrhiza* | M003155 | Haro, Ginda; Kakisawa, Hiroshi; Chemistry Letters; 9; (1990); p. 1599 - 1602. |
| F1 | *Salvia miltiorrhiza* | M003790 | Kohda; Takeda; Tanaka; Yamasaki; Yamashita; Kurokawa; Ishibashi; Chemical and Pharmaceutical Bulletin; vol. 37; 5; (1989); p. 1287 - 1290. |
| F1 | *Salvia miltiorrhiza* | M003864 | Asari, Fumika; Kusumi, Takenori; Zheng, Guo-Zhi; Cen, Ying-Zhou; Kakisawa, Hiroshi; Chemistry Letters; 10; (1990); p. 1885 - 1888. |
| F1 | *Salvia miltiorrhiza* | M004020 | Don, Ming-Jaw; Shen, Chien-Chang; Syu, Wan-Jr; Ding, Yi-Huei; Sun, Chang-Ming; Phytochemistry; vol. 67; 5; (2006); p. 497 - 503. |
| F1 | *Salvia miltiorrhiza* | M004272 | Lay IS, et al. Planta Med, 2003, 69, 26 |
| F1 | *Salvia miltiorrhiza* | M004333 | (1) Buckingham J(Executive Editor): et al. Dictionary of Natural Products, Vol 1-7, Chapman & Hall, London, 1994; 1995, Vol 8; 1996, Vol 9; 1997, Vol 10; 1998, Vol 11.. (2) XU G, et al. Chem Pharm Bull, 2005, 53 (12): 1575. |
| F1 | *Salvia miltiorrhiza* | M004374 | Ryu, Shi Yong; Lee, Chong Ock; Choi, Sang Un; Planta Medica; vol. 63; 4; (1997); p. 339 - 342. |
| F1 | *Salvia miltiorrhiza* | M004983 | Don, Ming-Jaw; Shen, Chien-Chang; Syu, Wan-Jr; Ding, Yi-Huei; Sun, Chang-Ming; Phytochemistry; vol. 67; 5; (2006); p. 497 - 503. |
| F1 | *Salvia miltiorrhiza* | M005058 | Lia, Xiao-Jun; Tang, Hao-Yu; Duan, Jia-Li; Gao, Jin-Ming; Xue, Quan-Hong; Natural Product Research; vol. 27; 4-5; (2013); p. 496 - 499. |
| F1 | *Salvia miltiorrhiza* | M005137 | Ryu, Shi Yong; Lee, Chong Ock; Choi, Sang Un; Planta Medica; vol. 63; 4; (1997); p. 339 - 342. |
| F1 | *Salvia miltiorrhiza* | M005193 | Luo, Hou-Wei; Wu, Bao-Jing; Wu, Mei-Yu; Yong, Zhong-Gen; Niwa, Masatake; Hirata, Yoshimasa; Phytochemistry (Elsevier); vol. 24; 4; (1985); p. 815 - 818. |
| F1 | *Salvia miltiorrhiza* | M005571 | Ikeshiro, Yasumasa; Mase, Izumi; Tomita, Yutaka; Phytochemistry (Elsevier); vol. 28; 11; (1989); p. 3139 - 3142. |
| F1 | *Salvia miltiorrhiza* | M005610 | Nguyen, Tien Dat; Jin, Xuejun; Lee, Jeong-Hyung; Lee, Dongho; Hong, Young-Soo; Lee, Kyeong; Young, Ho Kim; Jung, Joon Lee; Journal of Natural Products; vol. 70; 7; (2007); p. 1093 - 1097. |
| F1 | *Salvia miltiorrhiza* | M006014 | Kohda; Takeda; Tanaka; Yamasaki; Yamashita; Kurokawa; Ishibashi; Chemical and Pharmaceutical Bulletin; vol. 37; 5; (1989); p. 1287 - 1290. |
| F1 | *Salvia miltiorrhiza* | M006364 | (1) 阴健等. 中药现代研究与临床应用(1). 北京: 学苑出版社, 1993. (2) 江苏新医学院. 中药大辞典. 上海: 上海科学技术出版社, 1977. (3) 白银娟等. 中国药学杂志, 1997, 32 (8): 462. (4) 国家中医药管理局《中华本草》编委会. 中华本草. Vol. 1-30 上海:上海科学技术出版社, 1999. (5) Buckingham J(Executive Editor): et al. Dictionary of Natural Products, Vol 1-7, Chapman & Hall, London, 1994; 1995, Vol 8; 1996, Vol 9; 1997, Vol 10; 1998, Vol 11.. (6) 欧明等. 简明中药成�质植� 北京: 中国医药科技出版社, 2003. |
| F1 | *Salvia miltiorrhiza* | M006365 | Don, Ming-Jaw; Shen, Chien-Chang; Syu, Wan-Jr; Ding, Yi-Huei; Sun, Chang-Ming; Phytochemistry; vol. 67; 5; (2006); p. 497 - 503. |
| F1 | *Salvia miltiorrhiza* | M006699 | Yao, Fang; Zhang, De-Wu; Qu, Gui-Wu; Li, Gui-Sheng; Dai, Sheng Jun; Journal of Asian Natural Products Research; vol. 14; 9; (2012); p. 913 - 917,5. |
| F1 | *Salvia miltiorrhiza* | M006936 | ScinoPharm Taiwan Ltd.; US2010/48618; A1; (2010). |
| F1 | *Salvia miltiorrhiza* | M007104 | Yan, Xijun; Wu, Naifeng; Guo, Zhixin; Ye, Zhengliang; Liu, Yan; US2005/37094; A1; (2005). |
| F1 | *Salvia miltiorrhiza* | M007281 | Liu, Pu; Hu, Pan; Deng, Rui-Xue; Li, Ru; Yang, Li; Yin, Wei-Ping; Helvetica Chimica Acta; vol. 94; 1; (2011); p. 136 - 141. |
| F1 | *Salvia miltiorrhiza* | M007380 | (1) 阴健等. 中药现代研究与临床应用(1). 北京: 学苑出版社, 1993. (2) 孙文基等. 天然活性成分简明手册. 北京: 中国医药科技出版社, 1998. (3) 国家中医药管理局《中华本草》编委会. 中华本草. Vol. 1-30 上海:上海科学技术出版社, 1999. (4) Buckingham J(Executive Editor): et al. Dictionary of Natural Products, Vol 1-7, Chapman & Hall, London, 1994; 1995, Vol 8; 1996, Vol 9; 1997, Vol 10; 1998, Vol 11.. (5) Kitajima J, et al. Chem Pharm Bull, 2004, 52 (8): 1013. (6) CHOUDHARY MI, et al. Chem Pharm Bull, 2005, 53 (11): 1469. (7) Lin YL, et al. JNP, 2002, 65 (5): 745. (8) Janicsák G, et al. Planta Med, 2003, 69, 1156. |
| F1 | *Salvia miltiorrhiza* | M007637 | Asari, Fumika; Kusumi, Takenori; Zheng, Guo-Zhi; Cen, Ying-Zhou; Kakisawa, Hiroshi; Chemistry Letters; 10; (1990); p. 1885 - 1888. |
| F1 | *Salvia miltiorrhiza* | M007794 | Lia, Xiao-Jun; Tang, Hao-Yu; Duan, Jia-Li; Gao, Jin-Ming; Xue, Quan-Hong; Natural Product Research; vol. 27; 4-5; (2013); p. 496 - 499. |
| F1 | *Salvia miltiorrhiza* | M008326 | Lia, Xiao-Jun; Tang, Hao-Yu; Duan, Jia-Li; Gao, Jin-Ming; Xue, Quan-Hong; Natural Product Research; vol. 27; 4-5; (2013); p. 496 - 499. |
| F1 | *Salvia miltiorrhiza* | M008377 | Takiura; Yakugaku Zasshi; vol. 61; (1941); p. 475,481.; Takiura; Koizumi; Chemical and Pharmaceutical Bulletin; vol. 10; (1962); p. 112,114. |
| F1 | *Salvia miltiorrhiza* | M008384 | Luo, Hou-Wei; Wu, Bao-Jing; Wu, Mei-Yu; Yong, Zhong-Gen; Niwa, Masatake; Hirata, Yoshimasa; Phytochemistry (Elsevier); vol. 24; 4; (1985); p. 815 - 818. |
| F1 | *Salvia miltiorrhiza* | M008601 | 陈蕙芳等. 植物活性成分辞典. 第1册. 北京: 中国医药科技出版社, 2001 |
| F1 | *Salvia miltiorrhiza* | M009024 | (1) 孙文基等. 天然活性成分简明手册. 北京: 中国医药科技出版社, 1998. (2) Fang C等. 化学学报, 1976, 34, 197. |
| F1 | *Salvia miltiorrhiza* | M009353 | Zhang, De-Wu; Liu, Xiao; Xie, Dan; Chen, Ridao; Tao, Xiao-Yu; Zou, Jian-Hua; Dai, Jungui; Chemical and Pharmaceutical Bulletin; vol. 61; 5; (2013); p. 576 - 580. |
| F1 | *Salvia miltiorrhiza* | M009494 | 罗厚蔚等. 药学学报, 1988, 23 (11): 830 |
| F1 | *Salvia miltiorrhiza* | M009540 | Lin, Hang-Ching; Chang, Wen-Liang; Phytochemistry; vol. 53; 8; (2000); p. 951 - 953. |
| F1 | *Salvia miltiorrhiza* | M009812 | Lin, Hang-Ching; Ding, Hsiou-Yu; Chang, Wen-Liang; Journal of Natural Products; vol. 64; 5; (2001); p. 648 - 650. |
| F1 | *Salvia miltiorrhiza* | M009912 | Don, Ming-Jaw; Shen, Chien-Chang; Syu, Wan-Jr; Ding, Yi-Huei; Sun, Chang-Ming; Phytochemistry; vol. 67; 5; (2006); p. 497 - 503. |
| F1 | *Salvia miltiorrhiza* | M009987 | Chang, Hson Mou; Choang, Tai Francis; Chui, Kuk Ying; Hon, Po Ming; Lee, Chi Ming; et al.; Journal of Chemical Research, Miniprint; 4; (1990); p. 877 - 886. |
| F1 | *Salvia miltiorrhiza* | M010192 | Lia, Xiao-Jun; Tang, Hao-Yu; Duan, Jia-Li; Gao, Jin-Ming; Xue, Quan-Hong; Natural Product Research; vol. 27; 4-5; (2013); p. 496 - 499. |
| F1 | *Salvia miltiorrhiza* | M010269 | Don, Ming-Jaw; Shen, Chien-Chang; Lin, Yun-Lian; Syu, Wan-Jr; Ding, Yi-Huei; Sun, Chang-Ming; Journal of Natural Products; vol. 68; 7; (2005); p. 1066 - 1070. |
| F1 | *Salvia miltiorrhiza* | M010402 | Chang, Hson Mou; Cheng, Kwok Ping; Choang, Tai Francis; Chow, Hak Fun; Chui, Kuk Ying; et al.; Journal of Organic Chemistry; vol. 55; 11; (1990); p. 3537 - 3543. |
| F1 | *Salvia miltiorrhiza* | M010422 | Zhang, De-Wu; Liu, Xiao; Xie, Dan; Chen, Ridao; Tao, Xiao-Yu; Zou, Jian-Hua; Dai, Jungui; Chemical and Pharmaceutical Bulletin; vol. 61; 5; (2013); p. 576 - 580. |
| F1 | *Salvia miltiorrhiza* | M010426 | Don, Ming-Jaw; Shen, Chien-Chang; Syu, Wan-Jr; Ding, Yi-Huei; Sun, Chang-Ming; Phytochemistry; vol. 67; 5; (2006); p. 497 - 503. |
| F1 | *Salvia miltiorrhiza* | M010548 | Wang, Dandan; Girard, Thomas J.; Kasten, Thomas P.; LaChance, Rhonda M.; Miller-Wideman, Margaret A.; Durley, Richard C.; Journal of Natural Products; vol. 61; 11; (1998); p. 1352 - 1355. |
| F1 | *Salvia miltiorrhiza* | M010675 | Nguyen, Tien Dat; Jin, Xuejun; Lee, Jeong-Hyung; Lee, Dongho; Hong, Young-Soo; Lee, Kyeong; Young, Ho Kim; Jung, Joon Lee; Journal of Natural Products; vol. 70; 7; (2007); p. 1093 - 1097. |
| F1 | *Salvia miltiorrhiza* | M010890 | Don, Ming-Jaw; Shen, Chien-Chang; Lin, Yun-Lian; Syu, Wan-Jr; Ding, Yi-Huei; Sun, Chang-Ming; Journal of Natural Products; vol. 68; 7; (2005); p. 1066 - 1070. |
| F1 | *Salvia miltiorrhiza* | M011304 | Retrieved from CNPD |
| F1 | *Salvia miltiorrhiza* | M011373 | Chang, Hson Mou; Choang, Tai Francis; Chui, Kuk Ying; Hon, Po Ming; Lee, Chi Ming; et al.; Journal of Chemical Research, Miniprint; 4; (1990); p. 877 - 886. |
| F1 | *Salvia miltiorrhiza* | M011546 | Lin, Hang-Ching; Chang, Wen-Liang; Phytochemistry; vol. 53; 8; (2000); p. 951 - 953. |
| F1 | *Salvia miltiorrhiza* | M011637 | Young, Jeffrey; US2006/83798; A1; (2006). |
| F1 | *Salvia miltiorrhiza* | M011774 | Nguyen, Tien Dat; Jin, Xuejun; Lee, Jeong-Hyung; Lee, Dongho; Hong, Young-Soo; Lee, Kyeong; Young, Ho Kim; Jung, Joon Lee; Journal of Natural Products; vol. 70; 7; (2007); p. 1093 - 1097. |
| F1 | *Salvia miltiorrhiza* | M011785 | Haro; Mori; Ishitsuka; Kusumi; Inouye; Kakisawa; Bulletin of the Chemical Society of Japan; vol. 64; 11; (1991); p. 3422 - 3426. |
| F1 | *Salvia miltiorrhiza* | M012240 | Nguyen, Tien Dat; Jin, Xuejun; Lee, Jeong-Hyung; Lee, Dongho; Hong, Young-Soo; Lee, Kyeong; Young, Ho Kim; Jung, Joon Lee; Journal of Natural Products; vol. 70; 7; (2007); p. 1093 - 1097. |
| F1 | *Salvia miltiorrhiza* | M012302 | 国家中医药管理局《中华本草》编委会. 中华本草. Vol. 1-30 上海:上海科学技术出版社, 1999 |
| F1 | *Salvia miltiorrhiza* | M012410 | Zhang, De-Wu; Liu, Xiao; Xie, Dan; Chen, Ridao; Tao, Xiao-Yu; Zou, Jian-Hua; Dai, Jungui; Chemical and Pharmaceutical Bulletin; vol. 61; 5; (2013); p. 576 - 580. |
| F1 | *Salvia miltiorrhiza* | M012606 | Lia, Xiao-Jun; Tang, Hao-Yu; Duan, Jia-Li; Gao, Jin-Ming; Xue, Quan-Hong; Natural Product Research; vol. 27; 4-5; (2013); p. 496 - 499. |
| F1 | *Salvia miltiorrhiza* | M012765 | Liu, Pu; Hu, Pan; Deng, Rui-Xue; Li, Ru; Yang, Li; Yin, Wei-Ping; Helvetica Chimica Acta; vol. 94; 1; (2011); p. 136 - 141. |
| F1 | *Salvia miltiorrhiza* | M012931 | Retrieved from CNPD |
| F1 | *Salvia miltiorrhiza* | M013118 | Onitsuka; Fujiu; Shinma; Maruyama; Chemical and Pharmaceutical Bulletin; vol. 31; 5; (1983); p. 1670 - 1675. |
| F1 | *Salvia miltiorrhiza* | M013119 | Asari, Fumika; Kusumi, Takenori; Zheng, Guo-Zhi; Cen, Ying-Zhou; Kakisawa, Hiroshi; Chemistry Letters; 10; (1990); p. 1885 - 1888. |
| F1 | *Salvia miltiorrhiza* | M013342 | Ryu, Shi Yong; Lee, Chong Ock; Choi, Sang Un; Planta Medica; vol. 63; 4; (1997); p. 339 - 342. |
| F1 | *Salvia miltiorrhiza* | M013432 | Haro; Mori; Ishitsuka; Kusumi; Inouye; Kakisawa; Bulletin of the Chemical Society of Japan; vol. 64; 11; (1991); p. 3422 - 3426. |
| F1 | *Salvia miltiorrhiza* | M013705 | 国家中医药管理局《中华本草》编委会. 中华本草. Vol. 1-30 上海:上海科学技术出版社, 1999 |
| F1 | *Salvia miltiorrhiza* | M013792 | Han, Yu Mi; Oh, Hyuncheol; Na, MinKyun; Kim, Beom Seok; Oh, Won Keun; Kim, Bo Yeon; Jeong, Dae Gwin; Ryu, Seong Eon; Sok, Dai-Eun; Ahn, Jong Seog; Biological and Pharmaceutical Bulletin; vol. 28; 9; (2005); p. 1795 - 1797. |
| F1 | *Salvia miltiorrhiza* | M013935 | Luo, Hou-Wei; Wu, Bao-Jing; Wu, Mei-Yu; Yong, Zhong-Gen; Niwa, Masatake; Hirata, Yoshimasa; Phytochemistry (Elsevier); vol. 24; 4; (1985); p. 815 - 818. |
| F1 | *Salvia miltiorrhiza* | M013945 | BIZMEDIC CO., LTD.; US2006/8541; A1; (2006). |
| F1 | *Salvia miltiorrhiza* | M014183 | Lusarczyk, Sylwester; Zimmermann, Stefanie; Kaiser, Marcel; Matkowski, Adam; Hamburger, Matthias; Adams, Michael; Planta Medica; vol. 77; 14; (2011); p. 1594 - 1596. |
| F1 | *Salvia miltiorrhiza* | M014228 | Kohda; Takeda; Tanaka; Yamasaki; Yamashita; Kurokawa; Ishibashi; Chemical and Pharmaceutical Bulletin; vol. 37; 5; (1989); p. 1287 - 1290. |
| F1 | *Salvia miltiorrhiza* | M014437 | Zhang, De-Wu; Liu, Xiao; Xie, Dan; Chen, Ridao; Tao, Xiao-Yu; Zou, Jian-Hua; Dai, Jungui; Chemical and Pharmaceutical Bulletin; vol. 61; 5; (2013); p. 576 - 580. |
| F1 | *Salvia miltiorrhiza* | M014522 | Hayashi et al.; Journal of the Chemical Society [Section] D: Chemical Communications; (1971); p. 541. |
| F1 | *Salvia miltiorrhiza* | M014600 | Retrieved from CNPD |
| F1 | *Salvia miltiorrhiza* | M014709 | (1) Ryu SY, et al. Planta Med, 1997, 63 (4): 339. (2) Ryu SY, et al. Planta Med, 1997, 63 (1): 44. |
| F1 | *Salvia miltiorrhiza* | M014790 | 阴健等. 中药现代研究与临床应用(1). 北京: 学苑出版社, 1993 |
| F1 | *Salvia miltiorrhiza* | M015251 | Ginda, Haro; Kusumi, Takenori; Ishitsuka, Midori O.; Kakisawa, Hiroshi; Weijie, Zhao; et al.; Tetrahedron Letters; vol. 29; 36; (1988); p. 4603 - 4606. |
| F1 | *Salvia miltiorrhiza* | M015289 | Don, Ming-Jaw; Shen, Chien-Chang; Syu, Wan-Jr; Ding, Yi-Huei; Sun, Chang-Ming; Phytochemistry; vol. 67; 5; (2006); p. 497 - 503. |
| F1 | *Salvia miltiorrhiza* | M015376 | Nguyen, Tien Dat; Jin, Xuejun; Lee, Jeong-Hyung; Lee, Dongho; Hong, Young-Soo; Lee, Kyeong; Young, Ho Kim; Jung, Joon Lee; Journal of Natural Products; vol. 70; 7; (2007); p. 1093 - 1097. |
| F1 | *Salvia miltiorrhiza* | M015377 | Haro; Mori; Ishitsuka; Kusumi; Inouye; Kakisawa; Bulletin of the Chemical Society of Japan; vol. 64; 11; (1991); p. 3422 - 3426. |
| F1 | *Salvia miltiorrhiza* | M015406 | Ryu, Shi Yong; Lee, Chong Ock; Choi, Sang Un; Planta Medica; vol. 63; 4; (1997); p. 339 - 342. |
| F1 | *Salvia miltiorrhiza* | M015561 | (1) 江苏新医学院. 中药大辞典. 上海: 上海科学技术出版社, 1977. (2) 宋振玉等. 中草药现代研究. 第2卷. 第25章 甾体原料植物. 226-254 北京: 北京医科大学中国协和医科大学联合出版社, 1996. (3) 国家中医药管理局《中华本草》编委会. 中华本草. Vol. 1-30 上海:上海科学技术出版社, 1999. (4) 张援虎等. 天然产物研究与开发, 2003, 15 (4): 349. |
| F1 | *Salvia miltiorrhiza* | M015606 | Wang, Chao-Yun; Ma, Fu-Lu; Liu, Jun-Tian; Tian, Jing-Wei; Fu, Feng-Hua; Biological and Pharmaceutical Bulletin; vol. 30; 1; (2007); p. 44 - 47. |
| F1 | *Salvia miltiorrhiza* | M017539 | Lusarczyk, Sylwester; Zimmermann, Stefanie; Kaiser, Marcel; Matkowski, Adam; Hamburger, Matthias; Adams, Michael; Planta Medica; vol. 77; 14; (2011); p. 1594 - 1596. |
| F1 | *Salvia miltiorrhiza* | M017640 | Zhang Y, et al. Planta Med, 2004, 70 (2): 138 |
| F1 | *Salvia miltiorrhiza* | M017954 | Zhang, De-Wu; Liu, Xiao; Xie, Dan; Chen, Ridao; Tao, Xiao-Yu; Zou, Jian-Hua; Dai, Jungui; Chemical and Pharmaceutical Bulletin; vol. 61; 5; (2013); p. 576 - 580. |
| F1 | *Salvia miltiorrhiza* | M018398 | Zhang, De-Wu; Liu, Xiao; Xie, Dan; Chen, Ridao; Tao, Xiao-Yu; Zou, Jian-Hua; Dai, Jungui; Chemical and Pharmaceutical Bulletin; vol. 61; 5; (2013); p. 576 - 580. |
| F1 | *Salvia miltiorrhiza* | M018832 | Zhang, De-Wu; Liu, Xiao; Xie, Dan; Chen, Ridao; Tao, Xiao-Yu; Zou, Jian-Hua; Dai, Jungui; Chemical and Pharmaceutical Bulletin; vol. 61; 5; (2013); p. 576 - 580. |
| F1 | *Salvia miltiorrhiza* | M019038 | Don, Ming-Jaw; Shen, Chien-Chang; Lin, Yun-Lian; Syu, Wan-Jr; Ding, Yi-Huei; Sun, Chang-Ming; Journal of Natural Products; vol. 68; 7; (2005); p. 1066 - 1070. |
| F1 | *Salvia miltiorrhiza* | M019440 | Choi, Jae Sue; Kang, Hye Sook; Jung, Hyun Ah; Jung, Jee H; Kang, Sam Sik; Fitoterapia; vol. 72; 1; (2001); p. 30 - 34. |
| F1 | *Salvia miltiorrhiza* | M020050 | Lia, Xiao-Jun; Tang, Hao-Yu; Duan, Jia-Li; Gao, Jin-Ming; Xue, Quan-Hong; Natural Product Research; vol. 27; 4-5; (2013); p. 496 - 499. |
| F1 | *Salvia miltiorrhiza* | M020453 | Lia, Xiao-Jun; Tang, Hao-Yu; Duan, Jia-Li; Gao, Jin-Ming; Xue, Quan-Hong; Natural Product Research; vol. 27; 4-5; (2013); p. 496 - 499. |
| F1 | *Salvia miltiorrhiza* | M020456 | Don, Ming-Jaw; Shen, Chien-Chang; Lin, Yun-Lian; Syu, Wan-Jr; Ding, Yi-Huei; Sun, Chang-Ming; Journal of Natural Products; vol. 68; 7; (2005); p. 1066 - 1070. |
| F1 | *Salvia miltiorrhiza* | M020825 | Yao, Fang; Zhang, De-Wu; Qu, Gui-Wu; Li, Gui-Sheng; Dai, Sheng Jun; Journal of Asian Natural Products Research; vol. 14; 9; (2012); p. 913 - 917,5. |
| F1 | *Salvia miltiorrhiza* | M021564 | Lin, Yun-Lian; Lee, Ting-Fang; Huang, Yeh-Jeng; Huang, Yi-Tsau; Journal of Pharmacy and Pharmacology; vol. 58; 7; (2006); p. 933 - 939. |
| F1 | *Salvia miltiorrhiza* | M021789 | Zhang, De-Wu; Liu, Xiao; Xie, Dan; Chen, Ridao; Tao, Xiao-Yu; Zou, Jian-Hua; Dai, Jungui; Chemical and Pharmaceutical Bulletin; vol. 61; 5; (2013); p. 576 - 580. |
| F1 | *Salvia miltiorrhiza* | M021934 | Haro; Mori; Ishitsuka; Kusumi; Inouye; Kakisawa; Bulletin of the Chemical Society of Japan; vol. 64; 11; (1991); p. 3422 - 3426. |
| F1 | *Salvia miltiorrhiza* | M022096 | Ryu, Shi Yong; Lee, Chong Ock; Choi, Sang Un; Planta Medica; vol. 63; 4; (1997); p. 339 - 342. |
| F1 | *Salvia miltiorrhiza* | M022257 | Han, Yu Mi; Oh, Hyuncheol; Na, MinKyun; Kim, Beom Seok; Oh, Won Keun; Kim, Bo Yeon; Jeong, Dae Gwin; Ryu, Seong Eon; Sok, Dai-Eun; Ahn, Jong Seog; Biological and Pharmaceutical Bulletin; vol. 28; 9; (2005); p. 1795 - 1797. |
| F1 | *Salvia miltiorrhiza* | M022385 | 杨保津等. 药学学报, 1981, 16 (11): 837 |
| F1 | *Salvia miltiorrhiza* | M022548 | (1) Ryu SY, et al. Planta Med, 1997, 63 (4): 339. (2) Ryu SY, et al. Planta Med, 1997, 63 (1): 44. |
| F1 | *Salvia miltiorrhiza* | M022580 | Lia, Xiao-Jun; Tang, Hao-Yu; Duan, Jia-Li; Gao, Jin-Ming; Xue, Quan-Hong; Natural Product Research; vol. 27; 4-5; (2013); p. 496 - 499. |
| F1 | *Salvia miltiorrhiza* | M023121 | Luo, Hou-Wei; Wu, Bao-Jing; Wu, Mei-Yu; Yong, Zhong-Gen; Niwa, Masatake; Hirata, Yoshimasa; Phytochemistry (Elsevier); vol. 24; 4; (1985); p. 815 - 818. |
| F1 | *Salvia miltiorrhiza* | M023255 | (1) 阴健等. 中药现代研究与临床应用(1). 北京: 学苑出版社, 1993. (2) 季宇彬等. 中药抗肿瘤有效成分药理与应用. 哈尔滨: 黑龙江科学技术出版社, 1995. (3) 季宇彬等. 中药抗肿瘤有效成分药理与应用. 哈尔滨: 黑龙�蒲Ъ际醭霭嫔� 1998. (4) 江苏新医学院. 中药大辞典. 上海: 上海科学技术出版社, 1977. (5) 孙文基等. 天然活性成分简明手册. 北京: 中国医药科技出版社, 1998. (6) 国家中医药管理局《中华本草》编委会. 中华本草. Vol. 1-30 上海:上海科学技术出版社, 1999. (7) Tsai PL, et al. Planta Med, 2004, 70, 1069. (8) Calixto JB, et al. Planta Med, 2003, 69 (11): 973. (9) Calixto JB, et al. Planta Med, 2004, 70 (1): 93. (10) Tang W, et al. Planta Med, 2003, 69, 97. (11) Cheng K-T, et al. Planta Med, 2003, 69, 300. (12) 欧明等. �蛎髦幸┏煞质植� 北京: 中国医药科技出版社, 2003. |
| F1 | *Salvia miltiorrhiza* | M023488 | Retrieved from CNPD |
| F1 | *Salvia miltiorrhiza* | M023545 | Retrieved from CNPD |
| F1 | *Salvia miltiorrhiza* | M023707 | Choi, Jae Sue; Kang, Hye Sook; Jung, Hyun Ah; Jung, Jee H; Kang, Sam Sik; Fitoterapia; vol. 72; 1; (2001); p. 30 - 34. |
| F1 | *Salvia miltiorrhiza* | M024245 | Don, Ming-Jaw; Shen, Chien-Chang; Lin, Yun-Lian; Syu, Wan-Jr; Ding, Yi-Huei; Sun, Chang-Ming; Journal of Natural Products; vol. 68; 7; (2005); p. 1066 - 1070. |
| F1 | *Salvia miltiorrhiza* | M024381 | Kohda; Takeda; Tanaka; Yamasaki; Yamashita; Kurokawa; Ishibashi; Chemical and Pharmaceutical Bulletin; vol. 37; 5; (1989); p. 1287 - 1290. |
| F1 | *Salvia miltiorrhiza* | M024453 | Lin, Hang-Ching; Ding, Hsiou-Yu; Chang, Wen-Liang; Journal of Natural Products; vol. 64; 5; (2001); p. 648 - 650. |
| F1 | *Salvia miltiorrhiza* | M024478 | Yagi; Okamura; Tanonaka; Takeo; Planta Medica; vol. 60; 5; (1994); p. 405 - 409. |
| F1 | *Salvia miltiorrhiza* | M024621 | Ikeshiro, Yasumasa; Mase, Izumi; Tomita, Yutaka; Phytochemistry (Elsevier); vol. 28; 11; (1989); p. 3139 - 3142. |
| F1 | *Salvia miltiorrhiza* | M025058 | Yan, Xijun; Wu, Naifeng; Guo, Zhixin; Ye, Zhengliang; Liu, Yan; US2005/37094; A1; (2005). |
| F1 | *Salvia miltiorrhiza* | M025749 | Lia, Xiao-Jun; Tang, Hao-Yu; Duan, Jia-Li; Gao, Jin-Ming; Xue, Quan-Hong; Natural Product Research; vol. 27; 4-5; (2013); p. 496 - 499. |
| F1 | *Salvia miltiorrhiza* | M025806 | Chang, Hson Mou; Cheng, Kwok Ping; Choang, Tai Francis; Chow, Hak Fun; Chui, Kuk Ying; et al.; Journal of Organic Chemistry; vol. 55; 11; (1990); p. 3537 - 3543. |
| F1 | *Salvia miltiorrhiza* | M026193 | Wang, Dandan; Girard, Thomas J.; Kasten, Thomas P.; LaChance, Rhonda M.; Miller-Wideman, Margaret A.; Durley, Richard C.; Journal of Natural Products; vol. 61; 11; (1998); p. 1352 - 1355. |
| F1 | *Salvia miltiorrhiza* | M026275 | Liu, Pu; Hu, Pan; Deng, Rui-Xue; Li, Ru; Yang, Li; Yin, Wei-Ping; Helvetica Chimica Acta; vol. 94; 1; (2011); p. 136 - 141. |
| F1 | *Salvia miltiorrhiza* | M026363 | Ryu, Shi Yong; Lee, Chong Ock; Choi, Sang Un; Planta Medica; vol. 63; 4; (1997); p. 339 - 342. |
| F1 | *Salvia miltiorrhiza* | M026401 | (1) 阴健等. 中药现代研究与临床应用(1). 北京: 学苑出版社, 1993. (2) 江苏新医学院. 中药大辞典. 上海: 上海科学技术出版社, 1977. |
| F1 | *Salvia miltiorrhiza* | M026421 | Luo, Hou-Wei; Wu, Bao-Jing; Wu, Mei-Yu; Yong, Zhong-Gen; Niwa, Masatake; Hirata, Yoshimasa; Phytochemistry (Elsevier); vol. 24; 4; (1985); p. 815 - 818. |
| F1 | *Salvia miltiorrhiza* | M026458 | Ryu, Shi Yong; Lee, Chong Ock; Choi, Sang Un; Planta Medica; vol. 63; 4; (1997); p. 339 - 342. |
| F1 | *Salvia miltiorrhiza* | M026497 | Lee AR, et al. JNP, 1987, 50 (2): 157 |
| F1 | *Salvia miltiorrhiza* | M026657 | Luo, Hou-Wei; Wu, Bao-Jing; Wu, Mei-Yu; Yong, Zhong-Gen; Niwa, Masatake; Hirata, Yoshimasa; Phytochemistry (Elsevier); vol. 24; 4; (1985); p. 815 - 818. |
| F1 | *Salvia miltiorrhiza* | M026792 | Chang, Hson Mou; Cheng, Kwok Ping; Choang, Tai Francis; Chow, Hak Fun; Chui, Kuk Ying; et al.; Journal of Organic Chemistry; vol. 55; 11; (1990); p. 3537 - 3543. |
| F1 | *Salvia miltiorrhiza* | M026803 | Nakanishi, Tsutomu; Miyasaka, Hitoshi; Nasu, Masao; Hashimoto, Hideko; Yonedo, Kaisuke; Phytochemistry (Elsevier); vol. 22; 3; (1983); p. 721 - 722. |
| F1 | *Salvia miltiorrhiza* | M027033 | 罗厚蔚等. 药学学报, 1985, 20 (7): 542 |
| F1 | *Salvia miltiorrhiza* | M027035 | Nguyen, Tien Dat; Jin, Xuejun; Lee, Jeong-Hyung; Lee, Dongho; Hong, Young-Soo; Lee, Kyeong; Young, Ho Kim; Jung, Joon Lee; Journal of Natural Products; vol. 70; 7; (2007); p. 1093 - 1097. |
| F1 | *Salvia miltiorrhiza* | M027038 | Nakao; Fukushima; Yakugaku Zasshi; vol. 54; (1934); p. 844,855, 857; engl. Ref. S. 154, 158; Chem.Abstr.; (1935); p. 788. |
| F1 | *Salvia miltiorrhiza* | M027665 | Don, Ming-Jaw; Shen, Chien-Chang; Syu, Wan-Jr; Ding, Yi-Huei; Sun, Chang-Ming; Phytochemistry; vol. 67; 5; (2006); p. 497 - 503. |
| F1 | *Salvia miltiorrhiza* | M027962 | Retrieved from CNPD |
| F1 | *Salvia miltiorrhiza* | M028464 | Ryu, Shi Yong; Lee, Chong Ock; Choi, Sang Un; Planta Medica; vol. 63; 4; (1997); p. 339 - 342. |
| F1 | *Salvia miltiorrhiza* | M029066 | Sun, Chang-Ming; Chin, Tsung-Mei; Lin, Yun-Lian; Chen, Chien-Jui; Chen, Wei-Chou; Wu, Tian-Shung; Don, Ming-Jaw; Heterocycles; vol. 68; 2; (2006); p. 247 - 255. |
| F1 | *Salvia miltiorrhiza* | M029095 | Yang, Zhen; Hon, Po Ming; Chui, Kuk Ying; Xu, Zun Le; Chang, Hson Mou; et al.; Tetrahedron Letters; vol. 32; 18; (1991); p. 2061 - 2064. |
| F1 | *Salvia miltiorrhiza* | M029210 | Kusumi, Takenori; Ooi, Takashi; Hayashi, Teruo; Kakisawa, Hiroshi; Phytochemistry (Elsevier); vol. 24; 9; (1985); p. 2118 - 2120. |
| F1 | *Salvia miltiorrhiza* | M029690 | Sun, Chang-Ming; Chin, Tsung-Mei; Lin, Yun-Lian; Chen, Chien-Jui; Chen, Wei-Chou; Wu, Tian-Shung; Don, Ming-Jaw; Heterocycles; vol. 68; 2; (2006); p. 247 - 255. |
| F1 | *Salvia miltiorrhiza* | M029823 | Luo, Hou Wei; Chen, Shaoxing; Lee, Junning; Snyder, John K.; Phytochemistry (Elsevier); vol. 27; 1; (1988); p. 290 - 292. |
| F1 | *Salvia miltiorrhiza* | M029850 | Retrieved from CNPD |
| F1 | *Salvia miltiorrhiza* | M030040 | Kang, Dae Gill; Oh, Hyuncheol; Sohn, Eun Jin; Hur, Tae Young; Lee, Kang Chang; Kim, Kwang Jin; Kim, Tai Yo; Lee, Ho Sub; Life Sciences; vol. 75; 15; (2004); p. 1801 - 1816. |
| F1 | *Salvia miltiorrhiza* | M030229 | Choi, Jae Sue; Kang, Hye Sook; Jung, Hyun Ah; Jung, Jee H; Kang, Sam Sik; Fitoterapia; vol. 72; 1; (2001); p. 30 - 34. |
| F1 | *Salvia miltiorrhiza* | M030328 | Ryu, Shi Yong; No, Zaesung; Kim, Sung Hoon; Ahn, Jong Woong; Planta Medica; vol. 63; 1; (1997); p. 44 - 46. |
| F1 | *Salvia miltiorrhiza* | M030390 | (1) 阴健等. 中药现代研究与临床应用(1). 北京: 学苑出版社, 1993. (2) 陈蕙芳等. 植物活性成分辞典. 第1册. 北京: 中国医药科技出版社, 2001. |
| F1 | *Salvia miltiorrhiza* | M030545 | Takiura; Yakugaku Zasshi; vol. 61; (1941); p. 475,481. |
| F1 | *Salvia miltiorrhiza* | M030679 | Don, Ming-Jaw; Shen, Chien-Chang; Syu, Wan-Jr; Ding, Yi-Huei; Sun, Chang-Ming; Phytochemistry; vol. 67; 5; (2006); p. 497 - 503. |
| F1 | *Salvia miltiorrhiza* | M030893 | Chang, Hson Mou; Cheng, Kwok Ping; Choang, Tai Francis; Chow, Hak Fun; Chui, Kuk Ying; et al.; Journal of Organic Chemistry; vol. 55; 11; (1990); p. 3537 - 3543. |
| F1 | *Salvia miltiorrhiza* | M031548 | Han, Yu Mi; Oh, Hyuncheol; Na, MinKyun; Kim, Beom Seok; Oh, Won Keun; Kim, Bo Yeon; Jeong, Dae Gwin; Ryu, Seong Eon; Sok, Dai-Eun; Ahn, Jong Seog; Biological and Pharmaceutical Bulletin; vol. 28; 9; (2005); p. 1795 - 1797. |
| F1 | *Salvia miltiorrhiza* | M031648 | Haro; Mori; Ishitsuka; Kusumi; Inouye; Kakisawa; Bulletin of the Chemical Society of Japan; vol. 64; 11; (1991); p. 3422 - 3426. |
| F1 | *Salvia miltiorrhiza* | M031667 | Ryu, Shi Yong; Lee, Chong Ock; Choi, Sang Un; Planta Medica; vol. 63; 4; (1997); p. 339 - 342. |
| F1 | *Salvia miltiorrhiza* | M031970 | Ryu, Shi Yong; No, Zaesung; Kim, Sung Hoon; Ahn, Jong Woong; Planta Medica; vol. 63; 1; (1997); p. 44 - 46. |
| F1 | *Salvia miltiorrhiza* | M032051 | Luo, Hou-Wei; Ji, Jiang; Wu, Mei-Yu; Yong Zhong-Gen; Niwa, Masatake; Hirata, Yoshimasa; Chemical & Pharmaceutical Bulletin; vol. 34; 8; (1986); p. 3166 - 3168. |
| F1 | *Salvia miltiorrhiza* | M032056 | Chang, Hson Mou; Cheng, Kwok Ping; Choang, Tai Francis; Chow, Hak Fun; Chui, Kuk Ying; et al.; Journal of Organic Chemistry; vol. 55; 11; (1990); p. 3537 - 3543. |
| F1 | *Salvia miltiorrhiza* | M032361 | Zhang, De-Wu; Liu, Xiao; Xie, Dan; Chen, Ridao; Tao, Xiao-Yu; Zou, Jian-Hua; Dai, Jungui; Chemical and Pharmaceutical Bulletin; vol. 61; 5; (2013); p. 576 - 580. |
| F1 | *Salvia miltiorrhiza* | M032385 | Lia, Xiao-Jun; Tang, Hao-Yu; Duan, Jia-Li; Gao, Jin-Ming; Xue, Quan-Hong; Natural Product Research; vol. 27; 4-5; (2013); p. 496 - 499. |
| F1 | *Salvia miltiorrhiza* | M032407 | Honda; Koezuka; Tabata; Chemical and Pharmaceutical Bulletin; vol. 36; 1; (1988); p. 408 - 411. |
| F1 | *Salvia miltiorrhiza* | M032529 | Park, Ji-Young; Kim, Jang Hoon; Kim, Young Min; Jeong, Hyung Jae; Kim, Dae Wook; Park, Ki Hun; Kwon, Hyung-Jun; Park, Su-Jin; Lee, Woo Song; Ryu, Young Bae; Bioorganic and Medicinal Chemistry; vol. 20; 19; (2012); p. 5928 - 5935. |
| F1 | *Salvia miltiorrhiza* | M032583 | Retrieved from CNPD |
| F1 | *Salvia miltiorrhiza* | M032987 | Retrieved from CNPD |
| F1 | *Salvia miltiorrhiza* | M033467 | 国家中医药管理局《中华本草》编委会. 中华本草. Vol. 1-30 上海:上海科学技术出版社, 1999 |
| F1 | *Salvia miltiorrhiza* | M033722 | Haro; Mori; Ishitsuka; Kusumi; Inouye; Kakisawa; Bulletin of the Chemical Society of Japan; vol. 64; 11; (1991); p. 3422 - 3426. |
| F1 | *Salvia miltiorrhiza* | M033792 | Yan, Xijun; Wu, Naifeng; Guo, Zhixin; Ye, Zhengliang; Liu, Yan; US2005/37094; A1; (2005). |
| F1 | *Salvia miltiorrhiza* | M033836 | 国家中医药管理局《中华本草》编委会. 中华本草. Vol. 1-30 上海:上海科学技术出版社, 1999 |
| F1 | *Schisandra sphenanthera* | M000259 | Ikeya; Miki; Okada; Mitsuhashi; Chai; Chemical and Pharmaceutical Bulletin; vol. 38; 5; (1990); p. 1408 - 1411. |
| F1 | *Schisandra sphenanthera* | M000285 | Ikeya; Miki; Okada; Mitsuhashi; Chai; Chemical and Pharmaceutical Bulletin; vol. 38; 5; (1990); p. 1408 - 1411. |
| F1 | *Schisandra sphenanthera* | M000797 | Xiao, Wei-Lie; Pu, Jian-Xin; Chang, Ying; Li, Xiao-Li; Huang, Sheng-Xiong; Yang, Liu-Meng; Li, Li-Mei; Lu, Yang; Zheng, Yong-Tang; Li, Rong-Tao; Zheng, Qi-Tai; Sun, Han-Dong; Organic Letters; vol. 8; 7; (2006); p. 1475 - 1478. |
| F1 | *Schisandra sphenanthera* | M001861 | Li, Yun-Fang; Jiang, Yan; Huang, Jing-Fang; Yang, Guang-Zhong; Journal of Asian Natural Products Research; vol. 15; 9; (2013); p. 934 - 940. |
| F1 | *Schisandra sphenanthera* | M002116 | (1) 阴健等. 中药现代研究与临床应用(1). 北京: 学苑出版社, 1993. (2) 王洪洁等. 药学学报, 1985, 20 (11): 832. (3) 孙文基等. 天然活性成分简明手册. 北京: 中国医药科技出版社, 1998. (4) Chen DF, et al. JNP, 2002, 65 (9): 1242,. (5) Lee IS, et al. Planta Med, 2003, 69, 63. |
| F1 | *Schisandra sphenanthera* | M002206 | Xiao, Wei-Lie; Yang, Liu-Meng; Li, Li-Mei; Pu, Jian-Xin; Huang, Sheng-Xiong; Weng, Zhi-Ying; Lei, Chun; Liu, Jing-Ping; Wang, Rui-Rui; Zheng, Yong-Tang; Li, Rong-Tao; Sun, Han-Dong; Tetrahedron Letters; vol. 48; 31; (2007); p. 5543 - 5546. |
| F1 | *Schisandra sphenanthera* | M002521 | Jin, Yong-Sheng; Du, Jing-Ling; Zou, Xiao-Hua; Zhao, Fei; Tuo, Xi-Ping; Chen, Hai-Sheng; Asian Journal of Chemistry; vol. 25; 4; (2013); p. 2321 - 2322. |
| F1 | *Schisandra sphenanthera* | M002689 | Jin, Yong-Sheng; Du, Jing-Ling; Zou, Xiao-Hua; Zhao, Fei; Tuo, Xi-Ping; Chen, Hai-Sheng; Asian Journal of Chemistry; vol. 25; 4; (2013); p. 2321 - 2322. |
| F1 | *Schisandra sphenanthera* | M003872 | Jin, Yong-Sheng; Du, Jing-Ling; Zou, Xiao-Hua; Zhao, Fei; Tuo, Xi-Ping; Chen, Hai-Sheng; Asian Journal of Chemistry; vol. 25; 4; (2013); p. 2321 - 2322. |
| F1 | *Schisandra sphenanthera* | M004287 | (1) 国家中医药管理局《中华本草》编委会. 中华本草. Vol. 1-30 上海:上海科学技术出版社, 1999. (2) 李晓光等. 中国中药杂志, 2003, 28 (12): 1120. (3) 周英等. 中国药学杂志, 2003, 38(2): 81. |
| F1 | *Schisandra sphenanthera* | M004633 | Ren, Rong; Ci, Xin-Xin; Li, Hai-Zhou; Li, Hong-Mei; Luo, Guo-Jun; Li, Rong-Tao; Deng, Xu-Ming; Zeitschrift fur Naturforschung - Section B Journal of Chemical Sciences; vol. 65; 2; (2010); p. 211 - 218. |
| F1 | *Schisandra sphenanthera* | M005021 | Liang, Cheng-Qin; Hu, Jing; Shi, Yi-Ming; Shang, Shan-Zhai; Du, Xue; Zhan, Rui; Xiong, Wen-Yong; Zhang, Hong-Bin; Xiao, Wei-Lie; Sun, Han-Dong; Chemical and Pharmaceutical Bulletin; vol. 61; 1; (2013); p. 96 - 100. |
| F1 | *Schisandra sphenanthera* | M005183 | Ikeya; Miki; Okada; Mitsuhashi; Chai; Chemical and Pharmaceutical Bulletin; vol. 38; 5; (1990); p. 1408 - 1411. |
| F1 | *Schisandra sphenanthera* | M005695 | Xiao, Wei-Lie; Huang, Sheng-Xiong; Wang, Rui-Rui; Zhong, Jia-Liang; Gao, Xue-Mei; He, Fei; Pu, Jian-Xin; Lu, Yang; Zheng, Yong-Tang; Zheng, Qi-Tai; Sun, Han-Dong; Phytochemistry; vol. 69; 16; (2008); p. 2862 - 2866. |
| F1 | *Schisandra sphenanthera* | M006256 | Jin, Yong-Sheng; Du, Jing-Ling; Zou, Xiao-Hua; Zhao, Fei; Tuo, Xi-Ping; Chen, Hai-Sheng; Asian Journal of Chemistry; vol. 25; 4; (2013); p. 2321 - 2322. |
| F1 | *Schisandra sphenanthera* | M007325 | Jiang, Yan; Yang, Guang-Zhong; Chen, Yu; Liao, Mao-Chuan; Liu, Xiang-Ming; Chen, Su; Liu, Lu; Lei, Xin-Xiang; Helvetica Chimica Acta; vol. 94; 3; (2011); p. 491 - 496. |
| F1 | *Schisandra sphenanthera* | M008051 | Ikeya; Miki; Okada; Mitsuhashi; Chai; Chemical and Pharmaceutical Bulletin; vol. 38; 5; (1990); p. 1408 - 1411. |
| F1 | *Schisandra sphenanthera* | M009307 | Li, Yun-Fang; Jiang, Yan; Huang, Jing-Fang; Yang, Guang-Zhong; Journal of Asian Natural Products Research; vol. 15; 9; (2013); p. 934 - 940. |
| F1 | *Schisandra sphenanthera* | M010078 | Liang, Cheng-Qin; Hu, Jing; Shi, Yi-Ming; Shang, Shan-Zhai; Du, Xue; Zhan, Rui; Xiong, Wen-Yong; Zhang, Hong-Bin; Xiao, Wei-Lie; Sun, Han-Dong; Chemical and Pharmaceutical Bulletin; vol. 61; 1; (2013); p. 96 - 100. |
| F1 | *Schisandra sphenanthera* | M010679 | Xiao, Wei-Lie; Pu, Jian-Xin; Chang, Ying; Li, Xiao-Li; Huang, Sheng-Xiong; Yang, Liu-Meng; Li, Li-Mei; Lu, Yang; Zheng, Yong-Tang; Li, Rong-Tao; Zheng, Qi-Tai; Sun, Han-Dong; Organic Letters; vol. 8; 7; (2006); p. 1475 - 1478. |
| F1 | *Schisandra sphenanthera* | M011151 | Mendbayar, Khongorzul; Lo, I-Wen; Liaw, Chia-Ching; Lin, Yu-Chi; Fazary, Ahmed E.; Kuo, Yuh-Chi; Wang, Hsiu-Ju; Chiang, Been Huang; Liou, Shorong-Shii; Shen, Ya-Ching; Helvetica Chimica Acta; vol. 94; 12; (2011); p. 2295 - 2302. |
| F1 | *Schisandra sphenanthera* | M011284 | Cui, Yu-Xin; Hua, Su-Ming; Cheng, Jin-Long; Yue, Jian-Min; Chen, Yao-Zu; Magnetic Resonance in Chemistry; vol. 27; 10; (1989); p. 998 - 1001. |
| F1 | *Schisandra sphenanthera* | M011709 | Jin, Yong-Sheng; Du, Jing-Ling; Zou, Xiao-Hua; Zhao, Fei; Tuo, Xi-Ping; Chen, Hai-Sheng; Asian Journal of Chemistry; vol. 25; 4; (2013); p. 2321 - 2322. |
| F1 | *Schisandra sphenanthera* | M012107 | Chen, Yu-Chen; Liaw, Chia-Ching; Cheng, Yuan-Bin; Lin, Yu-Chi; Chen, Chung-Hsiung; Huang, Yi-Tasu; Liou, Shorong-Shii; Chen, Shun-Ying; Chien, Ching-Te; Lee, Guo-Chi; Shen, Ya-Ching; Bioorganic and Medicinal Chemistry Letters; vol. 23; 3; (2013); p. 880 - 885. |
| F1 | *Schisandra sphenanthera* | M012154 | Ren, Rong; Ci, Xin-Xin; Li, Hai-Zhou; Li, Hong-Mei; Luo, Guo-Jun; Li, Rong-Tao; Deng, Xu-Ming; Zeitschrift fur Naturforschung - Section B Journal of Chemical Sciences; vol. 65; 2; (2010); p. 211 - 218. |
| F1 | *Schisandra sphenanthera* | M013289 | Liang, Cheng-Qin; Hu, Jing; Shi, Yi-Ming; Shang, Shan-Zhai; Du, Xue; Zhan, Rui; Xiong, Wen-Yong; Zhang, Hong-Bin; Xiao, Wei-Lie; Sun, Han-Dong; Chemical and Pharmaceutical Bulletin; vol. 61; 1; (2013); p. 96 - 100. |
| F1 | *Schisandra sphenanthera* | M013693 | Jiang, Yan; Yang, Guang-Zhong; Chen, Yu; Liao, Mao-Chuan; Liu, Xiang-Ming; Chen, Su; Liu, Lu; Lei, Xin-Xiang; Helvetica Chimica Acta; vol. 94; 3; (2011); p. 491 - 496. |
| F1 | *Schisandra sphenanthera* | M014132 | 周英等. 中国药学杂志, 2003, 38(2): 81 |
| F1 | *Schisandra sphenanthera* | M014355 | Ikeya, Yukinobu; Sugama, Ko; Okada, Minoru; Mitsuhashi, Hiroshi; Phytochemistry (Elsevier); vol. 30; 3; (1991); p. 975 - 980. |
| F1 | *Schisandra sphenanthera* | M014384 | He, Fei; Pu, Jian-Xin; Huang, Sheng-Xiong; Wang, Yuan-Yuan; Xiao, Wei-Lie; Li, Li-Mei; Liu, Jing-Ping; Zhang, Hai-Bo; Li, Yan; Sun, Han-Dong; Organic Letters; vol. 12; 6; (2010); p. 1208 - 1211. |
| F1 | *Schisandra sphenanthera* | M014721 | Ikeya, Yukinobu; Sugama, Ko; Okada, Minoru; Mitsuhashi, Hiroshi; Phytochemistry (Elsevier); vol. 30; 3; (1991); p. 975 - 980. |
| F1 | *Schisandra sphenanthera* | M015349 | He, Fei; Pu, Jian-Xin; Huang, Sheng-Xiong; Wang, Yuan-Yuan; Xiao, Wei-Lie; Li, Li-Mei; Liu, Jing-Ping; Zhang, Hai-Bo; Li, Yan; Sun, Han-Dong; Organic Letters; vol. 12; 6; (2010); p. 1208 - 1211. |
| F1 | *Schisandra sphenanthera* | M016116 | Ren, Rong; Ci, Xin-Xin; Li, Hai-Zhou; Li, Hong-Mei; Luo, Guo-Jun; Li, Rong-Tao; Deng, Xu-Ming; Zeitschrift fur Naturforschung - Section B Journal of Chemical Sciences; vol. 65; 2; (2010); p. 211 - 218. |
| F1 | *Schisandra sphenanthera* | M017129 | He, Fei; Li, Xing-Yao; Yang, Guang-Yu; Li, Xiao-Nian; Luo, Xiao; Zou, Juan; Li, Yan; Xiao, Wei-Lie; Sun, Han-Dong; Tetrahedron; vol. 68; 2; (2012); p. 440 - 446. |
| F1 | *Schisandra sphenanthera* | M017130 | He, Fei; Li, Xing-Yao; Yang, Guang-Yu; Li, Xiao-Nian; Luo, Xiao; Zou, Juan; Li, Yan; Xiao, Wei-Lie; Sun, Han-Dong; Tetrahedron; vol. 68; 2; (2012); p. 440 - 446. |
| F1 | *Schisandra sphenanthera* | M017131 | He, Fei; Li, Xing-Yao; Yang, Guang-Yu; Li, Xiao-Nian; Luo, Xiao; Zou, Juan; Li, Yan; Xiao, Wei-Lie; Sun, Han-Dong; Tetrahedron; vol. 68; 2; (2012); p. 440 - 446. |
| F1 | *Schisandra sphenanthera* | M017132 | He, Fei; Li, Xing-Yao; Yang, Guang-Yu; Li, Xiao-Nian; Luo, Xiao; Zou, Juan; Li, Yan; Xiao, Wei-Lie; Sun, Han-Dong; Tetrahedron; vol. 68; 2; (2012); p. 440 - 446. |
| F1 | *Schisandra sphenanthera* | M017133 | He, Fei; Li, Xing-Yao; Yang, Guang-Yu; Li, Xiao-Nian; Luo, Xiao; Zou, Juan; Li, Yan; Xiao, Wei-Lie; Sun, Han-Dong; Tetrahedron; vol. 68; 2; (2012); p. 440 - 446. |
| F1 | *Schisandra sphenanthera* | M017134 | He, Fei; Li, Xing-Yao; Yang, Guang-Yu; Li, Xiao-Nian; Luo, Xiao; Zou, Juan; Li, Yan; Xiao, Wei-Lie; Sun, Han-Dong; Tetrahedron; vol. 68; 2; (2012); p. 440 - 446. |
| F1 | *Schisandra sphenanthera* | M017135 | He, Fei; Li, Xing-Yao; Yang, Guang-Yu; Li, Xiao-Nian; Luo, Xiao; Zou, Juan; Li, Yan; Xiao, Wei-Lie; Sun, Han-Dong; Tetrahedron; vol. 68; 2; (2012); p. 440 - 446. |
| F1 | *Schisandra sphenanthera* | M017136 | He, Fei; Li, Xing-Yao; Yang, Guang-Yu; Li, Xiao-Nian; Luo, Xiao; Zou, Juan; Li, Yan; Xiao, Wei-Lie; Sun, Han-Dong; Tetrahedron; vol. 68; 2; (2012); p. 440 - 446. |
| F1 | *Schisandra sphenanthera* | M017215 | Mendbayar, Khongorzul; Lo, I-Wen; Liaw, Chia-Ching; Lin, Yu-Chi; Fazary, Ahmed E.; Kuo, Yuh-Chi; Wang, Hsiu-Ju; Chiang, Been Huang; Liou, Shorong-Shii; Shen, Ya-Ching; Helvetica Chimica Acta; vol. 94; 12; (2011); p. 2295 - 2302. |
| F1 | *Schisandra sphenanthera* | M017216 | Mendbayar, Khongorzul; Lo, I-Wen; Liaw, Chia-Ching; Lin, Yu-Chi; Fazary, Ahmed E.; Kuo, Yuh-Chi; Wang, Hsiu-Ju; Chiang, Been Huang; Liou, Shorong-Shii; Shen, Ya-Ching; Helvetica Chimica Acta; vol. 94; 12; (2011); p. 2295 - 2302. |
| F1 | *Schisandra sphenanthera* | M017217 | Mendbayar, Khongorzul; Lo, I-Wen; Liaw, Chia-Ching; Lin, Yu-Chi; Fazary, Ahmed E.; Kuo, Yuh-Chi; Wang, Hsiu-Ju; Chiang, Been Huang; Liou, Shorong-Shii; Shen, Ya-Ching; Helvetica Chimica Acta; vol. 94; 12; (2011); p. 2295 - 2302. |
| F1 | *Schisandra sphenanthera* | M017331 | Ma, Wen-Hui; Tan, Ce-Ming; He, Jian-Cheng; Duan, Peng-Shan; Qin, Lu-Ping; Chemistry of Natural Compounds; vol. 47; 5; (2011); p. 713 - 715. |
| F1 | *Schisandra sphenanthera* | M017332 | Ma, Wen-Hui; Tan, Ce-Ming; He, Jian-Cheng; Duan, Peng-Shan; Qin, Lu-Ping; Chemistry of Natural Compounds; vol. 47; 5; (2011); p. 713 - 715. |
| F1 | *Schisandra sphenanthera* | M017688 | Ikeya; Miki; Okada; Mitsuhashi; Chai; Chemical and Pharmaceutical Bulletin; vol. 38; 5; (1990); p. 1408 - 1411. |
| F1 | *Schisandra sphenanthera* | M018092 | Liang, Cheng-Qin; Luo, Rong-Hua; Yan, Ju-Ming; Li, Yan; Li, Xiao-Nian; Shi, Yi-Ming; Shang, Shan-Zhai; Gao, Zhong-Hua; Yang, Liu-Meng; Zheng, Yong-Tang; Xiao, Wei-Lie; Zhang, Hong-Bin; Sun, Han-Dong; Archives of Pharmacal Research; vol. 37; 2; (2014); p. 168 - 174. |
| F1 | *Schisandra sphenanthera* | M018111 | Li, Yun-Fang; Jiang, Yan; Huang, Jing-Fang; Yang, Guang-Zhong; Journal of Asian Natural Products Research; vol. 15; 9; (2013); p. 934 - 940. |
| F1 | *Schisandra sphenanthera* | M018528 | Liang, Cheng-Qin; Luo, Rong-Hua; Yan, Ju-Ming; Li, Yan; Li, Xiao-Nian; Shi, Yi-Ming; Shang, Shan-Zhai; Gao, Zhong-Hua; Yang, Liu-Meng; Zheng, Yong-Tang; Xiao, Wei-Lie; Zhang, Hong-Bin; Sun, Han-Dong; Archives of Pharmacal Research; vol. 37; 2; (2014); p. 168 - 174. |
| F1 | *Schisandra sphenanthera* | M018543 | Li, Yun-Fang; Jiang, Yan; Huang, Jing-Fang; Yang, Guang-Zhong; Journal of Asian Natural Products Research; vol. 15; 9; (2013); p. 934 - 940. |
| F1 | *Schisandra sphenanthera* | M018787 | Chen, Yu-Chen; Liaw, Chia-Ching; Cheng, Yuan-Bin; Lin, Yu-Chi; Chen, Chung-Hsiung; Huang, Yi-Tasu; Liou, Shorong-Shii; Chen, Shun-Ying; Chien, Ching-Te; Lee, Guo-Chi; Shen, Ya-Ching; Bioorganic and Medicinal Chemistry Letters; vol. 23; 3; (2013); p. 880 - 885. |
| F1 | *Schisandra sphenanthera* | M018967 | Liang, Cheng-Qin; Luo, Rong-Hua; Yan, Ju-Ming; Li, Yan; Li, Xiao-Nian; Shi, Yi-Ming; Shang, Shan-Zhai; Gao, Zhong-Hua; Yang, Liu-Meng; Zheng, Yong-Tang; Xiao, Wei-Lie; Zhang, Hong-Bin; Sun, Han-Dong; Archives of Pharmacal Research; vol. 37; 2; (2014); p. 168 - 174. |
| F1 | *Schisandra sphenanthera* | M019381 | Liang, Cheng-Qin; Luo, Rong-Hua; Yan, Ju-Ming; Li, Yan; Li, Xiao-Nian; Shi, Yi-Ming; Shang, Shan-Zhai; Gao, Zhong-Hua; Yang, Liu-Meng; Zheng, Yong-Tang; Xiao, Wei-Lie; Zhang, Hong-Bin; Sun, Han-Dong; Archives of Pharmacal Research; vol. 37; 2; (2014); p. 168 - 174. |
| F1 | *Schisandra sphenanthera* | M020022 | Liang, Cheng-Qin; Hu, Jing; Shi, Yi-Ming; Shang, Shan-Zhai; Du, Xue; Zhan, Rui; Xiong, Wen-Yong; Zhang, Hong-Bin; Xiao, Wei-Lie; Sun, Han-Dong; Chemical and Pharmaceutical Bulletin; vol. 61; 1; (2013); p. 96 - 100. |
| F1 | *Schisandra sphenanthera* | M020416 | Liang, Cheng-Qin; Hu, Jing; Shi, Yi-Ming; Shang, Shan-Zhai; Du, Xue; Zhan, Rui; Xiong, Wen-Yong; Zhang, Hong-Bin; Xiao, Wei-Lie; Sun, Han-Dong; Chemical and Pharmaceutical Bulletin; vol. 61; 1; (2013); p. 96 - 100. |
| F1 | *Schisandra sphenanthera* | M020612 | Li, Yun-Fang; Jiang, Yan; Huang, Jing-Fang; Yang, Guang-Zhong; Journal of Asian Natural Products Research; vol. 15; 9; (2013); p. 934 - 940. |
| F1 | *Schisandra sphenanthera* | M020716 | Jiang, Yan; Yang, Guang-Zhong; Chen, Yu; Liao, Mao-Chuan; Liu, Xiang-Ming; Chen, Su; Liu, Lu; Lei, Xin-Xiang; Helvetica Chimica Acta; vol. 94; 3; (2011); p. 491 - 496. |
| F1 | *Schisandra sphenanthera* | M020842 | Liang, Cheng-Qin; Hu, Jing; Shi, Yi-Ming; Shang, Shan-Zhai; Du, Xue; Zhan, Rui; Xiong, Wen-Yong; Zhang, Hong-Bin; Xiao, Wei-Lie; Sun, Han-Dong; Chemical and Pharmaceutical Bulletin; vol. 61; 1; (2013); p. 96 - 100. |
| F1 | *Schisandra sphenanthera* | M021076 | Jin, Yong-Sheng; Du, Jing-Ling; Zou, Xiao-Hua; Zhao, Fei; Tuo, Xi-Ping; Chen, Hai-Sheng; Asian Journal of Chemistry; vol. 25; 4; (2013); p. 2321 - 2322. |
| F1 | *Schisandra sphenanthera* | M021137 | He, Fei; Pu, Jian-Xin; Huang, Sheng-Xiong; Wang, Yuan-Yuan; Xiao, Wei-Lie; Li, Li-Mei; Liu, Jing-Ping; Zhang, Hai-Bo; Li, Yan; Sun, Han-Dong; Organic Letters; vol. 12; 6; (2010); p. 1208 - 1211. |
| F1 | *Schisandra sphenanthera* | M021292 | Liang, Cheng-Qin; Hu, Jing; Shi, Yi-Ming; Shang, Shan-Zhai; Du, Xue; Zhan, Rui; Xiong, Wen-Yong; Zhang, Hong-Bin; Xiao, Wei-Lie; Sun, Han-Dong; Chemical and Pharmaceutical Bulletin; vol. 61; 1; (2013); p. 96 - 100. |
| F1 | *Schisandra sphenanthera* | M021322 | Jin, Yong-Sheng; Du, Jing-Ling; Zou, Xiao-Hua; Zhao, Fei; Tuo, Xi-Ping; Chen, Hai-Sheng; Asian Journal of Chemistry; vol. 25; 4; (2013); p. 2321 - 2322. |
| F1 | *Schisandra sphenanthera* | M021507 | Li, Yun-Fang; Jiang, Yan; Huang, Jing-Fang; Yang, Guang-Zhong; Journal of Asian Natural Products Research; vol. 15; 9; (2013); p. 934 - 940. |
| F1 | *Schisandra sphenanthera* | M021602 | Jiang, Yan; Yang, Guang-Zhong; Chen, Yu; Liao, Mao-Chuan; Liu, Xiang-Ming; Chen, Su; Liu, Lu; Lei, Xin-Xiang; Helvetica Chimica Acta; vol. 94; 3; (2011); p. 491 - 496. |
| F1 | *Schisandra sphenanthera* | M021731 | Liang, Cheng-Qin; Hu, Jing; Shi, Yi-Ming; Shang, Shan-Zhai; Du, Xue; Zhan, Rui; Xiong, Wen-Yong; Zhang, Hong-Bin; Xiao, Wei-Lie; Sun, Han-Dong; Chemical and Pharmaceutical Bulletin; vol. 61; 1; (2013); p. 96 - 100. |
| F1 | *Schisandra sphenanthera* | M022879 | Jin, Yong-Sheng; Du, Jing-Ling; Zou, Xiao-Hua; Zhao, Fei; Tuo, Xi-Ping; Chen, Hai-Sheng; Asian Journal of Chemistry; vol. 25; 4; (2013); p. 2321 - 2322. |
| F1 | *Schisandra sphenanthera* | M023243 | Li, Yun-Fang; Jiang, Yan; Huang, Jing-Fang; Yang, Guang-Zhong; Journal of Asian Natural Products Research; vol. 15; 9; (2013); p. 934 - 940. |
| F1 | *Schisandra sphenanthera* | M023507 | Xiao, Wei-Lie; Yang, Liu-Meng; Li, Li-Mei; Pu, Jian-Xin; Huang, Sheng-Xiong; Weng, Zhi-Ying; Lei, Chun; Liu, Jing-Ping; Wang, Rui-Rui; Zheng, Yong-Tang; Li, Rong-Tao; Sun, Han-Dong; Tetrahedron Letters; vol. 48; 31; (2007); p. 5543 - 5546. |
| F1 | *Schisandra sphenanthera* | M023877 | He, Fei; Pu, Jian-Xin; Huang, Sheng-Xiong; Wang, Yuan-Yuan; Xiao, Wei-Lie; Li, Li-Mei; Liu, Jing-Ping; Zhang, Hai-Bo; Li, Yan; Sun, Han-Dong; Organic Letters; vol. 12; 6; (2010); p. 1208 - 1211. |
| F1 | *Schisandra sphenanthera* | M024092 | Jin, Yong-Sheng; Du, Jing-Ling; Zou, Xiao-Hua; Zhao, Fei; Tuo, Xi-Ping; Chen, Hai-Sheng; Asian Journal of Chemistry; vol. 25; 4; (2013); p. 2321 - 2322. |
| F1 | *Schisandra sphenanthera* | M024261 | Jin, Yong-Sheng; Du, Jing-Ling; Zou, Xiao-Hua; Zhao, Fei; Tuo, Xi-Ping; Chen, Hai-Sheng; Asian Journal of Chemistry; vol. 25; 4; (2013); p. 2321 - 2322. |
| F1 | *Schisandra sphenanthera* | M024448 | Xiao, Wei-Lie; Huang, Sheng-Xiong; Wang, Rui-Rui; Zhong, Jia-Liang; Gao, Xue-Mei; He, Fei; Pu, Jian-Xin; Lu, Yang; Zheng, Yong-Tang; Zheng, Qi-Tai; Sun, Han-Dong; Phytochemistry; vol. 69; 16; (2008); p. 2862 - 2866. |
| F1 | *Schisandra sphenanthera* | M024528 | Xiao, Wei-Lie; Yang, Liu-Meng; Li, Li-Mei; Pu, Jian-Xin; Huang, Sheng-Xiong; Weng, Zhi-Ying; Lei, Chun; Liu, Jing-Ping; Wang, Rui-Rui; Zheng, Yong-Tang; Li, Rong-Tao; Sun, Han-Dong; Tetrahedron Letters; vol. 48; 31; (2007); p. 5543 - 5546. |
| F1 | *Schisandra sphenanthera* | M025106 | Ren, Rong; Ci, Xin-Xin; Li, Hai-Zhou; Li, Hong-Mei; Luo, Guo-Jun; Li, Rong-Tao; Deng, Xu-Ming; Zeitschrift fur Naturforschung - Section B Journal of Chemical Sciences; vol. 65; 2; (2010); p. 211 - 218. |
| F1 | *Schisandra sphenanthera* | M025169 | Ikeya; Miki; Okada; Mitsuhashi; Chai; Chemical and Pharmaceutical Bulletin; vol. 38; 5; (1990); p. 1408 - 1411. |
| F1 | *Schisandra sphenanthera* | M025718 | Ren, Rong; Ci, Xin-Xin; Li, Hai-Zhou; Li, Hong-Mei; Luo, Guo-Jun; Li, Rong-Tao; Deng, Xu-Ming; Zeitschrift fur Naturforschung - Section B Journal of Chemical Sciences; vol. 65; 2; (2010); p. 211 - 218. |
| F1 | *Schisandra sphenanthera* | M026944 | 国家中医药管理局《中华本草》编委会. 中华本草. Vol. 1-30 上海:上海科学技术出版社, 1999 |
| F1 | *Schisandra sphenanthera* | M027095 | Ren, Rong; Ci, Xin-Xin; Li, Hai-Zhou; Li, Hong-Mei; Luo, Guo-Jun; Li, Rong-Tao; Deng, Xu-Ming; Zeitschrift fur Naturforschung - Section B Journal of Chemical Sciences; vol. 65; 2; (2010); p. 211 - 218. |
| F1 | *Schisandra sphenanthera* | M027170 | 周英等. 中国药学杂志, 2003, 38(2): 81 |
| F1 | *Schisandra sphenanthera* | M027968 | Chen, Yu-Chen; Liaw, Chia-Ching; Cheng, Yuan-Bin; Lin, Yu-Chi; Chen, Chung-Hsiung; Huang, Yi-Tasu; Liou, Shorong-Shii; Chen, Shun-Ying; Chien, Ching-Te; Lee, Guo-Chi; Shen, Ya-Ching; Bioorganic and Medicinal Chemistry Letters; vol. 23; 3; (2013); p. 880 - 885. |
| F1 | *Schisandra sphenanthera* | M028219 | Ikeya; Miki; Okada; Mitsuhashi; Chai; Chemical and Pharmaceutical Bulletin; vol. 38; 5; (1990); p. 1408 - 1411. |
| F1 | *Schisandra sphenanthera* | M028729 | Li, Yun-Fang; Jiang, Yan; Huang, Jing-Fang; Yang, Guang-Zhong; Journal of Asian Natural Products Research; vol. 15; 9; (2013); p. 934 - 940. |
| F1 | *Schisandra sphenanthera* | M029599 | Ikeya, Yukinobu; Sugama, Ko; Okada, Minoru; Mitsuhashi, Hiroshi; Phytochemistry (Elsevier); vol. 30; 3; (1991); p. 975 - 980. |
| F1 | *Schisandra sphenanthera* | M029686 | Jiang, Yan; Yang, Guang-Zhong; Chen, Yu; Liao, Mao-Chuan; Liu, Xiang-Ming; Chen, Su; Liu, Lu; Lei, Xin-Xiang; Helvetica Chimica Acta; vol. 94; 3; (2011); p. 491 - 496. |
| F1 | *Schisandra sphenanthera* | M030250 | Xiao, Wei-Lie; Yang, Liu-Meng; Li, Li-Mei; Pu, Jian-Xin; Huang, Sheng-Xiong; Weng, Zhi-Ying; Lei, Chun; Liu, Jing-Ping; Wang, Rui-Rui; Zheng, Yong-Tang; Li, Rong-Tao; Sun, Han-Dong; Tetrahedron Letters; vol. 48; 31; (2007); p. 5543 - 5546. |
| F1 | *Schisandra sphenanthera* | M030312 | Li, Yun-Fang; Jiang, Yan; Huang, Jing-Fang; Yang, Guang-Zhong; Journal of Asian Natural Products Research; vol. 15; 9; (2013); p. 934 - 940. |
| F1 | *Schisandra sphenanthera* | M031368 | Yue, Jian-min; Xu-Jun; Chen, Yao-zu; Phytochemistry (Elsevier); vol. 35; 4; (1994); p. 1068 - 1069. |
| F1 | *Schisandra sphenanthera* | M032085 | Liang, Cheng-Qin; Hu, Jing; Shi, Yi-Ming; Shang, Shan-Zhai; Du, Xue; Zhan, Rui; Xiong, Wen-Yong; Zhang, Hong-Bin; Xiao, Wei-Lie; Sun, Han-Dong; Chemical and Pharmaceutical Bulletin; vol. 61; 1; (2013); p. 96 - 100. |
| F1 | *Schisandra sphenanthera* | M032128 | Jin, Yong-Sheng; Du, Jing-Ling; Zou, Xiao-Hua; Zhao, Fei; Tuo, Xi-Ping; Chen, Hai-Sheng; Asian Journal of Chemistry; vol. 25; 4; (2013); p. 2321 - 2322. |
| F1 | *Schisandra sphenanthera* | M032475 | Ren, Rong; Ci, Xin-Xin; Li, Hai-Zhou; Li, Hong-Mei; Luo, Guo-Jun; Li, Rong-Tao; Deng, Xu-Ming; Zeitschrift fur Naturforschung - Section B Journal of Chemical Sciences; vol. 65; 2; (2010); p. 211 - 218. |
| F1 | *Schisandra sphenanthera* | M032766 | Ren, Rong; Ci, Xin-Xin; Li, Hai-Zhou; Li, Hong-Mei; Luo, Guo-Jun; Li, Rong-Tao; Deng, Xu-Ming; Zeitschrift fur Naturforschung - Section B Journal of Chemical Sciences; vol. 65; 2; (2010); p. 211 - 218. |
| F1 | *Schisandra sphenanthera* | M033229 | Ikeya; Miki; Okada; Mitsuhashi; Chai; Chemical and Pharmaceutical Bulletin; vol. 38; 5; (1990); p. 1408 - 1411. |
| F1 | *Schisandra sphenanthera* | M033555 | Ikeya; Miki; Okada; Mitsuhashi; Chai; Chemical and Pharmaceutical Bulletin; vol. 38; 5; (1990); p. 1408 - 1411. |
| F1 | *Schisandra sphenanthera* | M033617 | Xiao, Wei-Lie; Huang, Sheng-Xiong; Wang, Rui-Rui; Zhong, Jia-Liang; Gao, Xue-Mei; He, Fei; Pu, Jian-Xin; Lu, Yang; Zheng, Yong-Tang; Zheng, Qi-Tai; Sun, Han-Dong; Phytochemistry; vol. 69; 16; (2008); p. 2862 - 2866. |
| F1 | *Schisandra sphenanthera* | M034023 | Jin, Yong-Sheng; Du, Jing-Ling; Zou, Xiao-Hua; Zhao, Fei; Tuo, Xi-Ping; Chen, Hai-Sheng; Asian Journal of Chemistry; vol. 25; 4; (2013); p. 2321 - 2322. |
| F1 | *Smilax glabra* | M000074 | Chien; Adam; Pharmazie; vol. 34; (1979); p. 841,842. |
| F1 | *Smilax glabra* | M000560 | Xu, Shuo; Shang, Ming-Ying; Liu, Guang-Xue; Xu, Feng; Wang, Xuan; Shou, Cheng-Chao; Cai, Shao-Qing; Molecules; vol. 18; 5; (2013); p. 5265 - 5287. |
| F1 | *Smilax glabra* | M001995 | Yuan, Jiuzhi; Li, Wei; Koike, Kazuo; Chen, Yingjie; Nikaido, Tamotsu; Heterocycles; vol. 60; 7; (2003); p. 1633 - 1637. |
| F1 | *Smilax glabra* | M002480 | Yuan, Jiuzhi; Li, Wei; Koike, Kazuo; Chen, Yingjie; Nikaido, Tamotsu; Heterocycles; vol. 60; 7; (2003); p. 1633 - 1637. |
| F1 | *Smilax glabra* | M003996 | Yuan, Jiuzhi; Li, Wei; Koike, Kazuo; Chen, Yingjie; Nikaido, Tamotsu; Heterocycles; vol. 60; 7; (2003); p. 1633 - 1637. |
| F1 | *Smilax glabra* | M004752 | Xu, Shuo; Shang, Ming-Ying; Liu, Guang-Xue; Xu, Feng; Wang, Xuan; Shou, Cheng-Chao; Cai, Shao-Qing; Molecules; vol. 18; 5; (2013); p. 5265 - 5287. |
| F1 | *Smilax glabra* | M005337 | Chen, Ting; Li, Jian-Xin; Xu, Qiang; Phytochemistry; vol. 53; 8; (2000); p. 1051 - 1055. |
| F1 | *Smilax glabra* | M005404 | Xu, Shuo; Shang, Ming-Ying; Liu, Guang-Xue; Xu, Feng; Wang, Xuan; Shou, Cheng-Chao; Cai, Shao-Qing; Molecules; vol. 18; 5; (2013); p. 5265 - 5287. |
| F1 | *Smilax glabra* | M005588 | Chen, Ting; Li, Jianxin; Cao, Jingsong; Xu, Qiang; Komatsu, Katsuko; Namba, Tsuneo; Planta Medica; vol. 65; 1; (1999); p. 56 - 59. |
| F1 | *Smilax glabra* | M006025 | (1) Chen T, et al. Planta Med, 1999, 65 (1): 56. (2) K?rm?z?bekmez H, et al. Planta Med, 2004, 70 (8): 711. |
| F1 | *Smilax glabra* | M006085 | Xu, Shuo; Shang, Ming-Ying; Liu, Guang-Xue; Xu, Feng; Wang, Xuan; Shou, Cheng-Chao; Cai, Shao-Qing; Molecules; vol. 18; 5; (2013); p. 5265 - 5287. |
| F1 | *Smilax glabra* | M006368 | Chen, Ting; Li, Jian-Xin; Xu, Qiang; Phytochemistry; vol. 53; 8; (2000); p. 1051 - 1055. |
| F1 | *Smilax glabra* | M007277 | Xu, Shuo; Shang, Ming-Ying; Liu, Guang-Xue; Xu, Feng; Wang, Xuan; Shou, Cheng-Chao; Cai, Shao-Qing; Molecules; vol. 18; 5; (2013); p. 5265 - 5287. |
| F1 | *Smilax glabra* | M007635 | Yuan, Jiuzhi; Li, Wei; Koike, Kazuo; Chen, Yingjie; Nikaido, Tamotsu; Heterocycles; vol. 60; 7; (2003); p. 1633 - 1637. |
| F1 | *Smilax glabra* | M008055 | Xu, Shuo; Shang, Ming-Ying; Liu, Guang-Xue; Xu, Feng; Wang, Xuan; Shou, Cheng-Chao; Cai, Shao-Qing; Molecules; vol. 18; 5; (2013); p. 5265 - 5287. |
| F1 | *Smilax glabra* | M008130 | Xu, Shuo; Shang, Ming-Ying; Liu, Guang-Xue; Xu, Feng; Wang, Xuan; Shou, Cheng-Chao; Cai, Shao-Qing; Molecules; vol. 18; 5; (2013); p. 5265 - 5287. |
| F1 | *Smilax glabra* | M008884 | Yuan, Jiuzhi; Li, Wei; Koike, Kazuo; Chen, Yingjie; Nikaido, Tamotsu; Heterocycles; vol. 60; 7; (2003); p. 1633 - 1637. |
| F1 | *Smilax glabra* | M008919 | (1) 李伊庆等. 药学学报, 1996, 31 (2): 761. (2) 陈广耀等. 中国中药杂志, 1996, 21 (6): 355. (3) KONISHI T, et al. Chem Pharm Bull, 2003, 51 (10): 1142. |
| F1 | *Smilax glabra* | M009001 | Yuan, Jiuzhi; Li, Wei; Koike, Kazuo; Chen, Yingjie; Nikaido, Tamotsu; Heterocycles; vol. 60; 7; (2003); p. 1633 - 1637. |
| F1 | *Smilax glabra* | M009327 | Xu, Shuo; Shang, Ming-Ying; Liu, Guang-Xue; Xu, Feng; Wang, Xuan; Shou, Cheng-Chao; Cai, Shao-Qing; Molecules; vol. 18; 5; (2013); p. 5265 - 5287. |
| F1 | *Smilax glabra* | M009539 | Chien; Adam; Pharmazie; vol. 34; (1979); p. 841,842. |
| F1 | *Smilax glabra* | M009757 | Yuan, Jiuzhi; Li, Wei; Koike, Kazuo; Chen, Yingjie; Nikaido, Tamotsu; Heterocycles; vol. 60; 7; (2003); p. 1633 - 1637. |
| F1 | *Smilax glabra* | M009870 | Xu, Shuo; Shang, Ming-Ying; Liu, Guang-Xue; Xu, Feng; Wang, Xuan; Shou, Cheng-Chao; Cai, Shao-Qing; Molecules; vol. 18; 5; (2013); p. 5265 - 5287. |
| F1 | *Smilax glabra* | M010220 | Chen, Ting; Li, Jianxin; Cao, Jingsong; Xu, Qiang; Komatsu, Katsuko; Namba, Tsuneo; Planta Medica; vol. 65; 1; (1999); p. 56 - 59. |
| F1 | *Smilax glabra* | M010812 | Xu, Shuo; Shang, Ming-Ying; Liu, Guang-Xue; Xu, Feng; Wang, Xuan; Shou, Cheng-Chao; Cai, Shao-Qing; Molecules; vol. 18; 5; (2013); p. 5265 - 5287. |
| F1 | *Smilax glabra* | M010978 | Xu, Shuo; Shang, Ming-Ying; Liu, Guang-Xue; Xu, Feng; Wang, Xuan; Shou, Cheng-Chao; Cai, Shao-Qing; Molecules; vol. 18; 5; (2013); p. 5265 - 5287. |
| F1 | *Smilax glabra* | M011141 | Yuan, Jiuzhi; Li, Wei; Koike, Kazuo; Chen, Yingjie; Nikaido, Tamotsu; Heterocycles; vol. 60; 7; (2003); p. 1633 - 1637. |
| F1 | *Smilax glabra* | M011867 | Chen, Ting; Li, Jianxin; Cao, Jingsong; Xu, Qiang; Komatsu, Katsuko; Namba, Tsuneo; Planta Medica; vol. 65; 1; (1999); p. 56 - 59. |
| F1 | *Smilax glabra* | M012094 | Chen, Ting; Li, Jianxin; Cao, Jingsong; Xu, Qiang; Komatsu, Katsuko; Namba, Tsuneo; Planta Medica; vol. 65; 1; (1999); p. 56 - 59. |
| F1 | *Smilax glabra* | M012254 | Xu, Shuo; Shang, Ming-Ying; Liu, Guang-Xue; Xu, Feng; Wang, Xuan; Shou, Cheng-Chao; Cai, Shao-Qing; Molecules; vol. 18; 5; (2013); p. 5265 - 5287. |
| F1 | *Smilax glabra* | M012581 | Chen, Ting; Li, Jian-Xin; Xu, Qiang; Phytochemistry; vol. 53; 8; (2000); p. 1051 - 1055. |
| F1 | *Smilax glabra* | M012601 | (1) 江苏新医学院. 中药大辞典. 上海: 上海科学技术出版社, 1977. (2) 易以军等. 药学学报, 1995, 30 (9): 718. (3) 孙文基等. 天然活性成分简明手册. 北京: 中国医药科技出版社, 1998. (4) 国家中医药管理局《中华�静荨繁辔� 中华本草. Vol. 1-30 上海:上海科学技术出版社, 1999. (5) Wu PL, et al. Chem Pharm Bull, 2005, 53 (1): 56. (6) TAO J, et al. Chem Pharm Bull, 2003, 51 (6): 654. (7) LEU Y-L, et al. Chem Pharm Bull, 2005, 53 (7): 853. (8) WU Q, et al. Chem Pharm Bull, 2005, 53 (8): 1065. (9) Pan WB, et al. JNP, 2003, 66 (1): 161. (10) Morikawa T, et al. JNP, 2003, 66 (5): 638. (11) Mo S, et al. JNP, 2004, 67 (5): 823. |
| F1 | *Smilax glabra* | M012639 | Xu, Shuo; Shang, Ming-Ying; Liu, Guang-Xue; Xu, Feng; Wang, Xuan; Shou, Cheng-Chao; Cai, Shao-Qing; Molecules; vol. 18; 5; (2013); p. 5265 - 5287. |
| F1 | *Smilax glabra* | M013251 | Xu, Shuo; Shang, Ming-Ying; Liu, Guang-Xue; Xu, Feng; Wang, Xuan; Shou, Cheng-Chao; Cai, Shao-Qing; Molecules; vol. 18; 5; (2013); p. 5265 - 5287. |
| F1 | *Smilax glabra* | M013631 | Xu, Shuo; Shang, Ming-Ying; Liu, Guang-Xue; Xu, Feng; Wang, Xuan; Shou, Cheng-Chao; Cai, Shao-Qing; Molecules; vol. 18; 5; (2013); p. 5265 - 5287. |
| F1 | *Smilax glabra* | M014203 | Xu, Shuo; Shang, Ming-Ying; Liu, Guang-Xue; Xu, Feng; Wang, Xuan; Shou, Cheng-Chao; Cai, Shao-Qing; Molecules; vol. 18; 5; (2013); p. 5265 - 5287. |
| F1 | *Smilax glabra* | M014455 | Xu, Shuo; Shang, Ming-Ying; Liu, Guang-Xue; Xu, Feng; Wang, Xuan; Shou, Cheng-Chao; Cai, Shao-Qing; Molecules; vol. 18; 5; (2013); p. 5265 - 5287. |
| F1 | *Smilax glabra* | M015119 | Chen, Ting; Li, Jian-Xin; Xu, Qiang; Phytochemistry; vol. 53; 8; (2000); p. 1051 - 1055. |
| F1 | *Smilax glabra* | M015628 | Xu, Shuo; Shang, Ming-Ying; Liu, Guang-Xue; Xu, Feng; Wang, Xuan; Shou, Cheng-Chao; Cai, Shao-Qing; Molecules; vol. 18; 5; (2013); p. 5265 - 5287. |
| F1 | *Smilax glabra* | M017951 | Xu, Shuo; Shang, Ming-Ying; Liu, Guang-Xue; Xu, Feng; Wang, Xuan; Shou, Cheng-Chao; Cai, Shao-Qing; Molecules; vol. 18; 5; (2013); p. 5265 - 5287. |
| F1 | *Smilax glabra* | M018226 | Xu, Shuo; Shang, Ming-Ying; Liu, Guang-Xue; Xu, Feng; Wang, Xuan; Shou, Cheng-Chao; Cai, Shao-Qing; Molecules; vol. 18; 5; (2013); p. 5265 - 5287. |
| F1 | *Smilax glabra* | M018396 | Xu, Shuo; Shang, Ming-Ying; Liu, Guang-Xue; Xu, Feng; Wang, Xuan; Shou, Cheng-Chao; Cai, Shao-Qing; Molecules; vol. 18; 5; (2013); p. 5265 - 5287. |
| F1 | *Smilax glabra* | M018564 | Xu, Shuo; Shang, Ming-Ying; Liu, Guang-Xue; Xu, Feng; Wang, Xuan; Shou, Cheng-Chao; Cai, Shao-Qing; Molecules; vol. 18; 5; (2013); p. 5265 - 5287. |
| F1 | *Smilax glabra* | M018565 | Xu, Shuo; Shang, Ming-Ying; Liu, Guang-Xue; Xu, Feng; Wang, Xuan; Shou, Cheng-Chao; Cai, Shao-Qing; Molecules; vol. 18; 5; (2013); p. 5265 - 5287. |
| F1 | *Smilax glabra* | M018678 | Xu, Shuo; Shang, Ming-Ying; Liu, Guang-Xue; Xu, Feng; Wang, Xuan; Shou, Cheng-Chao; Cai, Shao-Qing; Molecules; vol. 18; 5; (2013); p. 5265 - 5287. |
| F1 | *Smilax glabra* | M018830 | Xu, Shuo; Shang, Ming-Ying; Liu, Guang-Xue; Xu, Feng; Wang, Xuan; Shou, Cheng-Chao; Cai, Shao-Qing; Molecules; vol. 18; 5; (2013); p. 5265 - 5287. |
| F1 | *Smilax glabra* | M019243 | Xu, Shuo; Shang, Ming-Ying; Liu, Guang-Xue; Xu, Feng; Wang, Xuan; Shou, Cheng-Chao; Cai, Shao-Qing; Molecules; vol. 18; 5; (2013); p. 5265 - 5287. |
| F1 | *Smilax glabra* | M019670 | Xu, Shuo; Shang, Ming-Ying; Liu, Guang-Xue; Xu, Feng; Wang, Xuan; Shou, Cheng-Chao; Cai, Shao-Qing; Molecules; vol. 18; 5; (2013); p. 5265 - 5287. |
| F1 | *Smilax glabra* | M020241 | Xu, Shuo; Shang, Ming-Ying; Liu, Guang-Xue; Xu, Feng; Wang, Xuan; Shou, Cheng-Chao; Cai, Shao-Qing; Molecules; vol. 18; 5; (2013); p. 5265 - 5287. |
| F1 | *Smilax glabra* | M020470 | Xu, Shuo; Shang, Ming-Ying; Liu, Guang-Xue; Xu, Feng; Wang, Xuan; Shou, Cheng-Chao; Cai, Shao-Qing; Molecules; vol. 18; 5; (2013); p. 5265 - 5287. |
| F1 | *Smilax glabra* | M020635 | Xu, Shuo; Shang, Ming-Ying; Liu, Guang-Xue; Xu, Feng; Wang, Xuan; Shou, Cheng-Chao; Cai, Shao-Qing; Molecules; vol. 18; 5; (2013); p. 5265 - 5287. |
| F1 | *Smilax glabra* | M020666 | Xu, Shuo; Shang, Ming-Ying; Liu, Guang-Xue; Xu, Feng; Wang, Xuan; Shou, Cheng-Chao; Cai, Shao-Qing; Molecules; vol. 18; 5; (2013); p. 5265 - 5287. |
| F1 | *Smilax glabra* | M020677 | Xu, Shuo; Shang, Ming-Ying; Liu, Guang-Xue; Xu, Feng; Wang, Xuan; Shou, Cheng-Chao; Cai, Shao-Qing; Molecules; vol. 18; 5; (2013); p. 5265 - 5287. |
| F1 | *Smilax glabra* | M020889 | Xu, Shuo; Shang, Ming-Ying; Liu, Guang-Xue; Xu, Feng; Wang, Xuan; Shou, Cheng-Chao; Cai, Shao-Qing; Molecules; vol. 18; 5; (2013); p. 5265 - 5287. |
| F1 | *Smilax glabra* | M021347 | Xu, Shuo; Shang, Ming-Ying; Liu, Guang-Xue; Xu, Feng; Wang, Xuan; Shou, Cheng-Chao; Cai, Shao-Qing; Molecules; vol. 18; 5; (2013); p. 5265 - 5287. |
| F1 | *Smilax glabra* | M022109 | Chen T, et al. Phytochemistry, 2000, 53 (8): 1051 |
| F1 | *Smilax glabra* | M022285 | Yuan, Jiuzhi; Li, Wei; Koike, Kazuo; Chen, Yingjie; Nikaido, Tamotsu; Heterocycles; vol. 60; 7; (2003); p. 1633 - 1637. |
| F1 | *Smilax glabra* | M022333 | Xu, Shuo; Shang, Ming-Ying; Liu, Guang-Xue; Xu, Feng; Wang, Xuan; Shou, Cheng-Chao; Cai, Shao-Qing; Molecules; vol. 18; 5; (2013); p. 5265 - 5287. |
| F1 | *Smilax glabra* | M022909 | Xu, Shuo; Shang, Ming-Ying; Liu, Guang-Xue; Xu, Feng; Wang, Xuan; Shou, Cheng-Chao; Cai, Shao-Qing; Molecules; vol. 18; 5; (2013); p. 5265 - 5287. |
| F1 | *Smilax glabra* | M023318 | Yuan, Jiuzhi; Li, Wei; Koike, Kazuo; Chen, Yingjie; Nikaido, Tamotsu; Heterocycles; vol. 60; 7; (2003); p. 1633 - 1637. |
| F1 | *Smilax glabra* | M024219 | 易以军等. 药学学报, 1998, 33 (11): 873 |
| F1 | *Smilax glabra* | M024503 | (1) 江苏新医学院. 中药大辞典. 上海: 上海科学技术出版社, 1977. (2) 陈广耀等. 中国中药杂志, 1996, 21 (6): 355. (3) Buckingham J(Executive Editor): et al. Dictionary of Natural Products, Vol 1-7, Chapman & Hall, London, 1994; 1995, Vol 8; 1996, Vol 9; 1997, Vol 10; 1998, Vol 11.. |
| F1 | *Smilax glabra* | M025509 | Xu, Shuo; Shang, Ming-Ying; Liu, Guang-Xue; Xu, Feng; Wang, Xuan; Shou, Cheng-Chao; Cai, Shao-Qing; Molecules; vol. 18; 5; (2013); p. 5265 - 5287. |
| F1 | *Smilax glabra* | M025711 | Chien; Adam; Pharmazie; vol. 34; (1979); p. 841,842. |
| F1 | *Smilax glabra* | M025952 | Chen, Ting; Li, Jianxin; Cao, Jingsong; Xu, Qiang; Komatsu, Katsuko; Namba, Tsuneo; Planta Medica; vol. 65; 1; (1999); p. 56 - 59. |
| F1 | *Smilax glabra* | M026190 | Xu, Shuo; Shang, Ming-Ying; Liu, Guang-Xue; Xu, Feng; Wang, Xuan; Shou, Cheng-Chao; Cai, Shao-Qing; Molecules; vol. 18; 5; (2013); p. 5265 - 5287. |
| F1 | *Smilax glabra* | M026420 | Chen, Ting; Li, Jianxin; Cao, Jingsong; Xu, Qiang; Komatsu, Katsuko; Namba, Tsuneo; Planta Medica; vol. 65; 1; (1999); p. 56 - 59. |
| F1 | *Smilax glabra* | M026956 | Chen, Ting; Li, Jianxin; Cao, Jingsong; Xu, Qiang; Komatsu, Katsuko; Namba, Tsuneo; Planta Medica; vol. 65; 1; (1999); p. 56 - 59. |
| F1 | *Smilax glabra* | M027905 | Xu, Shuo; Shang, Ming-Ying; Liu, Guang-Xue; Xu, Feng; Wang, Xuan; Shou, Cheng-Chao; Cai, Shao-Qing; Molecules; vol. 18; 5; (2013); p. 5265 - 5287. |
| F1 | *Smilax glabra* | M028031 | Xu, Shuo; Shang, Ming-Ying; Liu, Guang-Xue; Xu, Feng; Wang, Xuan; Shou, Cheng-Chao; Cai, Shao-Qing; Molecules; vol. 18; 5; (2013); p. 5265 - 5287. |
| F1 | *Smilax glabra* | M028652 | Chen, Ting; Li, Jian-Xin; Xu, Qiang; Phytochemistry; vol. 53; 8; (2000); p. 1051 - 1055. |
| F1 | *Smilax glabra* | M029090 | Xu, Shuo; Shang, Ming-Ying; Liu, Guang-Xue; Xu, Feng; Wang, Xuan; Shou, Cheng-Chao; Cai, Shao-Qing; Molecules; vol. 18; 5; (2013); p. 5265 - 5287. |
| F1 | *Smilax glabra* | M030479 | Chen, Ting; Li, Jian-Xin; Xu, Qiang; Phytochemistry; vol. 53; 8; (2000); p. 1051 - 1055. |
| F1 | *Smilax glabra* | M030633 | Xu, Shuo; Shang, Ming-Ying; Liu, Guang-Xue; Xu, Feng; Wang, Xuan; Shou, Cheng-Chao; Cai, Shao-Qing; Molecules; vol. 18; 5; (2013); p. 5265 - 5287. |
| F1 | *Smilax glabra* | M030826 | Xu, Shuo; Shang, Ming-Ying; Liu, Guang-Xue; Xu, Feng; Wang, Xuan; Shou, Cheng-Chao; Cai, Shao-Qing; Molecules; vol. 18; 5; (2013); p. 5265 - 5287. |
| F1 | *Smilax glabra* | M031238 | Xu, Shuo; Shang, Ming-Ying; Liu, Guang-Xue; Xu, Feng; Wang, Xuan; Shou, Cheng-Chao; Cai, Shao-Qing; Molecules; vol. 18; 5; (2013); p. 5265 - 5287. |
| F1 | *Smilax glabra* | M032354 | Chen, Ting; Li, Jian-Xin; Xu, Qiang; Phytochemistry; vol. 53; 8; (2000); p. 1051 - 1055. |
| F1 | *Smilax glabra* | M033079 | Chen T, et al. Phytochemistry, 2000, 53 (8): 1051 |
| F1 | *Smilax glabra* | M033495 | Xu, Shuo; Shang, Ming-Ying; Liu, Guang-Xue; Xu, Feng; Wang, Xuan; Shou, Cheng-Chao; Cai, Shao-Qing; Molecules; vol. 18; 5; (2013); p. 5265 - 5287. |
| F1 | *Taraxacum mongolicum* | M018997 | Liu, Jifeng; Zhang, Nenling; Liu, Mengqi; Natural Product Research; vol. 28; 7; (2014); p. 420 - 423. |
| F1 | *Taraxacum mongolicum* | M027410 | (1) 阴健等. 中药现代研究与临床应用(1). 北京: 学苑出版社, 1993. (2) 国家中医药管理局《中华本草》编委会. 中华本草. Vol. 1-30 上海:上海科学技术出版社, 1999. |
| F1 | *Taraxacum mongolicum* | M029284 | (1) 阴健等. 中药现代研究与临床应用(1). 北京: 学苑出版社, 1993. (2) 国家中医药管理局《中华本草》编委会. 中华本草. Vol. 1-30 上海:上海科学技术出版社, 1999. |
| F2 | *Acorus tatarinowii* | M001683 | Hu; Feng; Planta Medica; vol. 66; 7; (2000); p. 662 - 664. |
| F2 | *Acorus tatarinowii* | M002120 | Tong, Xiao-Gang; Zhou, Li-Li; Wang, Yue-Hu; Xia, Chengfeng; Wang, Ye; Liang, Min; Hou, Fan-Fan; Cheng, Yong-Xian; Organic Letters; vol. 12; 8; (2010); p. 1844 - 1847. |
| F2 | *Acorus tatarinowii* | M003284 | Tong, Xiao-Gang; Wu, Gui-Sheng; Huang, Cheng-Gang; Lu, Qing; Wang, Yue-Hu; Long, Chun-Lin; Luo, Huai-Rong; Zhu, Hua-Jie; Cheng, Yong-Xian; Journal of Natural Products; vol. 73; 6; (2010); p. 1160 - 1163. |
| F2 | *Acorus tatarinowii* | M004136 | Hu; Feng; Planta Medica; vol. 66; 7; (2000); p. 662 - 664. |
| F2 | *Acorus tatarinowii* | M004726 | Tong, Xiao-Gang; Wu, Gui-Sheng; Huang, Cheng-Gang; Lu, Qing; Wang, Yue-Hu; Long, Chun-Lin; Luo, Huai-Rong; Zhu, Hua-Jie; Cheng, Yong-Xian; Journal of Natural Products; vol. 73; 6; (2010); p. 1160 - 1163. |
| F2 | *Acorus tatarinowii* | M004757 | Tong, Xiao-Gang; Wu, Gui-Sheng; Huang, Cheng-Gang; Lu, Qing; Wang, Yue-Hu; Long, Chun-Lin; Luo, Huai-Rong; Zhu, Hua-Jie; Cheng, Yong-Xian; Journal of Natural Products; vol. 73; 6; (2010); p. 1160 - 1163. |
| F2 | *Acorus tatarinowii* | M005571 | Hu; Feng; Planta Medica; vol. 66; 7; (2000); p. 662 - 664. |
| F2 | *Acorus tatarinowii* | M007938 | Tong, Xiao-Gang; Qiu, Bin; Luo, Gui-Fen; Zhang, Xiao-Fang; Cheng, Yong-Xian; Journal of Asian Natural Products Research; vol. 12; 6; (2010); p. 438 - 442. |
| F2 | *Acorus tatarinowii* | M008351 | Hu; Feng; Planta Medica; vol. 66; 7; (2000); p. 662 - 664. |
| F2 | *Acorus tatarinowii* | M009315 | Hu; Feng; Planta Medica; vol. 66; 7; (2000); p. 662 - 664. |
| F2 | *Acorus tatarinowii* | M010010 | Hu; Feng; Planta Medica; vol. 66; 7; (2000); p. 662 - 664. |
| F2 | *Acorus tatarinowii* | M010678 | Tong, Xiao-Gang; Wu, Gui-Sheng; Huang, Cheng-Gang; Lu, Qing; Wang, Yue-Hu; Long, Chun-Lin; Luo, Huai-Rong; Zhu, Hua-Jie; Cheng, Yong-Xian; Journal of Natural Products; vol. 73; 6; (2010); p. 1160 - 1163. |
| F2 | *Acorus tatarinowii* | M011835 | Tong, Xiao-Gang; Qiu, Bin; Luo, Gui-Fen; Zhang, Xiao-Fang; Cheng, Yong-Xian; Journal of Asian Natural Products Research; vol. 12; 6; (2010); p. 438 - 442. |
| F2 | *Acorus tatarinowii* | M013422 | Tong, Xiao-Gang; Wu, Gui-Sheng; Huang, Cheng-Gang; Lu, Qing; Wang, Yue-Hu; Long, Chun-Lin; Luo, Huai-Rong; Zhu, Hua-Jie; Cheng, Yong-Xian; Journal of Natural Products; vol. 73; 6; (2010); p. 1160 - 1163. |
| F2 | *Acorus tatarinowii* | M014572 | Tong, Xiao-Gang; Wu, Gui-Sheng; Huang, Cheng-Gang; Lu, Qing; Wang, Yue-Hu; Long, Chun-Lin; Luo, Huai-Rong; Zhu, Hua-Jie; Cheng, Yong-Xian; Journal of Natural Products; vol. 73; 6; (2010); p. 1160 - 1163. |
| F2 | *Acorus tatarinowii* | M015969 | Tong, Xiao-Gang; Wu, Gui-Sheng; Huang, Cheng-Gang; Lu, Qing; Wang, Yue-Hu; Long, Chun-Lin; Luo, Huai-Rong; Zhu, Hua-Jie; Cheng, Yong-Xian; Journal of Natural Products; vol. 73; 6; (2010); p. 1160 - 1163. |
| F2 | *Acorus tatarinowii* | M016485 | Ni, Gang; Shen, Zhu-Fang; Lu, Yang; Wang, Ying-Hong; Tang, Yan-Bo; Chen, Ruo-Yun; Hao, Zhi-You; Yu, De-Quan; Journal of Organic Chemistry; vol. 76; 7; (2011); p. 2056 - 2061. |
| F2 | *Acorus tatarinowii* | M016486 | Ni, Gang; Shen, Zhu-Fang; Lu, Yang; Wang, Ying-Hong; Tang, Yan-Bo; Chen, Ruo-Yun; Hao, Zhi-You; Yu, De-Quan; Journal of Organic Chemistry; vol. 76; 7; (2011); p. 2056 - 2061. |
| F2 | *Acorus tatarinowii* | M019595 | Tong, Xiao-Gang; Qiu, Bin; Luo, Gui-Fen; Zhang, Xiao-Fang; Cheng, Yong-Xian; Journal of Asian Natural Products Research; vol. 12; 6; (2010); p. 438 - 442. |
| F2 | *Acorus tatarinowii* | M022049 | Tong, Xiao-Gang; Qiu, Bin; Luo, Gui-Fen; Zhang, Xiao-Fang; Cheng, Yong-Xian; Journal of Asian Natural Products Research; vol. 12; 6; (2010); p. 438 - 442. |
| F2 | *Acorus tatarinowii* | M022649 | Hu; Feng; Planta Medica; vol. 66; 7; (2000); p. 662 - 664. |
| F2 | *Acorus tatarinowii* | M023463 | Hu; Feng; Planta Medica; vol. 66; 7; (2000); p. 662 - 664. |
| F2 | *Acorus tatarinowii* | M023483 | Tong, Xiao-Gang; Qiu, Bin; Luo, Gui-Fen; Zhang, Xiao-Fang; Cheng, Yong-Xian; Journal of Asian Natural Products Research; vol. 12; 6; (2010); p. 438 - 442. |
| F2 | *Acorus tatarinowii* | M023489 | Tong, Xiao-Gang; Wu, Gui-Sheng; Huang, Cheng-Gang; Lu, Qing; Wang, Yue-Hu; Long, Chun-Lin; Luo, Huai-Rong; Zhu, Hua-Jie; Cheng, Yong-Xian; Journal of Natural Products; vol. 73; 6; (2010); p. 1160 - 1163. |
| F2 | *Acorus tatarinowii* | M025563 | (1) 阴健等. 中药现代研究与临床应用(1). 北京: 学苑出版社, 1993. (2) 季宇彬等. 中药抗肿瘤有效成分药理与应用. 哈尔滨: 黑龙江科学技术出版社, 1995. (3) 宋振玉等. 中草药现代研究. 第2卷. 第28章 九里香. 333-361 北京: 北京医科大学中国协和医科大学联合出版社, 1996. (4) 黄西峰. 中国中药杂志, 1997, 22 (4): 247. (5) 孙文基等. 天然活性成分简明手册. 北京: 中国医药科技出版社, 1998. (6) 国家中医药管理局《中华本草》编委会. 中华本草. Vol. 1-30 上海:上海科学技术出版社, 1999. (7) Chen Y-C, et al. Planta Med, 2004, 70 (2): 174. (8) 欧明等. 简明中药成分手册. 北京: 中国医药科技出版社, 2003. |
| F2 | *Acorus tatarinowii* | M026030 | Hu; Feng; Planta Medica; vol. 66; 7; (2000); p. 662 - 664. |
| F2 | *Acorus tatarinowii* | M026378 | Tong, Xiao-Gang; Zhou, Li-Li; Wang, Yue-Hu; Xia, Chengfeng; Wang, Ye; Liang, Min; Hou, Fan-Fan; Cheng, Yong-Xian; Organic Letters; vol. 12; 8; (2010); p. 1844 - 1847. |
| F2 | *Acorus tatarinowii* | M027405 | Tong, Xiao-Gang; Wu, Gui-Sheng; Huang, Cheng-Gang; Lu, Qing; Wang, Yue-Hu; Long, Chun-Lin; Luo, Huai-Rong; Zhu, Hua-Jie; Cheng, Yong-Xian; Journal of Natural Products; vol. 73; 6; (2010); p. 1160 - 1163. |
| F2 | *Acorus tatarinowii* | M027634 | Tong, Xiao-Gang; Wu, Gui-Sheng; Huang, Cheng-Gang; Lu, Qing; Wang, Yue-Hu; Long, Chun-Lin; Luo, Huai-Rong; Zhu, Hua-Jie; Cheng, Yong-Xian; Journal of Natural Products; vol. 73; 6; (2010); p. 1160 - 1163. |
| F2 | *Acorus tatarinowii* | M028697 | Tong, Xiao-Gang; Qiu, Bin; Luo, Gui-Fen; Zhang, Xiao-Fang; Cheng, Yong-Xian; Journal of Asian Natural Products Research; vol. 12; 6; (2010); p. 438 - 442. |
| F2 | *Acorus tatarinowii* | M029614 | Tong, Xiao-Gang; Qiu, Bin; Luo, Gui-Fen; Zhang, Xiao-Fang; Cheng, Yong-Xian; Journal of Asian Natural Products Research; vol. 12; 6; (2010); p. 438 - 442. |
| F2 | *Acorus tatarinowii* | M030508 | Hu; Feng; Planta Medica; vol. 66; 7; (2000); p. 662 - 664. |
| F2 | *Acorus tatarinowii* | M030962 | Hu; Feng; Planta Medica; vol. 66; 7; (2000); p. 662 - 664. |
| F2 | *Acorus tatarinowii* | M032810 | Hu; Feng; Planta Medica; vol. 66; 7; (2000); p. 662 - 664. |
| F2 | *Angelica sinensis* | M000029 | (1) 阴健等. 中药现代研究与临床应用(1). 北京: 学苑出版社, 1993. (2) 江苏新医学院. 中药大辞典. 上海: 上海科学技术出版社, 1977. (3) 孙文基等. 天然活性成分简明手册. 北京: 中国医药科技出版社, 1998. (4) 国�抑幸揭┕芾砭帧吨谢静荨繁辔� 中华本草. Vol. 1-30 上海:上海科学技术出版社, 1999. (5) 易杨华. 中国药学杂志, 1990, 25 (10): 585. |
| F2 | *Angelica sinensis* | M000681 | (1) 阴健等. 中药现代研究与临床应用(1). 北京: 学苑出版社, 1993. (2) 张恩娟等. 中国中药杂志, 1993, 18 (1): 37. (3) 孙文基等. 天然活性成分简明手册. 北京: 中国医药科技出版社, 1998. (4) Dou H, et al. JNP, 2002, 65 (12): 1777. |
| F2 | *Angelica sinensis* | M001018 | (1) 阴健等. 中药现代研究与临床应用(1). 北京: 学苑出版社, 1993. (2) 孙文基等. 天然活性成分简明手册. 北京: 中国医药科技出版社, 1998. (3) 国家中医药管理局《中华本草》编委会. 中华本草. Vol. 1-30 上海:上海科学技术出版社, 1999. (4) Hua AN, et al. 南京中医药大学学报, 1990, 6 (3): 179. |
| F2 | *Angelica sinensis* | M001142 | Hon, Po-Ming; Lee, Chi-Ming; Choang, Tai Francis; Chui, Kuk-Ying; Wong, Henry N. C.; Phytochemistry (Elsevier); vol. 29; 4; (1990); p. 1189 - 1191. |
| F2 | *Angelica sinensis* | M001908 | 国家中医药管理局《中华本草》编委会. 中华本草. Vol. 1-30 上海:上海科学技术出版社, 1999 |
| F2 | *Angelica sinensis* | M002088 | 国家中医药管理局《中华本草》编委会. 中华本草. Vol. 1-30 上海:上海科学技术出版社, 1999 |
| F2 | *Angelica sinensis* | M002182 | Retrieved from CNPD |
| F2 | *Angelica sinensis* | M002360 | 阴健等. 中药现代研究与临床应用(1). 北京: 学苑出版社, 1993 |
| F2 | *Angelica sinensis* | M002590 | Deng, Shixin; Chen, Shao-Nong; Yao, Ping; Nikolic, Dejan; Van Breemen, Richard B.; Bolton, Judy L.; Fong, Harry H. S.; Farnsworth, Norman R.; Pauli, Guido F.; Journal of Natural Products; vol. 69; 4; (2006); p. 536 - 541. |
| F2 | *Angelica sinensis* | M002679 | 阴健等. 中药现代研究与临床应用(1). 北京: 学苑出版社, 1993 |
| F2 | *Angelica sinensis* | M003307 | (1) 阴健等. 中药现代研究与临床应用(1). 北京: 学苑出版社, 1993. (2) 孙文基等. 天然活性成分简明手册. 北京: 中国医药科技出版社, 1998. |
| F2 | *Angelica sinensis* | M003359 | Deng, Shixin; Chen, Shao-Nong; Yao, Ping; Nikolic, Dejan; Van Breemen, Richard B.; Bolton, Judy L.; Fong, Harry H. S.; Farnsworth, Norman R.; Pauli, Guido F.; Journal of Natural Products; vol. 69; 4; (2006); p. 536 - 541. |
| F2 | *Angelica sinensis* | M003872 | (1) 阴健等. 中药现代研究与临床应用(1). 北京: 学苑出版社, 1993. (2) 饶高雄等. 中国中药杂志, 1995, 20 (12): 740. (3) 顾志平等. 中国中药杂志, 1997, 22 (1): 40. (4) 孙文基等. 天然活性成分简明手册. 北京: �泄揭┛萍汲霭嫔� 1998. (5) Buckingham J(Executive Editor): et al. Dictionary of Natural Products, Vol 1-7, Chapman & Hall, London, 1994; 1995, Vol 8; 1996, Vol 9; 1997, Vol 10; 1998, Vol 11.. (6) Morikawa T, et al. JNP, 2002, 65 (10): 1468. |
| F2 | *Angelica sinensis* | M003889 | (1) 阴健等. 中药现代研究与临床应用(1). 北京: 学苑出版社, 1993. (2) 季宇彬等. 中药抗肿瘤有效成分药理与应用. 哈尔滨: 黑龙江科学技术出版社, 1995. (3) 宋振玉等. 中草药现代研究. 第2卷. 第28章 九里香. 333-361 北京: 北京医科大学中国协和医科大学联合出版社, 1996. (4) 张印俊等. 药学学报, 1998, 33 (11): 836. (5) 饶高雄等. 中国中药杂志, 1996, 21 (8): 482. (6) 宋蔚等. 中国中药杂志, 1997, 22 (6): 359. (7) 王栋等. 中国中药杂志, 1997, 22 (8): 486. (8) 孙文基等. 天然�钚猿煞旨蛎魇植� 北京: 中国医药科技出版社, 1998. (9) 国家中医药管理局《中华本草》编委会. 中华本草. Vol. 1-30 上海:上海科学技术出版社, 1999. (10) El-Khrisy EAM, et al.Chem. Abstr., 1992, 117, 86802g. (11) Uno T, et al.Chem. Abstr., 1972, 76, 56567b. (12) Chaurasia N, et al.Chem. Abstr., 1986, 104, 85467w. (13) Khvorost PP, et al.Chem. Abstr., 1981, 94, 136170d. (14) Wu PL, et al. Chem Pharm Bull, 2005, 53 (1): 56. (15) Syu WJ, et al. JNP, 2001, 64 (9): 1232. (16) Yun BS, et al. JNP, 2001, 64 (9): 1238. (17) YUAN Z, et al. Chem Pharm Bull, 2002, 50 (1): 73. (18) MORIKAWA T, et al. Chem Pharm Bull, 2003, 51 (1): 62. (19) WANG N-H, et al. Chem Pharm Bull, 2003, 51 (1): 68. (20) WU T-S, et al. Chem [...truncated...] |
| F2 | *Angelica sinensis* | M004163 | Deng, Shixin; Chen, Shao-Nong; Yao, Ping; Nikolic, Dejan; Van Breemen, Richard B.; Bolton, Judy L.; Fong, Harry H. S.; Farnsworth, Norman R.; Pauli, Guido F.; Journal of Natural Products; vol. 69; 4; (2006); p. 536 - 541. |
| F2 | *Angelica sinensis* | M004675 | Deng, Shixin; Chen, Shao-Nong; Yao, Ping; Nikolic, Dejan; Van Breemen, Richard B.; Bolton, Judy L.; Fong, Harry H. S.; Farnsworth, Norman R.; Pauli, Guido F.; Journal of Natural Products; vol. 69; 4; (2006); p. 536 - 541. |
| F2 | *Angelica sinensis* | M005974 | (1) 阴健等. 中药现代研究与临床应用(1). 北京: 学苑出版社, 1993. (2) 国家中医药管理局《中华本草》编委会. 中华本草. Vol. 1-30 上海:上海科学技术出版社, 1999. |
| F2 | *Angelica sinensis* | M006085 | Deng, Shixin; Chen, Shao-Nong; Yao, Ping; Nikolic, Dejan; Van Breemen, Richard B.; Bolton, Judy L.; Fong, Harry H. S.; Farnsworth, Norman R.; Pauli, Guido F.; Journal of Natural Products; vol. 69; 4; (2006); p. 536 - 541. |
| F2 | *Angelica sinensis* | M006222 | Deng, Shixin; Chen, Shao-Nong; Yao, Ping; Nikolic, Dejan; Van Breemen, Richard B.; Bolton, Judy L.; Fong, Harry H. S.; Farnsworth, Norman R.; Pauli, Guido F.; Journal of Natural Products; vol. 69; 4; (2006); p. 536 - 541. |
| F2 | *Angelica sinensis* | M006375 | (1) 阴健等. 中药现代研究与临床应用(1). 北京: 学苑出版社, 1993. (2) 江苏新医学院. 中药大辞典. 上海: 上海科学技术出版社, 1977. (3) 孙文基等. 天然活性成分简明手册. 北京: 中国医药科技出版社, 1998. (4) 国�抑幸揭┕芾砭帧吨谢静荨繁辔� 中华本草. Vol. 1-30 上海:上海科学技术出版社, 1999. (5) 刈米达夫著, 杨本文译. 植物化学, 科学出版社, 北京, 1985. |
| F2 | *Angelica sinensis* | M006850 | (1) 阴健等. 中药现代研究与临床应用(1). 北京: 学苑出版社, 1993. (2) 李其生等. 中国中药杂志, 1993, 18 (8): 486. (3) 肖永庆等. 中国中药杂志, 1995, 20 (5): 294. (4) 肖永庆等. 中国中药杂志, 1995, 20 (7): 423. (5) 饶高雄等. 中国中药杂志, 1995, 20 (12): 740. (6) 顾志平等. 中国中药杂志, 1997, 22 (1): 40. (7) 韦松等. 中国中药杂志, 1997, 22 (5): 293. (8) 宋蔚等. 中国中药杂志, 1997, 22 (6): 359. (9) 梁培瑜等. 中国中药杂志, 1998, 23 (1): 39. (10) 王海燕等. 中国中药杂志, 1998, 23 (3): 167. (11) 国家中医药管理局《中华本草》编委会. 中华本草. Vol. 1-30 上海:上海科学技术出版社, 1999. (12) Buckingham J(Executive Editor): et al. Dictionary of Natural Products, Vol 1-7, Chapman & Hall, London, 1994; 1995, Vol 8; 1996, Vol 9; 1997, Vol 10; 1998, Vol 11.. (13) Sautour M, et al. Chem Pharm Bull, 2004, 52 (10): 1235. (14) BEGUM S, et al. Chem Pharm Bull, 2003, 51 (2): 134. (15) Calixto JB, et al. Planta Med, 2003, 69 (11): 973. (16) Lin WY, et al. Planta Med, 2003, 69, 757. |
| F2 | *Angelica sinensis* | M006919 | (1) 阴健等. 中药现代研究与临床应用(1). 北京: 学苑出版社, 1993. (2) 季宇彬等. 中药抗肿瘤有效成分药理与应用. 哈尔滨: 黑龙江科学技术出版社, 1995. (3) 李彤梅等. 药学学报, 1998, 33 (8): 591. (4) 傅宏征等. 中国药学杂志, 1998, 33 (3): 140. (5) 周燕生等. 中国中药杂志, 1994, 19 (3): 162. (6) 徐丽珍等. 中国中药杂志, 1994, 19 (11): 675. (7) 陈妙华等. 中国中药杂志, 1993, 18 (7): 424. (8) 石磊等. 中国中药杂志, 1997, 22 (12): 743. (9) 徐丽萍等. 中国中药杂志, 1998, 23 (5): 293. (10) 孙文基等. 天然活性成分简明手册. 北京: 中国医药科技出版社, 1998. (11) 国家中医药管理局《中华本草》编委会. 中华本草. Vol. 1-30 上海:上海科学技术出版社, 1999. (12) Zheng Y, et al. JNP, 2004, 67 (9): 1617. (13) 欧明等. 简明中药成分手册. 北京: 中国医药科技出版社, 2003. |
| F2 | *Angelica sinensis* | M007255 | (1) 阴健等. 中药现代研究与临床应用(1). 北京: 学苑出版社, 1993. (2) 陈若云等. 药学学报, 1995, 30 (7): 526. (3) 国家中医药管理局《中华本草》编委会. 中华本草. Vol. 1-30 上海:上海科学技术出版社, 1999. (4) Calixto JB, et al. Planta Med, 2003, 69 (11): 973. |
| F2 | *Angelica sinensis* | M007385 | 阴健等. 中药现代研究与临床应用(1). 北京: 学苑出版社, 1993 |
| F2 | *Angelica sinensis* | M007418 | 国家中医药管理局《中华本草》编委会. 中华本草. Vol. 1-30 上海:上海科学技术出版社, 1999 |
| F2 | *Angelica sinensis* | M007760 | 黄伟晖等. 药学学报, 2003, 38 (9): 680 |
| F2 | *Angelica sinensis* | M008893 | 阴健等. 中药现代研究与临床应用(1). 北京: 学苑出版社, 1993 |
| F2 | *Angelica sinensis* | M008923 | Deng, Shixin; Chen, Shao-Nong; Yao, Ping; Nikolic, Dejan; Van Breemen, Richard B.; Bolton, Judy L.; Fong, Harry H. S.; Farnsworth, Norman R.; Pauli, Guido F.; Journal of Natural Products; vol. 69; 4; (2006); p. 536 - 541. |
| F2 | *Angelica sinensis* | M009104 | (1) 阴健等. 中药现代研究与临床应用(1). 北京: 学苑出版社, 1993. (2) 国家中医药管理局《中华本草》编委会. 中华本草. Vol. 1-30 上海:上海科学技术出版社, 1999. (3) 吴知行等. 中国药科大学学报, 1994, 25 (4): 202. |
| F2 | *Angelica sinensis* | M009554 | (1) 阴健等. 中药现代研究与临床应用(1). 北京: 学苑出版社, 1993. (2) 张卫东等. 药学学报, 1992, 27 (9): 670. (3) 孙文基等. 天然活性成分简明手册. 北京: 中国医药科技出版社, 1998. (4) 国家中医药管理局《中华本草》编委会. 中华本草. Vol. 1-30 上海:上海科学技术出版社, 1999. (5) LI C-Y, et al. Chem Pharm Bull, 2002, 50 (10): 1305. (6) Li CY, et al. JNP, 2002, 65 (10): 1452. |
| F2 | *Angelica sinensis* | M009761 | (1) 李其生等. 中国中药杂志, 1993, 18 (8): 486. (2) 国家中医药管理局《中华本草》编委会. 中华本草. Vol. 1-30 上海:上海科学技术出版社, 1999. (3) 于澍仁等. 药学学报, 1984, 19 (8): 566. (4) Ozaki Y, et al. 药学杂志(日), 1989, 109 (6): 402. (5) Chem. Abstr., 1989, 111, P239574n. (6) Buckingham J(Executive Editor): et al. Dictionary of Natural Products, Vol 1-7, Chapman & Hall, London, 1994; 1995, Vol 8; 1996, Vol 9; 1997, Vol 10; 1998, Vol 11.. |
| F2 | *Angelica sinensis* | M009947 | Deng, Shixin; Chen, Shao-Nong; Yao, Ping; Nikolic, Dejan; Van Breemen, Richard B.; Bolton, Judy L.; Fong, Harry H. S.; Farnsworth, Norman R.; Pauli, Guido F.; Journal of Natural Products; vol. 69; 4; (2006); p. 536 - 541. |
| F2 | *Angelica sinensis* | M010181 | 阴健等. 中药现代研究与临床应用(1). 北京: 学苑出版社, 1993 |
| F2 | *Angelica sinensis* | M010230 | 阴健等. 中药现代研究与临床应用(1). 北京: 学苑出版社, 1993 |
| F2 | *Angelica sinensis* | M010372 | (1) 阴健等. 中药现代研究与临床应用(1). 北京: 学苑出版社, 1993. (2) 宋振玉等. 中草药现代研究. 第3卷. 第33章 沉香. 1-21 北京: 北京医科大学中国协和医科大学联合出版社, 1997. (3) AWALE S, et al. Chem Pharm Bull, 2005, 53 (6): 710. (4) LEU Y-L, et al. Chem Pharm Bull, 2005, 53 (7): 853. |
| F2 | *Angelica sinensis* | M010398 | (1) 阴健等. 中药现代研究与临床应用(1). 北京: 学苑出版社, 1993. (2) 孙文基等. 天然活性成分简明手册. 北京: 中国医药科技出版社, 1998. (3) 国家中医药管理局《中华本草》编委会. 中华本草. Vol. 1-30 上海:上海科学技术出版社, 1999. (4) Buckingham J(Executive Editor): et al. Dictionary of Natural Products, Vol 1-7, Chapman & Hall, London, 1994; 1995, Vol 8; 1996, Vol 9; 1997, Vol 10; 1998, Vol 11.. |
| F2 | *Angelica sinensis* | M010708 | (1) 阴健等. 中药现代研究与临床应用(1). 北京: 学苑出版社, 1993. (2) 毛士龙等. 药学学报, 1996, 31 (1): 118. (3) Yoshikawa M, et al. JNP, 2003, 66 (7): 922. (4) Carcache-Blanco EJ, et al. JNP, 2003, 67 (1): 126. (5) Faizi S, et al. Planta Med, 2003, 69, 350. |
| F2 | *Angelica sinensis* | M011000 | (1) 阴健等. 中药现代研究与临床应用(1). 北京: 学苑出版社, 1993. (2) 国家中医药管理局《中华本草》编委会. 中华本草. Vol. 1-30 上海:上海科学技术出版社, 1999. |
| F2 | *Angelica sinensis* | M011318 | 黄伟晖等. 药学学报, 2003, 38 (9): 680 |
| F2 | *Angelica sinensis* | M012564 | (1) 阴健等. 中药现代研究与临床应用(1). 北京: 学苑出版社, 1993. (2) 孙文基等. 天然活性成分简明手册. 北京: 中国医药科技出版社, 1998. (3) 国家中医药管理局《中华本草》编委会. 中华本草. Vol. 1-30 上海:上海科学技术出版社, 1999. |
| F2 | *Angelica sinensis* | M012697 | 阴健等. 中药现代研究与临床应用(1). 北京: 学苑出版社, 1993 |
| F2 | *Angelica sinensis* | M012774 | 阴健等. 中药现代研究与临床应用(1). 北京: 学苑出版社, 1993 |
| F2 | *Angelica sinensis* | M012885 | Deng, Shixin; Chen, Shao-Nong; Yao, Ping; Nikolic, Dejan; Van Breemen, Richard B.; Bolton, Judy L.; Fong, Harry H. S.; Farnsworth, Norman R.; Pauli, Guido F.; Journal of Natural Products; vol. 69; 4; (2006); p. 536 - 541. |
| F2 | *Angelica sinensis* | M013716 | (1) 阴健等. 中药现代研究与临床应用(1). 北京: 学苑出版社, 1993. (2) 孙文基等. 天然活性成分简明手册. 北京: 中国医药科技出版社, 1998. |
| F2 | *Angelica sinensis* | M013833 | Buckingham J(Executive Editor): et al. Dictionary of Natural Products, Vol 1-7, Chapman & Hall, London, 1994; 1995, Vol 8; 1996, Vol 9; 1997, Vol 10; 1998, Vol 11. |
| F2 | *Angelica sinensis* | M014504 | 阴健等. 中药现代研究与临床应用(1). 北京: 学苑出版社, 1993 |
| F2 | *Angelica sinensis* | M014560 | 阴健等. 中药现代研究与临床应用(1). 北京: 学苑出版社, 1993 |
| F2 | *Angelica sinensis* | M014760 | (1) 汪纪武等. 植物药有效成分手册. 北京: 人民卫生出版社, 1986. (2) 阴健等. 中药现代研究与临床应用(1). 北京: 学苑出版社, 1993. (3) Shin S, et al. Planta Med, 2004, 70, 1090. |
| F2 | *Angelica sinensis* | M015010 | 阴健等. 中药现代研究与临床应用(1). 北京: 学苑出版社, 1993 |
| F2 | *Angelica sinensis* | M015284 | 国家中医药管理局《中华本草》编委会. 中华本草. Vol. 1-30 上海:上海科学技术出版社, 1999 |
| F2 | *Angelica sinensis* | M015310 | 阴健等. 中药现代研究与临床应用(1). 北京: 学苑出版社, 1993 |
| F2 | *Angelica sinensis* | M015874 | (1) 阴健等. 中药现代研究与临床应用(1). 北京: 学苑出版社, 1993. (2) 国家中医药管理局《中华本草》编委会. 中华本草. Vol. 1-30 上海:上海科学技术出版社, 1999. |
| F2 | *Angelica sinensis* | M016844 | Yang, Nian-Yun; Zhou, Gui-Sheng; Tang, Yu-Ping; Yan, Hui; Guo, Sheng; Liu, Pei; Duan, Jin-Ao; Song, Bing-Sheng; He, Zi-Qing; Fitoterapia; vol. 82; 4; (2011); p. 692 - 695. |
| F2 | *Angelica sinensis* | M016845 | Yang, Nian-Yun; Zhou, Gui-Sheng; Tang, Yu-Ping; Yan, Hui; Guo, Sheng; Liu, Pei; Duan, Jin-Ao; Song, Bing-Sheng; He, Zi-Qing; Fitoterapia; vol. 82; 4; (2011); p. 692 - 695. |
| F2 | *Angelica sinensis* | M019624 | Short Survey; Yang, Nian-Yun; Jiang, Shu; Shang, Er-Xin; Tang, Yu-Ping; Duan, Jin-Ao; Journal of Chemical Research; vol. 36; 11; (2012); p. 647 - 647. |
| F2 | *Angelica sinensis* | M019865 | Trivedi et al.; Cesko-Slovenska Farmacie; vol. 15; (1966); p. 206,209. |
| F2 | *Angelica sinensis* | M020237 | National Dong Hwa University; Chiou, Tzyy-Wen; Harn, Horng-Jyh; Lin, Shinn-Zong; EP2606883; A1; (2013). |
| F2 | *Angelica sinensis* | M020290 | Xie, Jing-Jing; Lu, Jia; Qian, Zheng-Ming; Yu; Duan, Jin-Ao; Li, Shao-Ping; Molecules; vol. 14; 1; (2009); p. 555 - 565. |
| F2 | *Angelica sinensis* | M021266 | Li, Xing-Nuo; Chen, Yuan-Yi; Cheng, Dong-Ping; Tong, Sheng-Qiang; Yan, Ji-Zhong; Qu, Hai-Bin; Natural Product Research; vol. 26; 19; (2012); p. 1782 - 1786,5.; Li, Xing-Nuo; Chen, Yuan-Yi; Cheng, Dong-Ping; Tong, Sheng-Qiang; Qu, Hai-Bin; Yan, Ji-Zhong; Natural Product Research; vol. 26; 19; (2012); p. 1782 - 1786. |
| F2 | *Angelica sinensis* | M022026 | 江苏新医学院. 中药大辞典. 上海: 上海科学技术出版社, 1977 |
| F2 | *Angelica sinensis* | M022322 | 阴健等. 中药现代研究与临床应用(1). 北京: 学苑出版社, 1993 |
| F2 | *Angelica sinensis* | M023044 | (1) 阴健等. 中药现代研究与临床应用(1). 北京: 学苑出版社, 1993. (2) 江苏新医学院. 中药大辞典. 上海: 上海科学技术出版社, 1977. (3) 国家中医药管理局《中华本草》编委会. 中华本草. Vol. 1-30 上海:上海科学技术出版社, 1999. |
| F2 | *Angelica sinensis* | M023048 | 阴健等. 中药现代研究与临床应用(1). 北京: 学苑出版社, 1993 |
| F2 | *Angelica sinensis* | M024367 | Deng, Shixin; Chen, Shao-Nong; Yao, Ping; Nikolic, Dejan; Van Breemen, Richard B.; Bolton, Judy L.; Fong, Harry H. S.; Farnsworth, Norman R.; Pauli, Guido F.; Journal of Natural Products; vol. 69; 4; (2006); p. 536 - 541. |
| F2 | *Angelica sinensis* | M024426 | 阴健等. 中药现代研究与临床应用(1). 北京: 学苑出版社, 1993 |
| F2 | *Angelica sinensis* | M024856 | (1) 江苏新医学院. 中药大辞典. 上海: 上海科学技术出版社, 1977. (2) 国家中医药管理局《中华本草》编委会. 中华本草. Vol. 1-30 上海:上海科学技术出版社, 1999. |
| F2 | *Angelica sinensis* | M025149 | Deng, Shixin; Chen, Shao-Nong; Yao, Ping; Nikolic, Dejan; Van Breemen, Richard B.; Bolton, Judy L.; Fong, Harry H. S.; Farnsworth, Norman R.; Pauli, Guido F.; Journal of Natural Products; vol. 69; 4; (2006); p. 536 - 541. |
| F2 | *Angelica sinensis* | M025258 | (1) 阴健等. 中药现代研究与临床应用(1). 北京: 学苑出版社, 1993. (2) 江苏新医学院. 中药大辞典. 上海: 上海科学技术出版社, 1977. (3) 国家中医药管理局《中华本草》编委会. 中华本草. Vol. 1-30 上海:上海科学技术出版社, 1999. |
| F2 | *Angelica sinensis* | M025330 | Deng, Shixin; Chen, Shao-Nong; Yao, Ping; Nikolic, Dejan; Van Breemen, Richard B.; Bolton, Judy L.; Fong, Harry H. S.; Farnsworth, Norman R.; Pauli, Guido F.; Journal of Natural Products; vol. 69; 4; (2006); p. 536 - 541. |
| F2 | *Angelica sinensis* | M025438 | 苏东敏等. 药学学报, 2005, 40 (2): 141 |
| F2 | *Angelica sinensis* | M025532 | Deng, Shixin; Chen, Shao-Nong; Yao, Ping; Nikolic, Dejan; Van Breemen, Richard B.; Bolton, Judy L.; Fong, Harry H. S.; Farnsworth, Norman R.; Pauli, Guido F.; Journal of Natural Products; vol. 69; 4; (2006); p. 536 - 541. |
| F2 | *Angelica sinensis* | M025882 | Deng, Shixin; Chen, Shao-Nong; Yao, Ping; Nikolic, Dejan; Van Breemen, Richard B.; Bolton, Judy L.; Fong, Harry H. S.; Farnsworth, Norman R.; Pauli, Guido F.; Journal of Natural Products; vol. 69; 4; (2006); p. 536 - 541. |
| F2 | *Angelica sinensis* | M026459 | Deng, Shixin; Chen, Shao-Nong; Yao, Ping; Nikolic, Dejan; Van Breemen, Richard B.; Bolton, Judy L.; Fong, Harry H. S.; Farnsworth, Norman R.; Pauli, Guido F.; Journal of Natural Products; vol. 69; 4; (2006); p. 536 - 541. |
| F2 | *Angelica sinensis* | M026949 | Deng, Shixin; Chen, Shao-Nong; Yao, Ping; Nikolic, Dejan; Van Breemen, Richard B.; Bolton, Judy L.; Fong, Harry H. S.; Farnsworth, Norman R.; Pauli, Guido F.; Journal of Natural Products; vol. 69; 4; (2006); p. 536 - 541. |
| F2 | *Angelica sinensis* | M027757 | 阴健等. 中药现代研究与临床应用(1). 北京: 学苑出版社, 1993 |
| F2 | *Angelica sinensis* | M027787 | Deng, Shixin; Chen, Shao-Nong; Yao, Ping; Nikolic, Dejan; Van Breemen, Richard B.; Bolton, Judy L.; Fong, Harry H. S.; Farnsworth, Norman R.; Pauli, Guido F.; Journal of Natural Products; vol. 69; 4; (2006); p. 536 - 541. |
| F2 | *Angelica sinensis* | M028027 | Deng, Shixin; Chen, Shao-Nong; Yao, Ping; Nikolic, Dejan; Van Breemen, Richard B.; Bolton, Judy L.; Fong, Harry H. S.; Farnsworth, Norman R.; Pauli, Guido F.; Journal of Natural Products; vol. 69; 4; (2006); p. 536 - 541. |
| F2 | *Angelica sinensis* | M028061 | (1) 江苏新医学院. 中药大辞典. 上海: 上海科学技术出版社, 1977. (2) 宋振玉等. 中草药现代研究. 第2卷. 第17章 当归. 1-51 北京: 北京医科大学中国协和医科大学联合出版社, 1996. (3) 宋振玉等. 中草药现代研究. ��卷. 第18章 旱芹. 52-70 北京: 北京医科大学中国协和医科大学联合出版社, 1996. |
| F2 | *Angelica sinensis* | M028851 | 阴健等. 中药现代研究与临床应用(1). 北京: 学苑出版社, 1993 |
| F2 | *Angelica sinensis* | M029284 | (1) 阴健等. 中药现代研究与临床应用(1). 北京: 学苑出版社, 1993. (2) 国家中医药管理局《中华本草》编委会. 中华本草. Vol. 1-30 上海:上海科学技术出版社, 1999. |
| F2 | *Angelica sinensis* | M029558 | 阴健等. 中药现代研究与临床应用(1). 北京: 学苑出版社, 1993 |
| F2 | *Angelica sinensis* | M029937 | Deng, Shixin; Chen, Shao-Nong; Yao, Ping; Nikolic, Dejan; Van Breemen, Richard B.; Bolton, Judy L.; Fong, Harry H. S.; Farnsworth, Norman R.; Pauli, Guido F.; Journal of Natural Products; vol. 69; 4; (2006); p. 536 - 541. |
| F2 | *Angelica sinensis* | M031075 | (1) 阴健等. 中药现代研究与临床应用(1). 北京: 学苑出版社, 1993. (2) 孙文基等. 天然活性成分简明手册. 北京: 中国医药科技出版社, 1998. (3) 国家中医药管理局《中华本草》编委会. 中华本草. Vol. 1-30 上海:上海科学技术出版社, 1999. (4) Buckingham J(Executive Editor): et al. Dictionary of Natural Products, Vol 1-7, Chapman & Hall, London, 1994; 1995, Vol 8; 1996, Vol 9; 1997, Vol 10; 1998, Vol 11.. (5) CHAN Y-Y, et al. Chem Pharm Bull, 2005, 53 (7): 836. |
| F2 | *Angelica sinensis* | M031093 | 阴健等. 中药现代研究与临床应用(1). 北京: 学苑出版社, 1993 |
| F2 | *Angelica sinensis* | M031155 | Li, Xing-Nuo; Chen, Yuan-Yi; Cheng, Dong-Ping; Tong, Sheng-Qiang; Yan, Ji-Zhong; Qu, Hai-Bin; Natural Product Research; vol. 26; 19; (2012); p. 1782 - 1786,5.; Li, Xing-Nuo; Chen, Yuan-Yi; Cheng, Dong-Ping; Tong, Sheng-Qiang; Qu, Hai-Bin; Yan, Ji-Zhong; Natural Product Research; vol. 26; 19; (2012); p. 1782 - 1786. |
| F2 | *Angelica sinensis* | M032127 | (1) 阴健等. 中药现代研究与临床应用(1). 北京: 学苑出版社, 1993. (2) 国家中医药管理局《中华本草》编委会. 中华本草. Vol. 1-30 上海:上海科学技术出版社, 1999. |
| F2 | *Angelica sinensis* | M032515 | Deng, Shixin; Chen, Shao-Nong; Yao, Ping; Nikolic, Dejan; Van Breemen, Richard B.; Bolton, Judy L.; Fong, Harry H. S.; Farnsworth, Norman R.; Pauli, Guido F.; Journal of Natural Products; vol. 69; 4; (2006); p. 536 - 541. |
| F2 | *Angelica sinensis* | M033251 | 阴健等. 中药现代研究与临床应用(1). 北京: 学苑出版社, 1993 |
| F2 | *Angelica sinensis* | M033995 | 阴健等. 中药现代研究与临床应用(1). 北京: 学苑出版社, 1993 |
| F2 | *Baphicacanthus cusia* | M001362 | (1) 邹继纯等. 药学学报, 1985, 20 (1): 45. (2) 国家中医药管理局《中华本草》编委会. 中华本草. Vol. 1-30 上海:上海科学技术出版社, 1999. |
| F2 | *Baphicacanthus cusia* | M001859 | (1) 江苏新医学院. 中药大辞典. 上海: 上海科学技术出版社, 1977. (2) 王建华等. 中国药学杂志, 1994, 29 (5): 268. (3) 孙文基等. 天然活性成分简明手册. 北京: 中国医药科技出版社, 1998. (4) 国家中医药管理局《�谢静荨繁辔� 中华本草. Vol. 1-30 上海:上海科学技术出版社, 1999. (5) Wu TS, et al. Phytochemistry, 1999, 52, 901. (6) Chumkaew P, et al. Chem Pharm Bull, 2005, 53 (1): 95. (7) Tanaka T, et al. Chem Pharm Bull, 2004, 52 (10): 1242. (8) Mutai C, et al. Phytochemistry, 2004, 65, 1159. (9) Tanaka R, et al. Planta Med, 2004, 70, 1234. (10) TORIUMI Y, et al. Chem Pharm Bull, 2003, 51 (1): 89. (11) Calixto JB, et al. Planta Med, 2003, 69 (11): 973. (12) NAKANISHI T, et al. Chem Pharm Bull, 2005, 53 (2): 229. (13) KIEM PV, et al. Chem Pharm Bull, 2005, 53 (4): 428. (14) Gutierrez-Lugo M-T, et al. Planta Med, 2004, 70 (3): 263. (15) Madureira AM, et al. Planta Med, 2004, 70 (9): 828. (16) Puapairoj P, et al. Planta Med, 2005, 71 (3): 208. (17) Li XQ, et al. Planta Med, 2003, 69, 356. (18) Chaaib F, et al. Planta Med, 2003, 69, 316. |
| F2 | *Baphicacanthus cusia* | M006509 | (1) 阴健等. 中药现代研究与临床应用(1). 北京: 学苑出版社, 1993. (2) 孙文基等. 天然活性成分简明手册. 北京: 中国医药科技出版社, 1998. (3) 国家中医药管理局《中华本草》编委会. 中华本草. Vol. 1-30 上海:上海科学技术出版社, 1999. (4) Heinemann C, et al. Planta Med, 2004, 70 (5): 385. (5) Oberthür C, et al. Planta Med, 2004, 70 (7): 642. |
| F2 | *Baphicacanthus cusia* | M007555 | (1) 阴健等. 中药现代研究与临床应用(1). 北京: 学苑出版社, 1993. (2) 陈妙华等. 中国中药杂志, 1993, 18 (7): 424. (3) 金秀莲等. 中国中药杂志, 1994, 19 (11): 695. (4) 倪慕云等. 中国中药杂志, 1989, 14 (7): 41. (5) 孙文基等. 天然活性成分简明手册. 北京: 中国医药科技出版社, 1998. (6) 国家中医药管理局《中华本草》编委会. 中华本草. Vol. 1-30 上海:上海科学技术出版社, 1999. (7) Tanaka T, et al. Chem Pharm Bull, 2004, 52 (10): 1242. (8) Kanchanapoom T, et al. Chem Pharm Bull, 2004, 52 (8): 980. (9) Pinar ?ahin F, et al. Phytochemistry, 2004, 65, 2095. (10) HARPUT US, et al. Chem Pharm Bull, 2002, 50 (6): 869. (11) ONO M, et al. Chem Pharm Bull, 2005, 53 (9): 1175. (12) He ZD, et al. JNP, 2003, 66 (6): 851. (13) Lin L-C, et al. Planta Med, 2004, 70 (1): 50. (14) K?rm?z?bekmez H, et al. Planta Med, 2004, 70 (8): 711. (15) Pu XP, et al. Planta Med, 2003, 69, 65. (16) Martin-Nizard F, et al. Planta Med, 2003, 69, 207. (17) Budzianowska A, et al. Planta Med, 2004, 70 (9): 834. (18) Abougazar H, et al. Planta Med, 2003, 69, 814. (19) Boje K, et al. Planta Med, 2003, 69, 820. |
| F2 | *Baphicacanthus cusia* | M008101 | (1) 季宇彬等. 中药抗肿瘤有效成分药理与应用. 哈尔滨: 黑龙江科学技术出版社, 1995. (2) 孙文基等. 天然活性成分简明手册. 北京: 中国医药科技出版社, 1998. (3) 国家中医药管理局《中华本草》编委会. 中华本草. Vol. 1-30 上海:上海科学技术出版社, 1999. (4) 欧明等. 简明中药成分手册. 北京: 中国医药科技出版社, 2003. |
| F2 | *Baphicacanthus cusia* | M010268 | Tanaka T, et al. Chem Pharm Bull, 2004, 52 (10): 1242 |
| F2 | *Baphicacanthus cusia* | M013248 | (1) 国家中医药管理局《中华本草》编委会. 中华本草(精选本上下册). 上海: 上海科学技术出版社, 1998. (2) Leclerc S, et al. J. Biol. Chem., 2001, 276 (1): 251. |
| F2 | *Baphicacanthus cusia* | M013402 | Tanaka T, et al. Chem Pharm Bull, 2004, 52 (10): 1242 |
| F2 | *Baphicacanthus cusia* | M015729 | Tanaka T, et al. Chem Pharm Bull, 2004, 52 (10): 1242 |
| F2 | *Baphicacanthus cusia* | M026404 | Tanaka T, et al. Chem Pharm Bull, 2004, 52 (10): 1242 |
| F2 | *Baphicacanthus cusia* | M028269 | Tanaka T, et al. Chem Pharm Bull, 2004, 52 (10): 1242 |
| F2 | *Baphicacanthus cusia* | M031379 | (1) 李典鹏等. 药学学报, 1999, 34 (1): 43. (2) Tanaka T, et al. Chem Pharm Bull, 2004, 52 (10): 1242. (3) MIN B-S, et al. Chem Pharm Bull, 2003, 51 (11): 1322. |
| F2 | *Boswellia carterii* | M000881 | Morikawa, Toshio; Oominami, Hideo; Matsuda, Hisashi; Yoshikawa, Masayuki; Chemical and Pharmaceutical Bulletin; vol. 58; 11; (2010); p. 1541 - 1544. |
| F2 | *Boswellia carterii* | M000997 | Morikawa, Toshio; Oominami, Hideo; Matsuda, Hisashi; Yoshikawa, Masayuki; Chemical and Pharmaceutical Bulletin; vol. 58; 11; (2010); p. 1541 - 1544. |
| F2 | *Boswellia carterii* | M001215 | Morikawa, Toshio; Oominami, Hideo; Matsuda, Hisashi; Yoshikawa, Masayuki; Chemical and Pharmaceutical Bulletin; vol. 58; 11; (2010); p. 1541 - 1544. |
| F2 | *Boswellia carterii* | M001790 | Morikawa, Toshio; Oominami, Hideo; Matsuda, Hisashi; Yoshikawa, Masayuki; Chemical and Pharmaceutical Bulletin; vol. 58; 11; (2010); p. 1541 - 1544. |
| F2 | *Boswellia carterii* | M002181 | YISSUM, RESEARCH DEVELOPMENT COMPANY OF THE HEBREW UNIVERSITY OF JERUSALEM; ARIEL-UNIVERSITY RESEARCH AND DEVELOPMENT COMPANY LTD. 41; WO2008/65666; A2; (2008). |
| F2 | *Boswellia carterii* | M002280 | Yoshikawa, Masayuki; Morikawa, Toshio; Oominami, Hideo; Matsuda, Hisashi; Chemical and Pharmaceutical Bulletin; vol. 57; 9; (2009); p. 957 - 964. |
| F2 | *Boswellia carterii* | M002702 | Morikawa, Toshio; Oominami, Hideo; Matsuda, Hisashi; Yoshikawa, Masayuki; Chemical and Pharmaceutical Bulletin; vol. 58; 11; (2010); p. 1541 - 1544. |
| F2 | *Boswellia carterii* | M002939 | 周金云等. 药学学报, 2002, 37 (8): 633 |
| F2 | *Boswellia carterii* | M006317 | Yoshikawa, Masayuki; Morikawa, Toshio; Oominami, Hideo; Matsuda, Hisashi; Chemical and Pharmaceutical Bulletin; vol. 57; 9; (2009); p. 957 - 964. |
| F2 | *Boswellia carterii* | M007187 | Wang, Feng; Li, Zhan-Lin; Cui, Hong-Hua; Hua, Hui-Ming; Jing, Yong-Kui; Liang, Sheng-Wang; Journal of Asian Natural Products Research; vol. 13; 3; (2011); p. 193 - 197. |
| F2 | *Boswellia carterii* | M007557 | Yoshikawa, Masayuki; Morikawa, Toshio; Oominami, Hideo; Matsuda, Hisashi; Chemical and Pharmaceutical Bulletin; vol. 57; 9; (2009); p. 957 - 964. |
| F2 | *Boswellia carterii* | M007599 | Morikawa, Toshio; Oominami, Hideo; Matsuda, Hisashi; Yoshikawa, Masayuki; Chemical and Pharmaceutical Bulletin; vol. 58; 11; (2010); p. 1541 - 1544. |
| F2 | *Boswellia carterii* | M007609 | (1) 国家中医药管理局《中华本草》编委会. 中华本草. Vol. 1-30 上海:上海科学技术出版社, 1999. (2) Buckingham J(Executive Editor): et al. Dictionary of Natural Products, Vol 1-7, Chapman & Hall, London, 1994; 1995, Vol 8; 1996, Vol 9; 1997, Vol 10; 1998, Vol 11.. |
| F2 | *Boswellia carterii* | M007619 | Morikawa, Toshio; Oominami, Hideo; Matsuda, Hisashi; Yoshikawa, Masayuki; Chemical and Pharmaceutical Bulletin; vol. 58; 11; (2010); p. 1541 - 1544. |
| F2 | *Boswellia carterii* | M008598 | Beaucourt; Monatshefte fuer Chemie; vol. 53/54; (1929); p. 900.; Tschirch; Halbey; Archiv der Pharmazie (Weinheim, Germany); vol. 236; (1898); p. 1898,489. |
| F2 | *Boswellia carterii* | M008658 | Yoshikawa, Masayuki; Morikawa, Toshio; Oominami, Hideo; Matsuda, Hisashi; Chemical and Pharmaceutical Bulletin; vol. 57; 9; (2009); p. 957 - 964. |
| F2 | *Boswellia carterii* | M008764 | Culioli G, et al. Phytochemistry, 2003, 62, 537 |
| F2 | *Boswellia carterii* | M008874 | Yoshikawa, Masayuki; Morikawa, Toshio; Oominami, Hideo; Matsuda, Hisashi; Chemical and Pharmaceutical Bulletin; vol. 57; 9; (2009); p. 957 - 964. |
| F2 | *Boswellia carterii* | M009135 | 国家中医药管理局《中华本草》编委会. 中华本草. Vol. 1-30 上海:上海科学技术出版社, 1999 |
| F2 | *Boswellia carterii* | M009447 | Wang, Feng; Li, Zhan-Lin; Cui, Hong-Hua; Hua, Hui-Ming; Jing, Yong-Kui; Liang, Sheng-Wang; Journal of Asian Natural Products Research; vol. 13; 3; (2011); p. 193 - 197. |
| F2 | *Boswellia carterii* | M010214 | Fattorusso, Ernesto; Santacroce, Ciro; Xaasan, Cabdi F.; Phytochemistry (Elsevier); vol. 22; 12; (1983); p. 2868 - 2869. |
| F2 | *Boswellia carterii* | M010872 | Wang, Feng; Li, Zhan-Lin; Cui, Hong-Hua; Hua, Hui-Ming; Jing, Yong-Kui; Liang, Sheng-Wang; Journal of Asian Natural Products Research; vol. 13; 3; (2011); p. 193 - 197. |
| F2 | *Boswellia carterii* | M010937 | Morikawa, Toshio; Oominami, Hideo; Matsuda, Hisashi; Yoshikawa, Masayuki; Chemical and Pharmaceutical Bulletin; vol. 58; 11; (2010); p. 1541 - 1544. |
| F2 | *Boswellia carterii* | M011032 | Fattorusso, Ernesto; Santacroce, Ciro; Xaasan, Cabdi F.; Phytochemistry (Elsevier); vol. 22; 12; (1983); p. 2868 - 2869. |
| F2 | *Boswellia carterii* | M011674 | Calixto JB, et al. Planta Med, 2003, 69 (11): 973 |
| F2 | *Boswellia carterii* | M011995 | Morikawa, Toshio; Oominami, Hideo; Matsuda, Hisashi; Yoshikawa, Masayuki; Chemical and Pharmaceutical Bulletin; vol. 58; 11; (2010); p. 1541 - 1544. |
| F2 | *Boswellia carterii* | M012065 | Yoshikawa, Masayuki; Morikawa, Toshio; Oominami, Hideo; Matsuda, Hisashi; Chemical and Pharmaceutical Bulletin; vol. 57; 9; (2009); p. 957 - 964. |
| F2 | *Boswellia carterii* | M012230 | Morikawa, Toshio; Oominami, Hideo; Matsuda, Hisashi; Yoshikawa, Masayuki; Chemical and Pharmaceutical Bulletin; vol. 58; 11; (2010); p. 1541 - 1544. |
| F2 | *Boswellia carterii* | M012455 | Yoshikawa, Masayuki; Morikawa, Toshio; Oominami, Hideo; Matsuda, Hisashi; Chemical and Pharmaceutical Bulletin; vol. 57; 9; (2009); p. 957 - 964. |
| F2 | *Boswellia carterii* | M012982 | Morikawa, Toshio; Oominami, Hideo; Matsuda, Hisashi; Yoshikawa, Masayuki; Chemical and Pharmaceutical Bulletin; vol. 58; 11; (2010); p. 1541 - 1544. |
| F2 | *Boswellia carterii* | M013299 | Beaucourt; Monatshefte fuer Chemie; vol. 53/54; (1929); p. 900.; Tschirch; Halbey; Archiv der Pharmazie (Weinheim, Germany); vol. 236; (1898); p. 1898,489. |
| F2 | *Boswellia carterii* | M013469 | Yoshikawa, Masayuki; Morikawa, Toshio; Oominami, Hideo; Matsuda, Hisashi; Chemical and Pharmaceutical Bulletin; vol. 57; 9; (2009); p. 957 - 964. |
| F2 | *Boswellia carterii* | M013715 | Morikawa, Toshio; Oominami, Hideo; Matsuda, Hisashi; Yoshikawa, Masayuki; Chemical and Pharmaceutical Bulletin; vol. 58; 11; (2010); p. 1541 - 1544. |
| F2 | *Boswellia carterii* | M014275 | Yoshikawa, Masayuki; Morikawa, Toshio; Oominami, Hideo; Matsuda, Hisashi; Chemical and Pharmaceutical Bulletin; vol. 57; 9; (2009); p. 957 - 964. |
| F2 | *Boswellia carterii* | M014455 | Sailer, Eckart-Roderich; Hoernlein, Rainer F.; Subramanian, Lakshminarayanapuram R.; Ammon, Hermann P.T.; Safayhi, Hasan; Archiv der Pharmazie; vol. 329; 1; (1996); p. 54 - 56. |
| F2 | *Boswellia carterii* | M014517 | Fattorusso, Ernesto; Santacroce, Ciro; Xaasan, Cabdi F.; Phytochemistry (Elsevier); vol. 22; 12; (1983); p. 2868 - 2869. |
| F2 | *Boswellia carterii* | M014582 | Yoshikawa, Masayuki; Morikawa, Toshio; Oominami, Hideo; Matsuda, Hisashi; Chemical and Pharmaceutical Bulletin; vol. 57; 9; (2009); p. 957 - 964. |
| F2 | *Boswellia carterii* | M015244 | Yoshikawa, Masayuki; Morikawa, Toshio; Oominami, Hideo; Matsuda, Hisashi; Chemical and Pharmaceutical Bulletin; vol. 57; 9; (2009); p. 957 - 964. |
| F2 | *Boswellia carterii* | M015986 | Wang, Feng; Li, Zhan-Lin; Cui, Hong-Hua; Hua, Hui-Ming; Jing, Yong-Kui; Liang, Sheng-Wang; Journal of Asian Natural Products Research; vol. 13; 3; (2011); p. 193 - 197. |
| F2 | *Boswellia carterii* | M016360 | Wang, Feng; Li, Zhan-Lin; Cui, Hong-Hua; Hua, Hui-Ming; Jing, Yong-Kui; Liang, Sheng-Wang; Journal of Asian Natural Products Research; vol. 13; 3; (2011); p. 193 - 197. |
| F2 | *Boswellia carterii* | M016361 | Wang, Feng; Li, Zhan-Lin; Cui, Hong-Hua; Hua, Hui-Ming; Jing, Yong-Kui; Liang, Sheng-Wang; Journal of Asian Natural Products Research; vol. 13; 3; (2011); p. 193 - 197. |
| F2 | *Boswellia carterii* | M016362 | Wang, Feng; Li, Zhan-Lin; Cui, Hong-Hua; Hua, Hui-Ming; Jing, Yong-Kui; Liang, Sheng-Wang; Journal of Asian Natural Products Research; vol. 13; 3; (2011); p. 193 - 197. |
| F2 | *Boswellia carterii* | M017584 | Wang, Feng; Li, Zhan-Lin; Cui, Hong-Hua; Hua, Hui-Ming; Jing, Yong-Kui; Liang, Sheng-Wang; Journal of Asian Natural Products Research; vol. 13; 3; (2011); p. 193 - 197. |
| F2 | *Boswellia carterii* | M018019 | Wang, Yan-Gai; Ren, Jin; Wang, Ai-Guo; Yang, Jian-Bo; Ji, Teng-Fei; Ma, Qin-Ge; Tian, Jin; Su, Ya-Lun; Journal of Natural Products; vol. 76; 11; (2013); p. 2074 - 2079. |
| F2 | *Boswellia carterii* | M018020 | Wang, Yan-Gai; Ren, Jin; Wang, Ai-Guo; Yang, Jian-Bo; Ji, Teng-Fei; Ma, Qin-Ge; Tian, Jin; Su, Ya-Lun; Journal of Natural Products; vol. 76; 11; (2013); p. 2074 - 2079. |
| F2 | *Boswellia carterii* | M018464 | Wang, Yan-Gai; Ren, Jin; Wang, Ai-Guo; Yang, Jian-Bo; Ji, Teng-Fei; Ma, Qin-Ge; Tian, Jin; Su, Ya-Lun; Journal of Natural Products; vol. 76; 11; (2013); p. 2074 - 2079. |
| F2 | *Boswellia carterii* | M018902 | Wang, Yan-Gai; Ren, Jin; Wang, Ai-Guo; Yang, Jian-Bo; Ji, Teng-Fei; Ma, Qin-Ge; Tian, Jin; Su, Ya-Lun; Journal of Natural Products; vol. 76; 11; (2013); p. 2074 - 2079. |
| F2 | *Boswellia carterii* | M019315 | Wang, Yan-Gai; Ren, Jin; Wang, Ai-Guo; Yang, Jian-Bo; Ji, Teng-Fei; Ma, Qin-Ge; Tian, Jin; Su, Ya-Lun; Journal of Natural Products; vol. 76; 11; (2013); p. 2074 - 2079. |
| F2 | *Boswellia carterii* | M020142 | Wang, Yan-Gai; Ren, Jin; Wang, Ai-Guo; Yang, Jian-Bo; Ji, Teng-Fei; Ma, Qin-Ge; Tian, Jin; Su, Ya-Lun; Journal of Natural Products; vol. 76; 11; (2013); p. 2074 - 2079. |
| F2 | *Boswellia carterii* | M020970 | Wang, Yan-Gai; Ren, Jin; Wang, Ai-Guo; Yang, Jian-Bo; Ji, Teng-Fei; Ma, Qin-Ge; Tian, Jin; Su, Ya-Lun; Journal of Natural Products; vol. 76; 11; (2013); p. 2074 - 2079. |
| F2 | *Boswellia carterii* | M021424 | Wang, Yan-Gai; Ren, Jin; Wang, Ai-Guo; Yang, Jian-Bo; Ji, Teng-Fei; Ma, Qin-Ge; Tian, Jin; Su, Ya-Lun; Journal of Natural Products; vol. 76; 11; (2013); p. 2074 - 2079. |
| F2 | *Boswellia carterii* | M021859 | Wang, Yan-Gai; Ren, Jin; Wang, Ai-Guo; Yang, Jian-Bo; Ji, Teng-Fei; Ma, Qin-Ge; Tian, Jin; Su, Ya-Lun; Journal of Natural Products; vol. 76; 11; (2013); p. 2074 - 2079. |
| F2 | *Boswellia carterii* | M022840 | Morikawa, Toshio; Oominami, Hideo; Matsuda, Hisashi; Yoshikawa, Masayuki; Chemical and Pharmaceutical Bulletin; vol. 58; 11; (2010); p. 1541 - 1544. |
| F2 | *Boswellia carterii* | M023784 | 国家中医药管理局《中华本草》编委会. 中华本草. Vol. 1-30 上海:上海科学技术出版社, 1999 |
| F2 | *Boswellia carterii* | M024164 | Morikawa, Toshio; Oominami, Hideo; Matsuda, Hisashi; Yoshikawa, Masayuki; Chemical and Pharmaceutical Bulletin; vol. 58; 11; (2010); p. 1541 - 1544. |
| F2 | *Boswellia carterii* | M024607 | (1) 国家中医药管理局《中华本草》编委会. 中华本草. Vol. 1-30 上海:上海科学技术出版社, 1999. (2) Buckingham J(Executive Editor): et al. Dictionary of Natural Products, Vol 1-7, Chapman & Hall, London, 1994; 1995, Vol 8; 1996, Vol 9; 1997, Vol 10; 1998, Vol 11.. |
| F2 | *Boswellia carterii* | M024666 | Morikawa, Toshio; Oominami, Hideo; Matsuda, Hisashi; Yoshikawa, Masayuki; Chemical and Pharmaceutical Bulletin; vol. 58; 11; (2010); p. 1541 - 1544. |
| F2 | *Boswellia carterii* | M025306 | Fattorusso, Ernesto; Santacroce, Ciro; Xaasan, Cabdi F.; Phytochemistry (Elsevier); vol. 22; 12; (1983); p. 2868 - 2869. |
| F2 | *Boswellia carterii* | M025381 | Morikawa, Toshio; Oominami, Hideo; Matsuda, Hisashi; Yoshikawa, Masayuki; Chemical and Pharmaceutical Bulletin; vol. 58; 11; (2010); p. 1541 - 1544. |
| F2 | *Boswellia carterii* | M025414 | Sailer, Eckart-Roderich; Hoernlein, Rainer F.; Subramanian, Lakshminarayanapuram R.; Ammon, Hermann P.T.; Safayhi, Hasan; Archiv der Pharmazie; vol. 329; 1; (1996); p. 54 - 56. |
| F2 | *Boswellia carterii* | M025864 | Morikawa, Toshio; Oominami, Hideo; Matsuda, Hisashi; Yoshikawa, Masayuki; Chemical and Pharmaceutical Bulletin; vol. 58; 11; (2010); p. 1541 - 1544. |
| F2 | *Boswellia carterii* | M027176 | Pailer M, et al. Monatsh Chem, 1981, 112 (3): 341 |
| F2 | *Boswellia carterii* | M027875 | 国家中医药管理局《中华本草》编委会. 中华本草. Vol. 1-30 上海:上海科学技术出版社, 1999 |
| F2 | *Boswellia carterii* | M028517 | Sailer, Eckart-Roderich; Hoernlein, Rainer F.; Subramanian, Lakshminarayanapuram R.; Ammon, Hermann P.T.; Safayhi, Hasan; Archiv der Pharmazie; vol. 329; 1; (1996); p. 54 - 56. |
| F2 | *Boswellia carterii* | M028538 | (1) Pailer M, et al. Monatsh Chem, 1981, 112 (3): 341. (2) 刘锁兰等. 中国中药杂志, 1991, 16 (6): 359. |
| F2 | *Boswellia carterii* | M029693 | Morikawa, Toshio; Oominami, Hideo; Matsuda, Hisashi; Yoshikawa, Masayuki; Chemical and Pharmaceutical Bulletin; vol. 58; 11; (2010); p. 1541 - 1544. |
| F2 | *Boswellia carterii* | M030379 | (1) Pailer M, et al. Monatsh Chem, 1981, 112 (3): 341. (2) Kameoka H, et al.Chem. Abstr., 1979, 90, 69070y. |
| F2 | *Boswellia carterii* | M030729 | (1) 江苏新医学院. 中药大辞典. 上海: 上海科学技术出版社, 1977. (2) 国家中医药管理局《中华本草》编委会. 中华本草. Vol. 1-30 上海:上海科学技术出版社, 1999. |
| F2 | *Boswellia carterii* | M030914 | Yoshikawa, Masayuki; Morikawa, Toshio; Oominami, Hideo; Matsuda, Hisashi; Chemical and Pharmaceutical Bulletin; vol. 57; 9; (2009); p. 957 - 964. |
| F2 | *Boswellia carterii* | M031021 | Morikawa, Toshio; Oominami, Hideo; Matsuda, Hisashi; Yoshikawa, Masayuki; Chemical and Pharmaceutical Bulletin; vol. 58; 11; (2010); p. 1541 - 1544. |
| F2 | *Boswellia carterii* | M031263 | 国家中医药管理局《中华本草》编委会. 中华本草. Vol. 1-30 上海:上海科学技术出版社, 1999 |
| F2 | *Boswellia carterii* | M031808 | Fattorusso, Ernesto; Santacroce, Ciro; Xaasan, Cabdi F.; Phytochemistry (Elsevier); vol. 22; 12; (1983); p. 2868 - 2869. |
| F2 | *Boswellia carterii* | M032507 | Morikawa, Toshio; Oominami, Hideo; Matsuda, Hisashi; Yoshikawa, Masayuki; Chemical and Pharmaceutical Bulletin; vol. 58; 11; (2010); p. 1541 - 1544. |
| F2 | *Boswellia carterii* | M032856 | Sailer, Eckart-Roderich; Hoernlein, Rainer F.; Subramanian, Lakshminarayanapuram R.; Ammon, Hermann P.T.; Safayhi, Hasan; Archiv der Pharmazie; vol. 329; 1; (1996); p. 54 - 56. |
| F2 | *Boswellia carterii* | M032923 | Morikawa, Toshio; Oominami, Hideo; Matsuda, Hisashi; Yoshikawa, Masayuki; Chemical and Pharmaceutical Bulletin; vol. 58; 11; (2010); p. 1541 - 1544. |
| F2 | *Boswellia carterii* | M033497 | Wang, Feng; Li, Zhan-Lin; Cui, Hong-Hua; Hua, Hui-Ming; Jing, Yong-Kui; Liang, Sheng-Wang; Journal of Asian Natural Products Research; vol. 13; 3; (2011); p. 193 - 197. |
| F2 | *Boswellia carterii* | M033673 | Yoshikawa, Masayuki; Morikawa, Toshio; Oominami, Hideo; Matsuda, Hisashi; Chemical and Pharmaceutical Bulletin; vol. 57; 9; (2009); p. 957 - 964. |
| F2 | *Boswellia carterii* | M034198 | Morikawa, Toshio; Oominami, Hideo; Matsuda, Hisashi; Yoshikawa, Masayuki; Chemical and Pharmaceutical Bulletin; vol. 58; 11; (2010); p. 1541 - 1544. |
| F2 | *Carthamus tinctorius* | M000015 | Allen; Thomas; Phytochemistry (Elsevier); vol. 10; (1971); p. 1579,1580. |
| F2 | *Carthamus tinctorius* | M000067 | Akihisa, Toshihiro; Oinuma, Hirotoshi; Tamura, Toshitake; Kasahara, Yoshimasa; Kumaki, Kunio; et al.; Phytochemistry (Elsevier); vol. 36; 1; (1994); p. 105 - 108. |
| F2 | *Carthamus tinctorius* | M000543 | Retrieved from CNPD |
| F2 | *Carthamus tinctorius* | M000569 | (1) 阴健等. 中药现代研究与临床应用(1). 北京: 学苑出版社, 1993. (2) 欧明等. 简明中药成分手册. 北京: 中国医药科技出版社, 2003. |
| F2 | *Carthamus tinctorius* | M000697 | 国家中医药管理局《中华本草》编委会. 中华本草. Vol. 1-30 上海:上海科学技术出版社, 1999 |
| F2 | *Carthamus tinctorius* | M000780 | Yin, Hong-Bin; He, Zhi-Sheng; Tetrahedron Letters; vol. 41; 12; (2000); p. 1955 - 1958. |
| F2 | *Carthamus tinctorius* | M001457 | 李锋等. 中草药, 2004, 35 (3): 247 |
| F2 | *Carthamus tinctorius* | M002205 | (1) 江苏新医学院. 中药大辞典. 上海: 上海科学技术出版社, 1977. (2) 国家中医药管理局《中华本草》编委会. 中华本草. Vol. 1-30 上海:上海科学技术出版社, 1999. (3) 陈蕙芳等. 植物活性成分辞典. 第1册. 北京: 中国医药科技出版社, 2001. |
| F2 | *Carthamus tinctorius* | M002557 | Hilditch,T.P.; The Chemical Constitution of Natural Fats, 3.Aufl. <London 1956> S.172,173. |
| F2 | *Carthamus tinctorius* | M002823 | Kazuma, Kohei; Takahashi, Takashi; Sato, Katsura; Takeuchi, Hisatomo; Matsumoto, Takeshi; Okuno, Toshikatsu; Bioscience, Biotechnology and Biochemistry; vol. 64; 8; (2000); p. 1588 - 1599. |
| F2 | *Carthamus tinctorius* | M002862 | Bohlmann,F. et al.; Chemische Berichte; vol. 99; (1966); p. 3433 - 3436. |
| F2 | *Carthamus tinctorius* | M002920 | (1) 阴健等. 中药现代研究与临床应用(1). 北京: 学苑出版社, 1993. (2) 欧明等. 简明中药成分手册. 北京: 中国医药科技出版社, 2003. |
| F2 | *Carthamus tinctorius* | M003009 | Akihisa, Toshihiro; Yasukawa, Ken; Oinuma, Hirotoshi; Kasahara, Yoshimasa; Yamanouchi, Sakae; Takido, Michio; Kumaki, Kunio; Tamura, Toshitake; Phytochemistry; vol. 43; 6; (1996); p. 1255 - 1260. |
| F2 | *Carthamus tinctorius* | M003214 | Akihisa, Toshihiro; Yasukawa, Ken; Oinuma, Hirotoshi; Kasahara, Yoshimasa; Yamanouchi, Sakae; Takido, Michio; Kumaki, Kunio; Tamura, Toshitake; Phytochemistry; vol. 43; 6; (1996); p. 1255 - 1260. |
| F2 | *Carthamus tinctorius* | M003402 | Zhang, Ge; Guo, Mei-Li; Li, Run-Ping; Li, Ying; Zhang, Han-Ming; Su, Zhong-Wu; Chemistry of Natural Compounds; vol. 45; 3; (2009); p. 398 - 401. |
| F2 | *Carthamus tinctorius* | M003449 | Hattori, Masao; Huang, Xin-li; Che, Qing-Ming; Kawata, Yukio; Tezuka, Yasuhiro; et al.; Phytochemistry (Elsevier); vol. 31; 11; (1992); p. 4001 - 4004. |
| F2 | *Carthamus tinctorius* | M003534 | (1) 江苏新医学院. 中药大辞典. 上海: 上海科学技术出版社, 1977. (2) 国家中医药管理局《中华本草》编委会. 中华本草. Vol. 1-30 上海:上海科学技术出版社, 1999. |
| F2 | *Carthamus tinctorius* | M003569 | Han, Shu-Yan; Li, Hai-Xia; Bai, Chang-Cai; Wang, Li; Tu, Peng-Fei; Chemistry and Biodiversity; vol. 7; 2; (2010); p. 383 - 391. |
| F2 | *Carthamus tinctorius* | M003772 | Bohlmann,F. et al.; Chemische Berichte; vol. 99; (1966); p. 3433 - 3436. |
| F2 | *Carthamus tinctorius* | M003791 | Kazuma, Kohei; Shirai, Eriko; Wada, Mizu; Umeo, Kazuhiro; Sato, Atsushi; et al.; Bioscience, Biotechnology, and Biochemistry; vol. 59; 8; (1995); p. 1588 - 1590. |
| F2 | *Carthamus tinctorius* | M003829 | Binder et al.; Phytochemistry (Elsevier); vol. 17; (1978); p. 315,317. |
| F2 | *Carthamus tinctorius* | M003874 | (1) Buttery RG, et al.Chem. Abstr., 1990, 113, 210415s. (2) Ronald GB, et al. J Agric Food Chem, 1990, 36, 1245. |
| F2 | *Carthamus tinctorius* | M003891 | (1) 国家中医药管理局《中华本草》编委会. 中华本草. Vol. 1-30 上海:上海科学技术出版社, 1999. (2) Buckingham J(Executive Editor): et al. Dictionary of Natural Products, Vol 1-7, Chapman & Hall, London, 1994; 1995, Vol 8; 1996, Vol 9; 1997, Vol 10; 1998, Vol 11.. |
| F2 | *Carthamus tinctorius* | M003938 | Sato, Hiroji; Kawagishi, Hirokazu; Nishimura, Tsutomu; Yoneyama, Syozou; Yoshimoto, Yuko; et al.; Agricultural and Biological Chemistry; vol. 49; 10; (1985); p. 2969 - 2974. |
| F2 | *Carthamus tinctorius* | M004231 | Yang, Wen-Zhi; Qiao, Xue; Bo, Tao; Wang, Qing; Guo, De-An; Ye, Min; Rapid Communications in Mass Spectrometry; vol. 28; 4; (2014); p. 385 - 395. |
| F2 | *Carthamus tinctorius* | M004357 | (1) Buckingham J(Executive Editor): et al. Dictionary of Natural Products, Vol 1-7, Chapman & Hall, London, 1994; 1995, Vol 8; 1996, Vol 9; 1997, Vol 10; 1998, Vol 11.. (2) JIANG Y, et al. Chem Pharm Bull, 2005, 53 (9): 1164. |
| F2 | *Carthamus tinctorius* | M004395 | Hattori, Masao; Huang, Xin-li; Che, Qing-Ming; Kawata, Yukio; Tezuka, Yasuhiro; et al.; Phytochemistry (Elsevier); vol. 31; 11; (1992); p. 4001 - 4004. |
| F2 | *Carthamus tinctorius* | M004590 | Retrieved from CNPD |
| F2 | *Carthamus tinctorius* | M004632 | Nishibe et al.; Phytochemistry (Elsevier); vol. 11; (1972); p. 2623. |
| F2 | *Carthamus tinctorius* | M005228 | 阴健等. 中药现代研究与临床应用(1). 北京: 学苑出版社, 1993 |
| F2 | *Carthamus tinctorius* | M005302 | Furuya, Tsutomu; Yoshikawa, Takafumi; Kimura, Takako; Kaneko, Hiroko; Phytochemistry (Elsevier); vol. 26; 10; (1987); p. 2741 - 2748. |
| F2 | *Carthamus tinctorius* | M005342 | Ahmed; Marzouk; El-Khrisy; Abdel Wahab; El-Din; Pharmazie; vol. 55; 8; (2000); p. 621 - 622. |
| F2 | *Carthamus tinctorius* | M005470 | Akihisa, Toshihiro; Oinuma, Hirotoshi; Tamura, Toshitake; Kasahara, Yoshimasa; Kumaki, Kunio; et al.; Phytochemistry (Elsevier); vol. 36; 1; (1994); p. 105 - 108. |
| F2 | *Carthamus tinctorius* | M005515 | Kumazawa, Toshihiro; Sato, Shingo; Kanenari, Daisuke; Kunimatsu, Akira; Hirose, Ryoji; et al.; Chemistry Letters; 12; (1994); p. 2343 - 2344. |
| F2 | *Carthamus tinctorius* | M005572 | Binder et al.; Phytochemistry (Elsevier); vol. 17; (1978); p. 315,317. |
| F2 | *Carthamus tinctorius* | M005960 | Kogiso et al.; Tetrahedron Letters; (1976); p. 109. |
| F2 | *Carthamus tinctorius* | M006069 | (1) 国家中医药管理局《中华本草》编委会. 中华本草. Vol. 1-30 上海:上海科学技术出版社, 1999. (2) Junichi O, et al.Chem. Abstr., 1981, 95, 43572s. |
| F2 | *Carthamus tinctorius* | M006110 | Yamato M, et al. Chem. Abstr., 1990, 112, 204663j |
| F2 | *Carthamus tinctorius* | M006185 | Retrieved from CNPD |
| F2 | *Carthamus tinctorius* | M006230 | Sakamura, Sadao; Terayama, Yoshihiko; Kawakatsu, Satomi; Ichihara, Akitami; Saito, Hideya; Agricultural and Biological Chemistry; vol. 44; 12; (1980); p. 2951 - 2954. |
| F2 | *Carthamus tinctorius* | M006408 | Zhang, Ge; Guo, Mei-Li; Li, Run-Ping; Li, Ying; Zhang, Han-Ming; Su, Zhong-Wu; Chemistry of Natural Compounds; vol. 45; 3; (2009); p. 398 - 401. |
| F2 | *Carthamus tinctorius* | M006618 | Bohlmann,F. et al.; Chemische Berichte; vol. 99; (1966); p. 3433 - 3436. |
| F2 | *Carthamus tinctorius* | M006806 | (1) 阴健等. 中药现代研究与临床应用(1). 北京: 学苑出版社, 1993. (2) 孙文基等. 天然活性成分简明手册. 北京: 中国医药科技出版社, 1998. (3) 国家中医药管理局《中华本草》编委会. 中华本草. Vol. 1-30 上海:上海科学技术出版社, 1999. |
| F2 | *Carthamus tinctorius* | M006909 | Hattori, Masao; Huang, Xin-li; Che, Qing-Ming; Kawata, Yukio; Tezuka, Yasuhiro; et al.; Phytochemistry (Elsevier); vol. 31; 11; (1992); p. 4001 - 4004. |
| F2 | *Carthamus tinctorius* | M007021 | Akihisa, Toshihiro; Oinuma, Hirotoshi; Tamura, Toshitake; Kasahara, Yoshimasa; Kumaki, Kunio; et al.; Phytochemistry (Elsevier); vol. 36; 1; (1994); p. 105 - 108. |
| F2 | *Carthamus tinctorius* | M007166 | Yoo, Hye Hyun; Park, Jeong Hill; Kwon, Sung Won; Bioscience, Biotechnology and Biochemistry; vol. 70; 11; (2006); p. 2783 - 2785. |
| F2 | *Carthamus tinctorius* | M007271 | 国家中医药管理局《中华本草》编委会. 中华本草. Vol. 1-30 上海:上海科学技术出版社, 1999 |
| F2 | *Carthamus tinctorius* | M007369 | Advanced Gene Technology, Corp.; EP1205182; B1; (2005). |
| F2 | *Carthamus tinctorius* | M007397 | Han, Shu-Yan; Li, Hai-Xia; Bai, Chang-Cai; Wang, Li; Tu, Peng-Fei; Chemistry and Biodiversity; vol. 7; 2; (2010); p. 383 - 391. |
| F2 | *Carthamus tinctorius* | M007429 | Retrieved from CNPD |
| F2 | *Carthamus tinctorius* | M007435 | Retrieved from CNPD |
| F2 | *Carthamus tinctorius* | M007769 | 国家中医药管理局《中华本草》编委会. 中华本草. Vol. 1-30 上海:上海科学技术出版社, 1999 |
| F2 | *Carthamus tinctorius* | M007827 | Retrieved from CNPD |
| F2 | *Carthamus tinctorius* | M007833 | Suleimanov; Chemistry of Natural Compounds; vol. 40; 1; (2004); p. 13 - 15. |
| F2 | *Carthamus tinctorius* | M007844 | 国家中医药管理局《中华本草》编委会. 中华本草. Vol. 1-30 上海:上海科学技术出版社, 1999 |
| F2 | *Carthamus tinctorius* | M008015 | He, Jun; Shen, Yi; Jiang, Jian-Shuang; Yang, Ya-Nan; Feng, Zi-Ming; Zhang, Pei-Cheng; Yuan, Shao-Peng; Hou, Qi; Carbohydrate Research; vol. 346; 13; (2011); p. 1903 - 1908. |
| F2 | *Carthamus tinctorius* | M008130 | Suleimanov; Chemistry of Natural Compounds; vol. 40; 1; (2004); p. 13 - 15. |
| F2 | *Carthamus tinctorius* | M008269 | Zhang, Ge; Guo, Mei-Li; Li, Run-Ping; Li, Ying; Zhang, Han-Ming; Su, Zhong-Wu; Chemistry of Natural Compounds; vol. 45; 3; (2009); p. 398 - 401. |
| F2 | *Carthamus tinctorius* | M008334 | Binder et al.; Phytochemistry (Elsevier); vol. 17; (1978); p. 315,317. |
| F2 | *Carthamus tinctorius* | M008428 | Yang, Wen-Zhi; Qiao, Xue; Bo, Tao; Wang, Qing; Guo, De-An; Ye, Min; Rapid Communications in Mass Spectrometry; vol. 28; 4; (2014); p. 385 - 395. |
| F2 | *Carthamus tinctorius* | M008471 | Hattori, Masao; Huang, Xin-li; Che, Qing-Ming; Kawata, Yukio; Tezuka, Yasuhiro; et al.; Phytochemistry (Elsevier); vol. 31; 11; (1992); p. 4001 - 4004. |
| F2 | *Carthamus tinctorius* | M008706 | Zhou, Yu-Zhi; Ma, Hong-Yu; Chen, Huan; Qiao, Li; Yao, Yao; Cao, Jia-Qing; Pei, Yue-Hu; Chemical and Pharmaceutical Bulletin; vol. 54; 10; (2006); p. 1455 - 1456. |
| F2 | *Carthamus tinctorius* | M008762 | Sakamura, Sadao; Terayama, Yoshihiko; Kawakatsu, Satomi; Ichihara, Akitami; Saito, Hideya; Agricultural and Biological Chemistry; vol. 44; 12; (1980); p. 2951 - 2954. |
| F2 | *Carthamus tinctorius* | M008808 | Sakamura, Sadao; Terayama, Yoshihiko; Kawakatsu, Satomi; Ichihara, Akitami; Saito, Hideya; Agricultural and Biological Chemistry; vol. 44; 12; (1980); p. 2951 - 2954. |
| F2 | *Carthamus tinctorius* | M008986 | (1) 阴健等. 中药现代研究与临床应用(1). 北京: 学苑出版社, 1993. (2) 饶高雄等. 药学学报, 1991, 26 (1): 30. (3) 杨峻山等. 药学学报, 1993, 28 (3): 197. (4) 赵余庆等. 中国中药杂志, 1993, 18 (7): 428. (5) �母咝鄣� 中国中药杂志, 1993, 18 (12): 736. (6) 饶高雄等. 中国中药杂志, 1995, 20 (12): 740. (7) 国家中医药管理局《中华本草》编委会. 中华本草. Vol. 1-30 上海:上海科学技术出版社, 1999. (8) TORIUMI Y, et al. Chem Pharm Bull, 2003, 51 (1): 89. (9) Carcache-Blanco EJ, et al. JNP, 2003, 67 (1): 126. |
| F2 | *Carthamus tinctorius* | M009036 | Zhu H, et al. Planta Med, 2003, 69, 429 |
| F2 | *Carthamus tinctorius* | M009111 | Meselhy; Kadota; Momose; Hatakeyama; Kusai; Hattori; Namba; Chemical and Pharmaceutical Bulletin; vol. 41; 10; (1993); p. 1796 - 1802. |
| F2 | *Carthamus tinctorius* | M009146 | 国家中医药管理局《中华本草》编委会. 中华本草. Vol. 1-30 上海:上海科学技术出版社, 1999 |
| F2 | *Carthamus tinctorius* | M009470 | Binder et al.; Phytochemistry (Elsevier); vol. 14; (1975); p. 2085,2086. |
| F2 | *Carthamus tinctorius* | M009514 | Kazuma, Kohei; Takahashi, Takashi; Sato, Katsura; Takeuchi, Hisatomo; Matsumoto, Takeshi; Okuno, Toshikatsu; Bioscience, Biotechnology and Biochemistry; vol. 64; 8; (2000); p. 1588 - 1599. |
| F2 | *Carthamus tinctorius* | M009594 | Kazuma, Kohei; Takahashi, Takashi; Sato, Katsura; Takeuchi, Hisatomo; Matsumoto, Takeshi; Okuno, Toshikatsu; Bioscience, Biotechnology and Biochemistry; vol. 64; 8; (2000); p. 1588 - 1599. |
| F2 | *Carthamus tinctorius* | M009773 | (1) 阴健等. 中药现代研究与临床应用(1). 北京: 学苑出版社, 1993. (2) Ma WZ, et al. JNP, 2003, 66 (3): 441. (3) Yang XD, et al. Phytochemistry, 2001, 58, 1245. |
| F2 | *Carthamus tinctorius* | M009922 | Suleimanov; Chemistry of Natural Compounds; vol. 40; 1; (2004); p. 13 - 15. |
| F2 | *Carthamus tinctorius* | M010095 | Hattori, Masao; Huang, Xin-li; Che, Qing-Ming; Kawata, Yukio; Tezuka, Yasuhiro; et al.; Phytochemistry (Elsevier); vol. 31; 11; (1992); p. 4001 - 4004. |
| F2 | *Carthamus tinctorius* | M010294 | Akihisa, Toshihiro; Yasukawa, Ken; Oinuma, Hirotoshi; Kasahara, Yoshimasa; Yamanouchi, Sakae; Takido, Michio; Kumaki, Kunio; Tamura, Toshitake; Phytochemistry; vol. 43; 6; (1996); p. 1255 - 1260. |
| F2 | *Carthamus tinctorius* | M010652 | Akihisa, Toshihiro; Oinuma, Hirotoshi; Tamura, Toshitake; Kasahara, Yoshimasa; Kumaki, Kunio; et al.; Phytochemistry (Elsevier); vol. 36; 1; (1994); p. 105 - 108. |
| F2 | *Carthamus tinctorius* | M010818 | Bohlmann,F. et al.; Chemische Berichte; vol. 99; (1966); p. 3433 - 3436. |
| F2 | *Carthamus tinctorius* | M010880 | 国家中医药管理局《中华本草》编委会. 中华本草. Vol. 1-30 上海:上海科学技术出版社, 1999 |
| F2 | *Carthamus tinctorius* | M010882 | 孙文基等. 天然活性成分简明手册. 北京: 中国医药科技出版社, 1998 |
| F2 | *Carthamus tinctorius* | M010883 | Cho, Man-Ho; Paik, Young-Sook; Hahn, Tae-Ryong; Journal of Agricultural and Food Chemistry; vol. 48; 9; (2000); p. 3917 - 3921. |
| F2 | *Carthamus tinctorius* | M010900 | Retrieved from CNPD |
| F2 | *Carthamus tinctorius* | M011067 | 国家中医药管理局《中华本草》编委会. 中华本草. Vol. 1-30 上海:上海科学技术出版社, 1999 |
| F2 | *Carthamus tinctorius* | M011182 | Kazuma, Kohei; Takahashi, Takashi; Sato, Katsura; Takeuchi, Hisatomo; Matsumoto, Takeshi; Okuno, Toshikatsu; Bioscience, Biotechnology and Biochemistry; vol. 64; 8; (2000); p. 1588 - 1599. |
| F2 | *Carthamus tinctorius* | M011700 | Kazuma, Kohei; Takahashi, Takashi; Sato, Katsura; Takeuchi, Hisatomo; Matsumoto, Takeshi; Okuno, Toshikatsu; Bioscience, Biotechnology and Biochemistry; vol. 64; 8; (2000); p. 1588 - 1599. |
| F2 | *Carthamus tinctorius* | M011879 | Ichihara; Noda; Agricultural and Biological Chemistry; vol. 39; (1975); p. 1103,1105,1106. |
| F2 | *Carthamus tinctorius* | M011938 | Akihisa, Toshihiro; Oinuma, Hirotoshi; Tamura, Toshitake; Kasahara, Yoshimasa; Kumaki, Kunio; et al.; Phytochemistry (Elsevier); vol. 36; 1; (1994); p. 105 - 108. |
| F2 | *Carthamus tinctorius* | M012196 | Kazuma, Kohei; Takahashi, Takashi; Sato, Katsura; Takeuchi, Hisatomo; Matsumoto, Takeshi; Okuno, Toshikatsu; Bioscience, Biotechnology and Biochemistry; vol. 64; 8; (2000); p. 1588 - 1599. |
| F2 | *Carthamus tinctorius* | M012459 | Takahashi, Yoshiyuki; Saito, Koshi; Yanagiya, Mitsutoshi; Ikura, Mitsuhiko; Hikichi, Kunio; et al.; Tetrahedron Letters; vol. 25; 23; (1984); p. 2471 - 2474. |
| F2 | *Carthamus tinctorius* | M012509 | Zhao, Gang; Qin, Guo-Wei; Gai, Yue; Guo, Li-He; Chemical and Pharmaceutical Bulletin; vol. 58; 7; (2010); p. 950 - 952. |
| F2 | *Carthamus tinctorius* | M012665 | Akihisa, Toshihiro; Yasukawa, Ken; Oinuma, Hirotoshi; Kasahara, Yoshimasa; Yamanouchi, Sakae; Takido, Michio; Kumaki, Kunio; Tamura, Toshitake; Phytochemistry; vol. 43; 6; (1996); p. 1255 - 1260. |
| F2 | *Carthamus tinctorius* | M013391 | Nagatsu, Akito; Zhang, Hui Li; Watanabe, Toshihiro; Taniguchi, Nari; Hatano, Keiichiro; Mizukami, Hajime; Sakakibara, Jinsaku; Chemical and Pharmaceutical Bulletin; vol. 46; 6; (1998); p. 1044 - 1047. |
| F2 | *Carthamus tinctorius* | M013581 | Akihisa, Toshihiro; Oinuma, Hirotoshi; Tamura, Toshitake; Kasahara, Yoshimasa; Kumaki, Kunio; et al.; Phytochemistry (Elsevier); vol. 36; 1; (1994); p. 105 - 108. |
| F2 | *Carthamus tinctorius* | M013882 | 阴健等. 中药现代研究与临床应用(1). 北京: 学苑出版社, 1993 |
| F2 | *Carthamus tinctorius* | M014052 | Bohlmann,F.; Zdero,C.; Chemische Berichte; vol. 103; (1970); p. 2853 - 2855. |
| F2 | *Carthamus tinctorius* | M014514 | Ahmed; Marzouk; El-Khrisy; Abdel Wahab; El-Din; Pharmazie; vol. 55; 8; (2000); p. 621 - 622. |
| F2 | *Carthamus tinctorius* | M014910 | Review; Hui Li Zhang; Nagatsu; Sakakibara; Chemical and Pharmaceutical Bulletin; vol. 44; 4; (1996); p. 874 - 876. |
| F2 | *Carthamus tinctorius* | M015118 | Akihisa, Toshihiro; Oinuma, Hirotoshi; Tamura, Toshitake; Kasahara, Yoshimasa; Kumaki, Kunio; et al.; Phytochemistry (Elsevier); vol. 36; 1; (1994); p. 105 - 108. |
| F2 | *Carthamus tinctorius* | M015181 | Akihisa, Toshihiro; Oinuma, Hirotoshi; Tamura, Toshitake; Kasahara, Yoshimasa; Kumaki, Kunio; et al.; Phytochemistry (Elsevier); vol. 36; 1; (1994); p. 105 - 108. |
| F2 | *Carthamus tinctorius* | M015409 | (1) 阴健等. 中药现代研究与临床应用(1). 北京: 学苑出版社, 1993. (2) 国家中医药管理局《中华本草》编委会. 中华本草. Vol. 1-30 上海:上海科学技术出版社, 1999. |
| F2 | *Carthamus tinctorius* | M015510 | Zhou, Yu-Zhi; Ma, Hong-Yu; Chen, Huan; Qiao, Li; Yao, Yao; Cao, Jia-Qing; Pei, Yue-Hu; Chemical and Pharmaceutical Bulletin; vol. 54; 10; (2006); p. 1455 - 1456. |
| F2 | *Carthamus tinctorius* | M015819 | Hattori, Masao; Huang, Xin-li; Che, Qing-Ming; Kawata, Yukio; Tezuka, Yasuhiro; et al.; Phytochemistry (Elsevier); vol. 31; 11; (1992); p. 4001 - 4004. |
| F2 | *Carthamus tinctorius* | M015850 | Zhang, Hui Li; Nagatsu, Akito; Watanabe, Toshihiro; Sakakibara, Jinsaku; Okuyama, Harumi; Chemical and Pharmaceutical Bulletin; vol. 45; 12; (1997); p. 1910 - 1914. |
| F2 | *Carthamus tinctorius* | M015966 | (1) 阴健等. 中药现代研究与临床应用(1). 北京: 学苑出版社, 1993. (2) 国家中医药管理局《中华本草》编委会. 中华本草. Vol. 1-30 上海:上海科学技术出版社, 1999. |
| F2 | *Carthamus tinctorius* | M016917 | He, Jun; Shen, Yi; Jiang, Jian-Shuang; Yang, Ya-Nan; Feng, Zi-Ming; Zhang, Pei-Cheng; Yuan, Shao-Peng; Hou, Qi; Carbohydrate Research; vol. 346; 13; (2011); p. 1903 - 1908. |
| F2 | *Carthamus tinctorius* | M016918 | He, Jun; Shen, Yi; Jiang, Jian-Shuang; Yang, Ya-Nan; Feng, Zi-Ming; Zhang, Pei-Cheng; Yuan, Shao-Peng; Hou, Qi; Carbohydrate Research; vol. 346; 13; (2011); p. 1903 - 1908. |
| F2 | *Carthamus tinctorius* | M016919 | He, Jun; Shen, Yi; Jiang, Jian-Shuang; Yang, Ya-Nan; Feng, Zi-Ming; Zhang, Pei-Cheng; Yuan, Shao-Peng; Hou, Qi; Carbohydrate Research; vol. 346; 13; (2011); p. 1903 - 1908. |
| F2 | *Carthamus tinctorius* | M016921 | He, Jun; Shen, Yi; Jiang, Jian-Shuang; Yang, Ya-Nan; Feng, Zi-Ming; Zhang, Pei-Cheng; Yuan, Shao-Peng; Hou, Qi; Carbohydrate Research; vol. 346; 13; (2011); p. 1903 - 1908. |
| F2 | *Carthamus tinctorius* | M016922 | He, Jun; Shen, Yi; Jiang, Jian-Shuang; Yang, Ya-Nan; Feng, Zi-Ming; Zhang, Pei-Cheng; Yuan, Shao-Peng; Hou, Qi; Carbohydrate Research; vol. 346; 13; (2011); p. 1903 - 1908. |
| F2 | *Carthamus tinctorius* | M016923 | He, Jun; Shen, Yi; Jiang, Jian-Shuang; Yang, Ya-Nan; Feng, Zi-Ming; Zhang, Pei-Cheng; Yuan, Shao-Peng; Hou, Qi; Carbohydrate Research; vol. 346; 13; (2011); p. 1903 - 1908. |
| F2 | *Carthamus tinctorius* | M016924 | He, Jun; Shen, Yi; Jiang, Jian-Shuang; Yang, Ya-Nan; Feng, Zi-Ming; Zhang, Pei-Cheng; Yuan, Shao-Peng; Hou, Qi; Carbohydrate Research; vol. 346; 13; (2011); p. 1903 - 1908. |
| F2 | *Carthamus tinctorius* | M016925 | He, Jun; Shen, Yi; Jiang, Jian-Shuang; Yang, Ya-Nan; Feng, Zi-Ming; Zhang, Pei-Cheng; Yuan, Shao-Peng; Hou, Qi; Carbohydrate Research; vol. 346; 13; (2011); p. 1903 - 1908. |
| F2 | *Carthamus tinctorius* | M017651 | Retrieved from CNPD |
| F2 | *Carthamus tinctorius* | M017719 | Roh, Jung Seop; Han, Ji Young; Kim, Jung Han; Hwang, Jae Kwan; Biological and Pharmaceutical Bulletin; vol. 27; 12; (2004); p. 1976 - 1978. |
| F2 | *Carthamus tinctorius* | M017752 | Jiang, Jian-Shuang; He, Jun; Feng, Zi-Ming; Zhang, Pei-Cheng; Organic Letters; vol. 12; 6; (2010); p. 1196 - 1199. |
| F2 | *Carthamus tinctorius* | M018049 | Hattori, Masao; Huang, Xin-li; Che, Qing-Ming; Kawata, Yukio; Tezuka, Yasuhiro; et al.; Phytochemistry (Elsevier); vol. 31; 11; (1992); p. 4001 - 4004. |
| F2 | *Carthamus tinctorius* | M018051 | Clementi, Catia; Basconi, Gloria; Pellegrino, Roberto; Romani, Aldo; Dyes and Pigments; vol. 103; (2014); p. 127 - 137. |
| F2 | *Carthamus tinctorius* | M018172 | Ichihara; Noda; Agricultural and Biological Chemistry; vol. 39; (1975); p. 1103,1105,1106. |
| F2 | *Carthamus tinctorius* | M018198 | Jiang, Jian-Shuang; He, Jun; Feng, Zi-Ming; Zhang, Pei-Cheng; Organic Letters; vol. 12; 6; (2010); p. 1196 - 1199. |
| F2 | *Carthamus tinctorius* | M018522 | Yang, Wen-Zhi; Qiao, Xue; Bo, Tao; Wang, Qing; Guo, De-An; Ye, Min; Rapid Communications in Mass Spectrometry; vol. 28; 4; (2014); p. 385 - 395. |
| F2 | *Carthamus tinctorius* | M018610 | Kuliev, A. A.; Gigienova, E. I.; Umarov, A. U.; Kuliev, V. B.; Aslanov, S. M.; Chemistry of Natural Compounds; vol. 18; 1; (1982); p. 32 - 35; Khimiya Prirodnykh Soedinenii; vol. 18; 1; (1982); p. 36 - 40. |
| F2 | *Carthamus tinctorius* | M018863 | Jiang, Jian-Shuang; Chen, Zhong; Yang, Ya-Nan; Feng, Zi-Ming; Zhang, Pei-Cheng; Journal of Asian Natural Products Research; vol. 15; 5; (2013); p. 427 - 432. |
| F2 | *Carthamus tinctorius* | M019053 | Jiang, Jian-Shuang; He, Jun; Feng, Zi-Ming; Zhang, Pei-Cheng; Organic Letters; vol. 12; 6; (2010); p. 1196 - 1199. |
| F2 | *Carthamus tinctorius* | M019127 | Kazuma, Kohei; Takahashi, Takashi; Sato, Katsura; Takeuchi, Hisatomo; Matsumoto, Takeshi; Okuno, Toshikatsu; Bioscience, Biotechnology and Biochemistry; vol. 64; 8; (2000); p. 1588 - 1599. |
| F2 | *Carthamus tinctorius* | M019475 | Jiang, Jian-Shuang; He, Jun; Feng, Zi-Ming; Zhang, Pei-Cheng; Organic Letters; vol. 12; 6; (2010); p. 1196 - 1199. |
| F2 | *Carthamus tinctorius* | M019482 | Ichihara; Noda; Agricultural and Biological Chemistry; vol. 39; (1975); p. 1103,1105,1106. |
| F2 | *Carthamus tinctorius* | M019778 | Yang, Wen-Zhi; Qiao, Xue; Bo, Tao; Wang, Qing; Guo, De-An; Ye, Min; Rapid Communications in Mass Spectrometry; vol. 28; 4; (2014); p. 385 - 395. |
| F2 | *Carthamus tinctorius* | M020184 | Yang, Wen-Zhi; Qiao, Xue; Bo, Tao; Wang, Qing; Guo, De-An; Ye, Min; Rapid Communications in Mass Spectrometry; vol. 28; 4; (2014); p. 385 - 395. |
| F2 | *Carthamus tinctorius* | M020286 | Ichihara; Noda; Agricultural and Biological Chemistry; vol. 39; (1975); p. 1103,1105,1106. |
| F2 | *Carthamus tinctorius* | M020560 | Clementi, Catia; Basconi, Gloria; Pellegrino, Roberto; Romani, Aldo; Dyes and Pigments; vol. 103; (2014); p. 127 - 137. |
| F2 | *Carthamus tinctorius* | M020583 | Yang, Wen-Zhi; Qiao, Xue; Bo, Tao; Wang, Qing; Guo, De-An; Ye, Min; Rapid Communications in Mass Spectrometry; vol. 28; 4; (2014); p. 385 - 395. |
| F2 | *Carthamus tinctorius* | M020679 | Ichihara; Noda; Agricultural and Biological Chemistry; vol. 39; (1975); p. 1103,1105,1106. |
| F2 | *Carthamus tinctorius* | M021020 | Yang, Wen-Zhi; Qiao, Xue; Bo, Tao; Wang, Qing; Guo, De-An; Ye, Min; Rapid Communications in Mass Spectrometry; vol. 28; 4; (2014); p. 385 - 395. |
| F2 | *Carthamus tinctorius* | M021099 | Roh, Jung Seop; Han, Ji Young; Kim, Jung Han; Hwang, Jae Kwan; Biological and Pharmaceutical Bulletin; vol. 27; 12; (2004); p. 1976 - 1978. |
| F2 | *Carthamus tinctorius* | M021119 | Ichihara; Noda; Agricultural and Biological Chemistry; vol. 39; (1975); p. 1103,1105,1106. |
| F2 | *Carthamus tinctorius* | M021136 | Jiang, Jian-Shuang; He, Jun; Feng, Zi-Ming; Zhang, Pei-Cheng; Organic Letters; vol. 12; 6; (2010); p. 1196 - 1199. |
| F2 | *Carthamus tinctorius* | M021585 | Jiang, Jian-Shuang; He, Jun; Feng, Zi-Ming; Zhang, Pei-Cheng; Organic Letters; vol. 12; 6; (2010); p. 1196 - 1199. |
| F2 | *Carthamus tinctorius* | M021756 | Binder et al.; Phytochemistry (Elsevier); vol. 17; (1978); p. 315,317. |
| F2 | *Carthamus tinctorius* | M021892 | Clementi, Catia; Basconi, Gloria; Pellegrino, Roberto; Romani, Aldo; Dyes and Pigments; vol. 103; (2014); p. 127 - 137. |
| F2 | *Carthamus tinctorius* | M021956 | Sakamura, Sadao; Terayama, Yoshihiko; Kawakatsu, Satomi; Ichihara, Akitami; Saito, Hideya; Agricultural and Biological Chemistry; vol. 44; 12; (1980); p. 2951 - 2954. |
| F2 | *Carthamus tinctorius* | M022752 | 阴健等. 中药现代研究与临床应用(1). 北京: 学苑出版社, 1993 |
| F2 | *Carthamus tinctorius* | M023117 | Review; Hui Li Zhang; Nagatsu; Sakakibara; Chemical and Pharmaceutical Bulletin; vol. 44; 4; (1996); p. 874 - 876. |
| F2 | *Carthamus tinctorius* | M023299 | Bohlmann,F. et al.; Chemische Berichte; vol. 99; (1966); p. 3433 - 3436. |
| F2 | *Carthamus tinctorius* | M023309 | Sato, Hiroji; Kawagishi, Hirokazu; Nishimura, Tsutomu; Yoneyama, Syozou; Yoshimoto, Yuko; et al.; Agricultural and Biological Chemistry; vol. 49; 10; (1985); p. 2969 - 2974. |
| F2 | *Carthamus tinctorius* | M023701 | (1) 李艳梅等. 药学学报, 1998, 33 (8): 626. (2) Buckingham J(Executive Editor): et al. Dictionary of Natural Products, Vol 1-7, Chapman & Hall, London, 1994; 1995, Vol 8; 1996, Vol 9; 1997, Vol 10; 1998, Vol 11.. |
| F2 | *Carthamus tinctorius* | M024147 | Cho, Man-Ho; Paik, Young-Sook; Hahn, Tae-Ryong; Journal of Agricultural and Food Chemistry; vol. 48; 9; (2000); p. 3917 - 3921. |
| F2 | *Carthamus tinctorius* | M024544 | Cai, Yi-Zhong; Mei Sun; Jie Xing; Luo, Qiong; Corke, Harold; Life Sciences; vol. 78; 25; (2006); p. 2872 - 2888. |
| F2 | *Carthamus tinctorius* | M024682 | Meselhy; Kadota; Momose; Hattori; Namba; Chemical and pharmaceutical bulletin; vol. 40; 12; (1992); p. 3355 - 3357. |
| F2 | *Carthamus tinctorius* | M024819 | Ahmed; Marzouk; El-Khrisy; Abdel Wahab; El-Din; Pharmazie; vol. 55; 8; (2000); p. 621 - 622. |
| F2 | *Carthamus tinctorius* | M024868 | Nagatsu, Akito; Zhang, Hui Li; Watanabe, Toshihiro; Taniguchi, Nari; Hatano, Keiichiro; Mizukami, Hajime; Sakakibara, Jinsaku; Chemical and Pharmaceutical Bulletin; vol. 46; 6; (1998); p. 1044 - 1047. |
| F2 | *Carthamus tinctorius* | M025138 | Sato, Hiroji; Kawagishi, Hirokazu; Nishimura, Tsutomu; Yoneyama, Syozou; Yoshimoto, Yuko; et al.; Agricultural and Biological Chemistry; vol. 49; 10; (1985); p. 2969 - 2974. |
| F2 | *Carthamus tinctorius* | M025624 | Akihisa, Toshihiro; Yasukawa, Ken; Oinuma, Hirotoshi; Kasahara, Yoshimasa; Yamanouchi, Sakae; Takido, Michio; Kumaki, Kunio; Tamura, Toshitake; Phytochemistry; vol. 43; 6; (1996); p. 1255 - 1260. |
| F2 | *Carthamus tinctorius* | M026053 | He, Jun; Shen, Yi; Jiang, Jian-Shuang; Yang, Ya-Nan; Feng, Zi-Ming; Zhang, Pei-Cheng; Yuan, Shao-Peng; Hou, Qi; Carbohydrate Research; vol. 346; 13; (2011); p. 1903 - 1908. |
| F2 | *Carthamus tinctorius* | M026172 | Akihisa, Toshihiro; Oinuma, Hirotoshi; Tamura, Toshitake; Kasahara, Yoshimasa; Kumaki, Kunio; et al.; Phytochemistry (Elsevier); vol. 36; 1; (1994); p. 105 - 108. |
| F2 | *Carthamus tinctorius* | M026190 | Hattori, Masao; Huang, Xin-li; Che, Qing-Ming; Kawata, Yukio; Tezuka, Yasuhiro; et al.; Phytochemistry (Elsevier); vol. 31; 11; (1992); p. 4001 - 4004. |
| F2 | *Carthamus tinctorius* | M026236 | Hattori, Masao; Huang, Xin-li; Che, Qing-Ming; Kawata, Yukio; Tezuka, Yasuhiro; et al.; Phytochemistry (Elsevier); vol. 31; 11; (1992); p. 4001 - 4004. |
| F2 | *Carthamus tinctorius* | M026277 | Kazuma, Kohei; Takahashi, Takashi; Sato, Katsura; Takeuchi, Hisatomo; Matsumoto, Takeshi; Okuno, Toshikatsu; Bioscience, Biotechnology and Biochemistry; vol. 64; 8; (2000); p. 1588 - 1599. |
| F2 | *Carthamus tinctorius* | M026416 | Retrieved from CNPD |
| F2 | *Carthamus tinctorius* | M026718 | Review; Hui Li Zhang; Nagatsu; Sakakibara; Chemical and Pharmaceutical Bulletin; vol. 44; 4; (1996); p. 874 - 876. |
| F2 | *Carthamus tinctorius* | M026727 | Schlieper; Justus Liebigs Annalen der Chemie; vol. 58; (1846); p. 357,358.; Preisser; Journal fuer Praktische Chemie (Leipzig); vol. <1> 32; (1844); p. 129,141.; Kuroda; Journal of the Chemical Society; (1930); p. 767; Chem. Zentralbl.; vol. 100; II; (1929); p. 432. |
| F2 | *Carthamus tinctorius* | M026804 | Hattori, Masao; Huang, Xin-li; Che, Qing-Ming; Kawata, Yukio; Tezuka, Yasuhiro; et al.; Phytochemistry (Elsevier); vol. 31; 11; (1992); p. 4001 - 4004. |
| F2 | *Carthamus tinctorius* | M027161 | Kazuma, Kohei; Takahashi, Takashi; Sato, Katsura; Takeuchi, Hisatomo; Matsumoto, Takeshi; Okuno, Toshikatsu; Bioscience, Biotechnology and Biochemistry; vol. 64; 8; (2000); p. 1588 - 1599. |
| F2 | *Carthamus tinctorius* | M027231 | Bohlmann,F. et al.; Chemische Berichte; vol. 99; (1966); p. 3433 - 3436.; Kogiso et al.; Tetrahedron Letters; (1976); p. 109. |
| F2 | *Carthamus tinctorius* | M027394 | Meselhy; Kadota; Momose; Hatakeyama; Kusai; Hattori; Namba; Chemical and Pharmaceutical Bulletin; vol. 41; 10; (1993); p. 1796 - 1802. |
| F2 | *Carthamus tinctorius* | M027410 | Akihisa, Toshihiro; Yasukawa, Ken; Oinuma, Hirotoshi; Kasahara, Yoshimasa; Yamanouchi, Sakae; Takido, Michio; Kumaki, Kunio; Tamura, Toshitake; Phytochemistry; vol. 43; 6; (1996); p. 1255 - 1260. |
| F2 | *Carthamus tinctorius* | M027518 | Sakamura, Sadao; Terayama, Yoshihiko; Kawakatsu, Satomi; Ichihara, Akitami; Saito, Hideya; Agricultural and Biological Chemistry; vol. 44; 12; (1980); p. 2951 - 2954. |
| F2 | *Carthamus tinctorius* | M027589 | Akihisa, Toshihiro; Oinuma, Hirotoshi; Tamura, Toshitake; Kasahara, Yoshimasa; Kumaki, Kunio; et al.; Phytochemistry (Elsevier); vol. 36; 1; (1994); p. 105 - 108. |
| F2 | *Carthamus tinctorius* | M027702 | Bohlmann,F. et al.; Chemische Berichte; vol. 99; (1966); p. 3433 - 3436. |
| F2 | *Carthamus tinctorius* | M028060 | Tsutiya; JP4974; (1969); Ref. Zh., Khim.; vol. 5; N600P; (1970). |
| F2 | *Carthamus tinctorius* | M028063 | Suleimanov; Chemistry of Natural Compounds; vol. 40; 1; (2004); p. 13 - 15. |
| F2 | *Carthamus tinctorius* | M028188 | (1) 阴健等. 中药现代研究与临床应用(1). 北京: 学苑出版社, 1993. (2) 孙文基等. 天然活性成分简明手册. 北京: 中国医药科技出版社, 1998. (3) 国家中医药管理局《中华本草》编委会. 中华本草. Vol. 1-30 上海:上海科学技术出版社, 1999. (4) OHASHI K, et al. Chem Pharm Bull, 2003, 51 (3): 343. (5) Morikawa T, et al. JNP, 2002, 65 (10): 1468. (6) Park WS, et al. Planta Med, 2003, 69, 459. |
| F2 | *Carthamus tinctorius* | M029090 | Kuliev, A. A.; Gigienova, E. I.; Umarov, A. U.; Kuliev, V. B.; Aslanov, S. M.; Chemistry of Natural Compounds; vol. 18; 1; (1982); p. 32 - 35; Khimiya Prirodnykh Soedinenii; vol. 18; 1; (1982); p. 36 - 40. |
| F2 | *Carthamus tinctorius* | M029231 | Zhang, Ge; Guo, Mei-Li; Li, Run-Ping; Li, Ying; Zhang, Han-Ming; Su, Zhong-Wu; Chemistry of Natural Compounds; vol. 45; 3; (2009); p. 398 - 401. |
| F2 | *Carthamus tinctorius* | M029357 | Zhou, Yu-Zhi; Ma, Hong-Yu; Chen, Huan; Qiao, Li; Yao, Yao; Cao, Jia-Qing; Pei, Yue-Hu; Chemical and Pharmaceutical Bulletin; vol. 54; 10; (2006); p. 1455 - 1456. |
| F2 | *Carthamus tinctorius* | M029778 | Retrieved from CNPD |
| F2 | *Carthamus tinctorius* | M030148 | Kazuma, Kohei; Shirai, Eriko; Wada, Mizu; Umeo, Kazuhiro; Sato, Atsushi; et al.; Bioscience, Biotechnology, and Biochemistry; vol. 59; 8; (1995); p. 1588 - 1590. |
| F2 | *Carthamus tinctorius* | M030253 | Akihisa, Toshihiro; Oinuma, Hirotoshi; Tamura, Toshitake; Kasahara, Yoshimasa; Kumaki, Kunio; et al.; Phytochemistry (Elsevier); vol. 36; 1; (1994); p. 105 - 108. |
| F2 | *Carthamus tinctorius* | M030400 | (1) 阴健等. 中药现代研究与临床应用(1). 北京: 学苑出版社, 1993. (2) 杨峻山等. 药学学报, 1993, 28 (3): 197. |
| F2 | *Carthamus tinctorius* | M030847 | Schlieper; Justus Liebigs Annalen der Chemie; vol. 58; (1846); p. 357,358.; Preisser; Journal fuer Praktische Chemie (Leipzig); vol. <1> 32; (1844); p. 129,141.; Kametaka; Perkin; Journal of the Chemical Society; vol. 97; (1910); p. 1420.; Kuroda; Journal of the Chemical Society; (1930); p. 767; Chem. Zentralbl.; vol. 100; II; (1929); p. 432. |
| F2 | *Carthamus tinctorius* | M031381 | Zhang, Ge; Guo, Mei-Li; Li, Run-Ping; Li, Ying; Zhang, Han-Ming; Su, Zhong-Wu; Chemistry of Natural Compounds; vol. 45; 3; (2009); p. 398 - 401. |
| F2 | *Carthamus tinctorius* | M031444 | Jiang, Jian-Shuang; He, Jun; Feng, Zi-Ming; Zhang, Pei-Cheng; Organic Letters; vol. 12; 6; (2010); p. 1196 - 1199. |
| F2 | *Carthamus tinctorius* | M031583 | Yang, Wen-Zhi; Qiao, Xue; Bo, Tao; Wang, Qing; Guo, De-An; Ye, Min; Rapid Communications in Mass Spectrometry; vol. 28; 4; (2014); p. 385 - 395. |
| F2 | *Carthamus tinctorius* | M031706 | 国家中医药管理局《中华本草》编委会. 中华本草. Vol. 1-30 上海:上海科学技术出版社, 1999 |
| F2 | *Carthamus tinctorius* | M032021 | Binder et al.; Phytochemistry (Elsevier); vol. 17; (1978); p. 315,317. |
| F2 | *Carthamus tinctorius* | M032246 | Akihisa, Toshihiro; Yasukawa, Ken; Oinuma, Hirotoshi; Kasahara, Yoshimasa; Yamanouchi, Sakae; Takido, Michio; Kumaki, Kunio; Tamura, Toshitake; Phytochemistry; vol. 43; 6; (1996); p. 1255 - 1260. |
| F2 | *Carthamus tinctorius* | M032329 | Binder et al.; Phytochemistry (Elsevier); vol. 17; (1978); p. 315,317. |
| F2 | *Carthamus tinctorius* | M032824 | Sakamura, Sadao; Terayama, Yoshihiko; Kawakatsu, Satomi; Ichihara, Akitami; Saito, Hideya; Agricultural and Biological Chemistry; vol. 44; 12; (1980); p. 2951 - 2954. |
| F2 | *Carthamus tinctorius* | M033212 | Yin, Hong-Bin; He, Zhi-Sheng; Ye, Yang; Journal of Natural Products; vol. 63; 8; (2000); p. 1164 - 1165. |
| F2 | *Carthamus tinctorius* | M033849 | Akihisa, Toshihiro; Oinuma, Hirotoshi; Tamura, Toshitake; Kasahara, Yoshimasa; Kumaki, Kunio; et al.; Phytochemistry (Elsevier); vol. 36; 1; (1994); p. 105 - 108. |
| F2 | *Carthamus tinctorius* | M033909 | Akihisa, Toshihiro; Yasukawa, Ken; Oinuma, Hirotoshi; Kasahara, Yoshimasa; Yamanouchi, Sakae; Takido, Michio; Kumaki, Kunio; Tamura, Toshitake; Phytochemistry; vol. 43; 6; (1996); p. 1255 - 1260. |
| F2 | *Carthamus tinctorius* | M033930 | Kazuma, Kohei; Takahashi, Takashi; Sato, Katsura; Takeuchi, Hisatomo; Matsumoto, Takeshi; Okuno, Toshikatsu; Bioscience, Biotechnology and Biochemistry; vol. 64; 8; (2000); p. 1588 - 1599. |
| F2 | *Carthamus tinctorius* | M033950 | Retrieved from CNPD |
| F2 | *Carthamus tinctorius* | M034045 | Retrieved from CNPD |
| F2 | *Carthamus tinctorius* | M034061 | Akihisa, Toshihiro; Yasukawa, Ken; Oinuma, Hirotoshi; Kasahara, Yoshimasa; Yamanouchi, Sakae; Takido, Michio; Kumaki, Kunio; Tamura, Toshitake; Phytochemistry; vol. 43; 6; (1996); p. 1255 - 1260. |
| F2 | *Carthamus tinctorius* | M034353 | Saunders; Journal of the American Oil Chemists' Society; vol. 47; (1970); p. 255. |
| F2 | *Curcuma kwangsiensis* | M001223 | Li, Jun; Zhao, Feng; Li, Ming Zhi; Chen, Li Xia; Qiu, Feng; Journal of Natural Products; vol. 73; 10; (2010); p. 1667 - 1671. |
| F2 | *Curcuma kwangsiensis* | M001456 | Li, Jun; Zhao, Feng; Li, Ming Zhi; Chen, Li Xia; Qiu, Feng; Journal of Natural Products; vol. 73; 10; (2010); p. 1667 - 1671. |
| F2 | *Curcuma kwangsiensis* | M003505 | Li, Jun; Zhao, Feng; Li, Ming Zhi; Chen, Li Xia; Qiu, Feng; Journal of Natural Products; vol. 73; 10; (2010); p. 1667 - 1671. |
| F2 | *Curcuma kwangsiensis* | M005125 | Li, Jun; Liao, Chun-Ru; Wei, Jun-Qi; Chen, Li-Xia; Zhao, Feng; Qiu, Feng; Bioorganic and Medicinal Chemistry Letters; vol. 21; 18; (2011); p. 5363 - 5369. |
| F2 | *Curcuma kwangsiensis* | M006116 | Li, Jun; Liao, Chun-Ru; Wei, Jun-Qi; Chen, Li-Xia; Zhao, Feng; Qiu, Feng; Bioorganic and Medicinal Chemistry Letters; vol. 21; 18; (2011); p. 5363 - 5369. |
| F2 | *Curcuma kwangsiensis* | M007413 | Schramm, Anja; Ebrahimi, Samad Nejad; Raith, Melanie; Zaugg, Janine; Rueda, Diana C.; Hering, Steffen; Hamburger, Matthias; Phytochemistry; vol. 96; (2013); p. 318 - 329. |
| F2 | *Curcuma kwangsiensis* | M007440 | Li, Jun; Zhao, Feng; Li, Ming Zhi; Chen, Li Xia; Qiu, Feng; Journal of Natural Products; vol. 73; 10; (2010); p. 1667 - 1671. |
| F2 | *Curcuma kwangsiensis* | M008419 | Li, Jun; Zhao, Feng; Li, Ming Zhi; Chen, Li Xia; Qiu, Feng; Journal of Natural Products; vol. 73; 10; (2010); p. 1667 - 1671. |
| F2 | *Curcuma kwangsiensis* | M009649 | Schramm, Anja; Ebrahimi, Samad Nejad; Raith, Melanie; Zaugg, Janine; Rueda, Diana C.; Hering, Steffen; Hamburger, Matthias; Phytochemistry; vol. 96; (2013); p. 318 - 329. |
| F2 | *Curcuma kwangsiensis* | M010492 | Li, Jun; Zhao, Feng; Li, Ming Zhi; Chen, Li Xia; Qiu, Feng; Journal of Natural Products; vol. 73; 10; (2010); p. 1667 - 1671. |
| F2 | *Curcuma kwangsiensis* | M010539 | Li, Jun; Zhao, Feng; Li, Ming Zhi; Chen, Li Xia; Qiu, Feng; Journal of Natural Products; vol. 73; 10; (2010); p. 1667 - 1671. |
| F2 | *Curcuma kwangsiensis* | M012499 | Li, Jun; Zhao, Feng; Li, Ming Zhi; Chen, Li Xia; Qiu, Feng; Journal of Natural Products; vol. 73; 10; (2010); p. 1667 - 1671. |
| F2 | *Curcuma kwangsiensis* | M013103 | Schramm, Anja; Ebrahimi, Samad Nejad; Raith, Melanie; Zaugg, Janine; Rueda, Diana C.; Hering, Steffen; Hamburger, Matthias; Phytochemistry; vol. 96; (2013); p. 318 - 329. |
| F2 | *Curcuma kwangsiensis* | M014889 | Li, Jun; Zhao, Feng; Li, Ming Zhi; Chen, Li Xia; Qiu, Feng; Journal of Natural Products; vol. 73; 10; (2010); p. 1667 - 1671. |
| F2 | *Curcuma kwangsiensis* | M015667 | Li, Jun; Zhao, Feng; Li, Ming Zhi; Chen, Li Xia; Qiu, Feng; Journal of Natural Products; vol. 73; 10; (2010); p. 1667 - 1671. |
| F2 | *Curcuma kwangsiensis* | M016223 | Li, Jun; Zhao, Feng; Li, Ming Zhi; Chen, Li Xia; Qiu, Feng; Journal of Natural Products; vol. 73; 10; (2010); p. 1667 - 1671. |
| F2 | *Curcuma kwangsiensis* | M016754 | Li, Jun; Liao, Chun-Ru; Wei, Jun-Qi; Chen, Li-Xia; Zhao, Feng; Qiu, Feng; Bioorganic and Medicinal Chemistry Letters; vol. 21; 18; (2011); p. 5363 - 5369. |
| F2 | *Curcuma kwangsiensis* | M016755 | Li, Jun; Liao, Chun-Ru; Wei, Jun-Qi; Chen, Li-Xia; Zhao, Feng; Qiu, Feng; Bioorganic and Medicinal Chemistry Letters; vol. 21; 18; (2011); p. 5363 - 5369. |
| F2 | *Curcuma kwangsiensis* | M016756 | Li, Jun; Liao, Chun-Ru; Wei, Jun-Qi; Chen, Li-Xia; Zhao, Feng; Qiu, Feng; Bioorganic and Medicinal Chemistry Letters; vol. 21; 18; (2011); p. 5363 - 5369. |
| F2 | *Curcuma kwangsiensis* | M016757 | Li, Jun; Liao, Chun-Ru; Wei, Jun-Qi; Chen, Li-Xia; Zhao, Feng; Qiu, Feng; Bioorganic and Medicinal Chemistry Letters; vol. 21; 18; (2011); p. 5363 - 5369. |
| F2 | *Curcuma kwangsiensis* | M016758 | Li, Jun; Liao, Chun-Ru; Wei, Jun-Qi; Chen, Li-Xia; Zhao, Feng; Qiu, Feng; Bioorganic and Medicinal Chemistry Letters; vol. 21; 18; (2011); p. 5363 - 5369. |
| F2 | *Curcuma kwangsiensis* | M016759 | Li, Jun; Liao, Chun-Ru; Wei, Jun-Qi; Chen, Li-Xia; Zhao, Feng; Qiu, Feng; Bioorganic and Medicinal Chemistry Letters; vol. 21; 18; (2011); p. 5363 - 5369. |
| F2 | *Curcuma kwangsiensis* | M016760 | Li, Jun; Liao, Chun-Ru; Wei, Jun-Qi; Chen, Li-Xia; Zhao, Feng; Qiu, Feng; Bioorganic and Medicinal Chemistry Letters; vol. 21; 18; (2011); p. 5363 - 5369. |
| F2 | *Curcuma kwangsiensis* | M016761 | Li, Jun; Liao, Chun-Ru; Wei, Jun-Qi; Chen, Li-Xia; Zhao, Feng; Qiu, Feng; Bioorganic and Medicinal Chemistry Letters; vol. 21; 18; (2011); p. 5363 - 5369. |
| F2 | *Curcuma kwangsiensis* | M016762 | Li, Jun; Liao, Chun-Ru; Wei, Jun-Qi; Chen, Li-Xia; Zhao, Feng; Qiu, Feng; Bioorganic and Medicinal Chemistry Letters; vol. 21; 18; (2011); p. 5363 - 5369. |
| F2 | *Curcuma kwangsiensis* | M016763 | Li, Jun; Liao, Chun-Ru; Wei, Jun-Qi; Chen, Li-Xia; Zhao, Feng; Qiu, Feng; Bioorganic and Medicinal Chemistry Letters; vol. 21; 18; (2011); p. 5363 - 5369. |
| F2 | *Curcuma kwangsiensis* | M017642 | Li, Jun; Liao, Chun-Ru; Wei, Jun-Qi; Chen, Li-Xia; Zhao, Feng; Qiu, Feng; Bioorganic and Medicinal Chemistry Letters; vol. 21; 18; (2011); p. 5363 - 5369. |
| F2 | *Curcuma kwangsiensis* | M017756 | Schramm, Anja; Ebrahimi, Samad Nejad; Raith, Melanie; Zaugg, Janine; Rueda, Diana C.; Hering, Steffen; Hamburger, Matthias; Phytochemistry; vol. 96; (2013); p. 318 - 329. |
| F2 | *Curcuma kwangsiensis* | M017778 | Schramm, Anja; Ebrahimi, Samad Nejad; Raith, Melanie; Zaugg, Janine; Rueda, Diana C.; Hering, Steffen; Hamburger, Matthias; Phytochemistry; vol. 96; (2013); p. 318 - 329. |
| F2 | *Curcuma kwangsiensis* | M020175 | Schramm, Anja; Ebrahimi, Samad Nejad; Raith, Melanie; Zaugg, Janine; Rueda, Diana C.; Hering, Steffen; Hamburger, Matthias; Phytochemistry; vol. 96; (2013); p. 318 - 329. |
| F2 | *Curcuma kwangsiensis* | M020570 | Schramm, Anja; Ebrahimi, Samad Nejad; Raith, Melanie; Zaugg, Janine; Rueda, Diana C.; Hering, Steffen; Hamburger, Matthias; Phytochemistry; vol. 96; (2013); p. 318 - 329. |
| F2 | *Curcuma kwangsiensis* | M021008 | Schramm, Anja; Ebrahimi, Samad Nejad; Raith, Melanie; Zaugg, Janine; Rueda, Diana C.; Hering, Steffen; Hamburger, Matthias; Phytochemistry; vol. 96; (2013); p. 318 - 329. |
| F2 | *Curcuma kwangsiensis* | M023355 | Li, Jun; Zhao, Feng; Li, Ming Zhi; Chen, Li Xia; Qiu, Feng; Journal of Natural Products; vol. 73; 10; (2010); p. 1667 - 1671. |
| F2 | *Curcuma kwangsiensis* | M023549 | Li, Jun; Zhao, Feng; Li, Ming Zhi; Chen, Li Xia; Qiu, Feng; Journal of Natural Products; vol. 73; 10; (2010); p. 1667 - 1671. |
| F2 | *Curcuma kwangsiensis* | M024142 | Schramm, Anja; Ebrahimi, Samad Nejad; Raith, Melanie; Zaugg, Janine; Rueda, Diana C.; Hering, Steffen; Hamburger, Matthias; Phytochemistry; vol. 96; (2013); p. 318 - 329. |
| F2 | *Curcuma kwangsiensis* | M027287 | Li, Jun; Zhao, Feng; Li, Ming Zhi; Chen, Li Xia; Qiu, Feng; Journal of Natural Products; vol. 73; 10; (2010); p. 1667 - 1671. |
| F2 | *Curcuma kwangsiensis* | M028500 | Li, Jun; Zhao, Feng; Li, Ming Zhi; Chen, Li Xia; Qiu, Feng; Journal of Natural Products; vol. 73; 10; (2010); p. 1667 - 1671. |
| F2 | *Curcuma kwangsiensis* | M028788 | Li, Jun; Liao, Chun-Ru; Wei, Jun-Qi; Chen, Li-Xia; Zhao, Feng; Qiu, Feng; Bioorganic and Medicinal Chemistry Letters; vol. 21; 18; (2011); p. 5363 - 5369. |
| F2 | *Curcuma kwangsiensis* | M029491 | Li, Jun; Zhao, Feng; Li, Ming Zhi; Chen, Li Xia; Qiu, Feng; Journal of Natural Products; vol. 73; 10; (2010); p. 1667 - 1671. |
| F2 | *Curcuma kwangsiensis* | M029639 | Li, Jun; Liao, Chun-Ru; Wei, Jun-Qi; Chen, Li-Xia; Zhao, Feng; Qiu, Feng; Bioorganic and Medicinal Chemistry Letters; vol. 21; 18; (2011); p. 5363 - 5369. |
| F2 | *Curcuma kwangsiensis* | M030901 | Li, Jun; Zhao, Feng; Li, Ming Zhi; Chen, Li Xia; Qiu, Feng; Journal of Natural Products; vol. 73; 10; (2010); p. 1667 - 1671. |
| F2 | *Curcuma kwangsiensis* | M031133 | Sasaki, Yohei; Goto, Hirozo; Tohda, Chihiro; Hatanaka, Fumiyuki; Shibahara, Naotoshi; Shimada, Yutaka; Terasawa, Katsutoshi; Komatsu, Katsuko; Biological and pharmaceutical bulletin; vol. 26; 8; (2003); p. 1135 - 1143. |
| F2 | *Curcuma kwangsiensis* | M031690 | Li, Jun; Liao, Chun-Ru; Wei, Jun-Qi; Chen, Li-Xia; Zhao, Feng; Qiu, Feng; Bioorganic and Medicinal Chemistry Letters; vol. 21; 18; (2011); p. 5363 - 5369. |
| F2 | *Curcuma kwangsiensis* | M032479 | Li, Jun; Zhao, Feng; Li, Ming Zhi; Chen, Li Xia; Qiu, Feng; Journal of Natural Products; vol. 73; 10; (2010); p. 1667 - 1671. |
| F2 | *Curcuma wenyujin* | M001446 | Dong, Jian-Yong; Ma, Xi-Yan; Cai, Xiao-Qin; Yan, Peng-Cheng; Yue, Lei; Lin, Chen; Shao, Wei-Wei; Phytochemistry; vol. 85; (2013); p. 122 - 128. |
| F2 | *Curcuma wenyujin* | M002315 | Harimaya; Gao; Ohkura; Kawamata; Iitaka; Guo; Inayama; Chemical and Pharmaceutical Bulletin; vol. 39; 4; (1991); p. 843 - 853. |
| F2 | *Curcuma wenyujin* | M002514 | Harimaya; Gao; Ohkura; Kawamata; Iitaka; Guo; Inayama; Chemical and Pharmaceutical Bulletin; vol. 39; 4; (1991); p. 843 - 853.; Gao; Xie; Harimaya; Kawamata; Iitaka; Inayama; Chemical and Pharmaceutical Bulletin; vol. 39; 4; (1991); p. 854 - 856. |
| F2 | *Curcuma wenyujin* | M002675 | Lou, Yan; Zhao, Feng; Wu, Zhaohua; Peng, Kai-Feng; Wei, Xing-Chuan; Chen, Li-Xia; Qiu, Feng; Helvetica Chimica Acta; vol. 92; 8; (2009); p. 1665 - 1672. |
| F2 | *Curcuma wenyujin* | M002715 | Lou, Yan; Zhao, Feng; Wu, Zhaohua; Peng, Kai-Feng; Wei, Xing-Chuan; Chen, Li-Xia; Qiu, Feng; Helvetica Chimica Acta; vol. 92; 8; (2009); p. 1665 - 1672. |
| F2 | *Curcuma wenyujin* | M003149 | Gao; Xie; Harimaya; Kawamata; Iitaka; Inayama; Chemical and Pharmaceutical Bulletin; vol. 39; 4; (1991); p. 854 - 856. |
| F2 | *Curcuma wenyujin* | M004543 | Ma, Zhong-Jun; Meng, Zhao-Ke; Zhang, Peng; Fitoterapia; vol. 80; 6; (2009); p. 374 - 376. |
| F2 | *Curcuma wenyujin* | M005755 | Dong, Jian-Yong; Ma, Xi-Yan; Cai, Xiao-Qin; Yan, Peng-Cheng; Yue, Lei; Lin, Chen; Shao, Wei-Wei; Phytochemistry; vol. 85; (2013); p. 122 - 128. |
| F2 | *Curcuma wenyujin* | M005928 | Yin, Guo-Ping; An, Yue-Wei; Hu, Guang; Zhu, Jing-Jing; Chen, Liang-Mian; Li, Liang-Chun; Wang, Zhi-Min; Journal of Asian Natural Products Research; vol. 15; 7; (2013); p. 723 - 730. |
| F2 | *Curcuma wenyujin* | M007819 | Gao, Ji-Fu; Xie, Ji-Hong; Iitaka, Yoichi; Inayama, Seiichi; Chemical & Pharmaceutical Bulletin; vol. 37; 1; (1989); p. 233 - 236. |
| F2 | *Curcuma wenyujin* | M008040 | Harimaya; Gao; Ohkura; Kawamata; Iitaka; Guo; Inayama; Chemical and Pharmaceutical Bulletin; vol. 39; 4; (1991); p. 843 - 853. |
| F2 | *Curcuma wenyujin* | M008448 | Retrieved from CNPD |
| F2 | *Curcuma wenyujin* | M009362 | Gao, Ji-Fu; Xie, Ji-Hong; Iitaka, Yoichi; Inayama, Seiichi; Chemical & Pharmaceutical Bulletin; vol. 37; 1; (1989); p. 233 - 236. |
| F2 | *Curcuma wenyujin* | M010347 | Harimaya; Gao; Ohkura; Kawamata; Iitaka; Guo; Inayama; Chemical and Pharmaceutical Bulletin; vol. 39; 4; (1991); p. 843 - 853. |
| F2 | *Curcuma wenyujin* | M011425 | Lou, Yan; Zhao, Feng; Wu, Zhaohua; Peng, Kai-Feng; Wei, Xing-Chuan; Chen, Li-Xia; Qiu, Feng; Helvetica Chimica Acta; vol. 92; 8; (2009); p. 1665 - 1672. |
| F2 | *Curcuma wenyujin* | M011489 | Lou, Yan; Zhao, Feng; Wu, Zhaohua; Peng, Kai-Feng; Wei, Xing-Chuan; Chen, Li-Xia; Qiu, Feng; Helvetica Chimica Acta; vol. 92; 8; (2009); p. 1665 - 1672. |
| F2 | *Curcuma wenyujin* | M011692 | Retrieved from CNPD |
| F2 | *Curcuma wenyujin* | M012738 | Huang, Wei; Zhang, Peng; Jin, Ye-Cheng; Shi, Qiang; Cheng, Yi-Yu; Qu, Hai-Bin; Ma, Zhong-Jun; Helvetica Chimica Acta; vol. 91; 5; (2008); p. 944 - 950. |
| F2 | *Curcuma wenyujin* | M012862 | Huang, Wei; Zhang, Peng; Jin, Ye-Cheng; Shi, Qiang; Cheng, Yi-Yu; Qu, Hai-Bin; Ma, Zhong-Jun; Helvetica Chimica Acta; vol. 91; 5; (2008); p. 944 - 950. |
| F2 | *Curcuma wenyujin* | M014330 | Qiu, Guanguan; Yan, Pengcheng; Shao, Weiwei; Zhou, Jie; Lin, Weiwei; Fang, Lianglian; Zhao, Xiaowei; Dong, Jianyong; Chemical and Pharmaceutical Bulletin; vol. 61; 9; (2013); p. 983 - 986. |
| F2 | *Curcuma wenyujin* | M015141 | Lou, Yan; Zhao, Feng; Wu, Zhaohua; Peng, Kai-Feng; Wei, Xing-Chuan; Chen, Li-Xia; Qiu, Feng; Helvetica Chimica Acta; vol. 92; 8; (2009); p. 1665 - 1672. |
| F2 | *Curcuma wenyujin* | M015839 | Lou, Yan; Zhao, Feng; He, Hao; Peng, Kai-Feng; Chen, Li-Xia; Qiu, Feng; Chemistry and Biodiversity; vol. 7; 5; (2010); p. 1245 - 1253. |
| F2 | *Curcuma wenyujin* | M016368 | Ma, Zhong-Jun; Meng, Zhao-Ke; Zhang, Peng; Fitoterapia; vol. 80; 6; (2009); p. 374 - 376. |
| F2 | *Curcuma wenyujin* | M016369 | Ma, Zhong-Jun; Meng, Zhao-Ke; Zhang, Peng; Fitoterapia; vol. 80; 6; (2009); p. 374 - 376. |
| F2 | *Curcuma wenyujin* | M016370 | Ma, Zhong-Jun; Meng, Zhao-Ke; Zhang, Peng; Fitoterapia; vol. 80; 6; (2009); p. 374 - 376. |
| F2 | *Curcuma wenyujin* | M016617 | Lou, Yan; Zhao, Feng; He, Hao; Peng, Kai-Feng; Chen, Li-Xia; Qiu, Feng; Chemistry and Biodiversity; vol. 7; 5; (2010); p. 1245 - 1253. |
| F2 | *Curcuma wenyujin* | M016618 | Lou, Yan; Zhao, Feng; He, Hao; Peng, Kai-Feng; Chen, Li-Xia; Qiu, Feng; Chemistry and Biodiversity; vol. 7; 5; (2010); p. 1245 - 1253. |
| F2 | *Curcuma wenyujin* | M016619 | Lou, Yan; Zhao, Feng; He, Hao; Peng, Kai-Feng; Chen, Li-Xia; Qiu, Feng; Chemistry and Biodiversity; vol. 7; 5; (2010); p. 1245 - 1253. |
| F2 | *Curcuma wenyujin* | M016620 | Lou, Yan; Zhao, Feng; He, Hao; Peng, Kai-Feng; Chen, Li-Xia; Qiu, Feng; Chemistry and Biodiversity; vol. 7; 5; (2010); p. 1245 - 1253. |
| F2 | *Curcuma wenyujin* | M016622 | Lou, Yan; Zhao, Feng; He, Hao; Peng, Kai-Feng; Chen, Li-Xia; Qiu, Feng; Chemistry and Biodiversity; vol. 7; 5; (2010); p. 1245 - 1253. |
| F2 | *Curcuma wenyujin* | M016623 | Lou, Yan; Zhao, Feng; He, Hao; Peng, Kai-Feng; Chen, Li-Xia; Qiu, Feng; Chemistry and Biodiversity; vol. 7; 5; (2010); p. 1245 - 1253. |
| F2 | *Curcuma wenyujin* | M017888 | Dong, Jian-Yong; Ma, Xi-Yan; Cai, Xiao-Qin; Yan, Peng-Cheng; Yue, Lei; Lin, Chen; Shao, Wei-Wei; Phytochemistry; vol. 85; (2013); p. 122 - 128. |
| F2 | *Curcuma wenyujin* | M017967 | Yin, Guo-Ping; An, Yue-Wei; Hu, Guang; Zhu, Jing-Jing; Chen, Liang-Mian; Li, Liang-Chun; Wang, Zhi-Min; Journal of Asian Natural Products Research; vol. 15; 7; (2013); p. 723 - 730. |
| F2 | *Curcuma wenyujin* | M018335 | Dong, Jian-Yong; Ma, Xi-Yan; Cai, Xiao-Qin; Yan, Peng-Cheng; Yue, Lei; Lin, Chen; Shao, Wei-Wei; Phytochemistry; vol. 85; (2013); p. 122 - 128. |
| F2 | *Curcuma wenyujin* | M018413 | Yin, Guo-Ping; An, Yue-Wei; Hu, Guang; Zhu, Jing-Jing; Chen, Liang-Mian; Li, Liang-Chun; Wang, Zhi-Min; Journal of Asian Natural Products Research; vol. 15; 7; (2013); p. 723 - 730. |
| F2 | *Curcuma wenyujin* | M018765 | Dong, Jian-Yong; Ma, Xi-Yan; Cai, Xiao-Qin; Yan, Peng-Cheng; Yue, Lei; Lin, Chen; Shao, Wei-Wei; Phytochemistry; vol. 85; (2013); p. 122 - 128. |
| F2 | *Curcuma wenyujin* | M019189 | Dong, Jian-Yong; Ma, Xi-Yan; Cai, Xiao-Qin; Yan, Peng-Cheng; Yue, Lei; Lin, Chen; Shao, Wei-Wei; Phytochemistry; vol. 85; (2013); p. 122 - 128. |
| F2 | *Curcuma wenyujin* | M020412 | Dong, Jian-Yong; Ma, Xi-Yan; Cai, Xiao-Qin; Yan, Peng-Cheng; Yue, Lei; Lin, Chen; Shao, Wei-Wei; Phytochemistry; vol. 85; (2013); p. 122 - 128. |
| F2 | *Curcuma wenyujin* | M020837 | Dong, Jian-Yong; Ma, Xi-Yan; Cai, Xiao-Qin; Yan, Peng-Cheng; Yue, Lei; Lin, Chen; Shao, Wei-Wei; Phytochemistry; vol. 85; (2013); p. 122 - 128. |
| F2 | *Curcuma wenyujin* | M020957 | Qiu, Guanguan; Yan, Pengcheng; Shao, Weiwei; Zhou, Jie; Lin, Weiwei; Fang, Lianglian; Zhao, Xiaowei; Dong, Jianyong; Chemical and Pharmaceutical Bulletin; vol. 61; 9; (2013); p. 983 - 986. |
| F2 | *Curcuma wenyujin* | M021286 | Dong, Jian-Yong; Ma, Xi-Yan; Cai, Xiao-Qin; Yan, Peng-Cheng; Yue, Lei; Lin, Chen; Shao, Wei-Wei; Phytochemistry; vol. 85; (2013); p. 122 - 128. |
| F2 | *Curcuma wenyujin* | M021559 | Yin, Guo-Ping; An, Yue-Wei; Hu, Guang; Zhu, Jing-Jing; Chen, Liang-Mian; Li, Liang-Chun; Wang, Zhi-Min; Journal of Asian Natural Products Research; vol. 15; 7; (2013); p. 723 - 730. |
| F2 | *Curcuma wenyujin* | M021724 | Dong, Jian-Yong; Ma, Xi-Yan; Cai, Xiao-Qin; Yan, Peng-Cheng; Yue, Lei; Lin, Chen; Shao, Wei-Wei; Phytochemistry; vol. 85; (2013); p. 122 - 128. |
| F2 | *Curcuma wenyujin* | M021809 | Yin, Guo-Ping; An, Yue-Wei; Hu, Guang; Zhu, Jing-Jing; Chen, Liang-Mian; Li, Liang-Chun; Wang, Zhi-Min; Journal of Asian Natural Products Research; vol. 15; 7; (2013); p. 723 - 730. |
| F2 | *Curcuma wenyujin* | M021845 | Qiu, Guanguan; Yan, Pengcheng; Shao, Weiwei; Zhou, Jie; Lin, Weiwei; Fang, Lianglian; Zhao, Xiaowei; Dong, Jianyong; Chemical and Pharmaceutical Bulletin; vol. 61; 9; (2013); p. 983 - 986. |
| F2 | *Curcuma wenyujin* | M023151 | Lou, Yan; Zhao, Feng; Wu, Zhaohua; Peng, Kai-Feng; Wei, Xing-Chuan; Chen, Li-Xia; Qiu, Feng; Helvetica Chimica Acta; vol. 92; 8; (2009); p. 1665 - 1672. |
| F2 | *Curcuma wenyujin* | M023434 | Harimaya; Gao; Ohkura; Kawamata; Iitaka; Guo; Inayama; Chemical and Pharmaceutical Bulletin; vol. 39; 4; (1991); p. 843 - 853. |
| F2 | *Curcuma wenyujin* | M025752 | Yin, Guo-Ping; An, Yue-Wei; Hu, Guang; Zhu, Jing-Jing; Chen, Liang-Mian; Li, Liang-Chun; Wang, Zhi-Min; Journal of Asian Natural Products Research; vol. 15; 7; (2013); p. 723 - 730. |
| F2 | *Curcuma wenyujin* | M026072 | Inayama, Seiichi; Gao, Ji-Fu; Hariyama, Kenzo; Hikichi, Manabu; Iitaka, Yoichi; et al.; Chemical & Pharmaceutical Bulletin; vol. 33; 5; (1985); p. 2179 - 2182. |
| F2 | *Curcuma wenyujin* | M026693 | Huang, Wei; Zhang, Peng; Jin, Ye-Cheng; Shi, Qiang; Cheng, Yi-Yu; Qu, Hai-Bin; Ma, Zhong-Jun; Helvetica Chimica Acta; vol. 91; 5; (2008); p. 944 - 950. |
| F2 | *Curcuma wenyujin* | M027638 | Harimaya; Gao; Ohkura; Kawamata; Iitaka; Guo; Inayama; Chemical and Pharmaceutical Bulletin; vol. 39; 4; (1991); p. 843 - 853. |
| F2 | *Curcuma wenyujin* | M028864 | Lou, Yan; Zhao, Feng; He, Hao; Peng, Kai-Feng; Chen, Li-Xia; Qiu, Feng; Chemistry and Biodiversity; vol. 7; 5; (2010); p. 1245 - 1253. |
| F2 | *Curcuma wenyujin* | M029283 | Lou, Yan; Zhao, Feng; Wu, Zhaohua; Peng, Kai-Feng; Wei, Xing-Chuan; Chen, Li-Xia; Qiu, Feng; Helvetica Chimica Acta; vol. 92; 8; (2009); p. 1665 - 1672. |
| F2 | *Curcuma wenyujin* | M029736 | Sasaki, Yohei; Goto, Hirozo; Tohda, Chihiro; Hatanaka, Fumiyuki; Shibahara, Naotoshi; Shimada, Yutaka; Terasawa, Katsutoshi; Komatsu, Katsuko; Biological and pharmaceutical bulletin; vol. 26; 8; (2003); p. 1135 - 1143. |
| F2 | *Curcuma wenyujin* | M031034 | Lou, Yan; Zhao, Feng; Wu, Zhaohua; Peng, Kai-Feng; Wei, Xing-Chuan; Chen, Li-Xia; Qiu, Feng; Helvetica Chimica Acta; vol. 92; 8; (2009); p. 1665 - 1672. |
| F2 | *Curcuma wenyujin* | M031132 | Lou, Yan; Zhao, Feng; He, Hao; Peng, Kai-Feng; Chen, Li-Xia; Qiu, Feng; Chemistry and Biodiversity; vol. 7; 5; (2010); p. 1245 - 1253. |
| F2 | *Cyperus rotundus* | M000034 | Thebtaranonth; Wanauppathamkul; Yuthavong; Phytochemistry; vol. 40; 1; (1995); p. 125 - 128. |
| F2 | *Cyperus rotundus* | M000806 | Hikino,H. et al.; Chemical and Pharmaceutical Bulletin; vol. 16; 1; (1968); p. 52 - 55. |
| F2 | *Cyperus rotundus* | M000944 | Yang, Jun-Li; Shi, Yan-Ping; Planta Medica; vol. 78; 1; (2012); p. 59 - 64. |
| F2 | *Cyperus rotundus* | M001099 | Ito, Tetsuro; Endo, Hidetatsu; Shinohara, Haruka; Oyama, Masayoshi; Akao, Yukihiro; Iinuma, Munekazu; Fitoterapia; vol. 83; 8; (2012); p. 1420 - 1429. |
| F2 | *Cyperus rotundus* | M001187 | Seo, Eun Ji; Lee, Dong-Ung; Kwak, Jong Hwan; Lee, Sun-Mee; Kim, Yeong Shik; Jung, Yi-Sook; Journal of Ethnopharmacology; vol. 135; 1; (2011); p. 48 - 54. |
| F2 | *Cyperus rotundus* | M001829 | Yang, Jun-Li; Shi, Yan-Ping; Planta Medica; vol. 78; 1; (2012); p. 59 - 64. |
| F2 | *Cyperus rotundus* | M002005 | Kim, Su Jung; Kim, Hyun Ji; Kim, Hye Jin; Jang, Young Pyo; Oh, Myung Sook; Jang, Dae Sik; Bulletin of the Korean Chemical Society; vol. 33; 9; (2012); p. 3115 - 3118. |
| F2 | *Cyperus rotundus* | M002136 | Sonwa, Mesmin Mekem; Koenig, Wilfried A.; Phytochemistry; vol. 58; 5; (2001); p. 799 - 810. |
| F2 | *Cyperus rotundus* | M002793 | Jeong, Sei-Joon; Miyamoto, Tomofumi; Inagaki, Masanori; Kim, Youn-Chul; Higuchi, Ryuichi; Journal of Natural Products; vol. 63; 5; (2000); p. 673 - 675. |
| F2 | *Cyperus rotundus* | M003058 | Hikino et al.; Chemical and Pharmaceutical Bulletin; vol. 14; (1966); p. 1439. |
| F2 | *Cyperus rotundus* | M003198 | Zhou, Zhongliu; Fu, Chunyan; Chemistry of Natural Compounds; vol. 48; 6; (2013); p. 963 - 965. |
| F2 | *Cyperus rotundus* | M003334 | Thebtaranonth; Wanauppathamkul; Yuthavong; Phytochemistry; vol. 40; 1; (1995); p. 125 - 128. |
| F2 | *Cyperus rotundus* | M003470 | Singh, P. N.; Singh, S. B.; Phytochemistry (Elsevier); vol. 19; (1980); p. 2056 - 2058. |
| F2 | *Cyperus rotundus* | M003518 | Seo, Eun Ji; Lee, Dong-Ung; Kwak, Jong Hwan; Lee, Sun-Mee; Kim, Yeong Shik; Jung, Yi-Sook; Journal of Ethnopharmacology; vol. 135; 1; (2011); p. 48 - 54. |
| F2 | *Cyperus rotundus* | M003876 | Trivedi,B. et al.; Collection of Czechoslovak Chemical Communications; vol. 29; (1964); p. 1675 - 1688. |
| F2 | *Cyperus rotundus* | M004122 | Kim, Su Jung; Ryu, Byeol; Kim, Ha-Yeong; Yang, Yeong-In; Ham, Jungyeob; Choi, Jung-Hye; Jang, Dae Sik; Bulletin of the Korean Chemical Society; vol. 34; 7; (2013); p. 2207 - 2210. |
| F2 | *Cyperus rotundus* | M004622 | Ha, Jeoung-Hee; Lee, Kwang-Youn; Choi, Hyoung-Chul; Cho, Jungsook; Kang, Byung-Soo; Lim, Jae-Chul; Lee, Dong-Ung; Biological and pharmaceutical bulletin; vol. 25; 1; (2002); p. 128 - 130. |
| F2 | *Cyperus rotundus* | M004774 | Ohira, Susumu; Hasegawa, Taisuke; Hayashi, Ken-Ichiro; Hoshino, Takuji; Takaoka, Daisuke; Nozaki, Hiroshi; Phytochemistry; vol. 47; 8; (1998); p. 1577 - 1581. |
| F2 | *Cyperus rotundus* | M005236 | Komai, Koichiro; Tang, Chung-Shih; Phytochemistry (Elsevier); vol. 28; 7; (1989); p. 1883 - 1886. |
| F2 | *Cyperus rotundus* | M005490 | Hikino H, et al. Phytochemistry, 1976, 15 (8): 1265 |
| F2 | *Cyperus rotundus* | M005536 | Iwamura et al.; Nippon Kagaku Kaishi; (1977); p. 1018. |
| F2 | *Cyperus rotundus* | M006438 | Kapadia,V.H. et al.; Tetrahedron; vol. 21; (1965); p. 607 - 618. |
| F2 | *Cyperus rotundus* | M006445 | 江苏新医学院. 中药大辞典. 上海: 上海科学技术出版社, 1977 |
| F2 | *Cyperus rotundus* | M006507 | Retrieved from CNPD |
| F2 | *Cyperus rotundus* | M006884 | Jeong, Sei-Joon; Miyamoto, Tomofumi; Inagaki, Masanori; Kim, Youn-Chul; Higuchi, Ryuichi; Journal of Natural Products; vol. 63; 5; (2000); p. 673 - 675. |
| F2 | *Cyperus rotundus* | M007393 | Jeong, Sei-Joon; Miyamoto, Tomofumi; Inagaki, Masanori; Kim, Youn-Chul; Higuchi, Ryuichi; Journal of Natural Products; vol. 63; 5; (2000); p. 673 - 675. |
| F2 | *Cyperus rotundus* | M007516 | Kim, Su Jung; Ryu, Byeol; Kim, Ha-Yeong; Yang, Yeong-In; Ham, Jungyeob; Choi, Jung-Hye; Jang, Dae Sik; Bulletin of the Korean Chemical Society; vol. 34; 7; (2013); p. 2207 - 2210. |
| F2 | *Cyperus rotundus* | M008169 | Motl et al.; Chemistry and Industry (London, United Kingdom); (1963); p. 1284. |
| F2 | *Cyperus rotundus* | M008848 | Xu, Yan; Zhang, Hong-Wu; Yu, Chang-Yuan; Lu, Yang; Chang, Ying; Zou, Zhong-Mei; Molecules; vol. 13; 10; (2008); p. 2474 - 2481. |
| F2 | *Cyperus rotundus* | M008928 | Sonwa, Mesmin Mekem; Koenig, Wilfried A.; Phytochemistry; vol. 58; 5; (2001); p. 799 - 810. |
| F2 | *Cyperus rotundus* | M009001 | Ito, Tetsuro; Endo, Hidetatsu; Shinohara, Haruka; Oyama, Masayoshi; Akao, Yukihiro; Iinuma, Munekazu; Fitoterapia; vol. 83; 8; (2012); p. 1420 - 1429. |
| F2 | *Cyperus rotundus* | M009382 | 国家中医药管理局《中华本草》编委会. 中华本草. Vol. 1-30 上海:上海科学技术出版社, 1999 |
| F2 | *Cyperus rotundus* | M010415 | Zhou, Zhongliu; Yin, Wenqing; Molecules; vol. 17; 11; (2012); p. 12636 - 12641. |
| F2 | *Cyperus rotundus* | M010625 | Trivedi,B. et al.; Collection of Czechoslovak Chemical Communications; vol. 29; (1964); p. 1675 - 1688. |
| F2 | *Cyperus rotundus* | M010688 | Xu, Yan; Zhang, Hong-Wu; Yu, Chang-Yuan; Lu, Yang; Chang, Ying; Zou, Zhong-Mei; Molecules; vol. 13; 10; (2008); p. 2474 - 2481. |
| F2 | *Cyperus rotundus* | M012051 | Ohira, Susumu; Hasegawa, Taisuke; Hayashi, Ken-Ichiro; Hoshino, Takuji; Takaoka, Daisuke; Nozaki, Hiroshi; Phytochemistry; vol. 47; 8; (1998); p. 1577 - 1581. |
| F2 | *Cyperus rotundus* | M012236 | 国家中医药管理局《中华本草》编委会. 中华本草. Vol. 1-30 上海:上海科学技术出版社, 1999 |
| F2 | *Cyperus rotundus* | M012804 | Trivedi,B. et al.; Collection of Czechoslovak Chemical Communications; vol. 29; (1964); p. 1675 - 1688. |
| F2 | *Cyperus rotundus* | M013211 | Singh, P. N.; Singh, S. B.; Phytochemistry (Elsevier); vol. 19; (1980); p. 2056 - 2058. |
| F2 | *Cyperus rotundus* | M013440 | 江苏新医学院. 中药大辞典. 上海: 上海科学技术出版社, 1977 |
| F2 | *Cyperus rotundus* | M013753 | Sonwa, Mesmin Mekem; Koenig, Wilfried A.; Phytochemistry; vol. 58; 5; (2001); p. 799 - 810. |
| F2 | *Cyperus rotundus* | M014150 | Seo, Eun Ji; Lee, Dong-Ung; Kwak, Jong Hwan; Lee, Sun-Mee; Kim, Yeong Shik; Jung, Yi-Sook; Journal of Ethnopharmacology; vol. 135; 1; (2011); p. 48 - 54. |
| F2 | *Cyperus rotundus* | M014211 | Hikino; Aota; Takemoto; Chemical and pharmaceutical bulletin; vol. 13; 5; (1965); p. 628 - 630. |
| F2 | *Cyperus rotundus* | M014668 | Xu, Yan; Zhang, Hong-Wu; Wan, Xiao-Chun; Zou, Zhong-Mei; Magnetic Resonance in Chemistry; vol. 47; 6; (2009); p. 527 - 531. |
| F2 | *Cyperus rotundus* | M014883 | Hikino et al.; Chemical and Pharmaceutical Bulletin; vol. 14; (1966); p. 890,895. |
| F2 | *Cyperus rotundus* | M015719 | Hikino; Aota; Takemoto; Chemical and pharmaceutical bulletin; vol. 15; 9; (1967); p. 1433 - 1435.; Hikino,H. et al.; Chemical and Pharmaceutical Bulletin; vol. 16; (1968); p. 1900 - 1906. |
| F2 | *Cyperus rotundus* | M017895 | Ito, Tetsuro; Endo, Hidetatsu; Shinohara, Haruka; Oyama, Masayoshi; Akao, Yukihiro; Iinuma, Munekazu; Fitoterapia; vol. 83; 8; (2012); p. 1420 - 1429. |
| F2 | *Cyperus rotundus* | M017983 | Zhou, Zhongliu; Zhang, Hualin; Medicinal Chemistry Research; vol. 22; 10; (2013); p. 4830 - 4835. |
| F2 | *Cyperus rotundus* | M018094 | Seo, Eun Ji; Lee, Dong-Ung; Kwak, Jong Hwan; Lee, Sun-Mee; Kim, Yeong Shik; Jung, Yi-Sook; Journal of Ethnopharmacology; vol. 135; 1; (2011); p. 48 - 54. |
| F2 | *Cyperus rotundus* | M018224 | Zhou, Zhongliu; Yin, Wenqing; Molecules; vol. 17; 11; (2012); p. 12636 - 12641. |
| F2 | *Cyperus rotundus* | M018345 | Ito, Tetsuro; Endo, Hidetatsu; Shinohara, Haruka; Oyama, Masayoshi; Akao, Yukihiro; Iinuma, Munekazu; Fitoterapia; vol. 83; 8; (2012); p. 1420 - 1429. |
| F2 | *Cyperus rotundus* | M018428 | Zhou, Zhongliu; Zhang, Hualin; Medicinal Chemistry Research; vol. 22; 10; (2013); p. 4830 - 4835. |
| F2 | *Cyperus rotundus* | M018538 | Zhou, Zhongliu; Yin, Wenqing; Zhang, Hualin; Feng, Zongcai; Xia, Jingmin; Natural Product Research; vol. 27; 19; (2013); p. 1732 - 1736. |
| F2 | *Cyperus rotundus* | M018773 | Ito, Tetsuro; Endo, Hidetatsu; Shinohara, Haruka; Oyama, Masayoshi; Akao, Yukihiro; Iinuma, Munekazu; Fitoterapia; vol. 83; 8; (2012); p. 1420 - 1429. |
| F2 | *Cyperus rotundus* | M018873 | Zhou, Zhongliu; Zhang, Hualin; Medicinal Chemistry Research; vol. 22; 10; (2013); p. 4830 - 4835. |
| F2 | *Cyperus rotundus* | M018894 | Kim, Su Jung; Ryu, Byeol; Kim, Ha-Yeong; Yang, Yeong-In; Ham, Jungyeob; Choi, Jung-Hye; Jang, Dae Sik; Bulletin of the Korean Chemical Society; vol. 34; 7; (2013); p. 2207 - 2210. |
| F2 | *Cyperus rotundus* | M018975 | Zhou, Zhongliu; Yin, Wenqing; Zhang, Hualin; Feng, Zongcai; Xia, Jingmin; Natural Product Research; vol. 27; 19; (2013); p. 1732 - 1736. |
| F2 | *Cyperus rotundus* | M019082 | Zhou, Zhongliu; Yin, Wenqing; Molecules; vol. 17; 11; (2012); p. 12636 - 12641. |
| F2 | *Cyperus rotundus* | M019280 | Zhou, Zhongliu; Zhang, Hualin; Medicinal Chemistry Research; vol. 22; 10; (2013); p. 4830 - 4835. |
| F2 | *Cyperus rotundus* | M019305 | Kim, Su Jung; Ryu, Byeol; Kim, Ha-Yeong; Yang, Yeong-In; Ham, Jungyeob; Choi, Jung-Hye; Jang, Dae Sik; Bulletin of the Korean Chemical Society; vol. 34; 7; (2013); p. 2207 - 2210. |
| F2 | *Cyperus rotundus* | M019388 | Zhou, Zhongliu; Yin, Wenqing; Zhang, Hualin; Feng, Zongcai; Xia, Jingmin; Natural Product Research; vol. 27; 19; (2013); p. 1732 - 1736. |
| F2 | *Cyperus rotundus* | M019519 | Yang, Jun-Li; Shi, Yan-Ping; Planta Medica; vol. 78; 1; (2012); p. 59 - 64. |
| F2 | *Cyperus rotundus* | M019701 | Zhou, Zhongliu; Zhang, Hualin; Medicinal Chemistry Research; vol. 22; 10; (2013); p. 4830 - 4835. |
| F2 | *Cyperus rotundus* | M019720 | Kim, Su Jung; Ryu, Byeol; Kim, Ha-Yeong; Yang, Yeong-In; Ham, Jungyeob; Choi, Jung-Hye; Jang, Dae Sik; Bulletin of the Korean Chemical Society; vol. 34; 7; (2013); p. 2207 - 2210. |
| F2 | *Cyperus rotundus* | M019790 | Zhou, Zhongliu; Yin, Wenqing; Zhang, Hualin; Feng, Zongcai; Xia, Jingmin; Natural Product Research; vol. 27; 19; (2013); p. 1732 - 1736. |
| F2 | *Cyperus rotundus* | M019928 | Yang, Jun-Li; Shi, Yan-Ping; Planta Medica; vol. 78; 1; (2012); p. 59 - 64. |
| F2 | *Cyperus rotundus* | M020113 | Zhou, Zhongliu; Zhang, Hualin; Medicinal Chemistry Research; vol. 22; 10; (2013); p. 4830 - 4835. |
| F2 | *Cyperus rotundus* | M020331 | Yang, Jun-Li; Shi, Yan-Ping; Planta Medica; vol. 78; 1; (2012); p. 59 - 64. |
| F2 | *Cyperus rotundus* | M020738 | Yang, Jun-Li; Shi, Yan-Ping; Planta Medica; vol. 78; 1; (2012); p. 59 - 64. |
| F2 | *Cyperus rotundus* | M020801 | Kim, Su Jung; Kim, Hyun Ji; Kim, Hye Jin; Jang, Young Pyo; Oh, Myung Sook; Jang, Dae Sik; Bulletin of the Korean Chemical Society; vol. 33; 9; (2012); p. 3115 - 3118. |
| F2 | *Cyperus rotundus* | M020841 | Zhou, Zhongliu; Yin, Wenqing; Molecules; vol. 17; 11; (2012); p. 12636 - 12641. |
| F2 | *Cyperus rotundus* | M020865 | Zhou, Zhongliu; Fu, Chunyan; Chemistry of Natural Compounds; vol. 48; 6; (2013); p. 963 - 965. |
| F2 | *Cyperus rotundus* | M021145 | Kim, Su Jung; Kim, Hyun Ji; Kim, Hye Jin; Jang, Young Pyo; Oh, Myung Sook; Jang, Dae Sik; Bulletin of the Korean Chemical Society; vol. 33; 9; (2012); p. 3115 - 3118. |
| F2 | *Cyperus rotundus* | M021181 | Yang, Jun-Li; Shi, Yan-Ping; Planta Medica; vol. 78; 1; (2012); p. 59 - 64. |
| F2 | *Cyperus rotundus* | M021203 | Ito, Tetsuro; Endo, Hidetatsu; Oyama, Masayoshi; Iinuma, Munekazu; Phytochemistry Letters; vol. 5; 2; (2012); p. 267 - 270. |
| F2 | *Cyperus rotundus* | M021249 | Kim, Su Jung; Kim, Hyun Ji; Kim, Hye Jin; Jang, Young Pyo; Oh, Myung Sook; Jang, Dae Sik; Bulletin of the Korean Chemical Society; vol. 33; 9; (2012); p. 3115 - 3118. |
| F2 | *Cyperus rotundus* | M021290 | Zhou, Zhongliu; Yin, Wenqing; Molecules; vol. 17; 11; (2012); p. 12636 - 12641. |
| F2 | *Cyperus rotundus* | M021319 | Zhou, Zhongliu; Fu, Chunyan; Chemistry of Natural Compounds; vol. 48; 6; (2013); p. 963 - 965. |
| F2 | *Cyperus rotundus* | M021409 | Yang, Jun-Li; Shi, Yan-Ping; Planta Medica; vol. 78; 1; (2012); p. 59 - 64. |
| F2 | *Cyperus rotundus* | M021452 | Ohira, Susumu; Hasegawa, Taisuke; Hayashi, Ken-Ichiro; Hoshino, Takuji; Takaoka, Daisuke; Nozaki, Hiroshi; Phytochemistry; vol. 47; 8; (1998); p. 1577 - 1581. |
| F2 | *Cyperus rotundus* | M021686 | Kim, Su Jung; Kim, Hyun Ji; Kim, Hye Jin; Jang, Young Pyo; Oh, Myung Sook; Jang, Dae Sik; Bulletin of the Korean Chemical Society; vol. 33; 9; (2012); p. 3115 - 3118. |
| F2 | *Cyperus rotundus* | M021732 | Ito, Tetsuro; Endo, Hidetatsu; Shinohara, Haruka; Oyama, Masayoshi; Akao, Yukihiro; Iinuma, Munekazu; Fitoterapia; vol. 83; 8; (2012); p. 1420 - 1429. |
| F2 | *Cyperus rotundus* | M021870 | Kim, Su Jung; Ryu, Byeol; Kim, Ha-Yeong; Yang, Yeong-In; Ham, Jungyeob; Choi, Jung-Hye; Jang, Dae Sik; Bulletin of the Korean Chemical Society; vol. 34; 7; (2013); p. 2207 - 2210. |
| F2 | *Cyperus rotundus* | M022397 | 江苏新医学院. 中药大辞典. 上海: 上海科学技术出版社, 1977 |
| F2 | *Cyperus rotundus* | M022681 | Thebtaranonth; Wanauppathamkul; Yuthavong; Phytochemistry; vol. 40; 1; (1995); p. 125 - 128. |
| F2 | *Cyperus rotundus* | M022998 | Ha, Jeoung-Hee; Lee, Kwang-Youn; Choi, Hyoung-Chul; Cho, Jungsook; Kang, Byung-Soo; Lim, Jae-Chul; Lee, Dong-Ung; Biological and pharmaceutical bulletin; vol. 25; 1; (2002); p. 128 - 130. |
| F2 | *Cyperus rotundus* | M023007 | Xu, Yan; Zhang, Hong-Wu; Wan, Xiao-Chun; Zou, Zhong-Mei; Magnetic Resonance in Chemistry; vol. 47; 6; (2009); p. 527 - 531. |
| F2 | *Cyperus rotundus* | M023202 | Ohira, Susumu; Hasegawa, Taisuke; Hayashi, Ken-Ichiro; Hoshino, Takuji; Takaoka, Daisuke; Nozaki, Hiroshi; Phytochemistry; vol. 47; 8; (1998); p. 1577 - 1581. |
| F2 | *Cyperus rotundus* | M023320 | 江苏新医学院. 中药大辞典. 上海: 上海科学技术出版社, 1977 |
| F2 | *Cyperus rotundus* | M023445 | Paknikar et al.; Tetrahedron Letters; (1977); p. 2121. |
| F2 | *Cyperus rotundus* | M024105 | Xu, Yan; Zhang, Hong-Wu; Yu, Chang-Yuan; Lu, Yang; Chang, Ying; Zou, Zhong-Mei; Molecules; vol. 13; 10; (2008); p. 2474 - 2481. |
| F2 | *Cyperus rotundus* | M024465 | Seo, Eun Ji; Lee, Dong-Ung; Kwak, Jong Hwan; Lee, Sun-Mee; Kim, Yeong Shik; Jung, Yi-Sook; Journal of Ethnopharmacology; vol. 135; 1; (2011); p. 48 - 54. |
| F2 | *Cyperus rotundus* | M025284 | Hikino et al.; Tetrahedron Letters; (1969); p. 2741. |
| F2 | *Cyperus rotundus* | M025613 | Komai, Koichiro; Tang, Chung-Shih; Phytochemistry (Elsevier); vol. 28; 7; (1989); p. 1883 - 1886. |
| F2 | *Cyperus rotundus* | M026773 | Trivedi,B. et al.; Collection of Czechoslovak Chemical Communications; vol. 29; (1964); p. 1675 - 1688. |
| F2 | *Cyperus rotundus* | M026864 | Xu, Yan; Zhang, Hong-Wu; Yu, Chang-Yuan; Lu, Yang; Chang, Ying; Zou, Zhong-Mei; Molecules; vol. 13; 10; (2008); p. 2474 - 2481. |
| F2 | *Cyperus rotundus* | M027115 | Komai, Koichiro; Tang, Chung-Shih; Phytochemistry (Elsevier); vol. 28; 7; (1989); p. 1883 - 1886. |
| F2 | *Cyperus rotundus* | M027369 | Ohira, Susumu; Hasegawa, Taisuke; Hayashi, Ken-Ichiro; Hoshino, Takuji; Takaoka, Daisuke; Nozaki, Hiroshi; Phytochemistry; vol. 47; 8; (1998); p. 1577 - 1581. |
| F2 | *Cyperus rotundus* | M027547 | Xu, Yan; Zhang, Hong-Wu; Wan, Xiao-Chun; Zou, Zhong-Mei; Magnetic Resonance in Chemistry; vol. 47; 6; (2009); p. 527 - 531. |
| F2 | *Cyperus rotundus* | M027818 | Zhou, Zhongliu; Yin, Wenqing; Molecules; vol. 17; 11; (2012); p. 12636 - 12641. |
| F2 | *Cyperus rotundus* | M027992 | Retrieved from CNPD |
| F2 | *Cyperus rotundus* | M028031 | Zhou, Zhongliu; Yin, Wenqing; Molecules; vol. 17; 11; (2012); p. 12636 - 12641. |
| F2 | *Cyperus rotundus* | M028033 | Ito, Tetsuro; Endo, Hidetatsu; Shinohara, Haruka; Oyama, Masayoshi; Akao, Yukihiro; Iinuma, Munekazu; Fitoterapia; vol. 83; 8; (2012); p. 1420 - 1429. |
| F2 | *Cyperus rotundus* | M028521 | Seo, Eun Ji; Lee, Dong-Ung; Kwak, Jong Hwan; Lee, Sun-Mee; Kim, Yeong Shik; Jung, Yi-Sook; Journal of Ethnopharmacology; vol. 135; 1; (2011); p. 48 - 54. |
| F2 | *Cyperus rotundus* | M028553 | Hikino,H. et al.; Chemical and Pharmaceutical Bulletin; vol. 17; (1969); p. 1390 - 1394. |
| F2 | *Cyperus rotundus* | M029671 | Retrieved from CNPD |
| F2 | *Cyperus rotundus* | M029760 | Zhou, Zhongliu; Yin, Wenqing; Zhang, Hualin; Feng, Zongcai; Xia, Jingmin; Natural Product Research; vol. 27; 19; (2013); p. 1732 - 1736. |
| F2 | *Cyperus rotundus* | M029795 | Hikino et al.; Tetrahedron Letters; (1969); p. 2741. |
| F2 | *Cyperus rotundus* | M030002 | Sonwa, Mesmin Mekem; Koenig, Wilfried A.; Phytochemistry; vol. 58; 5; (2001); p. 799 - 810. |
| F2 | *Cyperus rotundus* | M030124 | Zhou, Zhongliu; Yin, Wenqing; Molecules; vol. 17; 11; (2012); p. 12636 - 12641. |
| F2 | *Cyperus rotundus* | M030347 | Kim, Su Jung; Ryu, Byeol; Kim, Ha-Yeong; Yang, Yeong-In; Ham, Jungyeob; Choi, Jung-Hye; Jang, Dae Sik; Bulletin of the Korean Chemical Society; vol. 34; 7; (2013); p. 2207 - 2210. |
| F2 | *Cyperus rotundus* | M030364 | Iwamura et al.; Nippon Kagaku Kaishi; (1977); p. 1018. |
| F2 | *Cyperus rotundus* | M031023 | Yang, Jun-Li; Shi, Yan-Ping; Planta Medica; vol. 78; 1; (2012); p. 59 - 64. |
| F2 | *Cyperus rotundus* | M031025 | Liu, Pei; Liu, Li; Tang, Yu Ping; Duan, Jin Ao; Yang, Nian Yun; Chinese Chemical Letters; vol. 21; 5; (2010); p. 606 - 609. |
| F2 | *Cyperus rotundus* | M031715 | Xu, Yan; Zhang, Hong-Wu; Wan, Xiao-Chun; Zou, Zhong-Mei; Magnetic Resonance in Chemistry; vol. 47; 6; (2009); p. 527 - 531. |
| F2 | *Cyperus rotundus* | M031839 | Hikino; Aota; Takemoto; Chemical and pharmaceutical bulletin; vol. 15; 12; (1967); p. 1929 - 1933. |
| F2 | *Cyperus rotundus* | M032883 | Bradfield et al.; Journal of the Chemical Society; (1936); p. 667,675. |
| F2 | *Cyperus rotundus* | M033239 | Stierle, Andrea; Upadhyay, Rajeev; Strobel, Gary; Phytochemistry (Elsevier); vol. 30; 7; (1991); p. 2191 - 2192. |
| F2 | *Eupolyphaga sinensis* | M003706 | Jiang, Hai-Long; Luo, Xiao-Hong; Wang, Xiao-Zheng; Yang, Jun-Li; Yao, Xiao-Jun; Wu, Quan-Xiang; Crews, Phillip; Valeriote, Frederick A.; Fitoterapia; vol. 83; 7; (2012); p. 1275 - 1280,6. |
| F2 | *Eupolyphaga sinensis* | M017864 | Jiang, Hai-Long; Luo, Xiao-Hong; Wang, Xiao-Zheng; Yang, Jun-Li; Yao, Xiao-Jun; Wu, Quan-Xiang; Crews, Phillip; Valeriote, Frederick A.; Fitoterapia; vol. 83; 7; (2012); p. 1275 - 1280,6. |
| F2 | *Eupolyphaga sinensis* | M018314 | Jiang, Hai-Long; Luo, Xiao-Hong; Wang, Xiao-Zheng; Yang, Jun-Li; Yao, Xiao-Jun; Wu, Quan-Xiang; Crews, Phillip; Valeriote, Frederick A.; Fitoterapia; vol. 83; 7; (2012); p. 1275 - 1280,6. |
| F2 | *Eupolyphaga sinensis* | M021698 | Jiang, Hai-Long; Luo, Xiao-Hong; Wang, Xiao-Zheng; Yang, Jun-Li; Yao, Xiao-Jun; Wu, Quan-Xiang; Crews, Phillip; Valeriote, Frederick A.; Fitoterapia; vol. 83; 7; (2012); p. 1275 - 1280,6. |
| F2 | *Eupolyphaga sinensis* | M026605 | Retrieved from CNPD |
| F2 | *Gentiana macrophylla* | M000003 | Tan; Wolfender; Zhang; Ma; Fuzzati; Marston; Hostettmann; Phytochemistry; vol. 42; 5; (1996); p. 1305 - 1313. |
| F2 | *Gentiana macrophylla* | M001133 | Jiang, Zhi-Bo; Liu, Hong-Li; Liu, Xiao-Qing; Shang, Ji-Ning; Zhao, Jia-Rui; Yuan, Cheng-Shan; Natural Product Research; vol. 24; 14; (2010); p. 1365 - 1369. |
| F2 | *Gentiana macrophylla* | M005817 | Jiang, Zhi-Bo; Liu, Hong-Li; Liu, Xiao-Qing; Shang, Ji-Ning; Zhao, Jia-Rui; Yuan, Cheng-Shan; Natural Product Research; vol. 24; 14; (2010); p. 1365 - 1369. |
| F2 | *Gentiana macrophylla* | M007551 | Tan; Wolfender; Zhang; Ma; Fuzzati; Marston; Hostettmann; Phytochemistry; vol. 42; 5; (1996); p. 1305 - 1313. |
| F2 | *Gentiana macrophylla* | M008091 | Tan; Wolfender; Zhang; Ma; Fuzzati; Marston; Hostettmann; Phytochemistry; vol. 42; 5; (1996); p. 1305 - 1313. |
| F2 | *Gentiana macrophylla* | M008255 | Tan; Wolfender; Zhang; Ma; Fuzzati; Marston; Hostettmann; Phytochemistry; vol. 42; 5; (1996); p. 1305 - 1313. |
| F2 | *Gentiana macrophylla* | M008350 | 陈千良等. 中草药, 2005, 35 (1): 4 |
| F2 | *Gentiana macrophylla* | M010015 | Tan; Wolfender; Zhang; Ma; Fuzzati; Marston; Hostettmann; Phytochemistry; vol. 42; 5; (1996); p. 1305 - 1313. |
| F2 | *Gentiana macrophylla* | M010232 | Jiang, Zhi-Bo; Liu, Hong-Li; Liu, Xiao-Qing; Shang, Ji-Ning; Zhao, Jia-Rui; Yuan, Cheng-Shan; Natural Product Research; vol. 24; 14; (2010); p. 1365 - 1369. |
| F2 | *Gentiana macrophylla* | M010296 | Tan; Wolfender; Zhang; Ma; Fuzzati; Marston; Hostettmann; Phytochemistry; vol. 42; 5; (1996); p. 1305 - 1313. |
| F2 | *Gentiana macrophylla* | M010381 | Kondo V, et al. 生药学杂志(日), 1993, 47 (3): 342 |
| F2 | *Gentiana macrophylla* | M011892 | Fu; Sun; Yaoxue Xuebao; vol. 6; (1958); p. 198,201; Chem.Abstr.; (1959); p. 8310. |
| F2 | *Gentiana macrophylla* | M012960 | (1) 阴健等. 中药现代研究与临床应用(1). 北京: 学苑出版社, 1993. (2) 国家中医药管理局《中华本草》编委会. 中华本草. Vol. 1-30 上海:上海科学技术出版社, 1999. |
| F2 | *Gentiana macrophylla* | M013252 | Tan; Wolfender; Zhang; Ma; Fuzzati; Marston; Hostettmann; Phytochemistry; vol. 42; 5; (1996); p. 1305 - 1313. |
| F2 | *Gentiana macrophylla* | M015166 | Tan; Wolfender; Zhang; Ma; Fuzzati; Marston; Hostettmann; Phytochemistry; vol. 42; 5; (1996); p. 1305 - 1313. |
| F2 | *Gentiana macrophylla* | M022304 | Tan; Wolfender; Zhang; Ma; Fuzzati; Marston; Hostettmann; Phytochemistry; vol. 42; 5; (1996); p. 1305 - 1313. |
| F2 | *Gentiana macrophylla* | M024758 | Tan; Wolfender; Zhang; Ma; Fuzzati; Marston; Hostettmann; Phytochemistry; vol. 42; 5; (1996); p. 1305 - 1313. |
| F2 | *Gentiana macrophylla* | M024900 | Tan; Wolfender; Zhang; Ma; Fuzzati; Marston; Hostettmann; Phytochemistry; vol. 42; 5; (1996); p. 1305 - 1313. |
| F2 | *Gentiana macrophylla* | M026153 | (1) 陈千良等. 中草药, 2005, 35 (1): 4. (2) 陈千良等. 中国中药杂志, 2005, 30 (19): 1519. |
| F2 | *Gentiana macrophylla* | M027113 | Tan; Wolfender; Zhang; Ma; Fuzzati; Marston; Hostettmann; Phytochemistry; vol. 42; 5; (1996); p. 1305 - 1313. |
| F2 | *Gentiana macrophylla* | M029037 | Tan; Wolfender; Zhang; Ma; Fuzzati; Marston; Hostettmann; Phytochemistry; vol. 42; 5; (1996); p. 1305 - 1313. |
| F2 | *Gentiana macrophylla* | M030585 | Jiang, Zhi-Bo; Liu, Hong-Li; Liu, Xiao-Qing; Shang, Ji-Ning; Zhao, Jia-Rui; Yuan, Cheng-Shan; Natural Product Research; vol. 24; 14; (2010); p. 1365 - 1369. |
| F2 | *Gentiana macrophylla* | M031000 | Jiang, Zhi-Bo; Liu, Hong-Li; Liu, Xiao-Qing; Shang, Ji-Ning; Zhao, Jia-Rui; Yuan, Cheng-Shan; Natural Product Research; vol. 24; 14; (2010); p. 1365 - 1369. |
| F2 | *Gentiana macrophylla* | M032651 | Tan; Wolfender; Zhang; Ma; Fuzzati; Marston; Hostettmann; Phytochemistry; vol. 42; 5; (1996); p. 1305 - 1313. |
| F2 | *Gleditsia sinensis* | M001351 | Zhang, Zhizhen; Koike, Kazuo; Jia, Zhonghua; Nikaido, Tamotsu; Guo, Dean; Zheng, Junhua; Journal of Natural Products; vol. 62; 6; (1999); p. 877 - 881. |
| F2 | *Gleditsia sinensis* | M001656 | (1) Zhong L, et al. Planta Med, 2004, 70 (9): 797. (2) Zhong L, et al. Planta Med, 2003, 69, 561. |
| F2 | *Gleditsia sinensis* | M002163 | Lim, Jae-Chul; Park, Jong Hee; Budesinsky, Milos; Kasal, Alexander; Han, Yeong-Hwan; Koo, Byung-Soo; Lee, Seung-Il; Lee, Dong-Ung; Chemical and Pharmaceutical Bulletin; vol. 53; 5; (2005); p. 561 - 564. |
| F2 | *Gleditsia sinensis* | M002365 | Zhong L, et al. Planta Med, 2004, 70 (9): 797 |
| F2 | *Gleditsia sinensis* | M005019 | LIM J-C, et al. Chem Pharm Bull, 2005, 53 (5): 561 |
| F2 | *Gleditsia sinensis* | M005809 | Zhong L, et al. Planta Med, 2004, 70 (9): 797 |
| F2 | *Gleditsia sinensis* | M006306 | Zhang, Zhizhen; Koike, Kazuo; Jia, Zhonghua; Nikaido, Tamotsu; Guo, Dean; Zheng, Junhua; Journal of Natural Products; vol. 62; 5; (1999); p. 740 - 745. |
| F2 | *Gleditsia sinensis* | M006463 | Zhang, Zhizhen; Koike, Kazuo; Jia, Zhonghua; Nikaido, Tamotsu; Guo, Dean; Zheng, Junhua; Journal of Natural Products; vol. 62; 5; (1999); p. 740 - 745. |
| F2 | *Gleditsia sinensis* | M006530 | Zhang, Zhizhen; Koike, Kazuo; Jia, Zhonghua; Nikaido, Tamotsu; Guo, Dean; Zheng, Junhua; Phytochemistry; vol. 52; 4; (1999); p. 715 - 722. |
| F2 | *Gleditsia sinensis* | M007168 | Zhang ZZ, et al. Phytochemistry, 1999, 52, 715 |
| F2 | *Gleditsia sinensis* | M008271 | Zhang, Zhizhen; Koike, Kazuo; Jia, Zhonghua; Nikaido, Tamotsu; Guo, Dean; Zheng, Junhua; Chemical and Pharmaceutical Bulletin; vol. 47; 3; (1999); p. 388 - 393. |
| F2 | *Gleditsia sinensis* | M008946 | Zhang, Zhizhen; Koike, Kazuo; Jia, Zhonghua; Nikaido, Tamotsu; Guo, Dean; Zheng, Junhua; Phytochemistry; vol. 52; 4; (1999); p. 715 - 722. |
| F2 | *Gleditsia sinensis* | M009167 | Zhong L, et al. Planta Med, 2004, 70 (9): 797 |
| F2 | *Gleditsia sinensis* | M009230 | Zhong L, et al. Planta Med, 2004, 70 (9): 797 |
| F2 | *Gleditsia sinensis* | M009905 | Zhang, Zhizhen; Koike, Kazuo; Jia, Zhonghua; Nikaido, Tamotsu; Guo, Dean; Zheng, Junhua; Phytochemistry; vol. 52; 4; (1999); p. 715 - 722. |
| F2 | *Gleditsia sinensis* | M010089 | Zhang ZZ, et al. Phytochemistry, 1999, 52, 715 |
| F2 | *Gleditsia sinensis* | M011325 | Zhang ZZ, et al. Phytochemistry, 1999, 52, 715 |
| F2 | *Gleditsia sinensis* | M012337 | Zhong L, et al. Planta Med, 2004, 70 (9): 797 |
| F2 | *Gleditsia sinensis* | M012513 | Zhong L, et al. Planta Med, 2004, 70 (9): 797 |
| F2 | *Gleditsia sinensis* | M013466 | Zhong L, et al. Planta Med, 2004, 70 (9): 797 |
| F2 | *Gleditsia sinensis* | M013603 | Zhang, Zhizhen; Koike, Kazuo; Jia, Zhonghua; Nikaido, Tamotsu; Guo, Dean; Zheng, Junhua; Chemical and Pharmaceutical Bulletin; vol. 47; 3; (1999); p. 388 - 393. |
| F2 | *Gleditsia sinensis* | M014784 | (1) 孙文基等. 天然活性成分简明手册. 北京: 中国医药科技出版社, 1998. (2) OTSUKA H, et al. Chem Pharm Bull, 2001, 49 (6): 699. (3) Hsieh PW, et al. JNP, 2004, 67 (7): 1175. |
| F2 | *Gleditsia sinensis* | M015212 | Zhang, Zhizhen; Koike, Kazuo; Jia, Zhonghua; Nikaido, Tamotsu; Guo, Dean; Zheng, Junhua; Phytochemistry; vol. 52; 4; (1999); p. 715 - 722. |
| F2 | *Gleditsia sinensis* | M015567 | Zhong L, et al. Planta Med, 2004, 70 (9): 797 |
| F2 | *Gleditsia sinensis* | M015659 | Zhang ZZ, et al. Phytochemistry, 1999, 52, 715 |
| F2 | *Gleditsia sinensis* | M015922 | Zhong L, et al. Planta Med, 2004, 70 (9): 797 |
| F2 | *Gleditsia sinensis* | M019437 | Wu, Jianming; Li, Jian; Zhu, Zhiyong; Li, Jiang; Huang, Guojun; Tang, Yao; Gao, Xiaoping; Fitoterapia; vol. 81; 1; (2010); p. 8 - 10. |
| F2 | *Gleditsia sinensis* | M022101 | Zhang, Zhizhen; Koike, Kazuo; Jia, Zhonghua; Nikaido, Tamotsu; Guo, Dean; Zheng, Junhua; Phytochemistry; vol. 52; 4; (1999); p. 715 - 722. |
| F2 | *Gleditsia sinensis* | M022587 | Zhang, Zhizhen; Koike, Kazuo; Jia, Zhonghua; Nikaido, Tamotsu; Guo, Dean; Zheng, Junhua; Chemical and Pharmaceutical Bulletin; vol. 47; 3; (1999); p. 388 - 393. |
| F2 | *Gleditsia sinensis* | M023754 | Zhang, Zhizhen; Koike, Kazuo; Jia, Zhonghua; Nikaido, Tamotsu; Guo, Dean; Zheng, Junhua; Journal of Natural Products; vol. 62; 6; (1999); p. 877 - 881. |
| F2 | *Gleditsia sinensis* | M024385 | Zhang, Zhizhen; Koike, Kazuo; Jia, Zhonghua; Nikaido, Tamotsu; Guo, Dean; Zheng, Junhua; Journal of Natural Products; vol. 62; 6; (1999); p. 877 - 881. |
| F2 | *Gleditsia sinensis* | M024719 | Zhong L, et al. Planta Med, 2004, 70 (9): 797 |
| F2 | *Gleditsia sinensis* | M025790 | Zhong L, et al. Planta Med, 2004, 70 (9): 797 |
| F2 | *Gleditsia sinensis* | M026254 | Zhang, Zhizhen; Koike, Kazuo; Jia, Zhonghua; Nikaido, Tamotsu; Guo, Dean; Zheng, Junhua; Journal of Natural Products; vol. 62; 5; (1999); p. 740 - 745. |
| F2 | *Gleditsia sinensis* | M028175 | (1) 滕荣伟等. 云南植物研究, 2002, 24 (4): 531. (2) Zhang ZZ, et al. Phytochemistry, 1999, 52, 715. (3) Zhong L, et al. Planta Med, 2004, 70 (9): 797. |
| F2 | *Gleditsia sinensis* | M028221 | Zhang, Zhizhen; Koike, Kazuo; Jia, Zhonghua; Nikaido, Tamotsu; Guo, Dean; Zheng, Junhua; Phytochemistry; vol. 52; 4; (1999); p. 715 - 722. |
| F2 | *Gleditsia sinensis* | M028468 | Zhang, Zhizhen; Koike, Kazuo; Jia, Zhonghua; Nikaido, Tamotsu; Guo, Dean; Zheng, Junhua; Journal of Natural Products; vol. 62; 6; (1999); p. 877 - 881. |
| F2 | *Gleditsia sinensis* | M029068 | Zhang, Zhizhen; Koike, Kazuo; Jia, Zhonghua; Nikaido, Tamotsu; Guo, Dean; Zheng, Junhua; Journal of Natural Products; vol. 62; 5; (1999); p. 740 - 745. |
| F2 | *Gleditsia sinensis* | M029426 | Lim, Jae-Chul; Park, Jong Hee; Budesinsky, Milos; Kasal, Alexander; Han, Yeong-Hwan; Koo, Byung-Soo; Lee, Seung-Il; Lee, Dong-Ung; Chemical and Pharmaceutical Bulletin; vol. 53; 5; (2005); p. 561 - 564. |
| F2 | *Gleditsia sinensis* | M031765 | Zhong, Lei; Qu, Guiqing; Li, Ping; Han, Jian; Guo, Dean; Planta medica; vol. 69; 6; (2003); p. 561 - 563. |
| F2 | *Gleditsia sinensis* | M032723 | Zhong L, et al. Planta Med, 2004, 70 (9): 797 |
| F2 | *Gleditsia sinensis* | M033236 | Zhang, Zhizhen; Koike, Kazuo; Jia, Zhonghua; Nikaido, Tamotsu; Guo, Dean; Zheng, Junhua; Chemical and Pharmaceutical Bulletin; vol. 47; 3; (1999); p. 388 - 393. |
| F2 | *Gleditsia sinensis* | M033315 | Zhang ZZ, et al. Phytochemistry, 1999, 52, 715 |
| F2 | *Gleditsia sinensis* | M033654 | Lim, Jae-Chul; Park, Jong Hee; Budesinsky, Milos; Kasal, Alexander; Han, Yeong-Hwan; Koo, Byung-Soo; Lee, Seung-Il; Lee, Dong-Ung; Chemical and Pharmaceutical Bulletin; vol. 53; 5; (2005); p. 561 - 564. |
| F2 | *Gleditsia sinensis* | M033725 | Zhang, Zhizhen; Koike, Kazuo; Jia, Zhonghua; Nikaido, Tamotsu; Guo, Dean; Zheng, Junhua; Chemical and Pharmaceutical Bulletin; vol. 47; 3; (1999); p. 388 - 393. |
| F2 | *Gleditsia sinensis* | M033757 | (1) 季宇彬等. 中药抗肿瘤有效成分药理与应用. 哈尔滨: 黑龙江科学技术出版社, 1995. (2) 孙文基等. 天然活性成分简明手册. 北京: 中国医药科技出版社, 1998. |
| F2 | *Momordica cochinchinensis* | M003338 | Vuong, Le Thuy; US2004/24275; A1; (2004). |
| F2 | *Momordica cochinchinensis* | M003668 | Kawamura, Noriaki; Watanabe, Hitoshi; Oshio, Haruji; Phytochemistry (Elsevier); vol. 27; 11; (1988); p. 3585 - 3592. |
| F2 | *Momordica cochinchinensis* | M003669 | Vuong, Le Thuy; US2004/24275; A1; (2004). |
| F2 | *Momordica cochinchinensis* | M004124 | Vuong, Le Thuy; US2004/24275; A1; (2004). |
| F2 | *Momordica cochinchinensis* | M004842 | Kuwada; Yoshiki; Yakugaku Zasshi; vol. 57; (1937); p. 695,707; dtsch. Ref. S. 155; Yakugaku Zasshi; vol. 60; (1940); p. 85,90; dtsch. Ref. S. 25; Chem.Abstr.; (1937); p. 8542,1940 5088. |
| F2 | *Momordica cochinchinensis* | M004987 | Kawamura, Noriaki; Watanabe, Hitoshi; Oshio, Haruji; Phytochemistry (Elsevier); vol. 27; 11; (1988); p. 3585 - 3592. |
| F2 | *Momordica cochinchinensis* | M005186 | Hopkins et al.; Lipids; vol. 4; (1969); p. 89. |
| F2 | *Momordica cochinchinensis* | M007317 | Vuong, Le Thuy; US2004/24275; A1; (2004). |
| F2 | *Momordica cochinchinensis* | M007948 | Kawamura, Noriaki; Watanabe, Hitoshi; Oshio, Haruji; Phytochemistry (Elsevier); vol. 27; 11; (1988); p. 3585 - 3592. |
| F2 | *Momordica cochinchinensis* | M008463 | Murakami et al.; Tetrahedron Letters; (1966); p. 5137. |
| F2 | *Momordica cochinchinensis* | M010231 | Vuong, Le Thuy; US2004/24275; A1; (2004). |
| F2 | *Momordica cochinchinensis* | M010816 | Kawamura, Noriaki; Watanabe, Hitoshi; Oshio, Haruji; Phytochemistry (Elsevier); vol. 27; 11; (1988); p. 3585 - 3592. |
| F2 | *Momordica cochinchinensis* | M012261 | 江苏新医学院. 中药大辞典. 上海: 上海科学技术出版社, 1977 |
| F2 | *Momordica cochinchinensis* | M012419 | Iwamoto; Okabe; yamauchi; Chemical and Pharmaceutical Bulletin; vol. 33; 1; (1985); p. 1 - 7. |
| F2 | *Momordica cochinchinensis* | M012713 | Vuong, Le Thuy; US2004/24275; A1; (2004). |
| F2 | *Momordica cochinchinensis* | M013325 | Kawamura, Noriaki; Watanabe, Hitoshi; Oshio, Haruji; Phytochemistry (Elsevier); vol. 27; 11; (1988); p. 3585 - 3592. |
| F2 | *Momordica cochinchinensis* | M013387 | Iwamoto; Okabe; yamauchi; Chemical and Pharmaceutical Bulletin; vol. 33; 1; (1985); p. 1 - 7. |
| F2 | *Momordica cochinchinensis* | M014281 | Kawamura, Noriaki; Watanabe, Hitoshi; Oshio, Haruji; Phytochemistry (Elsevier); vol. 27; 11; (1988); p. 3585 - 3592. |
| F2 | *Momordica cochinchinensis* | M016715 | Kawamura, Noriaki; Watanabe, Hitoshi; Oshio, Haruji; Phytochemistry (Elsevier); vol. 27; 11; (1988); p. 3585 - 3592. |
| F2 | *Momordica cochinchinensis* | M016866 | Kawamura, Noriaki; Watanabe, Hitoshi; Oshio, Haruji; Phytochemistry (Elsevier); vol. 27; 11; (1988); p. 3585 - 3592. |
| F2 | *Momordica cochinchinensis* | M021875 | Iwamoto; Okabe; yamauchi; Chemical and Pharmaceutical Bulletin; vol. 33; 1; (1985); p. 1 - 7. |
| F2 | *Momordica cochinchinensis* | M025957 | Kawamura, Noriaki; Watanabe, Hitoshi; Oshio, Haruji; Phytochemistry (Elsevier); vol. 27; 11; (1988); p. 3585 - 3592. |
| F2 | *Momordica cochinchinensis* | M026193 | Vuong, Le Thuy; US2004/24275; A1; (2004). |
| F2 | *Momordica cochinchinensis* | M029628 | Vuong, Le Thuy; US2004/24275; A1; (2004). |
| F2 | *Momordica cochinchinensis* | M031458 | (1) 孙文基等. 天然活性成分简明手册. 北京: 中国医药科技出版社, 1998. (2) 赵维民等. 天然产物研究与开发, 1999, 11 (1): 1. (3) Buckingham J(Executive Editor): et al. Dictionary of Natural Products, Vol 1-7, Chapman & Hall, London, 1994; 1995, Vol 8; 1996, Vol 9; 1997, Vol 10; 1998, Vol 11.. (4) YOSHIKAWA M, et al. Chem Pharm Bull, 2001, 49 (7): 863. |
| F2 | *Momordica cochinchinensis* | M034000 | Kawamura, Noriaki; Watanabe, Hitoshi; Oshio, Haruji; Phytochemistry (Elsevier); vol. 27; 11; (1988); p. 3585 - 3592. |
| F2 | *Momordica cochinchinensis* | M034054 | 江苏新医学院. 中药大辞典. 上海: 上海科学技术出版社, 1977 |
| F2 | *Phellodendron amurense* | M000046 | Wu TS, et al. JNP, 2003, 66 (9): 1207 |
| F2 | *Phellodendron amurense* | M000059 | Honda, Keiichi; Hayashi, Nanao; Journal of Chemical Ecology; vol. 21; 10; (1995); p. 1531 - 1540. |
| F2 | *Phellodendron amurense* | M000204 | Leu, Chien-Hsing; Li, Chia-Ying; Yao, Xinsheng; Wu, Tian-Shung; Chemical and Pharmaceutical Bulletin; vol. 54; 9; (2006); p. 1308 - 1311. |
| F2 | *Phellodendron amurense* | M000262 | Ida, Yoshiteru; Satih, Yohko; Ohtsuka, Masumi; Nagasao, Miki; Shoji, Junzo; Phytochemistry (Elsevier); vol. 35; 1; (1994); p. 209 - 216. |
| F2 | *Phellodendron amurense* | M001194 | Wu, Tian-Shung; Hsu, Meei-Yu; Kuo, Ping-Chung; Sreenivasulu; Damu; Su, Chung-Ren; Li, Chia-Ying; Chang, Hsien-Chang; Journal of Natural Products; vol. 66; 9; (2003); p. 1207 - 1211. |
| F2 | *Phellodendron amurense* | M001667 | Leu, Chien-Hsing; Li, Chia-Ying; Yao, Xinsheng; Wu, Tian-Shung; Chemical and Pharmaceutical Bulletin; vol. 54; 9; (2006); p. 1308 - 1311. |
| F2 | *Phellodendron amurense* | M002056 | Ida, Yoshiteru; Satih, Yohko; Ohtsuka, Masumi; Nagasao, Miki; Shoji, Junzo; Phytochemistry (Elsevier); vol. 35; 1; (1994); p. 209 - 216. |
| F2 | *Phellodendron amurense* | M002318 | Ida, Yoshiteru; Satih, Yohko; Ohtsuka, Masumi; Nagasao, Miki; Shoji, Junzo; Phytochemistry (Elsevier); vol. 35; 1; (1994); p. 209 - 216. |
| F2 | *Phellodendron amurense* | M002468 | Ida, Yoshiteru; Satih, Yohko; Ohtsuka, Masumi; Nagasao, Miki; Shoji, Junzo; Phytochemistry (Elsevier); vol. 35; 1; (1994); p. 209 - 216. |
| F2 | *Phellodendron amurense* | M003050 | Ikuta, Akira; Nakamura, Takayuki; Urabe, Hisao; Phytochemistry; vol. 48; 2; (1998); p. 285 - 291. |
| F2 | *Phellodendron amurense* | M003269 | Leu, Chien-Hsing; Li, Chia-Ying; Yao, Xinsheng; Wu, Tian-Shung; Chemical and Pharmaceutical Bulletin; vol. 54; 9; (2006); p. 1308 - 1311. |
| F2 | *Phellodendron amurense* | M004428 | Leu, Chien-Hsing; Li, Chia-Ying; Yao, Xinsheng; Wu, Tian-Shung; Chemical and Pharmaceutical Bulletin; vol. 54; 9; (2006); p. 1308 - 1311. |
| F2 | *Phellodendron amurense* | M004519 | Wu, Tian-Shung; Hsu, Meei-Yu; Kuo, Ping-Chung; Sreenivasulu; Damu; Su, Chung-Ren; Li, Chia-Ying; Chang, Hsien-Chang; Journal of Natural Products; vol. 66; 9; (2003); p. 1207 - 1211. |
| F2 | *Phellodendron amurense* | M004565 | Ikuta, Akira; Urabe, Hisao; Nakamura, Takayuki; Journal of Natural Products; vol. 61; 8; (1998); p. 1012 - 1014. |
| F2 | *Phellodendron amurense* | M004705 | Nishioka; Yakugaku Zasshi; vol. 78; (1958); p. 1432; Chem.Abstr.; (1959); p. 8198. |
| F2 | *Phellodendron amurense* | M004742 | Retrieved from CNPD |
| F2 | *Phellodendron amurense* | M005285 | Wu, Tian-Shung; Hsu, Meei-Yu; Kuo, Ping-Chung; Sreenivasulu; Damu; Su, Chung-Ren; Li, Chia-Ying; Chang, Hsien-Chang; Journal of Natural Products; vol. 66; 9; (2003); p. 1207 - 1211. |
| F2 | *Phellodendron amurense* | M005404 | Wu, Tian-Shung; Hsu, Meei-Yu; Kuo, Ping-Chung; Sreenivasulu; Damu; Su, Chung-Ren; Li, Chia-Ying; Chang, Hsien-Chang; Journal of Natural Products; vol. 66; 9; (2003); p. 1207 - 1211. |
| F2 | *Phellodendron amurense* | M005424 | 江苏新医学院. 中药大辞典. 上海: 上海科学技术出版社, 1977 |
| F2 | *Phellodendron amurense* | M005869 | Leu, Chien-Hsing; Li, Chia-Ying; Yao, Xinsheng; Wu, Tian-Shung; Chemical and Pharmaceutical Bulletin; vol. 54; 9; (2006); p. 1308 - 1311. |
| F2 | *Phellodendron amurense* | M006012 | Wu TS, et al. JNP, 2003, 66 (9): 1207 |
| F2 | *Phellodendron amurense* | M006064 | Kawaguchi; Kim; Ishida; Ahn; Yamamoto; Yamaoka; Kozuka; Goto; Takahashi; Agricultural and Biological Chemistry; vol. 53; 10; (1989); p. 2635 - 2640. |
| F2 | *Phellodendron amurense* | M006104 | Leu, Chien-Hsing; Li, Chia-Ying; Yao, Xinsheng; Wu, Tian-Shung; Chemical and Pharmaceutical Bulletin; vol. 54; 9; (2006); p. 1308 - 1311. |
| F2 | *Phellodendron amurense* | M006253 | (1) 阴健等. 中药现代研究与临床应用(1). 北京: 学苑出版社, 1993. (2) 国家中医药管理局《中华本草》编委会. 中华本草. Vol. 1-30 上海:上海科学技术出版社, 1999. |
| F2 | *Phellodendron amurense* | M006416 | Kishi, Kuki; Yoshikawa, Kazuko; Arihara, Shigenobu; Phytochemistry (Elsevier); vol. 31; 4; (1992); p. 1335 - 1338. |
| F2 | *Phellodendron amurense* | M006730 | Wu, Tian-Shung; Hsu, Meei-Yu; Kuo, Ping-Chung; Sreenivasulu; Damu; Su, Chung-Ren; Li, Chia-Ying; Chang, Hsien-Chang; Journal of Natural Products; vol. 66; 9; (2003); p. 1207 - 1211. |
| F2 | *Phellodendron amurense* | M007237 | Wu, Tian-Shung; Hsu, Meei-Yu; Kuo, Ping-Chung; Sreenivasulu; Damu; Su, Chung-Ren; Li, Chia-Ying; Chang, Hsien-Chang; Journal of Natural Products; vol. 66; 9; (2003); p. 1207 - 1211. |
| F2 | *Phellodendron amurense* | M007461 | Wu TS, et al. JNP, 2003, 66 (9): 1207 |
| F2 | *Phellodendron amurense* | M007663 | (1) Gray AI, et al. Phytochemistry, 1988, 27 (6): 1805. (2) Su R, et al. Chem Pharm Bull, 1990, 38, 1616. (3) Itokawa H, et al. Chem Pharm Bull, 1992, 40, 1053. (4) Mulholland DA, et al. Phytochemistry, 1988, 27 (4): 1220. (5) Kishi K, et al. Phytochemistry, 1992, 31 (4): 1335. (6) Benosman A, et al. Phytochemistry, 1995, 40 (5): 1485. |
| F2 | *Phellodendron amurense* | M007888 | Kishi, Kuki; Yoshikawa, Kazuko; Arihara, Shigenobu; Phytochemistry (Elsevier); vol. 31; 4; (1992); p. 1335 - 1338. |
| F2 | *Phellodendron amurense* | M008302 | Wu, Tian-Shung; Hsu, Meei-Yu; Kuo, Ping-Chung; Sreenivasulu; Damu; Su, Chung-Ren; Li, Chia-Ying; Chang, Hsien-Chang; Journal of Natural Products; vol. 66; 9; (2003); p. 1207 - 1211. |
| F2 | *Phellodendron amurense* | M008307 | Wu, Tian-Shung; Hsu, Meei-Yu; Kuo, Ping-Chung; Sreenivasulu; Damu; Su, Chung-Ren; Li, Chia-Ying; Chang, Hsien-Chang; Journal of Natural Products; vol. 66; 9; (2003); p. 1207 - 1211. |
| F2 | *Phellodendron amurense* | M008381 | Leu, Chien-Hsing; Li, Chia-Ying; Yao, Xinsheng; Wu, Tian-Shung; Chemical and Pharmaceutical Bulletin; vol. 54; 9; (2006); p. 1308 - 1311. |
| F2 | *Phellodendron amurense* | M008402 | Ida, Yoshiteru; Satih, Yohko; Ohtsuka, Masumi; Nagasao, Miki; Shoji, Junzo; Phytochemistry (Elsevier); vol. 35; 1; (1994); p. 209 - 216. |
| F2 | *Phellodendron amurense* | M008514 | Bodaiski T, et al. Chem. Abstr., 1969, 71, 70452 |
| F2 | *Phellodendron amurense* | M008545 | Murayama; Shinozaki; Yakugaku Zasshi; (1926); p. 32; Chem. Zentralbl.; vol. 97; II; (1926); p. 2731. |
| F2 | *Phellodendron amurense* | M008557 | Leu, Chien-Hsing; Li, Chia-Ying; Yao, Xinsheng; Wu, Tian-Shung; Chemical and Pharmaceutical Bulletin; vol. 54; 9; (2006); p. 1308 - 1311. |
| F2 | *Phellodendron amurense* | M009052 | Leu, Chien-Hsing; Li, Chia-Ying; Yao, Xinsheng; Wu, Tian-Shung; Chemical and Pharmaceutical Bulletin; vol. 54; 9; (2006); p. 1308 - 1311. |
| F2 | *Phellodendron amurense* | M009732 | Hasegawa; Shirato; Journal of the American Chemical Society; vol. 75; (1953); p. 5507,5510. |
| F2 | *Phellodendron amurense* | M009996 | Chiang, Yu-Lung; Su, Chung-Ren; Kuo, Ping-Chung; Damu, Amooru G.; Wu, Tian-Shung; Heterocycles; vol. 68; 2; (2006); p. 339 - 345. |
| F2 | *Phellodendron amurense* | M010824 | Wu, Tian-Shung; Hsu, Meei-Yu; Kuo, Ping-Chung; Sreenivasulu; Damu; Su, Chung-Ren; Li, Chia-Ying; Chang, Hsien-Chang; Journal of Natural Products; vol. 66; 9; (2003); p. 1207 - 1211. |
| F2 | *Phellodendron amurense* | M011080 | (1) 阴健等. 中药现代研究与临床应用(1). 北京: 学苑出版社, 1993. (2) 廖静等. 中国中药杂志, 1994, 19 (10): 612. (3) 孙文基等. 天然活性成分简明手册. 北京: 中国医药科技出版社, 1998. |
| F2 | *Phellodendron amurense* | M011173 | Wu, Tian-Shung; Hsu, Meei-Yu; Kuo, Ping-Chung; Sreenivasulu; Damu; Su, Chung-Ren; Li, Chia-Ying; Chang, Hsien-Chang; Journal of Natural Products; vol. 66; 9; (2003); p. 1207 - 1211. |
| F2 | *Phellodendron amurense* | M011795 | Wu, Tian-Shung; Hsu, Meei-Yu; Kuo, Ping-Chung; Sreenivasulu; Damu; Su, Chung-Ren; Li, Chia-Ying; Chang, Hsien-Chang; Journal of Natural Products; vol. 66; 9; (2003); p. 1207 - 1211. |
| F2 | *Phellodendron amurense* | M011859 | Kunitomo; Yakugaku Zasshi; vol. 81; (1961); p. 1370; Chem.Abstr.; vol. 56; 8840; (1962). |
| F2 | *Phellodendron amurense* | M012020 | Ikuta, Akira; Nakamura, Takayuki; Urabe, Hisao; Phytochemistry; vol. 48; 2; (1998); p. 285 - 291. |
| F2 | *Phellodendron amurense* | M012113 | Shevchuk OI, et al. Khim Prir Soed, 1968, 4, 77; Chem Nat Compd (Engl Transl), 66 |
| F2 | *Phellodendron amurense* | M012308 | (1) 汪纪武等. 植物药有效成分手册. 北京: 人民卫生出版社, 1986. (2) 阴健等. 中药现代研究与临床应用(1). 北京: 学苑出版社, 1993. |
| F2 | *Phellodendron amurense* | M012389 | Kawaguchi; Kim; Ishida; Ahn; Yamamoto; Yamaoka; Kozuka; Goto; Takahashi; Agricultural and Biological Chemistry; vol. 53; 10; (1989); p. 2635 - 2640. |
| F2 | *Phellodendron amurense* | M012565 | Wu, Tian-Shung; Hsu, Meei-Yu; Kuo, Ping-Chung; Sreenivasulu; Damu; Su, Chung-Ren; Li, Chia-Ying; Chang, Hsien-Chang; Journal of Natural Products; vol. 66; 9; (2003); p. 1207 - 1211. |
| F2 | *Phellodendron amurense* | M012639 | Wu, Tian-Shung; Hsu, Meei-Yu; Kuo, Ping-Chung; Sreenivasulu; Damu; Su, Chung-Ren; Li, Chia-Ying; Chang, Hsien-Chang; Journal of Natural Products; vol. 66; 9; (2003); p. 1207 - 1211. |
| F2 | *Phellodendron amurense* | M013011 | Chiang, Yu-Lung; Su, Chung-Ren; Kuo, Ping-Chung; Damu, Amooru G.; Wu, Tian-Shung; Heterocycles; vol. 68; 2; (2006); p. 339 - 345. |
| F2 | *Phellodendron amurense* | M013074 | Tomita; Kunitomo; Yakugaku Zasshi; vol. 80; (1960); p. 880,884; Chem.Abstr.; 24828; (1960). |
| F2 | *Phellodendron amurense* | M013088 | Wu TS, et al. JNP, 2003, 66 (9): 1207 |
| F2 | *Phellodendron amurense* | M013200 | Ida, Yoshiteru; Satih, Yohko; Ohtsuka, Masumi; Nagasao, Miki; Shoji, Junzo; Phytochemistry (Elsevier); vol. 35; 1; (1994); p. 209 - 216. |
| F2 | *Phellodendron amurense* | M015149 | Ikuta, Akira; Nakamura, Takayuki; Urabe, Hisao; Phytochemistry; vol. 48; 2; (1998); p. 285 - 291. |
| F2 | *Phellodendron amurense* | M015294 | 汪纪武等. 植物药有效成分手册. 北京: 人民卫生出版社, 1986 |
| F2 | *Phellodendron amurense* | M015379 | Retrieved from CNPD |
| F2 | *Phellodendron amurense* | M015448 | Ida, Yoshiteru; Satih, Yohko; Ohtsuka, Masumi; Nagasao, Miki; Shoji, Junzo; Phytochemistry (Elsevier); vol. 35; 1; (1994); p. 209 - 216. |
| F2 | *Phellodendron amurense* | M015525 | Kawaguchi; Kim; Ishida; Ahn; Yamamoto; Yamaoka; Kozuka; Goto; Takahashi; Agricultural and Biological Chemistry; vol. 53; 10; (1989); p. 2635 - 2640. |
| F2 | *Phellodendron amurense* | M015617 | Wu, Tian-Shung; Hsu, Meei-Yu; Kuo, Ping-Chung; Sreenivasulu; Damu; Su, Chung-Ren; Li, Chia-Ying; Chang, Hsien-Chang; Journal of Natural Products; vol. 66; 9; (2003); p. 1207 - 1211. |
| F2 | *Phellodendron amurense* | M015808 | Ikuta, Akira; Urabe, Hisao; Nakamura, Takayuki; Journal of Natural Products; vol. 61; 8; (1998); p. 1012 - 1014. |
| F2 | *Phellodendron amurense* | M018210 | Wu, Tian-Shung; Hsu, Meei-Yu; Kuo, Ping-Chung; Sreenivasulu; Damu; Su, Chung-Ren; Li, Chia-Ying; Chang, Hsien-Chang; Journal of Natural Products; vol. 66; 9; (2003); p. 1207 - 1211. |
| F2 | *Phellodendron amurense* | M019033 | Leu, Chien-Hsing; Li, Chia-Ying; Yao, Xinsheng; Wu, Tian-Shung; Chemical and Pharmaceutical Bulletin; vol. 54; 9; (2006); p. 1308 - 1311. |
| F2 | *Phellodendron amurense* | M019495 | Ida, Yoshiteru; Satih, Yohko; Ohtsuka, Masumi; Nagasao, Miki; Shoji, Junzo; Phytochemistry (Elsevier); vol. 35; 1; (1994); p. 209 - 216. |
| F2 | *Phellodendron amurense* | M019869 | Leu, Chien-Hsing; Li, Chia-Ying; Yao, Xinsheng; Wu, Tian-Shung; Chemical and Pharmaceutical Bulletin; vol. 54; 9; (2006); p. 1308 - 1311. |
| F2 | *Phellodendron amurense* | M020293 | Kunitomo; Yakugaku Zasshi; vol. 82; (1962); p. 611; Chem.Abstr.; vol. 57; 4760; (1962). |
| F2 | *Phellodendron amurense* | M021078 | Wu, Tian-Shung; Hsu, Meei-Yu; Kuo, Ping-Chung; Sreenivasulu; Damu; Su, Chung-Ren; Li, Chia-Ying; Chang, Hsien-Chang; Journal of Natural Products; vol. 66; 9; (2003); p. 1207 - 1211. |
| F2 | *Phellodendron amurense* | M021551 | Ida, Yoshiteru; Satih, Yohko; Ohtsuka, Masumi; Nagasao, Miki; Shoji, Junzo; Phytochemistry (Elsevier); vol. 35; 1; (1994); p. 209 - 216. |
| F2 | *Phellodendron amurense* | M021600 | Wu, Tian-Shung; Hsu, Meei-Yu; Kuo, Ping-Chung; Sreenivasulu; Damu; Su, Chung-Ren; Li, Chia-Ying; Chang, Hsien-Chang; Journal of Natural Products; vol. 66; 9; (2003); p. 1207 - 1211. |
| F2 | *Phellodendron amurense* | M021962 | Ida, Yoshiteru; Satih, Yohko; Ohtsuka, Masumi; Nagasao, Miki; Shoji, Junzo; Phytochemistry (Elsevier); vol. 35; 1; (1994); p. 209 - 216. |
| F2 | *Phellodendron amurense* | M022467 | (1) 屠鹏飞等. 药学学报, 1999, 34 (1): 39. (2) Wu TS, et al. Phytochemistry, 1996, 43 (1): 133. (3) Wu PL, et al. Chem Pharm Bull, 2005, 53 (1): 56. (4) Ali MS, et al. JNP, 2001, 64 (3): 289. (5) Patnam R, et al. JNP, 2001, 64 (7): 948. (6) CHIU C-Y, et al. Chem Pharm Bull, 2005, 53 (9): 1118. (7) Pan WB, et al. JNP, 2003, 66 (1): 161. (8) Wu TS, et al. JNP, 2003, 66 (9): 1207. (9) McNally DJ, et al. JNP, 2003, 66 (9): 1280. (10) Chen J-J, et al. Planta Med, 2003, 69, 542. |
| F2 | *Phellodendron amurense* | M022757 | Nelson; Journal of the American Chemical Society; vol. 60; (1938); p. 920. |
| F2 | *Phellodendron amurense* | M022823 | (1) 孙文基等. 天然活性成分简明手册. 北京: 中国医药科技出版社, 1998. (2) Wu PL, et al. Chem Pharm Bull, 2005, 53 (1): 56. (3) CHIU C-Y, et al. Chem Pharm Bull, 2005, 53 (9): 1118. (4) Wu TS, et al. JNP, 2003, 66 (9): 1207. |
| F2 | *Phellodendron amurense* | M022879 | Wu, Tian-Shung; Hsu, Meei-Yu; Kuo, Ping-Chung; Sreenivasulu; Damu; Su, Chung-Ren; Li, Chia-Ying; Chang, Hsien-Chang; Journal of Natural Products; vol. 66; 9; (2003); p. 1207 - 1211. |
| F2 | *Phellodendron amurense* | M023492 | Kawaguchi; Kim; Ishida; Ahn; Yamamoto; Yamaoka; Kozuka; Goto; Takahashi; Agricultural and Biological Chemistry; vol. 53; 10; (1989); p. 2635 - 2640. |
| F2 | *Phellodendron amurense* | M023861 | Wada; Yagi; Matsumura; Sasaki; Sakata; Haga; Chemical and Pharmaceutical Bulletin; vol. 38; 8; (1990); p. 2332 - 2334. |
| F2 | *Phellodendron amurense* | M024013 | Ikuta, Akira; Nakamura, Takayuki; Urabe, Hisao; Phytochemistry; vol. 48; 2; (1998); p. 285 - 291. |
| F2 | *Phellodendron amurense* | M024023 | Leu, Chien-Hsing; Li, Chia-Ying; Yao, Xinsheng; Wu, Tian-Shung; Chemical and Pharmaceutical Bulletin; vol. 54; 9; (2006); p. 1308 - 1311. |
| F2 | *Phellodendron amurense* | M024634 | 江苏新医学院. 中药大辞典. 上海: 上海科学技术出版社, 1977 |
| F2 | *Phellodendron amurense* | M024671 | (1) Buckingham J(Executive Editor): et al. Dictionary of Natural Products, Vol 1-7, Chapman & Hall, London, 1994; 1995, Vol 8; 1996, Vol 9; 1997, Vol 10; 1998, Vol 11.. (2) 杜程芳等. 中国中药杂志, 2005, 30 (21): 1663. |
| F2 | *Phellodendron amurense* | M024989 | Ikuta, Akira; Nakamura, Takayuki; Urabe, Hisao; Phytochemistry; vol. 48; 2; (1998); p. 285 - 291. |
| F2 | *Phellodendron amurense* | M025553 | (1) 阴健等. 中药现代研究与临床应用(1). 北京: 学苑出版社, 1993. (2) 季宇彬等. 中药抗肿瘤有效成分药理与应用. 哈尔滨: 黑龙江科学技术出版社, 1995. (3) 阎玉凝等. 中国中药杂志, 1993, 18 (10): 615. (4) 孙文�� 天然活性成分简明手册. 北京: 中国医药科技出版社, 1998. (5) 郭幼莹等. 药学学报, 1999, 34 (9): 690. (6) 欧明等. 简明中药成分手册. 北京: 中国医药科技出版社, 2003. |
| F2 | *Phellodendron amurense* | M025639 | (1) Wu PL, et al. Chem Pharm Bull, 2005, 53 (1): 56. (2) Wu TS, et al. JNP, 2001, 64 (8): 1040. (3) LEU Y-L, et al. Chem Pharm Bull, 2005, 53 (7): 853. (4) Wu TS, et al. JNP, 2003, 66 (9): 1207. |
| F2 | *Phellodendron amurense* | M026113 | Bodalski; Lamer; Acta Poloniae Pharmaceutica; vol. 22; (1965); p. 302,304. |
| F2 | *Phellodendron amurense* | M026422 | Wu, Tian-Shung; Hsu, Meei-Yu; Kuo, Ping-Chung; Sreenivasulu; Damu; Su, Chung-Ren; Li, Chia-Ying; Chang, Hsien-Chang; Journal of Natural Products; vol. 66; 9; (2003); p. 1207 - 1211. |
| F2 | *Phellodendron amurense* | M026614 | Wu TS, et al. JNP, 2003, 66 (9): 1207 |
| F2 | *Phellodendron amurense* | M027425 | Wu, Tian-Shung; Hsu, Meei-Yu; Kuo, Ping-Chung; Sreenivasulu; Damu; Su, Chung-Ren; Li, Chia-Ying; Chang, Hsien-Chang; Journal of Natural Products; vol. 66; 9; (2003); p. 1207 - 1211. |
| F2 | *Phellodendron amurense* | M027822 | Wu, Tian-Shung; Hsu, Meei-Yu; Kuo, Ping-Chung; Sreenivasulu; Damu; Su, Chung-Ren; Li, Chia-Ying; Chang, Hsien-Chang; Journal of Natural Products; vol. 66; 9; (2003); p. 1207 - 1211. |
| F2 | *Phellodendron amurense* | M027906 | Wu, Tian-Shung; Hsu, Meei-Yu; Kuo, Ping-Chung; Sreenivasulu; Damu; Su, Chung-Ren; Li, Chia-Ying; Chang, Hsien-Chang; Journal of Natural Products; vol. 66; 9; (2003); p. 1207 - 1211. |
| F2 | *Phellodendron amurense* | M027949 | Tomita; Kunitomo; Yakugaku Zasshi; vol. 80; (1960); p. 880,884; Chem.Abstr.; 24828; (1960). |
| F2 | *Phellodendron amurense* | M028029 | Mori; Fuchigami; Inoue; Nagai; Koda; Nishioka; Planta Medica; vol. 60; 5; (1994); p. 445 - 449. |
| F2 | *Phellodendron amurense* | M028134 | Wu, Tian-Shung; Hsu, Meei-Yu; Kuo, Ping-Chung; Sreenivasulu; Damu; Su, Chung-Ren; Li, Chia-Ying; Chang, Hsien-Chang; Journal of Natural Products; vol. 66; 9; (2003); p. 1207 - 1211. |
| F2 | *Phellodendron amurense* | M028329 | Retrieved from CNPD |
| F2 | *Phellodendron amurense* | M028443 | Retrieved from CNPD |
| F2 | *Phellodendron amurense* | M028477 | Retrieved from CNPD |
| F2 | *Phellodendron amurense* | M028529 | Leu, Chien-Hsing; Li, Chia-Ying; Yao, Xinsheng; Wu, Tian-Shung; Chemical and Pharmaceutical Bulletin; vol. 54; 9; (2006); p. 1308 - 1311. |
| F2 | *Phellodendron amurense* | M028977 | Leu, Chien-Hsing; Li, Chia-Ying; Yao, Xinsheng; Wu, Tian-Shung; Chemical and Pharmaceutical Bulletin; vol. 54; 9; (2006); p. 1308 - 1311. |
| F2 | *Phellodendron amurense* | M028980 | Leu, Chien-Hsing; Li, Chia-Ying; Yao, Xinsheng; Wu, Tian-Shung; Chemical and Pharmaceutical Bulletin; vol. 54; 9; (2006); p. 1308 - 1311. |
| F2 | *Phellodendron amurense* | M029133 | Wu, Tian-Shung; Hsu, Meei-Yu; Kuo, Ping-Chung; Sreenivasulu; Damu; Su, Chung-Ren; Li, Chia-Ying; Chang, Hsien-Chang; Journal of Natural Products; vol. 66; 9; (2003); p. 1207 - 1211. |
| F2 | *Phellodendron amurense* | M029302 | Nelson; Journal of the American Chemical Society; vol. 60; (1938); p. 921. |
| F2 | *Phellodendron amurense* | M029420 | 汪纪武等. 植物药有效成分手册. 北京: 人民卫生出版社, 1986 |
| F2 | *Phellodendron amurense* | M029568 | Leu, Chien-Hsing; Li, Chia-Ying; Yao, Xinsheng; Wu, Tian-Shung; Chemical and Pharmaceutical Bulletin; vol. 54; 9; (2006); p. 1308 - 1311. |
| F2 | *Phellodendron amurense* | M029996 | Leu, Chien-Hsing; Li, Chia-Ying; Yao, Xinsheng; Wu, Tian-Shung; Chemical and Pharmaceutical Bulletin; vol. 54; 9; (2006); p. 1308 - 1311. |
| F2 | *Phellodendron amurense* | M030014 | 阴健等. 中药现代研究与临床应用(1). 北京: 学苑出版社, 1993 |
| F2 | *Phellodendron amurense* | M030711 | Ida, Yoshiteru; Satih, Yohko; Ohtsuka, Masumi; Nagasao, Miki; Shoji, Junzo; Phytochemistry (Elsevier); vol. 35; 1; (1994); p. 209 - 216. |
| F2 | *Phellodendron amurense* | M031153 | Mori; Fuchigami; Inoue; Nagai; Koda; Nishioka; Planta Medica; vol. 60; 5; (1994); p. 445 - 449. |
| F2 | *Phellodendron amurense* | M031280 | Ida, Yoshiteru; Satih, Yohko; Ohtsuka, Masumi; Nagasao, Miki; Shoji, Junzo; Phytochemistry (Elsevier); vol. 35; 1; (1994); p. 209 - 216. |
| F2 | *Phellodendron amurense* | M031293 | Kawaguchi; Kim; Ishida; Ahn; Yamamoto; Yamaoka; Kozuka; Goto; Takahashi; Agricultural and Biological Chemistry; vol. 53; 10; (1989); p. 2635 - 2640. |
| F2 | *Phellodendron amurense* | M031315 | Ikuta, Akira; Nakamura, Takayuki; Urabe, Hisao; Phytochemistry; vol. 48; 2; (1998); p. 285 - 291. |
| F2 | *Phellodendron amurense* | M031394 | Ida, Yoshiteru; Satih, Yohko; Ohtsuka, Masumi; Nagasao, Miki; Shoji, Junzo; Phytochemistry (Elsevier); vol. 35; 1; (1994); p. 209 - 216. |
| F2 | *Phellodendron amurense* | M031495 | 阴健等. 中药现代研究与临床应用(1). 北京: 学苑出版社, 1993 |
| F2 | *Phellodendron amurense* | M032155 | Wu, Tian-Shung; Hsu, Meei-Yu; Kuo, Ping-Chung; Sreenivasulu; Damu; Su, Chung-Ren; Li, Chia-Ying; Chang, Hsien-Chang; Journal of Natural Products; vol. 66; 9; (2003); p. 1207 - 1211. |
| F2 | *Phellodendron amurense* | M032628 | Wada; Yagi; Matsumura; Sasaki; Sakata; Haga; Chemical and Pharmaceutical Bulletin; vol. 38; 8; (1990); p. 2332 - 2334. |
| F2 | *Phellodendron amurense* | M032629 | Buckingham J(Executive Editor): et al. Dictionary of Natural Products, Vol 1-7, Chapman & Hall, London, 1994; 1995, Vol 8; 1996, Vol 9; 1997, Vol 10; 1998, Vol 11. |
| F2 | *Phellodendron amurense* | M032876 | Retrieved from CNPD |
| F2 | *Phellodendron amurense* | M033789 | Wu, Tian-Shung; Hsu, Meei-Yu; Kuo, Ping-Chung; Sreenivasulu; Damu; Su, Chung-Ren; Li, Chia-Ying; Chang, Hsien-Chang; Journal of Natural Products; vol. 66; 9; (2003); p. 1207 - 1211. |
| F2 | *Prunus persica* | M000002 | Retrieved from CNPD |
| F2 | *Prunus persica* | M000991 | Fan, Wenzhe; Tezuka, Yasuhiro; Kadota, Shigetoshi; Chemical & Pharmaceutical Bulletin; vol. 48; 7; (2000); p. 1055 - 1061. |
| F2 | *Prunus persica* | M001130 | Bhaskar, K. Vijaya; Chu, W.-L. Alexis; Gaskin, Paul A.; Mander, Lewis N.; Murofushi, Noboru; Pearce, David W.; Pharis, Richard P.; Takahashi, Nobutaka; Yamaguchi, Isomaro; Tetrahedron Letters; vol. 32; 43; (1991); p. 6203 - 6206. |
| F2 | *Prunus persica* | M001420 | Collot; Rabate; Comptes Rendus Hebdomadaires des Seances de l'Academie des Sciences; vol. 202; (1936); p. 1208. |
| F2 | *Prunus persica* | M001977 | (1) 江苏新医学院. 中药大辞典. 上海: 上海科学技术出版社, 1977. (2) 国家中医药管理局《中华本草》编委会. 中华本草. Vol. 1-30 上海:上海科学技术出版社, 1999. |
| F2 | *Prunus persica* | M002195 | El Lahlou, Hassane; Hirai, Nobuhiro; Tsuda, Mitsuya; Ohigashi, Hajime; Phytochemistry; vol. 52; 4; (1999); p. 623 - 629. |
| F2 | *Prunus persica* | M002450 | Fan, Wenzhe; Tezuka, Yasuhiro; Kadota, Shigetoshi; Chemical & Pharmaceutical Bulletin; vol. 48; 7; (2000); p. 1055 - 1061. |
| F2 | *Prunus persica* | M002683 | Retrieved from CNPD |
| F2 | *Prunus persica* | M003036 | Pacheco; Grouiller; Bulletin de la Societe Chimique de France; (1961); p. 1704. |
| F2 | *Prunus persica* | M003374 | Gross, Kenneth C.; Sams, Carl E.; Phytochemistry (Elsevier); vol. 23; 11; (1984); p. 2457 - 2462. |
| F2 | *Prunus persica* | M003598 | Fan, Wenzhe; Tezuka, Yasuhiro; Kadota, Shigetoshi; Chemical & Pharmaceutical Bulletin; vol. 48; 7; (2000); p. 1055 - 1061. |
| F2 | *Prunus persica* | M004329 | (1) 江苏新医学院. 中药大辞典. 上海: 上海科学技术出版社, 1977. (2) 孙文基等. 天然活性成分简明手册. 北京: 中国医药科技出版社, 1998. |
| F2 | *Prunus persica* | M005541 | Ohigashi; Minami; Fukui; et al.; Agricultural and Biological Chemistry; vol. 46; 10; (1982); p. 2555 - 2561. |
| F2 | *Prunus persica* | M005596 | Retrieved from CNPD |
| F2 | *Prunus persica* | M005917 | Gross, Kenneth C.; Sams, Carl E.; Phytochemistry (Elsevier); vol. 23; 11; (1984); p. 2457 - 2462. |
| F2 | *Prunus persica* | M006198 | Fan, Wenzhe; Tezuka, Yasuhiro; Kadota, Shigetoshi; Chemical & Pharmaceutical Bulletin; vol. 48; 7; (2000); p. 1055 - 1061. |
| F2 | *Prunus persica* | M006421 | Izzo, Riccardo; Scartazza, Andrea; Masia, Andrea; Galleschi, Luciano; Quartacci, Mike F.; Navari-Izzo, Flavia; Phytochemistry (Elsevier); vol. 39; 6; (1995); p. 1328 - 1334. |
| F2 | *Prunus persica* | M006655 | Fan, Wenzhe; Tezuka, Yasuhiro; Kadota, Shigetoshi; Chemical & Pharmaceutical Bulletin; vol. 48; 7; (2000); p. 1055 - 1061. |
| F2 | *Prunus persica* | M006737 | Gross, Kenneth C.; Sams, Carl E.; Phytochemistry (Elsevier); vol. 23; 11; (1984); p. 2457 - 2462. |
| F2 | *Prunus persica* | M006975 | Gross, Kenneth C.; Sams, Carl E.; Phytochemistry (Elsevier); vol. 23; 11; (1984); p. 2457 - 2462. |
| F2 | *Prunus persica* | M007184 | Fan, Wenzhe; Tezuka, Yasuhiro; Kadota, Shigetoshi; Chemical & Pharmaceutical Bulletin; vol. 48; 7; (2000); p. 1055 - 1061. |
| F2 | *Prunus persica* | M007201 | Fan, Wenzhe; Tezuka, Yasuhiro; Kadota, Shigetoshi; Chemical & Pharmaceutical Bulletin; vol. 48; 7; (2000); p. 1055 - 1061. |
| F2 | *Prunus persica* | M007382 | Fan, Wenzhe; Tezuka, Yasuhiro; Kadota, Shigetoshi; Chemical & Pharmaceutical Bulletin; vol. 48; 7; (2000); p. 1055 - 1061. |
| F2 | *Prunus persica* | M007437 | Kunz; Adam; Chem. Zentralbl.; vol. 77; I; (1906); p. 1850. |
| F2 | *Prunus persica* | M007532 | Fan, Wenzhe; Tezuka, Yasuhiro; Kadota, Shigetoshi; Chemical & Pharmaceutical Bulletin; vol. 48; 7; (2000); p. 1055 - 1061. |
| F2 | *Prunus persica* | M007756 | Knapp, Holger; Weigand, Cornelia; Gloser, Juergen; Winterhalter, Peter; Journal of Agricultural and Food Chemistry; vol. 45; 4; (1997); p. 1309 - 1313. |
| F2 | *Prunus persica* | M008118 | Retrieved from CNPD |
| F2 | *Prunus persica* | M008964 | Fan, Wenzhe; Tezuka, Yasuhiro; Kadota, Shigetoshi; Chemical & Pharmaceutical Bulletin; vol. 48; 7; (2000); p. 1055 - 1061. |
| F2 | *Prunus persica* | M009433 | (1) 阴健等. 中药现代研究与临床应用(1). 北京: 学苑出版社, 1993. (2) 江苏新医学院. 中药大辞典. 上海: 上海科学技术出版社, 1977. (3) 孙文基等. 天然活性成分简明手册. 北京: 中国医药科技出版社, 1998. (4) 国�抑幸揭┕芾砭帧吨谢静荨繁辔� 中华本草. Vol. 1-30 上海:上海科学技术出版社, 1999. |
| F2 | *Prunus persica* | M009790 | Fukuda, Toshiyuki; Ito, Hideyuki; Mukainaka, Teruo; Tokuda, Harukuni; Nishino, Hoyoku; Yoshida, Takashi; Biological and Pharmaceutical Bulletin; vol. 26; 2; (2003); p. 271 - 273. |
| F2 | *Prunus persica* | M010032 | Fan, Wenzhe; Tezuka, Yasuhiro; Kadota, Shigetoshi; Chemical & Pharmaceutical Bulletin; vol. 48; 7; (2000); p. 1055 - 1061. |
| F2 | *Prunus persica* | M010126 | Fan, Wenzhe; Tezuka, Yasuhiro; Kadota, Shigetoshi; Chemical & Pharmaceutical Bulletin; vol. 48; 7; (2000); p. 1055 - 1061. |
| F2 | *Prunus persica* | M011113 | Ohigashi; Minami; Fukui; et al.; Agricultural and Biological Chemistry; vol. 46; 10; (1982); p. 2555 - 2561. |
| F2 | *Prunus persica* | M011856 | (1) 汪纪武等. 植物药有效成分手册. 北京: 人民卫生出版社, 1986. (2) Takamoto S, et al. 药学杂志(日), 1977, 97 (1): 109. (3) 欧明等. 简明中药成分手册. 北京: 中国医药科技出版社, 2003. |
| F2 | *Prunus persica* | M011957 | Gross, Kenneth C.; Sams, Carl E.; Phytochemistry (Elsevier); vol. 23; 11; (1984); p. 2457 - 2462. |
| F2 | *Prunus persica* | M012105 | Fukuda, Toshiyuki; Ito, Hideyuki; Mukainaka, Teruo; Tokuda, Harukuni; Nishino, Hoyoku; Yoshida, Takashi; Biological and Pharmaceutical Bulletin; vol. 26; 2; (2003); p. 271 - 273. |
| F2 | *Prunus persica* | M012174 | El Lahlou, Hassane; Hirai, Nobuhiro; Tsuda, Mitsuya; Ohigashi, Hajime; Phytochemistry; vol. 52; 4; (1999); p. 623 - 629. |
| F2 | *Prunus persica* | M013486 | Fan, Wenzhe; Tezuka, Yasuhiro; Kadota, Shigetoshi; Chemical & Pharmaceutical Bulletin; vol. 48; 7; (2000); p. 1055 - 1061. |
| F2 | *Prunus persica* | M013880 | Retrieved from CNPD |
| F2 | *Prunus persica* | M014225 | (1) 汪纪武等. 植物药有效成分手册. 北京: 人民卫生出版社, 1986. (2) 阴健等. 中药现代研究与临床应用(1). 北京: 学苑出版社, 1993. (3) 江苏新医学院. 中药大辞典. 上海: 上海科学技术出版社, 1977. |
| F2 | *Prunus persica* | M014267 | Ohigashi; Minami; Fukui; et al.; Agricultural and Biological Chemistry; vol. 46; 10; (1982); p. 2555 - 2561. |
| F2 | *Prunus persica* | M014731 | Izzo, Riccardo; Scartazza, Andrea; Masia, Andrea; Galleschi, Luciano; Quartacci, Mike F.; Navari-Izzo, Flavia; Phytochemistry (Elsevier); vol. 39; 6; (1995); p. 1328 - 1334. |
| F2 | *Prunus persica* | M015112 | Yamaguchi et al.; Agricultural and Biological Chemistry; vol. 39; (1975); p. 2405,2410. |
| F2 | *Prunus persica* | M017661 | Retrieved from CNPD |
| F2 | *Prunus persica* | M021592 | El Lahlou, Hassane; Hirai, Nobuhiro; Tsuda, Mitsuya; Ohigashi, Hajime; Phytochemistry; vol. 52; 4; (1999); p. 623 - 629. |
| F2 | *Prunus persica* | M021999 | (1) 江苏新医学院. 中药大辞典. 上海: 上海科学技术出版社, 1977. (2) 国家中医药管理局《中华本草》编委会. 中华本草. Vol. 1-30 上海:上海科学技术出版社, 1999. |
| F2 | *Prunus persica* | M023676 | Fukuda, Toshiyuki; Ito, Hideyuki; Mukainaka, Teruo; Tokuda, Harukuni; Nishino, Hoyoku; Yoshida, Takashi; Biological and Pharmaceutical Bulletin; vol. 26; 2; (2003); p. 271 - 273. |
| F2 | *Prunus persica* | M023714 | Retrieved from CNPD |
| F2 | *Prunus persica* | M023948 | Gross, Kenneth C.; Sams, Carl E.; Phytochemistry (Elsevier); vol. 23; 11; (1984); p. 2457 - 2462. |
| F2 | *Prunus persica* | M024108 | Fan, Wenzhe; Tezuka, Yasuhiro; Kadota, Shigetoshi; Chemical & Pharmaceutical Bulletin; vol. 48; 7; (2000); p. 1055 - 1061. |
| F2 | *Prunus persica* | M024435 | Izzo, Riccardo; Scartazza, Andrea; Masia, Andrea; Galleschi, Luciano; Quartacci, Mike F.; Navari-Izzo, Flavia; Phytochemistry (Elsevier); vol. 39; 6; (1995); p. 1328 - 1334. |
| F2 | *Prunus persica* | M024727 | (1) 江苏新医学院. 中药大辞典. 上海: 上海科学技术出版社, 1977. (2) 国家中医药管理局《中华本草》编委会. 中华本草. Vol. 1-30 上海:上海科学技术出版社, 1999. (3) Buckingham J(Executive Editor): et al. Dictionary of Natural Products, Vol 1-7, Chapman & Hall, London, 1994; 1995, Vol 8; 1996, Vol 9; 1997, Vol 10; 1998, Vol 11.. |
| F2 | *Prunus persica* | M024783 | Izzo, Riccardo; Scartazza, Andrea; Masia, Andrea; Galleschi, Luciano; Quartacci, Mike F.; Navari-Izzo, Flavia; Phytochemistry (Elsevier); vol. 39; 6; (1995); p. 1328 - 1334. |
| F2 | *Prunus persica* | M024929 | Curl; Food Research; vol. 24; (1959); p. 413,416. |
| F2 | *Prunus persica* | M024936 | Morishige H, et al. 生药学杂志(日), 1983, 37 (1): 46 |
| F2 | *Prunus persica* | M025016 | Rosik et al.; Chemicke Zvesti; vol. 27; (1973); p. 688,689. |
| F2 | *Prunus persica* | M025234 | (1) 阴健等. 中药现代研究与临床应用(1). 北京: 学苑出版社, 1993. (2) 孙文基等. 天然活性成分简明手册. 北京: 中国医药科技出版社, 1998. |
| F2 | *Prunus persica* | M025298 | Retrieved from CNPD |
| F2 | *Prunus persica* | M025587 | El Lahlou, Hassane; Hirai, Nobuhiro; Tsuda, Mitsuya; Ohigashi, Hajime; Phytochemistry; vol. 52; 4; (1999); p. 623 - 629. |
| F2 | *Prunus persica* | M025647 | Fan, Wenzhe; Tezuka, Yasuhiro; Kadota, Shigetoshi; Chemical & Pharmaceutical Bulletin; vol. 48; 7; (2000); p. 1055 - 1061. |
| F2 | *Prunus persica* | M025726 | Siddiqui; Sen; Journal of the Indian Chemical Society; vol. 46; (1969); p. 755. |
| F2 | *Prunus persica* | M026099 | Molnar, Peter; Szabolcs, Jozsef; Radics, Lajos; Phytochemistry (Elsevier); vol. 26; 5; (1987); p. 1493 - 1496. |
| F2 | *Prunus persica* | M026260 | (1) 季宇彬等. 中药抗肿瘤有效成分药理与应用. 哈尔滨: 黑龙江科学技术出版社, 1995. (2) 江苏新医学院. 中药大辞典. 上海: 上海科学技术出版社, 1977. (3) 魏均娴等. 中国中药杂志, 1997, 22 (4): 228. (4) 尚明英�� 中国中药杂志, 1998, 23 (10): 614. (5) 孙文基等. 天然活性成分简明手册. 北京: 中国医药科技出版社, 1998. (6) 国家中医药管理局《中华本草》编委会. 中华本草. Vol. 1-30 上海:上海科学技术出版社, 1999. (7) Hou AJ, et al. JNP, 2001, 64 (1): 65. (8) Lee D, et al. JNP, 2001, 64 (10): 1286. (9) Danelutte AP, et al. Phytochemistry, 2003, 64, 555. (10) ZHANG Y-J, et al. Chem Pharm Bull, 2002, 50 (6): 841. (11) Calixto JB, et al. Planta Med, 2003, 69 (11): 973. (12) Lin JH, et al. JNP, 2002, 65 (5): 638. (13) Chiang YM, et al. JNP, 2003, 66 (8): 1070. (14) Park S-H, et al. Planta Med, 2005, 71 (1): 24. |
| F2 | *Prunus persica* | M026476 | Siddiqui; Sen; Journal of the Indian Chemical Society; vol. 46; (1969); p. 755. |
| F2 | *Prunus persica* | M026861 | Knapp, Holger; Weigand, Cornelia; Gloser, Juergen; Winterhalter, Peter; Journal of Agricultural and Food Chemistry; vol. 45; 4; (1997); p. 1309 - 1313. |
| F2 | *Prunus persica* | M026959 | (1) 阴健等. 中药现代研究与临床应用(1). 北京: 学苑出版社, 1993. (2) 季宇彬等. 中药抗肿瘤有效成分药理与应用. 哈尔滨: 黑龙江科学技术出版社, 1995. (3) 赵余庆等. 中国中药杂志, 1993, 18 (7): 428. (4) 孙文基等. 天然活性成分简明手册. 北京: 中国医药科技出版社, 1998. (5) 国家中医药管理局《中华本草》编委会. 中华本草. Vol. 1-30 上海:上海科学技术出版社, 1999. (6) 欧明等. 简明中药成分手册. 北京: 中国医药科技出版社, 2003. |
| F2 | *Prunus persica* | M027471 | Retrieved from CNPD |
| F2 | *Prunus persica* | M027597 | Fan, Wenzhe; Tezuka, Yasuhiro; Kadota, Shigetoshi; Chemical & Pharmaceutical Bulletin; vol. 48; 7; (2000); p. 1055 - 1061. |
| F2 | *Prunus persica* | M027598 | El Lahlou, Hassane; Hirai, Nobuhiro; Tsuda, Mitsuya; Ohigashi, Hajime; Phytochemistry; vol. 52; 4; (1999); p. 623 - 629. |
| F2 | *Prunus persica* | M027848 | Fan, Wenzhe; Tezuka, Yasuhiro; Kadota, Shigetoshi; Chemical & Pharmaceutical Bulletin; vol. 48; 7; (2000); p. 1055 - 1061. |
| F2 | *Prunus persica* | M028420 | Izzo, Riccardo; Scartazza, Andrea; Masia, Andrea; Galleschi, Luciano; Quartacci, Mike F.; Navari-Izzo, Flavia; Phytochemistry (Elsevier); vol. 39; 6; (1995); p. 1328 - 1334. |
| F2 | *Prunus persica* | M028991 | Takagi; Yamaki; Masuda; Kubota; Minami; Yakugaku Zasshi; vol. 97; 1; (1977); p. 109 - 111. |
| F2 | *Prunus persica* | M029233 | Fan, Wenzhe; Tezuka, Yasuhiro; Kadota, Shigetoshi; Chemical & Pharmaceutical Bulletin; vol. 48; 7; (2000); p. 1055 - 1061. |
| F2 | *Prunus persica* | M029323 | Siddiqui; Sen; Journal of the Indian Chemical Society; vol. 46; (1969); p. 755. |
| F2 | *Prunus persica* | M029750 | Fan, Wenzhe; Tezuka, Yasuhiro; Kadota, Shigetoshi; Chemical & Pharmaceutical Bulletin; vol. 48; 7; (2000); p. 1055 - 1061. |
| F2 | *Prunus persica* | M029927 | Fukuda, Toshiyuki; Ito, Hideyuki; Mukainaka, Teruo; Tokuda, Harukuni; Nishino, Hoyoku; Yoshida, Takashi; Biological and Pharmaceutical Bulletin; vol. 26; 2; (2003); p. 271 - 273. |
| F2 | *Prunus persica* | M030482 | Gross, Kenneth C.; Sams, Carl E.; Phytochemistry (Elsevier); vol. 23; 11; (1984); p. 2457 - 2462. |
| F2 | *Prunus persica* | M030517 | Kubala; Rosik; Collection of Czechoslovak Chemical Communications; vol. 42; (1977); p. 2809,2811,2812. |
| F2 | *Prunus persica* | M030666 | Fan, Wenzhe; Tezuka, Yasuhiro; Kadota, Shigetoshi; Chemical & Pharmaceutical Bulletin; vol. 48; 7; (2000); p. 1055 - 1061. |
| F2 | *Prunus persica* | M030979 | Molnar, Peter; Szabolcs, Jozsef; Radics, Lajos; Phytochemistry (Elsevier); vol. 26; 5; (1987); p. 1493 - 1496. |
| F2 | *Prunus persica* | M030991 | Yamaguchi et al.; Agricultural and Biological Chemistry; vol. 39; (1975); p. 2405,2410. |
| F2 | *Prunus persica* | M031176 | Fan, Wenzhe; Tezuka, Yasuhiro; Kadota, Shigetoshi; Chemical & Pharmaceutical Bulletin; vol. 48; 7; (2000); p. 1055 - 1061. |
| F2 | *Prunus persica* | M031854 | El Lahlou, Hassane; Hirai, Nobuhiro; Tsuda, Mitsuya; Ohigashi, Hajime; Phytochemistry; vol. 52; 4; (1999); p. 623 - 629. |
| F2 | *Prunus persica* | M031972 | 江苏新医学院. 中药大辞典. 上海: 上海科学技术出版社, 1977 |
| F2 | *Prunus persica* | M032088 | (1) 江苏新医学院. 中药大辞典. 上海: 上海科学技术出版社, 1977. (2) 孙文基等. 天然活性成分简明手册. 北京: 中国医药科技出版社, 1998. (3) ZHANG Y-J, et al. Chem Pharm Bull, 2002, 50 (6): 841. |
| F2 | *Prunus persica* | M032133 | Fan, Wenzhe; Tezuka, Yasuhiro; Kadota, Shigetoshi; Chemical & Pharmaceutical Bulletin; vol. 48; 7; (2000); p. 1055 - 1061. |
| F2 | *Prunus persica* | M032580 | Fukuda, Toshiyuki; Ito, Hideyuki; Mukainaka, Teruo; Tokuda, Harukuni; Nishino, Hoyoku; Yoshida, Takashi; Biological and Pharmaceutical Bulletin; vol. 26; 2; (2003); p. 271 - 273. |
| F2 | *Prunus persica* | M033170 | Fan, Wenzhe; Tezuka, Yasuhiro; Kadota, Shigetoshi; Chemical & Pharmaceutical Bulletin; vol. 48; 7; (2000); p. 1055 - 1061. |
| F2 | *Prunus persica* | M033228 | Fukuda, Toshiyuki; Ito, Hideyuki; Mukainaka, Teruo; Tokuda, Harukuni; Nishino, Hoyoku; Yoshida, Takashi; Biological and Pharmaceutical Bulletin; vol. 26; 2; (2003); p. 271 - 273. |
| F2 | *Prunus persica* | M033294 | Knapp, Holger; Weigand, Cornelia; Gloser, Juergen; Winterhalter, Peter; Journal of Agricultural and Food Chemistry; vol. 45; 4; (1997); p. 1309 - 1313. |
| F2 | *Prunus persica* | M033584 | (1) 俞文胜等. 中国中药杂志, 1993, 18 (9): 548. (2) 国家中医药管理局《中华本草》编委会. 中华本草. Vol. 1-30 上海:上海科学技术出版社, 1999. (3) 欧明等. 简明中药成分手册. 北京: 中国医药科技出版社, 2003. |
| F2 | *Prunus persica* | M033873 | El Lahlou, Hassane; Hirai, Nobuhiro; Tsuda, Mitsuya; Ohigashi, Hajime; Phytochemistry; vol. 52; 4; (1999); p. 623 - 629. |
| F2 | *Prunus persica* | M033877 | Fan, Wenzhe; Tezuka, Yasuhiro; Kadota, Shigetoshi; Chemical & Pharmaceutical Bulletin; vol. 48; 7; (2000); p. 1055 - 1061. |
| F2 | *Prunus persica* | M034055 | (1) 江苏新医学院. 中药大辞典. 上海: 上海科学技术出版社, 1977. (2) Buckingham J(Executive Editor): et al. Dictionary of Natural Products, Vol 1-7, Chapman & Hall, London, 1994; 1995, Vol 8; 1996, Vol 9; 1997, Vol 10; 1998, Vol 11.. |
| F2 | *Prunus persica* | M034141 | Yamaguchi et al.; Agricultural and Biological Chemistry; vol. 39; (1975); p. 2405,2410. |
| F2 | *Rheum palmatum* | M000099 | Wagner; Hoerhammer; Z. Naturforsch., B: Anorg. Chem., Org. Chem., Biochem., Biophys.,; vol. 18; (1963); p. 89. |
| F2 | *Rheum palmatum* | M001762 | Zhang, Cun; Li, Li; Xiao, Yong-Qing; Tian, Guo-Fang; Chen, Dong-Dong; Wang, Yun; Li, Yu-Tian; Huang, Wen-Qian; Journal of Asian Natural Products Research; vol. 12; 12; (2010); p. 1026 - 1032. |
| F2 | *Rheum palmatum* | M002138 | Tschirch; Eijken; Schweiz. Wochenschr. f. Chemie u. Pharmazie; vol. 42; p. 551,553; Chem. Zentralbl.; vol. 76; II; (1905); p. 144. |
| F2 | *Rheum palmatum* | M002578 | Tschirch; Eijken; Schweizerische Wochensch. f. Chemie u. Pharmazie; vol. 42; p. 553; Chem. Zentralbl.; vol. 76; II; (1905); p. 144.; Beilstein; Chemische Berichte; vol. 15; (1882); p. 902. |
| F2 | *Rheum palmatum* | M004237 | Wagner; Hoerhammer; Z. Naturforsch., B: Anorg. Chem., Org. Chem., Biochem., Biophys.,; vol. 18; (1963); p. 89. |
| F2 | *Rheum palmatum* | M005932 | Lemli et al.; Planta Medica; vol. 12; (1964); p. 107,109. |
| F2 | *Rheum palmatum* | M006353 | Wagner; Hoerhammer; Z. Naturforsch., B: Anorg. Chem., Org. Chem., Biochem., Biophys.,; vol. 18; (1963); p. 89. |
| F2 | *Rheum palmatum* | M006995 | (1) 阴健等. 中药现代研究与临床应用(1). 北京: 学苑出版社, 1993. (2) 国家中医药管理局《中华本草》编委会. 中华本草. Vol. 1-30 上海:上海科学技术出版社, 1999. (3) Buckingham J(Executive Editor): et al. Dictionary of Natural Products, Vol 1-7, Chapman & Hall, London, 1994; 1995, Vol 8; 1996, Vol 9; 1997, Vol 10; 1998, Vol 11.. (4) Aburjai TA, et al. Phytochemistry, 2000, 55, 407. |
| F2 | *Rheum palmatum* | M007300 | (1) 阴健等. 中药现代研究与临床应用(1). 北京: 学苑出版社, 1993. (2) 孙文基等. 天然活性成分简明手册. 北京: 中国医药科技出版社, 1998. (3) 国家中医药管理局《中华本草》编委会. 中华本草. Vol. 1-30 上海:上海科学技术出版社, 1999. (4) Krenn L, et al. JNP, 2003, 66 (8): 1107. |
| F2 | *Rheum palmatum* | M007612 | (1) 阴健等. 中药现代研究与临床应用(1). 北京: 学苑出版社, 1993. (2) 国家中医药管理局《中华本草》编委会. 中华本草. Vol. 1-30 上海:上海科学技术出版社, 1999. |
| F2 | *Rheum palmatum* | M008252 | (1) 阴健等. 中药现代研究与临床应用(1). 北京: 学苑出版社, 1993. (2) 国家中医药管理局《中华本草》编委会. 中华本草. Vol. 1-30 上海:上海科学技术出版社, 1999. |
| F2 | *Rheum palmatum* | M008306 | (1) 阴健等. 中药现代研究与临床应用(1). 北京: 学苑出版社, 1993. (2) 国家中医药管理局《中华本草》编委会. 中华本草. Vol. 1-30 上海:上海科学技术出版社, 1999. |
| F2 | *Rheum palmatum* | M008365 | Lemli et al.; Planta Medica; vol. 12; (1964); p. 107,109. |
| F2 | *Rheum palmatum* | M008476 | Wagner; Hoerhammer; Z. Naturforsch., B: Anorg. Chem., Org. Chem., Biochem., Biophys.,; vol. 18; (1963); p. 89. |
| F2 | *Rheum palmatum* | M008944 | Wagner; Hoerhammer; Z. Naturforsch., B: Anorg. Chem., Org. Chem., Biochem., Biophys.,; vol. 18; (1963); p. 89. |
| F2 | *Rheum palmatum* | M009065 | Lemli et al.; Pharmaceutisch Weekblad; vol. 98; (1963); p. 655,657. |
| F2 | *Rheum palmatum* | M009316 | 国家中医药管理局《中华本草》编委会. 中华本草. Vol. 1-30 上海:上海科学技术出版社, 1999 |
| F2 | *Rheum palmatum* | M009910 | (1) 阴健等. 中药现代研究与临床应用(1). 北京: 学苑出版社, 1993. (2) 国家中医药管理局《中华本草》编委会. 中华本草. Vol. 1-30 上海:上海科学技术出版社, 1999. |
| F2 | *Rheum palmatum* | M009924 | (1) 阴健等. 中药现代研究与临床应用(1). 北京: 学苑出版社, 1993. (2) 国家中医药管理局《中华本草》编委会. 中华本草. Vol. 1-30 上海:上海科学技术出版社, 1999. |
| F2 | *Rheum palmatum* | M010121 | (1) 江苏新医学院. 中药大辞典. 上海: 上海科学技术出版社, 1977. (2) 孙文基等. 天然活性成分简明手册. 北京: 中国医药科技出版社, 1998. |
| F2 | *Rheum palmatum* | M010531 | 国家中医药管理局《中华本草》编委会. 中华本草. Vol. 1-30 上海:上海科学技术出版社, 1999 |
| F2 | *Rheum palmatum* | M010755 | (1) 阴健等. 中药现代研究与临床应用(1). 北京: 学苑出版社, 1993. (2) 敏德等. 中国中药杂志, 1998, 23 (8): 486. (3) 国家中医药管理局《中华本草》编委会. 中华本草. Vol. 1-30 上海:上海科学技术出版社, 1999. (4) 肖培根等. 药学学报, 1980, 15 (1): 35. (5) Kubo I, et al. JNP, 1991, 54 (4): 1115. |
| F2 | *Rheum palmatum* | M010798 | Schnelle; Schratz; Planta Medica; vol. 14; (1966); p. 194,198. |
| F2 | *Rheum palmatum* | M012193 | Wang, Zhi-Wei; Wang, Jun-Song; Luo, Jun; Wei, Dan-Dan; Kong, Ling-Yi; Chemical and Pharmaceutical Bulletin; vol. 60; 2; (2012); p. 241 - 245. |
| F2 | *Rheum palmatum* | M012220 | Wagner; Hoerhammer; Z. Naturforsch., B: Anorg. Chem., Org. Chem., Biochem., Biophys.,; vol. 18; (1963); p. 89. |
| F2 | *Rheum palmatum* | M013476 | (1) 阴健等. 中药现代研究与临床应用(1). 北京: 学苑出版社, 1993. (2) 国家中医药管理局《中华本草》编委会. 中华本草. Vol. 1-30 上海:上海科学技术出版社, 1999. (3) XIAO et al. K. Chem Pharm Bull, 2002, 50 (5): 605. |
| F2 | *Rheum palmatum* | M014246 | 江苏新医学院. 中药大辞典. 上海: 上海科学技术出版社, 1977 |
| F2 | *Rheum palmatum* | M014421 | (1) 阴健等. 中药现代研究与临床应用(1). 北京: 学苑出版社, 1993. (2) 国家中医药管理局《中华本草》编委会. 中华本草. Vol. 1-30 上海:上海科学技术出版社, 1999. (3) Meksuriyen D, et al. JNP, 1988, 51 (6): 1129. (4) Takechi M, et al. Phytochemistry, 1985, 24 (10): 2245. (5) Kashiwada Y, et al. JNP, 1992, 55 (8): 1033. (6) 姚荣成等. 云南植物研究, 1989, 11 (2): 215. (7) Kashiwada Y, et al. Chem Pharm Bull, 1986, 34 (10): 4083. (8) Yazaki K, et al. Phytochemistry, 1989, 28 (2): 607. |
| F2 | *Rheum palmatum* | M014623 | 国家中医药管理局《中华本草》编委会. 中华本草. Vol. 1-30 上海:上海科学技术出版社, 1999 |
| F2 | *Rheum palmatum* | M015062 | (1) 阴健等. 中药现代研究与临床应用(1). 北京: 学苑出版社, 1993. (2) 国家中医药管理局《中华本草》编委会. 中华本草. Vol. 1-30 上海:上海科学技术出版社, 1999. (3) Buckingham J(Executive Editor): et al. Dictionary of Natural Products, Vol 1-7, Chapman & Hall, London, 1994; 1995, Vol 8; 1996, Vol 9; 1997, Vol 10; 1998, Vol 11.. (4) Kashiwada Y, et al. Chem Pharm Bull, 1986, 34 (10): 4083. |
| F2 | *Rheum palmatum* | M015711 | (1) 阴健等. 中药现代研究与临床应用(1). 北京: 学苑出版社, 1993. (2) 国家中医药管理局《中华本草》编委会. 中华本草. Vol. 1-30 上海:上海科学技术出版社, 1999. (3) Zhang YJ, et al. JNP, 2001, 64 (12): 1527. (4) De Leo M, et al. Planta Med, 2004, 70 (9): 841. |
| F2 | *Rheum palmatum* | M016111 | (1) 阴健等. 中药现代研究与临床应用(1). 北京: 学苑出版社, 1993. (2) 国家中医药管理局《中华本草》编委会. 中华本草. Vol. 1-30 上海:上海科学技术出版社, 1999. |
| F2 | *Rheum palmatum* | M017402 | Wang, Zhi-Wei; Wang, Jun-Song; Luo, Jun; Wei, Dan-Dan; Kong, Ling-Yi; Chemical and Pharmaceutical Bulletin; vol. 60; 2; (2012); p. 241 - 245. |
| F2 | *Rheum palmatum* | M017403 | Wang, Zhi-Wei; Wang, Jun-Song; Luo, Jun; Wei, Dan-Dan; Kong, Ling-Yi; Chemical and Pharmaceutical Bulletin; vol. 60; 2; (2012); p. 241 - 245. |
| F2 | *Rheum palmatum* | M017404 | Wang, Zhi-Wei; Wang, Jun-Song; Luo, Jun; Wei, Dan-Dan; Kong, Ling-Yi; Chemical and Pharmaceutical Bulletin; vol. 60; 2; (2012); p. 241 - 245. |
| F2 | *Rheum palmatum* | M017689 | (1) 阴健等. 中药现代研究与临床应用(1). 北京: 学苑出版社, 1993. (2) 国家中医药管理局《中华本草》编委会. 中华本草. Vol. 1-30 上海:上海科学技术出版社, 1999. |
| F2 | *Rheum palmatum* | M019088 | Wang, Zhi-Wei; Wang, Jun-Song; Luo, Jun; Wei, Dan-Dan; Kong, Ling-Yi; Chemical and Pharmaceutical Bulletin; vol. 60; 2; (2012); p. 241 - 245. |
| F2 | *Rheum palmatum* | M020305 | Zhang, Cun; Li, Li; Xiao, Yong-Qing; Tian, Guo-Fang; Chen, Dong-Dong; Wang, Yun; Li, Yu-Tian; Huang, Wen-Qian; Journal of Asian Natural Products Research; vol. 12; 12; (2010); p. 1026 - 1032. |
| F2 | *Rheum palmatum* | M022082 | (1) 阴健等. 中药现代研究与临床应用(1). 北京: 学苑出版社, 1993. (2) 国家中医药管理局《中华本草》编委会. 中华本草. Vol. 1-30 上海:上海科学技术出版社, 1999. |
| F2 | *Rheum palmatum* | M022267 | (1) 阴健等. 中药现代研究与临床应用(1). 北京: 学苑出版社, 1993. (2) 国家中医药管理局《中华本草》编委会. 中华本草. Vol. 1-30 上海:上海科学技术出版社, 1999. (3) Kashiwada Y, et al. Chem Pharm Bull, 1984, 32 (9): 3501. |
| F2 | *Rheum palmatum* | M022573 | (1) 阴健等. 中药现代研究与临床应用(1). 北京: 学苑出版社, 1993. (2) 国家中医药管理局《中华本草》编委会. 中华本草. Vol. 1-30 上海:上海科学技术出版社, 1999. |
| F2 | *Rheum palmatum* | M022588 | Lemli et al.; Pharmaceutisch Weekblad; vol. 98; (1963); p. 529. |
| F2 | *Rheum palmatum* | M024568 | (1) 阴健等. 中药现代研究与临床应用(1). 北京: 学苑出版社, 1993. (2) 国家中医药管理局《中华本草》编委会. 中华本草. Vol. 1-30 上海:上海科学技术出版社, 1999. |
| F2 | *Rheum palmatum* | M024624 | 国家中医药管理局《中华本草》编委会. 中华本草. Vol. 1-30 上海:上海科学技术出版社, 1999 |
| F2 | *Rheum palmatum* | M024973 | Cui, Xing-Ri; Tsukada, Maiko; Suzuki, Nao; Shimamura, Takeshi; Gao, Li; Koyanagi, Jyunichi; Komada, Fusao; Saito, Setsuo; European Journal of Medicinal Chemistry; vol. 43; 6; (2008); p. 1206 - 1215. |
| F2 | *Rheum palmatum* | M025327 | Zhang, Cun; Li, Li; Xiao, Yong-Qing; Tian, Guo-Fang; Chen, Dong-Dong; Wang, Yun; Li, Yu-Tian; Huang, Wen-Qian; Journal of Asian Natural Products Research; vol. 12; 12; (2010); p. 1026 - 1032. |
| F2 | *Rheum palmatum* | M025554 | (1) 阴健等. 中药现代研究与临床应用(1). 北京: 学苑出版社, 1993. (2) 国家中医药管理局《中华本草》编委会. 中华本草. Vol. 1-30 上海:上海科学技术出版社, 1999. (3) Zhang YJ, et al. JNP, 2001, 64 (6): 870. |
| F2 | *Rheum palmatum* | M025696 | (1) 阴健等. 中药现代研究与临床应用(1). 北京: 学苑出版社, 1993. (2) 国家中医药管理局《中华本草》编委会. 中华本草. Vol. 1-30 上海:上海科学技术出版社, 1999. |
| F2 | *Rheum palmatum* | M025705 | (1) 阴健等. 中药现代研究与临床应用(1). 北京: 学苑出版社, 1993. (2) 国家中医药管理局《中华本草》编委会. 中华本草. Vol. 1-30 上海:上海科学技术出版社, 1999. (3) Zhang YJ, et al. JNP, 2001, 64 (12): 1527. |
| F2 | *Rheum palmatum* | M026451 | Wagner; Hoerhammer; Z. Naturforsch., B: Anorg. Chem., Org. Chem., Biochem., Biophys.,; vol. 18; (1963); p. 89. |
| F2 | *Rheum palmatum* | M028648 | (1) 阴健等. 中药现代研究与临床应用(1). 北京: 学苑出版社, 1993. (2) 江苏新医学院. 中药大辞典. 上海: 上海科学技术出版社, 1977. (3) 钟永利等. 天然产物研究与开发, 1998, 10 (2): 15. (4) 国家中医药管理局《�谢静荨繁辔� 中华本草. Vol. 1-30 上海:上海科学技术出版社, 1999. (5) Krenn L, et al. JNP, 2003, 66 (8): 1107. (6) Chaubal R, et al. Planta Med, 2003, 69, 287. |
| F2 | *Rheum palmatum* | M029046 | (1) 汪纪武等. 植物药有效成分手册. 北京: 人民卫生出版社, 1986. (2) 季宇彬等. 中药抗肿瘤有效成分药理与应用. 哈尔滨: 黑龙江科学技术出版社, 1995. (3) 江苏新医学院. 中药大辞典. 上海: 上海科学技术出版社, 1977. (4) 国家中医药管理局《中华本草》编委会. 中华本草(精选本上下册). 上海: 上海科学技术出版社, 1998. (5) 欧明等. 简明中药成分手册. 北京: 中国医药科技出版社, 2003. |
| F2 | *Rheum palmatum* | M029348 | Cui, Xing-Ri; Tsukada, Maiko; Suzuki, Nao; Shimamura, Takeshi; Gao, Li; Koyanagi, Jyunichi; Komada, Fusao; Saito, Setsuo; European Journal of Medicinal Chemistry; vol. 43; 6; (2008); p. 1206 - 1215. |
| F2 | *Rheum palmatum* | M030001 | Tschirch; Eijken; Schweizer.Wochensch.f.Chemie u.Pharmazie; vol. 42; p. 551,553; Chem. Zentralbl.; vol. 76; II; (1905); p. 144. |
| F2 | *Rheum palmatum* | M030138 | (1) 阴健等. 中药现代研究与临床应用(1). 北京: 学苑出版社, 1993. (2) 国家中医药管理局《中华本草》编委会. 中华本草. Vol. 1-30 上海:上海科学技术出版社, 1999. |
| F2 | *Rheum palmatum* | M030507 | Schnelle; Schratz; Planta Medica; vol. 14; (1966); p. 194,198. |
| F2 | *Rheum palmatum* | M030776 | 国家中医药管理局《中华本草》编委会. 中华本草. Vol. 1-30 上海:上海科学技术出版社, 1999 |
| F2 | *Rheum palmatum* | M031235 | Wagner; Hoerhammer; Z. Naturforsch., B: Anorg. Chem., Org. Chem., Biochem., Biophys.,; vol. 18; (1963); p. 89. |
| F2 | *Rheum palmatum* | M031466 | (1) 阴健等. 中药现代研究与临床应用(1). 北京: 学苑出版社, 1993. (2) 国家中医药管理局《中华本草》编委会. 中华本草. Vol. 1-30 上海:上海科学技术出版社, 1999. |
| F2 | *Rheum palmatum* | M031515 | Lin, Yun-Lian; Wu, Ching-Fen; Huang, Yi-Tsau; Planta Medica; vol. 74; 10; (2008); p. 1246 - 1252. |
| F2 | *Rheum palmatum* | M031837 | (1) 阴健等. 中药现代研究与临床应用(1). 北京: 学苑出版社, 1993. (2) 国家中医药管理局《中华本草》编委会. 中华本草. Vol. 1-30 上海:上海科学技术出版社, 1999. |
| F2 | *Rheum palmatum* | M031946 | Tschirch; Eijken; Schweiz.Wochensch.f.Chemie u.Pharmazie; vol. 42; p. 553; Chem. Zentralbl.; vol. 76; II; (1905); p. 145.; Beilstein; Chemische Berichte; vol. 15; (1882); p. 901.; Schroff; Neues Jahrb.f.Pharmacie; vol. 15; (1861); p. 128. |
| F2 | *Rheum palmatum* | M032709 | Zhang, Cun; Li, Li; Xiao, Yong-Qing; Tian, Guo-Fang; Chen, Dong-Dong; Wang, Yun; Li, Yu-Tian; Huang, Wen-Qian; Journal of Asian Natural Products Research; vol. 12; 12; (2010); p. 1026 - 1032. |
| F2 | *Rheum palmatum* | M032845 | Cui, Xing-Ri; Tsukada, Maiko; Suzuki, Nao; Shimamura, Takeshi; Gao, Li; Koyanagi, Jyunichi; Komada, Fusao; Saito, Setsuo; European Journal of Medicinal Chemistry; vol. 43; 6; (2008); p. 1206 - 1215. |
| F2 | *Rheum palmatum* | M033172 | (1) 阴健等. 中药现代研究与临床应用(1). 北京: 学苑出版社, 1993. (2) 国家中医药管理局《中华本草》编委会. 中华本草. Vol. 1-30 上海:上海科学技术出版社, 1999. |
| F2 | *Rheum palmatum* | M033582 | Retrieved from CNPD |
| F2 | *Rheum palmatum* | M033801 | Lemli et al.; Planta Medica; vol. 12; (1964); p. 107,109. |
| F2 | *Rheum palmatum* | M033956 | Retrieved from CNPD |
| F2 | *Rheum palmatum* | M034190 | Bionovo, Inc., A Delaware Corporation; US2009/312437; A1; (2009). |
| F2 | *strychnos nux-vomica* | M000023 | Baser, Kemal H. C.; Bisset, Norman G.; Phytochemistry (Elsevier); vol. 21; 6; (1982); p. 1423 - 1430. |
| F2 | *strychnos nux-vomica* | M000179 | (1) 阴健等. 中药现代研究与临床应用(1). 北京: 学苑出版社, 1993. (2) 江苏新医学院. 中药大辞典. 上海: 上海科学技术出版社, 1977. (3) 王曙等. 药学学报, 1992, 27 (2): 117. (4) 彭江南等. 中国中药杂志, 1994, 19 (11): 676. (5) 国家中医药管理局《中华本草》编委会. 中华本草. Vol. 1-30 上海:上海科学技术出版社, 1999. (6) Buckingham J(Executive Editor): et al. Dictionary of Natural Products, Vol 1-7, Chapman & Hall, London, 1994; 1995, Vol 8; 1996, Vol 9; 1997, Vol 10; 1998, Vol 11.. (7) Calixto JB, et al. Planta Med, 2003, 69 (11): 973. |
| F2 | *strychnos nux-vomica* | M000612 | Retrieved from CNPD |
| F2 | *strychnos nux-vomica* | M000748 | Zhang, Xiaozhe; Xu, Qing; Xiao, Hongbin; Liang, Xinmiao; Phytochemistry; vol. 64; 8; (2003); p. 1341 - 1344. |
| F2 | *strychnos nux-vomica* | M001050 | (1) 阴健等. 中药现代研究与临床应用(1). 北京: 学苑出版社, 1993. (2) 季宇彬等. 中药抗肿瘤有效成分药理与应用. 哈尔滨: 黑龙江科学技术出版社, 1995. (3) 宋蔚等. 中国中药杂志, 1997, 22 (6): 359. (4) 凌云等. 中国中药杂志, 1998, 23 (4): 232. (5) 黄西峰. 中国中药杂志, 1997, 22 (4): 247. (6) 孙文基等. 天然活性成分简明手册. 北京: 中国医药科技出版社, 1998. (7) 国家中医药管理局《中华本草》编委会. 中华本草. Vol. 1-30 上海:上海科学技术出版社, 1999. (8) Kimura T, et al. Phytochemistry, 2004, 65, 423. (9) YUAN Z, et al. Chem Pharm Bull, 2002, 50 (1): 73. (10) KITAJIMA M, et al. Chem Pharm Bull, 2005, 53 (10): 1355. (11) Yoshikawa M, et al. JNP, 2002, 65 (8): 1151. (12) Hou CC, et al. JNP, 2003, 66 (5): 625. (13) Itoh A, et al. JNP, 2003, 66 (9): 1212. (14) 毛水春等. 中国药物化学杂志, 2004, 14 (6): 326. (15) K?rm?z?bekmez H, et al. Planta Med, 2004, 70 (8): 711. (16) Zidorn C, et al. Phytochemistry, 2005, 66, 1691. (17) Chaubal R, et al. Planta Med, 2003, 69, 287. (18) 欧明等. 简明中药成分手册. 北京: 中国医药科技出版社, 2003. |
| F2 | *strychnos nux-vomica* | M001769 | Retrieved from CNPD |
| F2 | *strychnos nux-vomica* | M001965 | (1) 江苏新医学院. 中药大辞典. 上海: 上海科学技术出版社, 1977. (2) Buckingham J(Executive Editor): et al. Dictionary of Natural Products, Vol 1-7, Chapman & Hall, London, 1994; 1995, Vol 8; 1996, Vol 9; 1997, Vol 10; 1998, Vol 11.. |
| F2 | *strychnos nux-vomica* | M003360 | Corsaro, M. M.; Giudicianni, I.; Lanzetta, R.; Marciano, C. E.; Monaco, P.; Parrilli, M.; Phytochemistry (Elsevier); vol. 39; 6; (1995); p. 1377 - 1380. |
| F2 | *strychnos nux-vomica* | M003969 | Retrieved from CNPD |
| F2 | *strychnos nux-vomica* | M004471 | Yang, Guang-Ming; Tu, Xia; Liu, Liang-Jing; Pan, Yang; Fitoterapia; vol. 81; 7; (2010); p. 932 - 936. |
| F2 | *strychnos nux-vomica* | M005383 | COUNCIL OF SCIENTIFIC and INDUSTRIAL RESEARCH; WO2007/60686; A1; (2007). |
| F2 | *strychnos nux-vomica* | M005602 | Baser, Kemal H. C.; Bisset, Norman G.; Phytochemistry (Elsevier); vol. 21; 6; (1982); p. 1423 - 1430. |
| F2 | *strychnos nux-vomica* | M005655 | (1) 阴健等. 中药现代研究与临床应用(1). 北京: 学苑出版社, 1993. (2) 杨秀伟等. 中国中药杂志, 1993, 18 (12): 739. |
| F2 | *strychnos nux-vomica* | M005955 | Baser, Kemal H. C.; Bisset, Norman G.; Phytochemistry (Elsevier); vol. 21; 6; (1982); p. 1423 - 1430. |
| F2 | *strychnos nux-vomica* | M006032 | Zhang X, et al. Phytochemistry, 2003, 64, 1341 |
| F2 | *strychnos nux-vomica* | M006155 | (1) 阴健等. 中药现代研究与临床应用(1). 北京: 学苑出版社, 1993. (2) Buckingham J(Executive Editor): et al. Dictionary of Natural Products, Vol 1-7, Chapman & Hall, London, 1994; 1995, Vol 8; 1996, Vol 9; 1997, Vol 10; 1998, Vol 11.. |
| F2 | *strychnos nux-vomica* | M006890 | Retrieved from CNPD |
| F2 | *strychnos nux-vomica* | M007225 | Baser, Kemal H. C.; Bisset, Norman G.; Phytochemistry (Elsevier); vol. 21; 6; (1982); p. 1423 - 1430. |
| F2 | *strychnos nux-vomica* | M007408 | (1) 阴健等. 中药现代研究与临床应用(1). 北京: 学苑出版社, 1993. (2) 梁晓天主编. 常用中药基础研究. 第二卷. 北京: 科学出版社, 2007. (3) 赵世萍等. 中草药, 1997, 28 (3): 187. (4) 国家中医药管理局《中华本草》编委会. 中华本草. Vol. 1-30 上海:上海科学技术出版社, 1999. |
| F2 | *strychnos nux-vomica* | M007562 | Zhang X, et al. Phytochemistry, 2003, 64, 1341 |
| F2 | *strychnos nux-vomica* | M007574 | 阴健等. 中药现代研究与临床应用(1). 北京: 学苑出版社, 1993 |
| F2 | *strychnos nux-vomica* | M009126 | Baser, Kemal H. C.; Bisset, Norman G.; Phytochemistry (Elsevier); vol. 21; 6; (1982); p. 1423 - 1430. |
| F2 | *strychnos nux-vomica* | M009498 | Baser, Kemal H. C.; Bisset, Norman G.; Phytochemistry (Elsevier); vol. 21; 6; (1982); p. 1423 - 1430. |
| F2 | *strychnos nux-vomica* | M010458 | Baser, Kemal H. C.; Bisset, Norman G.; Phytochemistry (Elsevier); vol. 21; 6; (1982); p. 1423 - 1430. |
| F2 | *strychnos nux-vomica* | M011555 | Baser, Kemal H. C.; Bisset, Norman G.; Phytochemistry (Elsevier); vol. 21; 6; (1982); p. 1423 - 1430. |
| F2 | *strychnos nux-vomica* | M012120 | Baser, Kemal H. C.; Bisset, Norman G.; Phytochemistry (Elsevier); vol. 21; 6; (1982); p. 1423 - 1430. |
| F2 | *strychnos nux-vomica* | M014469 | Yang, Guang-Ming; Tu, Xia; Liu, Liang-Jing; Pan, Yang; Fitoterapia; vol. 81; 7; (2010); p. 932 - 936. |
| F2 | *strychnos nux-vomica* | M015104 | 江苏新医学院. 中药大辞典. 上海: 上海科学技术出版社, 1977 |
| F2 | *strychnos nux-vomica* | M015736 | 阴健等. 中药现代研究与临床应用(1). 北京: 学苑出版社, 1993 |
| F2 | *strychnos nux-vomica* | M015955 | Biala; Tits; Penelle; Frederich; Brandt; Prosperi; Llabres; Angenot; Journal of Natural Products; vol. 61; 1; (1998); p. 139 - 141. |
| F2 | *strychnos nux-vomica* | M015959 | Baser, Kemal H. C.; Bisset, Norman G.; Phytochemistry (Elsevier); vol. 21; 6; (1982); p. 1423 - 1430. |
| F2 | *strychnos nux-vomica* | M017804 | Zhao, Nan; Li, Li; Liu, Jun-Hong; Zhuang, Peng-Yu; Yu, Shi-Shan; Ma, Shuang-Gang; Qu, Jing; Chen, Nai-Hong; Wu, Li-Jun; Tetrahedron; vol. 68; 16; (2012); p. 3288 - 3294. |
| F2 | *strychnos nux-vomica* | M017886 | Fu, Yanhui; Zhang, Yu; He, Hongping; Hou, Li; Di, Yingtong; Li, Shunlin; Luo, Xiaodong; Hao, Xiaojiang; Journal of Natural Products; vol. 75; 11; (2012); p. 1987 - 1990. |
| F2 | *strychnos nux-vomica* | M017930 | Zhang, Jing Yu; Qu, Wei; Liang, Jing Yu; Chemistry of Natural Compounds; vol. 48; 5; (2012); p. 831 - 833; Khim. Prir. Soedin.; vol. 48; 5; (2012); p. 740 - 741,2. |
| F2 | *strychnos nux-vomica* | M018782 | Jonville, Marie-Caroline; Dive, Georges; Angenot, Luc; Bero, Joanne; Tits, Monique; Ollivier, Evelyne; Frederich, Michel; Phytochemistry; vol. 87; (2013); p. 157 - 163. |
| F2 | *strychnos nux-vomica* | M019022 | Baser, Kemal H. C.; Bisset, Norman G.; Phytochemistry (Elsevier); vol. 21; 6; (1982); p. 1423 - 1430. |
| F2 | *strychnos nux-vomica* | M019110 | Zhao, Nan; Li, Li; Liu, Jun-Hong; Zhuang, Peng-Yu; Yu, Shi-Shan; Ma, Shuang-Gang; Qu, Jing; Chen, Nai-Hong; Wu, Li-Jun; Tetrahedron; vol. 68; 16; (2012); p. 3288 - 3294. |
| F2 | *strychnos nux-vomica* | M019201 | Jonville, Marie-Caroline; Dive, Georges; Angenot, Luc; Bero, Joanne; Tits, Monique; Ollivier, Evelyne; Frederich, Michel; Phytochemistry; vol. 87; (2013); p. 157 - 163. |
| F2 | *strychnos nux-vomica* | M019424 | Baser, Kemal H. C.; Bisset, Norman G.; Phytochemistry (Elsevier); vol. 21; 6; (1982); p. 1423 - 1430. |
| F2 | *strychnos nux-vomica* | M019425 | Fu, Yanhui; Zhang, Yu; He, Hongping; Hou, Li; Di, Yingtong; Li, Shunlin; Luo, Xiaodong; Hao, Xiaojiang; Journal of Natural Products; vol. 75; 11; (2012); p. 1987 - 1990. |
| F2 | *strychnos nux-vomica* | M019531 | Zhao, Nan; Li, Li; Liu, Jun-Hong; Zhuang, Peng-Yu; Yu, Shi-Shan; Ma, Shuang-Gang; Qu, Jing; Chen, Nai-Hong; Wu, Li-Jun; Tetrahedron; vol. 68; 16; (2012); p. 3288 - 3294. |
| F2 | *strychnos nux-vomica* | M019628 | Jonville, Marie-Caroline; Dive, Georges; Angenot, Luc; Bero, Joanne; Tits, Monique; Ollivier, Evelyne; Frederich, Michel; Phytochemistry; vol. 87; (2013); p. 157 - 163. |
| F2 | *strychnos nux-vomica* | M019939 | Zhao, Nan; Li, Li; Liu, Jun-Hong; Zhuang, Peng-Yu; Yu, Shi-Shan; Ma, Shuang-Gang; Qu, Jing; Chen, Nai-Hong; Wu, Li-Jun; Tetrahedron; vol. 68; 16; (2012); p. 3288 - 3294. |
| F2 | *strychnos nux-vomica* | M020032 | Jonville, Marie-Caroline; Dive, Georges; Angenot, Luc; Bero, Joanne; Tits, Monique; Ollivier, Evelyne; Frederich, Michel; Phytochemistry; vol. 87; (2013); p. 157 - 163. |
| F2 | *strychnos nux-vomica* | M020242 | Fu, Yanhui; Zhang, Yu; He, Hongping; Hou, Li; Di, Yingtong; Li, Shunlin; Luo, Xiaodong; Hao, Xiaojiang; Journal of Natural Products; vol. 75; 11; (2012); p. 1987 - 1990. |
| F2 | *strychnos nux-vomica* | M020342 | Zhao, Nan; Li, Li; Liu, Jun-Hong; Zhuang, Peng-Yu; Yu, Shi-Shan; Ma, Shuang-Gang; Qu, Jing; Chen, Nai-Hong; Wu, Li-Jun; Tetrahedron; vol. 68; 16; (2012); p. 3288 - 3294. |
| F2 | *strychnos nux-vomica* | M020429 | Jonville, Marie-Caroline; Dive, Georges; Angenot, Luc; Bero, Joanne; Tits, Monique; Ollivier, Evelyne; Frederich, Michel; Phytochemistry; vol. 87; (2013); p. 157 - 163. |
| F2 | *strychnos nux-vomica* | M020750 | Zhao, Nan; Li, Li; Liu, Jun-Hong; Zhuang, Peng-Yu; Yu, Shi-Shan; Ma, Shuang-Gang; Qu, Jing; Chen, Nai-Hong; Wu, Li-Jun; Tetrahedron; vol. 68; 16; (2012); p. 3288 - 3294. |
| F2 | *strychnos nux-vomica* | M021188 | Zhao, Nan; Li, Li; Liu, Jun-Hong; Zhuang, Peng-Yu; Yu, Shi-Shan; Ma, Shuang-Gang; Qu, Jing; Chen, Nai-Hong; Wu, Li-Jun; Tetrahedron; vol. 68; 16; (2012); p. 3288 - 3294. |
| F2 | *strychnos nux-vomica* | M021632 | Zhao, Nan; Li, Li; Liu, Jun-Hong; Zhuang, Peng-Yu; Yu, Shi-Shan; Ma, Shuang-Gang; Qu, Jing; Chen, Nai-Hong; Wu, Li-Jun; Tetrahedron; vol. 68; 16; (2012); p. 3288 - 3294. |
| F2 | *strychnos nux-vomica* | M023058 | Retrieved from CNPD |
| F2 | *strychnos nux-vomica* | M023232 | Merz; Lehmann; Archiv der Pharmazie (Weinheim, Germany); vol. 290; (1957); p. 543,556.; Merz; Krebs; Archiv der Pharmazie (Weinheim, Germany); vol. 275; (1937); p. 217,235.; Dunstan; Short; Pharmaceutical Journal; vol. <3> 14; (1884); p. 1025.; Rosenthaler; Schweiz. Apoth. Ztg.; vol. 61; (1923); p. 398,399. |
| F2 | *strychnos nux-vomica* | M023257 | Fu, Yanhui; Zhang, Yu; He, Hongping; Hou, Li; Di, Yingtong; Li, Shunlin; Luo, Xiaodong; Hao, Xiaojiang; Journal of Natural Products; vol. 75; 11; (2012); p. 1987 - 1990. |
| F2 | *strychnos nux-vomica* | M023532 | Baser, Kemal H. C.; Bisset, Norman G.; Phytochemistry (Elsevier); vol. 21; 6; (1982); p. 1423 - 1430. |
| F2 | *strychnos nux-vomica* | M024162 | Zhang, Xiaozhe; Xu, Qing; Xiao, Hongbin; Liang, Xinmiao; Phytochemistry; vol. 64; 8; (2003); p. 1341 - 1344. |
| F2 | *strychnos nux-vomica* | M024204 | Martin et al.; Journal of the Chemical Society; (1952); p. 3603. |
| F2 | *strychnos nux-vomica* | M025063 | Baser, Kemal H. C.; Bisset, Norman G.; Phytochemistry (Elsevier); vol. 21; 6; (1982); p. 1423 - 1430. |
| F2 | *strychnos nux-vomica* | M026069 | (1) 阴健等. 中药现代研究与临床应用(1). 北京: 学苑出版社, 1993. (2) Cai BC, et al. 生药学杂志(日), 1990, 44 (1): 42. (3) Cai BC, et al. 生药学杂志(日), 1995, 49 (1): 39. |
| F2 | *strychnos nux-vomica* | M026231 | Baser, Kemal H. C.; Bisset, Norman G.; Phytochemistry (Elsevier); vol. 21; 6; (1982); p. 1423 - 1430. |
| F2 | *strychnos nux-vomica* | M026795 | (1) 阴健等. 中药现代研究与临床应用(1). 北京: 学苑出版社, 1993. (2) 杨秀伟等. 中国中药杂志, 1993, 18 (12): 739. (3) Cai BC, et al. 和汉医药学杂志(日), 1995, 12 (3): 173. (4) Cai BC, et al. 生药学杂志(��, 1990, 44 (1): 42. (5) Cai BC, et al. 生药学杂志(日), 1995, 49 (1): 39. |
| F2 | *strychnos nux-vomica* | M027010 | Retrieved from CNPD |
| F2 | *strychnos nux-vomica* | M027485 | Corsaro, M. M.; Giudicianni, I.; Lanzetta, R.; Marciano, C. E.; Monaco, P.; Parrilli, M.; Phytochemistry (Elsevier); vol. 39; 6; (1995); p. 1377 - 1380. |
| F2 | *strychnos nux-vomica* | M027603 | Bisset, Norman G.; Choudhury, Abdul K.; Houghton, Peter J.; Phytochemistry (Elsevier); vol. 28; 5; (1989); p. 1553 - 1554. |
| F2 | *strychnos nux-vomica* | M027953 | Baser, Kemal H. C.; Bisset, Norman G.; Phytochemistry (Elsevier); vol. 21; 6; (1982); p. 1423 - 1430. |
| F2 | *strychnos nux-vomica* | M028421 | Corsaro, M. M.; Giudicianni, I.; Lanzetta, R.; Marciano, C. E.; Monaco, P.; Parrilli, M.; Phytochemistry (Elsevier); vol. 39; 6; (1995); p. 1377 - 1380. |
| F2 | *strychnos nux-vomica* | M028429 | Bisset,N.G.; Choudhury,A.K.; Phytochemistry (Elsevier); vol. 13; (1974); p. 265 - 269. |
| F2 | *strychnos nux-vomica* | M029601 | Baser, Kemal H. C.; Bisset, Norman G.; Phytochemistry (Elsevier); vol. 21; 6; (1982); p. 1423 - 1430. |
| F2 | *strychnos nux-vomica* | M029809 | Baser, Kemal H. C.; Bisset, Norman G.; Phytochemistry (Elsevier); vol. 21; 6; (1982); p. 1423 - 1430. |
| F2 | *strychnos nux-vomica* | M030408 | Guggisberg et al.; Helvetica Chimica Acta; vol. 49; (1966); p. 1. |
| F2 | *strychnos nux-vomica* | M031302 | Baser, Kemal H. C.; Bisset, Norman G.; Phytochemistry (Elsevier); vol. 21; 6; (1982); p. 1423 - 1430. |
| F2 | *strychnos nux-vomica* | M031822 | Fu, Yanhui; Zhang, Yu; He, Hongping; Hou, Li; Di, Yingtong; Li, Shunlin; Luo, Xiaodong; Hao, Xiaojiang; Journal of Natural Products; vol. 75; 11; (2012); p. 1987 - 1990. |
| F2 | *strychnos nux-vomica* | M032623 | 阴健等. 中药现代研究与临床应用(1). 北京: 学苑出版社, 1993 |
| F2 | *strychnos nux-vomica* | M033283 | (1) 阴健等. 中药现代研究与临床应用(1). 北京: 学苑出版社, 1993. (2) 杨秀伟等. 中国中药杂志, 1993, 18 (12): 739. (3) Cai BC, et al. 生药学杂志(日), 1990, 44 (1): 42. (4) Cai BC, et al. 生药学杂志(日), 1995, 49 (1): 39. |
| F2 | *strychnos nux-vomica* | M033784 | 江苏新医学院. 中药大辞典. 上海: 上海科学技术出版社, 1977 |
| F2 | *strychnos nux-vomica* | M034020 | Baser, Kemal H. C.; Bisset, Norman G.; Phytochemistry (Elsevier); vol. 21; 6; (1982); p. 1423 - 1430. |
| F2 | *strychnos nux-vomica* | M034338 | Rodriguez,F. et al.; Phytochemistry (Elsevier); vol. 18; (1979); p. 2065. |
| F3 | *Angelica sinensis* | M000029 | (1) 阴健等. 中药现代研究与临床应用(1). 北京: 学苑出版社, 1993. (2) 江苏新医学院. 中药大辞典. 上海: 上海科学技术出版社, 1977. (3) 孙文基等. 天然活性成分简明手册. 北京: 中国医药科技出版社, 1998. (4) 国�抑幸揭┕芾砭帧吨谢静荨繁辔� 中华本草. Vol. 1-30 上海:上海科学技术出版社, 1999. (5) 易杨华. 中国药学杂志, 1990, 25 (10): 585. |
| F3 | *Angelica sinensis* | M000681 | (1) 阴健等. 中药现代研究与临床应用(1). 北京: 学苑出版社, 1993. (2) 张恩娟等. 中国中药杂志, 1993, 18 (1): 37. (3) 孙文基等. 天然活性成分简明手册. 北京: 中国医药科技出版社, 1998. (4) Dou H, et al. JNP, 2002, 65 (12): 1777. |
| F3 | *Angelica sinensis* | M001018 | (1) 阴健等. 中药现代研究与临床应用(1). 北京: 学苑出版社, 1993. (2) 孙文基等. 天然活性成分简明手册. 北京: 中国医药科技出版社, 1998. (3) 国家中医药管理局《中华本草》编委会. 中华本草. Vol. 1-30 上海:上海科学技术出版社, 1999. (4) Hua AN, et al. 南京中医药大学学报, 1990, 6 (3): 179. |
| F3 | *Angelica sinensis* | M001142 | Hon, Po-Ming; Lee, Chi-Ming; Choang, Tai Francis; Chui, Kuk-Ying; Wong, Henry N. C.; Phytochemistry (Elsevier); vol. 29; 4; (1990); p. 1189 - 1191. |
| F3 | *Angelica sinensis* | M001908 | 国家中医药管理局《中华本草》编委会. 中华本草. Vol. 1-30 上海:上海科学技术出版社, 1999 |
| F3 | *Angelica sinensis* | M002088 | 国家中医药管理局《中华本草》编委会. 中华本草. Vol. 1-30 上海:上海科学技术出版社, 1999 |
| F3 | *Angelica sinensis* | M002182 | Retrieved from CNPD |
| F3 | *Angelica sinensis* | M002360 | 阴健等. 中药现代研究与临床应用(1). 北京: 学苑出版社, 1993 |
| F3 | *Angelica sinensis* | M002590 | Deng, Shixin; Chen, Shao-Nong; Yao, Ping; Nikolic, Dejan; Van Breemen, Richard B.; Bolton, Judy L.; Fong, Harry H. S.; Farnsworth, Norman R.; Pauli, Guido F.; Journal of Natural Products; vol. 69; 4; (2006); p. 536 - 541. |
| F3 | *Angelica sinensis* | M002679 | 阴健等. 中药现代研究与临床应用(1). 北京: 学苑出版社, 1993 |
| F3 | *Angelica sinensis* | M003307 | (1) 阴健等. 中药现代研究与临床应用(1). 北京: 学苑出版社, 1993. (2) 孙文基等. 天然活性成分简明手册. 北京: 中国医药科技出版社, 1998. |
| F3 | *Angelica sinensis* | M003359 | Deng, Shixin; Chen, Shao-Nong; Yao, Ping; Nikolic, Dejan; Van Breemen, Richard B.; Bolton, Judy L.; Fong, Harry H. S.; Farnsworth, Norman R.; Pauli, Guido F.; Journal of Natural Products; vol. 69; 4; (2006); p. 536 - 541. |
| F3 | *Angelica sinensis* | M003872 | (1) 阴健等. 中药现代研究与临床应用(1). 北京: 学苑出版社, 1993. (2) 饶高雄等. 中国中药杂志, 1995, 20 (12): 740. (3) 顾志平等. 中国中药杂志, 1997, 22 (1): 40. (4) 孙文基等. 天然活性成分简明手册. 北京: �泄揭┛萍汲霭嫔� 1998. (5) Buckingham J(Executive Editor): et al. Dictionary of Natural Products, Vol 1-7, Chapman & Hall, London, 1994; 1995, Vol 8; 1996, Vol 9; 1997, Vol 10; 1998, Vol 11.. (6) Morikawa T, et al. JNP, 2002, 65 (10): 1468. |
| F3 | *Angelica sinensis* | M003889 | (1) 阴健等. 中药现代研究与临床应用(1). 北京: 学苑出版社, 1993. (2) 季宇彬等. 中药抗肿瘤有效成分药理与应用. 哈尔滨: 黑龙江科学技术出版社, 1995. (3) 宋振玉等. 中草药现代研究. 第2卷. 第28章 九里香. 333-361 北京: 北京医科大学中国协和医科大学联合出版社, 1996. (4) 张印俊等. 药学学报, 1998, 33 (11): 836. (5) 饶高雄等. 中国中药杂志, 1996, 21 (8): 482. (6) 宋蔚等. 中国中药杂志, 1997, 22 (6): 359. (7) 王栋等. 中国中药杂志, 1997, 22 (8): 486. (8) 孙文基等. 天然�钚猿煞旨蛎魇植� 北京: 中国医药科技出版社, 1998. (9) 国家中医药管理局《中华本草》编委会. 中华本草. Vol. 1-30 上海:上海科学技术出版社, 1999. (10) El-Khrisy EAM, et al.Chem. Abstr., 1992, 117, 86802g. (11) Uno T, et al.Chem. Abstr., 1972, 76, 56567b. (12) Chaurasia N, et al.Chem. Abstr., 1986, 104, 85467w. (13) Khvorost PP, et al.Chem. Abstr., 1981, 94, 136170d. (14) Wu PL, et al. Chem Pharm Bull, 2005, 53 (1): 56. (15) Syu WJ, et al. JNP, 2001, 64 (9): 1232. (16) Yun BS, et al. JNP, 2001, 64 (9): 1238. (17) YUAN Z, et al. Chem Pharm Bull, 2002, 50 (1): 73. (18) MORIKAWA T, et al. Chem Pharm Bull, 2003, 51 (1): 62. (19) WANG N-H, et al. Chem Pharm Bull, 2003, 51 (1): 68. (20) WU T-S, et al. Chem [...truncated...] |
| F3 | *Angelica sinensis* | M004163 | Deng, Shixin; Chen, Shao-Nong; Yao, Ping; Nikolic, Dejan; Van Breemen, Richard B.; Bolton, Judy L.; Fong, Harry H. S.; Farnsworth, Norman R.; Pauli, Guido F.; Journal of Natural Products; vol. 69; 4; (2006); p. 536 - 541. |
| F3 | *Angelica sinensis* | M004675 | Deng, Shixin; Chen, Shao-Nong; Yao, Ping; Nikolic, Dejan; Van Breemen, Richard B.; Bolton, Judy L.; Fong, Harry H. S.; Farnsworth, Norman R.; Pauli, Guido F.; Journal of Natural Products; vol. 69; 4; (2006); p. 536 - 541. |
| F3 | *Angelica sinensis* | M005571 | (1) 阴健等. 中药现代研究与临床应用(1). 北京: 学苑出版社, 1993. (2) 毛士龙等. 药学学报, 1996, 31 (1): 118. (3) 顾莹等. 药学学报, 1997, 32 (1): 59. (4) 封士兰等. 中国中药杂志, 1994, 19 (10): 611. (5) 孙文基等. 天然活性成分简明手册. 北京: 中国医药科技出版社, 1998. (6) 国家中医药管理局《中华本草》编委会. 中华本草. Vol. 1-30 上海:上海科学技术出版社, 1999. (7) Wu PL, et al. Chem Pharm Bull, 2005, 53 (1): 56. (8) Wu TS, et al. JNP, 2001, 64 (1): 71. (9) Lin LC, et al. JNP, 2001, 64 (5): 674. (10) Wu TS, et al. JNP, 2001, 64 (8): 1040. (11) Jiang RW, et al. JNP, 2001, 64 (10): 1266. (12) Block S, et al. Phytochemistry, 2004, 65, 1165. (13) WU T-S, et al. Chem Pharm Bull, 2003, 51 (8): 948. (14) KIEM PV, et al. Chem Pharm Bull, 2005, 53 (4): 428. (15) LIM J-C, et al. Chem Pharm Bull, 2005, 53 (5): 561. (16) CHAN Y-Y, et al. Chem Pharm Bull, 2005, 53 (7): 836. (17) LEU Y-L, et al. Chem Pharm Bull, 2005, 53 (7): 853. (18) SUKSAMRARN A, et al. Chem Pharm Bull, 2005, 53 (10): 1327. (19) Liou MJ, et al. JNP, 2002, 65 (9): 1283. (20) Pan WB, et al. JNP, 2003, 66 (1): 161. (21 [...truncated...] |
| F3 | *Angelica sinensis* | M005974 | (1) 阴健等. 中药现代研究与临床应用(1). 北京: 学苑出版社, 1993. (2) 国家中医药管理局《中华本草》编委会. 中华本草. Vol. 1-30 上海:上海科学技术出版社, 1999. |
| F3 | *Angelica sinensis* | M006085 | Deng, Shixin; Chen, Shao-Nong; Yao, Ping; Nikolic, Dejan; Van Breemen, Richard B.; Bolton, Judy L.; Fong, Harry H. S.; Farnsworth, Norman R.; Pauli, Guido F.; Journal of Natural Products; vol. 69; 4; (2006); p. 536 - 541. |
| F3 | *Angelica sinensis* | M006222 | Deng, Shixin; Chen, Shao-Nong; Yao, Ping; Nikolic, Dejan; Van Breemen, Richard B.; Bolton, Judy L.; Fong, Harry H. S.; Farnsworth, Norman R.; Pauli, Guido F.; Journal of Natural Products; vol. 69; 4; (2006); p. 536 - 541. |
| F3 | *Angelica sinensis* | M006375 | (1) 阴健等. 中药现代研究与临床应用(1). 北京: 学苑出版社, 1993. (2) 江苏新医学院. 中药大辞典. 上海: 上海科学技术出版社, 1977. (3) 孙文基等. 天然活性成分简明手册. 北京: 中国医药科技出版社, 1998. (4) 国�抑幸揭┕芾砭帧吨谢静荨繁辔� 中华本草. Vol. 1-30 上海:上海科学技术出版社, 1999. (5) 刈米达夫著, 杨本文译. 植物化学, 科学出版社, 北京, 1985. |
| F3 | *Angelica sinensis* | M006850 | (1) 阴健等. 中药现代研究与临床应用(1). 北京: 学苑出版社, 1993. (2) 李其生等. 中国中药杂志, 1993, 18 (8): 486. (3) 肖永庆等. 中国中药杂志, 1995, 20 (5): 294. (4) 肖永庆等. 中国中药杂志, 1995, 20 (7): 423. (5) 饶高雄等. 中国中药杂志, 1995, 20 (12): 740. (6) 顾志平等. 中国中药杂志, 1997, 22 (1): 40. (7) 韦松等. 中国中药杂志, 1997, 22 (5): 293. (8) 宋蔚等. 中国中药杂志, 1997, 22 (6): 359. (9) 梁培瑜等. 中国中药杂志, 1998, 23 (1): 39. (10) 王海燕等. 中国中药杂志, 1998, 23 (3): 167. (11) 国家中医药管理局《中华本草》编委会. 中华本草. Vol. 1-30 上海:上海科学技术出版社, 1999. (12) Buckingham J(Executive Editor): et al. Dictionary of Natural Products, Vol 1-7, Chapman & Hall, London, 1994; 1995, Vol 8; 1996, Vol 9; 1997, Vol 10; 1998, Vol 11.. (13) Sautour M, et al. Chem Pharm Bull, 2004, 52 (10): 1235. (14) BEGUM S, et al. Chem Pharm Bull, 2003, 51 (2): 134. (15) Calixto JB, et al. Planta Med, 2003, 69 (11): 973. (16) Lin WY, et al. Planta Med, 2003, 69, 757. |
| F3 | *Angelica sinensis* | M006919 | (1) 阴健等. 中药现代研究与临床应用(1). 北京: 学苑出版社, 1993. (2) 季宇彬等. 中药抗肿瘤有效成分药理与应用. 哈尔滨: 黑龙江科学技术出版社, 1995. (3) 李彤梅等. 药学学报, 1998, 33 (8): 591. (4) 傅宏征等. 中国药学杂志, 1998, 33 (3): 140. (5) 周燕生等. 中国中药杂志, 1994, 19 (3): 162. (6) 徐丽珍等. 中国中药杂志, 1994, 19 (11): 675. (7) 陈妙华等. 中国中药杂志, 1993, 18 (7): 424. (8) 石磊等. 中国中药杂志, 1997, 22 (12): 743. (9) 徐丽萍等. 中国中药杂志, 1998, 23 (5): 293. (10) 孙文基等. 天然活性成分简明手册. 北京: 中国医药科技出版社, 1998. (11) 国家中医药管理局《中华本草》编委会. 中华本草. Vol. 1-30 上海:上海科学技术出版社, 1999. (12) Zheng Y, et al. JNP, 2004, 67 (9): 1617. (13) 欧明等. 简明中药成分手册. 北京: 中国医药科技出版社, 2003. |
| F3 | *Angelica sinensis* | M007255 | (1) 阴健等. 中药现代研究与临床应用(1). 北京: 学苑出版社, 1993. (2) 陈若云等. 药学学报, 1995, 30 (7): 526. (3) 国家中医药管理局《中华本草》编委会. 中华本草. Vol. 1-30 上海:上海科学技术出版社, 1999. (4) Calixto JB, et al. Planta Med, 2003, 69 (11): 973. |
| F3 | *Angelica sinensis* | M007385 | 阴健等. 中药现代研究与临床应用(1). 北京: 学苑出版社, 1993 |
| F3 | *Angelica sinensis* | M007418 | 国家中医药管理局《中华本草》编委会. 中华本草. Vol. 1-30 上海:上海科学技术出版社, 1999 |
| F3 | *Angelica sinensis* | M007760 | 黄伟晖等. 药学学报, 2003, 38 (9): 680 |
| F3 | *Angelica sinensis* | M008893 | 阴健等. 中药现代研究与临床应用(1). 北京: 学苑出版社, 1993 |
| F3 | *Angelica sinensis* | M008923 | Deng, Shixin; Chen, Shao-Nong; Yao, Ping; Nikolic, Dejan; Van Breemen, Richard B.; Bolton, Judy L.; Fong, Harry H. S.; Farnsworth, Norman R.; Pauli, Guido F.; Journal of Natural Products; vol. 69; 4; (2006); p. 536 - 541. |
| F3 | *Angelica sinensis* | M009104 | (1) 阴健等. 中药现代研究与临床应用(1). 北京: 学苑出版社, 1993. (2) 国家中医药管理局《中华本草》编委会. 中华本草. Vol. 1-30 上海:上海科学技术出版社, 1999. (3) 吴知行等. 中国药科大学学报, 1994, 25 (4): 202. |
| F3 | *Angelica sinensis* | M009554 | (1) 阴健等. 中药现代研究与临床应用(1). 北京: 学苑出版社, 1993. (2) 张卫东等. 药学学报, 1992, 27 (9): 670. (3) 孙文基等. 天然活性成分简明手册. 北京: 中国医药科技出版社, 1998. (4) 国家中医药管理局《中华本草》编委会. 中华本草. Vol. 1-30 上海:上海科学技术出版社, 1999. (5) LI C-Y, et al. Chem Pharm Bull, 2002, 50 (10): 1305. (6) Li CY, et al. JNP, 2002, 65 (10): 1452. |
| F3 | *Angelica sinensis* | M009761 | (1) 李其生等. 中国中药杂志, 1993, 18 (8): 486. (2) 国家中医药管理局《中华本草》编委会. 中华本草. Vol. 1-30 上海:上海科学技术出版社, 1999. (3) 于澍仁等. 药学学报, 1984, 19 (8): 566. (4) Ozaki Y, et al. 药学杂志(日), 1989, 109 (6): 402. (5) Chem. Abstr., 1989, 111, P239574n. (6) Buckingham J(Executive Editor): et al. Dictionary of Natural Products, Vol 1-7, Chapman & Hall, London, 1994; 1995, Vol 8; 1996, Vol 9; 1997, Vol 10; 1998, Vol 11.. |
| F3 | *Angelica sinensis* | M009947 | Deng, Shixin; Chen, Shao-Nong; Yao, Ping; Nikolic, Dejan; Van Breemen, Richard B.; Bolton, Judy L.; Fong, Harry H. S.; Farnsworth, Norman R.; Pauli, Guido F.; Journal of Natural Products; vol. 69; 4; (2006); p. 536 - 541. |
| F3 | *Angelica sinensis* | M010181 | 阴健等. 中药现代研究与临床应用(1). 北京: 学苑出版社, 1993 |
| F3 | *Angelica sinensis* | M010230 | 阴健等. 中药现代研究与临床应用(1). 北京: 学苑出版社, 1993 |
| F3 | *Angelica sinensis* | M010372 | (1) 阴健等. 中药现代研究与临床应用(1). 北京: 学苑出版社, 1993. (2) 宋振玉等. 中草药现代研究. 第3卷. 第33章 沉香. 1-21 北京: 北京医科大学中国协和医科大学联合出版社, 1997. (3) AWALE S, et al. Chem Pharm Bull, 2005, 53 (6): 710. (4) LEU Y-L, et al. Chem Pharm Bull, 2005, 53 (7): 853. |
| F3 | *Angelica sinensis* | M010398 | (1) 阴健等. 中药现代研究与临床应用(1). 北京: 学苑出版社, 1993. (2) 孙文基等. 天然活性成分简明手册. 北京: 中国医药科技出版社, 1998. (3) 国家中医药管理局《中华本草》编委会. 中华本草. Vol. 1-30 上海:上海科学技术出版社, 1999. (4) Buckingham J(Executive Editor): et al. Dictionary of Natural Products, Vol 1-7, Chapman & Hall, London, 1994; 1995, Vol 8; 1996, Vol 9; 1997, Vol 10; 1998, Vol 11.. |
| F3 | *Angelica sinensis* | M010708 | (1) 阴健等. 中药现代研究与临床应用(1). 北京: 学苑出版社, 1993. (2) 毛士龙等. 药学学报, 1996, 31 (1): 118. (3) Yoshikawa M, et al. JNP, 2003, 66 (7): 922. (4) Carcache-Blanco EJ, et al. JNP, 2003, 67 (1): 126. (5) Faizi S, et al. Planta Med, 2003, 69, 350. |
| F3 | *Angelica sinensis* | M011000 | (1) 阴健等. 中药现代研究与临床应用(1). 北京: 学苑出版社, 1993. (2) 国家中医药管理局《中华本草》编委会. 中华本草. Vol. 1-30 上海:上海科学技术出版社, 1999. |
| F3 | *Angelica sinensis* | M011318 | 黄伟晖等. 药学学报, 2003, 38 (9): 680 |
| F3 | *Angelica sinensis* | M012564 | (1) 阴健等. 中药现代研究与临床应用(1). 北京: 学苑出版社, 1993. (2) 孙文基等. 天然活性成分简明手册. 北京: 中国医药科技出版社, 1998. (3) 国家中医药管理局《中华本草》编委会. 中华本草. Vol. 1-30 上海:上海科学技术出版社, 1999. |
| F3 | *Angelica sinensis* | M012697 | 阴健等. 中药现代研究与临床应用(1). 北京: 学苑出版社, 1993 |
| F3 | *Angelica sinensis* | M012774 | 阴健等. 中药现代研究与临床应用(1). 北京: 学苑出版社, 1993 |
| F3 | *Angelica sinensis* | M012885 | Deng, Shixin; Chen, Shao-Nong; Yao, Ping; Nikolic, Dejan; Van Breemen, Richard B.; Bolton, Judy L.; Fong, Harry H. S.; Farnsworth, Norman R.; Pauli, Guido F.; Journal of Natural Products; vol. 69; 4; (2006); p. 536 - 541. |
| F3 | *Angelica sinensis* | M013716 | (1) 阴健等. 中药现代研究与临床应用(1). 北京: 学苑出版社, 1993. (2) 孙文基等. 天然活性成分简明手册. 北京: 中国医药科技出版社, 1998. |
| F3 | *Angelica sinensis* | M013833 | Buckingham J(Executive Editor): et al. Dictionary of Natural Products, Vol 1-7, Chapman & Hall, London, 1994; 1995, Vol 8; 1996, Vol 9; 1997, Vol 10; 1998, Vol 11. |
| F3 | *Angelica sinensis* | M014504 | 阴健等. 中药现代研究与临床应用(1). 北京: 学苑出版社, 1993 |
| F3 | *Angelica sinensis* | M014560 | 阴健等. 中药现代研究与临床应用(1). 北京: 学苑出版社, 1993 |
| F3 | *Angelica sinensis* | M014760 | (1) 汪纪武等. 植物药有效成分手册. 北京: 人民卫生出版社, 1986. (2) 阴健等. 中药现代研究与临床应用(1). 北京: 学苑出版社, 1993. (3) Shin S, et al. Planta Med, 2004, 70, 1090. |
| F3 | *Angelica sinensis* | M015010 | 阴健等. 中药现代研究与临床应用(1). 北京: 学苑出版社, 1993 |
| F3 | *Angelica sinensis* | M015284 | 国家中医药管理局《中华本草》编委会. 中华本草. Vol. 1-30 上海:上海科学技术出版社, 1999 |
| F3 | *Angelica sinensis* | M015310 | 阴健等. 中药现代研究与临床应用(1). 北京: 学苑出版社, 1993 |
| F3 | *Angelica sinensis* | M015874 | (1) 阴健等. 中药现代研究与临床应用(1). 北京: 学苑出版社, 1993. (2) 国家中医药管理局《中华本草》编委会. 中华本草. Vol. 1-30 上海:上海科学技术出版社, 1999. |
| F3 | *Angelica sinensis* | M016844 | Yang, Nian-Yun; Zhou, Gui-Sheng; Tang, Yu-Ping; Yan, Hui; Guo, Sheng; Liu, Pei; Duan, Jin-Ao; Song, Bing-Sheng; He, Zi-Qing; Fitoterapia; vol. 82; 4; (2011); p. 692 - 695. |
| F3 | *Angelica sinensis* | M016845 | Yang, Nian-Yun; Zhou, Gui-Sheng; Tang, Yu-Ping; Yan, Hui; Guo, Sheng; Liu, Pei; Duan, Jin-Ao; Song, Bing-Sheng; He, Zi-Qing; Fitoterapia; vol. 82; 4; (2011); p. 692 - 695. |
| F3 | *Angelica sinensis* | M019624 | Short Survey; Yang, Nian-Yun; Jiang, Shu; Shang, Er-Xin; Tang, Yu-Ping; Duan, Jin-Ao; Journal of Chemical Research; vol. 36; 11; (2012); p. 647 - 647. |
| F3 | *Angelica sinensis* | M019865 | Trivedi et al.; Cesko-Slovenska Farmacie; vol. 15; (1966); p. 206,209. |
| F3 | *Angelica sinensis* | M020237 | National Dong Hwa University; Chiou, Tzyy-Wen; Harn, Horng-Jyh; Lin, Shinn-Zong; EP2606883; A1; (2013). |
| F3 | *Angelica sinensis* | M020290 | Xie, Jing-Jing; Lu, Jia; Qian, Zheng-Ming; Yu; Duan, Jin-Ao; Li, Shao-Ping; Molecules; vol. 14; 1; (2009); p. 555 - 565. |
| F3 | *Angelica sinensis* | M021266 | Li, Xing-Nuo; Chen, Yuan-Yi; Cheng, Dong-Ping; Tong, Sheng-Qiang; Yan, Ji-Zhong; Qu, Hai-Bin; Natural Product Research; vol. 26; 19; (2012); p. 1782 - 1786,5.; Li, Xing-Nuo; Chen, Yuan-Yi; Cheng, Dong-Ping; Tong, Sheng-Qiang; Qu, Hai-Bin; Yan, Ji-Zhong; Natural Product Research; vol. 26; 19; (2012); p. 1782 - 1786. |
| F3 | *Angelica sinensis* | M022026 | 江苏新医学院. 中药大辞典. 上海: 上海科学技术出版社, 1977 |
| F3 | *Angelica sinensis* | M022322 | 阴健等. 中药现代研究与临床应用(1). 北京: 学苑出版社, 1993 |
| F3 | *Angelica sinensis* | M023044 | (1) 阴健等. 中药现代研究与临床应用(1). 北京: 学苑出版社, 1993. (2) 江苏新医学院. 中药大辞典. 上海: 上海科学技术出版社, 1977. (3) 国家中医药管理局《中华本草》编委会. 中华本草. Vol. 1-30 上海:上海科学技术出版社, 1999. |
| F3 | *Angelica sinensis* | M023048 | 阴健等. 中药现代研究与临床应用(1). 北京: 学苑出版社, 1993 |
| F3 | *Angelica sinensis* | M024367 | Deng, Shixin; Chen, Shao-Nong; Yao, Ping; Nikolic, Dejan; Van Breemen, Richard B.; Bolton, Judy L.; Fong, Harry H. S.; Farnsworth, Norman R.; Pauli, Guido F.; Journal of Natural Products; vol. 69; 4; (2006); p. 536 - 541. |
| F3 | *Angelica sinensis* | M024426 | 阴健等. 中药现代研究与临床应用(1). 北京: 学苑出版社, 1993 |
| F3 | *Angelica sinensis* | M024856 | (1) 江苏新医学院. 中药大辞典. 上海: 上海科学技术出版社, 1977. (2) 国家中医药管理局《中华本草》编委会. 中华本草. Vol. 1-30 上海:上海科学技术出版社, 1999. |
| F3 | *Angelica sinensis* | M025149 | Deng, Shixin; Chen, Shao-Nong; Yao, Ping; Nikolic, Dejan; Van Breemen, Richard B.; Bolton, Judy L.; Fong, Harry H. S.; Farnsworth, Norman R.; Pauli, Guido F.; Journal of Natural Products; vol. 69; 4; (2006); p. 536 - 541. |
| F3 | *Angelica sinensis* | M025258 | (1) 阴健等. 中药现代研究与临床应用(1). 北京: 学苑出版社, 1993. (2) 江苏新医学院. 中药大辞典. 上海: 上海科学技术出版社, 1977. (3) 国家中医药管理局《中华本草》编委会. 中华本草. Vol. 1-30 上海:上海科学技术出版社, 1999. |
| F3 | *Angelica sinensis* | M025330 | Deng, Shixin; Chen, Shao-Nong; Yao, Ping; Nikolic, Dejan; Van Breemen, Richard B.; Bolton, Judy L.; Fong, Harry H. S.; Farnsworth, Norman R.; Pauli, Guido F.; Journal of Natural Products; vol. 69; 4; (2006); p. 536 - 541. |
| F3 | *Angelica sinensis* | M025438 | 苏东敏等. 药学学报, 2005, 40 (2): 141 |
| F3 | *Angelica sinensis* | M025532 | Deng, Shixin; Chen, Shao-Nong; Yao, Ping; Nikolic, Dejan; Van Breemen, Richard B.; Bolton, Judy L.; Fong, Harry H. S.; Farnsworth, Norman R.; Pauli, Guido F.; Journal of Natural Products; vol. 69; 4; (2006); p. 536 - 541. |
| F3 | *Angelica sinensis* | M025882 | Deng, Shixin; Chen, Shao-Nong; Yao, Ping; Nikolic, Dejan; Van Breemen, Richard B.; Bolton, Judy L.; Fong, Harry H. S.; Farnsworth, Norman R.; Pauli, Guido F.; Journal of Natural Products; vol. 69; 4; (2006); p. 536 - 541. |
| F3 | *Angelica sinensis* | M026459 | Deng, Shixin; Chen, Shao-Nong; Yao, Ping; Nikolic, Dejan; Van Breemen, Richard B.; Bolton, Judy L.; Fong, Harry H. S.; Farnsworth, Norman R.; Pauli, Guido F.; Journal of Natural Products; vol. 69; 4; (2006); p. 536 - 541. |
| F3 | *Angelica sinensis* | M026949 | Deng, Shixin; Chen, Shao-Nong; Yao, Ping; Nikolic, Dejan; Van Breemen, Richard B.; Bolton, Judy L.; Fong, Harry H. S.; Farnsworth, Norman R.; Pauli, Guido F.; Journal of Natural Products; vol. 69; 4; (2006); p. 536 - 541. |
| F3 | *Angelica sinensis* | M027757 | 阴健等. 中药现代研究与临床应用(1). 北京: 学苑出版社, 1993 |
| F3 | *Angelica sinensis* | M027787 | Deng, Shixin; Chen, Shao-Nong; Yao, Ping; Nikolic, Dejan; Van Breemen, Richard B.; Bolton, Judy L.; Fong, Harry H. S.; Farnsworth, Norman R.; Pauli, Guido F.; Journal of Natural Products; vol. 69; 4; (2006); p. 536 - 541. |
| F3 | *Angelica sinensis* | M028027 | Deng, Shixin; Chen, Shao-Nong; Yao, Ping; Nikolic, Dejan; Van Breemen, Richard B.; Bolton, Judy L.; Fong, Harry H. S.; Farnsworth, Norman R.; Pauli, Guido F.; Journal of Natural Products; vol. 69; 4; (2006); p. 536 - 541. |
| F3 | *Angelica sinensis* | M028061 | (1) 江苏新医学院. 中药大辞典. 上海: 上海科学技术出版社, 1977. (2) 宋振玉等. 中草药现代研究. 第2卷. 第17章 当归. 1-51 北京: 北京医科大学中国协和医科大学联合出版社, 1996. (3) 宋振玉等. 中草药现代研究. ��卷. 第18章 旱芹. 52-70 北京: 北京医科大学中国协和医科大学联合出版社, 1996. |
| F3 | *Angelica sinensis* | M028851 | 阴健等. 中药现代研究与临床应用(1). 北京: 学苑出版社, 1993 |
| F3 | *Angelica sinensis* | M029284 | (1) 阴健等. 中药现代研究与临床应用(1). 北京: 学苑出版社, 1993. (2) 国家中医药管理局《中华本草》编委会. 中华本草. Vol. 1-30 上海:上海科学技术出版社, 1999. |
| F3 | *Angelica sinensis* | M029558 | 阴健等. 中药现代研究与临床应用(1). 北京: 学苑出版社, 1993 |
| F3 | *Angelica sinensis* | M029937 | Deng, Shixin; Chen, Shao-Nong; Yao, Ping; Nikolic, Dejan; Van Breemen, Richard B.; Bolton, Judy L.; Fong, Harry H. S.; Farnsworth, Norman R.; Pauli, Guido F.; Journal of Natural Products; vol. 69; 4; (2006); p. 536 - 541. |
| F3 | *Angelica sinensis* | M031075 | (1) 阴健等. 中药现代研究与临床应用(1). 北京: 学苑出版社, 1993. (2) 孙文基等. 天然活性成分简明手册. 北京: 中国医药科技出版社, 1998. (3) 国家中医药管理局《中华本草》编委会. 中华本草. Vol. 1-30 上海:上海科学技术出版社, 1999. (4) Buckingham J(Executive Editor): et al. Dictionary of Natural Products, Vol 1-7, Chapman & Hall, London, 1994; 1995, Vol 8; 1996, Vol 9; 1997, Vol 10; 1998, Vol 11.. (5) CHAN Y-Y, et al. Chem Pharm Bull, 2005, 53 (7): 836. |
| F3 | *Angelica sinensis* | M031093 | 阴健等. 中药现代研究与临床应用(1). 北京: 学苑出版社, 1993 |
| F3 | *Angelica sinensis* | M031155 | Li, Xing-Nuo; Chen, Yuan-Yi; Cheng, Dong-Ping; Tong, Sheng-Qiang; Yan, Ji-Zhong; Qu, Hai-Bin; Natural Product Research; vol. 26; 19; (2012); p. 1782 - 1786,5.; Li, Xing-Nuo; Chen, Yuan-Yi; Cheng, Dong-Ping; Tong, Sheng-Qiang; Qu, Hai-Bin; Yan, Ji-Zhong; Natural Product Research; vol. 26; 19; (2012); p. 1782 - 1786. |
| F3 | *Angelica sinensis* | M032127 | (1) 阴健等. 中药现代研究与临床应用(1). 北京: 学苑出版社, 1993. (2) 国家中医药管理局《中华本草》编委会. 中华本草. Vol. 1-30 上海:上海科学技术出版社, 1999. |
| F3 | *Angelica sinensis* | M032515 | Deng, Shixin; Chen, Shao-Nong; Yao, Ping; Nikolic, Dejan; Van Breemen, Richard B.; Bolton, Judy L.; Fong, Harry H. S.; Farnsworth, Norman R.; Pauli, Guido F.; Journal of Natural Products; vol. 69; 4; (2006); p. 536 - 541. |
| F3 | *Angelica sinensis* | M033251 | 阴健等. 中药现代研究与临床应用(1). 北京: 学苑出版社, 1993 |
| F3 | *Angelica sinensis* | M033995 | 阴健等. 中药现代研究与临床应用(1). 北京: 学苑出版社, 1993 |
| F3 | *Arctium lappa* | M000093 | 江苏新医学院. 中药大辞典. 上海: 上海科学技术出版社, 1977 |
| F3 | *Arctium lappa* | M000170 | Washino, Tsutomu; Yoshikura, Masahiro; Obata, Shigeo; Agricultural and Biological Chemistry; vol. 50; 1; (1986); p. 263 - 270. |
| F3 | *Arctium lappa* | M000486 | Ichihara; Oda; Numata; Sakamura; Tetrahedron Letters; vol. No. 44; (1976); p. 3961 - 3964. |
| F3 | *Arctium lappa* | M000604 | Washino, Tsutomu; Kobayashi, Hidetoshi; Ikawa, Yoshitomi; Agricultural and Biological Chemistry; vol. 51; 6; (1987); p. 1475 - 1480. |
| F3 | *Arctium lappa* | M001859 | Akihisa, Toshihiro; Yasukawa, Ken; Oinuma, Hirotoshi; Kasahara, Yoshimasa; Yamanouchi, Sakae; Takido, Michio; Kumaki, Kunio; Tamura, Toshitake; Phytochemistry; vol. 43; 6; (1996); p. 1255 - 1260. |
| F3 | *Arctium lappa* | M002031 | Han, Byung Hoon; Kang, Young Hwa; Yang, Hyun Ok; Park, Man Ki; Phytochemistry (Elsevier); vol. 37; 4; (1994); p. 1161 - 1164. |
| F3 | *Arctium lappa* | M002934 | Umehara; Sugawa; Kuroyanagi; Ueno; Taki; Chemical and Pharmaceutical Bulletin; vol. 41; 10; (1993); p. 1774 - 1779. |
| F3 | *Arctium lappa* | M002966 | Umehara; Nakamura; Miyase; Kuroyanagi; Ueno; Chemical and Pharmaceutical Bulletin; vol. 44; 12; (1996); p. 2300 - 2304. |
| F3 | *Arctium lappa* | M003009 | Akihisa, Toshihiro; Yasukawa, Ken; Oinuma, Hirotoshi; Kasahara, Yoshimasa; Yamanouchi, Sakae; Takido, Michio; Kumaki, Kunio; Tamura, Toshitake; Phytochemistry; vol. 43; 6; (1996); p. 1255 - 1260. |
| F3 | *Arctium lappa* | M003033 | 江苏新医学院. 中药大辞典. 上海: 上海科学技术出版社, 1977 |
| F3 | *Arctium lappa* | M003214 | Akihisa, Toshihiro; Yasukawa, Ken; Oinuma, Hirotoshi; Kasahara, Yoshimasa; Yamanouchi, Sakae; Takido, Michio; Kumaki, Kunio; Tamura, Toshitake; Phytochemistry; vol. 43; 6; (1996); p. 1255 - 1260. |
| F3 | *Arctium lappa* | M003322 | Umehara; Sugawa; Kuroyanagi; Ueno; Taki; Chemical and Pharmaceutical Bulletin; vol. 41; 10; (1993); p. 1774 - 1779. |
| F3 | *Arctium lappa* | M003829 | 国家中医药管理局《中华本草》编委会. 中华本草. Vol. 1-30 上海:上海科学技术出版社, 1999 |
| F3 | *Arctium lappa* | M003874 | (1) Buttery RG, et al.Chem. Abstr., 1990, 113, 210415s. (2) Ronald GB, et al. J Agric Food Chem, 1990, 36, 1245. |
| F3 | *Arctium lappa* | M003891 | (1) 国家中医药管理局《中华本草》编委会. 中华本草. Vol. 1-30 上海:上海科学技术出版社, 1999. (2) Buckingham J(Executive Editor): et al. Dictionary of Natural Products, Vol 1-7, Chapman & Hall, London, 1994; 1995, Vol 8; 1996, Vol 9; 1997, Vol 10; 1998, Vol 11.. |
| F3 | *Arctium lappa* | M003984 | Retrieved from CNPD |
| F3 | *Arctium lappa* | M004248 | Umehara; Sugawa; Kuroyanagi; Ueno; Taki; Chemical and Pharmaceutical Bulletin; vol. 41; 10; (1993); p. 1774 - 1779. |
| F3 | *Arctium lappa* | M004274 | Retrieved from CNPD |
| F3 | *Arctium lappa* | M005876 | Maruta, Yoshihiko; Kawabata, Jun; Niki, Ryoya; Journal of Agricultural and Food Chemistry; vol. 43; 10; (1995); p. 2592 - 2595. |
| F3 | *Arctium lappa* | M005897 | Maruta, Yoshihiko; Kawabata, Jun; Niki, Ryoya; Journal of Agricultural and Food Chemistry; vol. 43; 10; (1995); p. 2592 - 2595. |
| F3 | *Arctium lappa* | M005960 | 江苏新医学院. 中药大辞典. 上海: 上海科学技术出版社, 1977 |
| F3 | *Arctium lappa* | M006414 | Umehara; Sugawa; Kuroyanagi; Ueno; Taki; Chemical and Pharmaceutical Bulletin; vol. 41; 10; (1993); p. 1774 - 1779. |
| F3 | *Arctium lappa* | M007025 | Naya,K. et al.; Chemistry Letters; (1972); p. 235 - 236. |
| F3 | *Arctium lappa* | M007538 | Umehara; Nakamura; Miyase; Kuroyanagi; Ueno; Chemical and Pharmaceutical Bulletin; vol. 44; 12; (1996); p. 2300 - 2304. |
| F3 | *Arctium lappa* | M007844 | 国家中医药管理局《中华本草》编委会. 中华本草. Vol. 1-30 上海:上海科学技术出版社, 1999 |
| F3 | *Arctium lappa* | M008374 | Umehara; Nakamura; Miyase; Kuroyanagi; Ueno; Chemical and Pharmaceutical Bulletin; vol. 44; 12; (1996); p. 2300 - 2304. |
| F3 | *Arctium lappa* | M008510 | Washino, Tsutomu; Yoshikura, Masahiro; Obata, Shigeo; Agricultural and Biological Chemistry; vol. 50; 1; (1986); p. 263 - 270. |
| F3 | *Arctium lappa* | M008793 | 王海燕等. 药学学报, 1993, 28 (12): 911 |
| F3 | *Arctium lappa* | M009062 | Washino, Tsutomu; Yoshikura, Masahiro; Obata, Shigeo; Agricultural and Biological Chemistry; vol. 50; 1; (1986); p. 263 - 270. |
| F3 | *Arctium lappa* | M009440 | Shinoda; Kawagoye; Yakugaku Zasshi; vol. 49; (1929); p. 565,567; dtsch. Ref. S. 94; Chem. Zentralbl.; vol. 100; II; (1929); p. 1546. |
| F3 | *Arctium lappa* | M009870 | Umezawa, Toshiaki; Shimada, Mikio; Bioscience, Biotechnology and Biochemistry; vol. 60; 4; (1996); p. 736 - 737. |
| F3 | *Arctium lappa* | M009993 | Han, Byung Hoon; Kang, Young Hwa; Yang, Hyun Ok; Park, Man Ki; Phytochemistry (Elsevier); vol. 37; 4; (1994); p. 1161 - 1164. |
| F3 | *Arctium lappa* | M010212 | Retrieved from CNPD |
| F3 | *Arctium lappa* | M010294 | Akihisa, Toshihiro; Yasukawa, Ken; Oinuma, Hirotoshi; Kasahara, Yoshimasa; Yamanouchi, Sakae; Takido, Michio; Kumaki, Kunio; Tamura, Toshitake; Phytochemistry; vol. 43; 6; (1996); p. 1255 - 1260. |
| F3 | *Arctium lappa* | M010340 | Yamada et al.; Phytochemistry (Elsevier); vol. 14; (1975); p. 582. |
| F3 | *Arctium lappa* | M010701 | Shinoda; Kawagoye; Yakugaku Zasshi; vol. 49; (1929); p. 94; Chem. Zentralbl.; vol. 100; II; (1929); p. 1547. |
| F3 | *Arctium lappa* | M010818 | 江苏新医学院. 中药大辞典. 上海: 上海科学技术出版社, 1977 |
| F3 | *Arctium lappa* | M010974 | Maruta, Yoshihiko; Kawabata, Jun; Niki, Ryoya; Journal of Agricultural and Food Chemistry; vol. 43; 10; (1995); p. 2592 - 2595. |
| F3 | *Arctium lappa* | M011256 | Takasugi, Mitsuo; Kawashima, Shinji; Katsui, Nobukatsu; Shirata, Akira; Phytochemistry (Elsevier); vol. 26; 11; (1987); p. 2957 - 2958. |
| F3 | *Arctium lappa* | M011744 | Umehara; Sugawa; Kuroyanagi; Ueno; Taki; Chemical and Pharmaceutical Bulletin; vol. 41; 10; (1993); p. 1774 - 1779. |
| F3 | *Arctium lappa* | M011879 | Takasugi, Mitsuo; Kawashima, Shinji; Katsui, Nobukatsu; Shirata, Akira; Phytochemistry (Elsevier); vol. 26; 11; (1987); p. 2957 - 2958. |
| F3 | *Arctium lappa* | M012315 | 江苏新医学院. 中药大辞典. 上海: 上海科学技术出版社, 1977 |
| F3 | *Arctium lappa* | M012665 | Akihisa, Toshihiro; Yasukawa, Ken; Oinuma, Hirotoshi; Kasahara, Yoshimasa; Yamanouchi, Sakae; Takido, Michio; Kumaki, Kunio; Tamura, Toshitake; Phytochemistry; vol. 43; 6; (1996); p. 1255 - 1260. |
| F3 | *Arctium lappa* | M013472 | Washino, Tsutomu; Yoshikura, Masahiro; Obata, Shigeo; Agricultural and Biological Chemistry; vol. 50; 1; (1986); p. 263 - 270. |
| F3 | *Arctium lappa* | M013903 | 国家中医药管理局《中华本草》编委会. 中华本草. Vol. 1-30 上海:上海科学技术出版社, 1999 |
| F3 | *Arctium lappa* | M014151 | Ichihara et al.; Tetrahedron Letters; (1978); p. 3035. |
| F3 | *Arctium lappa* | M014365 | Umehara; Sugawa; Kuroyanagi; Ueno; Taki; Chemical and Pharmaceutical Bulletin; vol. 41; 10; (1993); p. 1774 - 1779. |
| F3 | *Arctium lappa* | M014410 | 江苏新医学院. 中药大辞典. 上海: 上海科学技术出版社, 1977 |
| F3 | *Arctium lappa* | M015768 | Umehara; Sugawa; Kuroyanagi; Ueno; Taki; Chemical and Pharmaceutical Bulletin; vol. 41; 10; (1993); p. 1774 - 1779. |
| F3 | *Arctium lappa* | M017663 | Retrieved from CNPD |
| F3 | *Arctium lappa* | M017703 | Retrieved from CNPD |
| F3 | *Arctium lappa* | M017704 | Retrieved from CNPD |
| F3 | *Arctium lappa* | M017874 | Yang, Ya-Nan; Zhang, Fan; Feng, Zi-Ming; Jiang, Jian-Shuang; Zhang, Pei-Cheng; Journal of Asian Natural Products Research; vol. 14; 10; (2012); p. 981 - 985,5.; Yang, Ya-Nan; Zhang, Fan; Feng, Zi-Ming; Jiang, Jian-Shuang; Zhang, Pei-Cheng; Journal of Asian Natural Products Research; vol. 14; 10; (2012); p. 981 - 985. |
| F3 | *Arctium lappa* | M018322 | Yang, Ya-Nan; Zhang, Fan; Feng, Zi-Ming; Jiang, Jian-Shuang; Zhang, Pei-Cheng; Journal of Asian Natural Products Research; vol. 14; 10; (2012); p. 981 - 985,5.; Yang, Ya-Nan; Zhang, Fan; Feng, Zi-Ming; Jiang, Jian-Shuang; Zhang, Pei-Cheng; Journal of Asian Natural Products Research; vol. 14; 10; (2012); p. 981 - 985. |
| F3 | *Arctium lappa* | M018743 | Yang, Ya-Nan; Zhang, Fan; Feng, Zi-Ming; Jiang, Jian-Shuang; Zhang, Pei-Cheng; Journal of Asian Natural Products Research; vol. 14; 10; (2012); p. 981 - 985,5.; Yang, Ya-Nan; Zhang, Fan; Feng, Zi-Ming; Jiang, Jian-Shuang; Zhang, Pei-Cheng; Journal of Asian Natural Products Research; vol. 14; 10; (2012); p. 981 - 985. |
| F3 | *Arctium lappa* | M019114 | Jeelani; Khuroo; Natural Product Research; vol. 26; 7; (2012); p. 654 - 658. |
| F3 | *Arctium lappa* | M019534 | Jeelani; Khuroo; Natural Product Research; vol. 26; 7; (2012); p. 654 - 658. |
| F3 | *Arctium lappa* | M021095 | Yang, Ya-Nan; Zhang, Fan; Feng, Zi-Ming; Jiang, Jian-Shuang; Zhang, Pei-Cheng; Journal of Asian Natural Products Research; vol. 14; 10; (2012); p. 981 - 985,5.; Yang, Ya-Nan; Zhang, Fan; Feng, Zi-Ming; Jiang, Jian-Shuang; Zhang, Pei-Cheng; Journal of Asian Natural Products Research; vol. 14; 10; (2012); p. 981 - 985. |
| F3 | *Arctium lappa* | M022175 | Umezawa, Toshiaki; Shimada, Mikio; Bioscience, Biotechnology and Biochemistry; vol. 60; 4; (1996); p. 736 - 737. |
| F3 | *Arctium lappa* | M022563 | Ichihara et al.; Tetrahedron Letters; (1978); p. 3035. |
| F3 | *Arctium lappa* | M022744 | Maruta, Yoshihiko; Kawabata, Jun; Niki, Ryoya; Journal of Agricultural and Food Chemistry; vol. 43; 10; (1995); p. 2592 - 2595. |
| F3 | *Arctium lappa* | M023307 | Umehara; Sugawa; Kuroyanagi; Ueno; Taki; Chemical and Pharmaceutical Bulletin; vol. 41; 10; (1993); p. 1774 - 1779. |
| F3 | *Arctium lappa* | M023618 | Park, So Young; Hong, Seong Su; Han, Xiang Hua; Hwang, Ji Sang; Lee, Dongho; Ro, Jai Seup; Hwang, Bang Yeon; Chemical and Pharmaceutical Bulletin; vol. 55; 1; (2007); p. 150 - 152. |
| F3 | *Arctium lappa* | M023863 | Washino, Tsutomu; Kobayashi, Hidetoshi; Ikawa, Yoshitomi; Agricultural and Biological Chemistry; vol. 51; 6; (1987); p. 1475 - 1480. |
| F3 | *Arctium lappa* | M023957 | Park, So Young; Hong, Seong Su; Han, Xiang Hua; Hwang, Ji Sang; Lee, Dongho; Ro, Jai Seup; Hwang, Bang Yeon; Chemical and Pharmaceutical Bulletin; vol. 55; 1; (2007); p. 150 - 152. |
| F3 | *Arctium lappa* | M023974 | Retrieved from CNPD |
| F3 | *Arctium lappa* | M024095 | Umehara; Sugawa; Kuroyanagi; Ueno; Taki; Chemical and Pharmaceutical Bulletin; vol. 41; 10; (1993); p. 1774 - 1779. |
| F3 | *Arctium lappa* | M025207 | Washino, Tsutomu; Yoshikura, Masahiro; Obata, Shigeo; Agricultural and Biological Chemistry; vol. 50; 1; (1986); p. 263 - 270. |
| F3 | *Arctium lappa* | M025230 | Higashinakasu, Keiko; Yamada, Kosumi; Shigemori, Hideyuki; Hasegawa, Koji; Heterocycles; vol. 65; 6; (2005); p. 1431 - 1437. |
| F3 | *Arctium lappa* | M025713 | Retrieved from CNPD |
| F3 | *Arctium lappa* | M026546 | Yamada, Kosumi; Anai, Toyoaki; Hasegawa, Koji; Phytochemistry (Elsevier); vol. 39; 5; (1995); p. 1031 - 1032. |
| F3 | *Arctium lappa* | M027127 | Umehara; Nakamura; Miyase; Kuroyanagi; Ueno; Chemical and Pharmaceutical Bulletin; vol. 44; 12; (1996); p. 2300 - 2304. |
| F3 | *Arctium lappa* | M027329 | Ichihara; Numata; Kanai; Sakamura; Agricultural and Biological Chemistry; vol. 41; 9; (1977); p. 1813 - 1814. |
| F3 | *Arctium lappa* | M027410 | Akihisa, Toshihiro; Yasukawa, Ken; Oinuma, Hirotoshi; Kasahara, Yoshimasa; Yamanouchi, Sakae; Takido, Michio; Kumaki, Kunio; Tamura, Toshitake; Phytochemistry; vol. 43; 6; (1996); p. 1255 - 1260. |
| F3 | *Arctium lappa* | M027657 | Retrieved from CNPD |
| F3 | *Arctium lappa* | M027783 | 国家中医药管理局《中华本草》编委会. 中华本草. Vol. 1-30 上海:上海科学技术出版社, 1999 |
| F3 | *Arctium lappa* | M027805 | Foelderak et al.; Acta Physica et Chemica; vol. 20; (1974); p. 459,461. |
| F3 | *Arctium lappa* | M028303 | Umehara; Sugawa; Kuroyanagi; Ueno; Taki; Chemical and Pharmaceutical Bulletin; vol. 41; 10; (1993); p. 1774 - 1779. |
| F3 | *Arctium lappa* | M028829 | Park, So Young; Hong, Seong Su; Han, Xiang Hua; Hwang, Ji Sang; Lee, Dongho; Ro, Jai Seup; Hwang, Bang Yeon; Chemical and Pharmaceutical Bulletin; vol. 55; 1; (2007); p. 150 - 152. |
| F3 | *Arctium lappa* | M029434 | Retrieved from CNPD |
| F3 | *Arctium lappa* | M029567 | Umehara; Sugawa; Kuroyanagi; Ueno; Taki; Chemical and Pharmaceutical Bulletin; vol. 41; 10; (1993); p. 1774 - 1779. |
| F3 | *Arctium lappa* | M029770 | Park, So Young; Hong, Seong Su; Han, Xiang Hua; Hwang, Ji Sang; Lee, Dongho; Ro, Jai Seup; Hwang, Bang Yeon; Chemical and Pharmaceutical Bulletin; vol. 55; 1; (2007); p. 150 - 152. |
| F3 | *Arctium lappa* | M029865 | Washino, Tsutomu; Yoshikura, Masahiro; Obata, Shigeo; Agricultural and Biological Chemistry; vol. 50; 1; (1986); p. 263 - 270. |
| F3 | *Arctium lappa* | M030133 | Naya,K. et al.; Chemistry Letters; (1972); p. 235 - 236. |
| F3 | *Arctium lappa* | M030304 | Retrieved from CNPD |
| F3 | *Arctium lappa* | M030774 | Washino, Tsutomu; Yoshikura, Masahiro; Obata, Shigeo; Agricultural and Biological Chemistry; vol. 50; 1; (1986); p. 263 - 270. |
| F3 | *Arctium lappa* | M030801 | Umehara; Sugawa; Kuroyanagi; Ueno; Taki; Chemical and Pharmaceutical Bulletin; vol. 41; 10; (1993); p. 1774 - 1779. |
| F3 | *Arctium lappa* | M031170 | (1) 季宇彬等. 中药抗肿瘤有效成分药理与应用. 哈尔滨: 黑龙江科学技术出版社, 1998. (2) 孙文基等. 天然活性成分简明手册. 北京: 中国医药科技出版社, 1998. (3) 陈蕙芳等. 植物活性成分辞典. 第2册. 北京: 中国医�┛萍汲霭嫔� 2001. (4) Buckingham J(Executive Editor): et al. Dictionary of Natural Products, Vol 1-7, Chapman & Hall, London, 1994; 1995, Vol 8; 1996, Vol 9; 1997, Vol 10; 1998, Vol 11.. |
| F3 | *Arctium lappa* | M031929 | Washino, Tsutomu; Yoshikura, Masahiro; Obata, Shigeo; Agricultural and Biological Chemistry; vol. 50; 1; (1986); p. 263 - 270. |
| F3 | *Arctium lappa* | M031951 | 江苏新医学院. 中药大辞典. 上海: 上海科学技术出版社, 1977 |
| F3 | *Arctium lappa* | M032021 | 国家中医药管理局《中华本草》编委会. 中华本草. Vol. 1-30 上海:上海科学技术出版社, 1999 |
| F3 | *Arctium lappa* | M032689 | Umehara; Sugawa; Kuroyanagi; Ueno; Taki; Chemical and Pharmaceutical Bulletin; vol. 41; 10; (1993); p. 1774 - 1779. |
| F3 | *Arctium lappa* | M033269 | Matsumoto; Hosono-Nishiyama; Yamada; Planta Medica; vol. 72; 3; (2006); p. 276 - 278. |
| F3 | *Arctium lappa* | M033351 | Ichihara; Numata; Kanai; Sakamura; Agricultural and Biological Chemistry; vol. 41; 9; (1977); p. 1813 - 1814. |
| F3 | *Arctium lappa* | M033773 | Maruta, Yoshihiko; Kawabata, Jun; Niki, Ryoya; Journal of Agricultural and Food Chemistry; vol. 43; 10; (1995); p. 2592 - 2595. |
| F3 | *Arctium lappa* | M033909 | Akihisa, Toshihiro; Yasukawa, Ken; Oinuma, Hirotoshi; Kasahara, Yoshimasa; Yamanouchi, Sakae; Takido, Michio; Kumaki, Kunio; Tamura, Toshitake; Phytochemistry; vol. 43; 6; (1996); p. 1255 - 1260. |
| F3 | *Arctium lappa* | M033937 | Washino, Tsutomu; Yoshikura, Masahiro; Obata, Shigeo; Agricultural and Biological Chemistry; vol. 50; 1; (1986); p. 263 - 270. |
| F3 | *Carthamus tinctorius* | M000015 | Allen; Thomas; Phytochemistry (Elsevier); vol. 10; (1971); p. 1579,1580. |
| F3 | *Carthamus tinctorius* | M000067 | Akihisa, Toshihiro; Oinuma, Hirotoshi; Tamura, Toshitake; Kasahara, Yoshimasa; Kumaki, Kunio; et al.; Phytochemistry (Elsevier); vol. 36; 1; (1994); p. 105 - 108. |
| F3 | *Carthamus tinctorius* | M000543 | Retrieved from CNPD |
| F3 | *Carthamus tinctorius* | M000569 | (1) 阴健等. 中药现代研究与临床应用(1). 北京: 学苑出版社, 1993. (2) 欧明等. 简明中药成分手册. 北京: 中国医药科技出版社, 2003. |
| F3 | *Carthamus tinctorius* | M000697 | 国家中医药管理局《中华本草》编委会. 中华本草. Vol. 1-30 上海:上海科学技术出版社, 1999 |
| F3 | *Carthamus tinctorius* | M000780 | Yin, Hong-Bin; He, Zhi-Sheng; Tetrahedron Letters; vol. 41; 12; (2000); p. 1955 - 1958. |
| F3 | *Carthamus tinctorius* | M001457 | 李锋等. 中草药, 2004, 35 (3): 247 |
| F3 | *Carthamus tinctorius* | M001683 | Kuliev, A. A.; Gigienova, E. I.; Umarov, A. U.; Kuliev, V. B.; Aslanov, S. M.; Chemistry of Natural Compounds; vol. 18; 1; (1982); p. 32 - 35; Khimiya Prirodnykh Soedinenii; vol. 18; 1; (1982); p. 36 - 40. |
| F3 | *Carthamus tinctorius* | M002205 | (1) 江苏新医学院. 中药大辞典. 上海: 上海科学技术出版社, 1977. (2) 国家中医药管理局《中华本草》编委会. 中华本草. Vol. 1-30 上海:上海科学技术出版社, 1999. (3) 陈蕙芳等. 植物活性成分辞典. 第1册. 北京: 中国医药科技出版社, 2001. |
| F3 | *Carthamus tinctorius* | M002557 | Hilditch,T.P.; The Chemical Constitution of Natural Fats, 3.Aufl. <London 1956> S.172,173. |
| F3 | *Carthamus tinctorius* | M002823 | Kazuma, Kohei; Takahashi, Takashi; Sato, Katsura; Takeuchi, Hisatomo; Matsumoto, Takeshi; Okuno, Toshikatsu; Bioscience, Biotechnology and Biochemistry; vol. 64; 8; (2000); p. 1588 - 1599. |
| F3 | *Carthamus tinctorius* | M002862 | Bohlmann,F. et al.; Chemische Berichte; vol. 99; (1966); p. 3433 - 3436. |
| F3 | *Carthamus tinctorius* | M002920 | (1) 阴健等. 中药现代研究与临床应用(1). 北京: 学苑出版社, 1993. (2) 欧明等. 简明中药成分手册. 北京: 中国医药科技出版社, 2003. |
| F3 | *Carthamus tinctorius* | M003402 | Zhang, Ge; Guo, Mei-Li; Li, Run-Ping; Li, Ying; Zhang, Han-Ming; Su, Zhong-Wu; Chemistry of Natural Compounds; vol. 45; 3; (2009); p. 398 - 401. |
| F3 | *Carthamus tinctorius* | M003449 | Hattori, Masao; Huang, Xin-li; Che, Qing-Ming; Kawata, Yukio; Tezuka, Yasuhiro; et al.; Phytochemistry (Elsevier); vol. 31; 11; (1992); p. 4001 - 4004. |
| F3 | *Carthamus tinctorius* | M003534 | (1) 江苏新医学院. 中药大辞典. 上海: 上海科学技术出版社, 1977. (2) 国家中医药管理局《中华本草》编委会. 中华本草. Vol. 1-30 上海:上海科学技术出版社, 1999. |
| F3 | *Carthamus tinctorius* | M003569 | Han, Shu-Yan; Li, Hai-Xia; Bai, Chang-Cai; Wang, Li; Tu, Peng-Fei; Chemistry and Biodiversity; vol. 7; 2; (2010); p. 383 - 391. |
| F3 | *Carthamus tinctorius* | M003772 | Bohlmann,F. et al.; Chemische Berichte; vol. 99; (1966); p. 3433 - 3436. |
| F3 | *Carthamus tinctorius* | M003791 | Kazuma, Kohei; Shirai, Eriko; Wada, Mizu; Umeo, Kazuhiro; Sato, Atsushi; et al.; Bioscience, Biotechnology, and Biochemistry; vol. 59; 8; (1995); p. 1588 - 1590. |
| F3 | *Carthamus tinctorius* | M003938 | Sato, Hiroji; Kawagishi, Hirokazu; Nishimura, Tsutomu; Yoneyama, Syozou; Yoshimoto, Yuko; et al.; Agricultural and Biological Chemistry; vol. 49; 10; (1985); p. 2969 - 2974. |
| F3 | *Carthamus tinctorius* | M004231 | Yang, Wen-Zhi; Qiao, Xue; Bo, Tao; Wang, Qing; Guo, De-An; Ye, Min; Rapid Communications in Mass Spectrometry; vol. 28; 4; (2014); p. 385 - 395. |
| F3 | *Carthamus tinctorius* | M004357 | (1) Buckingham J(Executive Editor): et al. Dictionary of Natural Products, Vol 1-7, Chapman & Hall, London, 1994; 1995, Vol 8; 1996, Vol 9; 1997, Vol 10; 1998, Vol 11.. (2) JIANG Y, et al. Chem Pharm Bull, 2005, 53 (9): 1164. |
| F3 | *Carthamus tinctorius* | M004395 | Hattori, Masao; Huang, Xin-li; Che, Qing-Ming; Kawata, Yukio; Tezuka, Yasuhiro; et al.; Phytochemistry (Elsevier); vol. 31; 11; (1992); p. 4001 - 4004. |
| F3 | *Carthamus tinctorius* | M004590 | Retrieved from CNPD |
| F3 | *Carthamus tinctorius* | M004632 | Nishibe et al.; Phytochemistry (Elsevier); vol. 11; (1972); p. 2623. |
| F3 | *Carthamus tinctorius* | M005228 | 阴健等. 中药现代研究与临床应用(1). 北京: 学苑出版社, 1993 |
| F3 | *Carthamus tinctorius* | M005302 | Furuya, Tsutomu; Yoshikawa, Takafumi; Kimura, Takako; Kaneko, Hiroko; Phytochemistry (Elsevier); vol. 26; 10; (1987); p. 2741 - 2748. |
| F3 | *Carthamus tinctorius* | M005342 | Ahmed; Marzouk; El-Khrisy; Abdel Wahab; El-Din; Pharmazie; vol. 55; 8; (2000); p. 621 - 622. |
| F3 | *Carthamus tinctorius* | M005470 | Akihisa, Toshihiro; Oinuma, Hirotoshi; Tamura, Toshitake; Kasahara, Yoshimasa; Kumaki, Kunio; et al.; Phytochemistry (Elsevier); vol. 36; 1; (1994); p. 105 - 108. |
| F3 | *Carthamus tinctorius* | M005515 | Kumazawa, Toshihiro; Sato, Shingo; Kanenari, Daisuke; Kunimatsu, Akira; Hirose, Ryoji; et al.; Chemistry Letters; 12; (1994); p. 2343 - 2344. |
| F3 | *Carthamus tinctorius* | M005572 | Binder et al.; Phytochemistry (Elsevier); vol. 17; (1978); p. 315,317. |
| F3 | *Carthamus tinctorius* | M006069 | (1) 国家中医药管理局《中华本草》编委会. 中华本草. Vol. 1-30 上海:上海科学技术出版社, 1999. (2) Junichi O, et al.Chem. Abstr., 1981, 95, 43572s. |
| F3 | *Carthamus tinctorius* | M006110 | Yamato M, et al. Chem. Abstr., 1990, 112, 204663j |
| F3 | *Carthamus tinctorius* | M006185 | Retrieved from CNPD |
| F3 | *Carthamus tinctorius* | M006230 | Sakamura, Sadao; Terayama, Yoshihiko; Kawakatsu, Satomi; Ichihara, Akitami; Saito, Hideya; Agricultural and Biological Chemistry; vol. 44; 12; (1980); p. 2951 - 2954. |
| F3 | *Carthamus tinctorius* | M006408 | Zhang, Ge; Guo, Mei-Li; Li, Run-Ping; Li, Ying; Zhang, Han-Ming; Su, Zhong-Wu; Chemistry of Natural Compounds; vol. 45; 3; (2009); p. 398 - 401. |
| F3 | *Carthamus tinctorius* | M006618 | Bohlmann,F. et al.; Chemische Berichte; vol. 99; (1966); p. 3433 - 3436. |
| F3 | *Carthamus tinctorius* | M006806 | (1) 阴健等. 中药现代研究与临床应用(1). 北京: 学苑出版社, 1993. (2) 孙文基等. 天然活性成分简明手册. 北京: 中国医药科技出版社, 1998. (3) 国家中医药管理局《中华本草》编委会. 中华本草. Vol. 1-30 上海:上海科学技术出版社, 1999. |
| F3 | *Carthamus tinctorius* | M006909 | Hattori, Masao; Huang, Xin-li; Che, Qing-Ming; Kawata, Yukio; Tezuka, Yasuhiro; et al.; Phytochemistry (Elsevier); vol. 31; 11; (1992); p. 4001 - 4004. |
| F3 | *Carthamus tinctorius* | M007021 | Akihisa, Toshihiro; Oinuma, Hirotoshi; Tamura, Toshitake; Kasahara, Yoshimasa; Kumaki, Kunio; et al.; Phytochemistry (Elsevier); vol. 36; 1; (1994); p. 105 - 108. |
| F3 | *Carthamus tinctorius* | M007166 | Yoo, Hye Hyun; Park, Jeong Hill; Kwon, Sung Won; Bioscience, Biotechnology and Biochemistry; vol. 70; 11; (2006); p. 2783 - 2785. |
| F3 | *Carthamus tinctorius* | M007271 | 国家中医药管理局《中华本草》编委会. 中华本草. Vol. 1-30 上海:上海科学技术出版社, 1999 |
| F3 | *Carthamus tinctorius* | M007369 | Advanced Gene Technology, Corp.; EP1205182; B1; (2005). |
| F3 | *Carthamus tinctorius* | M007397 | Han, Shu-Yan; Li, Hai-Xia; Bai, Chang-Cai; Wang, Li; Tu, Peng-Fei; Chemistry and Biodiversity; vol. 7; 2; (2010); p. 383 - 391. |
| F3 | *Carthamus tinctorius* | M007429 | Retrieved from CNPD |
| F3 | *Carthamus tinctorius* | M007435 | Retrieved from CNPD |
| F3 | *Carthamus tinctorius* | M007769 | 国家中医药管理局《中华本草》编委会. 中华本草. Vol. 1-30 上海:上海科学技术出版社, 1999 |
| F3 | *Carthamus tinctorius* | M007827 | Retrieved from CNPD |
| F3 | *Carthamus tinctorius* | M007833 | Suleimanov; Chemistry of Natural Compounds; vol. 40; 1; (2004); p. 13 - 15. |
| F3 | *Carthamus tinctorius* | M008015 | He, Jun; Shen, Yi; Jiang, Jian-Shuang; Yang, Ya-Nan; Feng, Zi-Ming; Zhang, Pei-Cheng; Yuan, Shao-Peng; Hou, Qi; Carbohydrate Research; vol. 346; 13; (2011); p. 1903 - 1908. |
| F3 | *Carthamus tinctorius* | M008130 | Suleimanov; Chemistry of Natural Compounds; vol. 40; 1; (2004); p. 13 - 15. |
| F3 | *Carthamus tinctorius* | M008269 | Zhang, Ge; Guo, Mei-Li; Li, Run-Ping; Li, Ying; Zhang, Han-Ming; Su, Zhong-Wu; Chemistry of Natural Compounds; vol. 45; 3; (2009); p. 398 - 401. |
| F3 | *Carthamus tinctorius* | M008334 | Binder et al.; Phytochemistry (Elsevier); vol. 17; (1978); p. 315,317. |
| F3 | *Carthamus tinctorius* | M008428 | Yang, Wen-Zhi; Qiao, Xue; Bo, Tao; Wang, Qing; Guo, De-An; Ye, Min; Rapid Communications in Mass Spectrometry; vol. 28; 4; (2014); p. 385 - 395. |
| F3 | *Carthamus tinctorius* | M008471 | Hattori, Masao; Huang, Xin-li; Che, Qing-Ming; Kawata, Yukio; Tezuka, Yasuhiro; et al.; Phytochemistry (Elsevier); vol. 31; 11; (1992); p. 4001 - 4004. |
| F3 | *Carthamus tinctorius* | M008706 | Zhou, Yu-Zhi; Ma, Hong-Yu; Chen, Huan; Qiao, Li; Yao, Yao; Cao, Jia-Qing; Pei, Yue-Hu; Chemical and Pharmaceutical Bulletin; vol. 54; 10; (2006); p. 1455 - 1456. |
| F3 | *Carthamus tinctorius* | M008762 | Sakamura, Sadao; Terayama, Yoshihiko; Kawakatsu, Satomi; Ichihara, Akitami; Saito, Hideya; Agricultural and Biological Chemistry; vol. 44; 12; (1980); p. 2951 - 2954. |
| F3 | *Carthamus tinctorius* | M008808 | Sakamura, Sadao; Terayama, Yoshihiko; Kawakatsu, Satomi; Ichihara, Akitami; Saito, Hideya; Agricultural and Biological Chemistry; vol. 44; 12; (1980); p. 2951 - 2954. |
| F3 | *Carthamus tinctorius* | M008986 | (1) 阴健等. 中药现代研究与临床应用(1). 北京: 学苑出版社, 1993. (2) 饶高雄等. 药学学报, 1991, 26 (1): 30. (3) 杨峻山等. 药学学报, 1993, 28 (3): 197. (4) 赵余庆等. 中国中药杂志, 1993, 18 (7): 428. (5) �母咝鄣� 中国中药杂志, 1993, 18 (12): 736. (6) 饶高雄等. 中国中药杂志, 1995, 20 (12): 740. (7) 国家中医药管理局《中华本草》编委会. 中华本草. Vol. 1-30 上海:上海科学技术出版社, 1999. (8) TORIUMI Y, et al. Chem Pharm Bull, 2003, 51 (1): 89. (9) Carcache-Blanco EJ, et al. JNP, 2003, 67 (1): 126. |
| F3 | *Carthamus tinctorius* | M009036 | Zhu H, et al. Planta Med, 2003, 69, 429 |
| F3 | *Carthamus tinctorius* | M009111 | Meselhy; Kadota; Momose; Hatakeyama; Kusai; Hattori; Namba; Chemical and Pharmaceutical Bulletin; vol. 41; 10; (1993); p. 1796 - 1802. |
| F3 | *Carthamus tinctorius* | M009146 | 国家中医药管理局《中华本草》编委会. 中华本草. Vol. 1-30 上海:上海科学技术出版社, 1999 |
| F3 | *Carthamus tinctorius* | M009470 | Binder et al.; Phytochemistry (Elsevier); vol. 14; (1975); p. 2085,2086. |
| F3 | *Carthamus tinctorius* | M009514 | Kazuma, Kohei; Takahashi, Takashi; Sato, Katsura; Takeuchi, Hisatomo; Matsumoto, Takeshi; Okuno, Toshikatsu; Bioscience, Biotechnology and Biochemistry; vol. 64; 8; (2000); p. 1588 - 1599. |
| F3 | *Carthamus tinctorius* | M009594 | Kazuma, Kohei; Takahashi, Takashi; Sato, Katsura; Takeuchi, Hisatomo; Matsumoto, Takeshi; Okuno, Toshikatsu; Bioscience, Biotechnology and Biochemistry; vol. 64; 8; (2000); p. 1588 - 1599. |
| F3 | *Carthamus tinctorius* | M009773 | (1) 阴健等. 中药现代研究与临床应用(1). 北京: 学苑出版社, 1993. (2) Ma WZ, et al. JNP, 2003, 66 (3): 441. (3) Yang XD, et al. Phytochemistry, 2001, 58, 1245. |
| F3 | *Carthamus tinctorius* | M009922 | Suleimanov; Chemistry of Natural Compounds; vol. 40; 1; (2004); p. 13 - 15. |
| F3 | *Carthamus tinctorius* | M010095 | Hattori, Masao; Huang, Xin-li; Che, Qing-Ming; Kawata, Yukio; Tezuka, Yasuhiro; et al.; Phytochemistry (Elsevier); vol. 31; 11; (1992); p. 4001 - 4004. |
| F3 | *Carthamus tinctorius* | M010652 | Akihisa, Toshihiro; Oinuma, Hirotoshi; Tamura, Toshitake; Kasahara, Yoshimasa; Kumaki, Kunio; et al.; Phytochemistry (Elsevier); vol. 36; 1; (1994); p. 105 - 108. |
| F3 | *Carthamus tinctorius* | M010880 | 国家中医药管理局《中华本草》编委会. 中华本草. Vol. 1-30 上海:上海科学技术出版社, 1999 |
| F3 | *Carthamus tinctorius* | M010882 | 孙文基等. 天然活性成分简明手册. 北京: 中国医药科技出版社, 1998 |
| F3 | *Carthamus tinctorius* | M010883 | Cho, Man-Ho; Paik, Young-Sook; Hahn, Tae-Ryong; Journal of Agricultural and Food Chemistry; vol. 48; 9; (2000); p. 3917 - 3921. |
| F3 | *Carthamus tinctorius* | M010900 | Retrieved from CNPD |
| F3 | *Carthamus tinctorius* | M011067 | 国家中医药管理局《中华本草》编委会. 中华本草. Vol. 1-30 上海:上海科学技术出版社, 1999 |
| F3 | *Carthamus tinctorius* | M011182 | Kazuma, Kohei; Takahashi, Takashi; Sato, Katsura; Takeuchi, Hisatomo; Matsumoto, Takeshi; Okuno, Toshikatsu; Bioscience, Biotechnology and Biochemistry; vol. 64; 8; (2000); p. 1588 - 1599. |
| F3 | *Carthamus tinctorius* | M011700 | Kazuma, Kohei; Takahashi, Takashi; Sato, Katsura; Takeuchi, Hisatomo; Matsumoto, Takeshi; Okuno, Toshikatsu; Bioscience, Biotechnology and Biochemistry; vol. 64; 8; (2000); p. 1588 - 1599. |
| F3 | *Carthamus tinctorius* | M011938 | Akihisa, Toshihiro; Oinuma, Hirotoshi; Tamura, Toshitake; Kasahara, Yoshimasa; Kumaki, Kunio; et al.; Phytochemistry (Elsevier); vol. 36; 1; (1994); p. 105 - 108. |
| F3 | *Carthamus tinctorius* | M012196 | Kazuma, Kohei; Takahashi, Takashi; Sato, Katsura; Takeuchi, Hisatomo; Matsumoto, Takeshi; Okuno, Toshikatsu; Bioscience, Biotechnology and Biochemistry; vol. 64; 8; (2000); p. 1588 - 1599. |
| F3 | *Carthamus tinctorius* | M012459 | Takahashi, Yoshiyuki; Saito, Koshi; Yanagiya, Mitsutoshi; Ikura, Mitsuhiko; Hikichi, Kunio; et al.; Tetrahedron Letters; vol. 25; 23; (1984); p. 2471 - 2474. |
| F3 | *Carthamus tinctorius* | M012509 | Zhao, Gang; Qin, Guo-Wei; Gai, Yue; Guo, Li-He; Chemical and Pharmaceutical Bulletin; vol. 58; 7; (2010); p. 950 - 952. |
| F3 | *Carthamus tinctorius* | M013391 | Nagatsu, Akito; Zhang, Hui Li; Watanabe, Toshihiro; Taniguchi, Nari; Hatano, Keiichiro; Mizukami, Hajime; Sakakibara, Jinsaku; Chemical and Pharmaceutical Bulletin; vol. 46; 6; (1998); p. 1044 - 1047. |
| F3 | *Carthamus tinctorius* | M013581 | Akihisa, Toshihiro; Oinuma, Hirotoshi; Tamura, Toshitake; Kasahara, Yoshimasa; Kumaki, Kunio; et al.; Phytochemistry (Elsevier); vol. 36; 1; (1994); p. 105 - 108. |
| F3 | *Carthamus tinctorius* | M013882 | 阴健等. 中药现代研究与临床应用(1). 北京: 学苑出版社, 1993 |
| F3 | *Carthamus tinctorius* | M014052 | Bohlmann,F.; Zdero,C.; Chemische Berichte; vol. 103; (1970); p. 2853 - 2855. |
| F3 | *Carthamus tinctorius* | M014514 | Ahmed; Marzouk; El-Khrisy; Abdel Wahab; El-Din; Pharmazie; vol. 55; 8; (2000); p. 621 - 622. |
| F3 | *Carthamus tinctorius* | M014910 | Review; Hui Li Zhang; Nagatsu; Sakakibara; Chemical and Pharmaceutical Bulletin; vol. 44; 4; (1996); p. 874 - 876. |
| F3 | *Carthamus tinctorius* | M015118 | Akihisa, Toshihiro; Oinuma, Hirotoshi; Tamura, Toshitake; Kasahara, Yoshimasa; Kumaki, Kunio; et al.; Phytochemistry (Elsevier); vol. 36; 1; (1994); p. 105 - 108. |
| F3 | *Carthamus tinctorius* | M015181 | Akihisa, Toshihiro; Oinuma, Hirotoshi; Tamura, Toshitake; Kasahara, Yoshimasa; Kumaki, Kunio; et al.; Phytochemistry (Elsevier); vol. 36; 1; (1994); p. 105 - 108. |
| F3 | *Carthamus tinctorius* | M015409 | (1) 阴健等. 中药现代研究与临床应用(1). 北京: 学苑出版社, 1993. (2) 国家中医药管理局《中华本草》编委会. 中华本草. Vol. 1-30 上海:上海科学技术出版社, 1999. |
| F3 | *Carthamus tinctorius* | M015510 | Zhou, Yu-Zhi; Ma, Hong-Yu; Chen, Huan; Qiao, Li; Yao, Yao; Cao, Jia-Qing; Pei, Yue-Hu; Chemical and Pharmaceutical Bulletin; vol. 54; 10; (2006); p. 1455 - 1456. |
| F3 | *Carthamus tinctorius* | M015819 | Hattori, Masao; Huang, Xin-li; Che, Qing-Ming; Kawata, Yukio; Tezuka, Yasuhiro; et al.; Phytochemistry (Elsevier); vol. 31; 11; (1992); p. 4001 - 4004. |
| F3 | *Carthamus tinctorius* | M015850 | Zhang, Hui Li; Nagatsu, Akito; Watanabe, Toshihiro; Sakakibara, Jinsaku; Okuyama, Harumi; Chemical and Pharmaceutical Bulletin; vol. 45; 12; (1997); p. 1910 - 1914. |
| F3 | *Carthamus tinctorius* | M015966 | (1) 阴健等. 中药现代研究与临床应用(1). 北京: 学苑出版社, 1993. (2) 国家中医药管理局《中华本草》编委会. 中华本草. Vol. 1-30 上海:上海科学技术出版社, 1999. |
| F3 | *Carthamus tinctorius* | M016917 | He, Jun; Shen, Yi; Jiang, Jian-Shuang; Yang, Ya-Nan; Feng, Zi-Ming; Zhang, Pei-Cheng; Yuan, Shao-Peng; Hou, Qi; Carbohydrate Research; vol. 346; 13; (2011); p. 1903 - 1908. |
| F3 | *Carthamus tinctorius* | M016918 | He, Jun; Shen, Yi; Jiang, Jian-Shuang; Yang, Ya-Nan; Feng, Zi-Ming; Zhang, Pei-Cheng; Yuan, Shao-Peng; Hou, Qi; Carbohydrate Research; vol. 346; 13; (2011); p. 1903 - 1908. |
| F3 | *Carthamus tinctorius* | M016919 | He, Jun; Shen, Yi; Jiang, Jian-Shuang; Yang, Ya-Nan; Feng, Zi-Ming; Zhang, Pei-Cheng; Yuan, Shao-Peng; Hou, Qi; Carbohydrate Research; vol. 346; 13; (2011); p. 1903 - 1908. |
| F3 | *Carthamus tinctorius* | M016921 | He, Jun; Shen, Yi; Jiang, Jian-Shuang; Yang, Ya-Nan; Feng, Zi-Ming; Zhang, Pei-Cheng; Yuan, Shao-Peng; Hou, Qi; Carbohydrate Research; vol. 346; 13; (2011); p. 1903 - 1908. |
| F3 | *Carthamus tinctorius* | M016922 | He, Jun; Shen, Yi; Jiang, Jian-Shuang; Yang, Ya-Nan; Feng, Zi-Ming; Zhang, Pei-Cheng; Yuan, Shao-Peng; Hou, Qi; Carbohydrate Research; vol. 346; 13; (2011); p. 1903 - 1908. |
| F3 | *Carthamus tinctorius* | M016923 | He, Jun; Shen, Yi; Jiang, Jian-Shuang; Yang, Ya-Nan; Feng, Zi-Ming; Zhang, Pei-Cheng; Yuan, Shao-Peng; Hou, Qi; Carbohydrate Research; vol. 346; 13; (2011); p. 1903 - 1908. |
| F3 | *Carthamus tinctorius* | M016924 | He, Jun; Shen, Yi; Jiang, Jian-Shuang; Yang, Ya-Nan; Feng, Zi-Ming; Zhang, Pei-Cheng; Yuan, Shao-Peng; Hou, Qi; Carbohydrate Research; vol. 346; 13; (2011); p. 1903 - 1908. |
| F3 | *Carthamus tinctorius* | M016925 | He, Jun; Shen, Yi; Jiang, Jian-Shuang; Yang, Ya-Nan; Feng, Zi-Ming; Zhang, Pei-Cheng; Yuan, Shao-Peng; Hou, Qi; Carbohydrate Research; vol. 346; 13; (2011); p. 1903 - 1908. |
| F3 | *Carthamus tinctorius* | M017651 | Retrieved from CNPD |
| F3 | *Carthamus tinctorius* | M017719 | Roh, Jung Seop; Han, Ji Young; Kim, Jung Han; Hwang, Jae Kwan; Biological and Pharmaceutical Bulletin; vol. 27; 12; (2004); p. 1976 - 1978. |
[truncated: 177,388 more chars]
