# Supplementary material for: Chinese Medicine for Psoriasis Vulgaris Based on Syndrome Pattern: A Network Pharmacological Study
Source: Evid Based Complement Alternat Med. 2020 Apr 28;2020:5239854. doi: 10.1155/2020/5239854 (PMC7204377; doi:10.1155/2020/5239854)
Supplement: Supplementary Materials — Figure S1: ADME/T properties of compounds of three TCM formulae. Table S1: herb composition and compounds of each formula retrieved from PDTCM. Table S2: psoriasis-related proteins. Table S3: compound-target binding energy lower than −12.28 kcal/mol. Table S4: GSEA results for three TCM formulae. [file 5239854.f1.zip › 5239854.f1/Table S2.docx]

**Table S2: Psoriasis-related proteins.**

| **UniProt ID** | **Protein Name** | **Gene** | **Available for docking** |
| --- | --- | --- | --- |
| A9UF02 | BCR/ABL fusion protein isoform X9 | BCR/ABL fusion | N/A |
| O14594 | Neurocan core protein | NCAN | N/A |
| O14646 | Chromodomain-helicase-DNA-binding protein 1 | CHD1 | N/A |
| O14727 | Apoptotic protease-activating factor 1 | APAF1 | 1Z6T |
| O14757 | Serine/threonine-protein kinase Chk1 | CHEK1 | 3U9N |
| O14788 | Tumor necrosis factor ligand superfamily member 11 | TNFSF11 | N/A |
| O14920 | Inhibitor of nuclear factor kappa-B kinase subunit beta | IKBKB | 4KIK |
| O15111 | Inhibitor of nuclear factor kappa-B kinase subunit alpha | CHUK | N/A |
| O15151 | Protein Mdm4 | MDM4 | 3LBJ |
| O15379 | Histone deacetylase 3 | HDAC3 | 4A69 |
| O15392 | Baculoviral IAP repeat-containing protein 5 | BIRC5 | 3UEC |
| O15438 | Canalicular multispecific organic anion transporter 2 | ABCC3 | N/A |
| O15439 | Multidrug resistance-associated protein 4 | ABCC4 | N/A |
| O15528 | 25-hydroxyvitamin D-1 alpha hydroxylase, mitochondrial | CYP27B1 | N/A |
| O15530 | 3-phosphoinositide-dependent protein kinase 1 | PDPK1 | 1W1D |
| O43174 | Cytochrome P450 26A1 | CYP26A1 | N/A |
| O43519 | RET proto-oncogene | RET | N/A |
| O43521 | Bcl-2-like protein 11 | BCL2L11 | N/A |
| O43524 | Forkhead box protein O3 | FOXO3 | N/A |
| O43525 | Potassium voltage-gated channel subfamily KQT member 3 | KCNQ3 | N/A |
| O43526 | Potassium voltage-gated channel subfamily KQT member 2 | KCNQ2 | N/A |
| O43741 | 5'-AMP-activated protein kinase subunit beta-2 | PRKAB2 | 2V8Q |
| O43915 | Vascular endothelial growth factor D | VEGFD | N/A |
| O60656 | UDP-glucuronosyltransferase 1-9 | UGT1A9 | N/A |
| O60674 | Tyrosine-protein kinase JAK2 | JAK2 | 3TJC |
| O60760 | Hematopoietic prostaglandin D synthase | HPGDS | 2CVD |
| O67135 | Acetoin utilization protein | acuC1 | 1C3R |
| O75015 | Low affinity immunoglobulin gamma Fc region receptor III-B | FCGR3B | 1T83 |
| O75330 | Hyaluronan mediated motility receptor | HMMR | N/A |
| O75469 | Nuclear receptor subfamily 1 group I member 2 | NR1I2 | 1M13 |
| O75582 | Ribosomal protein S6 kinase alpha-5 | RPS6KA5 | 3KN5 |
| O75911 | Short-chain dehydrogenase/reductase 3 | DHRS3 | N/A |
| O94788 | Retinal dehydrogenase 2 | ALDH1A2 | N/A |
| O95237 | Lecithin retinol acyltransferase | LRAT | N/A |
| O95342 | Bile salt export pump | ABCB11 | N/A |
| O95363 | Phenylalanine--tRNA ligase, mitochondrial | FARS2 | 3CMQ |
| O95644 | Nuclear factor of activated T-cells, cytoplasmic 1 | NFATC1 | N/A |
| O96017 | Serine/threonine-protein kinase Chk2 | CHEK2 | 2WTI |
| P00338 | L-lactate dehydrogenase A chain | LDHA | 1I10 |
| P00352 | Retinal dehydrogenase 1 | ALDH1A1 | N/A |
| P00374 | Dihydrofolate reductase | DHFR | 2W3A |
| P00390 | Glutathione reductase, mitochondrial | GSR | 2GH5 |
| P00439 | Phenylalanine-4-hydroxylase | PAH | 1TG2 |
| P00441 | Superoxide dismutase [Cu-Zn] | SOD1 | 2WZ0 |
| P00491 | Purine nucleoside phosphorylase | PNP | 3K8O |
| P00492 | Hypoxanthine-guanine phosphoribosyltransferase | HPRT1 | 1BZY |
| P00519 | Tyrosine-protein kinase ABL1 | ABL1 | 2HYY |
| P00533 | Epidermal growth factor receptor | EGFR | 1XKK |
| P00734 | Prothrombin | F2 | 3DUX |
| P00736 | Complement C1r subcomponent | C1R | N/A |
| P00748 | Coagulation factor XII | F12 | N/A |
| P00750 | Tissue-type plasminogen activator | PLAT | 1RTF |
| P00813 | Adenosine deaminase | ADA | 3IAR |
| P01024 | Complement C3 | C3 | N/A |
| P01031 | Complement C5 | C5 | N/A |
| P01106 | Myc proto-oncogene protein | MYC | N/A |
| P01112 | GTPase HRas | HRAS | 3KKM |
| P01130 | Low-density lipoprotein receptor | LDLR | N/A |
| P01133 | Pro-epidermal growth factor | EGF | N/A |
| P01137 | Transforming growth factor beta-1 | TGFB1 | N/A |
| P01374 | Lymphotoxin-alpha | LTA | N/A |
| P01375 | Tumor necrosis factor | TNF | 2AZ5 |
| P01579 | Interferon gamma | IFNG | N/A |
| P01583 | Interleukin-1 alpha | IL1A | N/A |
| P01584 | Interleukin-1 beta | IL1B | N/A |
| P01589 | Interleukin-2 receptor subunit alpha | IL2RA | N/A |
| P01730 | T-cell surface glycoprotein CD4 | CD4 | N/A |
| P01857 | Immunoglobulin heavy constant gamma 1 | IGHG1 | N/A |
| P02741 | C-reactive protein | CRP | 3L2Y |
| P02745 | Complement C1q subcomponent subunit A | C1QA | 2JG8 |
| P02746 | Complement C1q subcomponent subunit B | C1QB | N/A |
| P02747 | Complement C1q subcomponent subunit C | C1QC | 2WNU |
| P02763 | Alpha-1-acid glycoprotein 1 | ORM1 | N/A |
| P02768 | Serum albumin | ALB | N/A |
| P02774 | Vitamin D-binding protein | GC | 1J78 |
| P02788 | Lactotransferrin | LTF | N/A |
| P02818 | Osteocalcin | BGLAP | N/A |
| P03372 | Estrogen receptor | ESR1 | 2QXS |
| P03951 | Coagulation factor XI | F11 | 1ZOM |
| P03952 | Plasma kallikrein | KLKB1 | N/A |
| P03956 | Interstitial collagenase | MMP1 | 966C |
| P04049 | RAF proto-oncogene serine/threonine-protein kinase | RAF1 | 1GUA |
| P04054 | Phospholipase A2 | PLA2G1B | N/A |
| P04083 | Annexin A1 | ANXA1 | N/A |
| P04085 | Platelet-derived growth factor subunit A | PDGFA | N/A |
| P04150 | Glucocorticoid receptor | NR3C1 | 3H52 |
| P04229 | HLA class II histocompatibility antigen, DRB1-1 beta chain | HLA-DRB1 | 4GBX |
| P04234 | T-cell surface glycoprotein CD3 delta chain | CD3D | N/A |
| P04271 | Protein S100-B | S100B | 3HCM |
| P04278 | Sex hormone-binding globulin | SHBG | N/A |
| P04626 | Receptor tyrosine-protein kinase erbB-2 | ERBB2 | 3RCD |
| P04628 | Proto-oncogene Wnt-1 | WNT1 | N/A |
| P04629 | High affinity nerve growth factor receptor | NTRK1 | 4AOJ |
| P04637 | Cellular tumor antigen p53 | TP53 | 5G4N |
| P04792 | Heat shock protein beta-1 | HSPB1 | N/A |
| P04798 | Cytochrome P450 1A1 | CYP1A1 | 4I8V |
| P04818 | Thymidylate synthase | TYMS | 3EJL |
| P05067 | Amyloid-beta A4 protein | APP | 5BUO |
| P05093 | Steroid 17-alpha-hydroxylase/17,20 lyase | CYP17A1 | 3RUK |
| P05106 | Integrin beta-3 | ITGB3 | 2VDM |
| P05108 | Cholesterol side-chain cleavage enzyme, mitochondrial | CYP11A1 | 3NA0 |
| P05113 | Interleukin-5 | IL5 | N/A |
| P05129 | Protein kinase C gamma type | PRKCG | N/A |
| P05164 | Myeloperoxidase | MPO | 3ZS0 |
| P05177 | Cytochrome P450 1A2 | CYP1A2 | N/A |
| P05181 | Cytochrome P450 2E1 | CYP2E1 | N/A |
| P05230 | Fibroblast growth factor 1 | FGF1 | 3K1X |
| P05231 | Interleukin-6 | IL6 | 1ALU |
| P05305 | Endothelin-1 | EDN1 | N/A |
| P05362 | Intercellular adhesion molecule 1 | ICAM1 | 5MZA |
| P05412 | Transcription factor AP-1 | JUN | N/A |
| P05556 | Integrin beta-1 | ITGB1 | 3PFW |
| P05771 | Protein kinase C beta type | PRKCB | 2I0E |
| P06126 | T-cell surface glycoprotein CD1a | CD1A | 1XZ0 |
| P06239 | Tyrosine-protein kinase Lck | LCK | 3AC1 |
| P06241 | Tyrosine-protein kinase Fyn | FYN | 2DQ7 |
| P06401 | Progesterone receptor | PGR | 2W8Y |
| P06493 | Cyclin-dependent kinase 1 | CDK1 | 5LQF |
| P06702 | Protein S100-A9 | S100A9 | 1IRJ |
| P06729 | T-cell surface antigen CD2 | CD2 | N/A |
| P06746 | DNA polymerase beta | POLB | 3ISD |
| P06756 | Integrin alpha-V | ITGAV | N/A |
| P06850 | Corticoliberin | CRH | N/A |
| P07101 | Tyrosine 3-monooxygenase | TH | N/A |
| P07204 | Thrombomodulin | THBD | N/A |
| P07333 | Macrophage colony-stimulating factor 1 receptor | CSF1R | 3DPK |
| P07437 | Tubulin beta chain | TUBB | N/A |
| P07550 | Beta-2 adrenergic receptor | ADRB2 | 3NY8 |
| P07766 | T-cell surface glycoprotein CD3 epsilon chain | CD3E | N/A |
| P07900 | Heat shock protein HSP 90-alpha | HSP90AA1 | 3HEK |
| P07947 | Tyrosine-protein kinase Yes | YES1 | N/A |
| P07948 | Tyrosine-protein kinase Lyn | LYN | 3A4O |
| P07949 | Proto-oncogene tyrosine-protein kinase receptor Ret | RET | 2X2K |
| P08069 | Insulin-like growth factor 1 receptor | IGF1R | 3I81 |
| P08183 | Multidrug resistance protein 1 | ABCB1 | 6FN1 |
| P08185 | Corticosteroid-binding globulin | SERPINA6 | N/A |
| P08235 | Mineralocorticoid receptor | NR3C2 | 2OAX |
| P08246 | Neutrophil elastase | ELANE | 1B0F |
| P08253 | 72 kDa type IV collagenase | MMP2 | 1HOV |
| P08581 | Hepatocyte growth factor receptor | MET | 3R7O |
| P08631 | Tyrosine-protein kinase HCK | HCK | 2C0I |
| P08637 | Low affinity immunoglobulin gamma Fc region receptor III-A | FCGR3A | N/A |
| P08684 | Cytochrome P450 3A4 | CYP3A4 | N/A |
| P08887 | Interleukin-6 receptor subunit alpha | IL6R | N/A |
| P08922 | Proto-oncogene tyrosine-protein kinase ROS | ROS1 | 3ZBF |
| P09038 | Fibroblast growth factor 2 | FGF2 | 1BFB |
| P09210 | Glutathione S-transferase A2 | GSTA2 | 2WJU |
| P09455 | Retinol-binding protein 1 | RBP1 | 5HBS |
| P09619 | Platelet-derived growth factor receptor beta | PDGFRB | N/A |
| P09693 | T-cell surface glycoprotein CD3 gamma chain | CD3G | N/A |
| P09871 | Complement C1s subcomponent | C1S | 1ELV |
| P09874 | Poly [ADP-ribose] polymerase 1 | PARP1 | 3L3L |
| P09884 | DNA polymerase alpha catalytic subunit | POLA1 | N/A |
| P09917 | Arachidonate 5-lipoxygenase | ALOX5 | 3V99 |
| P0C0L4 | Complement C4-A | C4A | N/A |
| P0C0L5 | Complement C4-B | C4B | N/A |
| P10109 | Adrenodoxin, mitochondrial | FDX1 | 3N9Y |
| P10145 | Interleukin-8 | CXCL8 | N/A |
| P10275 | Androgen receptor | AR | 3L3X |
| P10276 | Retinoic acid receptor alpha | RARA | 3KMR |
| P10415 | Apoptosis regulator Bcl-2 | BCL2 | 2W3L |
| P10632 | Cytochrome P450 2C8 | CYP2C8 | N/A |
| P10635 | Cytochrome P450 2D6 | CYP2D6 | N/A |
| P10636 | Microtubule-associated protein tau | MAPT | N/A |
| P10721 | Mast/stem cell growth factor receptor Kit | KIT | 3G0E |
| P10745 | Retinol-binding protein 3 | RBP3 | N/A |
| P10826 | Retinoic acid receptor beta | RARB | 1XAP |
| P10915 | Hyaluronan and proteoglycan link protein 1 | HAPLN1 | N/A |
| P11021 | Endoplasmic reticulum chaperone BiP | HSPA5 | 3LDP |
| P11137 | Microtubule-associated protein 2 | MAP2 | N/A |
| P11168 | Solute carrier family 2, facilitated glucose transporter member 2 | SLC2A2 | N/A |
| P11274 | Breakpoint cluster region protein | BCR | N/A |
| P11362 | Fibroblast growth factor receptor 1 | FGFR1 | 2FGI |
| P11387 | DNA topoisomerase 1 | TOP1 | 1T8I |
| P11388 | DNA topoisomerase 2-alpha | TOP2A | 1ZXM |
| P11473 | Vitamin D3 receptor | VDR | 1DB1 |
| P11509 | Cytochrome P450 2A6 | CYP2A6 | N/A |
| P11511 | Aromatase | CYP19A1 | 3EQM |
| P11712 | Cytochrome P450 2C9 | CYP2C9 | N/A |
| P11802 | Cyclin-dependent kinase 4 | CDK4 | N/A |
| P11836 | B-lymphocyte antigen CD20 | MS4A1 | N/A |
| P12004 | Proliferating cell nuclear antigen | PCNA | 3VKX |
| P12104 | Fatty acid-binding protein, intestinal | FABP2 | 3AKM |
| P12268 | Inosine-5'-monophosphate dehydrogenase 2 | IMPDH2 | 1NF7 |
| P12271 | Retinaldehyde-binding protein 1 | RLBP1 | 3HX3 |
| P12314 | High affinity immunoglobulin gamma Fc receptor I | FCGR1A | N/A |
| P12318 | Low affinity immunoglobulin gamma Fc region receptor II-a | FCGR2A | 3RY6 |
| P12319 | High affinity immunoglobulin epsilon receptor subunit alpha | FCER1A | N/A |
| P12931 | Proto-oncogene tyrosine-protein kinase Src | SRC | 2H8H |
| P13423 | Protective antigen | pagA | N/A |
| P13500 | C-C motif chemokine 2 | CCL2 | N/A |
| P13501 | C-C motif chemokine 5 | CCL5 | 1U4L |
| P13569 | Cystic fibrosis transmembrane conductance regulator | CFTR | 3GD7 |
| P13611 | Versican core protein | VCAN | N/A |
| P13612 | Integrin alpha-4 | ITGA4 | N/A |
| P13631 | Retinoic acid receptor gamma | RARG | 3LBD |
| P13716 | Delta-aminolevulinic acid dehydratase | ALAD | 1E51 |
| P13727 | Bone marrow proteoglycan | PRG2 | N/A |
| P13843 | Fusion glycoprotein F0 | F | N/A |
| P14060 | 3 beta-hydroxysteroid dehydrogenase/Delta 5-->4-isomerase type 1 | HSD3B1 | N/A |
| P14324 | Farnesyl pyrophosphate synthase | FDPS | 3N45 |
| P14555 | Phospholipase A2, membrane associated | PLA2G2A | 1J1A |
| P14635 | G2/mitotic-specific cyclin-B1 | CCNB1 | 4YC3 |
| P14778 | Interleukin-1 receptor type 1 | IL1R1 | N/A |
| P14780 | Matrix metalloproteinase-9 | MMP9 | 1GKC |
| P14784 | Interleukin-2 receptor subunit beta | IL2RB | N/A |
| P15056 | Serine/threonine-protein kinase B-raf | BRAF | 2FB8 |
| P15121 | Aldose reductase | AKR1B1 | 2PD5 |
| P15144 | Aminopeptidase N | ANPEP | 4FYR |
| P15153 | Ras-related C3 botulinum toxin substrate 2 | RAC2 | 2W2X |
| P15309 | Prostatic acid phosphatase | ACPP | 1ND5 |
| P15328 | Folate receptor alpha | FOLR1 | 5IZQ |
| P15391 | B-lymphocyte antigen CD19 | CD19 | N/A |
| P15509 | Granulocyte-macrophage colony-stimulating factor receptor subunit alpha | CSF2RA | N/A |
| P15538 | Cytochrome P450 11B1, mitochondrial | CYP11B1 | N/A |
| P15692 | Vascular endothelial growth factor A | VEGFA | 4KZN |
| P16070 | CD44 antigen | CD44 | N/A |
| P16220 | Cyclic AMP-responsive element-binding protein 1 | CREB1 | N/A |
| P16234 | Platelet-derived growth factor receptor alpha | PDGFRA | N/A |
| P16410 | Cytotoxic T-lymphocyte protein 4 | CTLA4 | N/A |
| P16581 | E-selectin | SELE | 1G1T |
| P17181 | Interferon alpha/beta receptor 1 | IFNAR1 | N/A |
| P17252 | Protein kinase C alpha type | PRKCA | 3IW4 |
| P17612 | cAMP-dependent protein kinase catalytic subunit alpha | PRKACA | 3POO |
| P17735 | Tyrosine aminotransferase | TAT | N/A |
| P17948 | Vascular endothelial growth factor receptor 1 | FLT1 | 3HNG |
| P18510 | Interleukin-1 receptor antagonist protein | IL1RN | N/A |
| P18858 | DNA ligase 1 | LIG1 | N/A |
| P19099 | Cytochrome P450 11B2, mitochondrial | CYP11B2 | 4ZGX |
| P19174 | 1-phosphatidylinositol 4,5-bisphosphate phosphodiesterase gamma-1 | PLCG1 | N/A |
| P19320 | Vascular cell adhesion protein 1 | VCAM1 | N/A |
| P19419 | ETS domain-containing protein Elk-1 | ELK1 | N/A |
| P19652 | Alpha-1-acid glycoprotein 2 | ORM2 | 3APW |
| P19793 | Retinoic acid receptor RXR-alpha | RXRA | 3KWY |
| P19838 | Nuclear factor NF-kappa-B p105 subunit | NFKB1 | N/A |
| P20138 | Myeloid cell surface antigen CD33 | CD33 | N/A |
| P20248 | Cyclin-A2 | CCNA2 | N/A |
| P20276 | 50S ribosomal protein L2 | rpl2 | N/A |
| P20292 | Arachidonate 5-lipoxygenase-activating protein | ALOX5AP | 2Q7R |
| P20333 | Tumor necrosis factor receptor superfamily member 1B | TNFRSF1B | N/A |
| P20618 | Proteasome subunit beta type-1 | PSMB1 | N/A |
| P20701 | Integrin alpha-L | ITGAL | 3BQM |
| P20813 | Cytochrome P450 2B6 | CYP2B6 | N/A |
| P20815 | Cytochrome P450 3A5 | CYP3A5 | 5VEU |
| P20839 | Inosine-5'-monophosphate dehydrogenase 1 | IMPDH1 | 1JCN |
| P20963 | T-cell surface glycoprotein CD3 zeta chain | CD247 | N/A |
| P21281 | V-type proton ATPase subunit B, brain isoform | ATP6V1B2 | N/A |
| P21462 | fMet-Leu-Phe receptor | FPR1 | N/A |
| P21802 | Fibroblast growth factor receptor 2 | FGFR2 | 3B2T |
| P22102 | Trifunctional purine biosynthetic protein adenosine-3 | GART | 1RBM |
| P22455 | Fibroblast growth factor receptor 4 | FGFR4 | N/A |
| P22607 | Fibroblast growth factor receptor 3 | FGFR3 | N/A |
| P22888 | Lutropin-choriogonadotropic hormone receptor | LHCGR | N/A |
| P23219 | Prostaglandin G/H synthase 1 | PTGS1 | 3N8X |
| P23258 | Tubulin gamma-1 chain | TUBG1 | 3CB2 |
| P23297 | Protein S100-A1 | S100A1 | N/A |
| P23458 | Tyrosine-protein kinase JAK1 | JAK1 | 3EYG |
| P23921 | Ribonucleoside-diphosphate reductase large subunit | RRM1 | 2WGH |
| P24024 | Protein P | P | N/A |
| P24385 | G1/S-specific cyclin-D1 | CCND1 | N/A |
| P24462 | Cytochrome P450 3A7 | CYP3A7 | N/A |
| P24557 | Thromboxane-A synthase | TBXAS1 | N/A |
| P24752 | Acetyl-CoA acetyltransferase, mitochondrial | ACAT1 | N/A |
| P24864 | G1/S-specific cyclin-E1 | CCNE1 | N/A |
| P24941 | Cyclin-dependent kinase 2 | CDK2 | 1GZ8 |
| P25024 | C-X-C chemokine receptor type 1 | CXCR1 | N/A |
| P25101 | Endothelin-1 receptor | EDNRA | N/A |
| P25445 | Tumor necrosis factor receptor superfamily member 6 | FAS | N/A |
| P25963 | NF-kappa-B inhibitor alpha | NFKBIA | N/A |
| P26010 | Integrin beta-7 | ITGB7 | 3V4V |
| P26358 | DNA (cytosine-5)-methyltransferase 1 | DNMT1 | 3SWR |
| P26439 | 3 beta-hydroxysteroid dehydrogenase/Delta 5-->4-isomerase type 2 | HSD3B2 | N/A |
| P26951 | Interleukin-3 receptor subunit alpha | IL3RA | N/A |
| P27361 | Mitogen-activated protein kinase 3 | MAPK3 | 2ZOQ |
| P27707 | Deoxycytidine kinase | DCK | 2ZI5 |
| P27815 | cAMP-specific 3',5'-cyclic phosphodiesterase 4A | PDE4A | 3I8V |
| P27816 | Microtubule-associated protein 4 | MAP4 | N/A |
| P28062 | Proteasome subunit beta type-8 | PSMB8 | N/A |
| P28065 | Proteasome subunit beta type-9 | PSMB9 | N/A |
| P28074 | Proteasome subunit beta type-5 | PSMB5 | N/A |
| P28482 | Mitogen-activated protein kinase 1 | MAPK1 | 3I5Z |
| P28702 | Retinoic acid receptor RXR-beta | RXRB | 1H9U |
| P28845 | Corticosteroid 11-beta-dehydrogenase isozyme 1 | HSD11B1 | 3EY4 |
| P28908 | Tumor necrosis factor receptor superfamily member 8 | TNFRSF8 | N/A |
| P29034 | Protein S100-A2 | S100A2 | N/A |
| P29317 | Ephrin type-A receptor 2 | EPHA2 | 1MQB |
| P29353 | SHC-transforming protein 1 | SHC1 | N/A |
| P29460 | Interleukin-12 subunit beta | IL12B | 1F42 |
| P29474 | Nitric oxide synthase, endothelial | NOS3 | 1M9J |
| P30085 | UMP-CMP kinase | CMPK1 | N/A |
| P30291 | Wee1-like protein kinase | WEE1 | 3CQE |
| P30405 | Peptidyl-prolyl cis-trans isomerase F, mitochondrial | PPIF | 3RDC |
| P30411 | B2 bradykinin receptor | BDKRB2 | N/A |
| P30968 | Gonadotropin-releasing hormone receptor | GNRHR | N/A |
| P31151 | Protein S100-A7 | S100A7 | 2WOR |
| P31260 | Homeobox protein Hox-A10 | HOXA10 | N/A |
| P31350 | Ribonucleoside-diphosphate reductase subunit M2 | RRM2 | N/A |
| P31358 | CAMPATH-1 antigen | CD52 | N/A |
| P31749 | RAC-alpha serine/threonine-protein kinase | AKT1 | 2UVM |
| P31785 | Cytokine receptor common subunit gamma | IL2RG | N/A |
| P31939 | Bifunctional purine biosynthesis protein PURH | ATIC | 1PKX |
| P31994 | Low affinity immunoglobulin gamma Fc region receptor II-b | FCGR2B | N/A |
| P31995 | Low affinity immunoglobulin gamma Fc region receptor II-c | FCGR2C | N/A |
| P32927 | Cytokine receptor common subunit beta | CSF2RB | N/A |
| P33151 | Cadherin-5 | CDH5 | N/A |
| P33261 | Cytochrome P450 2C19 | CYP2C19 | 4GQS |
| P33527 | Multidrug resistance-associated protein 1 | ABCC1 | N/A |
| P33681 | T-lymphocyte activation antigen CD80 | CD80 | N/A |
| P34741 | Syndecan-2 | SDC2 | N/A |
| P35222 | Catenin beta-1 | CTNNB1 | 3TX7 |
| P35228 | Nitric oxide synthase, inducible | NOS2 | 4NOS |
| P35354 | Prostaglandin G/H synthase 2 | PTGS2 | 3LN1 |
| P35367 | Histamine H1 receptor | HRH1 | 3RZE |
| P35499 | Sodium channel protein type 4 subunit alpha | SCN4A | N/A |
| P35869 | Aryl hydrocarbon receptor | AHR | N/A |
| P35916 | Vascular endothelial growth factor receptor 3 | FLT4 | N/A |
| P35968 | Vascular endothelial growth factor receptor 2 | KDR | 2QU5 |
| P36507 | Dual specificity mitogen-activated protein kinase kinase 2 | MAP2K2 | 1S9I |
| P36888 | Receptor-type tyrosine-protein kinase FLT3 | FLT3 | 5X02 |
| P36897 | TGF-beta receptor type-1 | TGFBR1 | 2X7O |
| P37231 | Peroxisome proliferator-activated receptor gamma | PPARG | 3H0A |
| P38398 | Breast cancer type 1 susceptibility protein | BRCA1 | N/A |
| P38936 | Cyclin-dependent kinase inhibitor 1 | CDKN1A | N/A |
| P39023 | 60S ribosomal protein L3 | RPL3 | N/A |
| P39900 | Macrophage metalloelastase | MMP12 | 3EHX |
| P40306 | Proteasome subunit beta type-10 | PSMB10 | N/A |
| P40763 | Signal transducer and activator of transcription 3 | STAT3 | N/A |
| P41440 | Folate transporter 1 | SLC19A1 | N/A |
| P41743 | Protein kinase C iota type | PRKCI | 1ZRZ |
| P42081 | T-lymphocyte activation antigen CD86 | CD86 | N/A |
| P42229 | Signal transducer and activator of transcription 5A | STAT5A | N/A |
| P42336 | Phosphatidylinositol 4,5-bisphosphate 3-kinase catalytic subunit alpha isoform | PIK3CA | 3HHM |
| P42345 | Serine/threonine-protein kinase mTOR | MTOR | 1FAP |
| P42574 | Caspase-3 | CASP3 | 1GFW |
| P42684 | Tyrosine-protein kinase ABL2 | ABL2 | 3GVU |
| P42685 | Tyrosine-protein kinase FRK | FRK | N/A |
| P42898 | Methylenetetrahydrofolate reductase | MTHFR | N/A |
| P45983 | Mitogen-activated protein kinase 8 | MAPK8 | 3PZE |
| P46527 | Cyclin-dependent kinase inhibitor 1B | CDKN1B | N/A |
| P46531 | Neurogenic locus notch homolog protein 1 | NOTCH1 | 4D0E |
| P46721 | Solute carrier organic anion transporter family member 1A2 | SLCO1A2 | N/A |
| P47712 | Cytosolic phospholipase A2 | PLA2G4A | 1CJY |
| P47895 | Aldehyde dehydrogenase family 1 member A3 | ALDH1A3 | N/A |
| P48023 | Tumor necrosis factor ligand superfamily member 6 | FASLG | N/A |
| P48048 | ATP-sensitive inward rectifier potassium channel 1 | KCNJ1 | N/A |
| P48443 | Retinoic acid receptor RXR-gamma | RXRG | N/A |
| P48551 | Interferon alpha/beta receptor 2 | IFNAR2 | N/A |
| P49069 | Calcium signal-modulating cyclophilin ligand | CAMLG | N/A |
| P49238 | CX3C chemokine receptor 1 | CX3CR1 | N/A |
| P49721 | Proteasome subunit beta type-2 | PSMB2 | N/A |
| P49763 | Placenta growth factor | PGF | 1RV6 |
| P49765 | Vascular endothelial growth factor B | VEGFB | N/A |
| P49767 | Vascular endothelial growth factor C | VEGFC | 2X1W |
| P49788 | Retinoic acid receptor responder protein 1 | RARRES1 | N/A |
| P49815 | Tuberin | TSC2 | N/A |
| P49840 | Glycogen synthase kinase-3 alpha | GSK3A | N/A |
| P49841 | Glycogen synthase kinase-3 beta | GSK3B | 3I4B |
| P49916 | DNA ligase 3 | LIG3 | N/A |
| P50591 | Tumor necrosis factor ligand superfamily member 10 | TNFSF10 | N/A |
| P50613 | Cyclin-dependent kinase 7 | CDK7 | 1UA2 |
| P51580 | Thiopurine S-methyltransferase | TPMT | 2H11 |
| P51692 | Signal transducer and activator of transcription 5B | STAT5B | N/A |
| P51800 | Chloride channel protein ClC-Ka | CLCNKA | N/A |
| P51812 | Ribosomal protein S6 kinase alpha-3 | RPS6KA3 | 4D9T |
| P51843 | Nuclear receptor subfamily 0 group B member 1 | NR0B1 | N/A |
| P51946 | Cyclin-H | CCNH | N/A |
| P52209 | 6-phosphogluconate dehydrogenase, decarboxylating | PGD | 4GWK |
| P52333 | Tyrosine-protein kinase JAK3 | JAK3 | 3PJC |
| P53350 | Serine/threonine-protein kinase PLK1 | PLK1 | 4A4L |
| P53667 | LIM domain kinase 1 | LIMK1 | 3S95 |
| P53778 | Mitogen-activated protein kinase 12 | MAPK12 | 1CM8 |
| P53985 | Monocarboxylate transporter 1 | SLC16A1 | N/A |
| P54619 | 5'-AMP-activated protein kinase subunit gamma-1 | PRKAG1 | 2UV5 |
| P54646 | 5'-AMP-activated protein kinase catalytic subunit alpha-2 | PRKAA2 | 3AQV |
| P55008 | Allograft inflammatory factor 1 | AIF1 | N/A |
| P55210 | Caspase-7 | CASP7 | 1SHL |
| P55211 | Caspase-9 | CASP9 | 2AR9 |
| P55212 | Caspase-6 | CASP6 | 4NBL |
| P55957 | BH3-interacting domain death agonist | BID | N/A |
| P56282 | DNA polymerase epsilon subunit 2 | POLE2 | N/A |
| P56524 | Histone deacetylase 4 | HDAC4 | 2VQV |
| P57059 | Serine/threonine-protein kinase SIK1 | SIK1 | N/A |
| P60484 | Phosphatidylinositol 3,4,5-trisphosphate 3-phosphatase and dual-specificity protein phosphatase PTEN | PTEN | 5BZZ |
| P60568 | Interleukin-2 | IL2 | 1PW6 |
| P60763 | Ras-related C3 botulinum toxin substrate 3 | RAC3 | 2QME |
| P60953 | Cell division control protein 42 homolog | CDC42 | 1NF3 |
| P61073 | C-X-C chemokine receptor type 4 | CXCR4 | 3ODU |
| P62508 | Estrogen-related receptor gamma | ESRRG | 2P7A |
| P62937 | Peptidyl-prolyl cis-trans isomerase A | PPIA | 1W8L |
| P62942 | Peptidyl-prolyl cis-trans isomerase FKBP1A | FKBP1A | 2DG3 |
| P62993 | Growth factor receptor-bound protein 2 | GRB2 | 2H5K |
| P63000 | Ras-related C3 botulinum toxin substrate 1 | RAC1 | 3SBE |
| P68366 | Tubulin alpha-4A chain | TUBA4A | N/A |
| P78348 | Acid-sensing ion channel 1 | ASIC1 | N/A |
| P78559 | Microtubule-associated protein 1A | MAP1A | N/A |
| P80365 | Corticosteroid 11-beta-dehydrogenase isozyme 2 | HSD11B2 | N/A |
| P80511 | Protein S100-A12 | S100A12 | 2WC8 |
| P84022 | Mothers against decapentaplegic homolog 3 | SMAD3 | N/A |
| P84077 | ADP-ribosylation factor 1 | ARF1 | 1RE0 |
| P98066 | Tumor necrosis factor-inducible gene 6 protein | TNFAIP6 | N/A |
| P98170 | E3 ubiquitin-protein ligase XIAP | XIAP | 3HL5 |
| Q00534 | Cyclin-dependent kinase 6 | CDK6 | 3NUP |
| Q00535 | Cyclin-dependent-like kinase 5 | CDK5 | 3O0G |
| Q00653 | Nuclear factor NF-kappa-B p100 subunit | NFKB2 | N/A |
| Q00987 | E3 ubiquitin-protein ligase Mdm2 | MDM2 | 3TU1 |
| Q01094 | Transcription factor E2F1 | E2F1 | N/A |
| Q01362 | High affinity immunoglobulin epsilon receptor subunit beta | MS4A2 | N/A |
| Q01718 | Adrenocorticotropic hormone receptor | MC2R | N/A |
| Q02127 | Dihydroorotate dehydrogenase (quinone), mitochondrial | DHODH | 3F1Q |
| Q02156 | Protein kinase C epsilon type | PRKCE | N/A |
| Q02750 | Dual specificity mitogen-activated protein kinase kinase 1 | MAP2K1 | 3DY7 |
| Q02763 | Angiopoietin-1 receptor | TEK | 3L8P |
| Q02880 | DNA topoisomerase 2-beta | TOP2B | 3QX3 |
| Q02928 | Cytochrome P450 4A11 | CYP4A11 | N/A |
| Q03135 | Caveolin-1 | CAV1 | N/A |
| Q03181 | Peroxisome proliferator-activated receptor delta | PPARD | 3GZ9 |
| Q04759 | Protein kinase C theta type | PRKCQ | 1XJD |
| Q04760 | Lactoylglutathione lyase | GLO1 | 1QIN |
| Q04828 | Aldo-keto reductase family 1 member C1 | AKR1C1 | 3GUG |
| Q05397 | Focal adhesion kinase 1 | PTK2 | 3PXK |
| Q05513 | Protein kinase C zeta type | PRKCZ | N/A |
| Q05655 | Protein kinase C delta type | PRKCD | N/A |
| Q05932 | Folylpolyglutamate synthase, mitochondrial | FPGS | N/A |
| Q06187 | Tyrosine-protein kinase BTK | BTK | 3PIX |
| Q06203 | Amidophosphoribosyltransferase | PPAT | N/A |
| Q06278 | Aldehyde oxidase | AOX1 | 4UHX |
| Q07021 | Complement component 1 Q subcomponent-binding protein, mitochondrial | C1QBP | N/A |
| Q07343 | cAMP-specific 3',5'-cyclic phosphodiesterase 4B | PDE4B | 1XLX |
| Q07812 | Apoptosis regulator BAX | BAX | N/A |
| Q07817 | Bcl-2-like protein 1 | BCL2L1 | 3QKD |
| Q07864 | DNA polymerase epsilon catalytic subunit A | POLE | N/A |
| Q07869 | Peroxisome proliferator-activated receptor alpha | PPARA | 1I7G |
| Q07889 | Son of sevenless homolog 1 | SOS1 | 4URX |
| Q07973 | 1,25-dihydroxyvitamin D(3) 24-hydroxylase, mitochondrial | CYP24A1 | N/A |
| Q08345 | Epithelial discoidin domain-containing receptor 1 | DDR1 | 3ZOS |
| Q08499 | cAMP-specific 3',5'-cyclic phosphodiesterase 4D | PDE4D | 3G4K |
| Q08881 | Tyrosine-protein kinase ITK/TSK | ITK | 3T9T |
| Q09428 | ATP-binding cassette sub-family C member 8 | ABCC8 | N/A |
| Q12778 | Forkhead box protein O1 | FOXO1 | N/A |
| Q12809 | Potassium voltage-gated channel subfamily H member 2 | KCNH2 | N/A |
| Q12866 | Tyrosine-protein kinase Mer | MERTK | 3BPR |
| Q12908 | Ileal sodium/bile acid cotransporter | SLC10A2 | N/A |
| Q13131 | 5'-AMP-activated protein kinase catalytic subunit alpha-1 | PRKAA1 | 5EZV |
| Q13158 | FAS-associated death domain protein | FADD | N/A |
| Q13258 | Prostaglandin D2 receptor | PTGDR | N/A |
| Q13315 | Serine-protein kinase ATM | ATM | N/A |
| Q13464 | Rho-associated protein kinase 1 | ROCK1 | 2ESM |
| Q13485 | Mothers against decapentaplegic homolog 4 | SMAD4 | N/A |
| Q13489 | Baculoviral IAP repeat-containing protein 3 | BIRC3 | N/A |
| Q13490 | Baculoviral IAP repeat-containing protein 2 | BIRC2 | 3MUP |
| Q13509 | Tubulin beta-3 chain | TUBB3 | N/A |
| Q13535 | Serine/threonine-protein kinase ATR | ATR | N/A |
| Q13547 | Histone deacetylase 1 | HDAC1 | N/A |
| Q13555 | Calcium/calmodulin-dependent protein kinase type II subunit gamma | CAMK2G | 2V7O |
| Q13882 | Protein-tyrosine kinase 6 | PTK6 | N/A |
| Q13946 | High affinity cAMP-specific 3',5'-cyclic phosphodiesterase 7A | PDE7A | 1ZKL |
| Q14005 | Pro-interleukin-16 | IL16 | N/A |
| Q14094 | Cyclin-I | CCNI | N/A |
| Q14145 | Kelch-like ECH-associated protein 1 | KEAP1 | N/A |
| Q14181 | DNA polymerase alpha subunit B | POLA2 | N/A |
| Q14289 | Protein-tyrosine kinase 2-beta | PTK2B | 3FZS |
| Q14432 | cGMP-inhibited 3',5'-cyclic phosphodiesterase A | PDE3A | N/A |
| Q14520 | Hyaluronan-binding protein 2 | HABP2 | N/A |
| Q14534 | Squalene monooxygenase | SQLE | N/A |
| Q14626 | Interleukin-11 receptor subunit alpha | IL11RA | N/A |
| Q14654 | ATP-sensitive inward rectifier potassium channel 11 | KCNJ11 | N/A |
| Q14790 | Caspase-8 | CASP8 | 3KJN |
| Q14973 | Sodium/bile acid cotransporter | SLC10A1 | N/A |
| Q15116 | Programmed cell death protein 1 | PDCD1 | N/A |
| Q15125 | 3-beta-hydroxysteroid-Delta(8),Delta(7)-isomerase | EBP | N/A |
| Q15303 | Receptor tyrosine-protein kinase erbB-4 | ERBB4 | 2R4B |
| Q15418 | Ribosomal protein S6 kinase alpha-1 | RPS6KA1 | 2Z7R |
| Q15759 | Mitogen-activated protein kinase 11 | MAPK11 | 3GC9 |
| Q16539 | Mitogen-activated protein kinase 14 | MAPK14 | 1KV1 |
| Q16552 | Interleukin-17A | IL17A | N/A |
| Q16611 | Bcl-2 homologous antagonist/killer | BAK1 | 2LP8 |
| Q16647 | Prostacyclin synthase | PTGIS | 3B6H |
| Q16678 | Cytochrome P450 1B1 | CYP1B1 | N/A |
| Q16696 | Cytochrome P450 2A13 | CYP2A13 | N/A |
| Q16832 | Discoidin domain-containing receptor 2 | DDR2 | N/A |
| Q16881 | Thioredoxin reductase 1, cytoplasmic | TXNRD1 | 2ZZB |
| Q4U2R8 | Solute carrier family 22 member 6 | SLC22A6 | N/A |
| Q51911 | Peptostreptococcal albumin-binding protein | pab | N/A |
| Q53ET0 | CREB-regulated transcription coactivator 2 | CRTC2 | N/A |
| Q5JVS0 | Intracellular hyaluronan-binding protein 4 | HABP4 | N/A |
| Q5L2G3 | Uncharacterized oxidoreductase CzcO-like | GK0582 | N/A |
| Q5T3U5 | Multidrug resistance-associated protein 7 | ABCC10 | N/A |
| Q69600 | HBsAg | S gene | N/A |
| Q6NUM9 | All-trans-retinol 13,14-reductase | RETSAT | N/A |
| Q6UUV9 | CREB-regulated transcription coactivator 1 | CRTC1 | N/A |
| Q6UX15 | Layilin | LAYN | N/A |
| Q6UXS9 | Inactive caspase-12 | CASP12 | N/A |
| Q6ZQN7 | Solute carrier organic anion transporter family member 4C1 | SLCO4C1 | N/A |
| Q71U36 | Tubulin alpha-1A chain | TUBA1A | N/A |
| Q7LG56 | Ribonucleoside-diphosphate reductase subunit M2 B | RRM2B | N/A |
| Q86UW8 | Hyaluronan and proteoglycan link protein 4 | HAPLN4 | N/A |
| Q89ZI2 | O-GlcNAcase BT_4395 | BT_4395 | 2XJ7 |
| Q8MU52 | Glutathione S-transferase | GST | 2AAW |
| Q8N8N7 | Prostaglandin reductase 2 | PTGR2 | 2W4Q |
| Q8NBN7 | Retinol dehydrogenase 13 | RDH13 | N/A |
| Q8NFJ5 | Retinoic acid-induced protein 3 | GPRC5A | N/A |
| Q8NG66 | Serine/threonine-protein kinase Nek11 | NEK11 | N/A |
| Q8TC12 | Retinol dehydrogenase 11 | RDH11 | N/A |
| Q8TCC7 | Solute carrier family 22 member 8 | SLC22A8 | N/A |
| Q8WUI4 | Histone deacetylase 7 | HDAC7 | 3C0Z |
| Q8WWQ8 | Stabilin-2 | STAB2 | N/A |
| Q92574 | Hamartin | TSC1 | N/A |
| Q92637 | High affinity immunoglobulin gamma Fc receptor IB | FCGR1B | N/A |
| Q92731 | Estrogen receptor beta | ESR2 | 2QTU |
| Q92769 | Histone deacetylase 2 | HDAC2 | 3MAX |
| Q92781 | 11-cis retinol dehydrogenase | RDH5 | N/A |
| Q92820 | Gamma-glutamyl hydrolase | GGH | N/A |
| Q92887 | Canalicular multispecific organic anion transporter 1 | ABCC2 | N/A |
| Q92934 | Bcl2-associated agonist of cell death | BAD | N/A |
| Q95460 | Major histocompatibility complex class I-related gene protein | MR1 | 4GUP |
| Q969P6 | DNA topoisomerase I, mitochondrial | TOP1MT | N/A |
| Q969S8 | Histone deacetylase 10 | HDAC10 | N/A |
| Q96J66 | ATP-binding cassette sub-family C member 11 | ABCC11 | N/A |
| Q96LZ3 | Calcineurin subunit B type 2 | PPP3R2 | N/A |
| Q96NR8 | Retinol dehydrogenase 12 | RDH12 | N/A |
| Q96NT5 | Proton-coupled folate transporter | SLC46A1 | N/A |
| Q96S86 | Hyaluronan and proteoglycan link protein 3 | HAPLN3 | N/A |
| Q96SW2 | Protein cereblon | CRBN | N/A |
| Q99062 | Granulocyte colony-stimulating factor receptor | CSF3R | N/A |
| Q99584 | Protein S100-A13 | S100A13 | 2KOT |
| Q99683 | Mitogen-activated protein kinase kinase kinase 5 | MAP3K5 | 2CLQ |
| Q99835 | Smoothened homolog | SMO | N/A |
| Q9BTZ2 | Dehydrogenase/reductase SDR family member 4 | DHRS4 | 3O4R |
| Q9BUB5 | MAP kinase-interacting serine/threonine-protein kinase 1 | MKNK1 | N/A |
| Q9BY41 | Histone deacetylase 8 | HDAC8 | 1T69 |
| Q9BZV3 | Interphotoreceptor matrix proteoglycan 2 | IMPG2 | N/A |
| Q9H169 | Stathmin-4 | STMN4 | N/A |
| Q9H228 | Sphingosine 1-phosphate receptor 5 | S1PR5 | N/A |
| Q9H4B7 | Tubulin beta-1 chain | TUBB1 | N/A |
| Q9HB55 | Cytochrome P450 3A43 | CYP3A43 | N/A |
| Q9HBH5 | Retinol dehydrogenase 14 | RDH14 | N/A |
| Q9HBH9 | MAP kinase-interacting serine/threonine-protein kinase 2 | MKNK2 | 2HW7 |
| Q9NP31 | SH2 domain-containing protein 2A | SH2D2A | N/A |
| Q9NPD5 | Solute carrier organic anion transporter family member 1B3 | SLCO1B3 | N/A |
| Q9NPF7 | Interleukin-23 subunit alpha | IL23A | N/A |
| Q9NR09 | Baculoviral IAP repeat-containing protein 6 | BIRC6 | N/A |
| Q9NR28 | Diablo homolog, mitochondrial | DIABLO | 4TX5 |
| Q9NR33 | DNA polymerase epsilon subunit 4 | POLE4 | N/A |
| Q9NR96 | Toll-like receptor 9 | TLR9 | N/A |
| Q9NR97 | Toll-like receptor 8 | TLR8 | N/A |
| Q9NRF9 | DNA polymerase epsilon subunit 3 | POLE3 | N/A |
| Q9NS75 | Cysteinyl leukotriene receptor 2 | CYSLTR2 | N/A |
| Q9NSA0 | Solute carrier family 22 member 11 | SLC22A11 | N/A |
| Q9NSD9 | Phenylalanine--tRNA ligase beta subunit | FARSB | N/A |
| Q9NYB5 | Solute carrier organic anion transporter family member 1C1 | SLCO1C1 | N/A |
| Q9NYK1 | Toll-like receptor 7 | TLR7 | N/A |
| Q9NYR8 | Retinol dehydrogenase 8 | RDH8 | N/A |
| Q9NZK7 | Group IIE secretory phospholipase A2 | PLA2G2E | N/A |
| Q9QZR5 | Homeodomain-interacting protein kinase 2 | Hipk2 | N/A |
| Q9S427 | Protein-tyrosine-phosphatase | PPI | N/A |
| Q9UBN7 | Histone deacetylase 6 | HDAC6 | N/A |
| Q9UGI9 | 5'-AMP-activated protein kinase subunit gamma-3 | PRKAG3 | N/A |
| Q9UGJ0 | 5'-AMP-activated protein kinase subunit gamma-2 | PRKAG2 | N/A |
| Q9UGN5 | Poly [ADP-ribose] polymerase 2 | PARP2 | 3KJD |
| Q9UHI5 | Large neutral amino acids transporter small subunit 2 | SLC7A8 | N/A |
| Q9UIG8 | Solute carrier organic anion transporter family member 3A1 | SLCO3A1 | N/A |
| Q9UJT0 | Tubulin epsilon chain | TUBE1 | N/A |
| Q9UJT1 | Tubulin delta chain | TUBD1 | N/A |
| Q9UKV0 | Histone deacetylase 9 | HDAC9 | N/A |
| Q9UM73 | ALK tyrosine kinase receptor | ALK | 3LCS |
| Q9UNQ0 | ATP-binding cassette sub-family G member 2 | ABCG2 | 6ETI |
| Q9UPY5 | Cystine/glutamate transporter | SLC7A11 | N/A |
| Q9UQL6 | Histone deacetylase 5 | HDAC5 | N/A |
| Q9UQQ2 | SH2B adapter protein 3 | SH2B3 | N/A |
| Q9Y271 | Cysteinyl leukotriene receptor 1 | CYSLTR1 | N/A |
| Q9Y275 | Tumor necrosis factor ligand superfamily member 13B | TNFSF13B | N/A |
| Q9Y285 | Phenylalanine--tRNA ligase alpha subunit | FARSA | N/A |
| Q9Y2U5 | Mitogen-activated protein kinase kinase kinase 2 | MAP3K2 | N/A |
| Q9Y478 | 5'-AMP-activated protein kinase subunit beta-1 | PRKAB1 | N/A |
| Q9Y5Y4 | Prostaglandin D2 receptor 2 | PTGDR2 | N/A |
| Q9Y694 | Solute carrier family 22 member 7 | SLC22A7 | N/A |
| Q9Y6F1 | Poly [ADP-ribose] polymerase 3 | PARP3 | 3CE0 |
| Q9Y6L6 | Solute carrier organic anion transporter family member 1B1 | SLCO1B1 | N/A |
