# Supplementary material for: Chinese Medicine for Psoriasis Vulgaris Based on Syndrome Pattern: A Network Pharmacological Study
Source: Evid Based Complement Alternat Med. 2020 Apr 28;2020:5239854. doi: 10.1155/2020/5239854 (PMC7204377; doi:10.1155/2020/5239854)
Supplement: Supplementary Materials — Figure S1: ADME/T properties of compounds of three TCM formulae. Table S1: herb composition and compounds of each formula retrieved from PDTCM. Table S2: psoriasis-related proteins. Table S3: compound-target binding energy lower than −12.28 kcal/mol. Table S4: GSEA results for three TCM formulae. [file 5239854.f1.zip › 5239854.f1/Table S3.docx]

**Table S3: Compound-target binding energy lower than -12.28 kcal/mol.**

| **Target** | **PDB** | **Compound** | **Binding energy (kcal/mol)** |
| --- | --- | --- | --- |
| P10109 | 3N9Y | M011831 | -16.68 |
| P05108 | 3NA0 | M011831 | -16.48 |
| P27815 | 3I8V | M019624 | -16.27 |
| O67135 | 1C3R | M018782 | -15.85 |
| P05093 | 3RUK | M011831 | -15.82 |
| Q08499 | 3G4K | M018782 | -15.67 |
| P39900 | 3EHX | M015955 | -15.64 |
| P10109 | 3N9Y | M004519 | -15.63 |
| P05108 | 3NA0 | M004519 | -15.58 |
| P27815 | 3I8V | M018782 | -15.51 |
| Q07343 | 1XLX | M019624 | -15.51 |
| P05108 | 3NA0 | M013387 | -15.47 |
| P10109 | 3N9Y | M013387 | -15.33 |
| P00813 | 3IAR | M019624 | -15.23 |
| P10109 | 3N9Y | M001194 | -15.23 |
| P14780 | 1GKC | M020429 | -15.11 |
| P35968 | 2QU5 | M030533 | -15.11 |
| P19099 | 4ZGX | M009135 | -15.1 |
| P19099 | 4ZGX | M008651 | -15.08 |
| P35968 | 2QU5 | M004402 | -15.08 |
| P27815 | 3I8V | M019628 | -15.05 |
| P14780 | 1GKC | M015955 | -14.96 |
| P14780 | 1GKC | M019201 | -14.94 |
| P19099 | 4ZGX | M012298 | -14.87 |
| P27815 | 3I8V | M020032 | -14.84 |
| P14780 | 1GKC | M019628 | -14.83 |
| Q07343 | 1XLX | M018782 | -14.83 |
| P19099 | 4ZGX | M002163 | -14.8 |
| Q08499 | 3G4K | M020032 | -14.77 |
| P19099 | 4ZGX | M023398 | -14.73 |
| P02741 | 3L2Y | M019624 | -14.71 |
| O67135 | 1C3R | M010231 | -14.67 |
| P10109 | 3N9Y | M012419 | -14.67 |
| P05108 | 3NA0 | M012419 | -14.58 |
| P56524 | 2VQV | M020429 | -14.57 |
| P05108 | 3NA0 | M001194 | -14.55 |
| P05093 | 3RUK | M004519 | -14.52 |
| P39900 | 3EHX | M020429 | -14.51 |
| P10109 | 3N9Y | M007237 | -14.5 |
| Q07343 | 1XLX | M019628 | -14.5 |
| P10109 | 3N9Y | M018967 | -14.49 |
| P05093 | 3RUK | M013387 | -14.48 |
| P84077 | 1RE0 | M014384 | -14.47 |
| P14555 | 1J1A | M003399 | -14.46 |
| Q08499 | 3G4K | M019628 | -14.45 |
| P03956 | 966C | M026864 | -14.44 |
| P05108 | 3NA0 | M004987 | -14.44 |
| P07550 | 3NY8 | M018782 | -14.44 |
| P10109 | 3N9Y | M027425 | -14.42 |
| P07550 | 3NY8 | M019628 | -14.39 |
| P12271 | 3HX3 | M014132 | -14.38 |
| P19099 | 4ZGX | M009523 | -14.36 |
| P19099 | 4ZGX | M000604 | -14.34 |
| P35968 | 2QU5 | M010231 | -14.34 |
| P11388 | 1ZXM | M032128 | -14.33 |
| P14780 | 1GKC | M020032 | -14.32 |
| P10109 | 3N9Y | M032128 | -14.3 |
| P12271 | 3HX3 | M002163 | -14.3 |
| Q9UNQ0 | 6ETI | M020429 | -14.29 |
| P27815 | 3I8V | M020429 | -14.28 |
| P56524 | 2VQV | M004402 | -14.28 |
| P56524 | 2VQV | M023823 | -14.23 |
| P05093 | 3RUK | M016907 | -14.21 |
| P35968 | 2QU5 | M012713 | -14.19 |
| P04798 | 4I8V | M020621 | -14.18 |
| P12271 | 3HX3 | M013272 | -14.18 |
| P19099 | 4ZGX | M010708 | -14.18 |
| P07550 | 3NY8 | M020429 | -14.17 |
| P15121 | 2PD5 | M014132 | -14.17 |
| P11388 | 1ZXM | M021137 | -14.16 |
| P05108 | 3NA0 | M007237 | -14.15 |
| P28845 | 3EY4 | M011674 | -14.13 |
| P35968 | 2QU5 | M024929 | -14.13 |
| P19099 | 4ZGX | M016360 | -14.12 |
| P03956 | 966C | M015561 | -14.11 |
| P05108 | 3NA0 | M003237 | -14.1 |
| P11388 | 1ZXM | M021602 | -14.1 |
| P19099 | 4ZGX | M009938 | -14.1 |
| P03956 | 966C | M019201 | -14.09 |
| P15328 | 5IZQ | M019201 | -14.09 |
| P10109 | 3N9Y | M019816 | -14.08 |
| P19099 | 4ZGX | M033909 | -14.08 |
| P03956 | 966C | M015955 | -14.07 |
| P03956 | 966C | M028420 | -14.06 |
| P28845 | 3EY4 | M004977 | -14.06 |
| P56524 | 2VQV | M012713 | -14.06 |
| P11388 | 1ZXM | M028991 | -14.04 |
| O67135 | 1C3R | M022421 | -14.03 |
| Q8N8N7 | 2W4Q | M013211 | -14.02 |
| P19099 | 4ZGX | M011032 | -14 |
| P08253 | 1HOV | M026864 | -13.97 |
| P39900 | 3EHX | M024435 | -13.97 |
| P42684 | 3GVU | M030533 | -13.97 |
| P10109 | 3N9Y | M013452 | -13.95 |
| P03956 | 966C | M024435 | -13.94 |
| P05108 | 3NA0 | M027425 | -13.94 |
| P05093 | 3RUK | M023877 | -13.93 |
| P12271 | 3HX3 | M031368 | -13.93 |
| O14727 | 1Z6T | M020429 | -13.92 |
| P13569 | 3GD7 | M014384 | -13.92 |
| P04798 | 4I8V | M026833 | -13.91 |
| P10109 | 3N9Y | M004287 | -13.9 |
| P11388 | 1ZXM | M027170 | -13.9 |
| P12271 | 3HX3 | M011995 | -13.9 |
| P39900 | 3EHX | M019628 | -13.9 |
| P04150 | 3H52 | M014384 | -13.89 |
| P05108 | 3NA0 | M016909 | -13.89 |
| P29474 | 1M9J | M020429 | -13.89 |
| P51580 | 2H11 | M023863 | -13.88 |
| P03956 | 966C | M026833 | -13.87 |
| P15144 | 4FYR | M019624 | -13.87 |
| P03956 | 966C | M002205 | -13.86 |
| P04798 | 4I8V | M002163 | -13.85 |
| P07550 | 3NY8 | M020032 | -13.85 |
| Q07343 | 1XLX | M020032 | -13.85 |
| P19099 | 4ZGX | M004842 | -13.84 |
| P10109 | 3N9Y | M021322 | -13.82 |
| Q9UGN5 | 3KJD | M018782 | -13.82 |
| O67135 | 1C3R | M021319 | -13.81 |
| P03956 | 966C | M020429 | -13.81 |
| Q9UGN5 | 3KJD | M013452 | -13.81 |
| P04798 | 4I8V | M030514 | -13.8 |
| P10109 | 3N9Y | M001191 | -13.8 |
| P19099 | 4ZGX | M018092 | -13.8 |
| P11473 | 1DB1 | M011284 | -13.79 |
| Q08499 | 3G4K | M024765 | -13.79 |
| P19099 | 4ZGX | M008793 | -13.78 |
| P19099 | 4ZGX | M028060 | -13.78 |
| P39900 | 3EHX | M008651 | -13.78 |
| P39900 | 3EHX | M019201 | -13.78 |
| P56524 | 2VQV | M004329 | -13.78 |
| O67135 | 1C3R | M008651 | -13.77 |
| P19099 | 4ZGX | M002520 | -13.77 |
| P10109 | 3N9Y | M011284 | -13.76 |
| P12271 | 3HX3 | M029426 | -13.76 |
| P19099 | 4ZGX | M023863 | -13.76 |
| P19099 | 4ZGX | M034061 | -13.76 |
| Q07343 | 1XLX | M020429 | -13.76 |
| Q13946 | 1ZKL | M021732 | -13.76 |
| P08235 | 2OAX | M003009 | -13.75 |
| P10109 | 3N9Y | M021065 | -13.75 |
| P12271 | 3HX3 | M002520 | -13.75 |
| P04798 | 4I8V | M027665 | -13.74 |
| P11388 | 1ZXM | M018967 | -13.74 |
| P05108 | 3NA0 | M001533 | -13.73 |
| P05108 | 3NA0 | M018901 | -13.73 |
| P07550 | 3NY8 | M011161 | -13.73 |
| P05108 | 3NA0 | M018967 | -13.72 |
| P19099 | 4ZGX | M031236 | -13.72 |
| P35968 | 2QU5 | M003669 | -13.72 |
| Q08499 | 3G4K | M015955 | -13.72 |
| O67135 | 1C3R | M020032 | -13.71 |
| P08253 | 1HOV | M030533 | -13.71 |
| P11388 | 1ZXM | M011284 | -13.71 |
| P04150 | 3H52 | M015349 | -13.7 |
| P14555 | 1J1A | M013353 | -13.7 |
| P19099 | 4ZGX | M009447 | -13.7 |
| Q13490 | 3MUP | M002414 | -13.7 |
| P03956 | 966C | M007243 | -13.69 |
| P04798 | 4I8V | M022651 | -13.69 |
| P11388 | 1ZXM | M019381 | -13.68 |
| Q9UNQ0 | 6ETI | M011831 | -13.68 |
| P04150 | 3H52 | M033013 | -13.67 |
| P07550 | 3NY8 | M024765 | -13.67 |
| P26010 | 3V4V | M019624 | -13.67 |
| P39900 | 3EHX | M020032 | -13.67 |
| Q07343 | 1XLX | M015955 | -13.67 |
| Q08499 | 3G4K | M020429 | -13.66 |
| P03956 | 966C | M025645 | -13.65 |
| P04798 | 4I8V | M004115 | -13.65 |
| P08631 | 2C0I | M030533 | -13.65 |
| P28845 | 3EY4 | M025306 | -13.65 |
| P39900 | 3EHX | M008481 | -13.65 |
| P56524 | 2VQV | M010708 | -13.65 |
| P03956 | 966C | M023823 | -13.64 |
| P05093 | 3RUK | M001194 | -13.64 |
| P12271 | 3HX3 | M000208 | -13.64 |
| P14555 | 1J1A | M013387 | -13.64 |
| P19099 | 4ZGX | M010568 | -13.64 |
| P28845 | 3EY4 | M014384 | -13.64 |
| Q9UNQ0 | 6ETI | M015955 | -13.64 |
| O14727 | 1Z6T | M027653 | -13.63 |
| O75469 | 1M13 | M023823 | -13.62 |
| P15121 | 2PD5 | M005571 | -13.62 |
| P28845 | 3EY4 | M007966 | -13.62 |
| O67135 | 1C3R | M019628 | -13.61 |
| P05108 | 3NA0 | M019381 | -13.61 |
| P19099 | 4ZGX | M025643 | -13.6 |
| P28845 | 3EY4 | M014422 | -13.6 |
| P84077 | 1RE0 | M027649 | -13.6 |
| P12271 | 3HX3 | M028420 | -13.59 |
| P27707 | 2ZI5 | M013211 | -13.59 |
| P35968 | 2QU5 | M004329 | -13.59 |
| O67135 | 1C3R | M027649 | -13.58 |
| P03956 | 966C | M001683 | -13.58 |
| P28845 | 3EY4 | M010214 | -13.58 |
| P28845 | 3EY4 | M014763 | -13.58 |
| Q9UNQ0 | 6ETI | M019628 | -13.57 |
| P11388 | 1ZXM | M021322 | -13.56 |
| P12271 | 3HX3 | M001683 | -13.56 |
| P28845 | 3EY4 | M028941 | -13.56 |
| P28845 | 3EY4 | M033013 | -13.56 |
| P03956 | 966C | M008481 | -13.55 |
| P13631 | 3LBD | M006924 | -13.55 |
| P05108 | 3NA0 | M022800 | -13.53 |
| P28845 | 3EY4 | M031368 | -13.53 |
| P06401 | 2W8Y | M004842 | -13.52 |
| P11387 | 1T8I | M019201 | -13.52 |
| P12271 | 3HX3 | M010568 | -13.52 |
| P84077 | 1RE0 | M015349 | -13.52 |
| P03956 | 966C | M004752 | -13.51 |
| P08253 | 1HOV | M018782 | -13.51 |
| P11388 | 1ZXM | M004287 | -13.51 |
| P28845 | 3EY4 | M024666 | -13.51 |
| P35367 | 3RZE | M023398 | -13.51 |
| P35968 | 2QU5 | M026099 | -13.51 |
| P11473 | 1DB1 | M015349 | -13.5 |
| P12271 | 3HX3 | M004705 | -13.5 |
| P19099 | 4ZGX | M017584 | -13.5 |
| P12104 | 3AKM | M018528 | -13.49 |
| Q07343 | 1XLX | M019201 | -13.49 |
| P03956 | 966C | M006421 | -13.48 |
| P04798 | 4I8V | M029426 | -13.48 |
| P19099 | 4ZGX | M027649 | -13.48 |
| P39900 | 3EHX | M002414 | -13.48 |
| P03956 | 966C | M017716 | -13.47 |
| P39900 | 3EHX | M017716 | -13.47 |
| Q9UNQ0 | 6ETI | M001194 | -13.47 |
| P03956 | 966C | M014384 | -13.46 |
| P12271 | 3HX3 | M001790 | -13.46 |
| P15121 | 2PD5 | M031368 | -13.46 |
| P19099 | 4ZGX | M017133 | -13.46 |
| P28845 | 3EY4 | M025864 | -13.46 |
| P84077 | 1RE0 | M002163 | -13.46 |
| P28845 | 3EY4 | M007619 | -13.45 |
| Q9UNQ0 | 6ETI | M004519 | -13.45 |
| P19099 | 4ZGX | M019534 | -13.44 |
| P28845 | 3EY4 | M001215 | -13.44 |
| O67135 | 1C3R | M020429 | -13.43 |
| P19099 | 4ZGX | M023117 | -13.43 |
| P27707 | 2ZI5 | M016360 | -13.43 |
| P28845 | 3EY4 | M006052 | -13.43 |
| P56524 | 2VQV | M025643 | -13.43 |
| P28845 | 3EY4 | M019534 | -13.42 |
| P35968 | 2QU5 | M022421 | -13.42 |
| P05093 | 3RUK | M016215 | -13.41 |
| P56524 | 2VQV | M030533 | -13.41 |
| O14727 | 1Z6T | M018967 | -13.4 |
| O75469 | 1M13 | M006830 | -13.4 |
| P05106 | 2VDM | M019624 | -13.4 |
| P10109 | 3N9Y | M013211 | -13.4 |
| P03956 | 966C | M029882 | -13.39 |
| P11388 | 1ZXM | M018092 | -13.39 |
| P15121 | 2PD5 | M025532 | -13.39 |
| P19099 | 4ZGX | M014910 | -13.39 |
| P10109 | 3N9Y | M016908 | -13.38 |
| P19099 | 4ZGX | M023307 | -13.38 |
| P19099 | 4ZGX | M023861 | -13.38 |
| P19099 | 4ZGX | M024671 | -13.38 |
| P19099 | 4ZGX | M030304 | -13.38 |
| P27815 | 3I8V | M019201 | -13.38 |
| P84077 | 1RE0 | M001790 | -13.38 |
| P03956 | 966C | M025532 | -13.37 |
| P08253 | 1HOV | M024435 | -13.37 |
| P10109 | 3N9Y | M016866 | -13.37 |
| P11387 | 1T8I | M018782 | -13.37 |
| P03956 | 966C | M019628 | -13.36 |
| P12271 | 3HX3 | M025532 | -13.36 |
| P14555 | 1J1A | M003668 | -13.36 |
| P15121 | 2PD5 | M034198 | -13.36 |
| P03956 | 966C | M030533 | -13.35 |
| P04049 | 1GUA | M002414 | -13.35 |
| P11473 | 1DB1 | M012230 | -13.35 |
| P19099 | 4ZGX | M008598 | -13.35 |
| P51580 | 2H11 | M002414 | -13.35 |
| O75469 | 1M13 | M016215 | -13.34 |
| P03956 | 966C | M013272 | -13.34 |
| P05108 | 3NA0 | M012713 | -13.34 |
| P05108 | 3NA0 | M013452 | -13.34 |
| P28845 | 3EY4 | M033654 | -13.34 |
| P84077 | 1RE0 | M012665 | -13.34 |
| P28845 | 3EY4 | M011077 | -13.33 |
| P05108 | 3NA0 | M030384 | -13.32 |
| P14555 | 1J1A | M012419 | -13.32 |
| P27815 | 3I8V | M015955 | -13.32 |
| P39900 | 3EHX | M023398 | -13.32 |
| P05108 | 3NA0 | M010231 | -13.31 |
| P05108 | 3NA0 | M023823 | -13.31 |
| P15328 | 5IZQ | M020429 | -13.31 |
| P19099 | 4ZGX | M007663 | -13.31 |
| Q9BTZ2 | 3O4R | M020716 | -13.31 |
| P04150 | 3H52 | M028158 | -13.3 |
| P10109 | 3N9Y | M027246 | -13.3 |
| P12271 | 3HX3 | M015349 | -13.3 |
| P12271 | 3HX3 | M024666 | -13.3 |
| P51580 | 2H11 | M010376 | -13.3 |
| P51580 | 2H11 | M012713 | -13.3 |
| Q16647 | 3B6H | M002163 | -13.3 |
| P07550 | 3NY8 | M021203 | -13.29 |
| P10109 | 3N9Y | M004987 | -13.29 |
| P12271 | 3HX3 | M024435 | -13.29 |
| P19099 | 4ZGX | M028158 | -13.29 |
| P19099 | 4ZGX | M029426 | -13.29 |
| Q13946 | 1ZKL | M018782 | -13.29 |
| Q8N8N7 | 2W4Q | M013387 | -13.29 |
| P10826 | 1XAP | M002205 | -13.28 |
| P12104 | 3AKM | M027649 | -13.28 |
| O75469 | 1M13 | M016866 | -13.27 |
| P06401 | 2W8Y | M031236 | -13.27 |
| O67135 | 1C3R | M021732 | -13.26 |
| P19099 | 4ZGX | M024758 | -13.26 |
| P28845 | 3EY4 | M001790 | -13.26 |
| P39900 | 3EHX | M030099 | -13.26 |
| P84077 | 1RE0 | M004977 | -13.26 |
| O67135 | 1C3R | M015955 | -13.25 |
| P04798 | 4I8V | M004319 | -13.25 |
| P11387 | 1T8I | M004402 | -13.25 |
| P11473 | 1DB1 | M034198 | -13.25 |
| P12271 | 3HX3 | M026782 | -13.25 |
| P28845 | 3EY4 | M010294 | -13.25 |
| P29474 | 1M9J | M004519 | -13.25 |
| P37231 | 3H0A | M014384 | -13.25 |
| P05108 | 3NA0 | M030600 | -13.24 |
| P15121 | 2PD5 | M023398 | -13.24 |
| P28845 | 3EY4 | M031770 | -13.24 |
| P56524 | 2VQV | M002031 | -13.24 |
| P84077 | 1RE0 | M018092 | -13.24 |
| P03956 | 966C | M004319 | -13.23 |
| P10826 | 1XAP | M026864 | -13.23 |
| P28845 | 3EY4 | M002939 | -13.23 |
| P08253 | 1HOV | M020429 | -13.22 |
| P13631 | 3LBD | M004402 | -13.22 |
| P14555 | 1J1A | M001533 | -13.22 |
| P20815 | 5VEU | M020032 | -13.22 |
| P28845 | 3EY4 | M001968 | -13.22 |
| P51580 | 2H11 | M028060 | -13.22 |
| P84077 | 1RE0 | M026864 | -13.22 |
| Q8N8N7 | 2W4Q | M001605 | -13.22 |
| Q8N8N7 | 2W4Q | M018782 | -13.22 |
| P03956 | 966C | M004705 | -13.21 |
| P05108 | 3NA0 | M016908 | -13.21 |
| P10826 | 1XAP | M028420 | -13.21 |
| P11473 | 1DB1 | M014132 | -13.21 |
| P14555 | 1J1A | M026002 | -13.21 |
| P28845 | 3EY4 | M012665 | -13.21 |
| P39900 | 3EHX | M000744 | -13.21 |
| Q8N8N7 | 2W4Q | M033636 | -13.21 |
| O14727 | 1Z6T | M003198 | -13.2 |
| P03956 | 966C | M024929 | -13.2 |
| P04798 | 4I8V | M004705 | -13.2 |
| P06401 | 2W8Y | M002163 | -13.2 |
| P12271 | 3HX3 | M002205 | -13.2 |
| P19099 | 4ZGX | M025864 | -13.2 |
| O14727 | 1Z6T | M028991 | -13.19 |
| P03956 | 966C | M004402 | -13.19 |
| P06126 | 1XZ0 | M018092 | -13.19 |
| P06126 | 1XZ0 | M029693 | -13.19 |
| P10109 | 3N9Y | M013325 | -13.19 |
| P14555 | 1J1A | M031458 | -13.19 |
| P28845 | 3EY4 | M006317 | -13.19 |
| P51580 | 2H11 | M002163 | -13.19 |
| P07550 | 3NY8 | M019201 | -13.18 |
| P19099 | 4ZGX | M031808 | -13.18 |
| P26358 | 3SWR | M018092 | -13.18 |
| Q08499 | 3G4K | M019201 | -13.18 |
| O67135 | 1C3R | M004402 | -13.17 |
| P13631 | 3LBD | M026864 | -13.17 |
| P28845 | 3EY4 | M007599 | -13.17 |
| Q8N8N7 | 2W4Q | M034243 | -13.17 |
| O14727 | 1Z6T | M019628 | -13.16 |
| O67135 | 1C3R | M030533 | -13.16 |
| P08183 | 6FN1 | M003682 | -13.16 |
| P08253 | 1HOV | M015955 | -13.16 |
| P28845 | 3EY4 | M003009 | -13.16 |
| P37231 | 3H0A | M011284 | -13.16 |
| P39900 | 3EHX | M018782 | -13.16 |
| P84077 | 1RE0 | M013715 | -13.16 |
| Q8N8N7 | 2W4Q | M012298 | -13.16 |
| O14727 | 1Z6T | M032128 | -13.15 |
| O67135 | 1C3R | M005940 | -13.15 |
| P04798 | 4I8V | M002520 | -13.15 |
| P13631 | 3LBD | M026782 | -13.15 |
| P14555 | 1J1A | M027875 | -13.15 |
| P23458 | 3EYG | M019201 | -13.15 |
| P28845 | 3EY4 | M031808 | -13.15 |
| P37231 | 3H0A | M014132 | -13.15 |
| P37231 | 3H0A | M031368 | -13.15 |
| P05093 | 3RUK | M018773 | -13.14 |
| P11388 | 1ZXM | M024092 | -13.14 |
| P12271 | 3HX3 | M003214 | -13.14 |
| P28845 | 3EY4 | M018528 | -13.14 |
| P37231 | 3H0A | M004287 | -13.14 |
| P84077 | 1RE0 | M031368 | -13.14 |
| P08235 | 2OAX | M021791 | -13.13 |
| P10109 | 3N9Y | M008302 | -13.13 |
| P11473 | 1DB1 | M001215 | -13.13 |
| P12271 | 3HX3 | M004842 | -13.13 |
| P29474 | 1M9J | M018782 | -13.13 |
| P56524 | 2VQV | M024929 | -13.13 |
| P05108 | 3NA0 | M007948 | -13.12 |
| P05108 | 3NA0 | M016910 | -13.12 |
| P10826 | 1XAP | M001683 | -13.12 |
| P10826 | 1XAP | M007243 | -13.12 |
| P11387 | 1T8I | M030533 | -13.12 |
| P12271 | 3HX3 | M007243 | -13.12 |
| P17948 | 3HNG | M024929 | -13.12 |
| P19099 | 4ZGX | M025306 | -13.12 |
| P33261 | 4GQS | M014384 | -13.12 |
| P84077 | 1RE0 | M025532 | -13.12 |
| P03956 | 966C | M020032 | -13.11 |
| P11473 | 1DB1 | M031368 | -13.11 |
| P12271 | 3HX3 | M001215 | -13.11 |
| P13631 | 3LBD | M030533 | -13.11 |
| P39900 | 3EHX | M024965 | -13.11 |
| P10109 | 3N9Y | M007948 | -13.1 |
| P10109 | 3N9Y | M016910 | -13.1 |
| P15309 | 1ND5 | M026099 | -13.1 |
| P17948 | 3HNG | M012713 | -13.1 |
| P19099 | 4ZGX | M013272 | -13.1 |
| P19099 | 4ZGX | M017130 | -13.1 |
| P37231 | 3H0A | M017663 | -13.1 |
| P04150 | 3H52 | M008930 | -13.09 |
| P39900 | 3EHX | M006924 | -13.09 |
| P56524 | 2VQV | M003198 | -13.09 |
| Q13946 | 1ZKL | M024765 | -13.09 |
| P08631 | 2C0I | M010231 | -13.08 |
| P10109 | 3N9Y | M020716 | -13.08 |
| P11387 | 1T8I | M017130 | -13.08 |
| P14555 | 1J1A | M017333 | -13.08 |
| P19099 | 4ZGX | M002205 | -13.08 |
| P19099 | 4ZGX | M014063 | -13.08 |
| P19793 | 3KWY | M019628 | -13.08 |
| P28845 | 3EY4 | M005019 | -13.08 |
| P28845 | 3EY4 | M010232 | -13.08 |
| P28845 | 3EY4 | M011032 | -13.08 |
| Q8N8N7 | 2W4Q | M020429 | -13.08 |
| P10109 | 3N9Y | M012713 | -13.07 |
| P19099 | 4ZGX | M025711 | -13.07 |
| P20701 | 3BQM | M020429 | -13.07 |
| P56524 | 2VQV | M004519 | -13.07 |
| P00439 | 1TG2 | M018782 | -13.06 |
| P04798 | 4I8V | M006924 | -13.06 |
| P10826 | 1XAP | M004287 | -13.06 |
| P14555 | 1J1A | M033636 | -13.06 |
| P19099 | 4ZGX | M004977 | -13.06 |
| P28845 | 3EY4 | M000881 | -13.06 |
| P39900 | 3EHX | M010231 | -13.06 |
| P39900 | 3EHX | M024929 | -13.06 |
| P51580 | 2H11 | M030099 | -13.06 |
| O75469 | 1M13 | M031516 | -13.05 |
| P03956 | 966C | M023398 | -13.05 |
| P10826 | 1XAP | M026782 | -13.05 |
| P12271 | 3HX3 | M030014 | -13.05 |
| P42345 | 1FAP | M014384 | -13.05 |
| P84077 | 1RE0 | M034198 | -13.05 |
| P00519 | 2HYY | M030533 | -13.04 |
| P04798 | 4I8V | M030014 | -13.04 |
| P10109 | 3N9Y | M019381 | -13.04 |
| P10826 | 1XAP | M025726 | -13.04 |
| P14555 | 1J1A | M002520 | -13.04 |
| P28845 | 3EY4 | M002702 | -13.04 |
| P28845 | 3EY4 | M009135 | -13.04 |
| P08235 | 2OAX | M033654 | -13.03 |
| P04798 | 4I8V | M000700 | -13.02 |
| P10109 | 3N9Y | M003237 | -13.02 |
| P13631 | 3LBD | M028420 | -13.02 |
| P14555 | 1J1A | M020429 | -13.02 |
| P28845 | 3EY4 | M009523 | -13.02 |
| P28845 | 3EY4 | M012230 | -13.02 |
| P28845 | 3EY4 | M014582 | -13.02 |
| P28845 | 3EY4 | M027875 | -13.02 |
| P52333 | 3PJC | M013353 | -13.02 |
| O75469 | 1M13 | M013325 | -13.01 |
| P00519 | 2HYY | M024929 | -13.01 |
| P08631 | 2C0I | M012713 | -13.01 |
| P10826 | 1XAP | M013715 | -13.01 |
| P28845 | 3EY4 | M028517 | -13.01 |
| Q13946 | 1ZKL | M022800 | -13.01 |
| Q9UGN5 | 3KJD | M018092 | -13.01 |
| P07550 | 3NY8 | M015955 | -13 |
| P19099 | 4ZGX | M000208 | -13 |
| P19099 | 4ZGX | M014228 | -13 |
| P28845 | 3EY4 | M010937 | -13 |
| P51580 | 2H11 | M029426 | -13 |
| P07550 | 3NY8 | M002206 | -12.99 |
| P13631 | 3LBD | M019114 | -12.99 |
| P19099 | 4ZGX | M011649 | -12.99 |
| P19099 | 4ZGX | M017134 | -12.99 |
| P20815 | 5VEU | M019628 | -12.99 |
| P28845 | 3EY4 | M008468 | -12.99 |
| P39900 | 3EHX | M012713 | -12.99 |
| O14727 | 1Z6T | M017132 | -12.98 |
| O14757 | 3U9N | M019628 | -12.98 |
| P04150 | 3H52 | M025726 | -12.98 |
| P10109 | 3N9Y | M026002 | -12.98 |
| P12271 | 3HX3 | M006421 | -12.98 |
| P15121 | 2PD5 | M008651 | -12.98 |
| P84077 | 1RE0 | M029426 | -12.98 |
| P04049 | 1GUA | M031368 | -12.97 |
| P06126 | 1XZ0 | M007619 | -12.97 |
| P08235 | 2OAX | M004842 | -12.97 |
| P10826 | 1XAP | M024435 | -12.97 |
| P12271 | 3HX3 | M032923 | -12.97 |
| P14555 | 1J1A | M008930 | -12.97 |
| P14780 | 1GKC | M024929 | -12.97 |
| Q03181 | 3GZ9 | M008651 | -12.97 |
| Q9BTZ2 | 3O4R | M021322 | -12.97 |
| P04150 | 3H52 | M027875 | -12.96 |
| P09917 | 3V99 | M019628 | -12.96 |
| P10109 | 3N9Y | M016909 | -12.96 |
| P15121 | 2PD5 | M018782 | -12.96 |
| P28845 | 3EY4 | M031516 | -12.96 |
| P37231 | 3H0A | M019381 | -12.96 |
| P56524 | 2VQV | M027649 | -12.96 |
| P84077 | 1RE0 | M026782 | -12.96 |
| Q02127 | 3F1Q | M026833 | -12.96 |
| Q8N8N7 | 2W4Q | M004490 | -12.96 |
| Q9UGN5 | 3KJD | M019628 | -12.96 |
| O67135 | 1C3R | M014384 | -12.95 |
| P04150 | 3H52 | M029426 | -12.95 |
| P06401 | 2W8Y | M029426 | -12.95 |
| P12271 | 3HX3 | M019114 | -12.95 |
| P13631 | 3LBD | M025419 | -12.95 |
| P14780 | 1GKC | M002414 | -12.95 |
| P19099 | 4ZGX | M030014 | -12.95 |
| P26358 | 3SWR | M030533 | -12.95 |
| P27707 | 2ZI5 | M011639 | -12.95 |
| P39900 | 3EHX | M021319 | -12.95 |
| Q07869 | 1I7G | M019628 | -12.95 |
| O75469 | 1M13 | M014384 | -12.94 |
| P03956 | 966C | M008651 | -12.94 |
| P03956 | 966C | M010231 | -12.94 |
| P04049 | 1GUA | M011375 | -12.94 |
| P04150 | 3H52 | M024014 | -12.94 |
| P10109 | 3N9Y | M034198 | -12.94 |
| P13631 | 3LBD | M000604 | -12.94 |
| P19099 | 4ZGX | M011284 | -12.94 |
| P19099 | 4ZGX | M017131 | -12.94 |
| P19099 | 4ZGX | M025991 | -12.94 |
| P28845 | 3EY4 | M013708 | -12.94 |
| P37231 | 3H0A | M027649 | -12.94 |
| P39900 | 3EHX | M000604 | -12.94 |
| Q02127 | 3F1Q | M014384 | -12.94 |
| Q8N8N7 | 2W4Q | M014384 | -12.94 |
| O14727 | 1Z6T | M020032 | -12.93 |
| O67135 | 1C3R | M014910 | -12.93 |
| P04271 | 3HCM | M018782 | -12.93 |
| P05108 | 3NA0 | M026002 | -12.93 |
| P06401 | 2W8Y | M028420 | -12.93 |
| P10826 | 1XAP | M011284 | -12.93 |
| P28845 | 3EY4 | M028158 | -12.93 |
| P39900 | 3EHX | M026099 | -12.93 |
| Q9UGN5 | 3KJD | M020032 | -12.93 |
| P00519 | 2HYY | M012713 | -12.92 |
| P04798 | 4I8V | M011244 | -12.92 |
| P09210 | 2WJU | M020429 | -12.92 |
| P10109 | 3N9Y | M012298 | -12.92 |
| P10826 | 1XAP | M030014 | -12.92 |
| P14555 | 1J1A | M016215 | -12.92 |
| P14555 | 1J1A | M016908 | -12.92 |
| P15309 | 1ND5 | M010231 | -12.92 |
| P19099 | 4ZGX | M019340 | -12.92 |
| P19099 | 4ZGX | M034322 | -12.92 |
| P28845 | 3EY4 | M025381 | -12.92 |
| P28845 | 3EY4 | M032856 | -12.92 |
| P04049 | 1GUA | M011284 | -12.91 |
| P07550 | 3NY8 | M023823 | -12.91 |
| P13631 | 3LBD | M001683 | -12.91 |
| P14555 | 1J1A | M029090 | -12.91 |
| P28845 | 3EY4 | M007187 | -12.91 |
| P52333 | 3PJC | M014455 | -12.91 |
| P52333 | 3PJC | M025414 | -12.91 |
| P84077 | 1RE0 | M021181 | -12.91 |
| O15379 | 4A69 | M004467 | -12.9 |
| P06401 | 2W8Y | M002520 | -12.9 |
| P06401 | 2W8Y | M026864 | -12.9 |
| P11473 | 1DB1 | M001790 | -12.9 |
| P14780 | 1GKC | M023863 | -12.9 |
| P19099 | 4ZGX | M003198 | -12.9 |
| P05108 | 3NA0 | M011886 | -12.89 |
| P06401 | 2W8Y | M013272 | -12.89 |
| P19099 | 4ZGX | M023823 | -12.89 |
| P47712 | 1CJY | M019624 | -12.89 |
| P50613 | 1UA2 | M007037 | -12.89 |
| P52333 | 3PJC | M033636 | -12.89 |
| P56524 | 2VQV | M010231 | -12.89 |
| Q16647 | 3B6H | M029426 | -12.89 |
| Q8N8N7 | 2W4Q | M020498 | -12.89 |
| P04150 | 3H52 | M018092 | -12.88 |
| P05108 | 3NA0 | M027246 | -12.88 |
| P08253 | 1HOV | M012713 | -12.88 |
| P09210 | 2WJU | M030533 | -12.88 |
| P17948 | 3HNG | M030533 | -12.88 |
| P00519 | 2HYY | M003669 | -12.87 |
| P04049 | 1GUA | M014132 | -12.87 |
| P04150 | 3H52 | M010283 | -12.87 |
| P05093 | 3RUK | M007237 | -12.87 |
| P06401 | 2W8Y | M029686 | -12.87 |
| P10826 | 1XAP | M025532 | -12.87 |
| P12271 | 3HX3 | M026864 | -12.87 |
| P12271 | 3HX3 | M031236 | -12.87 |
| P14555 | 1J1A | M007948 | -12.87 |
| P28845 | 3EY4 | M012261 | -12.87 |
| P60763 | 2QME | M005571 | -12.87 |
| Q02763 | 3L8P | M019628 | -12.87 |
| P05108 | 3NA0 | M012298 | -12.86 |
| P08253 | 1HOV | M019628 | -12.86 |
| P10826 | 1XAP | M008930 | -12.86 |
| P28845 | 3EY4 | M011995 | -12.86 |
| P84077 | 1RE0 | M002205 | -12.86 |
| Q08499 | 3G4K | M019624 | -12.86 |
| O43741 | 2V8Q | M019381 | -12.85 |
| O75469 | 1M13 | M003914 | -12.85 |
| P11473 | 1DB1 | M028141 | -12.85 |
| P14780 | 1GKC | M018782 | -12.85 |
| P28845 | 3EY4 | M025414 | -12.85 |
| Q07869 | 1I7G | M020429 | -12.85 |
| O14727 | 1Z6T | M030466 | -12.84 |
| O15379 | 4A69 | M013452 | -12.84 |
| P03956 | 966C | M011375 | -12.84 |
| P04798 | 4I8V | M022163 | -12.84 |
| P11473 | 1DB1 | M031516 | -12.84 |
| P14555 | 1J1A | M019628 | -12.84 |
| P17948 | 3HNG | M004402 | -12.84 |
| P33261 | 4GQS | M031332 | -12.84 |
| Q13946 | 1ZKL | M020429 | -12.84 |
| P08253 | 1HOV | M010231 | -12.83 |
| P11387 | 1T8I | M018092 | -12.83 |
| P11388 | 1ZXM | M017133 | -12.83 |
| P14555 | 1J1A | M005398 | -12.83 |
| P17948 | 3HNG | M023398 | -12.83 |
| P56524 | 2VQV | M018528 | -12.83 |
| P84077 | 1RE0 | M001683 | -12.83 |
| Q16647 | 3B6H | M010568 | -12.83 |
| Q9UNQ0 | 6ETI | M019201 | -12.83 |
| O67135 | 1C3R | M030099 | -12.82 |
| P03956 | 966C | M002163 | -12.82 |
| P04798 | 4I8V | M008409 | -12.82 |
| P04798 | 4I8V | M023823 | -12.82 |
| P06401 | 2W8Y | M024164 | -12.82 |
| P08253 | 1HOV | M003669 | -12.82 |
| P10109 | 3N9Y | M024666 | -12.82 |
| P28845 | 3EY4 | M022840 | -12.82 |
| P37231 | 3H0A | M021322 | -12.82 |
| P84077 | 1RE0 | M014132 | -12.82 |
| Q02127 | 3F1Q | M004287 | -12.82 |
| Q02127 | 3F1Q | M021319 | -12.82 |
| P04150 | 3H52 | M004705 | -12.81 |
| P14555 | 1J1A | M029426 | -12.81 |
| P19099 | 4ZGX | M030971 | -12.81 |
| P19099 | 4ZGX | M034198 | -12.81 |
| P19652 | 3APW | M019628 | -12.81 |
| P39900 | 3EHX | M004329 | -12.81 |
| P05108 | 3NA0 | M020498 | -12.8 |
| P11473 | 1DB1 | M030703 | -12.8 |
| P12104 | 3AKM | M015349 | -12.8 |
| P14555 | 1J1A | M033654 | -12.8 |
| P19099 | 4ZGX | M008658 | -12.8 |
| P28845 | 3EY4 | M003914 | -12.8 |
| P28845 | 3EY4 | M027410 | -12.8 |
| P33261 | 4GQS | M004977 | -12.8 |
| P56524 | 2VQV | M022421 | -12.8 |
| P84077 | 1RE0 | M007243 | -12.8 |
| Q08345 | 3ZOS | M022421 | -12.8 |
| Q16647 | 3B6H | M004705 | -12.8 |
| Q9UNQ0 | 6ETI | M018528 | -12.8 |
| O75469 | 1M13 | M018782 | -12.79 |
| P08235 | 2OAX | M030585 | -12.79 |
| P13631 | 3LBD | M007243 | -12.79 |
| P14555 | 1J1A | M015955 | -12.79 |
| P14555 | 1J1A | M028941 | -12.79 |
| P19099 | 4ZGX | M032629 | -12.79 |
| P35367 | 3RZE | M000604 | -12.79 |
| P39900 | 3EHX | M004752 | -12.79 |
| Q16647 | 3B6H | M026782 | -12.79 |
| Q8N8N7 | 2W4Q | M024929 | -12.79 |
| O75469 | 1M13 | M012298 | -12.78 |
| P03956 | 966C | M026782 | -12.78 |
| P04150 | 3H52 | M031236 | -12.78 |
| P04798 | 4I8V | M025726 | -12.78 |
| P05093 | 3RUK | M004987 | -12.78 |
| P05108 | 3NA0 | M003399 | -12.78 |
| P08253 | 1HOV | M026833 | -12.78 |
| P10826 | 1XAP | M015561 | -12.78 |
| P28845 | 3EY4 | M015349 | -12.78 |
| P84077 | 1RE0 | M007663 | -12.78 |
| O67135 | 1C3R | M002205 | -12.77 |
| P04049 | 1GUA | M014384 | -12.77 |
| P05093 | 3RUK | M027425 | -12.77 |
| P05108 | 3NA0 | M033636 | -12.77 |
| P07550 | 3NY8 | M014384 | -12.77 |
| P08235 | 2OAX | M005571 | -12.77 |
| P08235 | 2OAX | M006317 | -12.77 |
| P10109 | 3N9Y | M001605 | -12.77 |
| P28845 | 3EY4 | M014132 | -12.77 |
| P29474 | 1M9J | M001194 | -12.77 |
| P39900 | 3EHX | M001762 | -12.77 |
| P84077 | 1RE0 | M021319 | -12.77 |
| Q8N8N7 | 2W4Q | M019628 | -12.77 |
| P05093 | 3RUK | M011284 | -12.76 |
| P06702 | 1IRJ | M018782 | -12.76 |
| P14555 | 1J1A | M030703 | -12.76 |
| P17252 | 3IW4 | M018782 | -12.76 |
| P19099 | 4ZGX | M021181 | -12.76 |
| P29474 | 1M9J | M018773 | -12.76 |
| P56524 | 2VQV | M031368 | -12.76 |
| Q02127 | 3F1Q | M004977 | -12.76 |
| Q08345 | 3ZOS | M011375 | -12.76 |
| P04150 | 3H52 | M014132 | -12.75 |
| P04150 | 3H52 | M030014 | -12.75 |
| P10109 | 3N9Y | M002939 | -12.75 |
| P10109 | 3N9Y | M022588 | -12.75 |
| P11388 | 1ZXM | M002163 | -12.75 |
| P12271 | 3HX3 | M018528 | -12.75 |
| P14555 | 1J1A | M033013 | -12.75 |
| P14780 | 1GKC | M004997 | -12.75 |
| P28845 | 3EY4 | M020716 | -12.75 |
| P29474 | 1M9J | M011831 | -12.75 |
| P39900 | 3EHX | M011375 | -12.75 |
| Q02127 | 3F1Q | M025645 | -12.75 |
| Q8N8N7 | 2W4Q | M020032 | -12.75 |
| O67135 | 1C3R | M016866 | -12.74 |
| P05108 | 3NA0 | M014517 | -12.74 |
| P08183 | 6FN1 | M024929 | -12.74 |
| P10826 | 1XAP | M029426 | -12.74 |
| P13631 | 3LBD | M023159 | -12.74 |
| P19099 | 4ZGX | M030466 | -12.74 |
| P28845 | 3EY4 | M006064 | -12.74 |
| P37231 | 3H0A | M028991 | -12.74 |
| P84077 | 1RE0 | M011284 | -12.74 |
| Q02750 | 3DY7 | M004977 | -12.74 |
| Q13490 | 3MUP | M019624 | -12.74 |
| P03956 | 966C | M029090 | -12.73 |
| P06126 | 1XZ0 | M019534 | -12.73 |
| P10109 | 3N9Y | M028941 | -12.73 |
| P14555 | 1J1A | M014384 | -12.73 |
| P19099 | 4ZGX | M019114 | -12.73 |
| P19099 | 4ZGX | M024164 | -12.73 |
| P20815 | 5VEU | M024765 | -12.73 |
| P20839 | 1JCN | M002414 | -12.73 |
| P39900 | 3EHX | M025419 | -12.73 |
| P52333 | 3PJC | M007619 | -12.73 |
| Q08345 | 3ZOS | M024929 | -12.73 |
| O75469 | 1M13 | M010232 | -12.72 |
| P04150 | 3H52 | M010568 | -12.72 |
| P04798 | 4I8V | M013006 | -12.72 |
| P08235 | 2OAX | M026864 | -12.72 |
| P09874 | 3L3L | M014001 | -12.72 |
| P12271 | 3HX3 | M023507 | -12.72 |
| P12271 | 3HX3 | M031808 | -12.72 |
| P14555 | 1J1A | M012261 | -12.72 |
| P15121 | 2PD5 | M024435 | -12.72 |
| P19099 | 4ZGX | M024435 | -12.72 |
| P19099 | 4ZGX | M026718 | -12.72 |
| P35228 | 4NOS | M010568 | -12.72 |
| P39900 | 3EHX | M023863 | -12.72 |
| P60763 | 2QME | M025532 | -12.72 |
| P84077 | 1RE0 | M023863 | -12.72 |
| Q9UGN5 | 3KJD | M023823 | -12.72 |
| O67135 | 1C3R | M011284 | -12.71 |
| P00439 | 1TG2 | M008651 | -12.71 |
| P04150 | 3H52 | M025624 | -12.71 |
| P04150 | 3H52 | M031368 | -12.71 |
| P04626 | 3RCD | M004287 | -12.71 |
| P04798 | 4I8V | M026250 | -12.71 |
| P05108 | 3NA0 | M020716 | -12.71 |
| P10109 | 3N9Y | M022800 | -12.71 |
| P10826 | 1XAP | M002280 | -12.71 |
| P10826 | 1XAP | M006421 | -12.71 |
| P12271 | 3HX3 | M034061 | -12.71 |
| P14555 | 1J1A | M004977 | -12.71 |
| P19099 | 4ZGX | M007243 | -12.71 |
| P20815 | 5VEU | M016866 | -12.71 |
| P28845 | 3EY4 | M026864 | -12.71 |
| P35367 | 3RZE | M023863 | -12.71 |
| Q8N8N7 | 2W4Q | M014063 | -12.71 |
| Q9UGN5 | 3KJD | M020429 | -12.71 |
| P00519 | 2HYY | M010231 | -12.7 |
| P04150 | 3H52 | M025532 | -12.7 |
| P10721 | 3G0E | M008651 | -12.7 |
| P11388 | 1ZXM | M012230 | -12.7 |
| P13631 | 3LBD | M023863 | -12.7 |
| P14555 | 1J1A | M015525 | -12.7 |
| P20815 | 5VEU | M018528 | -12.7 |
| P28845 | 3EY4 | M012765 | -12.7 |
| P28845 | 3EY4 | M029693 | -12.7 |
| P52333 | 3PJC | M025864 | -12.7 |
| Q8N8N7 | 2W4Q | M018773 | -12.7 |
| Q9UNQ0 | 6ETI | M020032 | -12.7 |
| P04798 | 4I8V | M020456 | -12.69 |
| P05106 | 2VDM | M018782 | -12.69 |
| P10109 | 3N9Y | M004329 | -12.69 |
| P11387 | 1T8I | M020032 | -12.69 |
| P13631 | 3LBD | M002205 | -12.69 |
| P19099 | 4ZGX | M010232 | -12.69 |
| P19099 | 4ZGX | M033351 | -12.69 |
| P28482 | 3I5Z | M019628 | -12.69 |
| P35367 | 3RZE | M027649 | -12.69 |
| O67135 | 1C3R | M025726 | -12.68 |
| O75469 | 1M13 | M003668 | -12.68 |
| P12271 | 3HX3 | M034198 | -12.68 |
| P19099 | 4ZGX | M021602 | -12.68 |
| P28845 | 3EY4 | M027170 | -12.68 |
| P28845 | 3EY4 | M034061 | -12.68 |
| P33261 | 4GQS | M009523 | -12.68 |
| P35367 | 3RZE | M011375 | -12.68 |
| P56524 | 2VQV | M012298 | -12.68 |
| P56524 | 2VQV | M014132 | -12.68 |
| P84077 | 1RE0 | M002520 | -12.68 |
| Q02763 | 3L8P | M020429 | -12.68 |
| Q07889 | 4URX | M018782 | -12.68 |
| O75469 | 1M13 | M012713 | -12.67 |
| P04150 | 3H52 | M028941 | -12.67 |
| P06401 | 2W8Y | M004705 | -12.67 |
| P10109 | 3N9Y | M019343 | -12.67 |
| P15328 | 5IZQ | M027649 | -12.67 |
| P29474 | 1M9J | M019201 | -12.67 |
| P35367 | 3RZE | M012532 | -12.67 |
| P42345 | 1FAP | M015349 | -12.67 |
| P56524 | 2VQV | M015955 | -12.67 |
| P60763 | 2QME | M025645 | -12.67 |
| Q16647 | 3B6H | M002520 | -12.67 |
| P04798 | 4I8V | M010890 | -12.66 |
| P05108 | 3NA0 | M014132 | -12.66 |
| P11473 | 1DB1 | M019381 | -12.66 |
| P12271 | 3HX3 | M025726 | -12.66 |
| P14555 | 1J1A | M031770 | -12.66 |
| P15121 | 2PD5 | M003009 | -12.66 |
| P15121 | 2PD5 | M010568 | -12.66 |
| P15121 | 2PD5 | M025726 | -12.66 |
| P19099 | 4ZGX | M031021 | -12.66 |
| P37231 | 3H0A | M020716 | -12.66 |
| P52333 | 3PJC | M022800 | -12.66 |
| P56524 | 2VQV | M028923 | -12.66 |
| Q08345 | 3ZOS | M010231 | -12.66 |
| P04150 | 3H52 | M001683 | -12.65 |
| P04150 | 3H52 | M033654 | -12.65 |
| P14780 | 1GKC | M000604 | -12.65 |
| P14780 | 1GKC | M011375 | -12.65 |
| P19099 | 4ZGX | M024014 | -12.65 |
| P19099 | 4ZGX | M026782 | -12.65 |
| P28845 | 3EY4 | M002163 | -12.65 |
| P28845 | 3EY4 | M021602 | -12.65 |
| P33261 | 4GQS | M024528 | -12.65 |
| P52333 | 3PJC | M011032 | -12.65 |
| P56524 | 2VQV | M008651 | -12.65 |
| P60763 | 2QME | M013272 | -12.65 |
| P84077 | 1RE0 | M013272 | -12.65 |
| P05108 | 3NA0 | M020032 | -12.64 |
| P10109 | 3N9Y | M011639 | -12.64 |
| P15121 | 2PD5 | M008930 | -12.64 |
| P15121 | 2PD5 | M032246 | -12.64 |
| P19099 | 4ZGX | M006421 | -12.64 |
| P28845 | 3EY4 | M014282 | -12.64 |
| P33261 | 4GQS | M020429 | -12.64 |
| P52333 | 3PJC | M007281 | -12.64 |
| P60763 | 2QME | M026833 | -12.64 |
| O75469 | 1M13 | M014578 | -12.63 |
| P00374 | 2W3A | M028141 | -12.63 |
| P10826 | 1XAP | M027875 | -12.63 |
| P19099 | 4ZGX | M013275 | -12.63 |
| P19099 | 4ZGX | M026864 | -12.63 |
| P28845 | 3EY4 | M033307 | -12.63 |
| P35354 | 3LN1 | M010231 | -12.63 |
| Q02763 | 3L8P | M018528 | -12.63 |
| Q07869 | 1I7G | M009523 | -12.63 |
| P03956 | 966C | M028991 | -12.62 |
| P05108 | 3NA0 | M008302 | -12.62 |
| P05108 | 3NA0 | M016215 | -12.62 |
| P07550 | 3NY8 | M008930 | -12.62 |
| P10109 | 3N9Y | M026605 | -12.62 |
| P15121 | 2PD5 | M024164 | -12.62 |
| P15309 | 1ND5 | M024929 | -12.62 |
| P19099 | 4ZGX | M005571 | -12.62 |
| P19099 | 4ZGX | M014422 | -12.62 |
| P35228 | 4NOS | M019534 | -12.62 |
| P84077 | 1RE0 | M024435 | -12.62 |
| P04150 | 3H52 | M001215 | -12.61 |
| P04150 | 3H52 | M002520 | -12.61 |
| P10109 | 3N9Y | M026487 | -12.61 |
| P10826 | 1XAP | M021322 | -12.61 |
| P11473 | 1DB1 | M018528 | -12.61 |
| P13631 | 3LBD | M030014 | -12.61 |
| P14780 | 1GKC | M030099 | -12.61 |
| P19099 | 4ZGX | M005695 | -12.61 |
| P20815 | 5VEU | M020429 | -12.61 |
| P28845 | 3EY4 | M016361 | -12.61 |
| P56524 | 2VQV | M020032 | -12.61 |
| P60763 | 2QME | M004705 | -12.61 |
| P84077 | 1RE0 | M005541 | -12.61 |
| Q16647 | 3B6H | M026833 | -12.61 |
| O14757 | 3U9N | M020032 | -12.6 |
| P04150 | 3H52 | M030703 | -12.6 |
| P04798 | 4I8V | M026782 | -12.6 |
| P05108 | 3NA0 | M010294 | -12.6 |
| P07550 | 3NY8 | M019534 | -12.6 |
| P08235 | 2OAX | M013708 | -12.6 |
| P09455 | 5HBS | M028060 | -12.6 |
| P10109 | 3N9Y | M001215 | -12.6 |
| P11388 | 1ZXM | M022588 | -12.6 |
| P11388 | 1ZXM | M031837 | -12.6 |
| P15121 | 2PD5 | M021791 | -12.6 |
| P28845 | 3EY4 | M027246 | -12.6 |
| P56524 | 2VQV | M026099 | -12.6 |
| P84077 | 1RE0 | M028141 | -12.6 |
| Q8N8N7 | 2W4Q | M008651 | -12.6 |
| Q9BTZ2 | 3O4R | M014455 | -12.6 |
| O75469 | 1M13 | M003342 | -12.59 |
| P05108 | 3NA0 | M022421 | -12.59 |
| P08235 | 2OAX | M014422 | -12.59 |
| P09455 | 5HBS | M014384 | -12.59 |
| P11473 | 1DB1 | M020716 | -12.59 |
| P12931 | 2H8H | M000604 | -12.59 |
| P13631 | 3LBD | M007189 | -12.59 |
| P14555 | 1J1A | M011886 | -12.59 |
| P17252 | 3IW4 | M008930 | -12.59 |
| P19099 | 4ZGX | M004705 | -12.59 |
| P19099 | 4ZGX | M020101 | -12.59 |
| P19099 | 4ZGX | M029090 | -12.59 |
| P20292 | 2Q7R | M011831 | -12.59 |
| P20815 | 5VEU | M013387 | -12.59 |
| P28845 | 3EY4 | M016362 | -12.59 |
| P35228 | 4NOS | M008651 | -12.59 |
| P84077 | 1RE0 | M020429 | -12.59 |
| Q9UGN5 | 3KJD | M005746 | -12.59 |
| Q9UNQ0 | 6ETI | M007237 | -12.59 |
| O14920 | 4KIK | M020429 | -12.58 |
| P04150 | 3H52 | M021181 | -12.58 |
| P05093 | 3RUK | M011330 | -12.58 |
| P05108 | 3NA0 | M013325 | -12.58 |
| P05108 | 3NA0 | M026487 | -12.58 |
| P08235 | 2OAX | M002520 | -12.58 |
| P08235 | 2OAX | M029426 | -12.58 |
| P10109 | 3N9Y | M012483 | -12.58 |
| P11473 | 1DB1 | M028941 | -12.58 |
| P11511 | 3EQM | M028060 | -12.58 |
| P20292 | 2Q7R | M007619 | -12.58 |
| P28845 | 3EY4 | M001859 | -12.58 |
| P35367 | 3RZE | M014063 | -12.58 |
| P84077 | 1RE0 | M028420 | -12.58 |
| Q16647 | 3B6H | M025532 | -12.58 |
| P05108 | 3NA0 | M011330 | -12.57 |
| P06126 | 1XZ0 | M030533 | -12.57 |
| P10109 | 3N9Y | M013715 | -12.57 |
| P10109 | 3N9Y | M028991 | -12.57 |
| P11388 | 1ZXM | M029426 | -12.57 |
| P13569 | 3GD7 | M001194 | -12.57 |
| P14555 | 1J1A | M025624 | -12.57 |
| P17948 | 3HNG | M010568 | -12.57 |
| P27707 | 2ZI5 | M020716 | -12.57 |
| P28845 | 3EY4 | M021791 | -12.57 |
| P29474 | 1M9J | M012298 | -12.57 |
| P35228 | 4NOS | M019628 | -12.57 |
| P39900 | 3EHX | M000849 | -12.57 |
| P42345 | 1FAP | M011032 | -12.57 |
| P84077 | 1RE0 | M025643 | -12.57 |
| Q16647 | 3B6H | M028420 | -12.57 |
| Q8N8N7 | 2W4Q | M012419 | -12.57 |
| Q8N8N7 | 2W4Q | M030533 | -12.57 |
| P08235 | 2OAX | M013272 | -12.56 |
| P08253 | 1HOV | M002163 | -12.56 |
| P08253 | 1HOV | M022421 | -12.56 |
| P10109 | 3N9Y | M003399 | -12.56 |
| P13631 | 3LBD | M024435 | -12.56 |
| P14555 | 1J1A | M009447 | -12.56 |
| P14555 | 1J1A | M012483 | -12.56 |
| P15328 | 5IZQ | M025711 | -12.56 |
| P19099 | 4ZGX | M017663 | -12.56 |
| P27707 | 2ZI5 | M009447 | -12.56 |
| P27707 | 2ZI5 | M021875 | -12.56 |
| P37231 | 3H0A | M018528 | -12.56 |
| P39900 | 3EHX | M000173 | -12.56 |
| P84077 | 1RE0 | M028033 | -12.56 |
| P84077 | 1RE0 | M030014 | -12.56 |
| Q9UNQ0 | 6ETI | M015349 | -12.56 |
| P03956 | 966C | M026099 | -12.55 |
| P04049 | 1GUA | M004287 | -12.55 |
| P04150 | 3H52 | M026864 | -12.55 |
| P06126 | 1XZ0 | M019628 | -12.55 |
| P10109 | 3N9Y | M010231 | -12.55 |
| P17948 | 3HNG | M008651 | -12.55 |
| P19099 | 4ZGX | M028420 | -12.55 |
| P20839 | 1JCN | M008651 | -12.55 |
| P35228 | 4NOS | M020429 | -12.55 |
| P37231 | 3H0A | M011744 | -12.55 |
| P51580 | 2H11 | M028992 | -12.55 |
| P52333 | 3PJC | M008930 | -12.55 |
| P56524 | 2VQV | M019201 | -12.55 |
| Q00535 | 3O0G | M020716 | -12.55 |
| Q02127 | 3F1Q | M020716 | -12.55 |
| O67135 | 1C3R | M020216 | -12.54 |
| P03956 | 966C | M000208 | -12.54 |
| P04818 | 3EJL | M020032 | -12.54 |
| P06126 | 1XZ0 | M004842 | -12.54 |
| P07550 | 3NY8 | M023863 | -12.54 |
| P09455 | 5HBS | M029426 | -12.54 |
| P10109 | 3N9Y | M011330 | -12.54 |
| P12271 | 3HX3 | M005571 | -12.54 |
| P14780 | 1GKC | M004329 | -12.54 |
| P14780 | 1GKC | M008651 | -12.54 |
| P15121 | 2PD5 | M000744 | -12.54 |
| P19099 | 4ZGX | M001683 | -12.54 |
| P19099 | 4ZGX | M018331 | -12.54 |
| P19099 | 4ZGX | M025532 | -12.54 |
| P19099 | 4ZGX | M030099 | -12.54 |
| P28845 | 3EY4 | M024523 | -12.54 |
| P39900 | 3EHX | M021391 | -12.54 |
| P42345 | 1FAP | M018528 | -12.54 |
| P84077 | 1RE0 | M000604 | -12.54 |
| P84077 | 1RE0 | M023398 | -12.54 |
| P84077 | 1RE0 | M029693 | -12.54 |
| O14727 | 1Z6T | M000486 | -12.53 |
| P04798 | 4I8V | M027807 | -12.53 |
| P04798 | 4I8V | M032462 | -12.53 |
| P08235 | 2OAX | M003914 | -12.53 |
| P08253 | 1HOV | M024929 | -12.53 |
| P10109 | 3N9Y | M018773 | -12.53 |
| P10109 | 3N9Y | M033013 | -12.53 |
| P15121 | 2PD5 | M008658 | -12.53 |
| P15328 | 5IZQ | M002206 | -12.53 |
| P19099 | 4ZGX | M003338 | -12.53 |
| P20839 | 1JCN | M030099 | -12.53 |
| P28845 | 3EY4 | M008874 | -12.53 |
| P28845 | 3EY4 | M018092 | -12.53 |
| P33261 | 4GQS | M011077 | -12.53 |
| P35222 | 3TX7 | M020032 | -12.53 |
| P51580 | 2H11 | M031236 | -12.53 |
| P56524 | 2VQV | M000604 | -12.53 |
| P60763 | 2QME | M002520 | -12.53 |
| P84077 | 1RE0 | M010212 | -12.53 |
| Q03181 | 3GZ9 | M029426 | -12.53 |
| Q08345 | 3ZOS | M003669 | -12.53 |
| O75469 | 1M13 | M019534 | -12.52 |
| P00374 | 2W3A | M033801 | -12.52 |
| P09455 | 5HBS | M002520 | -12.52 |
| P12104 | 3AKM | M028941 | -12.52 |
| P14555 | 1J1A | M020498 | -12.52 |
| P14780 | 1GKC | M023823 | -12.52 |
| P28845 | 3EY4 | M024014 | -12.52 |
| P39900 | 3EHX | M017235 | -12.52 |
| P42345 | 1FAP | M019628 | -12.52 |
| P84077 | 1RE0 | M014422 | -12.52 |
| P84077 | 1RE0 | M015599 | -12.52 |
| Q16647 | 3B6H | M007243 | -12.52 |
| Q8N8N7 | 2W4Q | M003668 | -12.52 |
| O14757 | 3U9N | M020429 | -12.51 |
| O67135 | 1C3R | M019201 | -12.51 |
| P00374 | 2W3A | M027905 | -12.51 |
| P04150 | 3H52 | M024435 | -12.51 |
| P04798 | 4I8V | M024245 | -12.51 |
| P05093 | 3RUK | M013353 | -12.51 |
| P05108 | 3NA0 | M003914 | -12.51 |
| P05108 | 3NA0 | M014384 | -12.51 |
| P10109 | 3N9Y | M020162 | -12.51 |
| P10109 | 3N9Y | M020662 | -12.51 |
| P11388 | 1ZXM | M011674 | -12.51 |
| P11511 | 3EQM | M012982 | -12.51 |
| P28845 | 3EY4 | M021322 | -12.51 |
| P51580 | 2H11 | M000700 | -12.51 |
| Q02750 | 3DY7 | M034198 | -12.51 |
| O14727 | 1Z6T | M027649 | -12.5 |
| P04049 | 1GUA | M018528 | -12.5 |
| P04150 | 3H52 | M028060 | -12.5 |
| P05093 | 3RUK | M030600 | -12.5 |
| P08253 | 1HOV | M000700 | -12.5 |
| P15328 | 5IZQ | M017584 | -12.5 |
| P17948 | 3HNG | M003669 | -12.5 |
| P20815 | 5VEU | M005940 | -12.5 |
| P28845 | 3EY4 | M005695 | -12.5 |
| P42684 | 3GVU | M003669 | -12.5 |
| P56524 | 2VQV | M003669 | -12.5 |
| P84077 | 1RE0 | M019534 | -12.5 |
| Q02750 | 3DY7 | M014384 | -12.5 |
| Q08345 | 3ZOS | M030099 | -12.5 |
| Q08499 | 3G4K | M014384 | -12.5 |
| O75469 | 1M13 | M012483 | -12.49 |
| O75469 | 1M13 | M012665 | -12.49 |
| O75469 | 1M13 | M025645 | -12.49 |
| P03956 | 966C | M018782 | -12.49 |
| P09210 | 2WJU | M020032 | -12.49 |
| P11473 | 1DB1 | M004977 | -12.49 |
| P11473 | 1DB1 | M008930 | -12.49 |
| P14555 | 1J1A | M005019 | -12.49 |
| P14555 | 1J1A | M024671 | -12.49 |
| P19099 | 4ZGX | M017716 | -12.49 |
| P19099 | 4ZGX | M021952 | -12.49 |
| P33261 | 4GQS | M006052 | -12.49 |
| P84077 | 1RE0 | M014517 | -12.49 |
| Q02750 | 3DY7 | M023823 | -12.49 |
| P04798 | 4I8V | M002205 | -12.48 |
| P04798 | 4I8V | M009679 | -12.48 |
| P06126 | 1XZ0 | M025864 | -12.48 |
| P10826 | 1XAP | M004842 | -12.48 |
| P12271 | 3HX3 | M008302 | -12.48 |
| P19099 | 4ZGX | M028602 | -12.48 |
| P20815 | 5VEU | M031368 | -12.48 |
| P33261 | 4GQS | M014578 | -12.48 |
| P42345 | 1FAP | M020429 | -12.48 |
| P52333 | 3PJC | M031808 | -12.48 |
| P56524 | 2VQV | M019628 | -12.48 |
| P84077 | 1RE0 | M001215 | -12.48 |
| P84077 | 1RE0 | M027410 | -12.48 |
| Q8N8N7 | 2W4Q | M019201 | -12.48 |
| P00374 | 2W3A | M020032 | -12.47 |
| P00439 | 1TG2 | M020032 | -12.47 |
| P03956 | 966C | M012713 | -12.47 |
| P04150 | 3H52 | M007243 | -12.47 |
| P04150 | 3H52 | M009135 | -12.47 |
| P09455 | 5HBS | M004977 | -12.47 |
| P10109 | 3N9Y | M023823 | -12.47 |
| P12104 | 3AKM | M001215 | -12.47 |
| P12931 | 2H8H | M031368 | -12.47 |
| P19099 | 4ZGX | M008764 | -12.47 |
| P19099 | 4ZGX | M020716 | -12.47 |
| P20815 | 5VEU | M014001 | -12.47 |
| P27707 | 2ZI5 | M004977 | -12.47 |
| P28845 | 3EY4 | M002504 | -12.47 |
| P29474 | 1M9J | M007619 | -12.47 |
| P51580 | 2H11 | M033670 | -12.47 |
| Q02763 | 3L8P | M002414 | -12.47 |
| P04150 | 3H52 | M026782 | -12.46 |
| P04798 | 4I8V | M022218 | -12.46 |
| P05093 | 3RUK | M004287 | -12.46 |
| P05108 | 3NA0 | M028941 | -12.46 |
| P06126 | 1XZ0 | M023823 | -12.46 |
| P08253 | 1HOV | M020032 | -12.46 |
| P10109 | 3N9Y | M031516 | -12.46 |
| P10826 | 1XAP | M008481 | -12.46 |
| P12104 | 3AKM | M029090 | -12.46 |
| P15121 | 2PD5 | M009523 | -12.46 |
| P20701 | 3BQM | M033013 | -12.46 |
| P27707 | 2ZI5 | M024528 | -12.46 |
| P56524 | 2VQV | M014063 | -12.46 |
| P56524 | 2VQV | M021319 | -12.46 |
| P03956 | 966C | M003669 | -12.45 |
| P04049 | 1GUA | M024095 | -12.45 |
| P04150 | 3H52 | M005571 | -12.45 |
| P05093 | 3RUK | M005398 | -12.45 |
| P08235 | 2OAX | M007599 | -12.45 |
| P10826 | 1XAP | M009135 | -12.45 |
| P14555 | 1J1A | M003198 | -12.45 |
| P14555 | 1J1A | M011284 | -12.45 |
| P15328 | 5IZQ | M018528 | -12.45 |
| P29474 | 1M9J | M008651 | -12.45 |
| P37231 | 3H0A | M004977 | -12.45 |
| P37231 | 3H0A | M015349 | -12.45 |
| P42345 | 1FAP | M020032 | -12.45 |
| P60763 | 2QME | M020429 | -12.45 |
| P84077 | 1RE0 | M005571 | -12.45 |
| Q16647 | 3B6H | M030014 | -12.45 |
| Q8N8N7 | 2W4Q | M004519 | -12.45 |
| O75469 | 1M13 | M021081 | -12.44 |
| P05108 | 3NA0 | M031485 | -12.44 |
| P08235 | 2OAX | M026275 | -12.44 |
| P08631 | 2C0I | M003669 | -12.44 |
| P10826 | 1XAP | M010568 | -12.44 |
| P11473 | 1DB1 | M025624 | -12.44 |
| P12104 | 3AKM | M034198 | -12.44 |
| P12271 | 3HX3 | M010294 | -12.44 |
| P14555 | 1J1A | M024523 | -12.44 |
| P15121 | 2PD5 | M014384 | -12.44 |
| P15328 | 5IZQ | M002520 | -12.44 |
| P15328 | 5IZQ | M024014 | -12.44 |
| P28845 | 3EY4 | M032923 | -12.44 |
| P39900 | 3EHX | M022262 | -12.44 |
| Q13946 | 1ZKL | M018528 | -12.44 |
| Q13946 | 1ZKL | M019201 | -12.44 |
| Q9Y6F1 | 3CE0 | M003198 | -12.44 |
| O14727 | 1Z6T | M011831 | -12.43 |
| O75469 | 1M13 | M011995 | -12.43 |
| P05108 | 3NA0 | M001605 | -12.43 |
| P05108 | 3NA0 | M026382 | -12.43 |
| P07550 | 3NY8 | M001790 | -12.43 |
| P11473 | 1DB1 | M033654 | -12.43 |
| P11511 | 3EQM | M017778 | -12.43 |
| P15121 | 2PD5 | M031236 | -12.43 |
| P15144 | 4FYR | M018782 | -12.43 |
| P19099 | 4ZGX | M028991 | -12.43 |
| P28845 | 3EY4 | M027461 | -12.43 |
| P37231 | 3H0A | M031332 | -12.43 |
| P39900 | 3EHX | M014085 | -12.43 |
| P39900 | 3EHX | M024621 | -12.43 |
| P42345 | 1FAP | M031368 | -12.43 |
| P84077 | 1RE0 | M028060 | -12.43 |
| Q16647 | 3B6H | M002205 | -12.43 |
| Q8N8N7 | 2W4Q | M018528 | -12.43 |
| Q9BTZ2 | 3O4R | M019628 | -12.43 |
| O14727 | 1Z6T | M021181 | -12.42 |
| P00374 | 2W3A | M018782 | -12.42 |
| P04150 | 3H52 | M013275 | -12.42 |
| P04798 | 4I8V | M001118 | -12.42 |
| P05093 | 3RUK | M001533 | -12.42 |
| P07550 | 3NY8 | M002414 | -12.42 |
| P09455 | 5HBS | M025726 | -12.42 |
| P10109 | 3N9Y | M014517 | -12.42 |
| P10826 | 1XAP | M003214 | -12.42 |
| P12271 | 3HX3 | M024164 | -12.42 |
| P15328 | 5IZQ | M008651 | -12.42 |
| P19099 | 4ZGX | M021266 | -12.42 |
| P19099 | 4ZGX | M030514 | -12.42 |
| P28845 | 3EY4 | M021081 | -12.42 |
| P42345 | 1FAP | M011639 | -12.42 |
| Q03181 | 3GZ9 | M027649 | -12.42 |
| P00492 | 1BZY | M019624 | -12.41 |
| P04150 | 3H52 | M007599 | -12.41 |
| P04150 | 3H52 | M011995 | -12.41 |
| P05108 | 3NA0 | M013211 | -12.41 |
| P05108 | 3NA0 | M019628 | -12.41 |
| P08235 | 2OAX | M002280 | -12.41 |
| P11387 | 1T8I | M019628 | -12.41 |
| P11387 | 1T8I | M024929 | -12.41 |
| P14555 | 1J1A | M004987 | -12.41 |
| P14555 | 1J1A | M006830 | -12.41 |
| P14555 | 1J1A | M013325 | -12.41 |
| P14555 | 1J1A | M014578 | -12.41 |
| P36507 | 1S9I | M025643 | -12.41 |
| P42345 | 1FAP | M019201 | -12.41 |
| P84077 | 1RE0 | M001099 | -12.41 |
| P84077 | 1RE0 | M009539 | -12.41 |
| Q13946 | 1ZKL | M020032 | -12.41 |
| O15379 | 4A69 | M000897 | -12.4 |
| P04150 | 3H52 | M004842 | -12.4 |
| P04150 | 3H52 | M027323 | -12.4 |
| P05108 | 3NA0 | M010872 | -12.4 |
| P08235 | 2OAX | M006421 | -12.4 |
| P09210 | 2WJU | M006830 | -12.4 |
| P10109 | 3N9Y | M011032 | -12.4 |
| P11388 | 1ZXM | M001239 | -12.4 |
| P11473 | 1DB1 | M018092 | -12.4 |
| P19099 | 4ZGX | M017688 | -12.4 |
| P20839 | 1JCN | M004287 | -12.4 |
| P37231 | 3H0A | M019628 | -12.4 |
| P42684 | 3GVU | M024929 | -12.4 |
| P56524 | 2VQV | M009135 | -12.4 |
| P84077 | 1RE0 | M014267 | -12.4 |
| P84077 | 1RE0 | M028329 | -12.4 |
| P84077 | 1RE0 | M030422 | -12.4 |
| Q02127 | 3F1Q | M008302 | -12.4 |
| Q8N8N7 | 2W4Q | M031458 | -12.4 |
| Q9UNQ0 | 6ETI | M014384 | -12.4 |
| P03956 | 966C | M012298 | -12.39 |
| P04818 | 3EJL | M019628 | -12.39 |
| P10109 | 3N9Y | M021602 | -12.39 |
| P10276 | 3KMR | M023863 | -12.39 |
| P10826 | 1XAP | M000604 | -12.39 |
| P10826 | 1XAP | M020716 | -12.39 |
| P11388 | 1ZXM | M023877 | -12.39 |
| P14555 | 1J1A | M018528 | -12.39 |
| P14555 | 1J1A | M021875 | -12.39 |
| P14780 | 1GKC | M010231 | -12.39 |
| P19099 | 4ZGX | M007281 | -12.39 |
| P19099 | 4ZGX | M023877 | -12.39 |
| P28845 | 3EY4 | M006672 | -12.39 |
| P29474 | 1M9J | M007237 | -12.39 |
| P29474 | 1M9J | M015955 | -12.39 |
| P37231 | 3H0A | M023863 | -12.39 |
| O75469 | 1M13 | M006317 | -12.38 |
| P04150 | 3H52 | M019114 | -12.38 |
| P05108 | 3NA0 | M026605 | -12.38 |
| P08235 | 2OAX | M033307 | -12.38 |
| P08253 | 1HOV | M004842 | -12.38 |
| P08253 | 1HOV | M029090 | -12.38 |
| P09455 | 5HBS | M014422 | -12.38 |
| P10109 | 3N9Y | M003914 | -12.38 |
| P11473 | 1DB1 | M031021 | -12.38 |
| P12271 | 3HX3 | M003914 | -12.38 |
| P14555 | 1J1A | M003237 | -12.38 |
| P19099 | 4ZGX | M002280 | -12.38 |
| P28845 | 3EY4 | M008463 | -12.38 |
| P28845 | 3EY4 | M031021 | -12.38 |
| P35222 | 3TX7 | M018782 | -12.38 |
| P39900 | 3EHX | M032462 | -12.38 |
| P60763 | 2QME | M028420 | -12.38 |
| P84077 | 1RE0 | M034061 | -12.38 |
| O14727 | 1Z6T | M014384 | -12.37 |
| O75469 | 1M13 | M001790 | -12.37 |
| O75469 | 1M13 | M007272 | -12.37 |
| O75469 | 1M13 | M024523 | -12.37 |
| O75469 | 1M13 | M027410 | -12.37 |
| P00519 | 2HYY | M008651 | -12.37 |
| P04150 | 3H52 | M003914 | -12.37 |
| P08631 | 2C0I | M024929 | -12.37 |
| P10109 | 3N9Y | M023877 | -12.37 |
| P10826 | 1XAP | M002520 | -12.37 |
| P11473 | 1DB1 | M005019 | -12.37 |
| P12271 | 3HX3 | M033909 | -12.37 |
| P17948 | 3HNG | M030099 | -12.37 |
| P19099 | 4ZGX | M020498 | -12.37 |
| P33261 | 4GQS | M014422 | -12.37 |
| P84077 | 1RE0 | M009135 | -12.37 |
| P84077 | 1RE0 | M018528 | -12.37 |
| Q02763 | 3L8P | M014384 | -12.37 |
| Q8N8N7 | 2W4Q | M034198 | -12.37 |
| P04150 | 3H52 | M003668 | -12.36 |
| P06401 | 2W8Y | M001683 | -12.36 |
| P11473 | 1DB1 | M026382 | -12.36 |
| P12104 | 3AKM | M008651 | -12.36 |
| P14555 | 1J1A | M007272 | -12.36 |
| P27815 | 3I8V | M018528 | -12.36 |
| P28845 | 3EY4 | M025624 | -12.36 |
| P33261 | 4GQS | M007663 | -12.36 |
| P39900 | 3EHX | M003669 | -12.36 |
| P39900 | 3EHX | M022421 | -12.36 |
| P39900 | 3EHX | M025298 | -12.36 |
| P84077 | 1RE0 | M025711 | -12.36 |
| P84077 | 1RE0 | M031236 | -12.36 |
| Q02763 | 3L8P | M030533 | -12.36 |
| Q16647 | 3B6H | M001683 | -12.36 |
| O14920 | 4KIK | M011831 | -12.35 |
| P00374 | 2W3A | M008365 | -12.35 |
| P00813 | 3IAR | M019201 | -12.35 |
| P04049 | 1GUA | M018967 | -12.35 |
| P05093 | 3RUK | M021203 | -12.35 |
| P05108 | 3NA0 | M010214 | -12.35 |
| P05771 | 2I0E | M020032 | -12.35 |
| P08235 | 2OAX | M010568 | -12.35 |
| P08235 | 2OAX | M014582 | -12.35 |
| P09455 | 5HBS | M013272 | -12.35 |
| P10109 | 3N9Y | M020556 | -12.35 |
| P14555 | 1J1A | M019514 | -12.35 |
| P15121 | 2PD5 | M003214 | -12.35 |
| P19099 | 4ZGX | M024634 | -12.35 |
| P28482 | 3I5Z | M020429 | -12.35 |
| P35228 | 4NOS | M020032 | -12.35 |
| P51580 | 2H11 | M008374 | -12.35 |
| P51580 | 2H11 | M013272 | -12.35 |
| Q00535 | 3O0G | M011831 | -12.35 |
| O67135 | 1C3R | M011375 | -12.34 |
| O75469 | 1M13 | M007619 | -12.34 |
| O75469 | 1M13 | M026605 | -12.34 |
| P03372 | 2QXS | M007663 | -12.34 |
| P04150 | 3H52 | M002146 | -12.34 |
| P07550 | 3NY8 | M033147 | -12.34 |
| P08631 | 2C0I | M027649 | -12.34 |
| P09917 | 3V99 | M012713 | -12.34 |
| P10109 | 3N9Y | M010294 | -12.34 |
| P11511 | 3EQM | M025438 | -12.34 |
| P14555 | 1J1A | M012625 | -12.34 |
| P17612 | 3POO | M004977 | -12.34 |
| P17948 | 3HNG | M012298 | -12.34 |
| P19099 | 4ZGX | M025726 | -12.34 |
| P27707 | 2ZI5 | M014281 | -12.34 |
| P41743 | 1ZRZ | M013211 | -12.34 |
| P60484 | 5BZZ | M012680 | -12.34 |
| P84077 | 1RE0 | M021322 | -12.34 |
| Q02750 | 3DY7 | M028141 | -12.34 |
| Q07869 | 1I7G | M020032 | -12.34 |
| Q8N8N7 | 2W4Q | M004402 | -12.34 |
| O15379 | 4A69 | M021771 | -12.33 |
| P04150 | 3H52 | M002205 | -12.33 |
| P08235 | 2OAX | M002163 | -12.33 |
| P11473 | 1DB1 | M007663 | -12.33 |
| P11473 | 1DB1 | M025645 | -12.33 |
| P11473 | 1DB1 | M027170 | -12.33 |
| P13569 | 3GD7 | M004287 | -12.33 |
| P14555 | 1J1A | M013452 | -12.33 |
| P26358 | 3SWR | M027246 | -12.33 |
| P29474 | 1M9J | M022800 | -12.33 |
| P51580 | 2H11 | M014132 | -12.33 |
| P56524 | 2VQV | M004752 | -12.33 |
| P56524 | 2VQV | M004977 | -12.33 |
| P84077 | 1RE0 | M012440 | -12.33 |
| P84077 | 1RE0 | M020920 | -12.33 |
| Q02127 | 3F1Q | M010568 | -12.33 |
| Q02763 | 3L8P | M004977 | -12.33 |
| Q07869 | 1I7G | M018092 | -12.33 |
| Q08499 | 3G4K | M018528 | -12.33 |
| Q16647 | 3B6H | M031236 | -12.33 |
| O75469 | 1M13 | M010283 | -12.32 |
| P02774 | 1J78 | M018782 | -12.32 |
| P04150 | 3H52 | M028141 | -12.32 |
| P04629 | 4AOJ | M027649 | -12.32 |
| P05093 | 3RUK | M012419 | -12.32 |
| P08235 | 2OAX | M005705 | -12.32 |
| P08235 | 2OAX | M028060 | -12.32 |
| P08253 | 1HOV | M023398 | -12.32 |
| P11473 | 1DB1 | M025643 | -12.32 |
| P12104 | 3AKM | M014384 | -12.32 |
| P12271 | 3HX3 | M031021 | -12.32 |
| P19099 | 4ZGX | M014132 | -12.32 |
| P19099 | 4ZGX | M032246 | -12.32 |
| P27707 | 2ZI5 | M021602 | -12.32 |
| P28845 | 3EY4 | M008527 | -12.32 |
| P29474 | 1M9J | M029426 | -12.32 |
| P42345 | 1FAP | M004977 | -12.32 |
| P84077 | 1RE0 | M004287 | -12.32 |
| P84077 | 1RE0 | M007599 | -12.32 |
| Q13946 | 1ZKL | M019628 | -12.32 |
| Q16647 | 3B6H | M024435 | -12.32 |
| O67135 | 1C3R | M002414 | -12.31 |
| P08235 | 2OAX | M001859 | -12.31 |
| P10109 | 3N9Y | M014384 | -12.31 |
| P13569 | 3GD7 | M018782 | -12.31 |
| P14555 | 1J1A | M000208 | -12.31 |
| P14555 | 1J1A | M013272 | -12.31 |
| P17948 | 3HNG | M023863 | -12.31 |
| P26358 | 3SWR | M031516 | -12.31 |
| P29474 | 1M9J | M027425 | -12.31 |
| P33261 | 4GQS | M027875 | -12.31 |
| P52333 | 3PJC | M016908 | -12.31 |
| P56524 | 2VQV | M018782 | -12.31 |
| P56524 | 2VQV | M024164 | -12.31 |
| P84077 | 1RE0 | M025438 | -12.31 |
| Q02750 | 3DY7 | M031368 | -12.31 |
| O67135 | 1C3R | M024929 | -12.3 |
| P04049 | 1GUA | M024092 | -12.3 |
| P04150 | 3H52 | M020920 | -12.3 |
| P05106 | 2VDM | M020429 | -12.3 |
| P06126 | 1XZ0 | M004402 | -12.3 |
| P07550 | 3NY8 | M004977 | -12.3 |
| P08235 | 2OAX | M030014 | -12.3 |
| P08631 | 2C0I | M029426 | -12.3 |
| P10109 | 3N9Y | M020032 | -12.3 |
| P10826 | 1XAP | M026992 | -12.3 |
| P11473 | 1DB1 | M027875 | -12.3 |
| P19652 | 3APW | M020032 | -12.3 |
| P28845 | 3EY4 | M026833 | -12.3 |
| P28845 | 3EY4 | M028141 | -12.3 |
| P33261 | 4GQS | M027461 | -12.3 |
| P37231 | 3H0A | M020032 | -12.3 |
| P51580 | 2H11 | M002520 | -12.3 |
| Q8N8N7 | 2W4Q | M031516 | -12.3 |
| O67135 | 1C3R | M000604 | -12.29 |
| P04150 | 3H52 | M013715 | -12.29 |
| P05093 | 3RUK | M013211 | -12.29 |
| P05106 | 2VDM | M002163 | -12.29 |
| P05108 | 3NA0 | M010816 | -12.29 |
| P06126 | 1XZ0 | M025711 | -12.29 |
| P08235 | 2OAX | M028420 | -12.29 |
| P09874 | 3L3L | M024765 | -12.29 |
| P10109 | 3N9Y | M021458 | -12.29 |
| P11387 | 1T8I | M011831 | -12.29 |
| P19099 | 4ZGX | M001968 | -12.29 |
| P19099 | 4ZGX | M012765 | -12.29 |
| P19099 | 4ZGX | M025438 | -12.29 |
| P20815 | 5VEU | M022800 | -12.29 |
| P20815 | 5VEU | M026487 | -12.29 |
| P26358 | 3SWR | M018967 | -12.29 |
| P28482 | 3I5Z | M020032 | -12.29 |
| P33261 | 4GQS | M010708 | -12.29 |
| P33261 | 4GQS | M018528 | -12.29 |
| P33261 | 4GQS | M028141 | -12.29 |
| P37231 | 3H0A | M028941 | -12.29 |
| P52333 | 3PJC | M003399 | -12.29 |
| P52333 | 3PJC | M013452 | -12.29 |
| Q9BTZ2 | 3O4R | M006830 | -12.29 |
| O14727 | 1Z6T | M030533 | -12.28 |
| O75469 | 1M13 | M001968 | -12.28 |
| P03956 | 966C | M004997 | -12.28 |
| P04049 | 1GUA | M030099 | -12.28 |
| P05093 | 3RUK | M013325 | -12.28 |
| P05108 | 3NA0 | M006052 | -12.28 |
| P05108 | 3NA0 | M015349 | -12.28 |
| P05108 | 3NA0 | M021458 | -12.28 |
| P05108 | 3NA0 | M024765 | -12.28 |
| P05164 | 3ZS0 | M014384 | -12.28 |
| P08235 | 2OAX | M001683 | -12.28 |
| P08235 | 2OAX | M024164 | -12.28 |
| P11473 | 1DB1 | M003914 | -12.28 |
| P11473 | 1DB1 | M014384 | -12.28 |
| P12104 | 3AKM | M018782 | -12.28 |
| P14555 | 1J1A | M031516 | -12.28 |
| P15328 | 5IZQ | M025062 | -12.28 |
| P15328 | 5IZQ | M033013 | -12.28 |
| P17948 | 3HNG | M004329 | -12.28 |
| P19099 | 4ZGX | M020621 | -12.28 |
| P19099 | 4ZGX | M022744 | -12.28 |
| P55212 | 4NBL | M030099 | -12.28 |
| Q08345 | 3ZOS | M004329 | -12.28 |
| Q9BTZ2 | 3O4R | M011284 | -12.28 |
