# Supplementary material for: Chinese Medicine for Psoriasis Vulgaris Based on Syndrome Pattern: A Network Pharmacological Study
Source: Evid Based Complement Alternat Med. 2020 Apr 28;2020:5239854. doi: 10.1155/2020/5239854 (PMC7204377; doi:10.1155/2020/5239854)
Supplement: Supplementary Materials — Figure S1: ADME/T properties of compounds of three TCM formulae. Table S1: herb composition and compounds of each formula retrieved from PDTCM. Table S2: psoriasis-related proteins. Table S3: compound-target binding energy lower than −12.28 kcal/mol. Table S4: GSEA results for three TCM formulae. [file 5239854.f1.zip › 5239854.f1/Table S4.docx]

**Table S4: GSEA results for three TCM formulae.**

| **Formula** | **Category** | **Term** | **Gene Count** | **P-Value** | **Fold Enrichment** |
| --- | --- | --- | --- | --- | --- |
| F1 | KEGG_PATHWAY | hsa05219:Bladder cancer | 7 | 1.18E-06 | 19.34 |
| F1 | KEGG_PATHWAY | hsa00140:Steroid hormone biosynthesis | 6 | 1.34E-04 | 11.72 |
| F1 | KEGG_PATHWAY | hsa05216:Thyroid cancer | 3 | 2.59E-02 | 11.72 |
| F1 | KEGG_PATHWAY | hsa05230:Central carbon metabolism in cancer | 6 | 2.15E-04 | 10.62 |
| F1 | KEGG_PATHWAY | hsa04913:Ovarian steroidogenesis | 4 | 8.64E-03 | 9.25 |
| F1 | KEGG_PATHWAY | hsa05213:Endometrial cancer | 4 | 1.02E-02 | 8.71 |
| F1 | KEGG_PATHWAY | hsa03320:PPAR signaling pathway | 5 | 2.62E-03 | 8.45 |
| F1 | KEGG_PATHWAY | hsa05221:Acute myeloid leukemia | 4 | 1.25E-02 | 8.09 |
| F1 | KEGG_PATHWAY | hsa05223:Non-small cell lung cancer | 4 | 1.25E-02 | 8.09 |
| F1 | KEGG_PATHWAY | hsa04370:VEGF signaling pathway | 4 | 1.57E-02 | 7.43 |
| F1 | KEGG_PATHWAY | hsa05204:Chemical carcinogenesis | 5 | 4.97E-03 | 7.08 |
| F1 | KEGG_PATHWAY | hsa05214:Glioma | 4 | 1.86E-02 | 6.97 |
| F1 | KEGG_PATHWAY | hsa05212:Pancreatic cancer | 4 | 1.86E-02 | 6.97 |
| F1 | KEGG_PATHWAY | hsa04919:Thyroid hormone signaling pathway | 7 | 4.28E-04 | 6.96 |
| F1 | KEGG_PATHWAY | hsa04066:HIF-1 signaling pathway | 6 | 1.53E-03 | 6.94 |
| F1 | KEGG_PATHWAY | hsa04915:Estrogen signaling pathway | 6 | 1.60E-03 | 6.87 |
| F1 | KEGG_PATHWAY | hsa04012:ErbB signaling pathway | 5 | 6.69E-03 | 6.51 |
| F1 | KEGG_PATHWAY | hsa05215:Prostate cancer | 5 | 6.96E-03 | 6.44 |
| F1 | KEGG_PATHWAY | hsa04917:Prolactin signaling pathway | 4 | 2.34E-02 | 6.38 |
| F1 | KEGG_PATHWAY | hsa04912:GnRH signaling pathway | 5 | 7.83E-03 | 6.22 |
| F1 | KEGG_PATHWAY | hsa00980:Metabolism of xenobiotics by cytochrome P450 | 4 | 2.61E-02 | 6.12 |
| F1 | KEGG_PATHWAY | hsa05222:Small cell lung cancer | 4 | 3.72E-02 | 5.33 |
| F1 | KEGG_PATHWAY | hsa04914:Progesterone-mediated oocyte maturation | 4 | 3.94E-02 | 5.21 |
| F1 | KEGG_PATHWAY | hsa04540:Gap junction | 4 | 4.06E-02 | 5.15 |
| F1 | KEGG_PATHWAY | hsa04024:cAMP signaling pathway | 9 | 2.81E-04 | 5.15 |
| F1 | KEGG_PATHWAY | hsa05205:Proteoglycans in cancer | 9 | 3.01E-04 | 5.10 |
| F1 | KEGG_PATHWAY | hsa05032:Morphine addiction | 4 | 4.41E-02 | 4.98 |
| F1 | KEGG_PATHWAY | hsa05161:Hepatitis B | 6 | 8.21E-03 | 4.69 |
| F1 | KEGG_PATHWAY | hsa04510:Focal adhesion | 8 | 1.91E-03 | 4.40 |
| F1 | KEGG_PATHWAY | hsa05200:Pathways in cancer | 15 | 4.60E-06 | 4.32 |
| F1 | KEGG_PATHWAY | hsa04910:Insulin signaling pathway | 5 | 3.14E-02 | 4.10 |
| F1 | KEGG_PATHWAY | hsa04015:Rap1 signaling pathway | 7 | 9.37E-03 | 3.78 |
| F1 | KEGG_PATHWAY | hsa04062:Chemokine signaling pathway | 6 | 2.21E-02 | 3.65 |
| F1 | KEGG_PATHWAY | hsa04921:Oxytocin signaling pathway | 5 | 4.78E-02 | 3.58 |
| F1 | KEGG_PATHWAY | hsa04014:Ras signaling pathway | 7 | 1.31E-02 | 3.51 |
| F1 | KEGG_PATHWAY | hsa05206:MicroRNAs in cancer | 8 | 1.12E-02 | 3.18 |
| F1 | KEGG_PATHWAY | hsa04151:PI3K-Akt signaling pathway | 8 | 2.89E-02 | 2.63 |
| F2 | KEGG_PATHWAY | hsa05216:Thyroid cancer | 5 | 2.88E-04 | 15.08 |
| F2 | KEGG_PATHWAY | hsa04370:VEGF signaling pathway | 9 | 3.33E-07 | 12.91 |
| F2 | KEGG_PATHWAY | hsa04913:Ovarian steroidogenesis | 7 | 1.62E-05 | 12.50 |
| F2 | KEGG_PATHWAY | hsa05223:Non-small cell lung cancer | 7 | 3.53E-05 | 10.93 |
| F2 | KEGG_PATHWAY | hsa05219:Bladder cancer | 5 | 1.11E-03 | 10.67 |
| F2 | KEGG_PATHWAY | hsa00140:Steroid hormone biosynthesis | 7 | 4.32E-05 | 10.56 |
| F2 | KEGG_PATHWAY | hsa04320:Dorso-ventral axis formation | 3 | 3.68E-02 | 9.72 |
| F2 | KEGG_PATHWAY | hsa05221:Acute myeloid leukemia | 6 | 3.92E-04 | 9.37 |
| F2 | KEGG_PATHWAY | hsa00591:Linoleic acid metabolism | 3 | 4.20E-02 | 9.05 |
| F2 | KEGG_PATHWAY | hsa04664:Fc epsilon RI signaling pathway | 7 | 1.07E-04 | 9.00 |
| F2 | KEGG_PATHWAY | hsa04960:Aldosterone-regulated sodium reabsorption | 4 | 9.45E-03 | 8.97 |
| F2 | KEGG_PATHWAY | hsa00590:Arachidonic acid metabolism | 6 | 6.31E-04 | 8.46 |
| F2 | KEGG_PATHWAY | hsa05214:Glioma | 6 | 7.84E-04 | 8.07 |
| F2 | KEGG_PATHWAY | hsa04012:ErbB signaling pathway | 8 | 5.04E-05 | 8.04 |
| F2 | KEGG_PATHWAY | hsa04066:HIF-1 signaling pathway | 9 | 1.27E-05 | 8.03 |
| F2 | KEGG_PATHWAY | hsa03320:PPAR signaling pathway | 6 | 9.01E-04 | 7.83 |
| F2 | KEGG_PATHWAY | hsa04961:Endocrine and other factor-regulated calcium reabsorption | 4 | 1.40E-02 | 7.77 |
| F2 | KEGG_PATHWAY | hsa04150:mTOR signaling pathway | 5 | 4.02E-03 | 7.54 |
| F2 | KEGG_PATHWAY | hsa04730:Long-term depression | 5 | 4.54E-03 | 7.29 |
| F2 | KEGG_PATHWAY | hsa05222:Small cell lung cancer | 7 | 3.67E-04 | 7.20 |
| F2 | KEGG_PATHWAY | hsa05231:Choline metabolism in cancer | 8 | 1.31E-04 | 6.93 |
| F2 | KEGG_PATHWAY | hsa04919:Thyroid hormone signaling pathway | 9 | 3.85E-05 | 6.91 |
| F2 | KEGG_PATHWAY | hsa05213:Endometrial cancer | 4 | 2.06E-02 | 6.73 |
| F2 | KEGG_PATHWAY | hsa05212:Pancreatic cancer | 5 | 6.04E-03 | 6.73 |
| F2 | KEGG_PATHWAY | hsa04912:GnRH signaling pathway | 7 | 5.30E-04 | 6.73 |
| F2 | KEGG_PATHWAY | hsa05110:Vibrio cholerae infection | 4 | 2.16E-02 | 6.60 |
| F2 | KEGG_PATHWAY | hsa05204:Chemical carcinogenesis | 6 | 2.00E-03 | 6.56 |
| F2 | KEGG_PATHWAY | hsa04662:B cell receptor signaling pathway | 5 | 7.46E-03 | 6.34 |
| F2 | KEGG_PATHWAY | hsa04726:Serotonergic synapse | 8 | 2.36E-04 | 6.30 |
| F2 | KEGG_PATHWAY | hsa04915:Estrogen signaling pathway | 7 | 8.30E-04 | 6.18 |
| F2 | KEGG_PATHWAY | hsa04917:Prolactin signaling pathway | 5 | 8.24E-03 | 6.16 |
| F2 | KEGG_PATHWAY | hsa05416:Viral myocarditis | 4 | 2.62E-02 | 6.14 |
| F2 | KEGG_PATHWAY | hsa05220:Chronic myeloid leukemia | 5 | 8.65E-03 | 6.07 |
| F2 | KEGG_PATHWAY | hsa05215:Prostate cancer | 6 | 3.04E-03 | 5.96 |
| F2 | KEGG_PATHWAY | hsa05032:Morphine addiction | 6 | 3.52E-03 | 5.77 |
| F2 | KEGG_PATHWAY | hsa05210:Colorectal cancer | 4 | 3.25E-02 | 5.64 |
| F2 | KEGG_PATHWAY | hsa04014:Ras signaling pathway | 14 | 1.16E-06 | 5.42 |
| F2 | KEGG_PATHWAY | hsa04720:Long-term potentiation | 4 | 3.81E-02 | 5.30 |
| F2 | KEGG_PATHWAY | hsa05205:Proteoglycans in cancer | 12 | 1.29E-05 | 5.25 |
| F2 | KEGG_PATHWAY | hsa04666:Fc gamma R-mediated phagocytosis | 5 | 1.47E-02 | 5.21 |
| F2 | KEGG_PATHWAY | hsa04510:Focal adhesion | 12 | 1.71E-05 | 5.10 |
| F2 | KEGG_PATHWAY | hsa04976:Bile secretion | 4 | 4.26E-02 | 5.07 |
| F2 | KEGG_PATHWAY | hsa04650:Natural killer cell mediated cytotoxicity | 7 | 2.44E-03 | 5.02 |
| F2 | KEGG_PATHWAY | hsa04920:Adipocytokine signaling pathway | 4 | 4.42E-02 | 5.00 |
| F2 | KEGG_PATHWAY | hsa04015:Rap1 signaling pathway | 12 | 2.05E-05 | 5.00 |
| F2 | KEGG_PATHWAY | hsa04540:Gap junction | 5 | 1.71E-02 | 4.97 |
| F2 | KEGG_PATHWAY | hsa04668:TNF signaling pathway | 6 | 6.72E-03 | 4.95 |
| F2 | KEGG_PATHWAY | hsa05140:Leishmaniasis | 4 | 4.58E-02 | 4.93 |
| F2 | KEGG_PATHWAY | hsa05161:Hepatitis B | 8 | 1.18E-03 | 4.83 |
| F2 | KEGG_PATHWAY | hsa05200:Pathways in cancer | 21 | 7.24E-09 | 4.67 |
| F2 | KEGG_PATHWAY | hsa05145:Toxoplasmosis | 6 | 1.05E-02 | 4.45 |
| F2 | KEGG_PATHWAY | hsa04670:Leukocyte transendothelial migration | 6 | 1.05E-02 | 4.45 |
| F2 | KEGG_PATHWAY | hsa04270:Vascular smooth muscle contraction | 6 | 1.08E-02 | 4.41 |
| F2 | KEGG_PATHWAY | hsa04916:Melanogenesis | 5 | 2.61E-02 | 4.37 |
| F2 | KEGG_PATHWAY | hsa04071:Sphingolipid signaling pathway | 6 | 1.12E-02 | 4.37 |
| F2 | KEGG_PATHWAY | hsa04380:Osteoclast differentiation | 6 | 1.59E-02 | 4.01 |
| F2 | KEGG_PATHWAY | hsa04024:cAMP signaling pathway | 9 | 1.66E-03 | 3.98 |
| F2 | KEGG_PATHWAY | hsa05160:Hepatitis C | 6 | 1.69E-02 | 3.95 |
| F2 | KEGG_PATHWAY | hsa04921:Oxytocin signaling pathway | 7 | 8.61E-03 | 3.88 |
| F2 | KEGG_PATHWAY | hsa04910:Insulin signaling pathway | 6 | 1.95E-02 | 3.80 |
| F2 | KEGG_PATHWAY | hsa04151:PI3K-Akt signaling pathway | 15 | 2.55E-05 | 3.80 |
| F2 | KEGG_PATHWAY | hsa04722:Neurotrophin signaling pathway | 5 | 4.62E-02 | 3.64 |
| F2 | KEGG_PATHWAY | hsa00230:Purine metabolism | 7 | 1.42E-02 | 3.48 |
| F2 | KEGG_PATHWAY | hsa05206:MicroRNAs in cancer | 11 | 1.27E-03 | 3.38 |
| F2 | KEGG_PATHWAY | hsa05164:Influenza A | 6 | 4.62E-02 | 3.02 |
| F2 | KEGG_PATHWAY | hsa04810:Regulation of actin cytoskeleton | 7 | 3.14E-02 | 2.90 |
| F2 | KEGG_PATHWAY | hsa04010:MAPK signaling pathway | 8 | 2.44E-02 | 2.74 |
| F2 | KEGG_PATHWAY | hsa01100:Metabolic pathways | 25 | 3.45E-03 | 1.78 |
| F3 | KEGG_PATHWAY | hsa05219:Bladder cancer | 6 | 5.21E-05 | 14.24 |
| F3 | KEGG_PATHWAY | hsa04370:VEGF signaling pathway | 8 | 2.28E-06 | 12.76 |
| F3 | KEGG_PATHWAY | hsa05221:Acute myeloid leukemia | 7 | 1.89E-05 | 12.17 |
| F3 | KEGG_PATHWAY | hsa04913:Ovarian steroidogenesis | 6 | 1.25E-04 | 11.92 |
| F3 | KEGG_PATHWAY | hsa00140:Steroid hormone biosynthesis | 7 | 2.32E-05 | 11.75 |
| F3 | KEGG_PATHWAY | hsa00591:Linoleic acid metabolism | 3 | 3.44E-02 | 10.07 |
| F3 | KEGG_PATHWAY | hsa00590:Arachidonic acid metabolism | 5 | 3.46E-03 | 7.85 |
| F3 | KEGG_PATHWAY | hsa05204:Chemical carcinogenesis | 6 | 1.23E-03 | 7.30 |
| F3 | KEGG_PATHWAY | hsa05222:Small cell lung cancer | 6 | 1.62E-03 | 6.87 |
| F3 | KEGG_PATHWAY | hsa04012:ErbB signaling pathway | 6 | 1.79E-03 | 6.71 |
| F3 | KEGG_PATHWAY | hsa05230:Central carbon metabolism in cancer | 4 | 2.67E-02 | 6.08 |
| F3 | KEGG_PATHWAY | hsa04066:HIF-1 signaling pathway | 6 | 3.03E-03 | 5.96 |
| F3 | KEGG_PATHWAY | hsa04915:Estrogen signaling pathway | 6 | 3.16E-03 | 5.90 |
| F3 | KEGG_PATHWAY | hsa03320:PPAR signaling pathway | 4 | 3.01E-02 | 5.81 |
| F3 | KEGG_PATHWAY | hsa04664:Fc epsilon RI signaling pathway | 4 | 3.12E-02 | 5.72 |
| F3 | KEGG_PATHWAY | hsa04662:B cell receptor signaling pathway | 4 | 3.24E-02 | 5.64 |
| F3 | KEGG_PATHWAY | hsa04917:Prolactin signaling pathway | 4 | 3.49E-02 | 5.48 |
| F3 | KEGG_PATHWAY | hsa05220:Chronic myeloid leukemia | 4 | 3.61E-02 | 5.41 |
| F3 | KEGG_PATHWAY | hsa04912:GnRH signaling pathway | 5 | 1.33E-02 | 5.35 |
| F3 | KEGG_PATHWAY | hsa00980:Metabolism of xenobiotics by cytochrome P450 | 4 | 3.87E-02 | 5.26 |
| F3 | KEGG_PATHWAY | hsa04014:Ras signaling pathway | 12 | 1.42E-05 | 5.17 |
| F3 | KEGG_PATHWAY | hsa04919:Thyroid hormone signaling pathway | 6 | 5.78E-03 | 5.12 |
| F3 | KEGG_PATHWAY | hsa05231:Choline metabolism in cancer | 5 | 1.89E-02 | 4.82 |
| F3 | KEGG_PATHWAY | hsa00230:Purine metabolism | 8 | 1.91E-03 | 4.42 |
| F3 | KEGG_PATHWAY | hsa05200:Pathways in cancer | 17 | 1.29E-06 | 4.21 |
| F3 | KEGG_PATHWAY | hsa04015:Rap1 signaling pathway | 9 | 1.19E-03 | 4.17 |
| F3 | KEGG_PATHWAY | hsa05161:Hepatitis B | 6 | 1.54E-02 | 4.03 |
| F3 | KEGG_PATHWAY | hsa05205:Proteoglycans in cancer | 8 | 3.92E-03 | 3.89 |
| F3 | KEGG_PATHWAY | hsa04510:Focal adhesion | 8 | 4.62E-03 | 3.78 |
| F3 | KEGG_PATHWAY | hsa04921:Oxytocin signaling pathway | 6 | 2.16E-02 | 3.70 |
| F3 | KEGG_PATHWAY | hsa04024:cAMP signaling pathway | 7 | 1.47E-02 | 3.44 |
| F3 | KEGG_PATHWAY | hsa05206:MicroRNAs in cancer | 10 | 2.17E-03 | 3.41 |
| F3 | KEGG_PATHWAY | hsa04151:PI3K-Akt signaling pathway | 12 | 6.25E-04 | 3.39 |
| F3 | KEGG_PATHWAY | hsa04144:Endocytosis | 7 | 4.58E-02 | 2.64 |
| F3 | KEGG_PATHWAY | hsa01100:Metabolic pathways | 22 | 8.26E-03 | 1.74 |
| F1 | GOTERM_BP_DIRECT | GO:0006700~C21-steroid hormone biosynthetic process | 4 | 5.48E-06 | 106.62 |
| F1 | GOTERM_BP_DIRECT | GO:0006776~vitamin A metabolic process | 3 | 3.45E-04 | 102.81 |
| F1 | GOTERM_BP_DIRECT | GO:0006704~glucocorticoid biosynthetic process | 3 | 5.88E-04 | 79.96 |
| F1 | GOTERM_BP_DIRECT | GO:0043114~regulation of vascular permeability | 3 | 7.33E-04 | 71.97 |
| F1 | GOTERM_BP_DIRECT | GO:0010745~negative regulation of macrophage derived foam cell differentiation | 3 | 1.26E-03 | 55.36 |
| F1 | GOTERM_BP_DIRECT | GO:0031641~regulation of myelination | 3 | 1.26E-03 | 55.36 |
| F1 | GOTERM_BP_DIRECT | GO:0033189~response to vitamin A | 4 | 5.18E-05 | 53.31 |
| F1 | GOTERM_BP_DIRECT | GO:2000251~positive regulation of actin cytoskeleton reorganization | 3 | 1.69E-03 | 47.98 |
| F1 | GOTERM_BP_DIRECT | GO:0006198~cAMP catabolic process | 3 | 1.69E-03 | 47.98 |
| F1 | GOTERM_BP_DIRECT | GO:0016125~sterol metabolic process | 4 | 8.38E-05 | 45.69 |
| F1 | GOTERM_BP_DIRECT | GO:0048011~neurotrophin TRK receptor signaling pathway | 3 | 1.92E-03 | 44.98 |
| F1 | GOTERM_BP_DIRECT | GO:0060324~face development | 3 | 2.44E-03 | 39.98 |
| F1 | GOTERM_BP_DIRECT | GO:0043401~steroid hormone mediated signaling pathway | 9 | 7.53E-11 | 37.88 |
| F1 | GOTERM_BP_DIRECT | GO:0043393~regulation of protein binding | 3 | 2.72E-03 | 37.88 |
| F1 | GOTERM_BP_DIRECT | GO:0071375~cellular response to peptide hormone stimulus | 3 | 3.01E-03 | 35.98 |
| F1 | GOTERM_BP_DIRECT | GO:0046697~decidualization | 3 | 3.01E-03 | 35.98 |
| F1 | GOTERM_BP_DIRECT | GO:0008202~steroid metabolic process | 6 | 8.62E-07 | 33.47 |
| F1 | GOTERM_BP_DIRECT | GO:0043552~positive regulation of phosphatidylinositol 3-kinase activity | 3 | 7.17E-03 | 23.21 |
| F1 | GOTERM_BP_DIRECT | GO:0007566~embryo implantation | 4 | 6.80E-04 | 22.85 |
| F1 | GOTERM_BP_DIRECT | GO:0030522~intracellular receptor signaling pathway | 3 | 1.06E-02 | 18.94 |
| F1 | GOTERM_BP_DIRECT | GO:0034446~substrate adhesion-dependent cell spreading | 3 | 1.06E-02 | 18.94 |
| F1 | GOTERM_BP_DIRECT | GO:0018107~peptidyl-threonine phosphorylation | 3 | 1.06E-02 | 18.94 |
| F1 | GOTERM_BP_DIRECT | GO:0042060~wound healing | 6 | 1.91E-05 | 17.99 |
| F1 | GOTERM_BP_DIRECT | GO:0038083~peptidyl-tyrosine autophosphorylation | 3 | 1.17E-02 | 17.99 |
| F1 | GOTERM_BP_DIRECT | GO:0008542~visual learning | 3 | 1.47E-02 | 15.99 |
| F1 | GOTERM_BP_DIRECT | GO:0006367~transcription initiation from RNA polymerase II promoter | 10 | 1.15E-08 | 15.78 |
| F1 | GOTERM_BP_DIRECT | GO:0030574~collagen catabolic process | 4 | 2.31E-03 | 14.99 |
| F1 | GOTERM_BP_DIRECT | GO:0042752~regulation of circadian rhythm | 3 | 1.73E-02 | 14.69 |
| F1 | GOTERM_BP_DIRECT | GO:0008203~cholesterol metabolic process | 4 | 2.75E-03 | 14.11 |
| F1 | GOTERM_BP_DIRECT | GO:0048511~rhythmic process | 3 | 2.08E-02 | 13.33 |
| F1 | GOTERM_BP_DIRECT | GO:0001934~positive regulation of protein phosphorylation | 7 | 1.35E-05 | 13.22 |
| F1 | GOTERM_BP_DIRECT | GO:0007584~response to nutrient | 4 | 3.49E-03 | 12.97 |
| F1 | GOTERM_BP_DIRECT | GO:0007623~circadian rhythm | 4 | 3.63E-03 | 12.79 |
| F1 | GOTERM_BP_DIRECT | GO:0022617~extracellular matrix disassembly | 4 | 3.77E-03 | 12.63 |
| F1 | GOTERM_BP_DIRECT | GO:0046777~protein autophosphorylation | 9 | 5.06E-07 | 12.55 |
| F1 | GOTERM_BP_DIRECT | GO:0018108~peptidyl-tyrosine phosphorylation | 8 | 3.05E-06 | 12.54 |
| F1 | GOTERM_BP_DIRECT | GO:0007169~transmembrane receptor protein tyrosine kinase signaling pathway | 5 | 6.53E-04 | 12.49 |
| F1 | GOTERM_BP_DIRECT | GO:0071456~cellular response to hypoxia | 5 | 6.53E-04 | 12.49 |
| F1 | GOTERM_BP_DIRECT | GO:0043406~positive regulation of MAP kinase activity | 3 | 2.45E-02 | 12.20 |
| F1 | GOTERM_BP_DIRECT | GO:0050728~negative regulation of inflammatory response | 4 | 4.20E-03 | 12.15 |
| F1 | GOTERM_BP_DIRECT | GO:0035264~multicellular organism growth | 4 | 4.35E-03 | 11.99 |
| F1 | GOTERM_BP_DIRECT | GO:0048661~positive regulation of smooth muscle cell proliferation | 3 | 2.53E-02 | 11.99 |
| F1 | GOTERM_BP_DIRECT | GO:0048013~ephrin receptor signaling pathway | 4 | 5.33E-03 | 11.16 |
| F1 | GOTERM_BP_DIRECT | GO:0014068~positive regulation of phosphatidylinositol 3-kinase signaling | 3 | 2.93E-02 | 11.07 |
| F1 | GOTERM_BP_DIRECT | GO:0030183~B cell differentiation | 3 | 3.01E-02 | 10.90 |
| F1 | GOTERM_BP_DIRECT | GO:0007059~chromosome segregation | 3 | 3.18E-02 | 10.58 |
| F1 | GOTERM_BP_DIRECT | GO:0007507~heart development | 8 | 9.93E-06 | 10.49 |
| F1 | GOTERM_BP_DIRECT | GO:0001938~positive regulation of endothelial cell proliferation | 3 | 3.27E-02 | 10.43 |
| F1 | GOTERM_BP_DIRECT | GO:0097190~apoptotic signaling pathway | 3 | 3.45E-02 | 10.14 |
| F1 | GOTERM_BP_DIRECT | GO:0048010~vascular endothelial growth factor receptor signaling pathway | 3 | 3.54E-02 | 10.00 |
| F1 | GOTERM_BP_DIRECT | GO:0050900~leukocyte migration | 5 | 1.59E-03 | 9.83 |
| F1 | GOTERM_BP_DIRECT | GO:0001666~response to hypoxia | 7 | 7.44E-05 | 9.76 |
| F1 | GOTERM_BP_DIRECT | GO:0016310~phosphorylation | 4 | 8.08E-03 | 9.60 |
| F1 | GOTERM_BP_DIRECT | GO:0001843~neural tube closure | 3 | 4.00E-02 | 9.35 |
| F1 | GOTERM_BP_DIRECT | GO:0006805~xenobiotic metabolic process | 3 | 4.09E-02 | 9.23 |
| F1 | GOTERM_BP_DIRECT | GO:0048015~phosphatidylinositol-mediated signaling | 4 | 9.47E-03 | 9.05 |
| F1 | GOTERM_BP_DIRECT | GO:0050731~positive regulation of peptidyl-tyrosine phosphorylation | 3 | 4.48E-02 | 8.78 |
| F1 | GOTERM_BP_DIRECT | GO:0051897~positive regulation of protein kinase B signaling | 3 | 4.67E-02 | 8.57 |
| F1 | GOTERM_BP_DIRECT | GO:0071222~cellular response to lipopolysaccharide | 4 | 1.13E-02 | 8.49 |
| F1 | GOTERM_BP_DIRECT | GO:0045766~positive regulation of angiogenesis | 4 | 1.18E-02 | 8.34 |
| F1 | GOTERM_BP_DIRECT | GO:0070374~positive regulation of ERK1 and ERK2 cascade | 6 | 7.61E-04 | 8.22 |
| F1 | GOTERM_BP_DIRECT | GO:0018105~peptidyl-serine phosphorylation | 4 | 1.48E-02 | 7.68 |
| F1 | GOTERM_BP_DIRECT | GO:0007568~aging | 5 | 4.73E-03 | 7.27 |
| F1 | GOTERM_BP_DIRECT | GO:0042493~response to drug | 9 | 3.37E-05 | 7.10 |
| F1 | GOTERM_BP_DIRECT | GO:0010628~positive regulation of gene expression | 7 | 7.18E-04 | 6.41 |
| F1 | GOTERM_BP_DIRECT | GO:0055114~oxidation-reduction process | 15 | 1.02E-07 | 6.08 |
| F1 | GOTERM_BP_DIRECT | GO:0016477~cell migration | 4 | 3.38E-02 | 5.58 |
| F1 | GOTERM_BP_DIRECT | GO:0043066~negative regulation of apoptotic process | 10 | 9.75E-05 | 5.27 |
| F1 | GOTERM_BP_DIRECT | GO:0008285~negative regulation of cell proliferation | 8 | 1.18E-03 | 4.85 |
| F1 | GOTERM_BP_DIRECT | GO:0043065~positive regulation of apoptotic process | 6 | 7.79E-03 | 4.80 |
| F1 | GOTERM_BP_DIRECT | GO:0008283~cell proliferation | 7 | 3.89E-03 | 4.59 |
| F1 | GOTERM_BP_DIRECT | GO:0000165~MAPK cascade | 5 | 2.27E-02 | 4.58 |
| F1 | GOTERM_BP_DIRECT | GO:0006468~protein phosphorylation | 8 | 2.63E-03 | 4.21 |
| F1 | GOTERM_BP_DIRECT | GO:0045892~negative regulation of transcription, DNA-templated | 8 | 4.32E-03 | 3.85 |
| F1 | GOTERM_BP_DIRECT | GO:0007165~signal transduction | 17 | 1.43E-05 | 3.51 |
| F1 | GOTERM_BP_DIRECT | GO:0045893~positive regulation of transcription, DNA-templated | 7 | 1.90E-02 | 3.26 |
| F1 | GOTERM_BP_DIRECT | GO:0045944~positive regulation of transcription from RNA polymerase II promoter | 13 | 5.93E-04 | 3.18 |
| F1 | GOTERM_BP_DIRECT | GO:0008284~positive regulation of cell proliferation | 6 | 4.25E-02 | 3.09 |
| F1 | GOTERM_BP_DIRECT | GO:0000122~negative regulation of transcription from RNA polymerase II promoter | 9 | 9.15E-03 | 3.00 |
| F2 | GOTERM_BP_DIRECT | GO:0060440~trachea formation | 3 | 4.68E-04 | 87.46 |
| F2 | GOTERM_BP_DIRECT | GO:0006700~C21-steroid hormone biosynthetic process | 4 | 1.44E-05 | 77.74 |
| F2 | GOTERM_BP_DIRECT | GO:0006776~vitamin A metabolic process | 3 | 6.53E-04 | 74.96 |
| F2 | GOTERM_BP_DIRECT | GO:0042908~xenobiotic transport | 3 | 6.53E-04 | 74.96 |
| F2 | GOTERM_BP_DIRECT | GO:0010863~positive regulation of phospholipase C activity | 3 | 1.11E-03 | 58.31 |
| F2 | GOTERM_BP_DIRECT | GO:0060020~Bergmann glial cell differentiation | 3 | 1.11E-03 | 58.31 |
| F2 | GOTERM_BP_DIRECT | GO:0006704~glucocorticoid biosynthetic process | 3 | 1.11E-03 | 58.31 |
| F2 | GOTERM_BP_DIRECT | GO:0031641~regulation of myelination | 4 | 4.82E-05 | 53.82 |
| F2 | GOTERM_BP_DIRECT | GO:0010745~negative regulation of macrophage derived foam cell differentiation | 4 | 4.82E-05 | 53.82 |
| F2 | GOTERM_BP_DIRECT | GO:0042359~vitamin D metabolic process | 3 | 1.38E-03 | 52.48 |
| F2 | GOTERM_BP_DIRECT | GO:0033189~response to vitamin A | 5 | 2.77E-06 | 48.59 |
| F2 | GOTERM_BP_DIRECT | GO:0006198~cAMP catabolic process | 4 | 7.60E-05 | 46.64 |
| F2 | GOTERM_BP_DIRECT | GO:0016125~sterol metabolic process | 5 | 5.34E-06 | 41.65 |
| F2 | GOTERM_BP_DIRECT | GO:0019372~lipoxygenase pathway | 3 | 2.37E-03 | 40.37 |
| F2 | GOTERM_BP_DIRECT | GO:0043101~purine-containing compound salvage | 3 | 2.76E-03 | 37.48 |
| F2 | GOTERM_BP_DIRECT | GO:0046697~decidualization | 4 | 1.86E-04 | 34.98 |
| F2 | GOTERM_BP_DIRECT | GO:0043401~steroid hormone mediated signaling pathway | 11 | 7.17E-13 | 33.76 |
| F2 | GOTERM_BP_DIRECT | GO:0030949~positive regulation of vascular endothelial growth factor receptor signaling pathway | 3 | 3.61E-03 | 32.80 |
| F2 | GOTERM_BP_DIRECT | GO:0032331~negative regulation of chondrocyte differentiation | 3 | 4.08E-03 | 30.87 |
| F2 | GOTERM_BP_DIRECT | GO:0038083~peptidyl-tyrosine autophosphorylation | 7 | 9.19E-08 | 30.61 |
| F2 | GOTERM_BP_DIRECT | GO:0007566~embryo implantation | 7 | 1.24E-07 | 29.15 |
| F2 | GOTERM_BP_DIRECT | GO:0048384~retinoic acid receptor signaling pathway | 3 | 4.57E-03 | 29.15 |
| F2 | GOTERM_BP_DIRECT | GO:0060324~face development | 3 | 4.57E-03 | 29.15 |
| F2 | GOTERM_BP_DIRECT | GO:0008202~steroid metabolic process | 6 | 4.23E-06 | 24.41 |
| F2 | GOTERM_BP_DIRECT | GO:2000352~negative regulation of endothelial cell apoptotic process | 3 | 1.09E-02 | 18.74 |
| F2 | GOTERM_BP_DIRECT | GO:0035116~embryonic hindlimb morphogenesis | 3 | 1.09E-02 | 18.74 |
| F2 | GOTERM_BP_DIRECT | GO:0034446~substrate adhesion-dependent cell spreading | 4 | 1.28E-03 | 18.41 |
| F2 | GOTERM_BP_DIRECT | GO:0045909~positive regulation of vasodilation | 3 | 1.16E-02 | 18.09 |
| F2 | GOTERM_BP_DIRECT | GO:0045776~negative regulation of blood pressure | 3 | 1.24E-02 | 17.49 |
| F2 | GOTERM_BP_DIRECT | GO:0031663~lipopolysaccharide-mediated signaling pathway | 3 | 1.41E-02 | 16.40 |
| F2 | GOTERM_BP_DIRECT | GO:0048538~thymus development | 4 | 1.84E-03 | 16.27 |
| F2 | GOTERM_BP_DIRECT | GO:0070555~response to interleukin-1 | 3 | 1.49E-02 | 15.90 |
| F2 | GOTERM_BP_DIRECT | GO:0010595~positive regulation of endothelial cell migration | 4 | 2.23E-03 | 15.21 |
| F2 | GOTERM_BP_DIRECT | GO:0001890~placenta development | 3 | 1.67E-02 | 14.99 |
| F2 | GOTERM_BP_DIRECT | GO:0006367~transcription initiation from RNA polymerase II promoter | 12 | 9.44E-10 | 13.81 |
| F2 | GOTERM_BP_DIRECT | GO:0030522~intracellular receptor signaling pathway | 3 | 1.95E-02 | 13.81 |
| F2 | GOTERM_BP_DIRECT | GO:0018107~peptidyl-threonine phosphorylation | 3 | 1.95E-02 | 13.81 |
| F2 | GOTERM_BP_DIRECT | GO:0008203~cholesterol metabolic process | 5 | 5.94E-04 | 12.86 |
| F2 | GOTERM_BP_DIRECT | GO:0032526~response to retinoic acid | 3 | 2.25E-02 | 12.80 |
| F2 | GOTERM_BP_DIRECT | GO:0007169~transmembrane receptor protein tyrosine kinase signaling pathway | 7 | 1.72E-05 | 12.75 |
| F2 | GOTERM_BP_DIRECT | GO:0001938~positive regulation of endothelial cell proliferation | 5 | 6.27E-04 | 12.68 |
| F2 | GOTERM_BP_DIRECT | GO:0007595~lactation | 3 | 2.35E-02 | 12.49 |
| F2 | GOTERM_BP_DIRECT | GO:0045429~positive regulation of nitric oxide biosynthetic process | 3 | 2.46E-02 | 12.20 |
| F2 | GOTERM_BP_DIRECT | GO:0043525~positive regulation of neuron apoptotic process | 3 | 2.46E-02 | 12.20 |
| F2 | GOTERM_BP_DIRECT | GO:0048661~positive regulation of smooth muscle cell proliferation | 4 | 4.75E-03 | 11.66 |
| F2 | GOTERM_BP_DIRECT | GO:0050727~regulation of inflammatory response | 4 | 5.45E-03 | 11.11 |
| F2 | GOTERM_BP_DIRECT | GO:0050728~negative regulation of inflammatory response | 5 | 1.04E-03 | 11.07 |
| F2 | GOTERM_BP_DIRECT | GO:0035264~multicellular organism growth | 5 | 1.09E-03 | 10.93 |
| F2 | GOTERM_BP_DIRECT | GO:0030574~collagen catabolic process | 4 | 5.70E-03 | 10.93 |
| F2 | GOTERM_BP_DIRECT | GO:0043627~response to estrogen | 4 | 5.95E-03 | 10.76 |
| F2 | GOTERM_BP_DIRECT | GO:0014068~positive regulation of phosphatidylinositol 3-kinase signaling | 4 | 5.95E-03 | 10.76 |
| F2 | GOTERM_BP_DIRECT | GO:0042752~regulation of circadian rhythm | 3 | 3.14E-02 | 10.71 |
| F2 | GOTERM_BP_DIRECT | GO:0045766~positive regulation of angiogenesis | 7 | 4.79E-05 | 10.65 |
| F2 | GOTERM_BP_DIRECT | GO:0050900~leukocyte migration | 7 | 6.67E-05 | 10.04 |
| F2 | GOTERM_BP_DIRECT | GO:0097190~apoptotic signaling pathway | 4 | 7.59E-03 | 9.85 |
| F2 | GOTERM_BP_DIRECT | GO:0050853~B cell receptor signaling pathway | 3 | 3.75E-02 | 9.72 |
| F2 | GOTERM_BP_DIRECT | GO:0016049~cell growth | 3 | 4.00E-02 | 9.37 |
| F2 | GOTERM_BP_DIRECT | GO:0022617~extracellular matrix disassembly | 4 | 9.15E-03 | 9.21 |
| F2 | GOTERM_BP_DIRECT | GO:0001666~response to hypoxia | 9 | 5.87E-06 | 9.15 |
| F2 | GOTERM_BP_DIRECT | GO:0018108~peptidyl-tyrosine phosphorylation | 8 | 2.57E-05 | 9.15 |
| F2 | GOTERM_BP_DIRECT | GO:0071456~cellular response to hypoxia | 5 | 2.15E-03 | 9.11 |
| F2 | GOTERM_BP_DIRECT | GO:0042060~wound healing | 4 | 1.05E-02 | 8.75 |
| F2 | GOTERM_BP_DIRECT | GO:0043410~positive regulation of MAPK cascade | 4 | 1.09E-02 | 8.64 |
| F2 | GOTERM_BP_DIRECT | GO:0006950~response to stress | 3 | 4.67E-02 | 8.60 |
| F2 | GOTERM_BP_DIRECT | GO:0001934~positive regulation of protein phosphorylation | 6 | 7.69E-04 | 8.26 |
| F2 | GOTERM_BP_DIRECT | GO:0007160~cell-matrix adhesion | 4 | 1.45E-02 | 7.77 |
| F2 | GOTERM_BP_DIRECT | GO:0071222~cellular response to lipopolysaccharide | 5 | 3.87E-03 | 7.74 |
| F2 | GOTERM_BP_DIRECT | GO:0016477~cell migration | 7 | 4.33E-04 | 7.12 |
| F2 | GOTERM_BP_DIRECT | GO:0046777~protein autophosphorylation | 7 | 4.33E-04 | 7.12 |
| F2 | GOTERM_BP_DIRECT | GO:0007229~integrin-mediated signaling pathway | 4 | 1.86E-02 | 7.07 |
| F2 | GOTERM_BP_DIRECT | GO:0018105~peptidyl-serine phosphorylation | 5 | 5.53E-03 | 7.00 |
| F2 | GOTERM_BP_DIRECT | GO:0042493~response to drug | 12 | 1.16E-06 | 6.90 |
| F2 | GOTERM_BP_DIRECT | GO:0051091~positive regulation of sequence-specific DNA binding transcription factor activity | 4 | 2.17E-02 | 6.66 |
| F2 | GOTERM_BP_DIRECT | GO:0042127~regulation of cell proliferation | 7 | 6.36E-04 | 6.62 |
| F2 | GOTERM_BP_DIRECT | GO:0043123~positive regulation of I-kappaB kinase/NF-kappaB signaling | 6 | 2.20E-03 | 6.52 |
| F2 | GOTERM_BP_DIRECT | GO:0001525~angiogenesis | 8 | 2.72E-04 | 6.28 |
| F2 | GOTERM_BP_DIRECT | GO:0030168~platelet activation | 4 | 2.75E-02 | 6.08 |
| F2 | GOTERM_BP_DIRECT | GO:0070374~positive regulation of ERK1 and ERK2 cascade | 6 | 3.16E-03 | 6.00 |
| F2 | GOTERM_BP_DIRECT | GO:0043065~positive regulation of apoptotic process | 10 | 5.04E-05 | 5.83 |
| F2 | GOTERM_BP_DIRECT | GO:0055114~oxidation-reduction process | 19 | 4.86E-09 | 5.61 |
| F2 | GOTERM_BP_DIRECT | GO:0007568~aging | 5 | 1.43E-02 | 5.30 |
| F2 | GOTERM_BP_DIRECT | GO:0051092~positive regulation of NF-kappaB transcription factor activity | 4 | 3.97E-02 | 5.26 |
| F2 | GOTERM_BP_DIRECT | GO:0010629~negative regulation of gene expression | 4 | 4.27E-02 | 5.11 |
| F2 | GOTERM_BP_DIRECT | GO:0007507~heart development | 5 | 2.02E-02 | 4.78 |
| F2 | GOTERM_BP_DIRECT | GO:0030198~extracellular matrix organization | 5 | 2.52E-02 | 4.46 |
| F2 | GOTERM_BP_DIRECT | GO:0000165~MAPK cascade | 6 | 1.65E-02 | 4.01 |
| F2 | GOTERM_BP_DIRECT | GO:0010628~positive regulation of gene expression | 6 | 1.65E-02 | 4.01 |
| F2 | GOTERM_BP_DIRECT | GO:0008285~negative regulation of cell proliferation | 9 | 1.82E-03 | 3.98 |
| F2 | GOTERM_BP_DIRECT | GO:0043066~negative regulation of apoptotic process | 10 | 1.09E-03 | 3.84 |
| F2 | GOTERM_BP_DIRECT | GO:0006468~protein phosphorylation | 9 | 4.30E-03 | 3.45 |
| F2 | GOTERM_BP_DIRECT | GO:0045893~positive regulation of transcription, DNA-templated | 10 | 2.54E-03 | 3.40 |
| F2 | GOTERM_BP_DIRECT | GO:0045087~innate immune response | 8 | 1.10E-02 | 3.25 |
| F2 | GOTERM_BP_DIRECT | GO:0006954~inflammatory response | 7 | 2.06E-02 | 3.23 |
| F2 | GOTERM_BP_DIRECT | GO:0007165~signal transduction | 21 | 6.17E-06 | 3.16 |
| F2 | GOTERM_BP_DIRECT | GO:0007155~cell adhesion | 8 | 1.53E-02 | 3.05 |
| F2 | GOTERM_BP_DIRECT | GO:0006810~transport | 6 | 4.75E-02 | 3.02 |
| F2 | GOTERM_BP_DIRECT | GO:0008284~positive regulation of cell proliferation | 8 | 1.65E-02 | 3.00 |
| F2 | GOTERM_BP_DIRECT | GO:0045944~positive regulation of transcription from RNA polymerase II promoter | 15 | 1.20E-03 | 2.67 |
| F2 | GOTERM_BP_DIRECT | GO:0000122~negative regulation of transcription from RNA polymerase II promoter | 10 | 2.06E-02 | 2.43 |
| F3 | GOTERM_BP_DIRECT | GO:0002068~glandular epithelial cell development | 3 | 7.22E-05 | 199.90 |
| F3 | GOTERM_BP_DIRECT | GO:0006700~C21-steroid hormone biosynthetic process | 4 | 9.57E-06 | 88.85 |
| F3 | GOTERM_BP_DIRECT | GO:0006776~vitamin A metabolic process | 3 | 4.99E-04 | 85.67 |
| F3 | GOTERM_BP_DIRECT | GO:0042908~xenobiotic transport | 3 | 4.99E-04 | 85.67 |
| F3 | GOTERM_BP_DIRECT | GO:0006704~glucocorticoid biosynthetic process | 3 | 8.50E-04 | 66.63 |
| F3 | GOTERM_BP_DIRECT | GO:0010863~positive regulation of phospholipase C activity | 3 | 8.50E-04 | 66.63 |
| F3 | GOTERM_BP_DIRECT | GO:0031641~regulation of myelination | 4 | 3.21E-05 | 61.51 |
| F3 | GOTERM_BP_DIRECT | GO:0043114~regulation of vascular permeability | 3 | 1.06E-03 | 59.97 |
| F3 | GOTERM_BP_DIRECT | GO:0033189~response to vitamin A | 5 | 1.61E-06 | 55.53 |
| F3 | GOTERM_BP_DIRECT | GO:0006198~cAMP catabolic process | 4 | 5.08E-05 | 53.31 |
| F3 | GOTERM_BP_DIRECT | GO:0071498~cellular response to fluid shear stress | 3 | 1.54E-03 | 49.98 |
| F3 | GOTERM_BP_DIRECT | GO:0016125~sterol metabolic process | 5 | 3.11E-06 | 47.60 |
| F3 | GOTERM_BP_DIRECT | GO:0010745~negative regulation of macrophage derived foam cell differentiation | 3 | 1.82E-03 | 46.13 |
| F3 | GOTERM_BP_DIRECT | GO:0043101~purine-containing compound salvage | 3 | 2.11E-03 | 42.84 |
| F3 | GOTERM_BP_DIRECT | GO:0046697~decidualization | 4 | 1.25E-04 | 39.98 |
| F3 | GOTERM_BP_DIRECT | GO:2000251~positive regulation of actin cytoskeleton reorganization | 3 | 2.43E-03 | 39.98 |
| F3 | GOTERM_BP_DIRECT | GO:1901216~positive regulation of neuron death | 3 | 3.13E-03 | 35.28 |
| F3 | GOTERM_BP_DIRECT | GO:0038083~peptidyl-tyrosine autophosphorylation | 7 | 4.07E-08 | 34.98 |
| F3 | GOTERM_BP_DIRECT | GO:0060324~face development | 3 | 3.51E-03 | 33.32 |
| F3 | GOTERM_BP_DIRECT | GO:0048384~retinoic acid receptor signaling pathway | 3 | 3.51E-03 | 33.32 |
| F3 | GOTERM_BP_DIRECT | GO:0043401~steroid hormone mediated signaling pathway | 9 | 3.43E-10 | 31.56 |
| F3 | GOTERM_BP_DIRECT | GO:0071375~cellular response to peptide hormone stimulus | 3 | 4.33E-03 | 29.99 |
| F3 | GOTERM_BP_DIRECT | GO:0007566~embryo implantation | 6 | 1.92E-06 | 28.56 |
| F3 | GOTERM_BP_DIRECT | GO:0034446~substrate adhesion-dependent cell spreading | 5 | 3.60E-05 | 26.30 |
| F3 | GOTERM_BP_DIRECT | GO:0043552~positive regulation of phosphatidylinositol 3-kinase activity | 4 | 4.74E-04 | 25.79 |
| F3 | GOTERM_BP_DIRECT | GO:0008202~steroid metabolic process | 5 | 5.91E-05 | 23.24 |
| F3 | GOTERM_BP_DIRECT | GO:2000352~negative regulation of endothelial cell apoptotic process | 3 | 8.39E-03 | 21.42 |
| F3 | GOTERM_BP_DIRECT | GO:2000145~regulation of cell motility | 3 | 8.39E-03 | 21.42 |
| F3 | GOTERM_BP_DIRECT | GO:0045909~positive regulation of vasodilation | 3 | 8.99E-03 | 20.68 |
| F3 | GOTERM_BP_DIRECT | GO:0045907~positive regulation of vasoconstriction | 3 | 1.09E-02 | 18.74 |
| F3 | GOTERM_BP_DIRECT | GO:0048167~regulation of synaptic plasticity | 3 | 1.09E-02 | 18.74 |
| F3 | GOTERM_BP_DIRECT | GO:0008542~visual learning | 4 | 1.42E-03 | 17.77 |
| F3 | GOTERM_BP_DIRECT | GO:0001890~placenta development | 3 | 1.29E-02 | 17.13 |
| F3 | GOTERM_BP_DIRECT | GO:0014068~positive regulation of phosphatidylinositol 3-kinase signaling | 5 | 2.99E-04 | 15.38 |
| F3 | GOTERM_BP_DIRECT | GO:0032526~response to retinoic acid | 3 | 1.75E-02 | 14.63 |
| F3 | GOTERM_BP_DIRECT | GO:0007169~transmembrane receptor protein tyrosine kinase signaling pathway | 7 | 7.89E-06 | 14.58 |
| F3 | GOTERM_BP_DIRECT | GO:0006367~transcription initiation from RNA polymerase II promoter | 11 | 3.79E-09 | 14.47 |
| F3 | GOTERM_BP_DIRECT | GO:0043525~positive regulation of neuron apoptotic process | 3 | 1.91E-02 | 13.95 |
| F3 | GOTERM_BP_DIRECT | GO:0048661~positive regulation of smooth muscle cell proliferation | 4 | 3.25E-03 | 13.33 |
| F3 | GOTERM_BP_DIRECT | GO:0018108~peptidyl-tyrosine phosphorylation | 10 | 6.32E-08 | 13.07 |
| F3 | GOTERM_BP_DIRECT | GO:0010595~positive regulation of endothelial cell migration | 3 | 2.17E-02 | 13.04 |
| F3 | GOTERM_BP_DIRECT | GO:0035264~multicellular organism growth | 5 | 6.60E-04 | 12.49 |
| F3 | GOTERM_BP_DIRECT | GO:0030574~collagen catabolic process | 4 | 3.91E-03 | 12.49 |
| F3 | GOTERM_BP_DIRECT | GO:0071222~cellular response to lipopolysaccharide | 7 | 2.01E-05 | 12.38 |
| F3 | GOTERM_BP_DIRECT | GO:0008203~cholesterol metabolic process | 4 | 4.63E-03 | 11.76 |
| F3 | GOTERM_BP_DIRECT | GO:0046777~protein autophosphorylation | 10 | 1.73E-07 | 11.62 |
| F3 | GOTERM_BP_DIRECT | GO:0001938~positive regulation of endothelial cell proliferation | 4 | 4.82E-03 | 11.59 |
| F3 | GOTERM_BP_DIRECT | GO:0071300~cellular response to retinoic acid | 4 | 5.02E-03 | 11.42 |
| F3 | GOTERM_BP_DIRECT | GO:0048010~vascular endothelial growth factor receptor signaling pathway | 4 | 5.43E-03 | 11.11 |
| F3 | GOTERM_BP_DIRECT | GO:0048511~rhythmic process | 3 | 2.93E-02 | 11.11 |
| F3 | GOTERM_BP_DIRECT | GO:0001934~positive regulation of protein phosphorylation | 7 | 3.90E-05 | 11.02 |
| F3 | GOTERM_BP_DIRECT | GO:0022617~extracellular matrix disassembly | 4 | 6.32E-03 | 10.52 |
| F3 | GOTERM_BP_DIRECT | GO:0071456~cellular response to hypoxia | 5 | 1.31E-03 | 10.41 |
| F3 | GOTERM_BP_DIRECT | GO:0043406~positive regulation of MAP kinase activity | 3 | 3.44E-02 | 10.16 |
| F3 | GOTERM_BP_DIRECT | GO:0050728~negative regulation of inflammatory response | 4 | 7.03E-03 | 10.12 |
| F3 | GOTERM_BP_DIRECT | GO:0042060~wound healing | 4 | 7.28E-03 | 10.00 |
| F3 | GOTERM_BP_DIRECT | GO:0043410~positive regulation of MAPK cascade | 4 | 7.53E-03 | 9.87 |
| F3 | GOTERM_BP_DIRECT | GO:0050900~leukocyte migration | 6 | 3.44E-04 | 9.83 |
| F3 | GOTERM_BP_DIRECT | GO:0050731~positive regulation of peptidyl-tyrosine phosphorylation | 4 | 7.79E-03 | 9.75 |
| F3 | GOTERM_BP_DIRECT | GO:0050727~regulation of inflammatory response | 3 | 3.88E-02 | 9.52 |
| F3 | GOTERM_BP_DIRECT | GO:0001666~response to hypoxia | 8 | 2.26E-05 | 9.30 |
| F3 | GOTERM_BP_DIRECT | GO:0008217~regulation of blood pressure | 3 | 4.11E-02 | 9.23 |
| F3 | GOTERM_BP_DIRECT | GO:0007059~chromosome segregation | 3 | 4.46E-02 | 8.82 |
| F3 | GOTERM_BP_DIRECT | GO:0045766~positive regulation of angiogenesis | 5 | 2.53E-03 | 8.69 |
| F3 | GOTERM_BP_DIRECT | GO:0035690~cellular response to drug | 3 | 4.58E-02 | 8.69 |
| F3 | GOTERM_BP_DIRECT | GO:0042127~regulation of cell proliferation | 8 | 3.60E-05 | 8.64 |
| F3 | GOTERM_BP_DIRECT | GO:0097190~apoptotic signaling pathway | 3 | 4.82E-02 | 8.45 |
| F3 | GOTERM_BP_DIRECT | GO:0007229~integrin-mediated signaling pathway | 4 | 1.30E-02 | 8.08 |
| F3 | GOTERM_BP_DIRECT | GO:0016310~phosphorylation | 4 | 1.34E-02 | 8.00 |
| F3 | GOTERM_BP_DIRECT | GO:0070374~positive regulation of ERK1 and ERK2 cascade | 6 | 1.75E-03 | 6.85 |
| F3 | GOTERM_BP_DIRECT | GO:0042493~response to drug | 10 | 1.89E-05 | 6.58 |
| F3 | GOTERM_BP_DIRECT | GO:0001525~angiogenesis | 7 | 8.26E-04 | 6.28 |
| F3 | GOTERM_BP_DIRECT | GO:0016477~cell migration | 5 | 1.04E-02 | 5.81 |
| F3 | GOTERM_BP_DIRECT | GO:0043066~negative regulation of apoptotic process | 13 | 2.30E-06 | 5.71 |
| F3 | GOTERM_BP_DIRECT | GO:0055114~oxidation-reduction process | 16 | 1.79E-07 | 5.40 |
| F3 | GOTERM_BP_DIRECT | GO:0010628~positive regulation of gene expression | 7 | 1.89E-03 | 5.34 |
| F3 | GOTERM_BP_DIRECT | GO:0007568~aging | 4 | 4.84E-02 | 4.85 |
| F3 | GOTERM_BP_DIRECT | GO:0043065~positive regulation of apoptotic process | 7 | 3.69E-03 | 4.66 |
| F3 | GOTERM_BP_DIRECT | GO:0008285~negative regulation of cell proliferation | 9 | 7.52E-04 | 4.54 |
| F3 | GOTERM_BP_DIRECT | GO:0000165~MAPK cascade | 5 | 4.09E-02 | 3.81 |
| F3 | GOTERM_BP_DIRECT | GO:0006954~inflammatory response | 7 | 1.12E-02 | 3.69 |
| F3 | GOTERM_BP_DIRECT | GO:0045892~negative regulation of transcription, DNA-templated | 9 | 3.22E-03 | 3.61 |
| F3 | GOTERM_BP_DIRECT | GO:0045893~positive regulation of transcription, DNA-templated | 9 | 3.90E-03 | 3.49 |
| F3 | GOTERM_BP_DIRECT | GO:0008284~positive regulation of cell proliferation | 8 | 8.21E-03 | 3.43 |
| F3 | GOTERM_BP_DIRECT | GO:0008283~cell proliferation | 6 | 3.49E-02 | 3.28 |
| F3 | GOTERM_BP_DIRECT | GO:0045944~positive regulation of transcription from RNA polymerase II promoter | 16 | 8.17E-05 | 3.26 |
| F3 | GOTERM_BP_DIRECT | GO:0007165~signal transduction | 18 | 4.38E-05 | 3.10 |
| F3 | GOTERM_BP_DIRECT | GO:0006468~protein phosphorylation | 7 | 2.53E-02 | 3.07 |
| F3 | GOTERM_BP_DIRECT | GO:0000122~negative regulation of transcription from RNA polymerase II promoter | 10 | 9.03E-03 | 2.78 |
| F1 | GOTERM_MF_DIRECT | GO:0046965~retinoid X receptor binding | 4 | 2.87E-05 | 64.31 |
| F1 | GOTERM_MF_DIRECT | GO:0004115~3',5'-cyclic-AMP phosphodiesterase activity | 3 | 1.67E-03 | 48.23 |
| F1 | GOTERM_MF_DIRECT | GO:0003707~steroid hormone receptor activity | 10 | 1.21E-12 | 43.06 |
| F1 | GOTERM_MF_DIRECT | GO:0008144~drug binding | 11 | 5.09E-13 | 34.90 |
| F1 | GOTERM_MF_DIRECT | GO:0004114~3',5'-cyclic-nucleotide phosphodiesterase activity | 3 | 3.94E-03 | 31.46 |
| F1 | GOTERM_MF_DIRECT | GO:0008395~steroid hydroxylase activity | 3 | 5.02E-03 | 27.83 |
| F1 | GOTERM_MF_DIRECT | GO:0004879~RNA polymerase II transcription factor activity, ligand-activated sequence-specific DNA binding | 4 | 4.24E-04 | 26.80 |
| F1 | GOTERM_MF_DIRECT | GO:0005496~steroid binding | 3 | 5.41E-03 | 26.80 |
| F1 | GOTERM_MF_DIRECT | GO:0019838~growth factor binding | 3 | 5.41E-03 | 26.80 |
| F1 | GOTERM_MF_DIRECT | GO:0004714~transmembrane receptor protein tyrosine kinase activity | 4 | 4.98E-04 | 25.38 |
| F1 | GOTERM_MF_DIRECT | GO:0019825~oxygen binding | 4 | 9.32E-04 | 20.52 |
| F1 | GOTERM_MF_DIRECT | GO:0016705~oxidoreductase activity, acting on paired donors, with incorporation or reduction of molecular oxygen | 4 | 1.63E-03 | 16.92 |
| F1 | GOTERM_MF_DIRECT | GO:0004497~monooxygenase activity | 4 | 1.72E-03 | 16.63 |
| F1 | GOTERM_MF_DIRECT | GO:0020037~heme binding | 9 | 8.45E-08 | 15.84 |
| F1 | GOTERM_MF_DIRECT | GO:0004715~non-membrane spanning protein tyrosine kinase activity | 3 | 1.52E-02 | 15.73 |
| F1 | GOTERM_MF_DIRECT | GO:0004175~endopeptidase activity | 3 | 2.06E-02 | 13.40 |
| F1 | GOTERM_MF_DIRECT | GO:0004713~protein tyrosine kinase activity | 7 | 1.70E-05 | 12.69 |
| F1 | GOTERM_MF_DIRECT | GO:0005506~iron ion binding | 8 | 2.95E-06 | 12.61 |
| F1 | GOTERM_MF_DIRECT | GO:0019903~protein phosphatase binding | 3 | 2.74E-02 | 11.48 |
| F1 | GOTERM_MF_DIRECT | GO:0019899~enzyme binding | 12 | 9.81E-08 | 8.69 |
| F1 | GOTERM_MF_DIRECT | GO:0004222~metalloendopeptidase activity | 4 | 1.11E-02 | 8.54 |
| F1 | GOTERM_MF_DIRECT | GO:0016491~oxidoreductase activity | 6 | 1.35E-03 | 7.23 |
| F1 | GOTERM_MF_DIRECT | GO:0008289~lipid binding | 4 | 2.39E-02 | 6.39 |
| F1 | GOTERM_MF_DIRECT | GO:0016301~kinase activity | 6 | 3.04E-03 | 6.00 |
| F1 | GOTERM_MF_DIRECT | GO:0008022~protein C-terminus binding | 4 | 3.85E-02 | 5.30 |
| F1 | GOTERM_MF_DIRECT | GO:0043565~sequence-specific DNA binding | 11 | 4.54E-05 | 5.12 |
| F1 | GOTERM_MF_DIRECT | GO:0005215~transporter activity | 4 | 4.98E-02 | 4.78 |
| F1 | GOTERM_MF_DIRECT | GO:0004672~protein kinase activity | 7 | 3.45E-03 | 4.70 |
| F1 | GOTERM_MF_DIRECT | GO:0008134~transcription factor binding | 5 | 2.89E-02 | 4.25 |
| F1 | GOTERM_MF_DIRECT | GO:0005102~receptor binding | 6 | 1.47E-02 | 4.10 |
| F1 | GOTERM_MF_DIRECT | GO:0019901~protein kinase binding | 6 | 1.88E-02 | 3.85 |
| F1 | GOTERM_MF_DIRECT | GO:0003682~chromatin binding | 6 | 2.18E-02 | 3.70 |
| F1 | GOTERM_MF_DIRECT | GO:0008270~zinc ion binding | 17 | 1.46E-05 | 3.51 |
| F1 | GOTERM_MF_DIRECT | GO:0005524~ATP binding | 17 | 2.75E-04 | 2.74 |
| F1 | GOTERM_MF_DIRECT | GO:0042802~identical protein binding | 8 | 3.29E-02 | 2.58 |
| F1 | GOTERM_MF_DIRECT | GO:0003700~transcription factor activity, sequence-specific DNA binding | 9 | 4.16E-02 | 2.26 |
| F1 | GOTERM_MF_DIRECT | GO:0003677~DNA binding | 13 | 3.76E-02 | 1.87 |
| F1 | GOTERM_MF_DIRECT | GO:0005515~protein binding | 46 | 1.84E-02 | 1.26 |
| F2 | GOTERM_MF_DIRECT | GO:0003708~retinoic acid receptor activity | 3 | 3.03E-04 | 106.62 |
| F2 | GOTERM_MF_DIRECT | GO:0050544~arachidonic acid binding | 3 | 3.03E-04 | 106.62 |
| F2 | GOTERM_MF_DIRECT | GO:0004115~3',5'-cyclic-AMP phosphodiesterase activity | 4 | 7.25E-05 | 47.39 |
| F2 | GOTERM_MF_DIRECT | GO:0046965~retinoid X receptor binding | 4 | 7.25E-05 | 47.39 |
| F2 | GOTERM_MF_DIRECT | GO:0005542~folic acid binding | 3 | 2.30E-03 | 41.01 |
| F2 | GOTERM_MF_DIRECT | GO:0003707~steroid hormone receptor activity | 12 | 1.06E-14 | 38.08 |
| F2 | GOTERM_MF_DIRECT | GO:0016712~oxidoreductase activity, acting on paired donors, with incorporation or reduction of molecular oxygen, reduced flavin or flavoprotein as one donor, and incorporation of one atom of oxygen | 3 | 3.07E-03 | 35.54 |
| F2 | GOTERM_MF_DIRECT | GO:0004697~protein kinase C activity | 3 | 3.07E-03 | 35.54 |
| F2 | GOTERM_MF_DIRECT | GO:0010181~FMN binding | 3 | 3.07E-03 | 35.54 |
| F2 | GOTERM_MF_DIRECT | GO:0004114~3',5'-cyclic-nucleotide phosphodiesterase activity | 4 | 2.73E-04 | 30.90 |
| F2 | GOTERM_MF_DIRECT | GO:0004879~RNA polymerase II transcription factor activity, ligand-activated sequence-specific DNA binding | 6 | 1.58E-06 | 29.62 |
| F2 | GOTERM_MF_DIRECT | GO:0008144~drug binding | 12 | 3.72E-13 | 28.06 |
| F2 | GOTERM_MF_DIRECT | GO:0008395~steroid hydroxylase activity | 4 | 3.96E-04 | 27.34 |
| F2 | GOTERM_MF_DIRECT | GO:0005496~steroid binding | 4 | 4.44E-04 | 26.33 |
| F2 | GOTERM_MF_DIRECT | GO:0030552~cAMP binding | 3 | 7.82E-03 | 22.21 |
| F2 | GOTERM_MF_DIRECT | GO:0003785~actin monomer binding | 3 | 9.14E-03 | 20.50 |
| F2 | GOTERM_MF_DIRECT | GO:0019838~growth factor binding | 3 | 9.84E-03 | 19.74 |
| F2 | GOTERM_MF_DIRECT | GO:0070330~aromatase activity | 3 | 9.84E-03 | 19.74 |
| F2 | GOTERM_MF_DIRECT | GO:0004715~non-membrane spanning protein tyrosine kinase activity | 5 | 1.23E-04 | 19.31 |
| F2 | GOTERM_MF_DIRECT | GO:0019825~oxygen binding | 5 | 1.34E-04 | 18.90 |
| F2 | GOTERM_MF_DIRECT | GO:0004714~transmembrane receptor protein tyrosine kinase activity | 4 | 1.22E-03 | 18.70 |
| F2 | GOTERM_MF_DIRECT | GO:0004497~monooxygenase activity | 6 | 1.75E-05 | 18.38 |
| F2 | GOTERM_MF_DIRECT | GO:0016705~oxidoreductase activity, acting on paired donors, with incorporation or reduction of molecular oxygen | 5 | 2.84E-04 | 15.59 |
| F2 | GOTERM_MF_DIRECT | GO:0050661~NADP binding | 3 | 1.62E-02 | 15.23 |
| F2 | GOTERM_MF_DIRECT | GO:0020037~heme binding | 11 | 4.51E-09 | 14.27 |
| F2 | GOTERM_MF_DIRECT | GO:0005506~iron ion binding | 12 | 8.55E-10 | 13.94 |
| F2 | GOTERM_MF_DIRECT | GO:0001046~core promoter sequence-specific DNA binding | 3 | 2.39E-02 | 12.40 |
| F2 | GOTERM_MF_DIRECT | GO:0042626~ATPase activity, coupled to transmembrane movement of substances | 3 | 2.49E-02 | 12.12 |
| F2 | GOTERM_MF_DIRECT | GO:0004713~protein tyrosine kinase activity | 9 | 7.57E-07 | 12.02 |
| F2 | GOTERM_MF_DIRECT | GO:0005080~protein kinase C binding | 3 | 2.71E-02 | 11.59 |
| F2 | GOTERM_MF_DIRECT | GO:0019903~protein phosphatase binding | 4 | 5.22E-03 | 11.28 |
| F2 | GOTERM_MF_DIRECT | GO:0097110~scaffold protein binding | 3 | 2.93E-02 | 11.11 |
| F2 | GOTERM_MF_DIRECT | GO:0004175~endopeptidase activity | 3 | 3.64E-02 | 9.87 |
| F2 | GOTERM_MF_DIRECT | GO:0050839~cell adhesion molecule binding | 3 | 4.68E-02 | 8.60 |
| F2 | GOTERM_MF_DIRECT | GO:0019899~enzyme binding | 13 | 3.20E-07 | 6.94 |
| F2 | GOTERM_MF_DIRECT | GO:0004222~metalloendopeptidase activity | 4 | 2.52E-02 | 6.29 |
| F2 | GOTERM_MF_DIRECT | GO:0016491~oxidoreductase activity | 7 | 8.80E-04 | 6.22 |
| F2 | GOTERM_MF_DIRECT | GO:0005088~Ras guanyl-nucleotide exchange factor activity | 4 | 2.64E-02 | 6.18 |
| F2 | GOTERM_MF_DIRECT | GO:0004672~protein kinase activity | 11 | 3.09E-05 | 5.44 |
| F2 | GOTERM_MF_DIRECT | GO:0043565~sequence-specific DNA binding | 13 | 2.98E-05 | 4.46 |
| F2 | GOTERM_MF_DIRECT | GO:0005215~transporter activity | 5 | 2.63E-02 | 4.40 |
| F2 | GOTERM_MF_DIRECT | GO:0008134~transcription factor binding | 7 | 5.09E-03 | 4.38 |
| F2 | GOTERM_MF_DIRECT | GO:0000287~magnesium ion binding | 5 | 2.72E-02 | 4.36 |
| F2 | GOTERM_MF_DIRECT | GO:0004872~receptor activity | 5 | 3.30E-02 | 4.09 |
| F2 | GOTERM_MF_DIRECT | GO:0005102~receptor binding | 8 | 3.56E-03 | 4.03 |
| F2 | GOTERM_MF_DIRECT | GO:0008270~zinc ion binding | 25 | 1.56E-08 | 3.80 |
| F2 | GOTERM_MF_DIRECT | GO:0004674~protein serine/threonine kinase activity | 8 | 5.01E-03 | 3.78 |
| F2 | GOTERM_MF_DIRECT | GO:0003713~transcription coactivator activity | 5 | 4.98E-02 | 3.58 |
| F2 | GOTERM_MF_DIRECT | GO:0005524~ATP binding | 23 | 1.83E-05 | 2.73 |
| F2 | GOTERM_MF_DIRECT | GO:0046982~protein heterodimerization activity | 7 | 4.53E-02 | 2.67 |
| F2 | GOTERM_MF_DIRECT | GO:0042803~protein homodimerization activity | 10 | 2.03E-02 | 2.43 |
| F2 | GOTERM_MF_DIRECT | GO:0003700~transcription factor activity, sequence-specific DNA binding | 12 | 1.77E-02 | 2.22 |
| F2 | GOTERM_MF_DIRECT | GO:0003677~DNA binding | 16 | 4.33E-02 | 1.70 |
| F2 | GOTERM_MF_DIRECT | GO:0005515~protein binding | 64 | 2.26E-03 | 1.29 |
| F3 | GOTERM_MF_DIRECT | GO:0003708~retinoic acid receptor activity | 3 | 2.37E-04 | 120.58 |
| F3 | GOTERM_MF_DIRECT | GO:0004115~3',5'-cyclic-AMP phosphodiesterase activity | 4 | 5.00E-05 | 53.59 |
| F3 | GOTERM_MF_DIRECT | GO:0046965~retinoid X receptor binding | 4 | 5.00E-05 | 53.59 |
| F3 | GOTERM_MF_DIRECT | GO:0016712~oxidoreductase activity, acting on paired donors, with incorporation or reduction of molecular oxygen, reduced flavin or flavoprotein as one donor, and incorporation of one atom of oxygen | 3 | 2.41E-03 | 40.19 |
| F3 | GOTERM_MF_DIRECT | GO:0003707~steroid hormone receptor activity | 10 | 6.75E-12 | 35.89 |
| F3 | GOTERM_MF_DIRECT | GO:0004114~3',5'-cyclic-nucleotide phosphodiesterase activity | 4 | 1.89E-04 | 34.95 |
| F3 | GOTERM_MF_DIRECT | GO:0008395~steroid hydroxylase activity | 4 | 2.75E-04 | 30.92 |
| F3 | GOTERM_MF_DIRECT | GO:0004714~transmembrane receptor protein tyrosine kinase activity | 5 | 3.53E-05 | 26.44 |
| F3 | GOTERM_MF_DIRECT | GO:0030552~cAMP binding | 3 | 6.15E-03 | 25.12 |
| F3 | GOTERM_MF_DIRECT | GO:0008144~drug binding | 9 | 3.48E-09 | 23.80 |
| F3 | GOTERM_MF_DIRECT | GO:0003785~actin monomer binding | 3 | 7.19E-03 | 23.19 |
| F3 | GOTERM_MF_DIRECT | GO:0019838~growth factor binding | 3 | 7.74E-03 | 22.33 |
| F3 | GOTERM_MF_DIRECT | GO:0070330~aromatase activity | 3 | 7.74E-03 | 22.33 |
| F3 | GOTERM_MF_DIRECT | GO:0005496~steroid binding | 3 | 7.74E-03 | 22.33 |
| F3 | GOTERM_MF_DIRECT | GO:0004715~non-membrane spanning protein tyrosine kinase activity | 5 | 7.58E-05 | 21.84 |
| F3 | GOTERM_MF_DIRECT | GO:0019825~oxygen binding | 5 | 8.25E-05 | 21.38 |
| F3 | GOTERM_MF_DIRECT | GO:0097110~scaffold protein binding | 5 | 8.97E-05 | 20.93 |
| F3 | GOTERM_MF_DIRECT | GO:0004497~monooxygenase activity | 6 | 9.50E-06 | 20.79 |
| F3 | GOTERM_MF_DIRECT | GO:0016705~oxidoreductase activity, acting on paired donors, with incorporation or reduction of molecular oxygen | 5 | 1.76E-04 | 17.63 |
| F3 | GOTERM_MF_DIRECT | GO:0020037~heme binding | 12 | 6.58E-11 | 17.60 |
| F3 | GOTERM_MF_DIRECT | GO:0004879~RNA polymerase II transcription factor activity, ligand-activated sequence-specific DNA binding | 3 | 1.35E-02 | 16.75 |
| F3 | GOTERM_MF_DIRECT | GO:0030331~estrogen receptor binding | 3 | 1.42E-02 | 16.29 |
| F3 | GOTERM_MF_DIRECT | GO:0004713~protein tyrosine kinase activity | 10 | 1.79E-08 | 15.11 |
| F3 | GOTERM_MF_DIRECT | GO:0005506~iron ion binding | 11 | 3.84E-09 | 14.45 |
| F3 | GOTERM_MF_DIRECT | GO:0005080~protein kinase C binding | 3 | 2.15E-02 | 13.11 |
| F3 | GOTERM_MF_DIRECT | GO:0004175~endopeptidase activity | 3 | 2.90E-02 | 11.16 |
| F3 | GOTERM_MF_DIRECT | GO:0042826~histone deacetylase binding | 5 | 1.60E-03 | 9.85 |
| F3 | GOTERM_MF_DIRECT | GO:0001618~virus receptor activity | 3 | 4.65E-02 | 8.61 |
| F3 | GOTERM_MF_DIRECT | GO:0019899~enzyme binding | 13 | 7.87E-08 | 7.85 |
| F3 | GOTERM_MF_DIRECT | GO:0004222~metalloendopeptidase activity | 4 | 1.82E-02 | 7.11 |
| F3 | GOTERM_MF_DIRECT | GO:0016491~oxidoreductase activity | 7 | 4.54E-04 | 7.03 |
| F3 | GOTERM_MF_DIRECT | GO:0004672~protein kinase activity | 11 | 1.02E-05 | 6.16 |
| F3 | GOTERM_MF_DIRECT | GO:0005215~transporter activity | 5 | 1.76E-02 | 4.97 |
| F3 | GOTERM_MF_DIRECT | GO:0000287~magnesium ion binding | 5 | 1.81E-02 | 4.93 |
| F3 | GOTERM_MF_DIRECT | GO:0004872~receptor activity | 5 | 2.22E-02 | 4.63 |
| F3 | GOTERM_MF_DIRECT | GO:0005102~receptor binding | 8 | 1.75E-03 | 4.55 |
| F3 | GOTERM_MF_DIRECT | GO:0043565~sequence-specific DNA binding | 11 | 2.21E-04 | 4.27 |
| F3 | GOTERM_MF_DIRECT | GO:0008134~transcription factor binding | 6 | 1.30E-02 | 4.25 |
| F3 | GOTERM_MF_DIRECT | GO:0016301~kinase activity | 5 | 3.10E-02 | 4.17 |
| F3 | GOTERM_MF_DIRECT | GO:0003713~transcription coactivator activity | 5 | 3.39E-02 | 4.05 |
| F3 | GOTERM_MF_DIRECT | GO:0008270~zinc ion binding | 21 | 6.70E-07 | 3.61 |
| F3 | GOTERM_MF_DIRECT | GO:0004674~protein serine/threonine kinase activity | 6 | 3.78E-02 | 3.21 |
| F3 | GOTERM_MF_DIRECT | GO:0003682~chromatin binding | 6 | 4.35E-02 | 3.08 |
| F3 | GOTERM_MF_DIRECT | GO:0005524~ATP binding | 21 | 2.76E-05 | 2.82 |
| F3 | GOTERM_MF_DIRECT | GO:0042803~protein homodimerization activity | 9 | 2.69E-02 | 2.48 |
| F3 | GOTERM_MF_DIRECT | GO:0005515~protein binding | 57 | 3.09E-03 | 1.30 |
